# Optimization of Class I Histone Deacetylase PROTACs Reveals that HDAC1/2 Degradation is Critical to Induce Apoptosis and Cell Arrest in Cancer Cells

**DOI:** 10.1021/acs.jmedchem.1c02179

**Published:** 2022-03-16

**Authors:** Joshua P. Smalley, India M. Baker, Wiktoria A. Pytel, Li-Ying Lin, Karen J. Bowman, John W. R. Schwabe, Shaun M. Cowley, James T. Hodgkinson

**Affiliations:** Leicester Institute of Structural and Chemical Biology, School of Chemistry, University of Leicester, Leicester LE1 7RH, U.K; Department of Molecular and Cell Biology, University of Leicester, Leicester LE1 7RH, U.K; Leicester Institute of Structural and Chemical Biology, School of Chemistry, University of Leicester, Leicester LE1 7RH, U.K; Leicester Institute of Structural and Chemical Biology, Department of Molecular and Cell Biology, University of Leicester, Leicester LE1 7RH, U.K; Department of Molecular and Cell Biology, University of Leicester, Leicester LE1 7RH, U.K; Leicester Institute of Structural and Chemical Biology, Department of Molecular and Cell Biology, University of Leicester, Leicester LE1 7RH, U.K; Department of Molecular and Cell Biology, University of Leicester, Leicester LE1 7RH, U.K; Leicester Institute of Structural and Chemical Biology, School of Chemistry, University of Leicester, Leicester LE1 7RH, U.K

## Abstract

Class I histone deacetylase (HDAC) enzymes 1, 2, and 3 organize chromatin as the catalytic subunits within seven distinct multiprotein corepressor complexes and are established drug targets. We report optimization studies of benzamide-based Von Hippel−Lindau (VHL) E3-ligase proteolysis targeting chimeras (PROTACs) and for the first time describe transcriptome perturbations resulting from these degraders. By modifying the linker and VHL ligand, we identified PROTACs **7, 9**, and **22** with submicromolar DC_50_ values for HDAC1 and/or HDAC3 in HCT116 cells. A hook effect was observed for HDAC3 that could be negated by modifying the position of attachment of the VHL ligand to the linker. The more potent HDAC1/2 degraders correlated with greater total differentially expressed genes and enhanced apoptosis in HCT116 cells. We demonstrate that HDAC1/2 degradation by PROTACs correlates with enhanced global gene expression and apoptosis, important for the development of more efficacious HDAC therapeutics with reduced side effects.

## Graphical abstract

**Figure:**
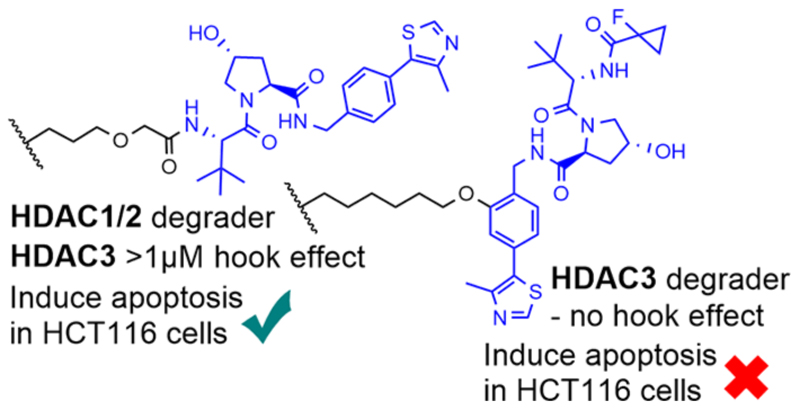

## Introduction

Class I histone deacetylase (HDAC) enzymes, HDAC1, 2, 3, and 8, are four out of eleven zinc-dependent HDAC enzymes, catalyzing the hydrolysis of acetyl groups in *N*-*ε*-acetyl-L-lysine residues in histones and nonhistone proteins.^1^ HDAC1/2 shares over 80% sequence homology, is localized in the nucleus, and exists in several multiprotein corepressor complexes including Sin3, CoREST, MiDAC, and NuRD.^1,2^ HDAC3 shares approximately 50% sequence homology with HDAC1/2, is also predominantly localized in the nucleus, and exists exclusively in the SMRT/NCoR corepressor complex.^1,3^ HDAC8, in contrast to HDAC1/2 and 3, can be found in both the nucleus and cytoplasm and is not present in corepressor complexes.^1,4^

Four HDAC inhibitors (HDACi) have been approved by the US FDA including the hydroxamic acids vorinostat, panobinostat, and belinostat and the cyclic peptide natural product romidepsin. These drugs are primarily used for the treatment of hematological cancers, with other HDACi currently in clinical trials. The approved hydroxamic acid HDACi drugs chelate Zn^2+^ in the eleven zinc-dependent HDAC enzymes, and despite being potent, HDACi generally exhibits limited selectivity between isoforms.^5^ The disulfide prodrug romidepsin lacks selectivity between HDAC1, 2, 3, 10, and 11.^5^ A lack of HDAC isoform selectivity among approved HDAC drugs might contribute to the undesired side effects associated with these drugs.^6−8^ Additionally, it has also been proposed that the rearrangement of the hydroxamic acid functional group present in many HDACi drugs to an isocyanate can lead to mutagenicity.^9^

Toward more efficacious HDAC therapeutics with reduced side effects, a number of studies have demonstrated that the selective targeting of HDAC1/2 and/or HDAC3 may be advantageous for specific diseases.^10−15^ For example, selective inhibitors of HDAC1/2 were more effective at inducing apoptosis in B-cell acute lymphoblastic leukemia compared to other B-cell malignances,^10^ while cutaneous T-cell lymphoma cell lines exhibited enhanced sensitivity to an HDAC3 selective inhibitor.^13^ Additionally, each of the individual corepressor complexes that incorporate HDAC1/2 and 3 has a distinct cellular function, and therefore, the selective targeting of individual complexes may have potential therapeutic benefits in differing clinical applications.^16,17^

**Figure 1:**
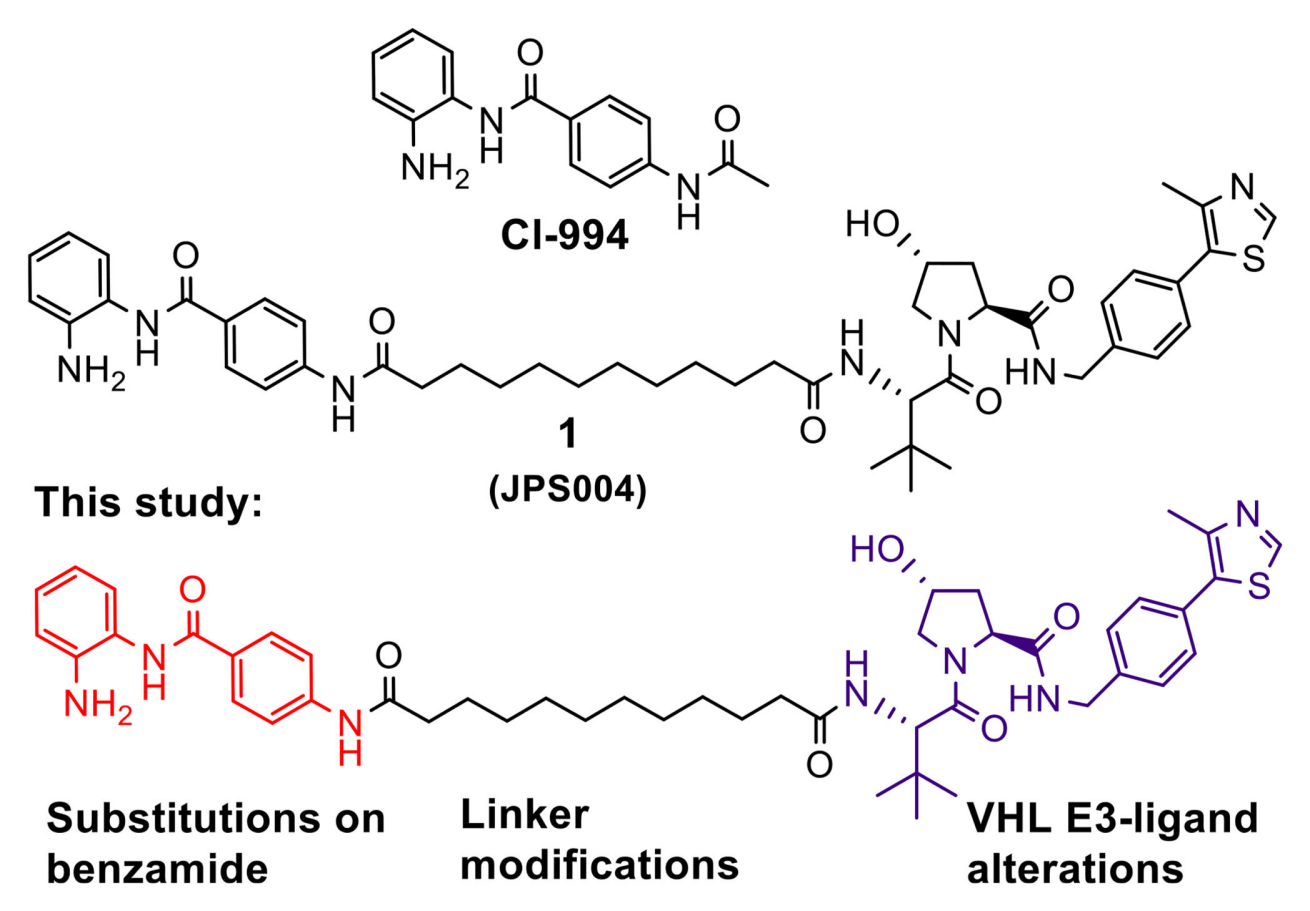
CI-994—HDAC1-, 2-, and 3-selective inhibitor. **1** (JPS004)—HDAC1, 2, and 3 protein degrader.^18^ This study; optimization studies of **1** (JPS004) on HDAC1, 2, and 3 degradation; and effects on global gene expression.

**Chart 1:**
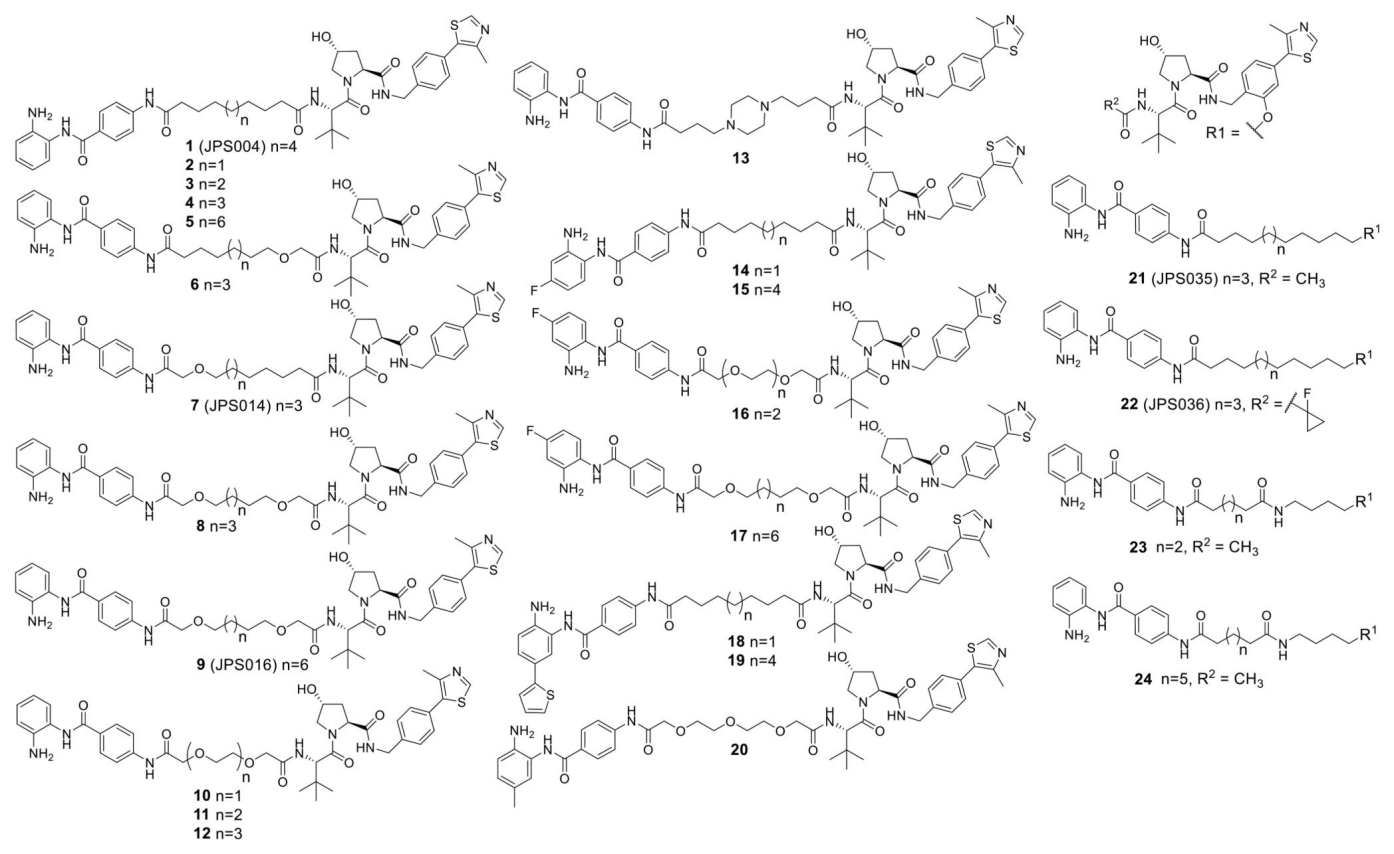
Compound Library Tested in This Study.

Investigating novel approaches to target HDAC1/2 and 3, we previously reported benzamide-based Von Hippel−Lindau (VHL) E3-ligase proteolysis targeting chimeras (PROTACs) as an alternative strategy to degrade, rather than inhibit, enzyme activity.^18^ PROTACs consist of a ligand for the protein of interest (POI), an E3-ligase ligand, and a linker that covalently bonds the two ligands.^19^ PROTACs recruit the endogenous ubiquitination machinery via the E3-ligase to polyubiquitinate the POI, tagging it for degradation by the proteasome.^20,21^ PROTAC **1** (JPS004) was based on the benzamide inhibitor CI-994, which exhibits selectivity for HDAC1/2 and 3 (Figure 1).^18^ We discovered a dependence on the linker length for HDAC1/2 and 3 degradation. Alkyl linkers consisting of 12 carbon atoms resulted in HDAC1/2 and 3 degradation in HCT116 colon cancer cells, while alkyl linkers of 6 carbon atoms, although inhibiting the HDAC1/CoREST complex in vitro, showed no activity in cells. The VHL E3-ligase ligand,^21^ in combination with the 12-atom alkyl linker, resulted in the most effective degradation. We wanted to carry out optimization studies of **1** with the aim of discovering novel PROTACs with enhanced degradation of HDAC1/2 and 3 and with differing selectivity profiles between these enzymes, allowing us to study the effects of removing these enzymes from the cell via proteasome-mediated degradation. To achieve this, we synthesized 23 novel heterobifunctional molecules making rationalized modifications to the benzamide, linker, and VHL E3-ligase ligand components (Chart 1). As HDAC1/2 and 3 also play an important role in the chromatin structure and transcription, for the first time, we also wanted to test the ability of such PROTACs to regulate global gene expression.

## Results

**Figure 2:**
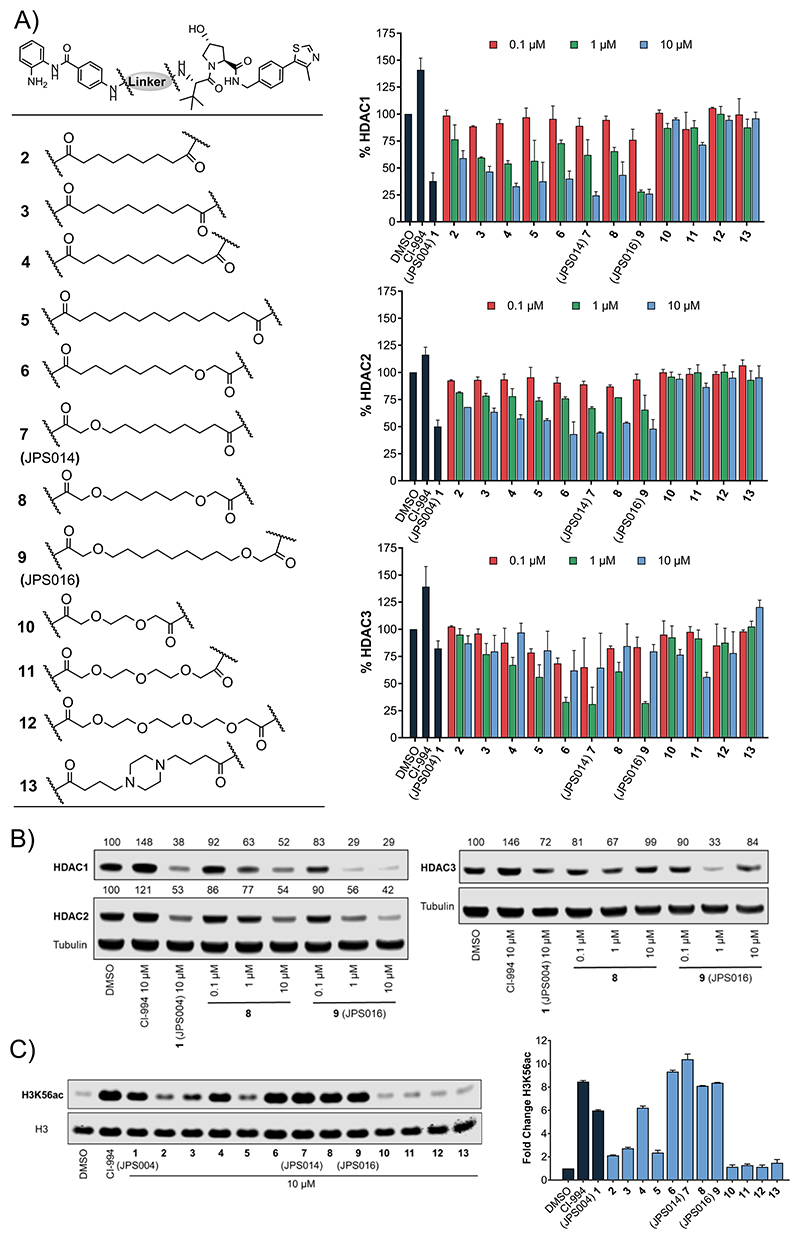
(A) Compounds **2**−**13** were screened at 0.1, 1.0, and 10 *μ*M with HDAC1, 2, and 3 abundance determined by quantitative western blotting with specific antibodies to HDAC1, 2, and 3 in HCT116 cells. CI-994 and **1** (JPS004) were also included at 10 *μ*M. Error bars represent the standard deviation of two independent biological replicates. Statistical analysis of the significance of degradation for **1, 7**, and **9** can be found in the Supporting Information (Figure S7). (B) Representative western blots demonstrating degradation by **8** and **9** (JPS016). (C) H3K56ac blot and fold change at 10 *μ*M. Error bars represent the average of two independent biological replicates.

We first wanted to investigate the effects of the PROTAC linker length and composition on their ability to induce HDAC1/2 and 3 degradation. PROTACs were synthesized with alkyl linkers, alkyl linkers incorporating one or two oxygen atoms, poly ethylene glycol (PEG) linkers, and a piperazine substituted linker, with lengths ranging from 8 to 15 atoms (Figure 2) (see the Experimental Section and the Supporting Information). Initially, each PROTAC was tested at 0.1, 1, and 10 *μ*M in HCT116 cells for 24 h; then, cell extracts were prepared and evaluated for HDAC1/2 and 3 degradation by quantitative western blotting. For direct comparison, novel PROTACs were screened side by side with our original PROTAC **1** at a concentration of 10 *μ*M, which previously caused maximum HDAC1/2 and 3 degradation (Figure 2) (blots available in Supporting Information Figure S1). To examine their ability to engage with HDAC enzymes in cells, we measured the ability of novel PROTACs to regulate levels of histone H3 Lys 56 acetylation (H3K56ac), a known HDAC1/2 substrate,^22^ which also provides an indirect indication of PROTAC cell permeability (blots available in Supporting Information Figure S2).

There was a stepwise increase in HDAC1 degradation with increasing alkyl linker length from 9 to 11 carbon atoms (**2, 3**, and **4**) at 1 and 10 *μ*M (Figure 2A). The 11-atom linker, **4**, exhibited HDAC1 degradation levels directly comparable to those of the 12-atom linker **1** at 10 *μ*M. The same trend was also observed for HDAC2 with increasing alkyl linker length (**2, 3**, and **4**); however, overall HDAC2 degradation was less pronounced in comparison to that of HDAC1. HDAC3 levels for **2, 3**, and **4** were not greatly reduced with these subtle changes in linker length. H3K56ac levels also increased with increasing linker length (Figure 2C—compare **2, 3**, and **4**), suggesting increased cell permeability and/or HDAC engagement with increasing linker length. The 11-atom linker **4** increased H3K56ac levels to the same degree as the 12-atom linker **1** (Figure 2C). The 14-atom alkyl linker **5** exhibited comparable HDAC1 and HDAC2 degradation to the shorter linkers (**1, 3**, and **4**); however, there was only a modest increase in H3K56ac compared to the shorter linkers, and we also noted solubility issues with **5**.

The incorporation of one oxygen atom into 12-atom linkers 6 and **7** (JPS014) resulted in HDAC1 and HDAC2 degradation comparable to that of **1** and even enhanced for 7 at 10 *μ*M, while HDAC3 degradation for both these PROTACs was also significantly enhanced compared to that of **1** surprisingly with the greater HDAC3 degradation at the lower concertation of 1 *μ*M. Compounds **6** and **7** also increased H3K56ac to comparable or greater levels than CI-994 and **1**.

The incorporation of two oxygen atoms into a 12-atom linker, **8**, resulted in a loss of HDAC3 degradation compared to **6** and **7**, while HDAC1 degradation was comparable to that of **1** but not maintained at 1 *μ*M. However, H3K56ac levels for **8** matched those of CI-994, suggesting that this molecule, while not an effective degrader as other compounds in the library, can still act as a class I HDACi. Incorporating 2 oxygen atoms into a 15-atom linker, **9** (JPS016), resulted in enhanced degradation levels compared to those of **1** for both HDAC1 and HDAC3 even at 1 *μ*M, while HDAC2 degradation was marginally increased compared to **1** at 10 *μ*M (Figure 2A,B). This degradation was mirrored with increased H3K56ac levels to the same levels as CI-994 (Figure 2C).

The compounds that incorporated PEG linkers, **10, 11**, and **12**, or a piperazine, **13**, resulted in an almost complete loss of HDAC1/2 degradation; HDAC3 degradation was also generally compromised. Additionally, compounds **10, 11, 12**, and **13** did not increase H3K56ac levels compared to the dimethyl sulfoxide (DMSO) control, suggesting that in HCT116 cells, these compounds do not act as degraders or inhibitors; we speculate that these compounds may not be reaching their class I HDAC targets in the nucleus. Overall, PROTACs **7** and **9** enhanced degradation compared to **1**, with **9** showing enhanced degradation for HDAC1 and HDAC3 at 1 *μ*M.

**Figure 3:**
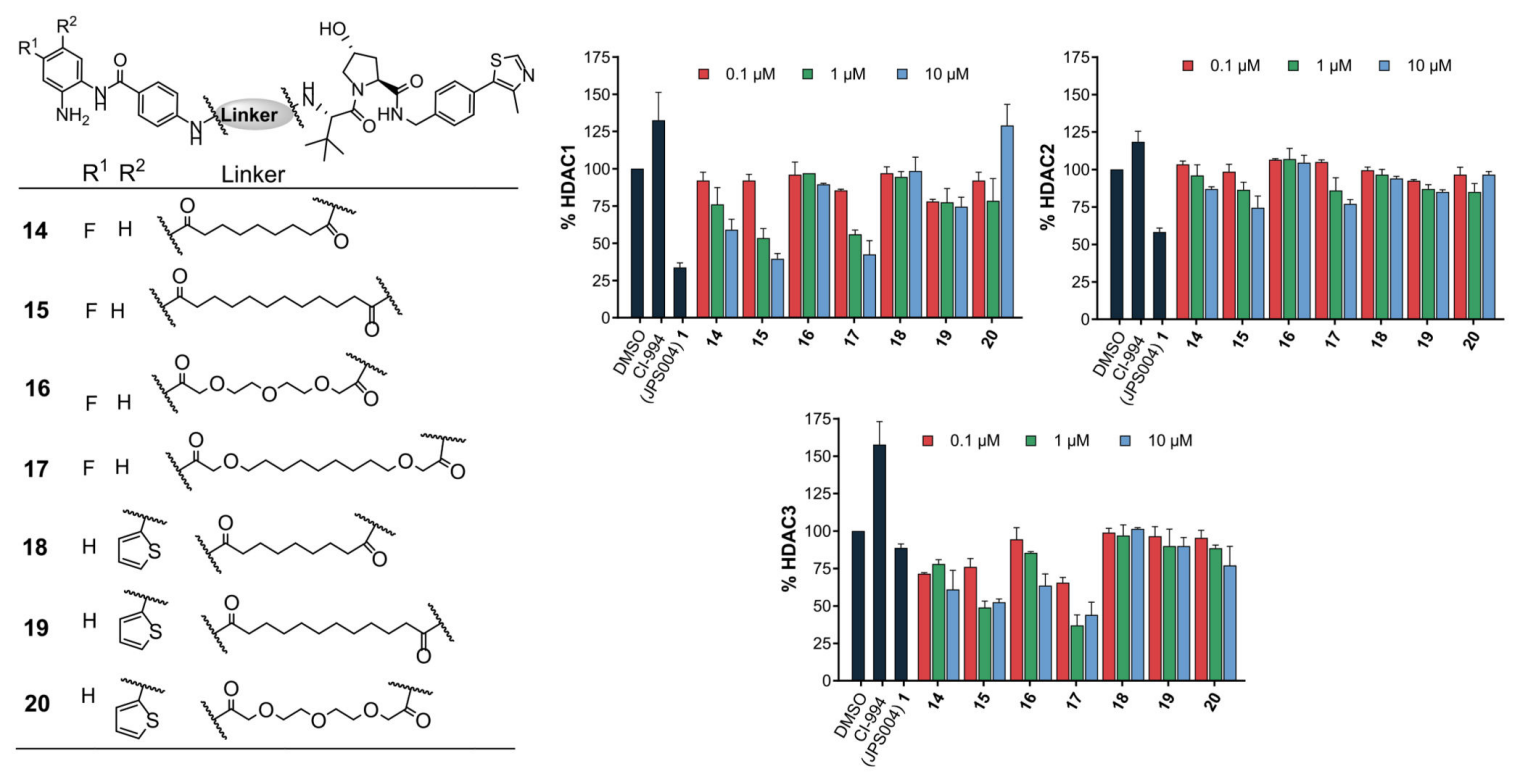
Compounds **14**−**20** were screened at 0.1, 1.0, and 10 *μ*M with HDAC1, 2, and 3 abundance determined by quantitative western blotting with specific antibodies to HDAC1, 2, and 3 in HCT116 cells. CI-994 and **1** (JPS004) also included at 10 *μ*M. Error bars represent the standard deviation of two independent biological replicates.

We next sought to investigate substitutions on the benzamide HDAC ligand of the PROTAC as it has been previously reported that substitutions with a fluorine atom on the 4-position of the anilide can increase selectivity for HDAC3,^23,24^ while the introduction of a thiophene heterocycle on the 5-position of the anilide can enhance HDAC1/2 inhibitory potency and selectivity.^25^ The 12-carbon linker with a fluorine atom, **15**, directly analogous to **1**, exhibited enhanced HDAC3 degradation compared to **1**; however, despite this increase, HDAC1 degradation was still marginally elevated over HDAC3 at 10 *μ*M (Figure 3). For the 15-atom linker, **17**, HDAC3 degradation was also enhanced at 1 *μ*M compared to **1** and degradation levels for HDAC3 were now greater than those of HDAC1 and HDAC2; however, significant HDAC1 degradation was also still observed at 1 *μ*M. The remaining fluorine-functionalized molecules **14** and **16** exhibited no gains in HDAC3 selectivity, with the PEG linker **16** exhibiting only modest HDAC3 degradation at 10 *μ*M. Compounds **14**−**17** did not increase H3K56ac to the same levels as **1** or CI-994, with only **17** exhibiting a greater than twofold increase in H3K56ac compared to the DMSO control (see Supporting Information Figure S2).

Introduction of the thiophene moiety unfortunately did not result in enhanced degradation potency of HDAC1/2 in **18, 19**, or **20**. However, **20**, with the PEG linker, did increase H3K56ac to levels similar to **1**, suggesting that this molecule can act as an inhibitor (Figure S2). Apart from the PEG linker analogue, **20**, we also noted that the thiophene-substituted analogues exhibited exceptionally poor aqueous solubility. Overall, aside from a modest enhancement in HDAC3 degradation levels comparatively to HDAC1 and HDAC2 with **17**, substitutions on the benzamide did not influence degradation selectivity or potency greatly. This perhaps suggests that formation of the ternary complex between the HDAC and VHL E3-ligase is more important for degradation than the affinity of the HDAC ligand in the PROTAC, which has been reported in other PROTACs also utilizing lower affinity ligands for the POI.^26,27^

**Figure 4:**
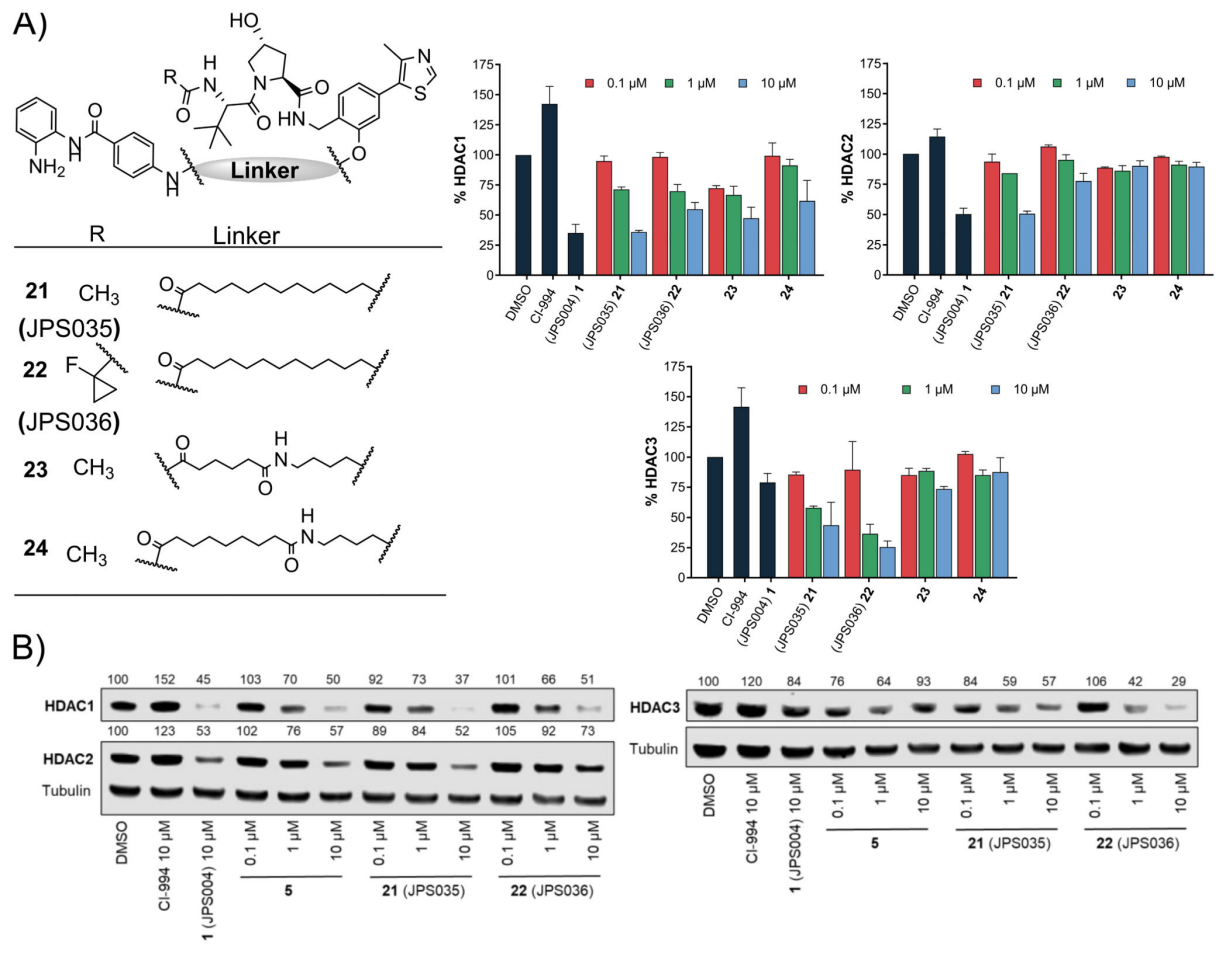
(A) Compounds **21**−**24** were screened at 0.1, 1.0, and 10 *μ*M with HDAC1, 2, and 3 abundance determined by quantitative western blotting with specific antibodies to HDAC1, 2, and 3 in HCT116 cells. CI-994 and **1** (JPS004) also included at 10 *μ*M. Error bars represent the standard deviation of two independent biological replicates. Statistical analysis of the significance of degradation for **21** and **22** can be found in the Supporting Information (Figure S7). (B) Representative western blots demonstrating degradation by **5, 21** (JPS016), and **22** (JPS036).

We wanted to investigate modifying the VHL E3-ligand as it had been previously shown that modifying the VHL-E3 ligand connectivity to the linker can modify the degradation selectivity profile of the PROTAC overall (Figure 4).^26^ At 10 *μ*M, **21** (JPS035) exhibited comparable HDAC1 and HDAC2 degradation to **1**, while **22** (JPS036) exhibited a reduction in HDAC1 and HDAC2 degradation compared to **1**. However, in addition, the fluorinated cyclopropane VHL analogue, **22**, reported to have higher affinity for VHL-E3 ligase than the acetyl VHL analogue in **21**,^28^ exhibited significantly enhanced HDAC3 degradation compared to **1** at both 1 and 10 *μ*M. This may suggest that recruitment of the VHL E3-ligase with **22** is more favorable toward forming a ternary complex with HDAC3 over HDAC1/2. Compound **21** increased H3K56ac levels significantly but not to the same levels as **1**, while the more prominent HDAC3 degrader **22** did not alter H3K56ac levels (Figure S2). Analogues **23** and **24** exhibited only modest degradation of HDAC1, and these compounds did not increase H3K56ac levels greater than the DMSO control (Figure S2).

Physiochemical property predictions of **1**−**24** were calculated using SwissADME^29^ and compared with the maximal degradation values observed for HDAC1, HDAC2, and HDAC3 with **1**−**24** (Table S1). The majority of molecules that exhibited ≥50% maximal degradation of either HDAC1, HDAC2, or HDAC3 had a clog*P* of ≥ 5.0 and topological polar surface area (TPSA) values of ≤ 242.6 Å^2^ with **8** and **23** being the only exceptions. The remaining molecules (exhibiting less than 50% maximal degradation of HDAC1, HDAC2, or HDAC3) had clog*P* values of < 5.0 with four exceptions, three of these exceptions exhibiting TPSA values > 242.6 Å^2^. Overall, in designing future class I HDAC PROTACs, in terms of physiochemical properties, maintaining a clog*P* of ≥ 5.0 and TPSA of ≤ 242.6 Å^2^ may serve as potential guidelines.

**Figure 5:**
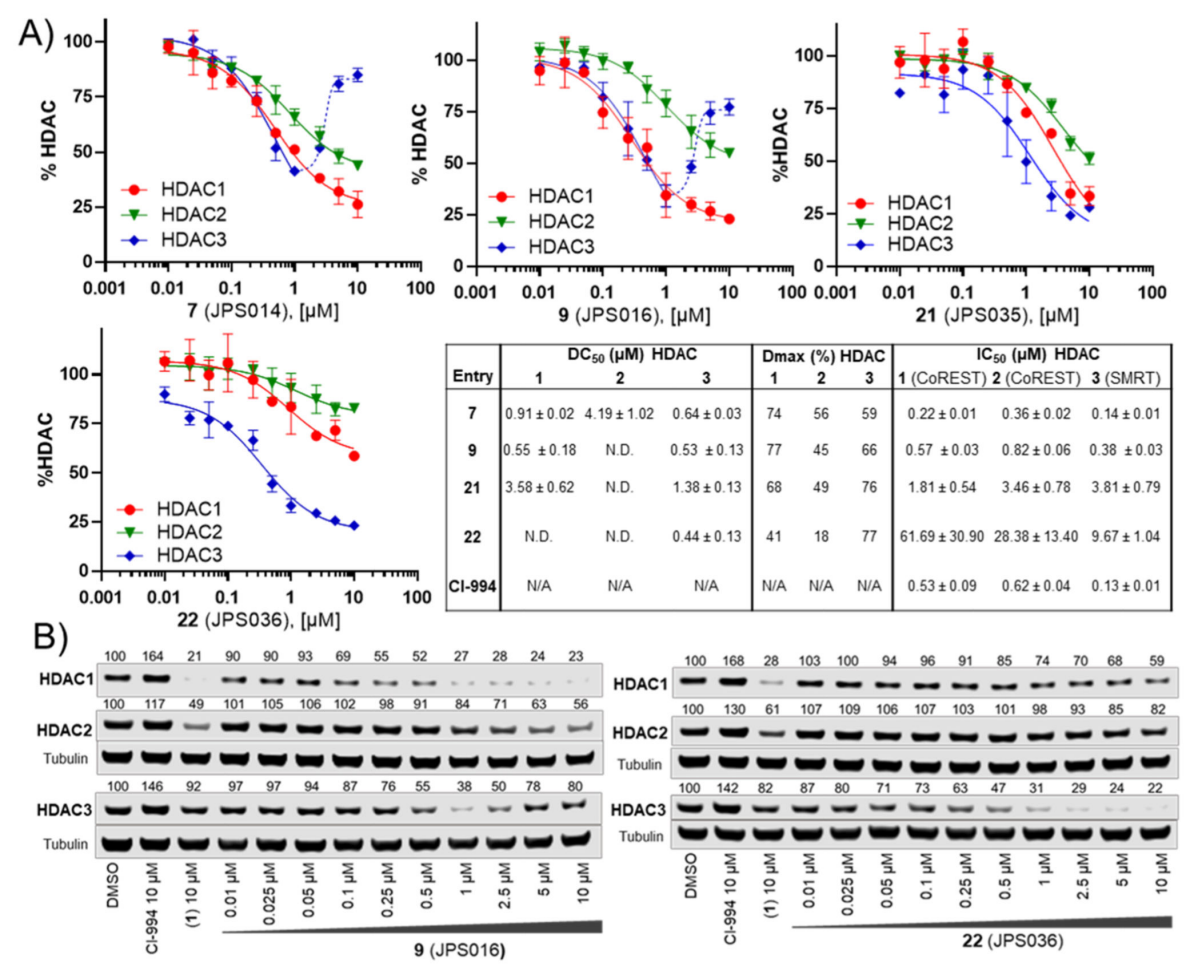
(A) Dose response curves, DC_50_ and Dmax calculations for compounds **7** (JPS014), **9** (JPS016), **21** (JPS035), and **22** (JPS036) in HCT116 cells after 24 h by quantitative western blotting. IC_50_ values also determined with the HDAC1-CoREST-LSD1, HDAC2-CoREST-LSD1, and HDAC3-SMRT complexes (see Supporting Information Figure S8). DC_50_ and Dmax values represent the average of two independent biological replicates, and IC_50_ values represent the average of three replicates. (B) Representative blots after 24 h in HCT116 cells with **9** (JPS016) and **22** (JPS036) (for all blots, see Supporting Information Figure S3).

We next sought to determine DC_50_ values for PROTACs **7, 9**, and **22**, which all exhibited >50% degradation for HDAC1 and/or HDAC3 at 1 *μ*M, while **21** was also chosen for direct comparison to structurally similar **22. 7** and **9** maintained submicromolar DC_50_ values for HDAC1 and HDAC3, with **7** displaying DC_50_ values of 0.91 ± 0.02 and 0.64 ± 0.03 *μ*M for HDAC1 and HDAC,3 respectively (Figure 5); **9** exhibited near-identical DC_50_ values of 0.55 ± 0.18 and 0.53 ± 0.13 *μ*M for HDAC1 and HDAC3, respectively. However, there was a notable observation in the dose−response curves of **7** and **9** for HDAC3 (all containing an amide bond to the L-*tert*-leucine residue of VHL); these PROTACs did not exhibit a standard dose−response curve for HDAC3 (Figure 5). At concentrations greater than 1 *μ*M, HDAC3 abundance increased rather than decreased, similar to the trend observed in the initial screening (Figure 2). This looks like a hook effect for HDAC3, while at concentrations greater than 1 *μ*M, HDAC1/2 levels continue to decrease, suggesting that at higher concentrations, HDAC3 degradation is compromised over HDAC1/2 degradation for these PROTACs. Intriguingly, this hook effect on HDAC3 was lost with PROTACs **21** and **22** (all containing an ether bond to the substituted phenyl substituent of VHL), with **22** exhibiting much more selective HDAC3 degradation over HDAC1/2. **21** and **22** now exhibited greater maximal degradation for HDAC3 over HDAC1, in comparison to **7** and **9**, which exhibit greater maximal degradation for HDAC1 over HDAC3. Notably, **22** exhibited a DC_50_ value of 0.44 ± 0.03 *μ*M for HDAC3 and a Dmax value of 77% for HDAC3, with the least HDAC1 and HDAC2 degradation (Dmax values 41 and 18%, respectively) compared to the other three PROTACs. One explanation for the loss of the hook effect and enhanced selectivity for HDAC3 in **21** and **22** could be the differential orientation of the recruited VHL E3-ligase in ternary complex formation compared to **7** and **9**.^26^

We also determined the IC_50_ values for **7, 9, 21**, and **22** and CI-994 with the purified HDAC1-LSD1-CoREST complex, HDAC2-LSD1-CoREST complex, and HDAC3-SMRT complex (Figures 5 and S8). CI-994 exhibited IC_50_ values of 0.53 ± 0.09 *μ*M for HDAC1 and 0.62 ± 0.07 *μ*M for HDAC2 in the CoREST complex and 0.13 ± 0.01 for the HDAC3-SMRT complex, comparable to the previous literature.^30^ The IC_50_ values for **7** and **9** remained in the submicromolar range for all three HDAC-containing complexes. However, surprisingly, **22** had a significant reduction in IC_50_ values compared to CI-994 in all three HDAC-containing complexes. This loss of inhibition, while maintaining submicromolar HDAC3 degradation, further supports a hypothesis for the promotion of a more favorable ternary complex between HDAC3 and **22**.

**Figure 6:**
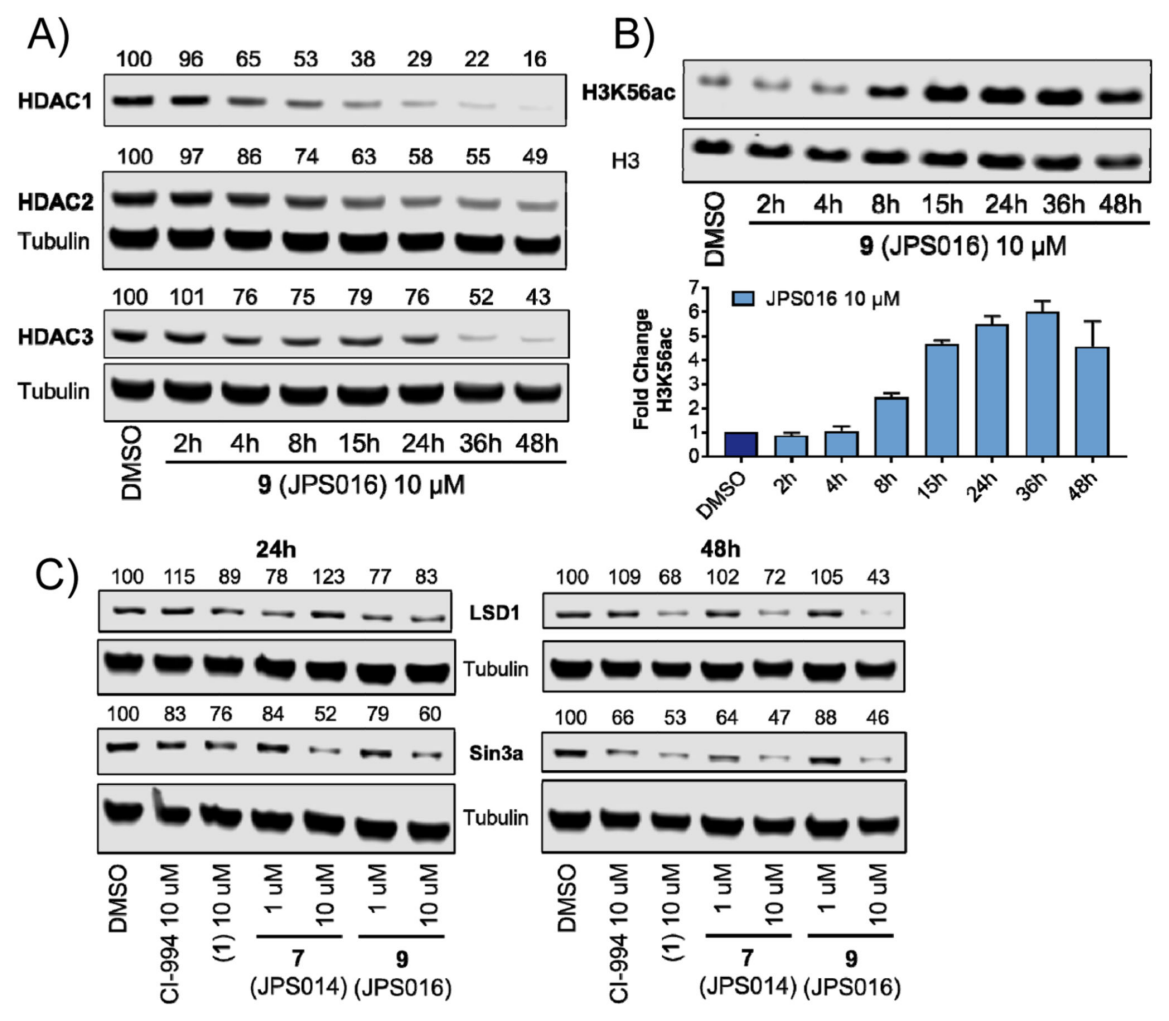
(A) HDAC1, 2, and 3 degradation levels over 48 h with **9** (JPS016) at 10 *μ*M (B) H3K56ac with **9** (JPS016) at 10 *μ*M and fold change over 48 h; error bars represent the standard deviation of two independent biological replicates. (C) Representative LSD1 and SIN3A HDAC1/2 complex partner blots after 24 and 48 h with **1** (JPS004), **7** (JPS014), and **9** (JPS016), performed in three independent biological replicates (see Supporting Information Figure S6).

We investigated the effects of **9** over 2, 4, 8, 15, 24, 36, and 48 h on HDAC1, 2, and 3 and levels H3K56ac at 1 *μ*M (see Supporting Information Figure S4) and 10 *μ*M (Figure 6A,B). Notable HDAC1/2 degradation was observed after only 4 h at 10 *μ*M, and degradation continued to increase over the 48 h time period, reaching Dmax values of 84% for HDAC1 and 51% for HDAC2 (Figure 6A). A twofold increase in H3K56ac was observed compared to the DMSO control after 8 h, reaching a maximum fold change after 36 h (Figure 6B). At 1 *μ*M, a similar trend was observed for HDAC1/2 degradation (Figure S4); however, maximal degradation was achieved after 24 h, and at 36 and 48 h, HDAC1/2 levels increased, possibly indicating inactivation of **9** at 1 *μ*M by metabolism or other pathways. At 10 *μ*M, over 24 h, little degradation was observed for HDAC3, as previously seen due to the hook effect (Figure 6A). However, at 36 and 48 h, HDAC3 degradation reached approximately 50%; we speculate that the metabolism of **9** may reduce its concentration, whereby the hook effect is negated for HDAC3. At 1 *μ*M, HDAC3 degradation was apparent from 4 h, and degradation reached maximum levels after 15 h (Figure S4). This was followed by HDAC3 levels starting to increase after 24 h, again supporting a possible time-dependent inactivation of **9** at 1 *μ*M.

To confirm that degradation was occurring via the proteasome and VHL E3-ligase, we synthesized a modified compound of **9** with the inactive VHL diasteroisomer, which as expected compromised degradation (see the Supporting Information, compound **25** and Figure S5). We also performed control experiments to investigate the effects on degradation in the presence of the proteasome inhibitor MG132 and competition experiments with the VHL ligand itself (Figure S5). The proteasome inhibitor alone modestly affected HDAC3 levels; however, despite this, degradation was still compromised in all other control experiments, providing strong evidence that **9** is recruiting the VHL E3-ligase to degrade HDAC1, 2, and 3 via the proteasome.

As class I HDACs exist in multiprotein corepressor complexes in vivo, we next wanted to determine the effects of PROTACs on components of these complexes.^1^ HDAC1/2 and 3 all contribute structurally to the integrity of their respective complexes;^31,32^ we therefore hypothesized that loss of the HDAC following degradation should also effect the stability of their binding partners. PROTACs **1, 7**, and **9** were screened for their effects on lysine-specific demethylase 1 (LSD1), a component of the CoREST complex,^33^ and SIN3A central component of the SIN3 complex. After 24 h, modest reductions in LSD1 levels were observed for **9**; however, after 48 h, when HDAC1/2 degradation is also more prominent, LSD1 was significantly reduced with all three PROTACs in comparison to the DMSO and CI-994 controls. The most potent HDAC1/2 degrader, **9**, reduced LSD1 levels to approximately 40% of controls. After 24 h with **7** and **9**, SIN3A levels were reduced to 50 and 60% abundance, respectively at 10 *μ*M; this was maintained after 48 h. However, we were surprised to observe that SIN3A levels were also significantly reduced in the presence of the inhibitor CI-994 especially after 48 h to near−same levels as PROTACs, suggesting that the effects are due to a combination of both HDAC inhibition and ubiquitin-dependent degradation pathways.

**Figure 7:**
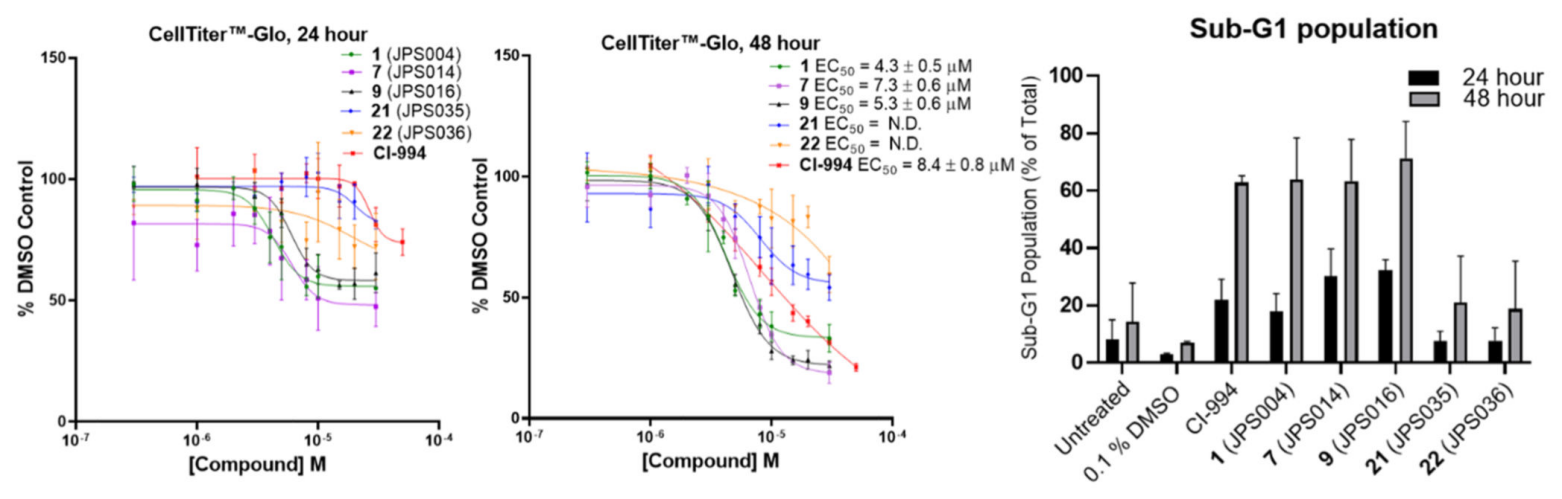
CellTiter-Glo and flow cytometry with CI-994, **1** (JPS004), **7** (JPS014), **9** (JPS016), **21** (JPS035), and **22** (JPS036). CellTiter-Glo experiments were performed with four independent biological replicates and EC_50_ values represent the average of the four replicates. Error bars in the flow cytometry experiments represent the standard deviation of two independent biological replicates.

The effects of novel PROTACs on cell viability were investigated with **1, 7, 9, 21**, and **22** using CellTiter-Glo and flow cytometry (Figure 7). After 24 h, **7** and **9**, the more potent HDAC1/2 degraders, had the highest percentage of cells in the sub-G1 phase, indicating substantial cell death when treated with PROTACs. After 48 h, **9** had the highest percentage of cells in the sub-G1 phase, followed equally by **1, 7**, and Cl-994. A similar trend was observed in the CellTiter-Glo assay after 48 h with cells showing the greatest sensitivity to treatment with **9, 1**, and **7** exhibiting EC_50_ values of 5.2 ± 0.6, 4.3 ± 0.5, and 7.3 ± 0.5 *μ*M, respectively, with the inhibitor CI-994 exhibiting an EC_50_ value of 8.4 ± 0.8 *μ*M. Interestingly, the HDAC3-selective PROTAC **22**, DC_50_ 0.44 ± 0.03 *μ*M and Dmax = 77% for HDAC3, and PROTAC **21** had little effects on cell viability (Figure 7). This implies that targeting HDAC1/2 is more important toward compromising cell viability in HCT116 cells than HDAC3. We also noted that at 10 *μ*M, **21** exhibits effective HDAC1/2 and 3 degradation at the 24 h time point (Figure 5) but does not compromise cell viability. However, in terms of DC_50,_ **21** is approximately a seven- and fourfold less-potent degrader of HDAC1 (**20** DC_50_ = 3.51 *μ*M, HDAC1) than **9** and **7**, respectively. We screened the inactive VHL diasteroisomer of **9** in flow cytometry with **9** and CI-994 (Figure S9) to further probe the differences between inhibition and degradation. The population of cells in the sub-G1 phase was equal between **9** and the inactive VHL diasteroisomer of **9** after 24 and 48 h. This suggests that inhibition with **9** is as effective in compromising cell viability and likely reflects that **9** and presumably the inactive VHL diastereoisomer of **9** are also effective submicromolar inhibitors of HDAC1, HDAC2, and HDAC3 (Figure 5).

**Figure 8:**
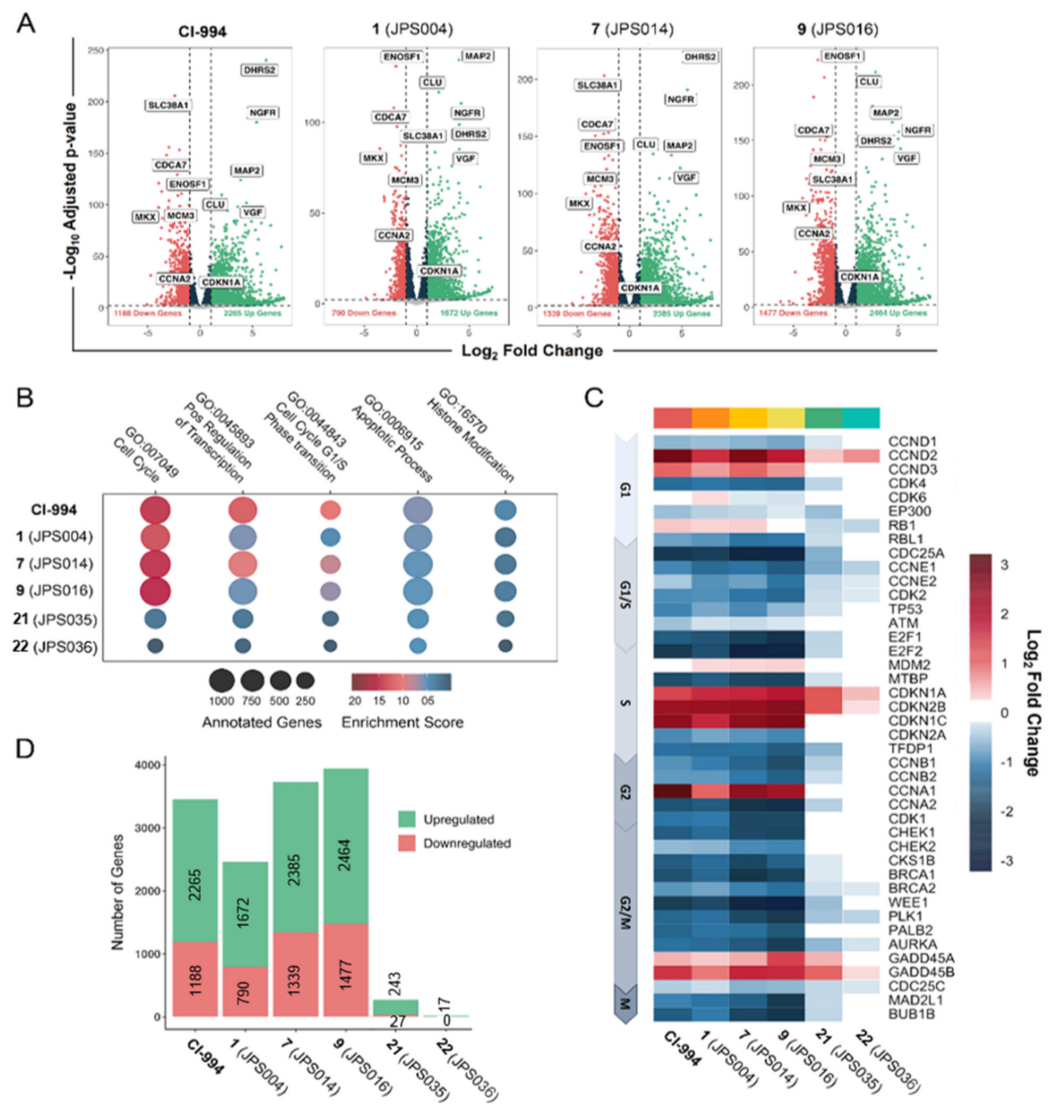
RNA-sequencing analysis in HCT116 cells reveals notable changes in gene expression following CI-994 or PROTAC treatment. (A) Volcano plots characterizing DEG patterns observed in HCT116 cells treated with the indicated PROTACs for 24 h. Performed with three independent biological replicates for each PROTAC or control. Significant DEGs were distinguished as exhibiting a *p*-adjusted value of < 0.01 and a log_2_ fold change of > 1 (fold change > 2). (B) Enrichment of key GO terms resulting from PROTAC treatment. (C) Heatmap of integral cell cycle regulator genes. (D) Number of significant DEGs following treatment with indicated PROTAC.

HDAC1/2 and 3 regulate global gene expression by manipulating histone acetylation levels across the genome. To examine the impact of PROTAC-mediated degradation on the HCT116 transcriptome, we performed RNA-seq with CI-994, **1, 7, 9, 21**, and **22**. Differential gene analysis (Figure 8A) revealed substantial transcriptional changes resulting from the majority of the PROTACs used (*p*-adjusted value of < 0.01 and a log2 fold change of 1). PROTACs **1, 7**, and **9** all displayed a striking phenotype, akin to CI-994. Differentially expressed gene (DEG) sets were subjected to gene ontology (GO) analysis. PROTAC treatment elicits a range of transcriptional changes to key cellular processes, including enrichment in cell cycle, apoptosis, and histone modification pathways (Figure 8B). The pronounced change in cell cycle-related genes is highlighted by the prominent downregulation of core regulatory factors, such as E2F1, CDK1, and cyclin E1, while there was upregulation of cell cycle inhibitors including p21 (CDKN1A) and p15 (CDKN2B) shown in Figure 8C. These changes are consistent between CI994, **1, 7**, and **9**, showing that both inhibition and degradation produce a strong antiproliferative phenotype in cancer cells. In addition, genes associated with apoptosis were also found to be significantly enriched (Figure S10, see the Supporting Information), including proapoptotic TP63 and PMAIP1 and DHSRS2, which has been previously characterized in the promotion of HDACi-mediated apoptosis through the attenuation of MDM2-dependent p53 degradation.^34,35^ There was a distinct correlation between the potency of degradation and the number of DEGs (compare Figure 5A with Figure 8D). PROTAC **9**, identified as the most potent HDAC1/2 degrader and cytotoxic compound by flow cytometry, exhibited the greatest level of differential gene expression with 2464 and 1477 up- and downregulated DEGs, respectively. Compared to CI-994, both **7** and **9** appear to show an increased number of DEGs, consistent with their ability to promote apoptosis (Figure 8D). In contrast, the less-potent HDAC1/2 and 3 degrader **21** showed approximately 10-fold less DEGs. However, perhaps even more interestingly, the HDAC3-selective PROTAC **22** (Figure 5A) showed the least effects on DEGs (Figures 8D and S10), indicating that HDAC1/2 compexes are the dominant HDAC isoforms in this cell type. Despite being relatively few, the majority of DEGs for **22** are upregulated, suggesting that HDAC3, as part of the NCoR/SMRT complex, operates as a classical corepressor complex, while HDAC1/2-containing complexes play roles in both gene repression and active gene transcription.

## Discussion

Through modifications to the linker and VHL ligand of benzamide-based class I HDAC PROTACS, we have discovered **7** (JPS014), **9** (JPS016), and **22** (JPS036) submicromolar degraders of HDAC1 and/or HDAC3. Subtle alterations in the VHL ligand and attachment to the linker can have significant effects on the degradation profile of these PROTACs. For example, **7** and **9** exhibit the hook effect for HDAC3, while **21** and **22** exhibit a standard dose response curve for HDAC3. We unexpectedly found that substitution of the acetyl group for a fluorinated cyclopropane ring in VHL led to an HDAC3-selective degrader **22**, although loss of HDAC3 alone did not cause significant cell death or changes in the transcriptome.

The more potent HDAC1/2 degraders **7** and **9** compromise LSD1 stability as part of the CoREST complex, highlighting a potential advantage to degrading class I HDACs, in addition to inhibition in situ. We also observed a strong correlation between HDAC1/2 degradation, induced cell death, and differential gene expression. PROTAC **9** appears to be improved in comparison to **1** with regards to changes in the transcriptome and apoptosis (Figures 7 and 8D). Both **7** and **9** showed a striking cell arrest phenotype when added to HCT116 cells with a significant reduction in proteins that promote G1/S transition, such E2F1, cyclin E1, and CDK2, with a concomitant increase in p21 (CDKN1A) and p15 (CDKN2B), similar to CI-994.

While HDAC3 and HDAC6 degraders have previously been reported in the literature,^23,36−39^ degraders of HDAC1 are less common. As far as we are aware, **7** and **9** are the first submicromolar degraders of HDAC1 reported. In a recent chemoproteomic study reported by Xiong et al. with PROTAC design based on pan-HDACi, HDAC3 and HDAC6 were found to be the most commonly degraded HDAC enzymes, with HDAC1/2 and HDAC9 being the least.^36^ Complementary to this study, we have demonstrated through a focused VHL-recruiting benzamide PROTAC library that effective HDAC1/2 degraders can be obtained. We anticipate that there will be great interest in more potent HDAC1/2 degraders as potential therapeutics and as reagents to unlock the diverse function of different corepressor complexes. We are optimistic that a degradation strategy can be harnessed to target individual class I HDAC complexes selectively and thus generate improved therapeutics that retain the benefits of HDACi activity but with much reduced side effects.

## Chemistry

**Scheme 1:**
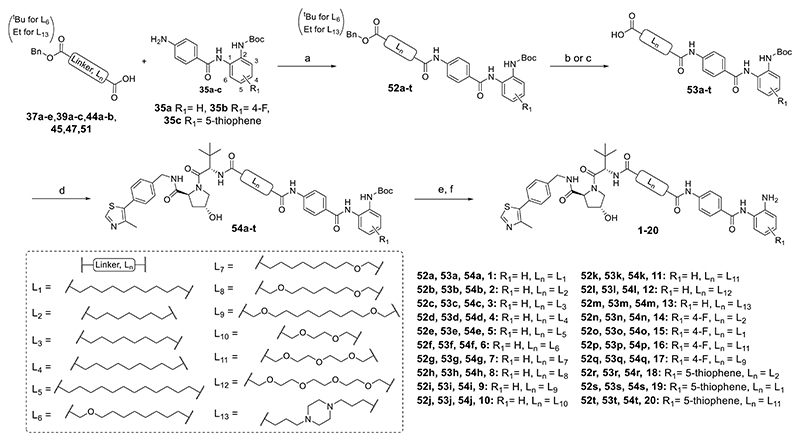
Compounds 1™20*^a^* ^*a*^Reagents and conditions: (a) HATU, *N*,*N*-diisopropylethylamine (DIPEA), dimethylformamide (DMF), r.t., overnight; (b) H_2_, Pd/C (10% weight), methanol (MeOH) or tetrahydrofuran (THF), r.t., overnight; (c) NaOH (0.4 M), MeOH or MeOH/DCM = 1:9, r.t., 4−16 h; (d) **VH_032 amine**, HATU, DIPEA, DMF, r.t., overnight; (e) TFA, DCM, r.t., 4 h; and (f) MP-carbonate resin, MeOH, r.t., 2 h.

**Scheme 2:**
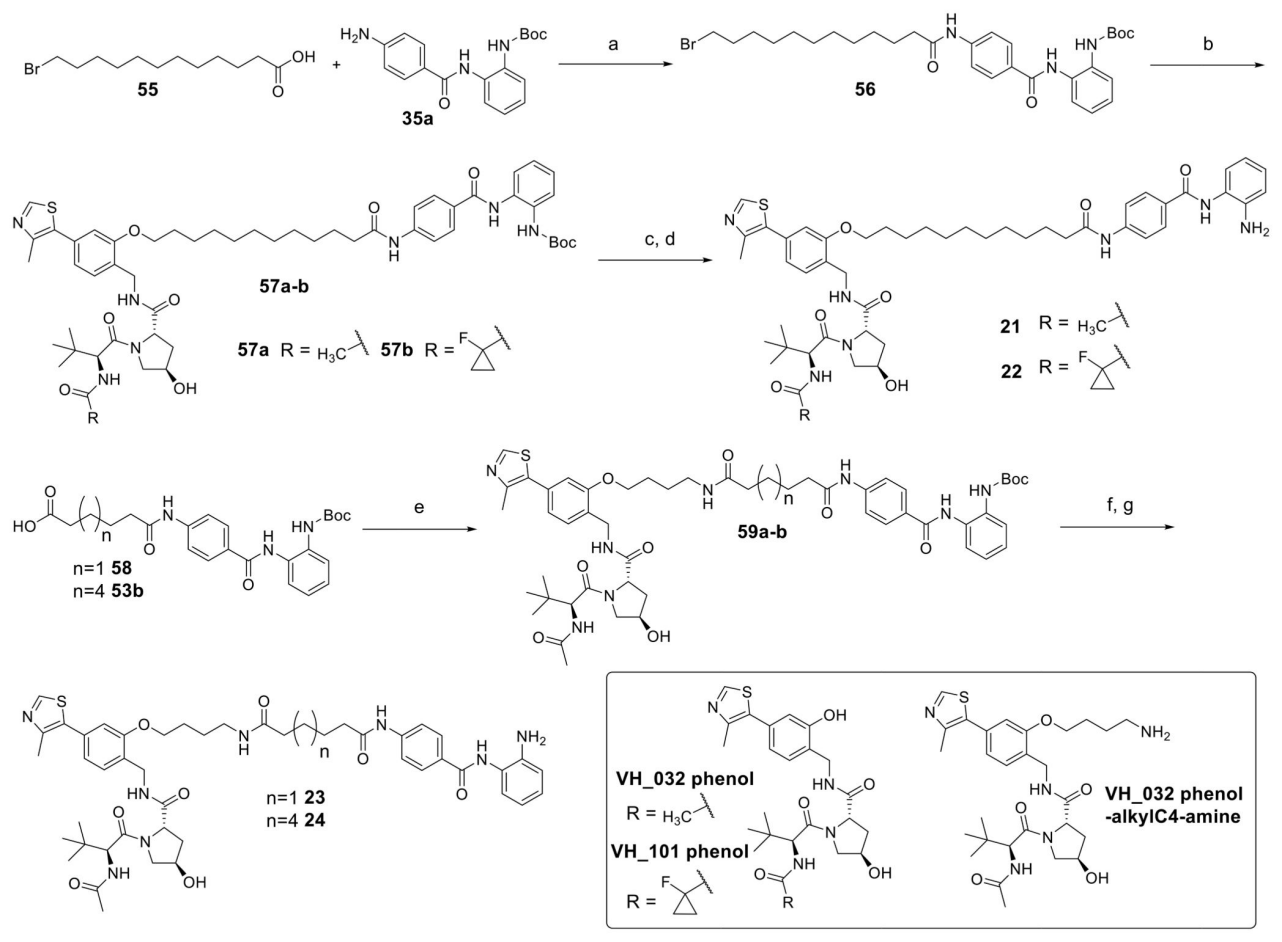
Compounds 21™24*^a^* ^*a*^Reagents and conditions: (a) HATU, DIPEA, DMF, r.t., overnight; (b) **VH_032 phenol** or **VH_101 phenol**, K_2_CO_3_, DMF, 70 °C, overnight; (c) TFA, DCM, r.t., 4 h; (d) MP-carbonate resin, MeOH, r.t., 2 h; (e) **VH_032 phenol-alkylC4-amine**, HATU, DIPEA, DMF, r.t., overnight; and (f) TFA, DCM, r.t., 4 h; (g) MP-carbonate resin, MeOH, r.t., 2 h.

Heterobifunctional molecules **1**−**20** were prepared in five steps (Scheme 1). Monoprotected linkers (**37a**−**e, 39a**−**c, 44a**−**b, 45, 47**, and **51**) were conjugated to substituted benzamides **35a**−**c** by amide coupling with hexafluorophosphate azabenzotriazole tetramethyl uronium (HATU). For the full synthesis and characterization of linkers and substituted benzamides, see the Supporting Information. The carboxyl protecting group in intermediates **52a**−**t** was removed by base saponification or hydrogenolysis to yield intermediates **53a**−**t**, which were conjugated to commercially available **VH_032 amine** via HATU-mediated amide coupling to give **54a**−**t**. The Boc protecting groups in intermediates **54a**−**t** were removed in trifluoroacetic acid (TFA)/dichloromethane (DCM), and after work-up, residual TFA was removed using a carbonate-based solid support resin, and final compounds **1**−**20** were purified by semipreparative high-performance liquid chromatography (HPLC) or column chromatography. Heterobifunctional molecules **21** and **22** were prepared in four steps (Scheme 2), the main difference to the preparation of **1**−**20** being a substitution reaction between **56** and **VH_032 phenol** in the preparation of **21** and **56** and **VH_101 phenol** in the preparation of **22**. Compounds **23** and **24** were prepared by amide coupling via HATU with **VH_032 phenol-alkylC4-amine** and **58** and **49b**, respectively, followed by Boc removal.

## Experimental Section

### General Chemical Methods

All reagents were purchased from commercially available sources and used without further purification. **VH_032 amine, VH_032 phenol, VH_101 phenol**, and **VH_032 phenol-alkylC4-amine** were purchased from Tocris Bioscience. Preparative column chromatography and flash column chromatography using a Biotage Isolera purification system were both performed using silica gel 60 (230−400 mesh). Semipreparative HPLC was performed on a Thermo Fisher Ultimate 3000 system with Chromeleon software on a Phenomenex Luna C18 column. The mobile phases were water and acetonitrile with a flow rate of 10 mL/min, 45 min gradient. NMR spectra were acquired using a Bruker 400 (^1^H, 400 MHz; ^13^C 101 MHz) instrument at ambient temperature using a deuterated solvent as a reference. High-resolution mass spectra (HRMS) were recorded on a Water Aquity XEVO Q ToF machine and measured in *m*/*z*. Analytical UPLC-MS data were collected on a Xevo G2-XS QToF mass spectrometer (Waters) coupled to an Acquity LC system (Waters) using an Acquity UPLC BEH C18 column (130 Å, 1.7 *μ*m, 2.1 × 50 mm, Waters). The mobile phases were water and acetonitrile with a flow rate of 0.6 mL/min, 10 min gradient. The purities of all final compounds were over 95% as determined by LC−MS analysis monitored at 260 nm and 310 nm. HPLC traces for **1** (JPS004), **7** (JPS014), 9 (JPS016), **21** (JPS035), and **22** (JPS036) are included in the Supporting Information. All intermediates and final compounds were fully assigned by ^1^H and ^13^C NMR using 2D NMR spectra (see the Supporting Information for full analysis). See the Supporting Information for synthetic schemes and general procedures for the preparation of carboxylic acid linker intermediates (**37a**−**e, 39a**−**c, 44a**−**b, 45, 47**, and **51**) and HDACi intermediates **(35a**−**c**).

### General Procedure for Preparing Compounds 1−20 as Described in Scheme 1, Showing the Synthesis of 7 (JPS014) as an Example

To a solution of **47** (130.0 mg, 0.403 mmol) in dry dimethylformamide (DMF) (4 mL) at 0 °C, *N*,*N*-diisopropylethylamine (DIPEA) (0.18 mL, 1.03 mmol) and HATU (183.9 mg, 0.484 mmol) were added. The reaction mixture was stirred for 15 min, after which a solution of amine **35a** (110.0 mg, 0.336 mmol) in DMF (2 mL) was added slowly, and the resultant solution was stirred at room temperature overnight. The reaction mixture was diluted in EtOAc (30 mL) and then washed with sat. NaHCO_3_ (2 × 15 mL) and sat. NaCl (2 × 15 mL). The organic layer was dried over MgSO_4_, filtered, and concentrated in vacuo to give the corresponding crude, which was chromatographically purified (0−100% EtOAc in hexane) to afford **52g** (157.4 mg, 0.247 mmol, 73% yield) as a colorless tar.

To a solution of the benzyl ester-protected HDACi-linker conjugate **52g** (120.2 mg, 0.190 mmol) in tetrahydrofuran (THF), Pd/C (10% wt) was added. The reaction flask was filled with nitrogen and evacuated three times using a Schlenk line, before a balloon of hydrogen was added, and the resultant mixture was stirred vigorously overnight. The reaction mixture was filtered through a glass microfiber filter paper, and the filtrate was concentrated in vacuo to afford **53g** (105.6 mg, 0.189 mmol, 99% yield) as a white solid.

To a solution of HDACi-linker acid **53g** (52.8 mg, 0.097 mmol) in dry DMF (1 mL) at 0 °C, DIPEA (0.042 mL, 0.239 mmol) and HATU (44.4 mg, 0.117 mmol) were added. The reaction mixture was stirred for 15 min, after which a solution of (4*R*)-3-methyl-L-valyl-4-hydroxy-*N*-[[4-(4-methyl-5-thiazolyl)phenyl]methyl]-L-prolinamide hydrochloride (**VH_032 amine**, 40.0 mg, 0.080 mmol) in DMF (1 mL) was added slowly, and the resultant solution was stirred at room temperature for 16 h. The reaction mixture was diluted in EtOAc (10 mL) and then washed with sat. NaHCO_3_ (2 × 5 mL) and sat. NaCl (2 × 5 mL). The organic layer was dried over MgSO_4_, filtered, and concentrated in vacuo to give the corresponding crude, which was chromatographically purified (0−10% MeOH in DCM) to afford **54g** (72.9 mg, 0.076 mmol, 95% yield) as a pale-yellow/white solid.

TFA (0.4 mL) was added to a stirring solution of Boc-protected PROTAC **54g** (52.8 mg, 0.097 mmol) in DCM (2 mL), and the resulting reaction mixture was stirred at room temperature for 4 h. The reaction mixture was concentrated in vacuo, dissolved in MeOH (2 mL), agitated in MP-carbonate resin (3.02 mmol/g loading capacity) for 3 h, and then filtered. The filtrate was concentrated in vacuo, and the resulting solid was dissolved in MeCN/H_2_O (1:1) and lyophilized to remove residual TFA impurities, affording **7** (50.6 mg, 0.059 mmol, 93% yield) as a pale-yellow solid. Prior to biological evaluation, the PROTAC was further purified by semipreparative HPLC (5−95% MeCN in H_2_O, 260 nm, 45 min gradient).

#### (2S,4R)-1-((S)-2-(9-(2-((4-((2-Aminophenyl)carbamoyl)phenyl)-amino)-2-oxoethoxy)nonanamido)-3,3-dimethylbutanoyl)-4-hydroxy-N-(4-(4-methylthiazol-5-yl)benzyl)pyrrolidine-2-carboxamide (7)

^1^H NMR (400 MHz, CD_3_OD): *δ*_H_ ppm 8.87 (s, 1 H), 8.64 (t, *J* = 5.9 Hz, 1 H),7.98 (d, *J* = 8.7 Hz, 2 H), 7.75−7.83 (m, 3H), 7.46 (d, *J* = 8.3 Hz, 2 H), 7.41 (d, *J* = 8.3 Hz, 2 H), 7.18 (dd, *J* = 7.7, 1.3 Hz, 1 H), 7.08 (apparent (app.) td, *J* = 7.7, 1.3 Hz, 1 H), 6.91 (dd, *J* = 7.7, 1.3 Hz, 1 H), 6.77 (app. td, *J* = 7.7, 1.3 Hz, 1 H), 4.62−4.65 (m, 1 H), 4.55−4.60 (m, 1 H), 4.47−4.54 (m, 2 H), 4.32−4.38 (m, 1 H), 4.10 (s, 2 H), 3.87−3.92 (m, 1 H), 3.77−3.82 (m, 1 H), 3.60 (t, *J* = 6.6 Hz, 2 H), 2.47 (s, 3 H), 2.19−2.32 (m, 3 H), 2.04−2.11 (m, 1 H), 1.66−1.72 (m, 2 H), 1.58−1.65 (m, 2 H), 1.33−1.44 (m, 8 H), 1.03 (s, 9 H). ^13^C NMR (101 MHz, CD_3_OD): *δ*_C_ ppm *δ* ppm 176.1, 174.6, 172.5, 171.4, 168.3, 153.0, 149.2, 143.9, 142.5, 140.4, 133.5, 131.6, 131.1, 130.5, 130.0, 129.1, 128.6, 127.8, 125.5, 120.9, 119.8, 118.9, 73.1, 71.5, 71.2, 61.0, 59.1, 58.2, 43.8, 39.1, 36.8, 36.7, 30.6, 30.5, 30.45, 30.4, 27.2 (2 C), 27.1, 16.0. HRMS (ESI) *m*/*z*: [M + H]^+^ calcd for C_46_H_60_N_7_O_7_S, 854.4275; found, 854.4268.

### Compounds Prepared As Described in Scheme 1: 1, 2, 3, 4, 5, 6, 8, 9, 10, 11, 12, 13, 14, 15, 16, 17, 18, 19 and 20

#### N1-(4-((2-Aminophenyl)carbamoyl)phenyl)-N12-((S)-1-((2S,4R)-4-hydroxy-2-((4-(4-methylthiazol-5-yl)benzyl)carbamoyl)pyrrolidin-1-yl)-3,3-dimethyl-1-oxobutan-2-yl)dodecanediamide (1)

^1^H NMR (400 MHz, CD_3_OD): *δ*_H_ ppm 8.87 (s, 1 H), 7.95 (d, *J* = 8.8 Hz, 2 H), 7.72 (d, *J* = 8.8 Hz, 2 H), 7.43−7.48 (m, 2 H), 7.38−7.42 (m, 2 H), 7.18 (dd, *J* = 7.8, 1.3 Hz, 1 H), 7.07 (app. td, *J* = 7.8, 1.3 Hz, 1 H), 6.90 (dd, *J* = 7.8, 1.3 Hz, 1 H), 6.76 (app. td, *J* = 7.8, 1.3 Hz, 1 H), 4.60−4.66 (m, 1 H), 4.55−4.60 (m, 1 H), 4.50−4.55 (m, 1 H), 4.47−4.50 (m, 1 H), 4.31−4.39 (m, 1 H), 3.86−3.93 (m, 1 H), 3.76−3.83 (m, 1 H), 2.47 (s, 3 H), 2.40 (t, *J* = 7.5 Hz, 2 H), 2.18−2.33 (m, 3 H), 2.03−2.12 (m, 1 H), 1.70 (quin, *J* = 7.5 Hz, 2 H), 1.53−1.64 (m, 2 H), 1.28−1.41 (m, 12 H), 1.03 (s, 9 H). ^13^C NMR (101 MHz, CD_3_OD): *δ*_C_ ppm 176.2, 175.1, 174.6, 172.5, 168.4, 153.0, 149.2, 144.0, 143.7, 140.4, 133.6, 131.7, 130.6, 130.5, 129.9, 129.1, 128.6, 127.8, 125.6, 120.4, 119.8, 118.9, 71.2, 61.0, 59.1, 58.2, 43.8, 39.1, 38.2, 36.8, 36.7, 30.7, 30.6, 30.55, 30.5, 30.4 (2 C), 27.2, 27.1, 26.9, 16.0. HRMS (ESI) *m*/*z*: [M + H]^+^ calcd for C_47_H_62_N_7_O_6_S, 852.4476; found, 852.4482.

#### N1-(4-((2-Aminophenyl)carbamoyl)phenyl)-N9-((S)-1-((2S,4R)-4-hydroxy-2-((4-(4-methylthiazol-5-yl)benzyl)carbamoyl)pyrrolidin-1-yl)-3,3-dimethyl-1-oxobutan-2-yl)nonanediamide (2)

^1^H NMR (400 MHz, CD_3_OD): *δ*_H_ ppm 8.87 (s, 1 H), 7.95 (d, *J* = 8.7 Hz, 2 H), 7.81 (d, *J* = 8.9 Hz, 1 H), 7.72 (d, *J* = 8.7 Hz, 2 H), 7.45 (d, *J* = 8.4 Hz, 2 H), 7.40 (d, *J* = 8.4 Hz, 2 H), 7.18 (app. dd, *J* = 7.8, 1.3 Hz, 1 H), 7.07 (app. td, *J* = 7.8, 1.3 Hz, 1 H), 6.90 (app. dd, *J* = 7.8, 1.3 Hz, 1 H), 6.76 (app. td, *J* = 7.8, 1.3 Hz, 1 H), 4.62−4.66 (m, 1 H), 4.55−4.60 (m, 1 H), 4.51−4.55 (m, 1 H), 4.48−4.50 (m, 1 H), 4.33−4.38 (m, 1 H), 3.87−3.94 (m, 1 H), 3.77−3.83 (m, 1 H), 2.47 (s, 3 H), 2.39 (t, *J* = 7.5 Hz, 2 H), 2.18−2.32 (m, 3 H), 2.03−2.11 (m, 1 H), 1.71 (quin, *J* = 7.5 Hz, 2 H), 1.62 (quin, *J* = 6.9 Hz, 2 H), 1.33−1.42 (m, 6 H), 1.03 (s, 9 H). ^13^C NMR (101 MHz, CD_3_OD): *δ*_C_ ppm 176.2, 175.1, 174.6, 172.5, 168.4, 153.0, 149.2, 143.9, 143.6, 140.4, 133.6, 131.6, 130.6, 130.5, 129.9, 129.1, 128.6, 127.8, 125.5, 120.4, 119.8, 118.9, 71.2, 61.0, 59.1, 58.2, 43.8, 39.1, 38.2, 36.75, 36.7, 30.3, 30.25, 30.2, 27.2, 27.1, 26.8, 16.0. HRMS (ESI) *m*/*z*: [M + H]^+^ calcd for C_44_H_56_N_7_O_6_S, 810.4013; found, 810.4005.

#### N1-(4-((2-Aminophenyl)carbamoyl)phenyl)-N10-((S)-1-((2S,4R)-4-hydroxy-2-((4-(4-methylthiazol5-yl)benzyl)carbamoyl)pyrrolidin-1-yl)-3,3-dimethyl-1-oxobutan-2-yl)decanediamide (3)

^1^H NMR (400 MHz, CD_3_OD): *δ*_H_ ppm 8.87 (s, 1 H), 7.95 (d, *J* = 8.7 Hz, 2 H), 7.72 (d, *J* = 8.7 Hz, 2 H), 7.44−7.48 (m, 2 H), 7.39−7.43 (m, 2 H), 7.18 (dd, *J* = 7.7, 1.4 Hz, 1 H), 7.08 (app. td, *J* = 7.7, 1.4 Hz, 1 H), 6.90 (dd, *J* = 7.7, 1.4 Hz, 1 H), 6.77 (app. td, *J* = 7.7, 1.4 Hz, 1 H), 4.64 (s, 1 H), 4.56−4.60 (m, 1 H), 4.53 (d, *J* = 15.1 Hz, 1 H), 4.47−4.50 (m, 1 H), 4.35 (d, *J* = 15.5 Hz, 1 H), 3.86−3.94 (m, 1 H), 3.76−3.84 (m, 1 H), 2.47 (s, 3 H), 2.40 (t, *J* = 7.4 Hz, 2 H), 2.19−2.32 (m, 3 H), 2.04−2.12 (m, 1 H), 1.67−1.76 (m, 2 H), 1.55−1.65 (m, 2 H), 1.33−1.39 (m, 8 H), 1.03 (s, 9 H). ^13^C NMR (101 MHz, CD_3_OD): *δ*_C_ ppm 176.2, 175.1, 174.6, 172.5, 168.3, 153.0, 149.1, 143.8, 143.6, 140.4, 133.7, 131.6, 130.6, 130.5, 129.9, 129.1, 128.6, 127.8, 125.6, 120.4, 119.9, 118.9, 71.2, 61.0, 59.1, 58.2, 43.8, 39.1, 38.2, 36.8, 36.7, 30.4, 30.35, 30.3, 30.25, 27.2, 27.1, 26.9, 16.0. HRMS (ESI) *m*/*z*: [M + H]^+^ calcd for C_45_H_58_N_7_O_6_S, 824.4169; found, 824.4160.

#### N1-(4-((2-Aminophenyl)carbamoyl)phenyl)-N11-((S)-1-((2S,4R)-4-hydroxy-2-((4-(4-methylthiazol5-yl)benzyl)carbamoyl)pyrrolidin-1-yl)-3,3-dimethyl-1-oxobutan-2-yl)undecanediamide (4)

^1^H NMR (400 MHz, CD_3_OD): *δ*_H_ ppm 8.86 (s, 1 H), 7.95 (d, *J* = 8.7 Hz, 2 H), 7.80 (d, *J* = 9.0 Hz, 1 H), 7.72 (d, *J* = 8.7 Hz, 2 H), 7.43−7.47 (m, 2 H), 7.38−7.42 (m, 2 H), 7.18 (dd, *J* = 7.8, 1.3 Hz, 1 H), 7.07 (app. td, *J* = 7.8, 1.3 Hz, 1 H), 6.90 (dd, *J* = 7.8, 1.3 Hz, 1 H), 6.77 (app. td, *J* = 7.8, 1.3 Hz, 1 H), 4.64 (d, *J* = 9.0 Hz, 1 H), 4.55−4.60 (m, 1 H), 4.52 (d, *J* = 15.5 Hz, 1 H), 4.46−4.50 (m, 1 H), 4.35 (d, *J* = 15.5 Hz, 1 H), 3.86−3.93 (m, 1 H), 3.75−3.82 (m, 1 H), 2.46 (s, 3 H), 2.39 (t, *J* = 7.5 Hz, 2 H), 2.17−2.31 (m, 3 H), 2.03−2.11 (m, 1 H), 1.70 (quin, *J* = 7.5 Hz, 2 H), 1.55−1.64 (m, 2 H), 1.30−1.39 (m, 10 H), 1.03 (s, 9 H). ^13^C NMR (101 MHz, CD_3_OD): *δ*_C_ ppm 176.2, 175.1, 174.6, 172.5, 168.4, 153.0, 149.2, 143.9, 143.6, 140.4, 133.6, 131.6, 130.6, 130.5, 129.9, 129.1, 128.6, 127.8, 125.6, 120.4, 119.8, 118.9, 71.2, 61.0, 59.1, 58.2, 43.8, 39.1, 38.2, 36.8, 36.7, 30.5, 30.45 (2 C), 30.4 (2 C), 27.2, 27.1, 26.9, 16.0. HRMS (ESI) *m*/*z*: [M + H]^+^ calcd for C_46_H_60_N_7_O_6_S, 838.4326; found, 838.4329.

#### N1-(4-((2-Aminophenyl)carbamoyl)phenyl)-N14-((S)-1-((2S,4R)-4-hydroxy-2-((4-(4-methylthiazol-5-yl)benzyl)carbamoyl)-pyrrolidin-1-yl)-3,3-dimethyl-1-oxobutan-2-yl)tetradecanediamide (5)

^1^H NMR (400 MHz,CD_3_OD): *δ*_H_ ppm 8.86 (s, 1 H), 7.95 (d, *J* = 8.7 Hz, 2 H), 7.72 (d, *J* = 8.7 Hz, 2 H), 7.43−7.47 (m, 2 H), 7.38−7.43 (m, 2 H), 7.18 (dd, *J* = 7.8, 1.3 Hz, 1 H), 7.06 (app. td, *J* = 7.8, 1.3 Hz, 1 H), 6.90 (dd, *J* = 7.8, 1.3 Hz, 1 H), 6.76 (app. td, *J* = 7.8, 1.3 Hz, 1 H), 4.63 (s, 1 H), 4.55−4.60 (m, 1 H), 4.52 (d, *J* = 15.5 Hz, 1 H), 4.46−4.50 (m, 1 H), 4.34 (d, *J* = 15.5 Hz, 1 H), 3.86−3.93 (m, 1 H), 3.75−3.82 (m, 1 H), 2.46 (s, 3 H), 2.39 (t, *J* = 7.4 Hz, 2 H), 2.17−2.32 (m, 3 H), 2.03−2.11 (m, 1 H), 1.70 (quin, *J* = 7.4 Hz, 2 H), 1.52−1.65 (m, 2 H), 1.27−1.39 (m, 16 H), 1.03 (s, 9 H). ^13^C NMR (101 MHz, CD_3_OD): *δ*_C_ ppm 176.2, 175.1, 174.6, 172.5, 168.4, 153.0, 149.2, 143.9, 143.6, 140.4, 133.6, 131.6, 130.6, 130.5, 129.9, 129.1, 128.6, 127.8, 125.6, 120.4, 119.8, 118.9, 71.2, 61.0, 59.1, 58.2, 43.8, 39.0, 38.2, 36.8, 36.7, 30.8 (2 C), 30.75, 30.7, 30.6 (2 C), 30.5, 30.4, 27.2, 27.15, 26.9, 16.0. HRMS (ESI) *m*/*z*: [M + H]^+^ calcd for C_49_H_66_N_7_O_6_S, 880.4795; found, 880.4762.

#### (2S,4R)-1-((S)-2-(2-((9-((4-((2-Aminophenyl)carbamoyl)phenyl)-amino)-9-oxononyl)oxy)acetamido)-3,3-dimethylbutanoyl)-4-hydroxy-N-(4-(4-methylthiazol-5-yl)benzyl)pyrrolidine-2-carboxamide (6)

^1^H NMR (400 MHz, CD_3_OD): *δ*_H_ ppm 8.86 (s, 1 H), 7.95 (d, *J* = 8.7 Hz, 2 H), 7.72 (d, *J* = 8.7 Hz, 2 H), 7.44−7.47 (m, 2 H), 7.39−7.43 (m, 2 H), 7.18 (dd, *J* = 7.7, 1.1 Hz, 1 H), 7.07 (app. td, *J* = 7.7, 1.1 Hz, 1 H), 6.90 (dd, *J* = 7.7, 1.1 Hz, 1 H), 6.77 (app. td, *J* = 7.7, 1.1 Hz, 1 H), 4.69 (s, 1 H), 4.56−4.63 (m, 1 H), 4.47−4.55 (m, 2 H), 4.36 (d, *J* = 15.5 Hz, 1 H), 3.96−4.00 (m, 1 H), 3.91−3.95 (m, 1 H), 3.85−3.90 (m, 1 H), 3.76−3.82 (m, 1 H), 3.55 (t, *J* = 6.3 Hz, 2 H), 2.47 (s, 3 H), 2.38 (t, *J* = 7.5 Hz, 2 H), 2.20−2.26 (m, 1 H), 2.04−2.12 (m, 1 H), 1.62−1.72 (m, 4 H), 1.36−1.46 (m, 8 H), 1.03 (s, 9 H). ^13^C NMR (101 MHz, CD_3_OD): *δ*_C_ ppm 175.0, 174.4, 172.1, 171.8, 168.3, 153.0, 149.1, 143.9, 143.6, 140.3, 133.6, 131.6, 130.6, 130.5, 129.9, 129.1, 128.6, 127.8, 125.5, 120.4, 119.8, 118.8, 73.1, 71.2, 70.8, 61.0, 58.3, 58.1, 43.9, 39.1, 38.2, 37.4, 30.7, 30.5 (2 C), 30.3, 27.3, 27.1, 26.9, 16.0. HRMS (ESI) *m*/*z*: [M + H]^+^ calcd for C_46_H_60_N_7_O_7_S, 854.4275; found, 854.4277.

#### (2S,4R)-1-((S)-2-(2-((6-(2-((4-((2-Aminophenyl)carbamoyl)-phenyl)amino)-2-oxoethoxy)hexyl) oxy)acetamido)-3,3-dimethylbutanoyl)-4-hydroxy-N-(4-(4-methylthiazol-5-yl)benzyl)pyrrolidine -2-carboxamide (8)

^1^H NMR (400 MHz, CD_3_OD): *δ*_H_ ppm 8.86 (s, 1 H), 7.97 (d, *J* = 8.6 Hz, 2 H), 7.76 (d, *J* = 8.6 Hz, 2 H), 7.43−7.47 (m, 2 H), 7.38−7.43 (m, 2 H), 7.18 (dd, *J* = 7.7, 1.1 Hz, 1 H), 7.07 (td, *J* = 7.7, 1.1 Hz, 1 H), 6.90 (dd, *J* = 7.7, 1.1 Hz, 1 H), 6.77 (td, *J* = 7.7, 1.1 Hz, 1 H), 4.69 (s, 1 H), 4.56−4.61 (m, 1 H), 4.53 (d, *J* = 15.5 Hz, 1 H), 4.48−4.51 (m, 1 H), 4.35 (d, *J* = 15.5 Hz, 1 H), 4.06−4.12 (m, 2 H), 3.99−4.01 (m, 1 H), 3.90−3.96 (m, 1 H), 3.84−3.90 (m, 1 H), 3.77−3.83 (m, 1 H), 3.55−3.62 (m, 4 H), 2.47 (s, 3 H), 2.19−2.27 (m, 1 H), 2.05−2.12 (m, 1 H), 1.65−1.75 (m, 4 H), 1.45−1.54 (m, 4 H), 1.03 (s, 9 H). ^13^C NMR (101 MHz, CD_3_OD): *δ*_C_ ppm 174.5, 172.1, 171.8, 171.4, 168.2, 153.0, 149.2, 144.0, 142.5, 140.3, 133.5, 131.6, 131.1, 130.5, 130.0, 129.1, 128.6, 127.8, 125.5, 120.9, 119.8, 118.8, 73.05, 73.0, 71.5, 71.2, 70.9, 61.0, 58.3, 58.1, 43.9, 39.1, 37.4, 30.7, 30.5, 27.2, 27.1 (2 C), 16.0. HRMS (ESI) *m*/*z*: [M + H]^+^ calcd for C_45_H_58_N_7_O_8_S, 856.4068; found, 856.4064.

#### (2S,4R)-1-((S)-2-(2-((9-(2-((4-((2-Aminophenyl)carbamoyl)-phenyl)amino)-2-oxoethoxy)nonyl)oxy)acetamido)-3,3-dimethylbutanoyl)-4-hydroxy-N-(4-(4-methylthiazol-5-yl)benzyl)-pyrrolidine-2-carboxamide (9)

^1^H NMR (400 MHz, CD_3_OD): *δ*_H_ ppm 8.87 (s, 1 H), 7.98 (d, *J* = 8.6 Hz, 2 H), 7.77 (d, *J* = 8.6 Hz, 2 H), 7.46 (d, *J* = 8.3 Hz, 2 H), 7.41 (d, *J* = 8.3 Hz, 2 H), 7.18 (dd, *J* = 7.7, 1.1 Hz, 1 H), 7.07 (app. td, *J* = 7.7, 1.1 Hz, 1 H), 6.90 (dd, *J* = 7.7, 1.1 Hz, 1 H), 6.77 (app. td, *J* = 7.7, 1.1 Hz, 1 H), 4.69 (s, 1 H), 4.56−4.61 (m, 1 H), 4.54 (d, *J* = 15.5 Hz, 1 H), 4.48−4.51 (m, 1 H), 4.35 (d, *J* = 15.5 Hz, 1 H), 4.09 (s, 2 H), 3.96−4.01 (m, 1 H), 3.91−3.96 (m, 1 H), 3.84−3.90 (m, 1 H), 3.76−3.82 (m, 1 H), 3.52−3.60 (m, 4 H), 2.47 (s, 3 H), 2.19−2.26 (m, 1 H), 2.04−2.11 (m, 1 H), 1.61−1.70 (m, 4 H), 1.33−1.44 (m, 10 H), 1.03 (s, 9 H). ^13^C NMR (101 MHz, CD_3_OD): *δ*_C_ ppm 174.4, 172.2, 171.8, 171.4, 168.2, 153.0, 149.2, 144.0, 142.5, 140.4, 133.6, 131.6, 131.1, 130.5, 130.0, 129.1, 128.7, 127.8, 125.5, 120.9, 119.8, 118.9, 73.2, 73.1, 71.5, 71.2, 70.9, 61.0, 58.3, 58.1, 43.9, 39.1, 37.4, 30.8, 30.75, 30.65, 30.6, 30.55, 27.4, 27.3, 27.1, 16.0. HRMS (ESI) *m*/*z*: [M + H]^+^ calcd for C_48_H_64_N_7_O_8_S, 898.4537; found, 898.4531.

#### (2S,4R)-1-((S)-2-(2-(2-(2-((4-((2-Aminophenyl)carbamoyl)-phenyl)amino)-2-oxoethoxy)ethoxy) acetamido)-3,3-dimethylbutanoyl)-4-hydroxy-N-(4-(4-methylthiazol-5-yl)benzyl)pyrrolidine-2-carboxamide (10)

^1^H NMR (400 MHz, CD_3_OD): *δ*_H_ ppm 8.85 (s, 1 H), 8.58 (t, *J* = 6.0 Hz, 1 H), 7.95 (d, *J* = 8.7 Hz, 2H), 7.75−7.85 (m, 3 H), 7.40−7.45 (m, 2 H), 7.36−7.40 (m, 2 H), 7.18 (dd, *J* = 7.7, 1.3 Hz, 1 H), 7.08 (app. td, *J* = 7.7, 1.3 Hz, 1 H), 6.91 (dd, *J* = 7.7, 1.3 Hz, 1 H), 6.77 (app. td, *J* = 7.7, 1.3 Hz, 1 H), 4.74 (d, *J* = 9.4 Hz, 1 H), 4.55−4.61 (m, 1 H), 4.49−4.52 (m, 1 H), 4.43−4.49 (m, 1 H), 4.30−4.36 (m, 1 H), 4.22 (s, 2 H), 4.14−4.19 (m, 1 H), 4.05−4.11 (m, 1 H), 3.86−3.91 (m, 1 H), 3.2−3.86 (m, 4 H), 3.77−3.81 (m, 1 H), 2.45 (s, 3 H), 2.19−2.26 (m, 1 H), 2.05−2.12 (m, 1 H), 1.04 (s, 9 H). ^13^C NMR (101 MHz, CD_3_OD): *δ*_C_ ppm 174.4, 172.1, 171.9, 171.2, 168.2, 152.9, 149.1, 144.0, 142.5, 140.3, 133.5, 131.6, 131.1, 130.5, 129.9, 129.0, 128.7, 127.8, 125.5, 121.0, 119.8, 118.8, 72.3, 72.1, 71.9, 71.2, 71.2, 61.0, 58.4, 58.3, 43.8, 39.1, 37.2, 27.1, 16.0. HRMS (ESI) *m*/*z*: [M + H]^+^ calcd for C_41_H_50_N_7_O_8_S, 800.3442; found, 800.3444.

#### (2S,4R)-1-((S)-14-((4-((2-Aminophenyl)carbamoyl)phenyl)-amino)-2-(tert-butyl)-4,14-dioxo-6,9,12-trioxa-3-azatetradecanoyl)-4-hydroxy-N-(4-(4-methylthiazol-5-yl)benzyl)pyrrolidine-2-carboxamide (11)

^1^H NMR (400 MHz, CD_3_OD): *δ*_H_ ppm 8.86 (s, 1 H), 8.62 (t, *J* = 6.1 Hz, 1 H), 7.97 (d, *J* = 8.7 Hz, 2 H), 7.75 (d, *J* = 8.7 Hz, 2 H), 7.64 (d, *J* = 9.4 Hz, 1 H), 7.43−7.46 (m, 2 H), 7.38−7.42 (m, 2 H), 7.19 (dd, *J* = 7.8, 1.3 Hz, 1 H), 7.09 (app. td, *J* = 7.8, 1.3 Hz, 1 H), 6.92 (dd, *J* = 7.8, 1.3 Hz, 1 H), 6.79 (app. td, *J* = 7.8, 1.3 Hz, 1 H), 4.69 (d, *J* = 9.6 Hz, 1 H), 4.55−4.59 (m, 1 H), 4.51−4.55 (m, 1 H), 4.47−4.51 (m, 1 H), 4.29−4.36 (m, 1 H), 4.14−4.18 (m, 1 H), 4.08−4.14 (m, 1 H), 4.01−4.05 (m, 1 H), 3.89−3.95 (m, 1 H), 3.84−3.87 (m, 1 H), 3.72−3.83 (m, 9 H), 2.47 (s, 3 H), 2.16−2.25 (m, 1 H), 2.04−2.12 (m, 1 H), 1.02 (s, 9 H). ^13^C NMR (101 MHz, CD_3_OD): *δ*_C_ ppm 174.5, 172.0, 171.7, 171.6, 168.3, 153.0, 149.2, 144.0, 142.4, 140.4, 133.5, 131.7, 131.3, 130.5, 130.0, 129.2, 128.7, 127.9, 125.5, 121.2, 119.8, 118.8, 72.3, 72.2, 71.7, 71.6, 71.5, 71.2, 71.1, 61.0, 58.3, 58.2, 43.9, 39.1, 37.4, 27.1, 16.0. HRMS (ESI) *m*/*z*: [M + H]^+^ calcd for C_43_H_54_N_7_O_9_S, 844.3704; found, 844.3702.

#### N1-(4-((2-Aminophenyl)carbamoyl)phenyl)-N14-((S)-1-((2S,4S)-4-hydroxy-2-((4-(4-methylthiazol-5-yl)benzyl)carbamoyl)-pyrrolidin-1-yl)-3,3-dimethyl-1-oxobutan-2-yl)-3,6,9,12-tetraoxatetradecanediamide (12)

^1^H NMR (400 MHz, CD_3_OD): *δ*_H_ ppm 8.86 (s, 1 H), 7.98 (d, *J* = 8.7 Hz, 2 H), 7.79 (d, *J* = 8.7 Hz, 2 H), 7.43−7.46 (m, 2 H), 7.39−7.42 (m, 2 H), 7.18 (dd, *J* = 7.7, 1.4 Hz, 1 H), 7.08 (app. td, *J* = 7.7, 1.4 Hz, 1 H), 6.90 (dd, *J* = 7.7, 1.4 Hz, 1 H), 6.77 (app. td, *J* = 7.7, 1.4 Hz, 1 H), 4.68 (s, 1 H), 4.56−4.58 (m, 1 H), 4.50−4.54 (m, 1 H), 4.47−4.50 (m, 1 H), 4.33 (d, *J* = 15.7 Hz, 1 H), 4.14 (s, 2 H), 4.01−4.05 (m, 1 H), 3.94−3.99 (m, 1 H), 3.84−3.88 (m, 1 H), 3.76−3.80 (m, 1 H), 3.66−3.75 (m, 12 H), 2.47 (s, 3 H), 2.18−2.23 (m, 1 H), 2.04−2.10 (m, 1 H), 1.03 (s, 9 H). ^13^C NMR (101 MHz, CD_3_OD): *δ*_C_ ppm 174.5, 172.1, 171.8, 171.5, 168.3, 153.0, 149.2, 144.0, 142.5, 140.4, 133.5, 131.6, 131.2, 130.5, 130.0, 129.1, 128.7, 127.9, 125.5, 121.0, 119.8, 118.8, 72.4, 72.3, 71.8, 71.7, 71.65, 71.6, 71.4, 71.2, 71.2, 60.9, 58.25, 58.2, 43.8, 39.1, 37.3, 27.1, 16.0. HRMS (ESI) *m*/*z*: [M + H]^+^ calcd for C_45_H_58_N_7_O_10_S, 888.3966; found, 888.3962.

#### (2S,4R)-1-((S)-2-(4-(4-(4-((4-((2-Aminophenyl)carbamoyl)-phenyl)amino)-4-oxobutyl)piperazin-1-yl)butanamido)-3,3-dimethylbutanoyl)-4-hydroxy-N-(4-(4-methylthiazol-5-yl)benzyl)-pyrrolidine-2-carboxamide (13)

^1^H NMR (400 MHz, CD_3_OD): *δ*_H_ 8.87 (s, 1 H), 7.96 (d, *J* = 8.7 Hz, 2 H), 7.73 (d, *J* = 8.7 Hz, 2 H), 7.45−7.50 (m, 2 H), 7.38−7.44 (m, 2 H), 7.18 (dd, *J* = 7.8, 1.3 Hz, 1 H), 7.07 (app. td, *J* = 7.8, 1.3 Hz, 1 H), 6.91 (dd, *J* = 7.8, 1.3 Hz, 1 H), 6.77 (app. td, *J* = 7.8, 1.3 Hz, 1 H), 4.62 (s, 1 H), 4.52−4.58 (m, 2 H), 4.46−4.51 (m, 1 H), 4.35 (d, *J* = 15.6 Hz, 1 H), 3.86−3.92 (m, 1 H), 3.76−3.83 (m, 1 H), 2.39−2.63 (m, 15 H), 2.32−2.37 (m, 2 H), 2.29 (t, *J* = 7.5 Hz, 2 H), 2.18−2.24 (m, 1 H), 2.04−2.11 (m, 1 H), 1.87−1.94 (m, 2 H), 1.74−1.83 (m, 2 H), 1.03 (s, 9 H). ^13^C NMR (101 MHz, CD_3_OD): *δ*_C_ ppm 175.6, 174.6, 174.5, 172.4, 168.4, 153.0, 149.2, 143.9, 143.7, 140.4, 133.6, 131.6, 130.6, 130.5, 129.9, 129.1, 128.6, 127.8, 125.6, 120.4, 119.8, 118.9, 71.2, 61.0, 59.2, 59.0, 58.7, 58.2, 53.9, 53.85, 43.8, 39.1, 36.7, 36.2, 34.5, 27.2, 23.8, 23.6, 16.0. HRMS (ESI) *m*/*z*: [M + H]^+^ calcd for C_47_H_62_N_9_O_6_S, 880.4544; found, 880.4548.

#### N1-(4-((2-Amino-4-fluorophenyl)carbamoyl)phenyl)-N9-((S)-1-((2S,4R)-4-hydroxy-2-((4-(4-methylthiazol-5-yl)benzyl)carbamoyl)-pyrrolidin-1-yl)-3,3-dimethyl-1-oxobutan-2-yl)nonanediamide (14)

^1^H NMR (400 MHz, CD_3_OD): *δ*_H_ ppm *δ* ppm 8.86 (s, 1 H), 7.94 (d, *J* = 8.7 Hz, 2 H), 7.71 (d, *J* = 8.7 Hz, 2 H), 7.42−7.47 (m, 2 H), 7.37−7.42 (m, 2 H), 7.11 (dd, *J*_*HH*_ = 8.6, *J*_*HF*_ = 6.0 Hz, 1 H), 6.58 (dd, *J*_*HF*_ = 10.7, *J*_*HH*_ = 2.8 Hz, 1 H), 6.41 (app. td, *J*_*HF*_ = 8.6, *J*_*HH*_ = 8.6, 2.8 Hz, 1 H), 4.63 (s, 1 H), 4.55−4.60 (m, 1 H), 4.52 (d, *J* = 15.5 Hz, 1 H), 4.46−4.50 (m, 1 H), 4.35 (d, *J* = 15.5 Hz, 1 H), 3.87−3.94 (m, 1 H), 3.75−3.82 (m, 1 H), 2.46 (s, 3 H), 2.39 (t, *J* = 7.5 Hz, 2 H), 2.18−2.32 (m, 3 H), 2.03−2.12 (m, 1 H), 1.70 (quin, *J* = 7.5 Hz, 2 H), 1.61 (quin, *J* = 7.2 Hz, 2 H), 1.32−1.40 (m, 6 H), 1.03 (s, 9 H). 13C NMR (101 MHz, CD_3_OD): *δ*_C_ ppm 176.2, 175.1, 174.6, 172.5, 168.7, 163.8 (d, *J*_*CF*_ = 241.3 Hz), 153.0, 149.1, 146.6 (d, *J*_*CF*_ = 11.6 Hz), 143.6, 140.4, 133.6, 131.6, 130.5, 130.4, 129.9, 129.7 (d, *J*_*CF*_ = 10.5 Hz), 129.1, 120.8 (d, *J*_*CF*_ = 1.7 Hz), 120.4, 105.2 (d, *J*_*CF*_ = 23.1 Hz), 104.1 (d, *J*_*CF*_ = 25.7 Hz), 71.2, 61.0, 59.1, 58.2, 43.8, 39.1, 38.2, 36.8, 36.7, 30.3, 30.2, 30.15, 27.2, 27.1, 26.8, 16.0. ^19^F NMR (376 MHz, CD_3_OD): *δ*_F_ ppm −117.5. HRMS (ESI) *m*/*z*: [M + H]^+^ calcd for C_44_H_55_FN_7_O_6_S, 828.3919; found, 828.3927.

#### N1-(4-((2-Amino-4-fluorophenyl)carbamoyl)phenyl)-N12-((S)-1-((2S,4R)-4-hydroxy-2-((4-(4-methylthiazol-5-yl)benzyl)carbamoyl)-pyrrolidin-1-yl)-3,3-dimethyl-1-oxobutan-2-yl)dodecanediamide (15)

^1^H NMR (400 MHz, CD_3_OD): *δ*_H_ ppm 8.86 (s, 1 H), 7.94 (d, *J* = 8.7 Hz, 2 H), 7.72 (d, *J* = 8.7 Hz, 2 H), 7.43−7.47 (m, 2 H), 7.37−7.42 (m, 2 H), 7.11 (dd, *J*_*HH*_ = 8.6, *J*_*HF*_ = 6.0 Hz, 1 H), 6.58 (dd, *J*_*HF*_ = 10.7, *J*_*HH*_ = 2.8 Hz, 1 H), 6.41 (app. td, *J*_*HF*_ = 8.6, *J*_*HH*_ = 8.6, 2.8 Hz, 1 H), 4.63 (s, 1 H), 4.55−4.60 (m, 1 H), 4.52 (d, *J* = 15.5 Hz, 1 H), 4.47−4.50 (m, 1 H), 4.35 (d, *J* = 15.5 Hz, 1 H), 3.86−3.93 (m, 1 H), 3.76−3.82 (m, 1 H), 2.46 (s, 3 H), 2.39 (t, *J* = 7.5 Hz, 2 H), 2.17−2.31 (m, 3 H), 2.03−2.12 (m, 1 H), 1.69 (quin, *J* = 7.5 Hz, 2 H), 1.53−1.63 (m, 2 H), 1.29−1.37 (m, 12 H), 1.03 (s, 9 H). ^13^C NMR (101 MHz, CD_3_OD): *δ*_C_ ppm 176.2, 175.1, 174.6, 172.5, 168.7, 163.8 (d, *J*_*CF*_ = 241.3 Hz), 153.0, 149.1, 146.6 (d, *J*_*CF*_ = 11.4 Hz), 143.7, 140.4, 133.5, 131.6, 130.5, 130.4, 129.9, 129.7 (d, *J*_*CF*_ = 10.3 Hz), 129.1, 120.8 (d, *J*_*CF*_ = 2.3 Hz), 120.4, 105.2 (d, *J*_*CF*_ = 23.1 Hz), 104.1 (d, *J*_*CF*_ = 25.6 Hz), 71.2, 61.0, 59.1, 58.2, 43.8, 39.1, 38.2, 36.8, 36.7, 30.65, 30.6, 30.5, 30.45, 30.4 (2 C), 27.2, 27.1, 26.9, 16.0. ^19^F NMR (376 MHz, CD_3_OD): *δ*_F_ ppm −117.5. HRMS (ESI) *m*/*z*: [M + H]^+^ calcd for C_47_H_61_FN_7_O_6_S, 870.4388; found, 870.4376.

#### (2S,4R)-1-((S)-14-((4-((2-Amino-4-fluorophenyl)carbamoyl)-phenyl)amino)-2-(tert-butyl)-4,14-dioxo-6,9,12-trioxa-3-azatetradecanoyl)-4-hydroxy-N-(4-(4-methylthiazol-5-yl)benzyl)-pyrrolidine-2-carboxamide (16)

^1^H NMR (400 MHz, CD_3_OD): *δ*_H_ ppm 8.85 (s, 1 H), 7.95 (d, *J* = 8.7 Hz, 2 H), 7.73 (d, *J* = 8.7 Hz, 2 H), 7.41−7.45 (m, 2 H), 7.37−7.41 (m, 2 H), 7.11 (dd, *J*_*HH*_ = 8.6, *J*_*HF*_ = 6.1 Hz, 1 H), 6.58 (dd, *J*_*HF*_ = 10.7, *J*_*HH*_ = 2.8 Hz, 1 H), 6.41 (app. td, *J*_*HF*_ = 8.6, *J*_*HH*_ = 8.6, 2.8 Hz, 1 H), 4.68 (s, 1 H), 4.56−4.60 (m, 1 H), 4.53 (d, *J* = 15.5 Hz, 1 H), 4.45−4.50 (m, 1 H), 4.32 (d, *J* = 15.5 Hz, 1 H), 4.12−4.19 (m, 1 H), 4.05−4.12 (m, 1 H), 4.02 (d, *J* = 15.7 Hz, 1 H), 3.91 (d, *J* = 15.6 Hz, 1 H), 3.82−3.87 (m, 1 H), 3.70−3.81 (m, 9 H), 2.46 (s, 3 H), 2.16−2.25 (m, 1 H), 2.03−2.12 (m, 1 H), 1.02 (s, 9 H). ^13^C NMR (101 MHz, CD_3_OD): *δ*_C_ ppm 174.5, 172.0, 171.7, 171.6, 168.6, 163.8 (d, *J*_*CF*_ = 241.3 Hz), 153.0, 149.2, 146.7 (d, *J*_*CF*_ = 11.6 Hz), 142.4, 140.4, 133.5, 131.7, 131.1, 130.5, 129.9, 129.8 (d, *J*_*CF*_ = 10.5 Hz), 129.1, 121.2, 120.7 (d, *J*_*CF*_ = 2.1 Hz), 105.1 (d, *J*_*CF*_ = 23.1 Hz), 104.0 (d, *J*_*CF*_ = 25.6 Hz), 72.3, 72.25, 71.7, 71.6, 71.55, 71.2, 71.1, 60.9, 58.3, 58.2, 43.9, 39.1, 37.3, 27.1, 16.0. ^19^F NMR (376 MHz, CD_3_OD): *δ* ppm −117.4. HRMS (ESI) *m*/*z*: [M + H]^+^ calcd for C_43_H_53_FN_7_O_9_S, 862.3610; found, 862.3609.

#### (2S,4R)-1-((S)-2-(2-((9-(2-((4-((2-Amino-4-fluorophenyl)-carbamoyl)phenyl)amino)-2-oxoethoxy)nonyl)oxy)acetamido)-3,3-dimethylbutanoyl)-4-hydroxy-N-(4-(4-methylthiazol-5-yl)-benzyl)pyrrolidine-2-carboxamide (17)

^1^H NMR (400 MHz, CD_3_OD): *δ*_H_ ppm 8.86 (s, 1 H), 7.96 (d, *J* = 8.7 Hz, 2 H), 7.75 (d, *J* = 8.7 Hz, 2 H), 7.42−7.47 (m, 2 H), 7.37−7.42 (m, 2 H), 7.11 (dd, *J*_*HH*_ = 8.6, *J*_*HF*_ = 6.1 Hz, 1 H), 6.58 (dd, *J*_*HF*_ = 10.7, *J*_*HH*_ = 2.8 Hz, 1 H), 6.41 (td, *J*_*HF*_ = 8.6, *J*_*HH*_ = 8.6, 2.8 Hz, 1 H), 4.68 (s, 1 H), 4.59 (dd, *J* = 9.0, 7.8 Hz, 1 H), 4.52 (d, *J* = 15.6 Hz, 1 H), 4.47−4.50 (m, 1 H), 4.34 (d, *J* = 15.6 Hz, 1 H), 4.07 (s, 2 H), 3.95 (d, *J* = 15.4 Hz, 1 H), 3.94 (d, *J* = 15.4 Hz, 1 H), 3.84−3.89 (m, 1 H), 3.74−3.82 (m, 1 H), 3.50−3.59 (m, 4 H), 2.46 (s, 3 H), 2.18−2.27 (m, 1 H), 2.02−2.12 (m, 1 H), 1.58−1.68 (m, 4 H), 1.32−1.43 (m, 10 H), 1.03 (s, 9 H). ^13^C NMR (101 MHz, CD_3_OD): *δ*_C_ ppm 174.4, 172.1, 171.8, 171.3, 168.6, 163.8 (d, *J*_*CF*_ = 241.7 Hz), 153.0, 149.1, 146.6 (d, *J*_*CF*_ = 11.6 Hz), 142.6, 140.3, 133.6, 131.6, 131.0, 130.5, 129.9, 129.7 (d, *J*_*CF*_ = 10.3 Hz), 129.1, 120.9, 120.8 (d, *J*_*CF*_ = 2.3 Hz), 105.1 (d, *J*_*CF*_ = 23.1 Hz), 104.0 (d, *J*_*CF*_ = 25.7 Hz), 73.2, 73.1, 71.5, 71.2, 70.8, 61.0, 58.3, 58.1, 43.9, 39.1, 37.3, 30.8, 30.75, 30.65, 30.6, 30.55, 27.4, 27.2, 27.1, 16.0. ^19^F NMR (376 MHz, CD_3_OD): *δ*_F_ ppm −117.4. HRMS (ESI) *m*/*z*: [M + H]^+^ calcd for C_48_H_63_FN_7_O_8_S, 916.4443; found, 916.4426.

#### N1-(4-((2-Amino-5-(thiophen-2-yl)phenyl)carbamoyl)phenyl)-N9-((S)-1-((2S,4R)-4-hydroxy-2-((4-(4-methylthiazol-5-yl)benzyl)-carbamoyl)pyrrolidin-1-yl)-3,3-dimethyl-1-oxobutan-2-yl)-nonanediamide (18)

^1^H NMR (400 MHz, CD_3_OD): *δ*_H_ ppm 8.85 (s, 1 H), 7.97 (d, *J* = 8.7 Hz, 2 H), 7.72 (d, *J* = 8.7 Hz, 2 H), 7.49 (d, *J* = 2.2 Hz, 1 H), 7.41−7.46 (m, 2 H), 7.37−7.41 (m, 2 H), 7.34 (dd, *J* = 8.3, 2.2 Hz, 1 H), 7.22 (dd, *J* = 5.1, 1.0 Hz, 1 H), 7.20 (dd, *J* = 3.7, 1.0 Hz, 1 H), 7.01 (dd, *J* = 5.1, 3.7 Hz, 1 H), 6.90 (d, *J* = 8.3 Hz, 1 H), 4.63 (s, 1 H), 4.55−4.61 (m, 1 H), 4.51 (d, *J* = 15.4 Hz, 1 H), 4.46−4.49 (m, 1 H), 4.34 (d, *J* = 15.4 Hz, 1 H), 3.86−3.93 (m, 1 H), 3.74−3.82 (m, 1 H), 2.45 (s, 3 H), 2.39 (t, *J* = 7.5 Hz, 2 H), 2.16−2.31 (m, 3 H), 2.02−2.11 (m, 1 H), 1.70 (quin, *J* = 7.5 Hz, 2 H), 1.60 (quin, *J* = 7.0 Hz, 2 H), 1.31−1.40 (m, 6 H), 1.03 (s, 9 H). ^13^C NMR (101 MHz, CD_3_OD): *δ*_C_ ppm 176.2, 175.1, 174.6, 172.5, 168.4, 153.0, 149.1, 145.8, 143.75, 143.7, 140.4, 133.6, 131.6, 130.5, 130.4, 130.0, 129.1, 129.0, 126.6, 126.2, 125.5, 125.3, 124.3, 122.7, 120.5, 118.9, 71.2, 61.0, 59.1, 58.2, 43.8, 39.1, 38.2, 36.75, 36.7, 30.3, 30.25, 30.2, 27.2, 27.1, 26.8, 16.0. HRMS (ESI) *m*/*z*: [M + H]^+^ calcd for C_48_H_58_N_7_O_6_S_2_, 892.3890; found, 892.3889.

#### N1-(4-((2-Amino-5-(thiophen-2-yl)phenyl)carbamoyl)phenyl)-N12-((S)-1-((2S,4R)-4-hydroxy-2-((4-(4-methylthiazol-5-yl)benzyl)-carbamoyl)pyrrolidin-1-yl)-3,3-dimethyl-1-oxobutan-2-yl)-dodecanediamide (19)

^1^H NMR (400 MHz, Methanol-*d*_*4*_): *δ*_H_ ppm 8.86 (s, 1 H), 7.98 (d, *J* = 8.7 Hz, 2 H), 7.73 (d, *J* = 8.7 Hz, 2 H), 7.49 (d, *J* = 2.1 Hz, 1 H), 7.43−7.47 (m, 2 H), 7.38−7.42 (m, 2 H), 7.35 (dd, *J* = 8.3, 2.1 Hz, 1 H), 7.16−7.26 (m, 2 H), 7.01 (dd, *J* = 5.1, 3.6 Hz, 1 H), 6.90 (d, *J* = 8.3 Hz, 1 H), 4.63 (s, 1 H), 4.55−4.60 (m, 1 H), 4.52 (d, *J* = 15.5 Hz, 1 H), 4.46−4.50 (m, 1 H), 4.34 (d, *J* = 15.5 Hz, 1 H), 3.86−3.92 (m, 1 H), 3.75−3.81 (m, 1 H), 2.46 (s, 3 H), 2.40 (t, *J* = 7.4 Hz, 2 H), 2.18−2.30 (m, 3 H), 2.03−2.12 (m, 1 H), 1.70 (quin, *J* = 7.4 Hz, 2 H), 1.54−1.63 (m, 2 H), 1.30−1.38 (m, 12 H), 1.03 (s, 9 H). ^13^C NMR (101 MHz, CD_3_OD): *δ*_C_ ppm 176.2, 175.1, 174.6, 172.5, 168.5, 153.0, 149.2, 145.8, 143.8, 143.7, 140.4, 133.6, 131.6, 130.5, 130.4, 130.0, 129.1, 129.0, 126.6, 126.2, 125.5, 125.3, 124.3, 122.7, 120.5, 118.9, 71.2, 61.0, 59.1, 58.2, 43.8, 39.1, 38.2, 36.8, 36.7, 30.6 (2 C), 30.5 (2 C), 30.45, 30.4, 27.2, 27.1, 26.9, 16.0. HRMS (ESI) *m*/*z*: [M + H]^+^ calcd for C_51_H_64_N_7_O_6_S_2_, 934.4359; found, 934.4355.

#### (2S,4R)-1-((S)-14-((4-((2-Amino-5-(thiophen-2-yl)phenyl)-carbamoyl)phenyl)amino)-2-(tert-butyl)-4,14-dioxo-6,9,12-trioxa-3-azatetradecanoyl)-4-hydroxy-N-(4-(4-methylthiazol-5-yl)benzyl)-pyrrolidine-2-carboxamide (20)

^1^H NMR (400 MHz, CD_3_OD): *δ*_H_ ppm 8.84 (s, 1 H), 7.98 (d, *J* = 8.7 Hz, 2 H), 7.75 (d, *J* = 8.7 Hz, 2 H), 7.49 (d, *J* = 2.1 Hz, 1 H), 7.40−7.44 (m, 2 H), 7.36−7.39 (m, 2 H), 7.34 (dd, *J* = 8.3, 2.1 Hz, 1 H), 7.22 (d, *J* = 5.0 Hz, 1 H), 7.19 (d, *J* = 3.7 Hz, 1 H), 7.01 (dd, *J* = 5.0, 3.7 Hz, 1 H), 6.89 (d, *J* = 8.3 Hz, 1 H), 4.68 (s, 1 H), 4.54−4.60 (m, 1 H), 4.51 (d, *J* = 15.5 Hz, 1 H), 4.46−4.49 (m, 1 H), 4.31 (d, *J* = 15.5 Hz, 1 H), 4.15 (d, *J* = 15.8 Hz, 1 H), 4.09 (d, *J* = 15.8 Hz, 1 H), 4.02 (d, *J* = 15.6 Hz, 1 H), 3.91 (d, *J* = 15.6 Hz, 1 H), 3.82−3.87 (m, 1 H), 3.69−3.81 (m, 9 H), 2.45 (s, 3 H), 2.16−2.24 (m, 1 H), 2.03−2.11 (m, 1 H), 1.02 (s, 9 H). ^13^C NMR (101 MHz, CD_3_OD): *δ*_C_ ppm 174.5, 172.0, 171.7, 171.6, 168.4, 153.0, 149.2, 145.8, 143.8, 142.5, 140.4, 133.5, 131.7, 131.2, 130.5, 130.0, 129.1, 129.0, 126.5, 126.2, 125.4, 125.3, 124.3, 122.7, 121.2, 118.9, 72.3, 72.25, 71.7, 71.6, 71.55, 71.2, 71.1, 61.0, 58.2, 43.9, 39.1, 37.8, 37.4, 27.1, 16.0. HRMS (ESI) *m*/*z*: [M + H]^+^ calcd for C_47_H_56_N_7_O_9_S_2_, 926.3581; found, 926.3556.

### Synthesis of Intermediate 56 as Described in Scheme 2

To a solution of acid linker **55** (332.6 mg, 1.19 mmol) in dry DMF (4 mL) at 0 °C, DIPEA (0.48 mL, 2.75 mmol) and HATU (522.7 mg, 1.37 mmol) were added. The reaction mixture was stirred for 15 min, after which a solution of **35a** (300.0 mg, 0.92 mmol) in DMF (2 mL) was added slowly, and the resultant solution was stirred at room temperature overnight. The reaction mixture was diluted in EtOAc (40 mL) and then washed with sat. NaHCO_3_ (2 × 20 mL) and sat. NaCl (2 × 20 mL). The organic layer was dried over MgSO_4_, filtered, and concentrated in vacuo to give the corresponding crude, which was purified by column chromatography (0−50% EtOAc in hexane) to give **56** (207.0 mg, 0.35 mmol, 38% yield) as a white solid.

#### tert-Butyl (2-(4-(12-Bromododecanamido)benzamido)phenyl)-carbamate (56)

^1^H NMR (400 MHz, CDCl_3_): *δ*_H_ ppm 9.25 (br s, 1 H), 7.92 (br s, 1 H), 7.86 (d, *J* = 8.7 Hz, 2 H), 7.68 (dd, *J* = 7.6, 1.8 Hz, 1 H), 7.58 (d, *J* = 8.7 Hz, 2 H), 7.29 (d, *J* = 7.6, 1.8 Hz, 1 H), 7.09−7.18 (m, 3 H), 3.40 (t, *J* = 6.9 Hz, 2 H), 2.34 (t, *J* = 7.6 Hz, 2 H), 1.85 (quin, *J* = 7.1 Hz, 2 H), 1.69 (quin, *J* = 7.4 Hz, 2 H), 1.50 (s, 9 H), 1.38−1.46 (m, 2 H), 1.26−1.35 (m, 12 H). HRMS (ESI) *m*/*z*: [M + H]^+^ calcd for C_30_H_43_^81^BrN_3_O_4_, 590.2416; found, 590.2411.

### Synthesis of Compound 21

A mixture of (2*S*,4*R*)-1-((*S*)-2-acetamido-3,3-dimethylbutanoyl)-4-hydroxy-*N*-(2-hydroxy-4-(4-methylthiazol-5-yl)benzyl)pyrrolidine-2-carboxamide (**VH_032 phenol**, 17.2 mg, 0.034 mmol), **56** (20.0 mg, 0.034 mmol), and K_2_CO_3_ (3 equiv) in dry DMF (0.8 mL) was stirred at 70 °C overnight. The reaction mixture was concentrated in vacuo to give the corresponding crude, which was purified by column chromatography (0−10% MeOH in DCM) to afford **57a** (17.8 mg, 0.018 mmol, 52% yield) as a white solid.

TFA (0.4 mL) was added to a stirring solution of Boc-protected PROTAC **57a** (17.8 mg, 0.018 mmol) in DCM (2 mL), and the resulting reaction mixture was stirred at room temperature for 4 h. The reaction mixture was concentrated in vacuo, dissolved in MeOH (2 mL), agitated in MP-carbonate resin (3.02 mmol/g loading capacity) for 3 h, and then filtered. The filtrate was concentrated in vacuo, and the resulting solid was dissolved in MeCN/H_2_O (1:1) and lyophilized to remove residual TFA impurities, affording **21** (16.1 mg, 0.018 mmol, 99% yield) as a white solid.

#### (2S,4R)-1-((S)-2-Acetamido-3,3-dimethylbutanoyl)-N-(2-((12-((4-((2-aminophenyl)carbamoyl) phenyl)amino)-12-oxododecyl)oxy)-4-(4-methylthiazol-5-yl)benzyl)-4-hydroxypyrrolidine-2-carboxamide (21)

^1^H NMR (400 MHz, CD_3_OD): *δ*_H_ ppm 8.86 (s, 1 H), 7.95 (d, *J* = 8.7 Hz, 2 H), 7.72 (d, *J* = 8.7 Hz, 2 H), 7.47 (d, *J* = 8.2 Hz, 1 H), 7.17 (dd, *J* = 7.7, 1.3 Hz, 1 H), 7.07 (app. td, *J* = 7.7, 1.3 Hz, 1 H), 6.94−7.00 (m, 2 H), 6.90 (dd, *J* = 7.7, 1.3 Hz, 1 H), 6.76 (app. td, *J* = 7.7, 1.3 Hz, 1 H), 4.57−4.65 (m, 2 H), 4.48−4.52 (m, 1 H), 4.46 (d, *J* = 16.1 Hz, 1 H), 4.39 (d, *J* = 16.1 Hz), 4.05 (t, *J* = 6.3 Hz, 2 H), 3.86−3.92 (m, 1 H), 3.75−3.81 (m, 1 H), 2.48 (s, 3 H), 2.40 (t, *J* = 7.5 Hz, 2 H), 2.17−2.25 (m, 1 H), 2.07−2.15 (m, 1 H), 1.99 (s, 3 H), 1.78−1.87 (m, 2 H), 1.71 (quin, *J* = 7.3 Hz, 2 H), 1.46−1.56 (m, 2 H), 1.32−1.42 (m, 12 H), 1.02 (s, 9 H). ^13^C NMR (101 MHz, CD_3_OD): *δ*_C_ ppm 175.1, 174.6, 173.3, 172.5, 168.4, 158.1, 152.9, 149.2, 143.9, 143.7, 133.8, 132.8, 130.5, 129.9, 129.7, 128.6, 128.1, 127.8, 125.6, 122.5, 120.4, 119.8, 118.9, 113.1, 71.2, 69.5, 60.9, 59.3, 58.1, 39.4, 39.0, 38.2, 36.6, 30.8, 30.75, 30.7, 30.6, 30.55, 30.5, 30.4, 27.4, 27.1, 26.9, 22.5, 16.1. HRMS (ESI) *m*/*z*: [M + H]^+^ calcd for C_49_H_66_N_7_O_7_S, 896.4744; found, 896.4744.

### Synthesis of Compound 22

A mixture of (2*S*,4*R*)-1-((*S*)-2-(1-fluorocyclopropane-1-carboxamido)-3,3-dimethylbutanoyl)-4-hydroxy-*N*-(2-hydroxy-4-(4-methylthiazol-5-yl)benzyl)pyrrolidine-2-carboxamide (**VH_101 phenol**, 18.6 mg, 0.034 mmol), **56** (20.0 mg, 0.034 mmol), and K_2_CO_3_ (3 equiv) in dry DMF (0.8 mL) was stirred at 70 °C overnight. The reaction was concentrated in vacuo to give the corresponding crude, which was purified by column chromatography (1−10% MeOH in DCM) to afford **57b** (24.2 mg, 0.022 mmol, 64% yield) as a white solid.

TFA (0.4 mL) was added to a stirring solution of Boc-protected PROTAC **57b** (24.2 mg, 0.022 mmol) in DCM (2 mL), and the resulting reaction mixture was stirred at room temperature for 4 h. The reaction mixture was concentrated in vacuo, dissolved in MeOH (2 mL), agitated in MP-carbonate resin (3.02 mmol/g loading capacity) for 3 h, and then filtered. The filtrate was concentrated in vacuo and then purified by column chromatography (2−5% MeOH in DCM) to afford **22** (11.7 mg, 0.012 mmol, 57% yield) as a white solid.

#### (2S,4R)-N-(2-((12-((4-((2-Aminophenyl)carbamoyl)phenyl)-amino)-12-oxododecyl)oxy)-4-(4-methylthiazol-5-yl)benzyl)-1-((S)-2-(1-fluorocyclopropane-1-carboxamido)-3,3-dimethylbutanoyl)-4-hydroxypyrrolidine-2-carboxamide (22)

^1^H NMR (400 MHz, CD_3_OD): *δ*_H_ ppm 8.86 (s, 1 H), 7.95 (d, *J* = 8.7 Hz, 2 H), 7.72 (d, *J* = 8.7 Hz, 2 H), 7.47 (d, *J* = 7.7 Hz, 1 H), 7.17 (dd, *J* = 7.8, 1.3 Hz, 1 H), 7.06 (app. td, *J* = 7.8, 1.3 Hz, 1 H), 6.96−7.02 (m, 2 H), 6.90 (dd, *J* = 7.8, 1.3 Hz, 1 H), 6.76 (app. td, *J* = 7.8, 1.3 Hz, 1 H), 4.74 (d, *J*_HF_ = 0.8 Hz, 1 H), 4.60−4.67 (m, 1 H), 4.49−4.53 (m, 1 H), 4.47 (d, *J* = 16.0 Hz, 1 H), 4.39 (d, *J* = 16.0 Hz, 1 H), 4.06 (t, *J* = 6.3 Hz, 2 H), 3.82−3.88 (m, 1 H), 3.76−3.81 (m, 1 H), 2.48 (s, 3 H), 2.40 (t, *J* = 7.5 Hz, 2 H), 2.19−2.27 (m, 1 H), 2.09−2.16 (m, 1 H), 1.78−1.89 (m, 2 H), 1.64−1.76 (m, 2 H), 1.48−1.57 (m, 2 H), 1.28−1.40 (m, 16 H), 1.04 (s, 9 H). ^13^C NMR (101 MHz, CD_3_OD): *δ*_C_ ppm 175.1, 174.4, 171.9, 171.6 (d, *J*_*CF*_ = 20.4 Hz), 168.4, 158.2, 152.9, 149.2, 143.9, 143.7, 133.8, 132.9, 130.5, 129.9, 129.7, 128.6, 128.1, 127.8, 125.6, 122.5, 120.4, 119.8, 118.9, 113.2, 79.3 (d, *J*_*CF*_ = 231.6 Hz), 71.2, 69.5, 60.9, 58.8, 58.3, 39.4, 39.0, 38.2, 37.5, 30.8, 30.75, 30.7, 30.6, 30.55, 30.5, 30.4, 27.4, 27.0, 26.9, 16.1, 14.1 (app. t, *J*_*CF*_ = 11.2 Hz). ^19^F NMR (376 MHz, CD_3_OD): *δ* ppm −199.4. HRMS (ESI) *m*/*z*: [M + H]^+^ calcd for C_51_H_67_FN_7_O_7_S, 940.4807; found, 940.4781.

### General Procedure for Preparing Compounds 23 and 24 as Described in Scheme 2, Showing Synthesis of 23 (JPS014) as an Example

To a solution of HDACi-linker acid **58** (19.7 mg, 0.043 mmol) in dry DMF (1 mL) at 0 °C, DIPEA (0.019 mL, 0.109 mmol) and HATU (18.0 mg, 0.047 mmol) were added. The reaction mixture was stirred for 15 min, after which a solution of (2*S*,4*R*)-1-((*S*)-2-acetamido-3,3-dimethylbutanoyl)-N-(2-(4-aminobutoxy)-4-(4-meth-ylthiazol-5-yl)benzyl)-4-hydroxypyrrolidine-2-carboxamide (**VH032 phenol-alkylC4-amine**, 24.0 mg, 0.034 mmol) in DMF (1 mL) was added slowly, and the resultant solution was stirred at room temperature overnight. The reaction mixture was diluted in EtOAc (10 mL) and then washed with sat. NaHCO_3_ (2 × 5 mL) and sat. NaCl (2 × 5 mL). The organic layer was dried over MgSO_4_, filtered, and concentrated in vacuo to give the corresponding crude, which was purified by column chromatography (2−10% MeOH in DCM) to afford **59a** (28.5 mg, 0.028 mmol, 83% yield) as a white solid.

TFA (0.4 mL) was added to a stirring solution of Boc-protected PROTAC **59a** (22.9 mg, 0.023 mmol) in DCM (2 mL), and the resulting reaction mixture was stirred at room temperature for 4 h. The reaction mixture was concentrated in vacuo, dissolved in MeOH (2 mL), agitated in MP-carbonate resin (3.02 mmol/g loading capacity) for 3 h, and then filtered. The filtrate was concentrated in vacuo, and the resulting solid was dissolved in MeCN/H_2_O (1:1) and lyophilized to remove residual TFA impurities, affording **23** (20.2 mg, 0.022 mmol 97% yield) as a pale-yellow solid.

#### N1-(4-(2-(((2S,4R)-1-((S)-2-Acetamido-3,3-dimethylbutanoyl)-4-hydroxypyrrolidine-2-carboxamido)methyl)-5-(4-methylthiazol-5-yl)phenoxy)butyl)-N6-(4-((2-aminophenyl)carbamoyl)phenyl)-adipamide (23)

^1^H NMR (400 MHz, CD_3_OD): *δ*_H_ ppm 8.85 (s, 1 H), 7.92 (d, *J* = 8.8 Hz, 2 H), 7.70 (d, *J* = 8.8 Hz, 2 H), 7.47 (d, *J* = 7.8 Hz, 1 H), 7.17 (dd, *J* = 7.7, 1.3 Hz, 1 H), 7.07 (app. td, *J* = 7.7, 1.3 Hz, 1 H), 6.94−7.00 (m, 2 H), 6.90 (dd, *J* = 7.7, 1.3 Hz, 1 H), 6.76 (app. td, *J* = 7.7, 1.3 Hz, 1 H), 4.58−4.64 (m, 2 H), 4.48−4.51 (m, 1 H), 4.45 (d, *J* = 15.9 Hz, 1 H), 4.39 (d, *J* = 15.9 Hz, 1 H), 4.07 (t, *J* = 6.1 Hz, 2 H), 3.85−3.93 (m, 1 H), 3.74−3.81 (m, 1 H), 3.27 (t, *J* = 7.0 Hz, 2 H), 2.47 (s, 3 H), 2.42 (t, *J* = 6.9 Hz, 2 H), 2.23 (t, *J* = 6.9 Hz, 2 H), 2.16−2.21 (m, 1 H), 2.06−2.14 (m, 1 H), 1.99 (s, 3 H), 1.82−1.91 (m, 2 H), 1.66−1.78 (m, 6 H), 1.01 (s, 9 H). ^13^C NMR (101 MHz, CD_3_OD): *δ*_C_ ppm 176.0, 174.6, 174.55, 173.3, 172.5, 168.4, 158.1, 153.0, 149.2, 143.9, 143.6, 133.8, 132.9, 130.5, 129.9, 129.8, 128.6, 128.1, 127.8, 125.6, 122.6, 120.4, 119.8, 118.9, 113.1, 71.2, 69.0, 60.9, 59.3, 58.1, 40.2, 39.4, 39.0, 37.9, 37.0, 36.6, 27.9, 27.3, 27.1, 26.8, 26.5, 22.5, 16.1. HRMS (ESI) *m*/*z*: [M + H]^+^ calcd for C_47_H_61_N_8_O_8_S, 897.4333; found, 897.4324.

### Compound 24 Prepared as Described in Scheme 2

#### N1-(4-(2-(((2S,4R)-1-((S)-2-Acetamido-3,3-dimethylbutanoyl)-4-hydroxypyrrolidine-2-carboxamido)methyl)-5-(4-methylthiazol-5-yl)-phenoxy)butyl)-N9-(4-((2-aminophenyl)carbamoyl)phenyl)-nonanediamide (24)

^1^H NMR (400 MHz, CD_3_OD): *δ*_H_ ppm 8.85 (s, 1 H), 7.93 (d, *J* = 8.7 Hz, 2 H), 7.71 (d, *J* = 8.7 Hz, 2 H), 7.47 (d, *J* = 8.2 Hz, 1 H), 7.17 (dd, *J* = 7.7, 1.3 Hz, 1 H), 7.07 (app. td, *J* = 7.7, 1.3 Hz, 1 H), 6.95−6.99 (m, 2 H), 6.90 (dd, *J* = 7.7, 1.3 Hz, 1 H), 6.76 (app. td, *J* = 7.7, 1.3 Hz, 1 H), 4.57−4.64 (m, 2 H), 4.48−4.52 (m, 1 H), 4.45 (d, *J* = 15.9 Hz, 1 H), 4.39 (d, *J* = 15.9 Hz, 1 H), 4.07 (t, *J* = 6.1 Hz, 2 H), 3.85−3.93 (m, 1 H), 3.74−3.81 (m, 1 H), 3.26 (t, *J* = 6.8 Hz, 2 H), 2.48 (s, 3 H), 2.38 (t, *J* = 7.4 Hz, 2 H), 2.14−2.22 (m, 3 H), 2.06−2.14 (m, 1 H), 1.99 (s, 3 H), 1.81−1.90 (m, 2 H), 1.66−1.76 (m, 4 H), 1.60 (quin, *J* = 7.2 Hz, 2 H), 1.33−1.40 (m, 6 H), 1.01 (s, 9 H). ^13^C NMR (101 MHz, CD_3_OD): *δ*_C_ ppm 176.4, 175.0, 174.6, 173.2, 172.5, 168.4, 158.0, 152.9, 149.2, 143.9, 143.6, 133.8, 132.9, 130.5, 129.9, 129.8, 128.6, 128.1, 127.8, 125.6, 122.6, 120.4, 119.8, 118.9, 113.1, 71.2, 69.0, 60.9, 59.3, 58.1, 40.1, 39.4, 39.0, 38.2, 37.3, 36.6, 30.3, 30.25, 30.2, 27.9, 27.3, 27.2, 27.1, 26.8, 22.5, 16.1. HRMS (ESI) *m*/*z*: [M + H]^+^ calcd for C_50_H_67_N_8_O_8_S, 939.4803; found, 939.4764.

### Cell Lines and Cell Culture

HCT116 human colon carcinoma cells were grown in Dulbecco’s modified Eagle medium (GIBCO, 41965-039) supplemented with 10% fetal bovine serum (Sigma) and 1X glutamine/penicillin/streptomycin (GIBCO, 10378−016). This cell line was incubated at 37 °C with 5% CO_2_. Cells were treated with PROTACs (0.01−10 *μ*M) alongside HDACi CI-994 (10 *μ*M).

### Western Blotting

HCT116 cells were seeded into 6-well plates (4 × 10^5^ cells/well for 24 h, 2 × 10^5^ cells/well for 48 h) for 24 h and then treated with DMSO or compounds at the indicated concentrations in fresh medium (5 mL total). After desired treatment time, the cells were harvested and then lysed in lysis buffer (50 mM Tris-HCl, 150 mM NaCl, 0.5% NP-40, 0.5% Triton X-100) supplemented with a protease inhibitor (Sigma, P8340). The suspension was incubated on ice for 30 min and centrifuged (18,000 rcf, 15 min, 4 °C); then, the supernatant was collected, and protein concentrations were quantified via Bradford Assay using Protein Assay Dye Reagent Concentrate (BIO-RAD). For histone extraction, an equal volume of 0.4 N H_2_SO_4_ was added to the pellets, and the extracts were placed at 4 °C overnight and centrifuged (18,000 rcf, 15 min, 4 °C), and then, the supernatant (histone extract) was collected. Western blots were run on NuPAGE 4−12% bis−Tris gels with 30 *μ*g of protein or 10 *μ*L of acid-extracted histone loaded per lane, using NuPAGE LDS sample buffer (4×). PageRuler Plus Prestained Ladder was used for size standards. After gel electrophoresis at 140 V for 90 min, the separated proteins were transferred onto a nitrocellulose membrane at 30 V for 60 min. The membranes were probed with primary antibodies (see the Supporting Information) for 60−90 min. Blots were developed with complimentary IRDye-conjugated secondary antibodies, and the bands were visualized using an Odyssey infrared imaging system. Image processing and band intensity quantification were performed using Image Studio Lite.

### CellTiter-Glo 2.0 Cell Viability Assay

Exponentially growing HCT116 cells (ATCC) were seeded at 3000 cells/well in 100 *μ*L medium into white, flat-bottomed, 96-well tissue culture plates (655083, Greiner Bio-One GmbH, Frickenhausen, Germany). The plates were incubated for 24 h and then treated with a range of concentrations of compounds (0.1−100 *μ*M) in fresh medium, in triplicate wells, at 1 or 0.1% DMSO (vehicle control = 1% or 0.1% DMSO) for 24, 48, or 72 h, in a total volume of 100 *μ*L. CellTiter-Glo 2.0 Reagent (Promega UK Ltd, G9242) was equilibrated to room temperature and added to the wells in a ratio of 1 volume reagent:5 volumes medium. Background luminescence was determined using medium alone plus reagent. The plates were shaken on an orbital shaker for 2 min to induce cell lysis and then incubated in the dark at room temperature for 10 min to stabilize the luminescent signal. Luminescence was captured on a CLARIOstar plate reader (BMG Labtech Ltd, UK) at 22 °C to give relative luminescence units and analyzed using BMG Labtech software. EC_50_ values were calculated by nonlinear regression (Hill plot) and log (inhibitor) versus response—variable slopes (four parameters) using GraphPad Prism 9 software. Each compound dose response was repeated as four independent biological replicates (*n* = 4).

### Apoptosis Flow Cytometry Assay

HCT116 cells were seeded (5 × 10^5^ cells/plate) into 6 cm tissue culture plates for 24 h and then treated with 10 *μ*M compounds (with 0.1% DMSO vehicle) or 0.1% DMSO control in fresh medium (5 mL total). The cells were then exposed to the compounds (10 *μ*M) for 24 or 48 h. To harvest the cells, the medium was transferred from each sample plate to 15 mL centrifuge tubes. Each well was washed with 1 mL of phosphate-buffered saline (PBS) and the PBS was transferred to the corresponding 15 mL centrifuge tube. The plates were trypsinized, 2 mL of medium was added, and the cells and medium were transferred to the corresponding 15 mL centrifuge tubes. The harvested cells were pelleted by centrifugation (200*g*, 5 min, 4 °C), washed with 1 mL PBS, and then pelleted again by centrifugation (200*g*, 5 min, 4 °C). The supernatant was discarded, and the pellets resuspended in 1 mL of 70% ethanol (ice-cold) to fix the cells. Samples were stored at 4 °C and analyzed within two weeks. PBS (1 mL) was added to each sample tube containing cells fixed in 70% ethanol. The cells were pelleted by centrifugation (200*g*, 5 min, 4 °C), and the PBS wash was repeated. The supernatant was discarded, and the pellet was resuspended in 50 *μ*L of RNase A (1 *μ*g/mL, Thermo Scientific, EN0531) and left at room temperature for 10 min to digest contaminating RNA. A total of 500 *μ*L of PI stain (50 *μ*g/mL, Invitrogen, P3566) was added to each tube, and the tubes were incubated in the dark, at room temperature, for 30 min. Results were acquired on a BD FACSCanto II flow cytometer and analyzed using BD FACSdiva software. Sub-G1 population was calculated as a percentage of total cell population. Two independent biological replicates were performed.

### RNA Sequencing (Seq)

RNA-seq analysis was performed in HCT116 cells treated with PROTACs (JPS004, JPS014, JPS016, JPS035, and JPS036) for 24 h at 10 *μ*M concentration. Total RNA was isolated using a Tri-reagent RNA miniprep kit (Zymogen; R2053), before RNA integrity, size, and purity were assessed using an Agilent Bioanalyzer. Library preparation and sequencing were performed by Novogene, using a NovaSeq 6000 PE150 platform at a read depth of 20 million. For bioinformatics analysis, see the Supporting Information. RNA-seq data from the study was deposited at the GEO database (GSE197985).

## Supplementary Material

**Supporting Information**

The Supporting Information is available free of charge at https://pubs.acs.org/doi/10.1021/acs.jmedchem.1c02179.

Chemical synthesis, full characterization data, western blots, and biochemical protocols (PDF)

Molecular formula strings (SMILES) and biological data of **1–24** (CSV)

Supplementary Information

## Abbreviations

CDK1: cyclin-dependent kinase 1
CDKN1A: cyclin-dependent kinase inhibitor 1A
CDKN2B: cyclin-dependent kinase inhibitor 2B
CDKN1C: cyclin-dependent kinase inhibitor 1C
CoREST: corepressor of repressor element1 silencing transcription factor
E2F1: E2F transcription factor 1
H3K56ac: histone3 lysine56 acetylation
HDAC: histone deacetylase
HDAC1/2: histone deacetylase 1 and histone deacetylase 2
HDAC2: histone deacetylase 2
HDAC3: histone deacetylase 3
HDACi: HDAC inhibitor
LSD1: lysine-specific demethylase 1
MiDAC: mitotic deacetylase complex
NuRD: nucleosome remodeling deacetylase
NCoR 1: nuclear receptor corepressor
PEG: poly ethylene glycol
POI: protein of interest
PROTAC: proteolysis targeting chimera
Sin3: switchindependent protein 3
SMRT: silencing mediator of retinoic acid
VHL: Von Hippel−Lindau
