# Supplementary material for: Optimization of Class I Histone Deacetylase PROTACs Reveals that HDAC1/2 Degradation is Critical to Induce Apoptosis and Cell Arrest in Cancer Cells
Source: J Med Chem. Author manuscript; Available in PMC 2025 Dec 15. (PMC9014412; doi:10.1021/acs.jmedchem.1c02179)
Supplement: Supplementary Information [file EMS211470-supplement-Supplementary_Information.pdf]

Supporting Information

Optimization of Class I Histone Deacetylase PROTACs Reveals HDAC1/2 Degradation is Critical to Induce Apoptosis and Cell Arrest in Cancer Cells

Joshua P. Smalley,[a] India M. Baker,[b] Wiktoria A. Pytel,[a] Li-Ying Lin,[c] Karen J. Bowman,[b] John W.R. Schwabe,[c] Shaun M. Cowley,[b*] and James T. Hodgkinson[a*]*

a. Leicester Institute of Structural and Chemical Biology, School of Chemistry, University of Leicester, Leicester, LE1 7RH, UK, E-mail: jthodgkinson@le.ac.uk

b. Department of Molecular and Cell Biology, University of Leicester, Leicester, LE1 7RH, UK, E-mail: smc57@leicester.ac.uk

c. Leicester Institute of Structural and Chemical Biology, Department of Molecular and Cell Biology, University of Leicester, Leicester, LE1 7RH, UK, E-mail: john.schwabe@le.ac.uk

Table of Contents

Supporting Information: Biology S3

1. Western Blotting S3

1.1. Western Blotting Analysis of Compounds **1-24** from Initial Screen S3

1.2. Representative Blots from Dose Response Experiment with **7**,**9**,**21**,**22** S8

1.3. Western Blotting Analysis from Further Experiments with **7** and **9** S11

3. Properties Table of Compounds **1-24** S14

2. *In Vitro* HDAC Assay with Class I HDAC Complexes S15

3. Apoptosis Flow Cytometry Assay S18

4. RNA Seq – Bioinformatics Analysis S18

Supporting Information: Chemistry S20

5. Materials and Methods S20

5.1. Preparation of HDAC Inhibitor (HDACi) Intermediates S22

5.2. Preparation of Carboxylic Acid Linker Intermediates S28

5.3. Preparation of Compounds **1-20** S36

5.4. Preparation of Compounds **21-24** S83

5.5. Preparation of JPS016 Negative Control (**25**) S90

6. UPLC Traces of Potent Degraders S93

References S96

Appendix: ^{1}H NMR and ^{13}C NMR of Novel PROTACs S97

Supporting Information: Biology

1. Western Blotting

Antibody Information

Primary Antibodies;

α-tubulin - Sigma, t5168 (1:10,000 dilution)

HDAC1 - Abcam, 109411 (1:2,000 dilution)

HDAC2 - Merck Millipore, 05-814 (1:2,000 dilution)

HDAC3 - Abcam, 32369 (1:2,000 dilution)

H3 - Merck Millipore, 05-499 (1:1,000 dilution)

H3K56Ac - Active Motif, 39082 (1:1,000 dilution)

LSD1 - Abcam, 129195 (1:10,000 dilution)

Sin3a - Abcam, 129087 (1:1,000 dilution)

Secondary Antibodies;

IRDye® 680LT - LI-COR Biosciences, 926-68023 (1:10,000 dilution)

IRDye® 800CW - LI-COR Biosciences, 926-32210 (1:10,000 dilution)

1.1. Western Blotting Analysis of Compounds **1-24** from Initial Screen

Figure S1. Western blotting of HDAC1/2/3 proteins in HCT116 cells treated with compounds 1-24. Representative blots of two independent biological replicates.

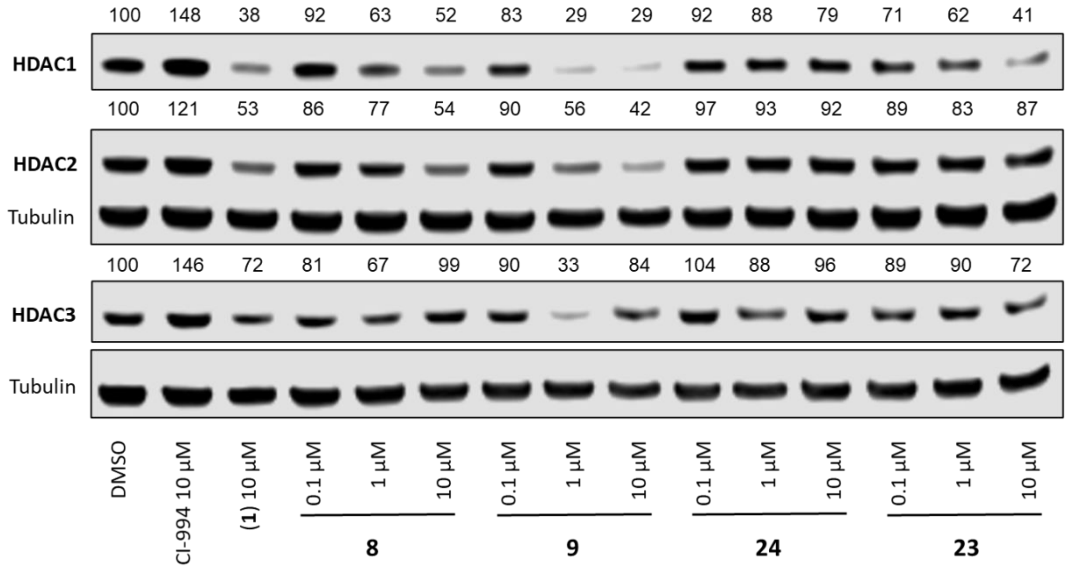

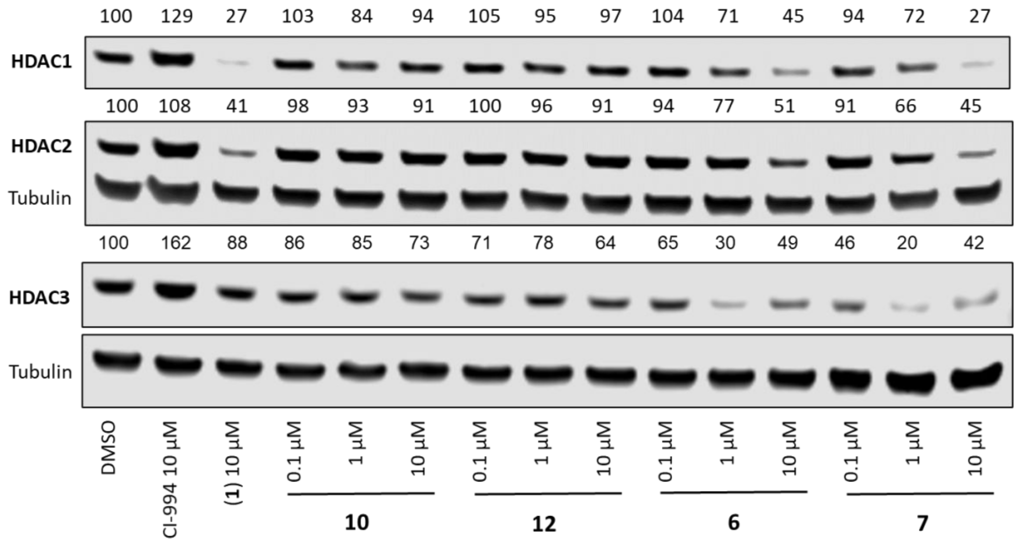
100 129 27 103 84 94 105 95 97 104 71 45 94 72 27
HDAC1
100 108 41 98 93 91 100 96 91 94 77 51 91 66 45
HDAC2
Tubulin
100 162 88 86 85 73 71 78 64 65 30 49 46 20 42
HDAC3
Tubulin
DMSO
CI-994 10 µM
(1) 10 µM
0.1 µM
1 µM
10 µM
10
12
6
7

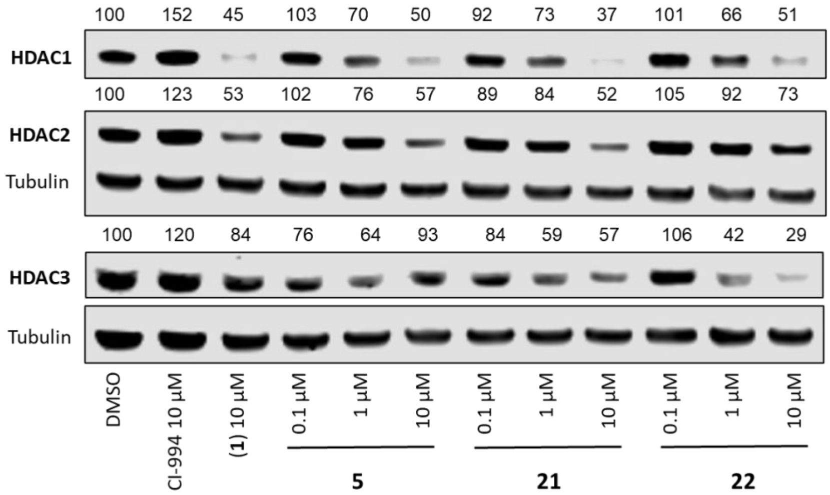
100 152 45 103 70 50 92 73 37 101 66 51
HDAC1
100 123 53 102 76 57 89 84 52 105 92 73
HDAC2
Tubulin
100 120 84 76 64 93 84 59 57 106 42 29
HDAC3
Tubulin
DMSO
CI-994 10 µM
(1) 10 µM
0.1 µM
1 µM
10 µM
5
21
22

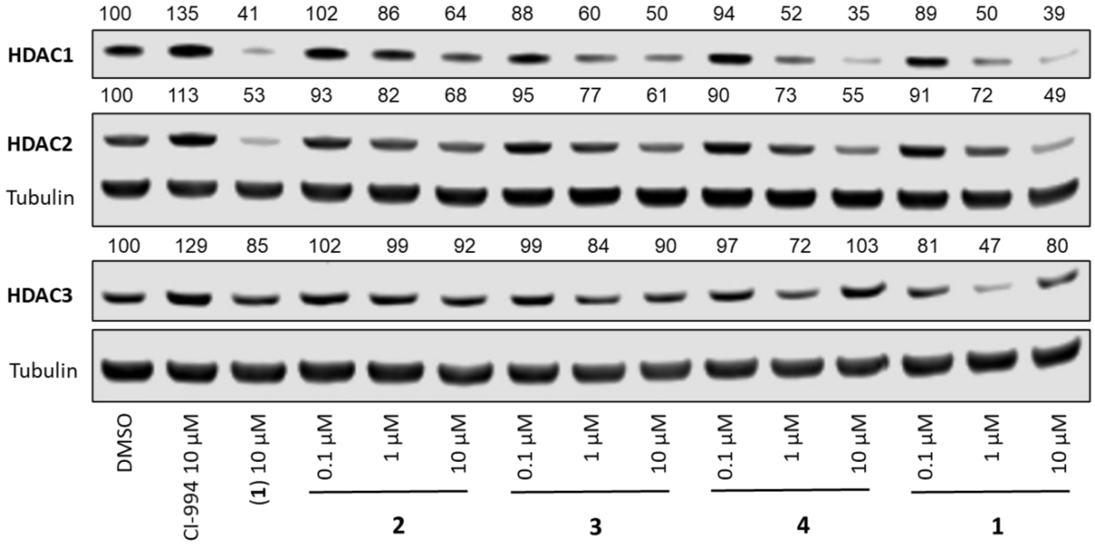
100 135 41 102 86 64 88 60 50 94 52 35 89 50 39
HDAC1
100 113 53 93 82 68 95 77 61 90 73 55 91 72 49
HDAC2
Tubulin
100 129 85 102 99 92 99 84 90 97 72 103 81 47 80
HDAC3
Tubulin
DMSO
CI-994 10 µM
(1) 10 µM
0.1 µM
1 µM
10 µM
2
3
4
1

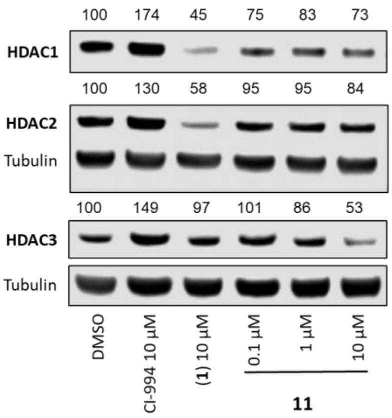
100 174 45 75 83 73
HDAC1
100 130 58 95 95 84
HDAC2
Tubulin
100 149 97 101 86 53
HDAC3
Tubulin
DMSO
CI-994 10 µM
(1) 10 µM
0.1 µM
1 µM
10 µM
11

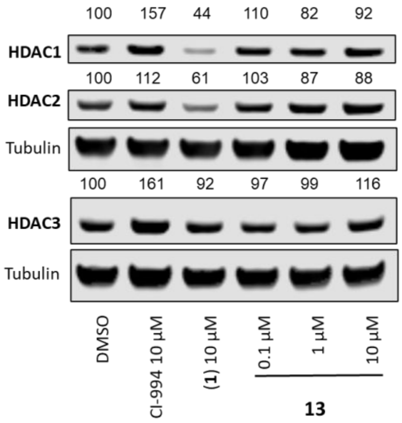
100 157 44 110 82 92
HDAC1
100 112 61 103 87 88
HDAC2
Tubulin
100 161 92 97 99 116
HDAC3
Tubulin
DMSO
CI-994 10 µM
(1) 10 µM
0.1 µM
1 µM
10 µM
13

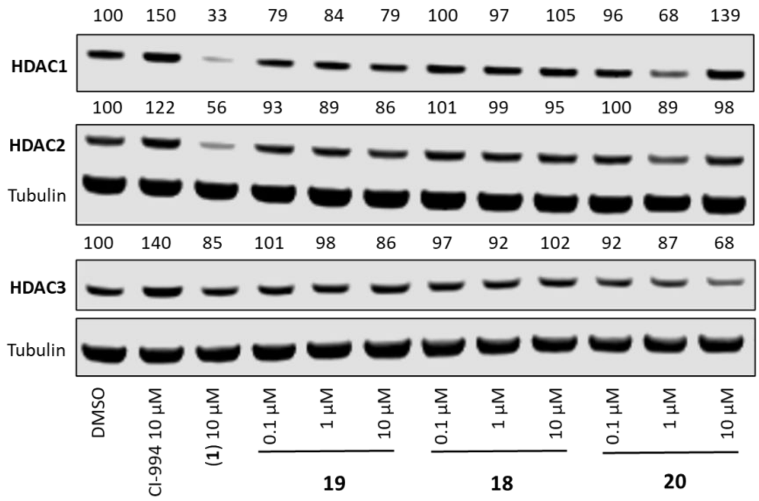
100 150 33 79 84 79 100 97 105 96 68 139
HDAC1
100 122 56 93 89 86 101 99 95 100 89 98
HDAC2
Tubulin
100 140 85 101 98 86 97 92 102 92 87 68
HDAC3
Tubulin
DMSO
CI-994 10 µM
(1) 10 µM
0.1 µM
1 µM
10 µM
0.1 µM
1 µM
10 µM
0.1 µM
1 µM
10 µM
19
18
20

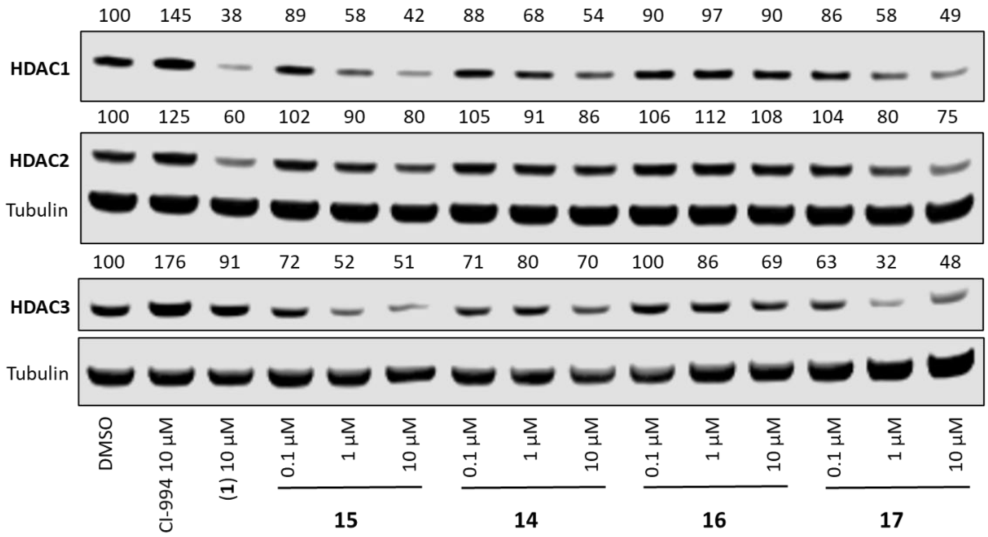
100 145 38 89 58 42 88 68 54 90 97 90 86 58 49
HDAC1
100 125 60 102 90 80 105 91 86 106 112 108 104 80 75
HDAC2
Tubulin
100 176 91 72 52 51 71 80 70 100 86 69 63 32 48
HDAC3
Tubulin
DMSO
CI-994 10 µM
(1) 10 µM
0.1 µM
1 µM
10 µM
0.1 µM
1 µM
10 µM
0.1 µM
1 µM
10 µM
0.1 µM
1 µM
10 µM
15
14
16
17

Figure S2. Western blotting of H3/H3K56ac in HCT116 cells treated with compounds 13-23, complete with representative graphs showing fold-change relative to DMSO. Error bars represent the average of two independent biological replicates.

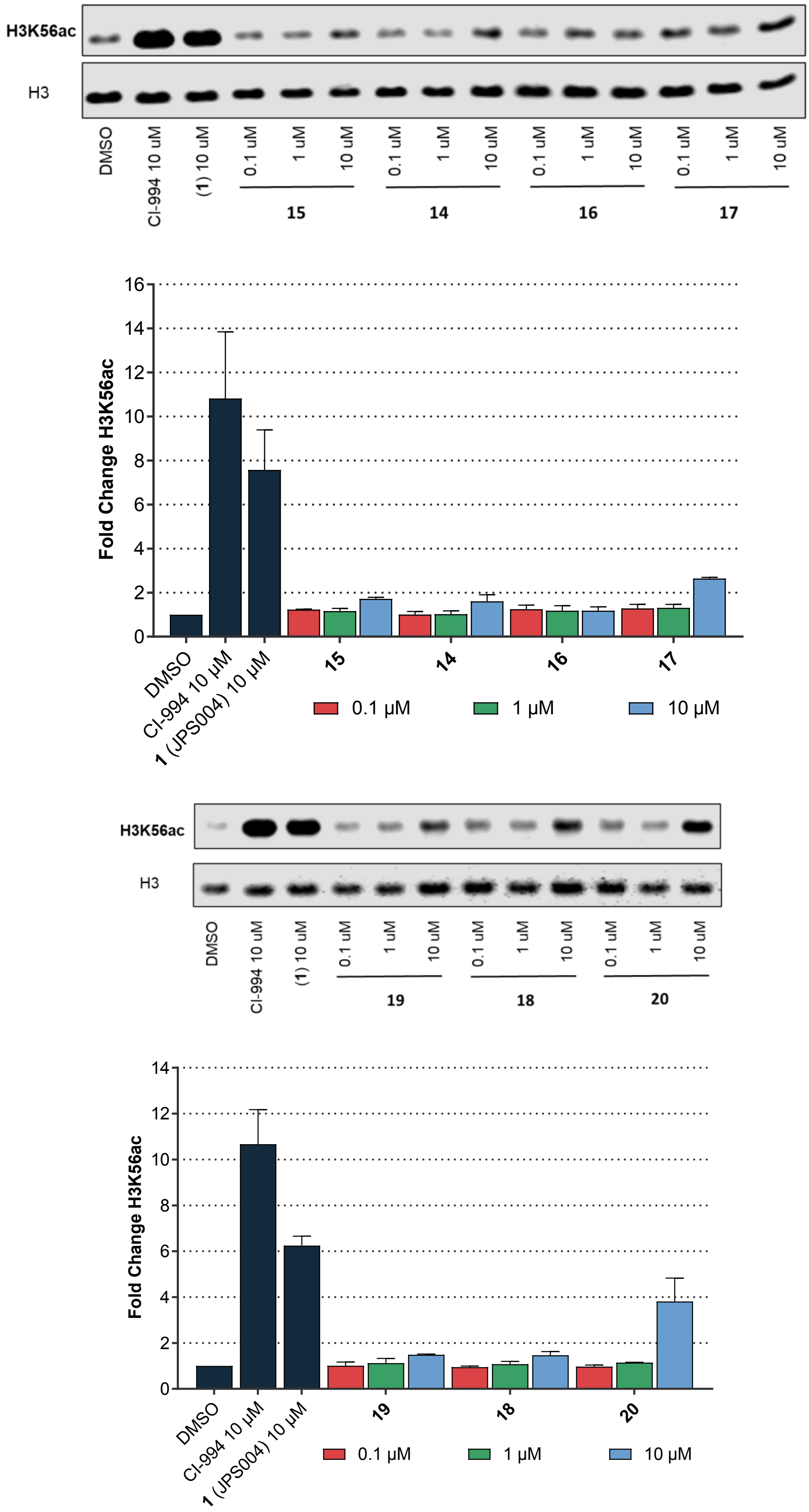

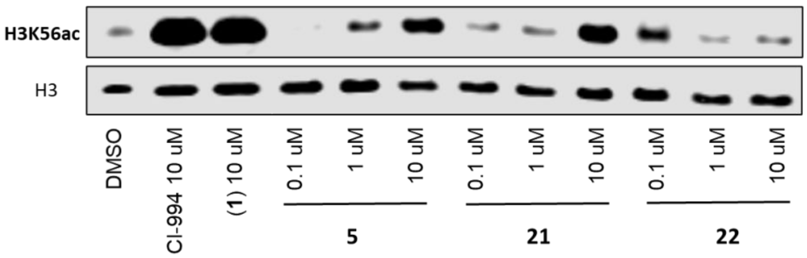
H3K56ac
H3
DMSO
CI-994 10 uM
(1) 10 uM
0.1 uM
1 uM
10 uM
5
21
22

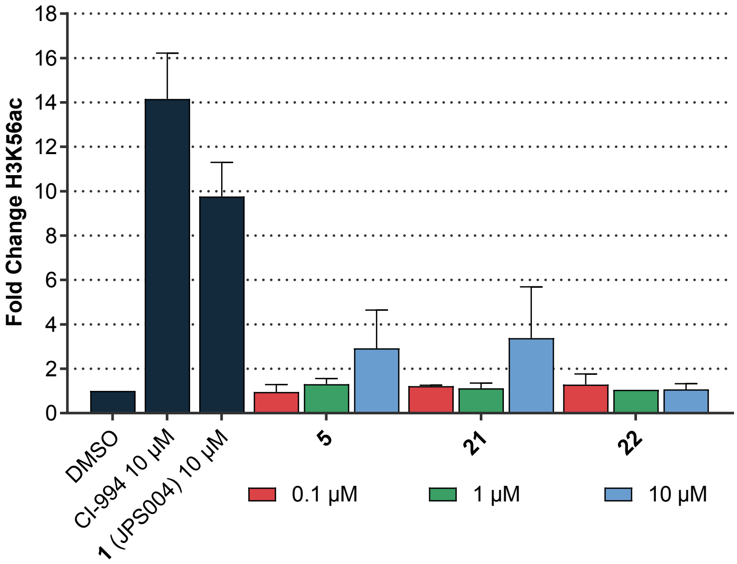
Fold Change H3K56ac
DMSO
CI-994 10 μM
1 (JPS004) 10 μM
5
21
22
0.1 μM
1 μM
10 μM

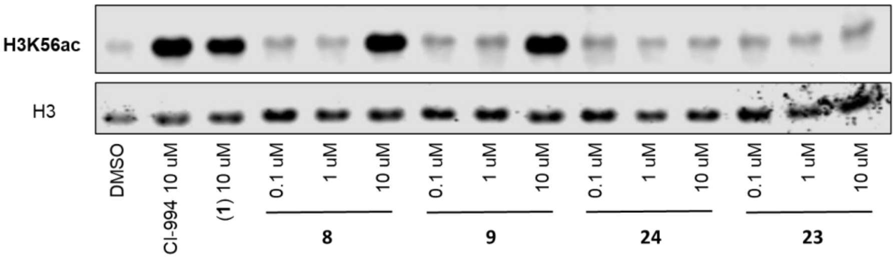
H3K56ac
H3
DMSO
CI-994 10 uM
(1) 10 uM
0.1 uM
1 uM
10 uM
8
9
24
23

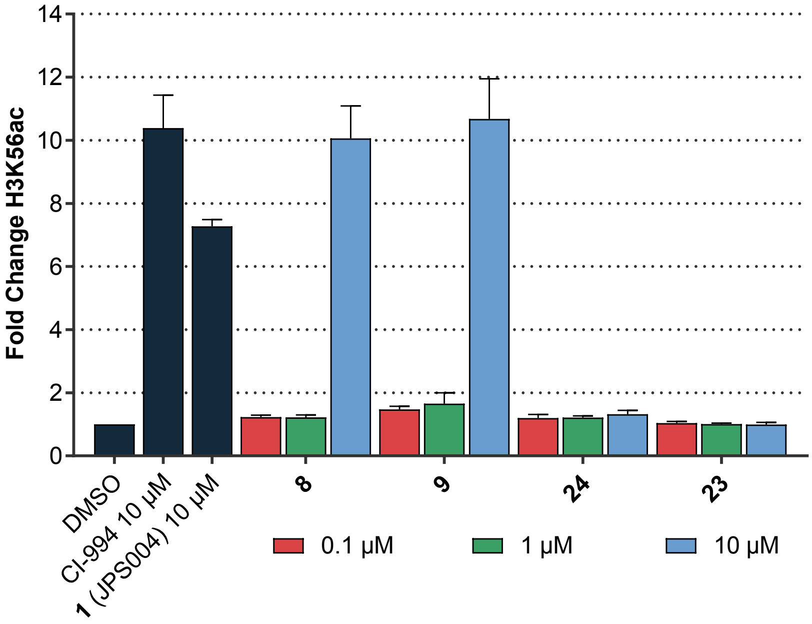
Fold Change H3K56ac
DMSO
CI-994 10 μM
1 (JPS004) 10 μM
8
9
24
23
0.1 μM
1 μM
10 μM

1.2. Representative Blots from Dose Response Experiment with **7**,**9**,**21**,**22**

Figure S3. Western blotting of HDAC1/2/3 proteins in HCT116 cells treated with compounds 7,9,21,22 at concentration range from 0.01-10 µM. Blots of two independent biological replicates.

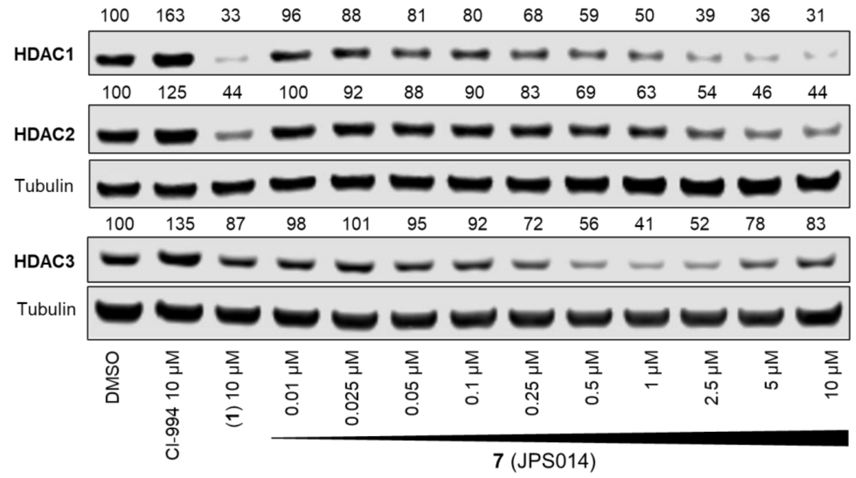

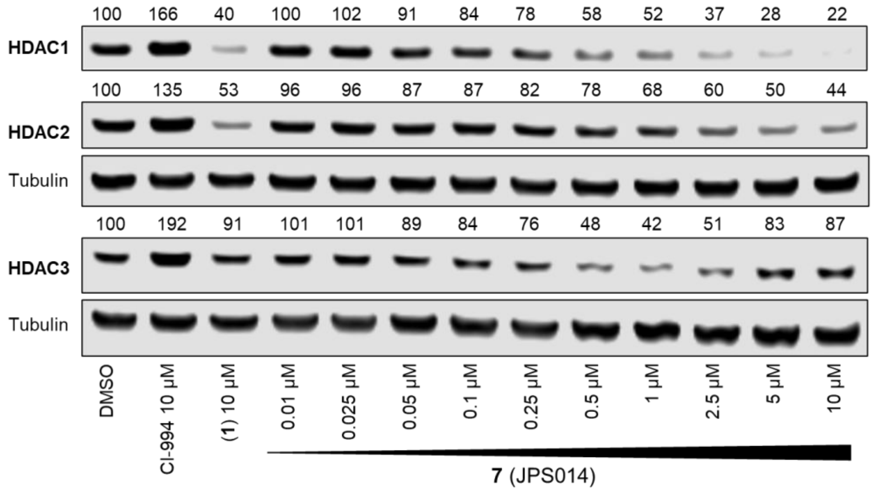

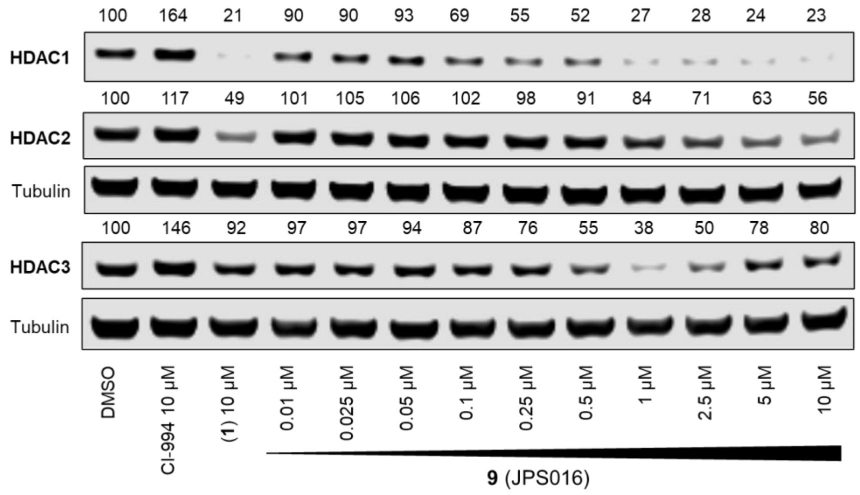

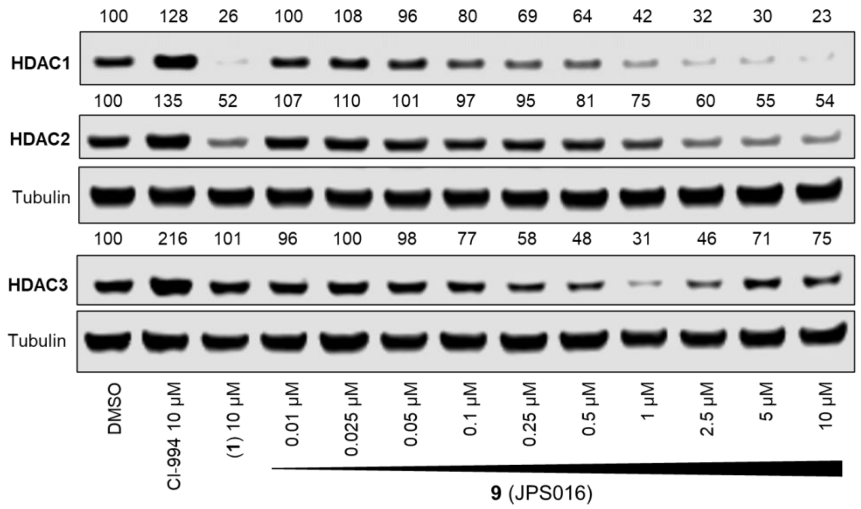
100 128 26 100 108 96 80 69 64 42 32 30 23
HDAC1
100 135 52 107 110 101 97 95 81 75 60 55 54
HDAC2
Tubulin
100 216 101 96 100 98 77 58 48 31 46 71 75
HDAC3
Tubulin
DMSO
CI-994 10 μM
(1) 10 μM
0.01 μM
0.025 μM
0.05 μM
0.1 μM
0.25 μM
0.5 μM
1 μM
2.5 μM
5 μM
10 μM
9 (JPS016)

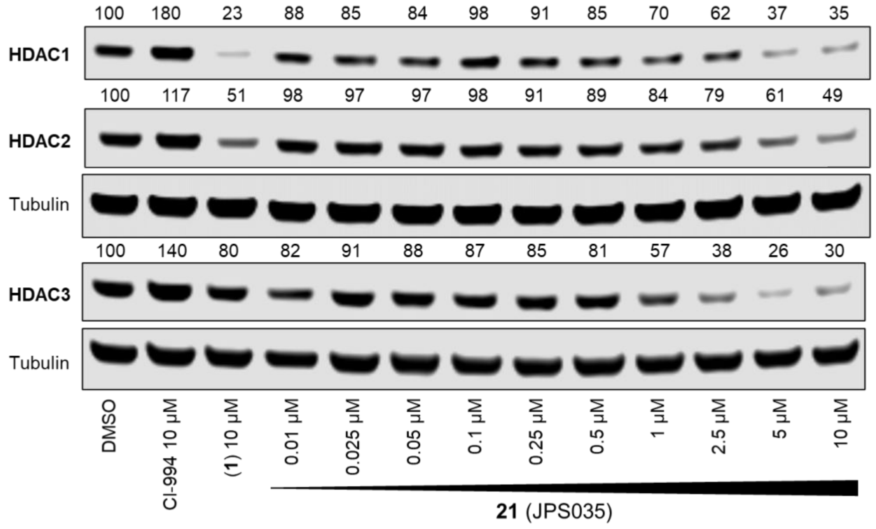
100 180 23 88 85 84 98 91 85 70 62 37 35
HDAC1
100 117 51 98 97 97 98 91 89 84 79 61 49
HDAC2
Tubulin
100 140 80 82 91 88 87 85 81 57 38 26 30
HDAC3
Tubulin
DMSO
CI-994 10 μM
(1) 10 μM
0.01 μM
0.025 μM
0.05 μM
0.1 μM
0.25 μM
0.5 μM
1 μM
2.5 μM
5 μM
10 μM
21 (JPS035)

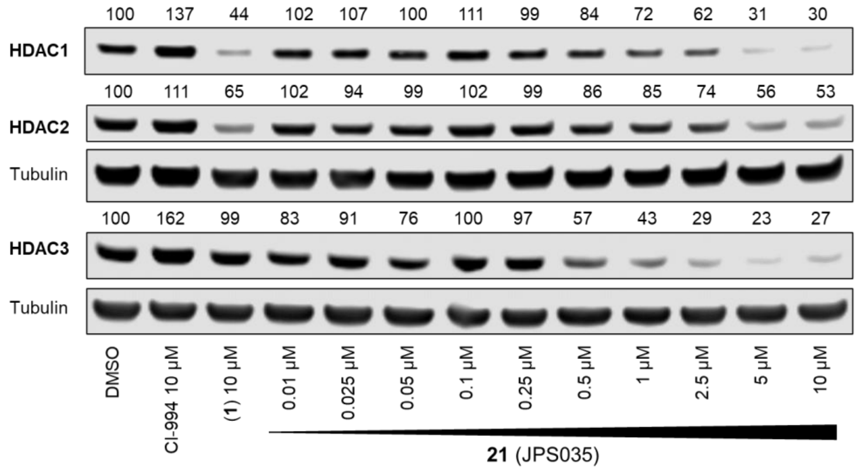
100 137 44 102 107 100 111 99 84 72 62 31 30
HDAC1
100 111 65 102 94 99 102 99 86 85 74 56 53
HDAC2
Tubulin
100 162 99 83 91 76 100 97 57 43 29 23 27
HDAC3
Tubulin
DMSO
CI-994 10 μM
(1) 10 μM
0.01 μM
0.025 μM
0.05 μM
0.1 μM
0.25 μM
0.5 μM
1 μM
2.5 μM
5 μM
10 μM
21 (JPS035)

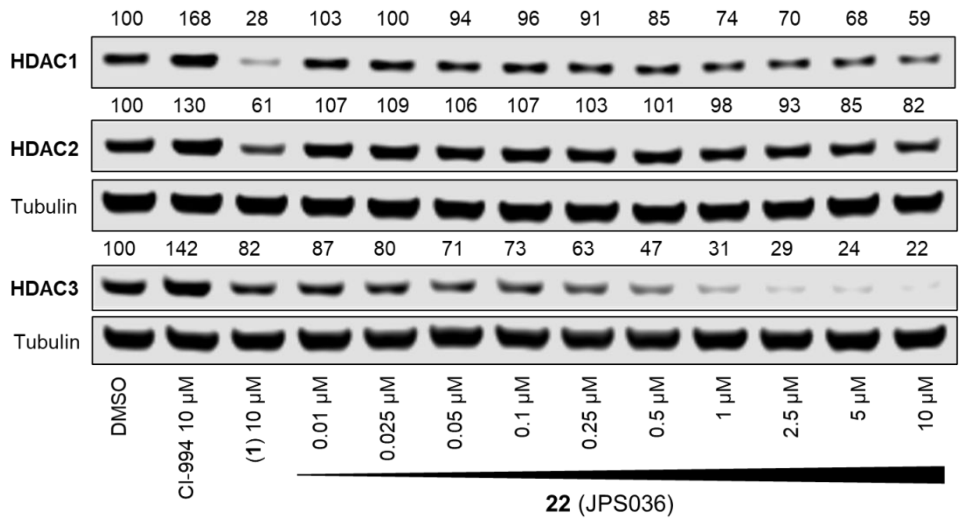
100 168 28 103 100 94 96 91 85 74 70 68 59
HDAC1
100 130 61 107 109 106 107 103 101 98 93 85 82
HDAC2
Tubulin
100 142 82 87 80 71 73 63 47 31 29 24 22
HDAC3
Tubulin
DMSO
CI-994 10 µM
(1) 10 µM
0.01 µM
0.025 µM
0.05 µM
0.1 µM
0.25 µM
0.5 µM
1 µM
2.5 µM
5 µM
10 µM
22 (JPS036)

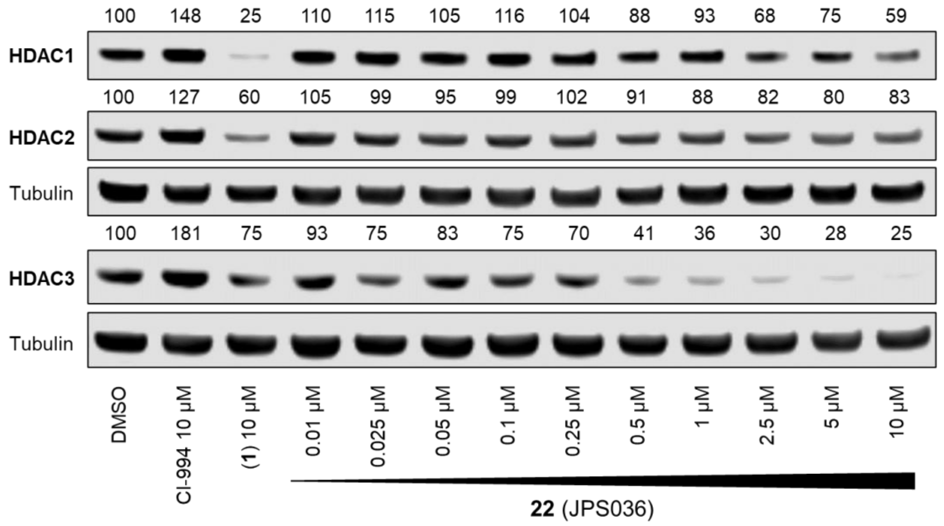
100 148 25 110 115 105 116 104 88 93 68 75 59
HDAC1
100 127 60 105 99 95 99 102 91 88 82 80 83
HDAC2
Tubulin
100 181 75 93 75 83 75 70 41 36 30 28 25
HDAC3
Tubulin
DMSO
CI-994 10 µM
(1) 10 µM
0.01 µM
0.025 µM
0.05 µM
0.1 µM
0.25 µM
0.5 µM
1 µM
2.5 µM
5 µM
10 µM
22 (JPS036)

1.3. Western Blotting Analysis from Further Experiments with **7** and **9**

Figure S4. Representative Western blot of HDAC1, 2 & 3 degradation levels over 2, 4, 8, 15, 24, 26 and 48 hours with 9 (JPS016) at 1 µM. Representative blots of two independent biological replicates.

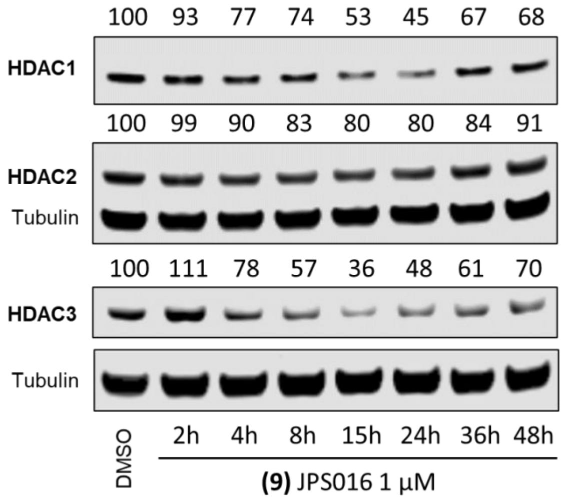

Figure S5. Representative Western blot of HDAC1, 2,3 and H3K56ac levels with 9 (JPS016), it's equivalent negative control 25 (JPS016NC) which possesses the inactive VHL diastereoisomer, along with proteosome inhibitor MG 132 and the VH032 VHL ligand. Representative blots of two independent biological replicates.

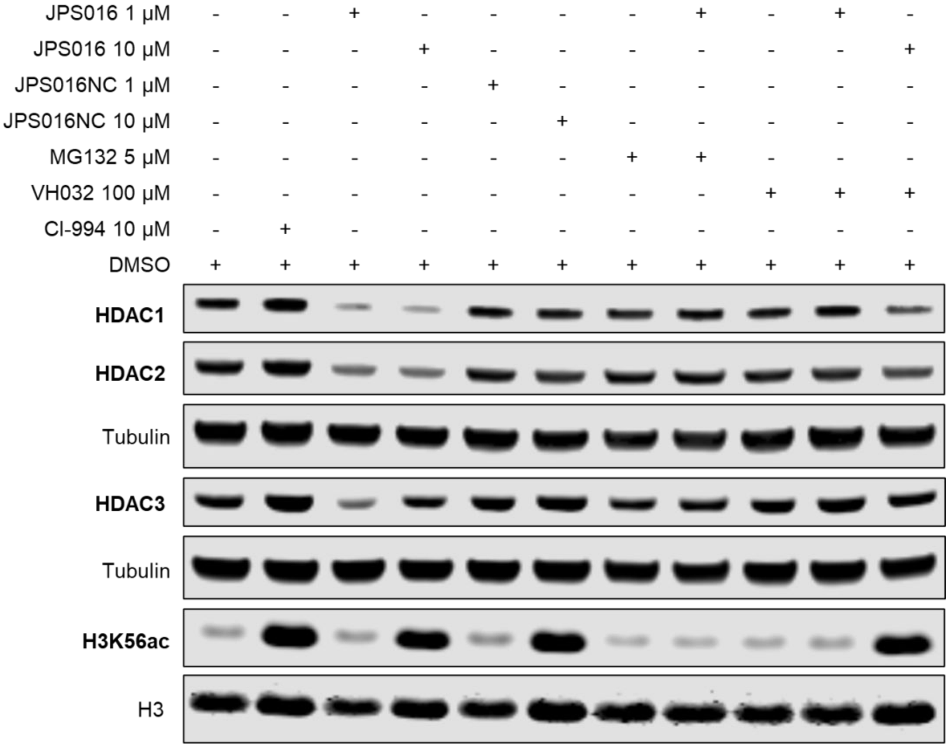

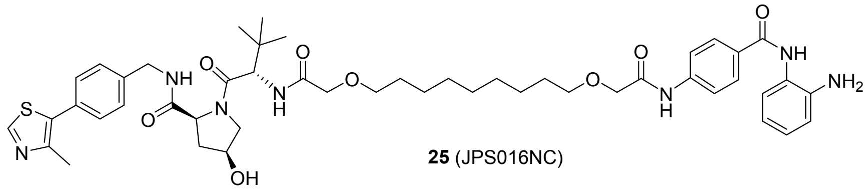

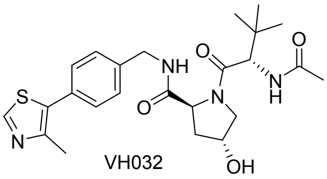

Figure S6. Representative Western blot of HDAC1/2/3 levels after 24 hours and 48 hours with 1 (JPS004), 7 (JPS014) and 9 (JPS016), and bar graph representation of HDAC1-3, LSD1 and Sin3a protein levels after 24 hours and 48 hours as the average of three independent biological replicates.

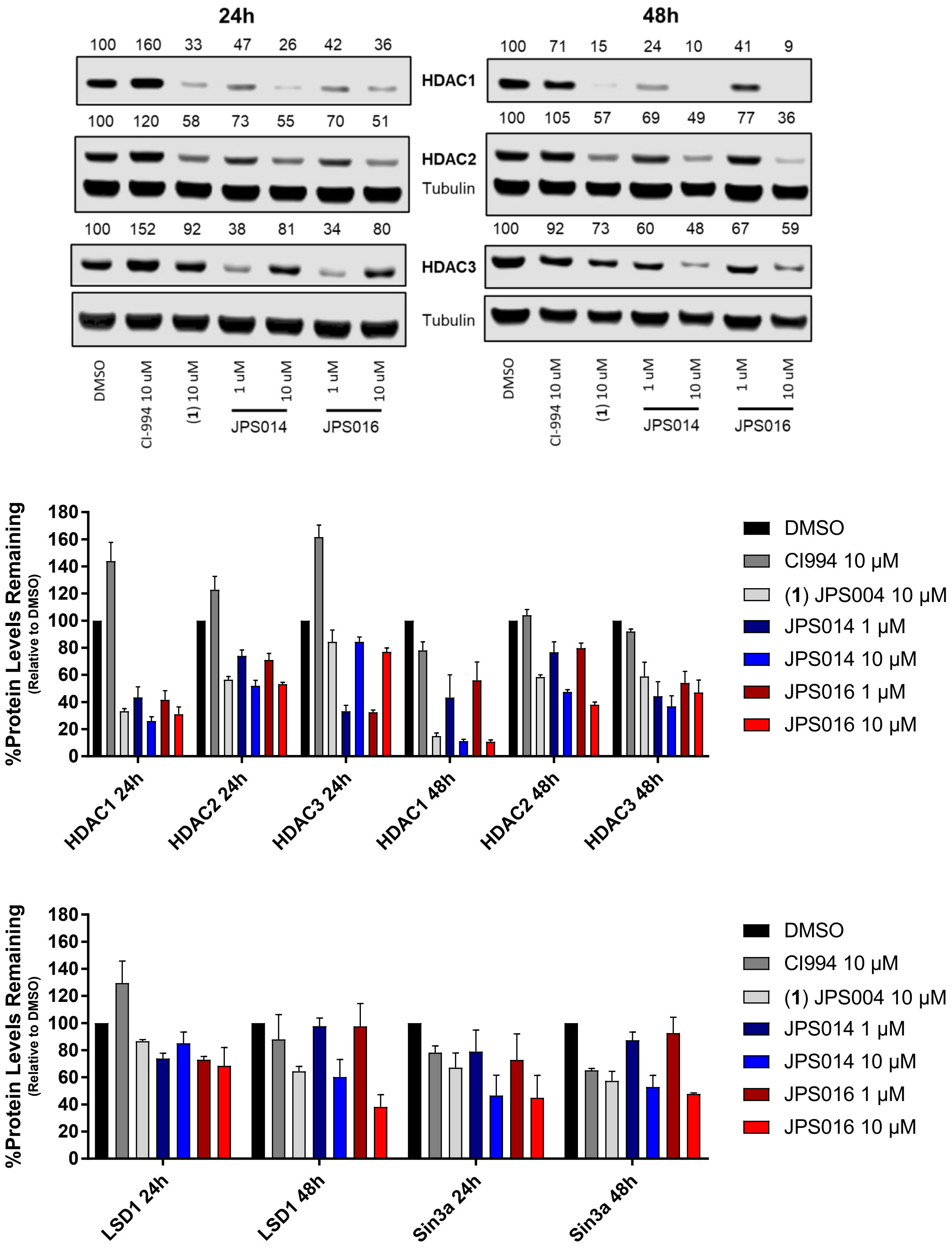

Figure S7. Statistical Analysis of PROTACs JPS014, JPS016, JPS035 and JPS036 vs DMSO control. The quantified data represent mean ± SEM (n = 3, *P<0.1, **P<0.01, ***P<0.001).

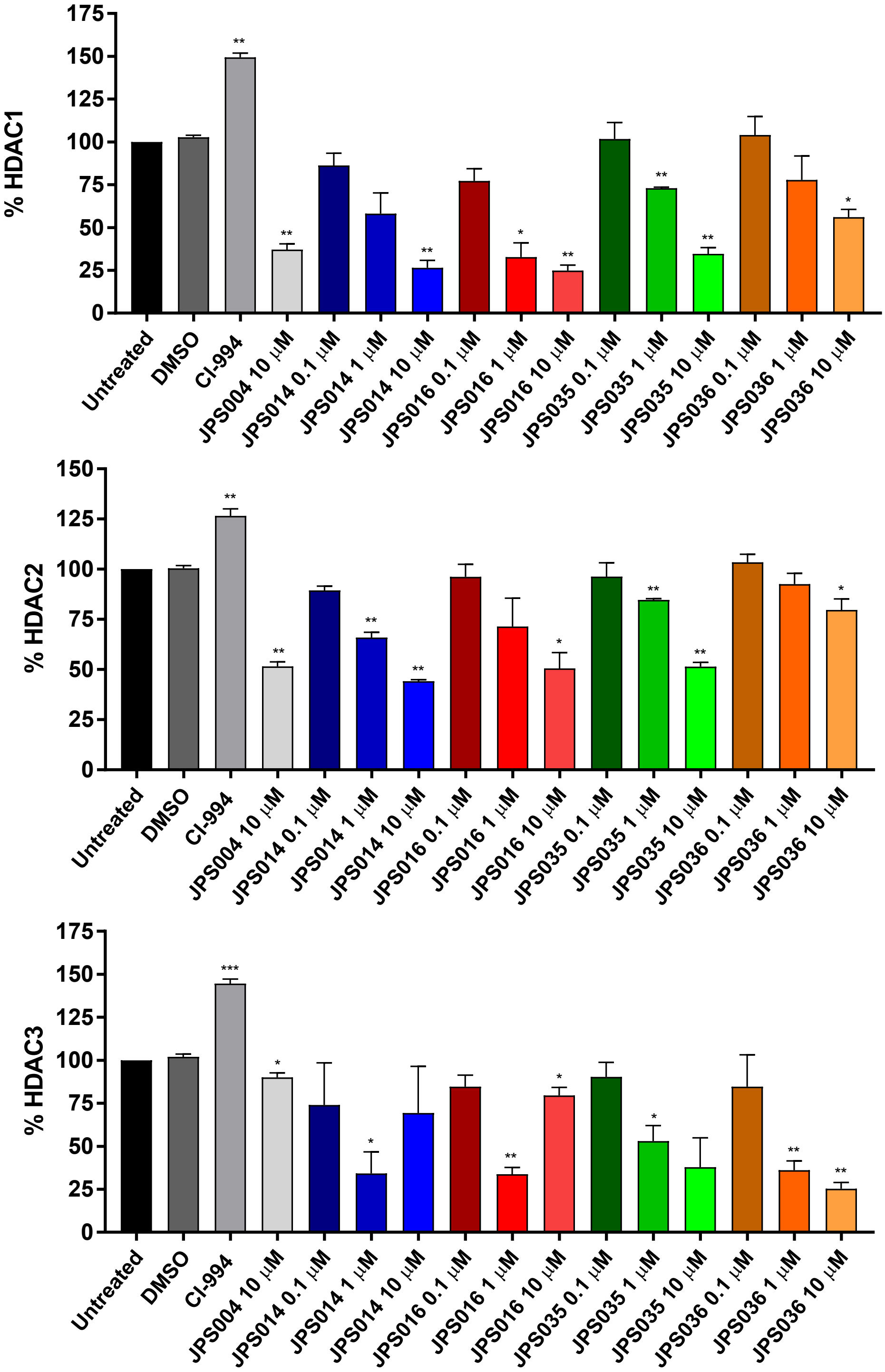

3. Properties Table of Compounds **1-24**

Table S1. Physiochemical properties[a] table for compounds 1-24, complete with average maximum HDAC1, HDAC2 and HDAC3 degradation levels[b].

Compound	Mwt	cLogP	TPSA (Å^2)	H-bond donors	H-bond acceptors	Avg. Max HDAC1 degradation	Avg. Max HDAC2 degradation	Avg. Max HDAC3 degradation
**1** (JPS04)	852.1	5.86	224.09	6	7	62%	50%	18%
**2**	810.02	4.89	224.09	6	7	41%	32%	3%
**3**	824.04	5.31	224.09	6	7	54%	37%	23%
**4**	838.07	5.7	224.09	6	7	67%	43%	33%
**5**	880.15	6.35	224.09	6	7	63%	44%	44%
**6**	854.07	5.12	233.32	6	8	60%	57%	67%
**7** (JPS014)	854.07	5.03	233.32	6	8	76%	56%	69%
**8**	856.04	4.14	242.55	6	9	57%	47%	39%
**9** (JPS016)	898.12	5.21	242.55	6	9	74%	52%	68%
**10**	799.93	2.91	242.55	6	9	13%	6%	24%
**11**	843.99	2.95	251.78	6	10	29%	14%	44%
**12**	888.04	3.08	261.01	6	11	6%	5%	22%
**13**	880.11	3.66	230.57	6	9	4%	5%	0%
**14**	828.01	5.33	224.09	6	8	41%	13%	39%
**15**	870.09	6.13	224.09	6	8	61%	26%	51%
**16**	861.98	3.32	251.78	6	11	11%	0%	37%
**17**	916.11	5.56	242.55	6	10	58%	23%	63%
**18**	892.14	6.26	252.33	6	7	6%	6%	3%
**19**	934.22	7.09	252.33	6	7	26%	15%	10%
**20**	926.11	4.36	280.02	6	10	22%	15%	23%
**21** (JPS035)	896.15	6.18	233.32	6	8	64%	50%	57%
**22** (JPS036)	940.18	6.71	233.32	6	9	45%	23%	75%
**23**	897.09	4.17	262.42	7	9	53%	14%	27%
**24**	939.17	5.28	262.42	7	9	38%	11%	15%

[a]Physiochemical property predictions of 1-23 were calculated using SwissADME (swissadme.ch). The cLogP values represent the average of five LogP predictions from the programme.

[b]Maximum degradation values for HDAC1, HDAC2 and HDAC3 were calculated from Figure S1. The average maximum degradation of HDAC1, HDAC2 and HDAC3 was used for the maximum degradation of 1 (JPS004). Entries highlighted in pale green exhibited ≥ 50% maximal degradation of either HDAC1, HDAC2, or HDAC3, whilst entries in dark green exhibited ≥ 70% maximal degradation of either HDAC1, HDAC2, or HDAC3.

Histone Deacetylase Assay. Inhibition experiments against HDAC1-CoREST-LSD1, HDAC2-CoREST-LSD1 and HDAC3-SMRT-DAD were conducted using a fluorescent HDAC assay. The assays were carried out utilising a black 96-well plates (Corning) with a reaction volume of 50 µL. HDAC complexes were expressed and purified as described previously.[1,2] 100 µM of Boc- (Ac)Lys-AMC was used as a substrate in each well. All determinations were performed in triplicate. The inhibitors/PROTACs were dissolved at 50 mM in DMSO, and then further diluted with 10% DMSO HDAC assay buffer (10% DMSO, 50 mM Tris pH 7.5, 50 mM NaCl, 0.1 mg/mL BSA) to micromolar concentrations. 1:3 serial dilutions were then carried out using 10% DMSO HDAC assay buffer to afford range of concentrations. 10 µL of each of these solutions were added to individual wells, followed by addition of 30 µL of the HDAC complex (12.5 nM) dissolved in HDAC assay buffer (50 mM Tris pH 7.5, 50 mM NaCl, 0.1 mg/mL BSA). The plate was then incubated at 20 ˚C, 100 RPM for 1 hour. After incubation, 10 µL of the Boc-(Ac)Lys-AMC substrate, dissolved in HDAC assay buffer, was added to each well. The plate was incubated at 30 ˚C, 100 RPM for 1 hour, followed by addition of 50 µL of a developer buffer (50 mM Tris pH 7.5, 100 mM NaCl, 10 mg/mL trypsin) to quench the reaction. The reaction was allowed to develop for 10 minutes at 30˚C, 100 RPM. Fluorescence intensity was determined with the Victor X5 plate reader (Perkin Elmer, λex = 335 nm, λem = 460 nm). IC_{50} values were calculated through the GraphPad Prism 7 software by non-linear regression, log (inhibitor) vs. response – variable slope (four parameters).

Figure S8. HDAC Assay Results of CI-994, JPS014, JPS016, JPS035 and JPS036 with the HDAC1-CoREST-LSD1, HDAC2-CoREST-LSD1 and HDAC3-SMRT complexes.

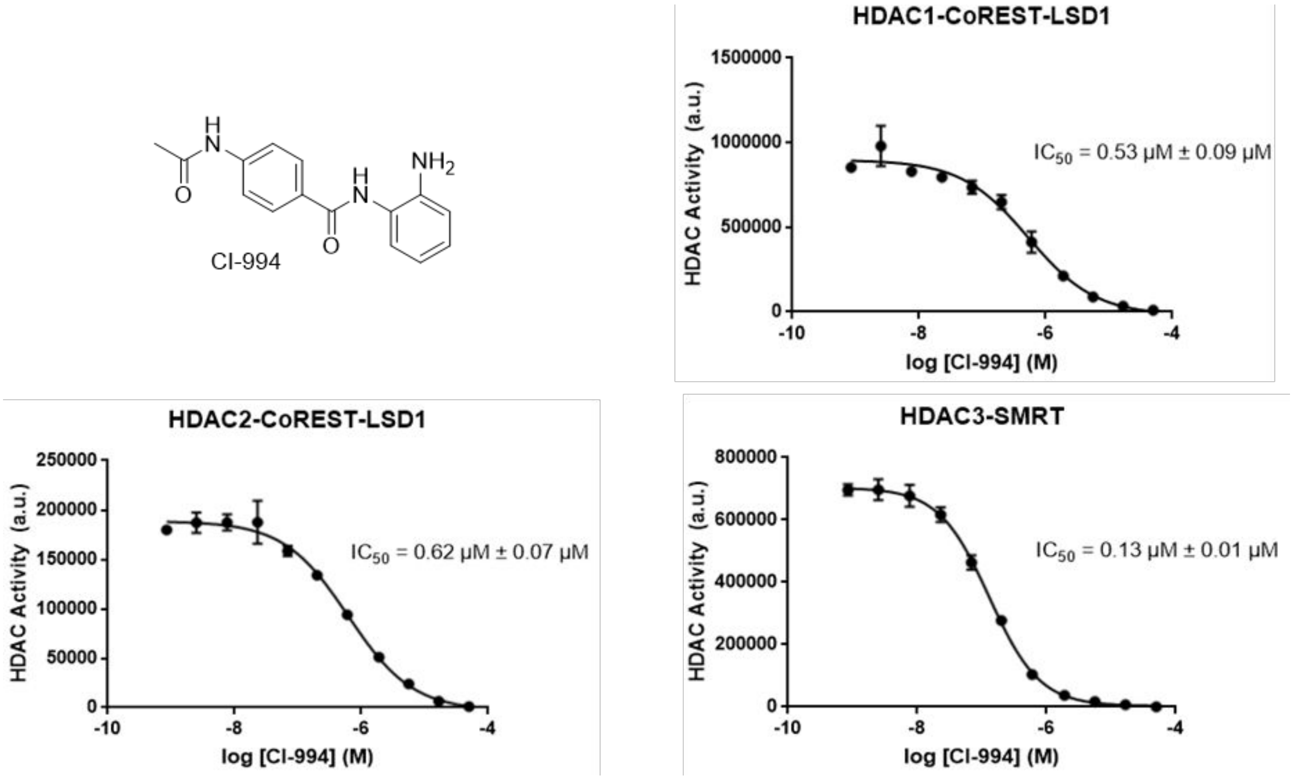

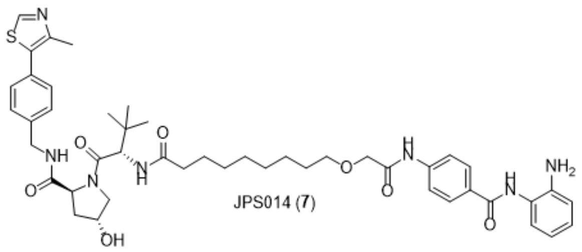
JPS014 (7)

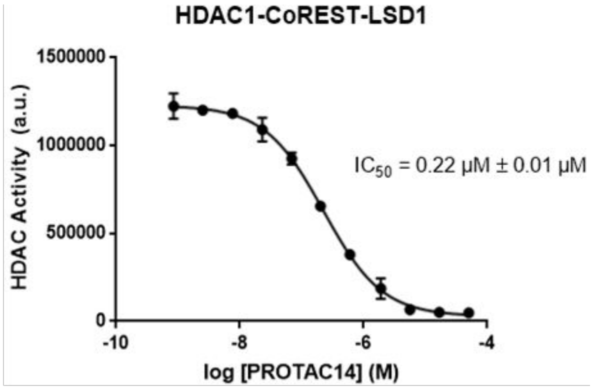
HDAC1-CoREST-LSD1
HDAC Activity (a.u.)
log [PROTAC14] (M)
IC50 = 0.22 μM ± 0.01 μM

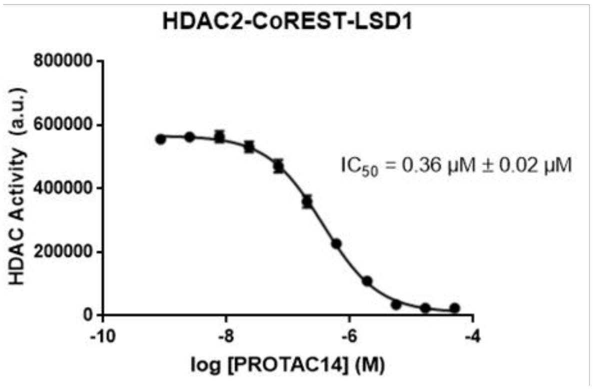
HDAC2-CoREST-LSD1
HDAC Activity (a.u.)
log [PROTAC14] (M)
IC50 = 0.36 μM ± 0.02 μM

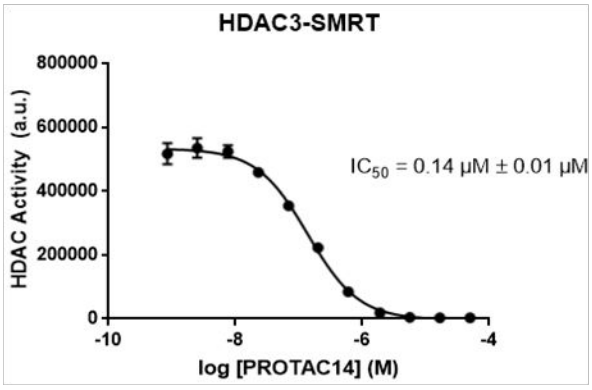
HDAC3-SMRT
HDAC Activity (a.u.)
log [PROTAC14] (M)
IC50 = 0.14 μM ± 0.01 μM

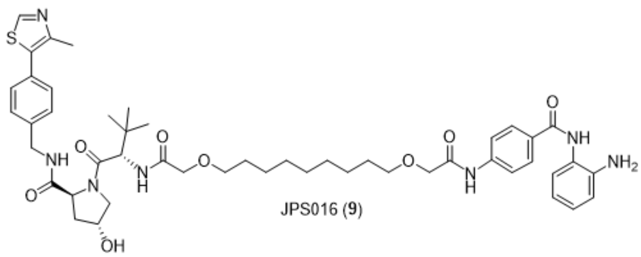
JPS016 (9)

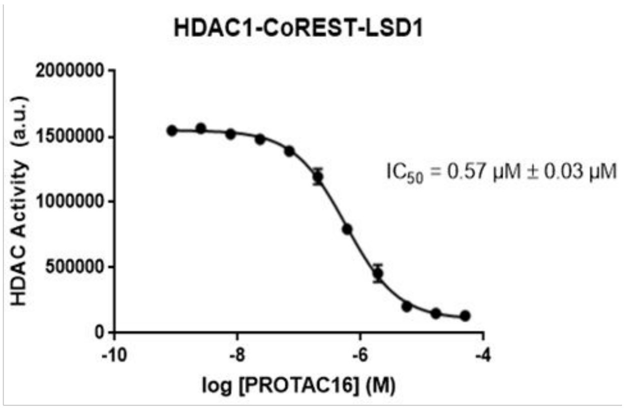
HDAC1-CoREST-LSD1
HDAC Activity (a.u.)
log [PROTAC16] (M)
IC50 = 0.57 μM ± 0.03 μM

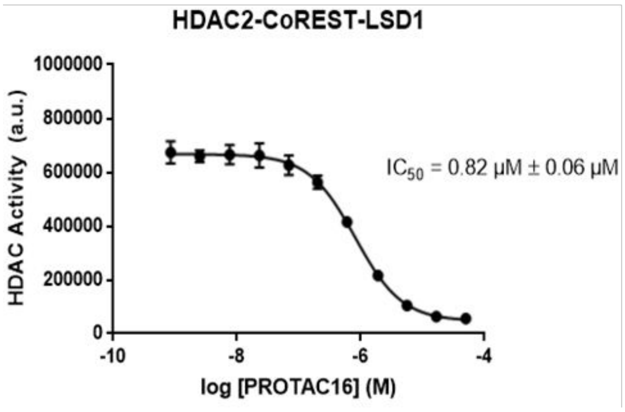
HDAC2-CoREST-LSD1
HDAC Activity (a.u.)
log [PROTAC16] (M)
IC50 = 0.82 μM ± 0.06 μM

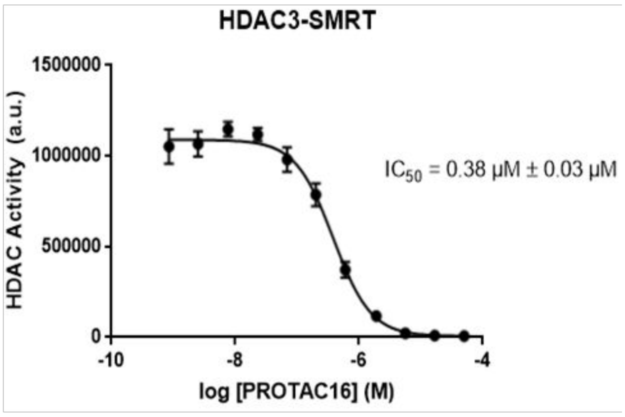
HDAC3-SMRT
HDAC Activity (a.u.)
log [PROTAC16] (M)
IC50 = 0.38 μM ± 0.03 μM

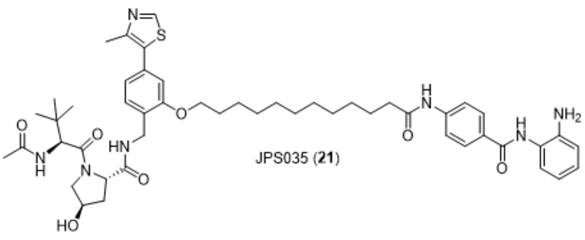

JPS035 (**21**)

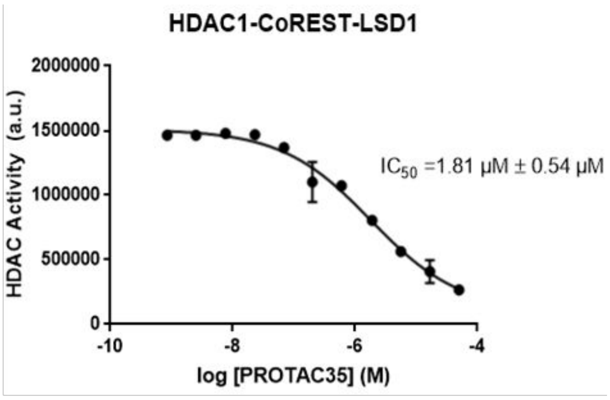

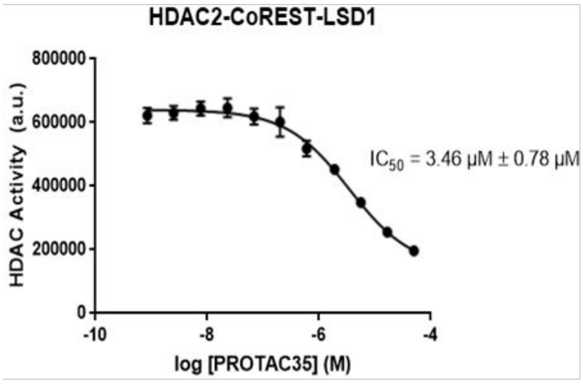

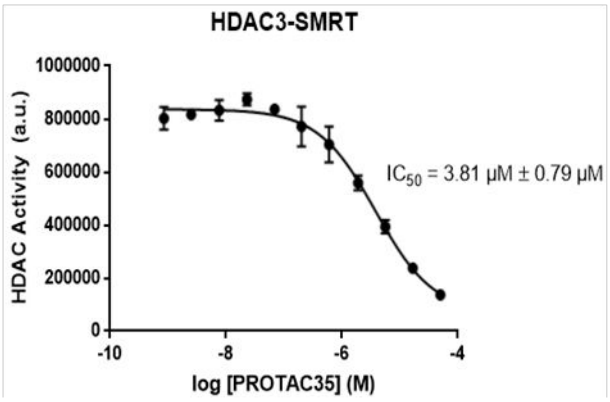

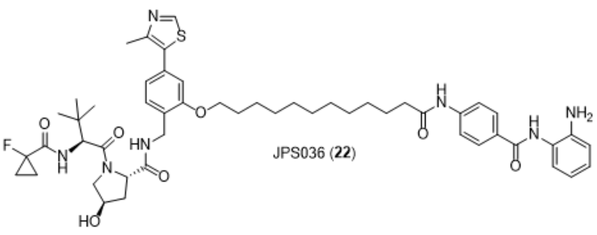

JPS036 (**22**)

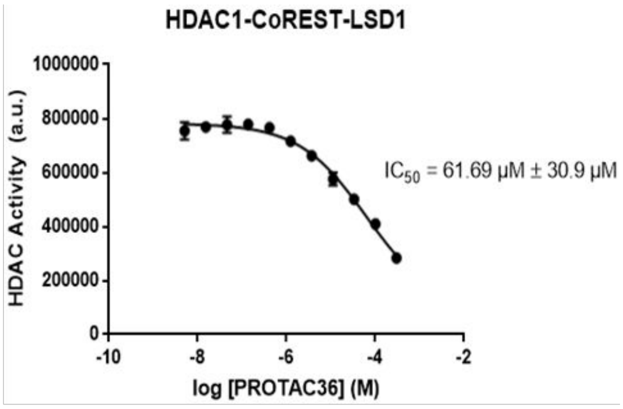

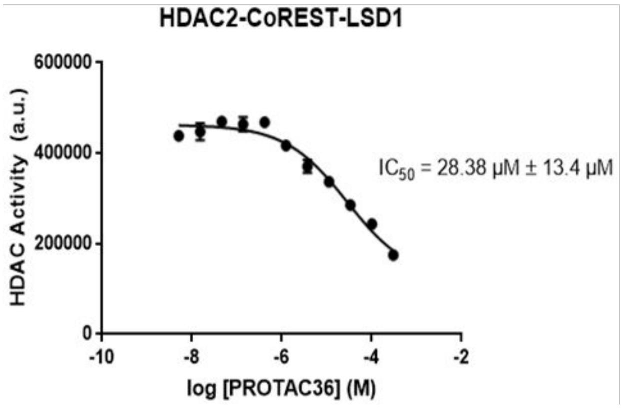

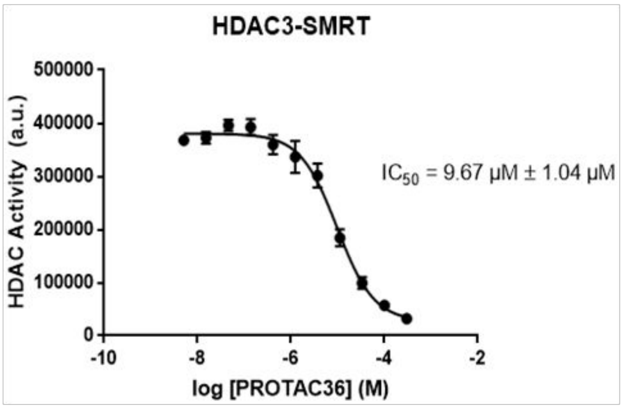

3. Apoptosis Flow Cytometry Assay

Figure S9. Apoptosis flow cytometry assay with 2.5 µM and 10 µM treatments of JPS016 and JPS016NC at 24 and 48 hours. Error bars represent the average of three independent biological replicates.

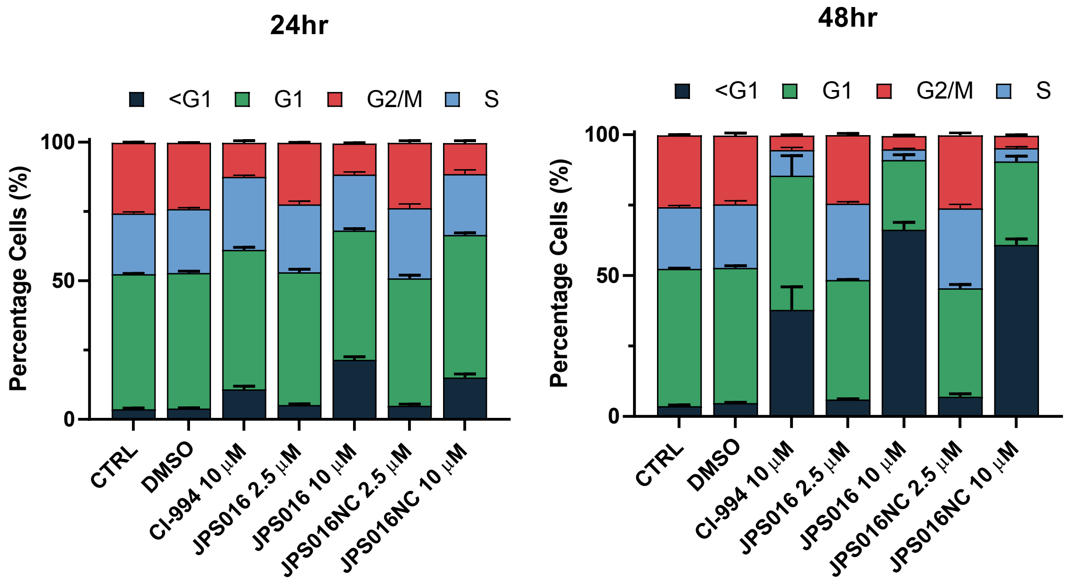

4. RNA Seq – Bioinformatics Analysis

Paired-end reads were aligned using HISAT2 (v2.2.1) and mapped to the HISAT2 GRCh38_tran index build.[3] Quality control was performed on raw and aligned data using FASTQC (v0.11.9) and summarised using MultiQC (v1.11).[4,5] The resulting SAM files were subsequently sorted and converted to the BAM format before indexing with SAMtools (v 1.12).[6] Reads were then counted across the exons of each gene and gene loci annotated using command-line program LiBiNorm (v2.4); run in HT-Seq compatibility mode (-z) and provided with the 'Homosapiens.GRCh38.104' GTF file obtained from Ensembl. LiBiNorm was run using the following parameters: *LiBiNorm count -z -r pos -i gene_name -s reverse <BAM file> Homo_sapiens.GRCh38.104.gtf <OUTPUT file>*.[7] Default normalisation of expression values and differential gene analysis was then carried out using the R package DESeq2.[8] Significance thresholds were set as a p-adjusted value < 0.01 and a fold change > 2 (log_2 fold change > 1) and implemented for down-stream analysis, with all plots produced in R. Gene ontology (GO) analysis was carried out using the Bioconductor package topGO using the human genome-wide annotation package *org.Hs.eg.db* to map gene identifiers to the relevant GO term.[9,10]

The raw data and processed count files from this study can be obtained from the Gene Expression Omnibus (GEO) database (https://www.ncbi.nlm.nih.gov/geo/) under the accession number GSE197985.

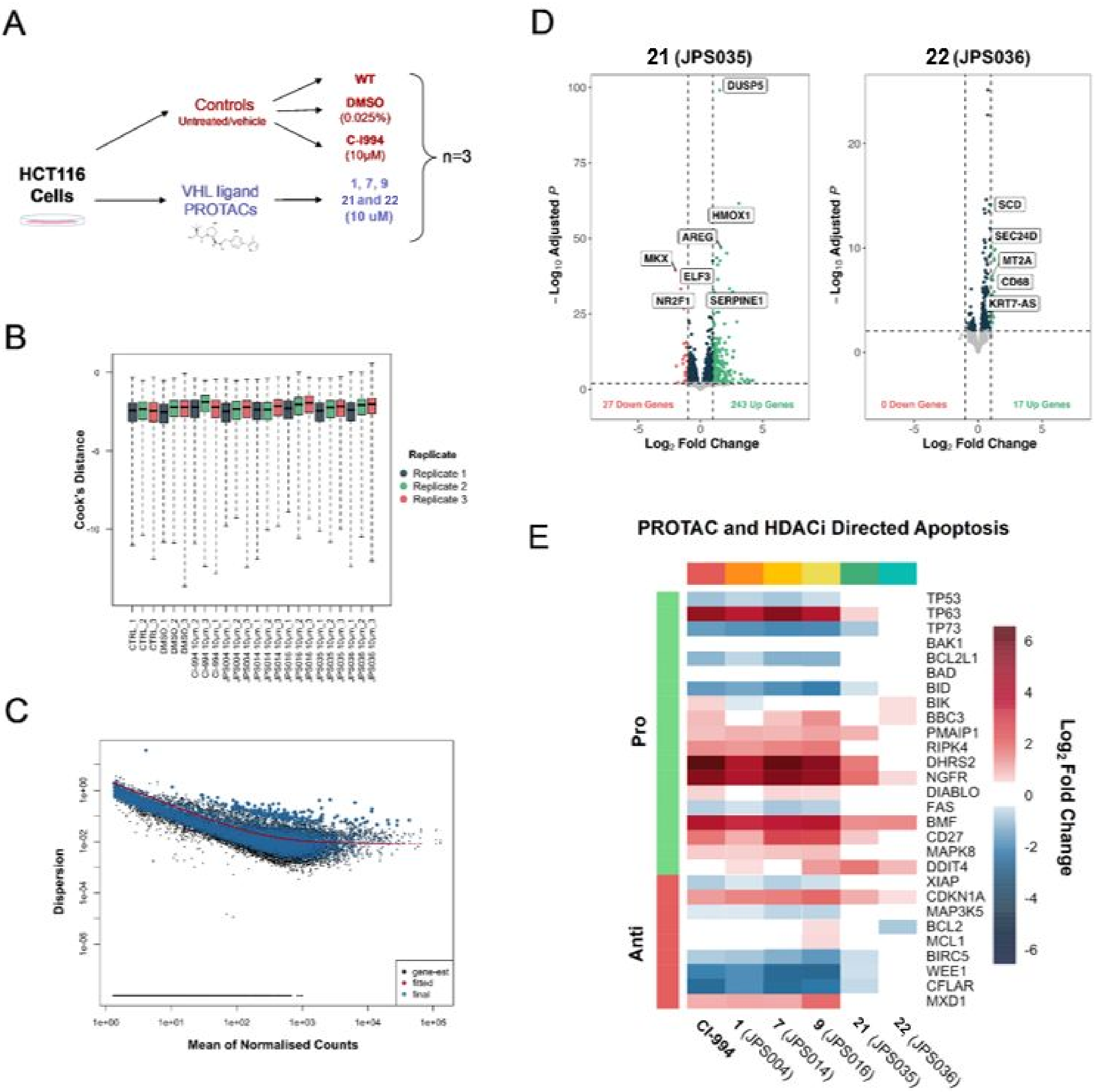

Figure S10. RNA-sequencing of HCT116 cells treated with Class I HDAC targeting PROTACs. (A) Schematic depiction of PROTAC treatments of HCT116 cells, where cells were treated with C-I994, JPS004, JPS014, JPS016, JPS035, and JPS036 at 10 µM with relevant controls for 24-hours. The experiment was performed with three independent biological replicates for each PROTAC or control. (B) RNA-sequence quality control. Cook's distance statistical test for outliers calculated by DESeq2 reveals no outlying samples in the analysis. (C) DESeq2 dispersion estimates show a decrease in dispersion as variance/counts increase as expected for data modelled with DESeq2. (D) RNA-sequencing of class I HDAC targeting PROTACs incorporating modifications to the VHL E3 ligase ligand. Volcano plots displaying differential gene expression (DEG) profiles of JPS035 and JPS036. (E) Influence of PROTAC mediated Class I HDAC degradation on the HCT116 transcriptome determined by RNA-sequencing. Heatmap of apoptotic genes involved in the initiation of apoptosis with DHRS2 displaying the largest change in differential gene expression. Heatmaps are presented as the log2 fold change of the mean normalised counts for each sample relative to the WT control sample.

Supporting Information: Chemistry

5. Materials and Methods

General Methods

General Method A. DIPEA (1.5 equiv.) was added to a solution of substituted aniline (1 equiv.) in dry DCM (2.5 mL/mmol) at 0 °C, followed by the dropwise addition of 4-nitrobenzoyl chloride (1.1 equiv.) as a solution in dry DCM. The mixture was stirred at 0 °C for 30 minutes, then at room temperature overnight. The reaction mixture was diluted with DCM and then washed with sat. $NaHCO_4$, 1M HCl and sat. NaCl. The organic layer was then dried over Na_2SO_4, concentrated *in vacuo,* then purified accordingly to afford the desired compound.

General Method B. Triethylamine (3 equiv.) was added to a solution of 4-aminobenzamide starting material (1 equiv.) in dry THF (10 mL/mmol) at 0 °C, followed by the dropwise addition of acetyl chloride (1.2 equiv.). The mixture was stirred at 0 °C for 30 minutes, then at room temperature for 2 hours. The reaction mixture was concentrated *in vacuo* to give the corresponding crude, which was chromatographically purified to afford the desired compound.

General Method C. TFA (20 equiv.) was added to a stirring solution of Boc-protected HDAC inhibitor/inhibitor-linker (1 equiv.) in DCM (10 mL/mmol) and the resulting reaction mixture stirred at room temperature for 6 hours. The reaction mixture was concentrated *in vacuo*, dissolved in MeOH (0.1 M), agitated in MP-carbonate resin (3.02 mmol/g loading capacity) for 2-3 hours and then filtered. The filtrate was concentrated *in vacuo* to afford the desired HDAC inhibitor/inhibitor-linker.

General Method D. To a solution of dicarboxylic acid (1 equiv.) in 1,4-dioxane/DMF (1:1, 4 mL/mmol), was added benzyl bromide (1 equiv.), followed by the addition of $NaHCO_3$ (1 equiv.). The resulting suspension was heated at 90 °C overnight. The reaction mixture was left to cool to room temperature and then concentrated *in vacuo*. The crude residue was then suspended in EtOAc and washed with sat. NaCl and water. The organic phase was dried over $MgSO_4$, filtered and concentrated *in vacuo* to afford the corresponding crude, which was chromatographically purified to afford the desired compound.

General Method E. To a solution of carboxylic acid linker intermediate (1.1-1.3 equiv.) in dry DMF (10 mL/mmol) at 0 °C, DIPEA (3 equiv.) and HATU (1.3-1.5 equiv.) were added. The reaction mixture was stirred for 15 minutes, after which a solution of amine (1 equiv.) in DMF was added slowly and the resultant solution stirred at room temperature overnight. The reaction mixture was diluted in EtOAc, then washed with sat. $NaHCO_3$ and sat. NaCl. The organic layer was dried over $MgSO_4$, filtered and concentrated *in vacuo* to give the corresponding crude, which was chromatographically purified to afford the desired compound.

General Method F. To a solution of the benzyl ester protected HDACi-linker conjugate (1 equiv.) in THF, Pd/C (10% wt) was added. The reaction flask was filled with nitrogen and evacuated 3 times using a Shlenk line, before a balloon of hydrogen was added and the resultant mixture stirred vigorously for 4-18 hours. The balloon of hydrogen was removed and the flask was flushed with nitrogen. The reaction mixture was filtered through a glass microfiber filter paper, and the filtrate concentrated *in vacuo* to afford the desired compound.

General Method G. To a solution of HDACi-linker acid (1.2 equiv.) in dry DMF (1 mL) at 0 °C, DIPEA (3 equiv.) and HATU (1.3 equiv.) were added. The reaction mixture was stirred for 15 minutes, after which a solution of (4R)-3-Methyl-L-valyl-4-hydroxy-N-[[4-(4-methyl-5-thiazolyl)phenyl]methyl]-L-prolinamide hydrochloride (**VH_032 amine**, 0.08-0.10 mmol) in DMF (1 mL) was added slowly and the resultant solution stirred at room temperature for 16 hours. The reaction mixture was diluted in EtOAc (10 mL), then washed with sat. $NaHCO_3$ (2 x 5 mL) and sat. NaCl (2 x 5 mL). The organic layer was dried over $MgSO_4$, filtered, and concentrated *in vacuo* to give the corresponding crude, which was chromatographically purified to afford the desired compound.

General Method H. TFA (0.4 mL or 20 equiv.) was added to a stirring solution of Boc-protected PROTAC (1 equiv.) in DCM (2 mL) and the resulting reaction mixture stirred at room temperature for 4-6 hours. The reaction mixture was concentrated *in vacuo*, dissolved in MeOH (2 mL), agitated in MP-carbonate resin (3.02 mmol/g loading capacity) for 2-3 hours and then filtered. The filtrate was concentrated *in vacuo* and the resulting solid dissolved in MeCN:H_2O (1:1) and lyophilised to remove residual TFA impurities, affording the final PROTAC.

General Method I. A mixture of E3 ligand phenol (1 equiv.), alkyl bromide (1 equiv.) and K_2CO_3 (3 equiv.) in dry DMF (0.8 mL) was stirred at 70 °C overnight. The reaction mixture was concentrated *in vacuo* to give the corresponding crude, which was chromatographically purified to afford the desired compound.

General Method J. To a solution of HDACi-linker acid (1.2 equiv.) in dry DMF (1 mL) at 0 °C, DIPEA (3 equiv.) and HATU (1.4 equiv.) were added. The reaction mixture was stirred for 15 minutes, after which a solution of (2S,4R)-1-((S)-2-Acetamido-3,3-dimethylbutanoyl)-N-(2-(4-aminobutoxy)-4-(4-methylthiazol-5-yl)benzyl)-4-hydroxypyrrolidine-2-carboxamide dihydrochloride (**VH032 phenol-alkylC4-amine**, 0.035 mmol) in DMF (1 mL) was added slowly and the resultant solution stirred at room temperature for 16 hours. The reaction mixture was diluted in EtOAc (10 mL), then washed with sat. $NaHCO_3$ (2 x 5 mL) and sat. NaCl (2 x 5 mL). The organic layer was dried over $MgSO_4$, filtered, and concentrated *in vacuo* to give the corresponding crude, which was chromatographically purified to afford the desired compound.

Scheme S1. Synthesis of HDAC inhibitor intermediates 35a-c.

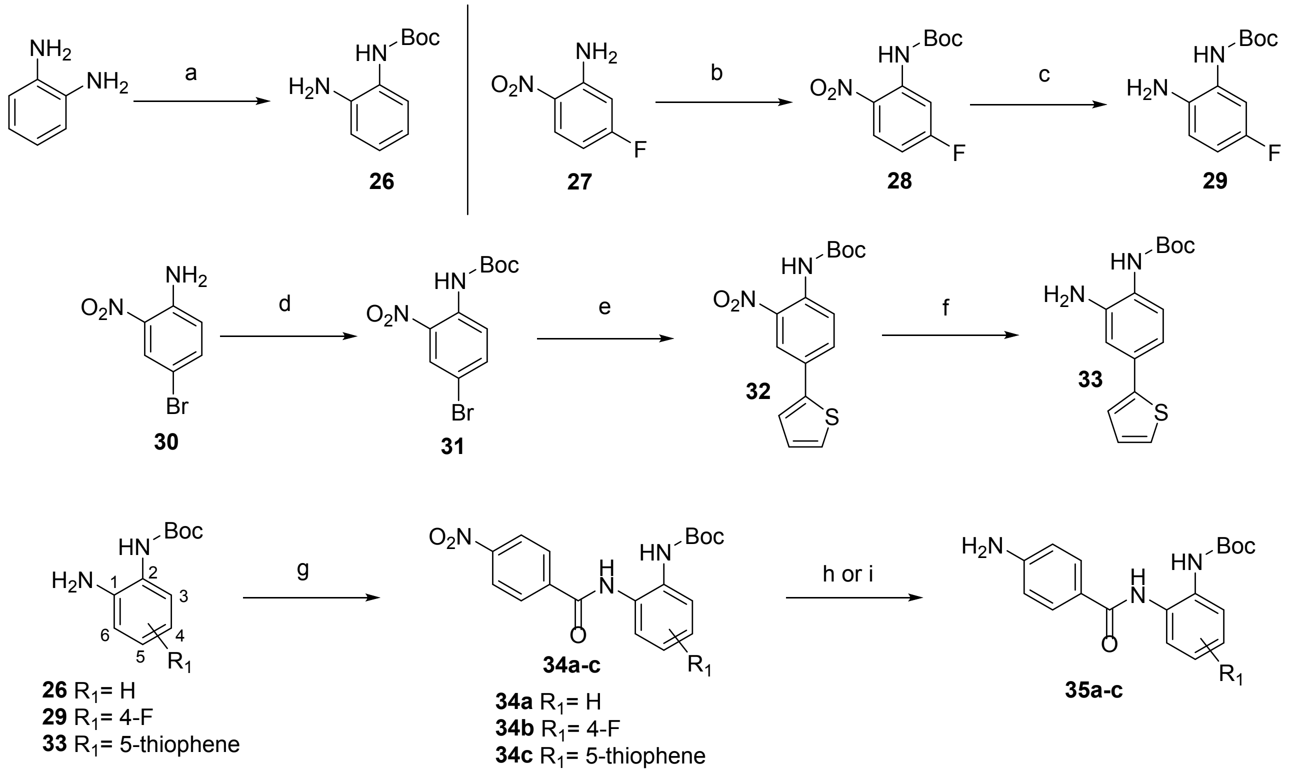

Reagents and conditions: (a) Di-tert-butyl decarbonate (Boc_2O), NEt_3,THF, 0 ºC for 3 h then r.t. overnight; (b) Boc_2O, NEt_3, 4-dimethylaminopyridine (DMAP), 80 ºC, overnight; (c) H_2, Pd/C, MeOH, r.t., 4 h; (d) Boc_2O, NEt_3, 4-dimethylaminopyridine (DMAP), 60 ºC, overnight; (e) thiophen-2-ylboronic acid, $Pd(PPh_3)_4$, Na_2CO_3, dimethoxyethane (DME):water = 2:1, 110 ºC, overnight; (f) H_2, Pd/C, MeOH, r.t., 4 h; (g) 4-nitrobenzoyl chloride, DIPEA, THF, 0 ºC - r.t., overnight; (h) H_2, Pd/C, MeOH, r.t., 7-16 h; (i) $SnCl_2$, MeOH : DCM = 1:1, r.t., 1 wk.

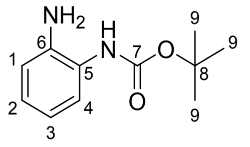

Tert-butyl (2-aminophenyl)carbamate (26): A solution of Boc_2O (6.05 g, 27.7 mmol) in THF (50 mL) was added dropwise over 3 hours to a solution of *o*-phenylenediamine (3.00 g, 27.7 mmol) and triethylamine (4.64 mL, 33.3 mmol) in THF (25 mL) at 0 °C, then the mixture was stirred at room temperature for 15 hours. The reaction was mixture concentrated *in vacuo* to afford a grey crystalline solid and then re-dissolved in EtOAc (50 mL). This solution was washed with water (2 x 30 mL) and sat. NaCl (2 x 30 mL), filtered over Na_2SO_4, then concentrated *in vacuo* to afford a yellow/grey solid. The crude solid was purified by column chromatography (solid load, 10-25% EtOAc in hexane) to afford **26** (4.72 g, 22.5 mmol, 82% yield) as a yellow/grey solid. ^{1}H NMR (400 MHz, $CDCl_3$) δ_H ppm 7.19 (d, *J*=7.7 Hz, 1 H, 1-CH), 6.92 (app. td, *J*=7.7, 1.3 Hz, 1 H, 2-CH), 6.70 (app. td, *J*=7.7, 1.3 Hz, 1 H, 3-CH), 6.68 (dd, *J*=7.7, 1.3 Hz, 1 H, 4-CH), 6.18 (br s,

1 H, NH), 3.64 (br s, 2 H, NH_2), 1.44 (s, 9 H, 9-CH_3). ^{13}C NMR (101 MHz, $CDCl_3$) δ_C ppm 153.9 (C7), 140.0 (C6), 126.2 (double intensity: C3, C5), 124.8 (C4), 119.6 (C2), 117.6 (C1), 80.5 (C8), 28.4 (C9). HRMS (ESI) m/z: $[M+Na]^+$ calculated for $C_{11}H_{16}N_2O_2Na$: 231.1109, found 231.1112.

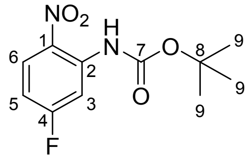

Tert-butyl (5-fluoro-2-nitrophenyl)carbamate (28): A solution of Boc_2O (4.61 g, 19.2 mmol) in THF (5 mL) was added dropwise to a solution of **27** (3.00 g, 19.2 mmol), DMAP (59 mg, 0.48 mmol) and triethylamine (1.54 mL, 11.06 mmol) in THF (35 mL) at 0 °C, and then the mixture was stirred at 80 °C for 15 hours. The reaction mixture was concentrated *in vacuo*, then redissolved in EtOAc (100 mL) and 2M HCl (100 mL) added. The EtOAc layer was collected and then the aqueous layer extracted with further EtOAc (2 x 100 mL). The combined organic layers were dried over Na_2SO_4, filtered and concentrated *in vacuo* to afford an orange oil (5.10 g). The crude product was purified by column chromatography (10-20% EtOAc in hexane) to afford **28** (2.77 g, 10.7 mmol, 56% yield) as a yellow crystalline solid. ^{1}H NMR (400 MHz, $CDCl_3$) δ_H ppm 9.89 (br s, 1 H, 2-NH), 8.41 (dd, J_{HF}=11.6, J_{HH}=2.8 Hz, 1 H, 3-CH), 8.27 (dd, J_{HH}=9.4, J_{HF}=5.9 Hz, 1 H, 6-CH), 6.78 (ddd, J_{HH}=9.4, J_{HF}=6.7, J_{HH}= 2.8 Hz, 1 H, 5-CH), 1.56 (s, 9 H, 9-CH_3). ^{13}C NMR (101 MHz, Chloroform-*d*) δ_C ppm 166.7 (d, J_{CF}=257.1 Hz, C4), 151.9 (C7), 138.6 (d, J_{CF}=14.1 Hz, C2), 132.0 (d, J_{CF}=1.5 Hz, C1), 128.7 (d, J_{CF}=11.6 Hz, C6), 109.5 (d, J_{CF}=24.2 Hz, C5), 107.1 (d, J_{CF}=29.9 Hz, C3), 82.4 (C8), 28.1 (C9). ^{19}F NMR (376 MHz, $CDCl_3$) δ_F ppm -97.7. HRMS (ESI, direct infusion) m/z: $[M-H]^-$ calculated for $C_{11}H_{12}N_2O_4F$: 255.0781, found 255.0780.

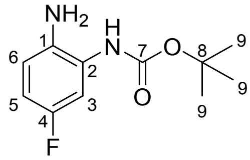

Tert-butyl (2-amino-5-fluorophenyl)carbamate (29): To a solution of **28** (1.68 g, 6.56 mmol) in MeOH (50 mL), 10% Pd/C (170 mg) was added. The reaction flask was filled with nitrogen and evacuated 3 times using a Shlenk line, before a balloon of hydrogen was added and the resultant mixture stirred vigorously for 4.5 hours. The balloon of hydrogen was removed and the flask was flushed with nitrogen. The reaction mixture was filtered through a glass microfiber filter and then the filtrate was concentrated *in vacuo* to afford **29** (1.43 g, 6.26 mmol, 96% yield) as a beige solid. ^{1}H NMR (400 MHz, $CDCl_3$) δ_H ppm 7.35 (br d, J_{HF}=9.4 Hz, 1 H, 3-CH), 6.70 - 6.76 (m, 1 H, 6-CH), 6.64 - 6.70 (m, 1 H, 5-CH), 3.44 (br s, 2 H, 1-NH_2), 1.53 (s, 9 H, 9-CH_3). ^{13}C NMR (101 MHz, $CDCl_3$) δ_C ppm 157.2 (d, J_{CF}=237.5 Hz, C4), 153.1 (C7), 133.2 (d, J_{CF}=1.5 Hz, C2), 127.8 (d, J_{CF}=10.3 Hz, C1), 119.1 (d, J_{CF}=9.2 Hz, C6), 111.0 (d, J_{CF}=22.3 Hz, C5), 109.5 (d, J_{CF}=27.3 Hz, C3), 80.9 (C8), 28.3 (C9). ^{19}F NMR (376 MHz, $CDCl_3$) δ_F ppm -122.4. MS (ESI) m/z: 227 $[M+H]^+$.

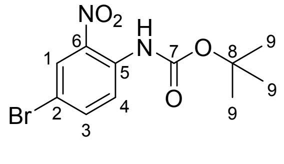

Tert-butyl (4-bromo-2-nitrophenyl)carbamate (31): A solution of Boc_2O (2.21 g, 10.14 mmol) in THF (5 mL) was added dropwise to a solution of **30** (2.00 g, 9.22 mmol), DMAP (0.113 g, 0.92 mmol) and triethylamine (1.54 mL, 11.06 mmol) in THF (35 mL) at 0 °C, and then the mixture was stirred at 60 °C for 16 hours. The reaction mixture was concentrated *in vacuo* and then purified by column chromatography (0-50% EtOAc in hexane) to afford **31** (1.524 g, 4.76 mmol, 47% yield) as a yellow crystalline solid. ^1H NMR (400 MHz, $CDCl_3$) δ_H ppm 9.61 (br s, 1 H, 5-NH), 8.52 (d, *J*=9.2 Hz, 1 H, 4-CH), 8.34 (d, *J*=2.4 Hz, 1 H, 1-CH), 7.69 (dd, *J*=9.2, 2.4 Hz, 1 H, 3-CH), 1.55 (s, 9 H, 9-CH_3). ^{13}C NMR (101 MHz, $CDCl_3$) δ_C ppm 151.9 (C7), 138.5 (C3), 136.1 (C6), 135.1 (C5), 128.2 (C1), 122.2 (C4), 113.7 (C2), 82.3 (C8), 28.1 (C9). HRMS (ESI) m/z: $[(M\text{-}Boc)+H]^+$ calculated for $C_6H_6BrN_2O_4$ (^{79}Br): 216.9613, found 216.9615. HRMS (ESI) m/z: $[(M\text{-}Boc)+H]^+$ calculated for $C_6H_6BrN_2O_4$ (^{81}Br): 218.9592, found 218.9594.

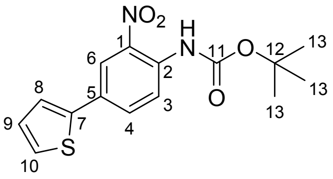

Tert-butyl (2-nitro-4-(thiophen-2-yl)phenyl)carbamate (32): To a solution of DME/water (2:1, 36 mL) was added thiophen-2-ylboronic acid (0.77 g, 6.05 mmol), **31** (1.60 g, 5.05 mmol), Na_2CO_3 (0.80 g, 7.57 mmol) and $Pd(PPh_3)_4$ (0.38 g, 0.33 mmol). The resulted mixture was stirred vigorously at 110 °C for 16 hours. The reaction mixture was diluted with more water (40 mL) and then the product was extracted with EtOAc (3 x 50 mL). The organic layers were combined, washed with water (2 x 70 mL), dried over $MgSO_4$, filtered and concentrated in vacuo to afford a brown solid (1.920 g). The crude product was purified by column chromatography (dry load, 0-50% EtOAc in hexane) to give **32** (1.21 g, 3.74 mmol, 74% yield) as an orange crystalline solid. ^1H NMR (400 MHz, $CDCl_3$) δ_H ppm 9.66 (s, 1 H, 2-NH), 8.60 (d, *J*=8.9 Hz, 1 H, 3-CH), 8.40 (d, *J*=2.1 Hz, 1 H, 6-CH), 7.82 (dd, *J*=8.9, 2.1 Hz, 1 H, 4-CH), 7.32 - 7.36 (m, 2 H, 8,10-CH), 7.11 (dd, *J*=5.1, 3.7 Hz, 1 H, 9-CH), 1.57 (s, 9 H, 13-CH_3). ^{13}C NMR (101 MHz, $CDCl_3$) δ_C ppm 152.1 (C11), 141.3 (C7), 136.0 (C1), 134.8 (C2), 132.9 (C4), 128.7 (C5), 128.3 (C9), 125.6 (C10), 123.9 (C8), 122.3 (C6), 121.2 (C3), 82.0 (C12), 28.2 (C13). HRMS (ESI) m/z: $[M-H]^-$ calculated for $C_{15}H_{15}N_2O_4S$: 319.0753, found 319.0753.

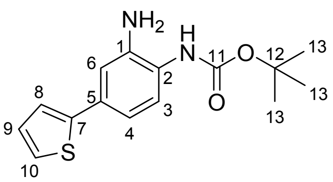

Tert-butyl (2-amino-4-(thiophen-2-yl)phenyl)carbamate (33): To a solution of **32** (1.181 g, 3.69 mmol) in MeOH (15 mL), 10% Pd/C (0.120 g) was added. The reaction flask was filled with nitrogen and evacuated 3 times using a Shlenk line, before a balloon of hydrogen was added and the resultant mixture stirred vigorously for 4 hours. The balloon of hydrogen was removed and the flask was flushed with nitrogen. The reaction mixture was filtered through a glass microfiber filter and then the filtrate was concentrated *in vacuo* to afford **33** (1.057 g, 3.60 mmol, 98% yield) as a brown solid. ^1H NMR (400 MHz, $CDCl_3$) δ_H ppm 7.28 - 7.31 (m, 1 H, 3-CH), 7.24 (dd, J=5.1, 1.1 Hz, 1 H, 8-CH), 7.22 (dd, J=3.6, 1.1 Hz, 1 H, 10-CH), 7.01 - 7.09 (m, 3 H, 4,6,9-CH), 6.35 (br s, 1 H, 2-NH), 3.53 (br s, 2 H, 1-NH_2), 1.52 (s, 9 H, 13-CH_3). ^{13}C NMR (101 MHz, $CDCl_3$)

δ_C ppm 153.8 (C11), 144.0 (C7), 139.5 (C1), 132.3 (C5), 127.9 (C9), 124.9 (C3), 124.5 (C8), 122.8 (C10), 120.7 (C8), 117.8 (C4), 115.2 (C6), 80.9 (C12), 28.3 (C13). HRMS (ESI) m/z: $[M+H]^+$ calculated for $C_{15}H_{19}N_2O_2S$: 291.1167, found 291.1167.

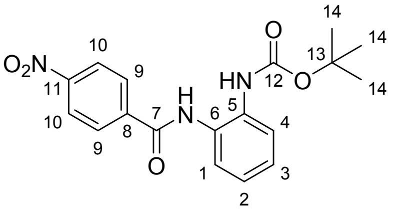

Tert-butyl (2-(4-nitrobenzamido)phenyl)carbamate (34a): Following general method A, **34a** was obtained from **26** (4.17 g, 20.0 mmol) and 4-nitrobenzoyl chloride (4.09 g, 22.0 mmol). The crude product was triturated in EtOH and then filtered to afford **34a** (5.52 g, 15.3 mmol, 76% yield) as a pale yellow solid. ^{1}H NMR (400 MHz, $CDCl_3$) δ_H ppm 9.79 (s, 1 H, NH), 8.30 (d, *J*=8.9 Hz, 2 H, 10-CH), 8.14 (d, *J*=8.9 Hz, 2 H, 9-CH), 7.84 (d, *J*=7.7 Hz, 1 H, 1-CH), 7.20 - 7.26 (m, 1 H, 3-CH), 7.14 - 7.18 (m, 2 H, 2-CH,4-CH), 6.86 (s, 1 H, NH), 1.52 (s, 9 H, 14-CH_3). ^{13}C NMR (101 MHz, $CDCl_3$) δ_C ppm 163.3 (C7), 155.0 (C12), 149.8 (C8), 140.0 (C11), 130.5 (C5), 129.6 (C6), 128.6 (C9), 126.3 (C2), 126.2 (C3), 125.9 (C1), 124.4 (C4), 123.7 (C10), 82.0 (C13), 28.3 (C14). HRMS (ESI) m/z: $[M+Na]^+$ calculated for $C_{18}H_{19}N_3O_5Na$: 380.1222, found 380.1223.

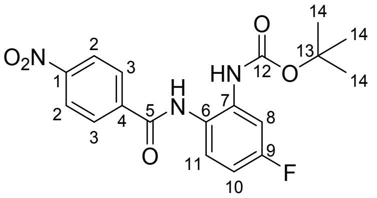

Tert-butyl (5-fluoro-2-(4-nitrobenzamido)phenyl)carbamate (34b): Following general method A, **34b** was obtained from **29** (1.27 g, 5.60 mmol) and 4-nitrobenzoyl chloride (1.15 g, 6.17 mmol). The crude product was purified by column chromatography (0-50% EtOAc in hexane) to afford **34b** (1.87 g, 4.93 mmol, 88% yield) as a fluffy, pale brown solid. ^{1}H NMR (400 MHz, $CDCl_3$) δ_H ppm 9.52 (br s, 1 H, 6-NH), 8.32 (d, *J*=8.8 Hz, 2 H, 2-CH), 8.13 (d, *J*=8.8 Hz, 2 H, 3-CH), 7.64 (dd, J_{HH}=8.9, J_{HF}=5.8 Hz, 1 H, 11-CH), 6.94 - 7.02 (m, 2 H, 8-CH, 7-NH), 6.86 - 6.93 (m, 1 H, 10-CH), 1.53 (s, 9 H, 14-CH_3). ^{13}C NMR (101 MHz, $CDCl_3$) δ_C ppm 163.6 (C5), 160.3 (d, J_{CF}=246.2 Hz, C9), 154.5 (C12), 149.9 (C1), 139.4 (C4), 131.8 (d, J_{CF}=10.5 Hz, C7), 128.5 (C3), 127.5 (d, J_{CF}=9.3 Hz, 11), 125.8 (d, J_{CF}=3.2 Hz, C6), 123.8 (C2), 112.7 (d, J_{CF}=22.3 Hz, C10), 111.1 (d, J_{CF}=25.2 Hz, C8), 82.3 (C13), 28.2 (C14). ^{19}F NMR (376 MHz, $CDCl_3$) δ_F ppm -114.2. HRMS (ESI) m/z: $[M+Na]^+$ calculated for $C_{18}H_{18}N_3O_5FNa$: 398.1128, found 398.1125.

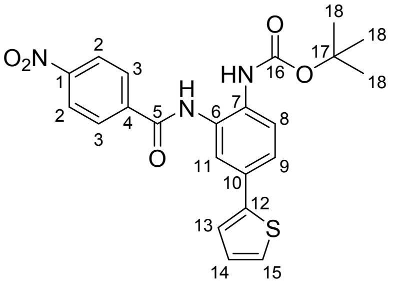

Tert-butyl (2-(4-nitrobenzamido)-4-(thiophen-2-yl)phenyl)carbamate (34c): Following general method A, **34c** was obtained from **33** (400 mg, 1.38 mmol) and 4-nitrobenzoyl chloride (284 mg, 1.53 mmol). The

crude product was triturated in EtOH and then filtered to afford **34c** (459 mg, 1.04 mmol, 76% yield) as a pale green solid. ^{1}H NMR (400 MHz, $CDCl_3$) δ_H ppm 10.19 (s, 1 H, 6-NH), 8.81 (s, 1 H, 7-NH), 8.40 (d, *J*=8.8 Hz, 2 H, 2-CH), 8.23 (d, *J*=8.8 Hz, 2 H, 3-CH), 7.79 (d, *J*=2.0 Hz, 1 H, 11-CH), 7.70 (d, *J*=8.4 Hz, 1 H, 8-CH), 7.54 (dd, *J*=8.4, 2.0 Hz, 1 H, 9-CH), 7.52 (dd, *J*=5.0, 1.0 Hz, 1 H, 13-CH), 7.46 (dd, *J*=3.6, 1.0 Hz, 1 H, 15-CH), 7.13 (dd, *J*=5.0, 3.6 Hz, 1 H, 14-CH), 1.45 (s, 9 H, 18-CH_3). ^{13}C NMR (101 MHz, $CDCl_3$) δ_C ppm 164.1 (C5), 153.2 (C16), 149.3 (C4), 142.6 (C12), 140.1 (C1), 132.0 (C10), 129.4 (C3), 129.3 (C6), 129.1 (C7), 128.5 (C14), 125.4 (C13), 123.7 (C8), 123.5 (C2), 123.4 (C15), 123.4 (C11), 123.1 (C9), 79.7 (C17), 28.0 (C18). HRMS (ESI) m/z: $[M+H]^+$ calculated for $C_{22}H_{22}N_3O_5S$: 440.1280, found 440.1280.

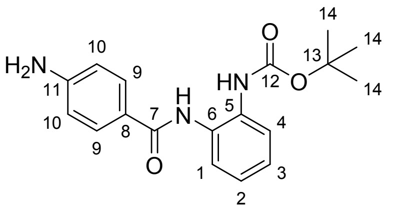

Tert-butyl (2-(4-aminobenzamido)phenyl)carbamate (35a): To a solution of **34a** (5.52 g, 15.3 mmol) in MeOH/THF (1:1, 100 mL), 10% Pd/C (0.55 g) was added. The reaction flask was filled with nitrogen and evacuated 3 times using a Shlenk line, before a balloon of hydrogen was added and the resultant mixture stirred vigorously for 18 hours. The balloon of hydrogen was removed and the flask was flushed with nitrogen. The reaction mixture was filtered through celite, then the celite was washed with more MeOH (3 x 50 mL) and the filtrate concentrated *in vacuo* to afford **35a** (5.23 g, 15.3 mmol, 100% yield) as a fluffy white crystalline solid. ^{1}H NMR (400 MHz, $CDCl_3$) δ_H ppm 8.87 (s, 1 H, NH), 7.78 (d, *J*=8.7 Hz, 2 H, 9-CH), 7.64 (dd, *J*=7.7, 1.7 Hz, 1 H, 1-CH), 7.29 (dd, *J*=7.7, 1.7 Hz, 1 H, 4-CH), 7.14 (app. td, *J*=7.7, 1.7 Hz, 1 H, 2-CH), 7.12 (app. td, *J*=7.7, 1.7 Hz, 1 H, 3-CH), 7.06 (s, 1 H, NH), 6.66 (d, *J*=8.7 Hz, 2 H, 10-CH), 4.05 (s, 2 H, 11-NH_2), 1.51 (s, 9 H, 14-CH_3). ^{13}C NMR (101 MHz, $CDCl_3$) δ_C ppm 165.7 (C7), 154.6 (C12), 150.1 (C8), 131.0 (C6), 130.3 (C5), 129.3 (C9), 125.7 (C1), 125.6 (C2,C3), 124.5 (C4), 123.5 (C11), 114.1 (C10), 81.0 (C13), 28.3 (C14). HRMS (ESI) m/z: $[M+Na]^+$ calculated for $C_{18}H_{21}N_3O_3Na$: 350.1481, found 350.1486.

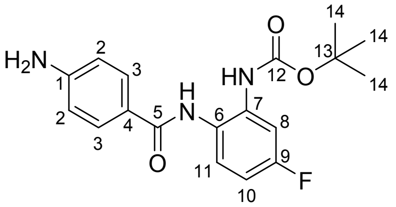

Tert-butyl (2-(4-aminobenzamido)-5-fluorophenyl)carbamate (35b): To a solution of **34b** (1.525 g, 4.063 mmol) in MeOH (100 mL), 10% Pd/C (0.155 g) was added. The reaction flask was filled with nitrogen and evacuated 3 times using a Shlenk line, before a balloon of hydrogen was added and the resultant mixture stirred vigorously for 7 hours. The balloon of hydrogen was removed and the flask was flushed with nitrogen. The reaction mixture was filtered through a glass microfiber filter and then the filtrate was concentrated *in vacuo* to afford **35b** (1.335 g, 3.827 mmol, 94% yield) as a pale grey crystalline solid. ^{1}H NMR (400 MHz, $CDCl_3$) δ_H ppm 8.61 (br s, 1 H, 6-NH), 7.76 (d, *J*=8.6 Hz, 2 H, 3-CH), 7.38 (dd, J_{HH}=8.9, J_{HF}=5.8 Hz, 1 H, 11-CH), 7.19 - 7.24 (m, 2 H, 8-CH, 7-NH), 6.74 - 6.81 (m, 1 H, 10-CH), 6.67 (d, *J*=8.6 Hz, 2 H, 2-CH), 4.08 (br s, 2 H, 1-NH_2), 1.50 (s, 9 H, 14-CH_3). ^{13}C NMR (101 MHz, $CDCl_3$) δ_C ppm 166.1 (C5), 160.3 (d, J_{CF}=244.5 Hz, 1

C9), 153.9 (C12), 150.2 (C1), 132.9 (d, J_{CF}=10.9 Hz, C7), 129.3 (C3), 127.3 (d, J_{CF}=9.5 Hz, C11), 125.7 (d, J_{CF}=3.1 Hz, C6), 122.9 (C4), 114.1 (C2), 111.6 (d, J_{CF}=22.5 Hz, C10), 110.6 (d, J_{CF}=25.9 Hz, C8), 81.3 (C13), 28.2 (C14). ^{19}F NMR (376 MHz, $CDCl_3$) δ_F ppm -115.1. HRMS (ESI) m/z: $[M+H]^+$ calculated for $C_{18}H_{21}N_3O_3F$: 346.1567, found 346.1563.

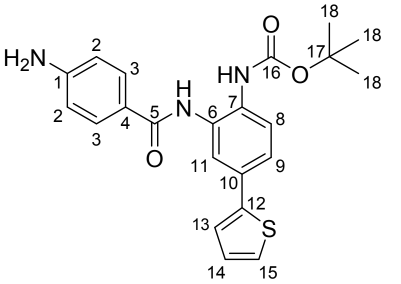

Tert-butyl (2-(4-aminobenzamido)-4-(thiophen-2-yl)phenyl)carbamate (35c): To a solution of **34c** (0.333 g, 0.76 mmol) in MeOH/DCM (1:1, 120 mL), $SnCl_2$ (0.863 g, 4.55 mmol) was added and the resultant mixture stirred at room temperature for 1 week. The reaction mixture was cooled to 0 °C, then saturated Na_2CO_3 (40 mL) was added slowly. The product was extracted into DCM (4 x 40 mL), then the organic layers were combined and washed with brine (2 x 80 mL). The organic layer was dried over Na_2SO_4, filtered and concentrated *in vacuo* to afford **35c** (0.283 g, 0.62 mmol, 82% yield) as a yellow crystalline solid. ^{1}H NMR (400 MHz, DMSO-d_6) δ_H ppm 9.59 (s, 1 H, 6-NH), 8.72 (br s, 1 H, 7-NH), 7.82 (d, J=2.1 Hz, 1 H, 11-CH), 7.71 (d, J=8.4 Hz, 2 H, 3-CH), 7.55 (d, J=8.5 Hz, 1 H, 8-CH), 7.52 (dd, J=5.0, 1.0 Hz, 1 H, 13-CH), 7.47 (dd, J=8.5, 2.1 Hz, 1 H, 9-CH), 7.44 (dd, J=3.6, 1.0 Hz, 1 H, 15-CH), 7.13 (dd, J=5.0, 3.6 Hz, 1 H, 14-CH), 6.62 (d, J=8.4 Hz, 2 H, 2-CH), 5.85 (br s, 2 H, 1-NH_2), 1.47 (s, 9 H, 18-CH_3). ^{13}C NMR (101 MHz, DMSO-d_6) δ_C ppm 165.4 (C5), 153.4 (C16), 152.6 (C4), 142.8 (C12), 130.8 (C6), 130.7 (C10), 129.7 (C7), 129.4 (C3), 128.5 (C14), 125.4 (C13), 124.3 (C8), 123.4 (C15), 122.4 (C11), 122.0 (C9), 120.0 (C1), 112.6 (C2), 79.8 (C17), 28.0 (C18). HRMS (ESI) m/z: $[M+H]^+$ calculated for $C_{22}H_{24}N_3O_3S$: 410.1538, found 410.1530.

Scheme S2. Synthesis of Linker Intermediates.

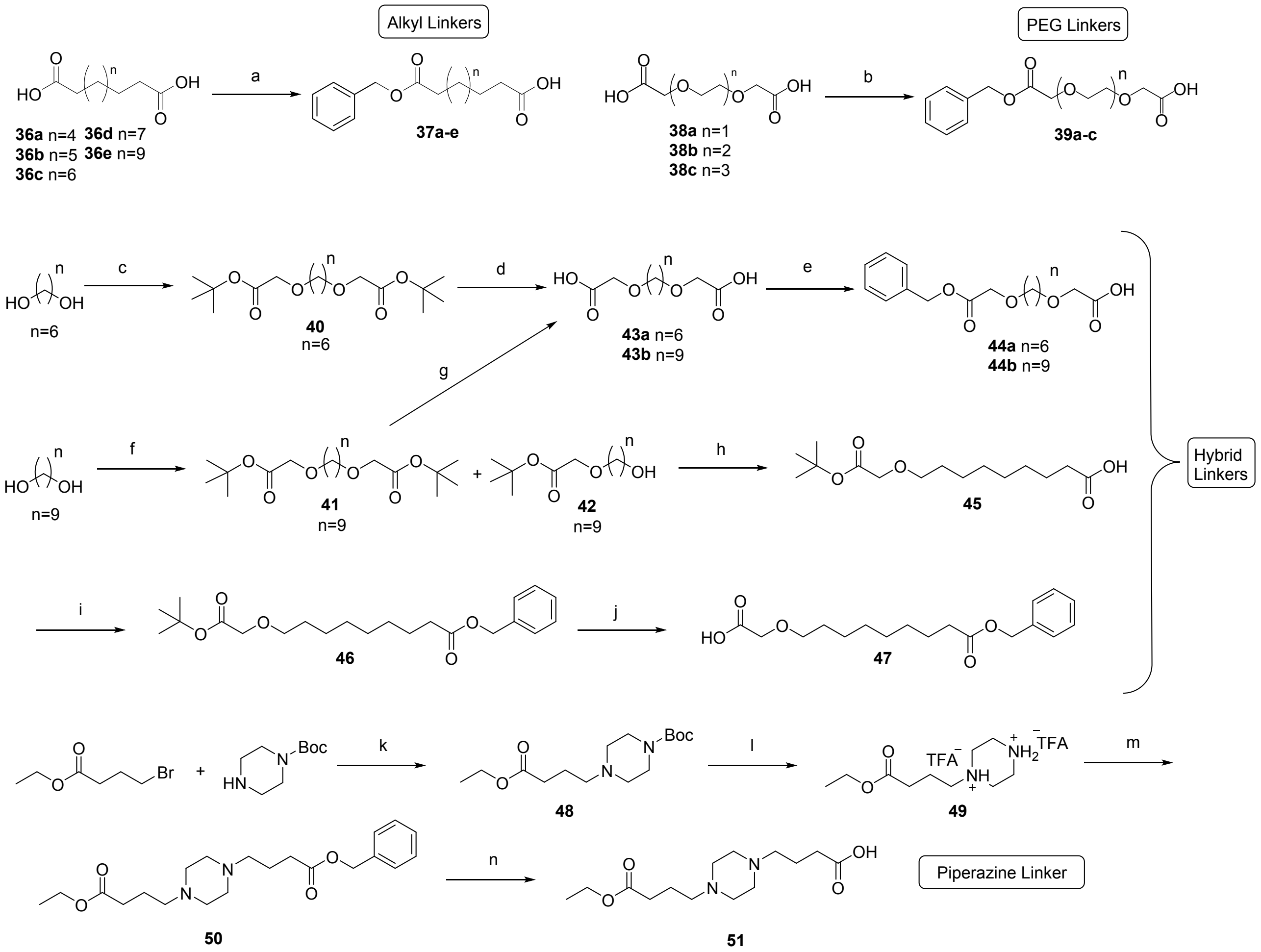

Reagents and conditions: (a) BnBr, $NaHCO_3$, 1,4-dioxane:DMF = 1:1, 90 ºC, overnight; (b) BnBr, $NaHCO_3$, 1,4-dioxane:DMF = 1:1, 90 ºC, overnight; (c) tert-butyl 2-bromoacetate, tetra-n-butylammonium bromide ($N(^nBu)_4Br$), NaOH (aq., 37% w/w), DCM, 0 ºC-r.t., 16 h; (d) TFA, DCM, r.t., 4 h; (e) BnBr, $NaHCO_3$, 1,4-dioxane:DMF = 1:1, 90 ºC, overnight; (f) tert-butyl 2-bromoacetate, tetra-n-butylammonium bromide ($N(^nBu)_4Br$), NaOH (aq., 37% w/w), DCM, 0 ºC-r.t., 16 h; (g) TFA, DCM, r.t., 4 h; (h) pyridinium dichromate (PDC), DMF, r.t., 6 h; (i) BnBr, NEt_3, 1,4-dioxane:DMF = 1:1, r.t., 5 h; (j) TFA, DCM, r.t., 4 h; (k) NEt_3, THF, r.t., overnight; (l) TFA, DCM, r.t., overnight; (m) benzyl 4-bromobutanoate, Na_2CO_3, MeCN, 60 ºC, overnight; (n) H2, Pd/C (10% weight), EtOH, r.t., overnight.

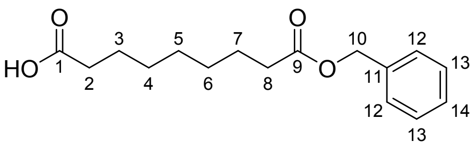

9-(benzyloxy)-9-oxononanoic acid (37a): Following general method D, **37a** was obtained from **36a** (2.00 g, 10.63 mmol) and benzyl bromide (1.26 mL, 10.63 mmol). The crude product was purified by column chromatography (50% EtOAc in hexane) to afford **37a** (1.083 g, 3.85 mmol, 36% yield) as a clear colourless

oil. ^{1}H NMR (400 MHz, $CDCl_3$) δ_H ppm 10.67 (br s, 1 H, CO_2H), 7.29 - 7.42 (m, 5 H, (12-14)-CH), 5.12 (s, 2 H, 10-CH_2), 2.31 - 2.39 (m, 4 H, 2-CH_2, 8-CH_2), 1.55 - 1.72 (m, 4 H, 3-CH_2, 7-CH_2), 1.28 - 1.37 (m, 6 H, (4-6)-CH_2). ^{13}C NMR (101 MHz, $CDCl_3$) δ_C ppm 179.8 (C1), 173.6 (C9), 136.1 (C11), 128.5 (C13), 128.2 (C12), 128.1 (C14), 66.1 (C10), 34.2 (C8), 33.9 (C2), 28.9 (CH_2), 28.85 (CH_2), 28.8 (CH_2), 24.8 (C7), 24.5 (C3). HRMS (ESI) m/z: $[M+Na]^+$ calculated for $C_{16}H_{22}O_4Na$: 301.1416, found 301.1415.

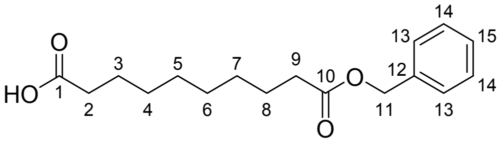

10-(benzyloxy)-10-oxodecanoic acid (37b): Following general method D, **37b** was obtained from **36b** (2.00 g, 9.89 mmol) and benzyl bromide (1.17 mL, 9.89 mmol). The crude product was purified by column chromatography (25% EtOAc in hexane) to afford **37b** (1.224 g, 4.19 mmol, 42% yield) as a clear colourless oil. ^{1}H NMR (400 MHz, $CDCl_3$) δ_H ppm 10.94 (br s, 1 H, 1-CO_2H), 7.30 - 7.41 (m, 5 H, (13-15)-CH), 5.13 (s, 2 H, 11-CH_2), 2.31 - 2.40 (m, 4 H, 2-CH_2,9-CH_2), 1.58 - 1.70 (m, 4 H, 3-CH_2,8-CH_2), 1.27 - 1.37 (m, 8 H, (4-7)-CH_2). ^{13}C NMR (101 MHz, $CDCl_3$) δ_C ppm 179.9 (C1), 173.7 (C10), 136.1 (C12), 128.5 (C14), 128.2 (C13), 128.1 (C15), 66.1 (C11), 34.3 (C9), 34.0 (C2), 29.0 (2x alkyl CH_2), 28.95 (alkyl CH_2), 28.9 (alkyl CH_2), 24.9 (C8), 24.6 (C3). HRMS (ESI) m/z: $[M+Na]^+$ calculated for $C_{17}H_{24}O_4Na$: 315.1572, found 315.1575.

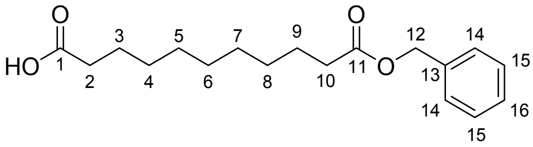

11-(benzyloxy)-11-oxoundecanoic acid (37c): Following general method D, **37c** was obtained from **36c** (2.00 g, 9.25 mmol) and benzyl bromide (1.10 mL, 9.25 mmol). The crude product was purified by column chromatography (50% EtOAc in hexane) to afford **37c** (0.641 g, 2.09 mmol, 23% yield) as a clear colourless oil. ^{1}H NMR (400 MHz, $CDCl_3$) δ_H ppm 10.82 (br s, 1 H, 1-CO_2H), 7.30 - 7.40 (m, 5 H, (14-16)-CH), 5.13 (s, 2 H, 12-CH_2), 2.32 - 2.40 (m, 4 H, 2-CH_2,10-CH_2), 1.59 - 1.71 (m, 4 H, 3-CH_2,9-CH_2), 1.26 - 1.37 (m, 10 H, (4-8)-CH_2). ^{13}C NMR (101 MHz, $CDCl_3$) δ_C ppm 180.0 (C1), 173.7 (C11), 136.1 (C13), 128.5 (C15), 128.2 (C14), 128.1 (C16), 66.1 (C12), 34.3 (C10), 34.0 (C2), 29.2 (alkyl CH_2), 29.15 (alkyl CH_2), 29.1 (alkyl CH_2), 29.05 (alkyl CH_2), 29.0 (alkyl CH_2), 24.9 (C9), 24.6 (C3). HRMS (ESI) m/z: $[M+Na]^+$ calculated for $C_{18}H_{26}O_4Na$: 329.1729, found 329.1734.

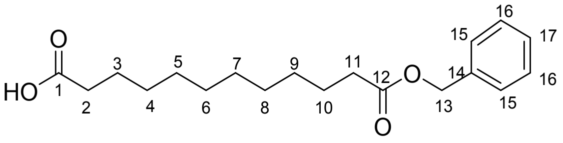

12-(benzyloxy)-12-oxododecanoic acid (37d): Following general method D, **37d** was obtained from **36d** (2.00 g, 8.68 mmol) and benzyl bromide (1.03 mL, 8.68 mmol). The crude product was purified by column chromatography (50% EtOAc in hexane) to afford **37d** (0.998g, 2.99 mmol, 34% yield) as a clear colourless oil. ^{1}H NMR (400 MHz, $CDCl_3$) δ_H ppm 11.07 (br s, 1 H, 1-CO_2H), 7.32 - 7.40 (m, 5 H, (15-17)-CH), 5.13 (s,

2 H, 13-CH_2), 2.30 - 2.42 (m, 4 H, 2-CH_2,11-CH_2), 1.57 - 1.71 (m, 4 H, 3-CH_2,10-CH_2), 1.23 - 1.37 (m, 12 H, (4-9)-CH_2). ^{13}C NMR (101 MHz, $CDCl_3$) δ_C ppm 179.9 (C1), 173.7 (C12), 136.1 (C14), 128.5 (C16), 128.2 (C15), 128.1 (C17), 66.1 (C13), 34.3 (C11), 34.0 (C2), 29.4 (alkyl CH_2), 29.3 (alkyl CH_2), 29.2 (2x alkyl CH_2), 29.1 (alkyl CH_2), 29.0 (alkyl CH_2), 24.9 (C10), 24.6 (C3). HRMS (ESI) m/z: $[M+Na]^+$ calculated for $C_{19}H_{28}O_4Na$: 343.1885, found 343.1887.

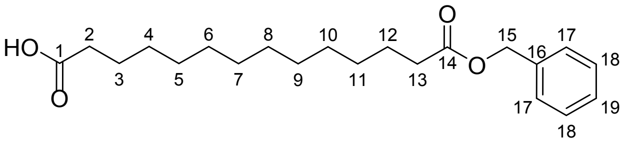

14-(benzyloxy)-14-oxotetradecanoic acid (37e): Following general method D, **37e** was obtained from **36e** (1.50 g, 5.81 mmol) and benzyl bromide (0.69 mL, 5.81 mmol). The crude product was purified by column chromatography (50% EtOAc in hexane) to afford **37e** (0.628 g, 1.78 mmol, 31% yield) as a clear colourless oil. ^{1}H NMR (400 MHz, $CDCl_3$) δ_H ppm 11.09 (s, 1 H, 1-CO_2H), 7.31 - 7.40 (m, 5 H, (17-19)-CH), 5.13 (s, 2 H, 15-CH_2), 2.31 - 2.40 (m, 4 H, (2,13-CH_2), 1.59 - 1.70 (m, 4 H, 3,12-CH_2), 1.21 - 1.39 (m, 16 H, (4-11)-CH_2). ^{13}C NMR (101 MHz, $CDCl_3$) δ_C ppm 179.8 (C1), 173.7 (C14), 136.1 (C16), 128.5 (C18), 128.15 (C17), 128.1 (C19), 66.0 (C15), 34.3 (C13), 34.0 (C2), 29.5 (alkyl CH_2 x 2), 29.4 (alkyl CH_2), 29.4 (alkyl CH_2), 29.2 (alkyl CH_2 x 2), 29.1 (alkyl CH_2), 29.0 (alkyl CH_2), 24.9 (C12), 24.6 (C3). HRMS (ESI) m/z: $[M+H]^+$ calculated for $C_{21}H_{33}O_4$: 349.2379, found 349.2374.

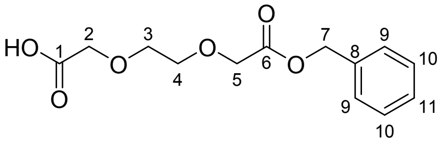

2-(2-(2-(benzyloxy)-2-oxoethoxy)ethoxy)acetic acid (39a): Following general method D, **39a** was obtained from **38a** (0.60 g, 3.37 mmol) and benzyl bromide (0.40 mL, 3.37 mmol). The crude product was purified by column chromatography (0-10% MeOH in DCM) to afford **39a** (0.508 g, 1.25 mmol, 37% yield) as a clear yellow oil. ^{1}H NMR (400 MHz, $CDCl_3$) δ_H ppm 9.07 (br s, 1 H, 1-OH) 7.32 - 7.39 (m, 5 H, (9-11)-CH) 5.20 (s, 2 H, 7-CH_2) 4.21 (s, 2 H, 5-CH_2) 4.18 (s, 2 H, 2-CH_2) 3.77 - 3.80 (m, 4 H, 3-CH_2,4-CH_2). ^{13}C NMR (101 MHz, $CDCl_3$) δ_C ppm 173.2 (C1), 170.1 (C6), 135.2 (C8), 128.6 (C10), 128.5 (C11), 128.4 (C9), 71.0 (C3), 70.8 (C4), 68.5 (C2), 68.4 (C5), 66.7 (C7). HRMS (ESI) m/z: $[M+H]^+$ calculated for $C_{13}H_{17}O_6$: 269.1025, found 269.1025.

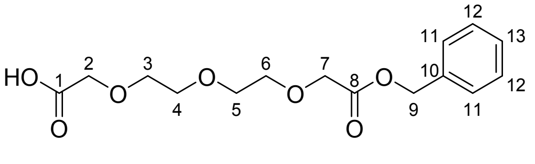

3-oxo-1-phenyl-2,5,8,11-tetraoxatridecan-13-oic acid (39b): Following general method D, **39b** was obtained from **38b** (1.00 g, 3.15 mmol) and benzyl bromide (0.37 mL, 3.15 mmol). The crude product was purified by column chromatography (0-10% MeOH in DCM) to afford **39b** (0.484 g, 1.53 mmol, 49% yield) as a clear yellow oil. ^{1}H NMR (400 MHz, $CDCl_3$) δ_H ppm 7.35 - 7.40 (m, 5 H, (11-13)-CH), 5.20 (s, 2 H, 9-CH_2), 4.20 (s, 2 H, 7-CH_2), 4.15 (s, 2 H, 2-CH_2), 3.73 - 3.77 (m, 6 H, (3,5,6)-CH_2), 3.69 - 3.72 (m, 2 H, 4-CH_2). ^{13}C NMR (101 MHz, $CDCl_3$) δ_C ppm 172.9 (C1), 170.4 (C8), 135.3 (C10), 128.6 (C12), 128.5 (C13),

128.4 (C11), 71.2 (C3), 70.7 (C6), 70.5 (C5), 70.2 (C4), 68.7 (C2), 68.5 (C7), 66.6 (C9). HRMS (ESI) m/z: $[M+H]^+$ calculated for $C_{15}H_{21}O_7$: 313.1287, found 313.1290.

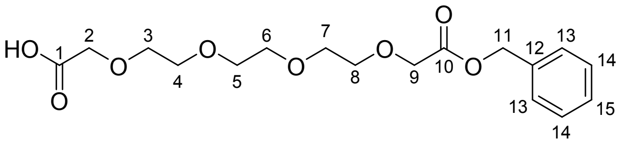

3-oxo-1-phenyl-2,5,8,11,14-pentaoxahexadecan-16-oic acid (39c): Following general method D, **39c** was obtained from **38c** (1.30 g, 4.62 mmol) and benzyl bromide (0.38 mL, 4.68 mmol). The crude product was purified by column chromatography (0-10% MeOH in DCM) to afford **39c** (0.503 g, 1.38 mmol, 30 %) as a clear yellow oil. ^{1}H NMR (400 MHz, $CDCl_3$) δ_H 8.13 (br s, 1 H, 1-OH), 7.32 - 7.48 (m, 5 H, (13-15)-CH), 5.18 (s, 2 H, 11-CH_2), 4.19 (s, 2 H, 9-CH_2), 4.14 (s, 2 H, 2-CH_2), 3.65 - 3.75 (m, 12 H, (3-8)-CH_2). ^{13}C NMR (101 MHz, $CDCl_3$) δ_C 173.0 (C1), 170.4 (C10), 135.4 (C12), 128.6 (C14), 128.5 (C15), 128.4 (C13), 71.0 (C5), 70.9 (C6), 70.5 (C4), 70.45 (C12), 70.35 (C3), 70.3 (C8), 68.7 (C9), 68.5 (C2), 66.6 (C11). HRMS (ESI) m/z: $[M+H]^+$ calculated for $C_{17}H_{25}O_8$: 357.1549, found 357.1546.

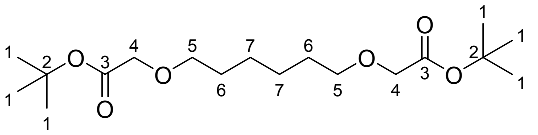

Bi-tert-butyl 2,2'-(hexane-1,6-diylbis(oxy))diacetate (40): Hexane-1,6-diol (1.50 g, 12.69 mmol) was dissolved in DCM (52 ml) before tert-butyl 2-bromoacetate (15.0 ml, 102.28 mmol) was added dropwise, followed by the addition of tetra-n-butylammonium bromide (4.50 g, 13.96 mmol). The reaction mixture was then cooled to 0 ˚C before NaOH (37% w/w, 52 ml) was added. The reaction mixture was then allowed to stir vigorously at room temperature for 16 hours. The crude reaction mixture was biphasic and the organic layer (top layer) was yellow in colour. The organic layer was separated then the aqueous layer was washed with DCM (3 x 15 ml). The organic layers were combined, dried over $MgSO_4$ before being filtered and concentrated *in vacuo* to yield a clear pale yellow oil (6.87 g), which slowly crystallised. The crude product was purified by column chromatography (1% MeOH in DCM) to afford **40** (1.015 g, 2.90 mmol, 23% yield) as a clear colourless oil. ^{1}H NMR (400 MHz, $CDCl_3$) δ_H ppm 3.94 (s, 4 H, 4-CH_2), 3.50 (t, *J*=6.6 Hz, 4 H, 5-CH_2), 1.59 - 1.67 (m, 4 H, 6-CH_2), 1.48 (s, 18 H, 1-CH_3), 1.36 - 1.43 (m, 4 H, 7-CH_2). ^{13}C NMR (101 MHz, $CDCl_3$) δ_C ppm 169.8 (C3), 81.4 (C2), 71.7 (C5), 68.8 (C4), 29.6 (C6), 28.1 (C1), 25.9 (C7). HRMS (ESI) m/z: $[M+Na]^+$ calculated for $C_{18}H_{34}O_6Na$: 369.2253, found 369.2255.[11]

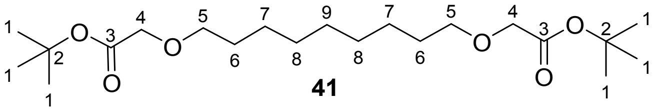
41
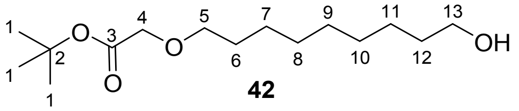
42

Di-tert-butyl 2,2'-(nonane-1,9-diylbis(oxy))diacetate (41) and tert-butyl 2-((9-hydroxynonyl) oxy)acetate (42): Nonane-1,9-diol (2.00 g, 12.48 mmol) was dissolved in DCM (50 ml) before tert-butyl 2-bromoacetate (5.53 ml, 37.44 mmol) was added dropwise, followed by the addition of tetra-n-butylammonium bromide (4.43 g, 13.73 mmol). The reaction mixture was then cooled to 0 ˚C before NaOH (37% w/w, 50 ml)

was added. The reaction mixture was then allowed to stir vigorously at room temperature for 16 hours. The crude reaction mixture was biphasic and the organic layer (top layer) was pale yellow in colour. The organic layer was separated then the aqueous layer was washed with DCM (3 x 15 ml). The organic layers were combined, dried over $MgSO_4$ before being filtered and concentrated *in vacuo* to yield a clear pale yellow oil. The crude product was purified by column chromatography (10-30% EtOAc in hexane) to afford **41** (2.80 g, 7.14 mmol, 57% yield) and **42** (1.387 g, 5.01 mmol, 40% yield) as clear colourless oils. **41**; ^1H NMR (400 MHz, $CDCl_3$) δ_H ppm 3.92 (s, 4 H, 4-CH_2), 3.48 (t, *J*=6.6 Hz, 4 H, 5-CH_2), 1.54 - 1.63 (m, 4 H, 6-CH_2), 1.46 (s, 18 H, 1-CH_3), 1.25 - 1.37 (m, 10 H, (7-9)-CH_2). ^{13}C NMR (101 MHz, $CDCl_3$) δ_C ppm 169.8 (C3), 81.3 (C2), 71.7 (C5), 68.7 (C4), 29.6 (C6), 29.4 (C8/9), 29.3 (C8/9), 28.0 (C1), 25.9 (C7). HRMS (ESI) m/z: $[M+Na]^+$ calculated for $C_{21}H_{40}O_6Na$: 411.2723, found 411.2723. **42**; ^1H NMR (400 MHz, $CDCl_3$) δ_H ppm 3.94 (s, 2 H, 4-CH_2), 3.64 (t, *J*=6.6 Hz, 2 H, 13-CH_2), 3.50 (t, *J*=6.6 Hz, 2 H, 5-CH_2), 2.01 (s, 1 H, OH), 1.58 - 1.65 (m, 2 H, 6-CH_2), 1.52 - 1.58 (m, 2 H, 12-CH_2), 1.48 (s, 9 H, 1-CH_3), 1.27 - 1.40 (m, 10 H, (7-11)-CH_2). ^{13}C NMR (101 MHz, $CDCl_3$) δ_C ppm 169.9 (C3), 81.4 (C2), 71.8 (C5), 68.7 (C4), 63.0 (C13), 32.7 (C12), 29.6 (C6), 29.4 (alkyl CH_2), 29.3 (alkyl CH_2), 29.3 (alkyl CH_2), 28.1 (C1), 26.0 (alkyl CH_2), 25.7 (alkyl CH_2). HRMS (ESI) m/z: $[M+Na]^+$ calculated for $C_{15}H_{30}O_4Na$: 297.2042, found 297.2046.

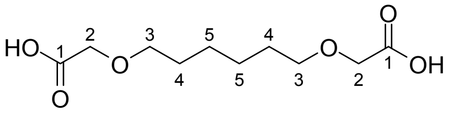

2,2'-(hexane-1,6-diylbis(oxy))diacetic acid (43a): TFA (0.4 mL) was added to a stirring solution of **40** (0.460 g, 1.33 mmol) in DCM (2 mL) and the resulting reaction mixture stirred at room temperature for 4 hours. The reaction mixture was concentrated *in vacuo* to afford **43a** (319.0 mg, 1.36 mmol, 99% yield) as a white solid. ^1H NMR (400 MHz, DMSO-*d6*) δ_H ppm 12.21 (br s, 2H, 1-CO_2H), 3.87 (s, 4 H, 2-CH_2), 3.34 (t, *J*=6.6 Hz, 4 H, 3-CH_2), 1.36 - 1.46 (m, 4 H, 4-CH_2), 1.17 - 1.26 (m, 4 H, 5-CH_2). ^{13}C NMR (101 MHz, DMSO-*d6*) δ_C ppm 171.8 (C1), 70.5 (C3), 67.4 (C2), 29.1 (C4), 25.4 (C5). HRMS (ESI) m/z: $[M+H]^+$ calculated for $C_{10}H_{19}O_6$: 235.1182, found 235.1182.

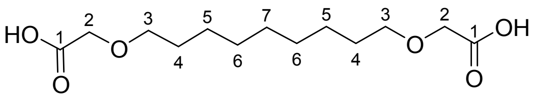

2,2'-(nonane-1,9-diylbis(oxy))diacetic acid (43b): TFA (3 mL) was added to a stirring solution of **41** (1.55 g, 3.99 mmol) in DCM (3 mL) and the resulting reaction mixture stirred at room temperature for 4.5 hours. The reaction mixture was concentrated *in vacuo* to afford **43b** (1.09 g, mmol, 3.95 mmol, 99% yield) as a white solid. ^1H NMR (400 MHz, DMSO-*d6*) δ_H ppm 12.49 (br s, 2 H, 1-CO_2H), 3.95 (s, 4 H, 2-CH_2), 3.41 (t, *J*=6.6 Hz, 4 H, 3-CH_2), 1.44 - 1.53 (m, 4 H, 4-CH_2), 1.21 - 1.32 (m, 10 H, (5-7)-CH_2). ^{13}C NMR (101 MHz, DMSO-*d6*) δ_C ppm 171.8 (C1), 70.5 (C3), 67.4 (C2), 29.1 (C4), 29.0 (C7), 28.8 (C6), 25.6 (C5). HRMS (ESI) m/z: $[M+H]^+$ calculated for $C_{13}H_{25}O_6$: 277.1651, found 277.1656.

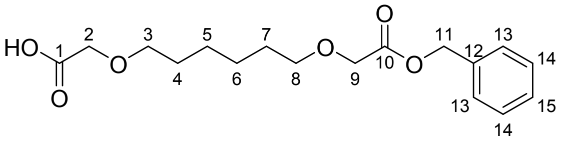

2-((6-(2-(benzyloxy)-2-oxoethoxy)hexyl)oxy)acetic acid (44a): Following general method D, **44a** was obtained from **43a** (0.304 g, 1.30 mmol) and benzyl bromide (0.15 mL, 1.30 mmol). The crude product was purified by column chromatography (0-100% EtOAc in hexane) to afford **44a** (0.110 g, 0.34 mmol, 26% yield) as a yellow oil. ^{1}H NMR (400 MHz, $CDCl_3$) δ_H ppm 8.42 (br s, 1 H, 1-CO_2H), 7.32 - 7.39 (m, 5 H, (12-15)-CH), 5.20 (s, 2 H, 11-CH_2), 4.12 (s, 2 H, 9-CH_2), 4.10 (s, 2 H, 2-CH_2), 3.56 (t, *J*=6.6 Hz, 2 H, 3-CH_2), 3.54 (t, *J*=6.6 Hz, 2 H, 8-CH_2), 1.58 - 1.70 (m, 4 H, 4,7-CH_2), 1.35 - 1.46 (m, 4 H, 5,6-CH_2). ^{13}C NMR (101 MHz, $CDCl_3$) δ_C ppm 173.9 (C1), 170.5 (C10), 135.4 (C12), 128.6 (C14), 128.4 (C15), 128.35 (C13), 71.9 (C3), 71.8 (C8), 68.2 (C9), 67.7 (C2), 66.5 (C11), 29.4 (C7), 29.3 (C4), 25.7 (C6), 25.6 (C5). HRMS (ESI) m/z: $[M+H]^+$ calculated for $C_{17}H_{25}O_6$: 325.1651, found 325.1659.

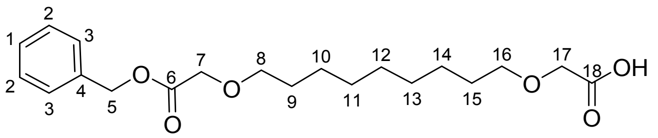

2-((9-(2-(benzyloxy)-2-oxoethoxy)nonyl)oxy)acetic acid (44b): Following general method D, **44b** was obtained from **43b** (1.047 g, 3.79 mmol) and benzyl bromide (0.45 mL, 3.79 mmol). The crude product was purified by column chromatography (0-50% EtOAc in hexane) to afford **44b** (0.463 g, 1.24 mmol, 33% yield) as a clear pale yellow oil. ^{1}H NMR (400 MHz, $CDCl_3$) δ_H ppm 9.71 (br s, 1 H, 18-CO_2H), 7.36 (s, 5 H, (1-3)-CH), 5.20 (s, 2 H, 5-CH_2), 4.12 (s, 2 H, 7-CH_2), 4.11 (s, 2 H, 17-CH_2), 3.56 (t, *J*=6.7 Hz, 2 H, 16-CH_2), 3.53 (t, *J*=6.7 Hz, 2 H, 8-CH_2), 1.62 (sxt, *J*=6.7 Hz, 4 H, 9,15-CH_2), 1.28 - 1.39 (m, 10 H, (10-14)-CH_2). ^{13}C NMR (101 MHz, $CDCl_3$) δ_C ppm 174.0 (C18), 170.5 (C6), 135.4 (C4), 128.6 (C2), 128.4 (C1), 128.4 (C3), 72.1 (C16), 72.0 (C8), 68.2 (C7), 67.7 (C17), 66.5 (C5), 29.5 (C9), 29.4 (C16), 29.3 (C11/12/13), 29.2 (C11/12/13), 29.2 (C11/12/13), 25.9 (C10), 25.8 (C14). HRMS (ESI) m/z: $[M+H]^+$ calculated for $C_{20}H_{31}O_6$: 367.2121, found 367.2121.

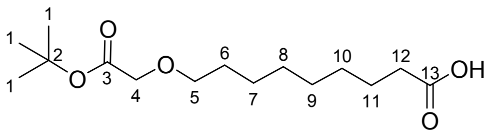

9-(2-(tert-butoxy)-2-oxoethoxy)nonanoic acid (45): Pyridinium dichromate (685.5 mg, 1.822 mmol) was added portionwise to a solution of **42** (100.0 mg, 0.364 mmol) in dry DMF (2 mL) and stirred at room temperature for 6 hours. The reaction mixture was diluted with 10% citric acid solution (30 mL) and extracted with EtOAc (4 x 20 mL). The combined organic layers were washed with sat. NaCl (3 x 30 mL), dried over Na_2SO_4, filtered and concentrated *in vacuo* to afford **45** (109.8 mg, 0.361 mmol, 99% yield) as a pale yellow oil. ^{1}H NMR (400 MHz, $CDCl_3$) δ_H 9.37 (br s, 1 H, 13-CO_2H), 3.95 (s, 2 H, 4-CH_2), 3.51 (t, *J*=6.6 Hz, 2 H, 5-CH_2), 2.36 (t, *J*=7.4 Hz, 2 H, 12-CH_2), 1.58 - 1.69 (m, 4 H, 6,11-CH_2), 1.49 (s, 9 H, 1-CH_3), 1.30 - 1.39 (m, 8 H, (7-10)-CH_2). ^{13}C NMR (101 MHz, $CDCl_3$) δ_C ppm 179.4 (C13), 169.9 (C3), 81.4 (C2), 71.7 (C5), 68.7 (C4), 33.9 (C12), 29.5 (C6), 29.2 (C8/9), 29.1 (C8/9), 28.9 (C10), 28.1 (C1), 25.9 (C7), 24.6 (C11). HRMS (ESI – ve, Direct Infusion) m/z: $[M-H]^-$ calculated for $C_{15}H_{27}O_5$: 287.1858, found 287.1862.

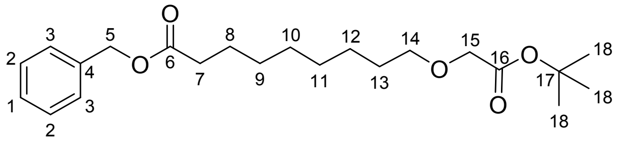

Benzyl 9-(2-(tert-butoxy)-2-oxoethoxy)nonanoate (46): To a solution of **45** (215.3 mg, 0.747 mmol) in 1,4-dioxane/DMF (1:1, 8 mL), was added benzyl bromide (0.10 mL, 0.821 mmol) followed by the addition of NEt_3 (0.12 mL, 0.885 mmol), then the resultant mixture was stirred at room temperature for 5 hours. The reaction mixture was concentrated *in vacuo* to afford a yellow oil, then the crude product was purified by column chromatography (0-40% EtOAc in hexane) to afford **46** (159.9 mg, 0.418 mmol, 56% yield) as a clear pale yellow oil. ^{1}H NMR (400 MHz, $CDCl_3$) δ_H ppm 7.31 - 7.39 (m, 5 H, (1-3)-CH), 5.12 (s, 2 H, 5-CH_2), 3.95 (s, 2 H, 15-CH_2), 3.50 (t, *J*=6.7 Hz, 2 H, 14-CH_2), 2.35 (t, *J*=7.6 Hz, 2 H, 7-CH_2), 1.56 - 1.67 (m, 4 H, 8,13-CH_2), 1.49 (s, 9 H, 18-CH_3), 1.27 - 1.39 (m, 8 H, (9-12)-CH_2). ^{13}C NMR (101 MHz, $CDCl_3$) δ_C ppm 173.6 (C6), 169.8 (C16), 136.1 (C4), 128.5 (C2), 128.2 (C3), 128.1 (C1), 81.4 (C17), 71.7 (C14), 68.8 (C15), 66.0 (C5), 34.3 (C7), 29.6 (C13), 29.2 (alkyl CH_2), 29.1 (alkyl CH_2), 29.0 (alkyl CH_2), 28.1 (C18), 25.9 (C9), 24.9 (C8). HRMS (ESI) m/z: $[M+Na]^+$ calculated for $C_{22}H_{34}O_5Na$: 401.2304, found 401.2303.

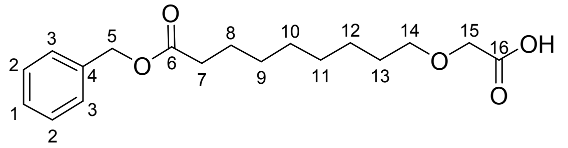

2-((9-(benzyloxy)-9-oxononyl)oxy)acetic acid (47): TFA (1 mL) was added to a stirring solution of **46** (159.9 mg, 0.422) in DCM (2 mL) and the resulting reaction mixture stirred at room temperature for 4 hours. The reaction mixture was concentrated *in vacuo* to afford **47** (132.5 mg, 0.407 mmol, 98% yield) as a clear colourless oil. ^{1}H NMR (400 MHz, $CDCl_3$) δ_H ppm 7.31 - 7.39 (m, 5 H, (1-3)-CH), 5.12 (s, 2 H, 5-CH_2), 4.11 (s, 2 H, 15-CH_2), 3.56 (t, *J*=6.6 Hz, 2 H, 14-CH_2), 2.36 (t, *J*=7.5 Hz, 2 H, 7-CH_2), 1.59 - 1.69 (m, 4 H, 8,13-CH_2), 1.27 - 1.39 (m, 8 H, (9-12)-CH_2). ^{13}C NMR (101 MHz, $CDCl_3$) δ_C ppm 173.9 (C16), 173.7 (C6), 136.1 (C4), 128.5 (C2), 128.1 (C1,C3), 72.1 (C14), 67.7 (C15), 66.1 (C5), 34.3 (C7), 29.3 (C13), 29.1 (alkyl CH_2), 29.05 (alkyl CH_2), 29.0 (alkyl CH_2), 25.8 (C12), 24.8 (C8). HRMS (ESI) m/z: $[M+H]^+$ calculated for $C_{18}H_{27}O_5$: 323.1858, found 323.1862.

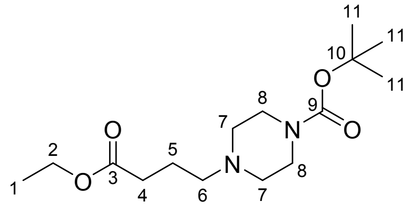

Tert-butyl 4-(4-ethoxy-4-oxobutyl)piperazine-1-carboxylate (48): To a solution of tert-butyl piperazine-1-carboxylate (2.00 g, 10.74 mmol) and NEt_3 (1.80 mL, 12.89 mmol) in dry THF (40 mL), ethyl 4-bromobutanoate (1.61 mL, 11.27 mmol) was added and the resultant solution stirred at room temperature overnight. EtOAc (40 mL) and 10% $NaHCO_3$ (40 mL) were added, the phases separated and the organic layer collected. The aqueous layer was then extrated with EtOAc (30 mL) and the organic extracts combined, washed with water (50 mL) and sat. NaCl (50 mL), dried over Na_2SO_4, filtered and the filtrate concentrated *in vacuo* to afford a cloudy white slurry (3.45 g). The crude product was purified by column chromatography (1-8% MeOH in DCM) to afford **48** (1.55 g, 5.06 mmol, 47% yield) as a clear colourless oil. ^{1}H NMR (400 MHz, $CDCl_3$) δ_H ppm 4.11 (q, *J*=7.2 Hz, 2 H, 2-CH_2), 3.39 - 3.50 (m, 4 H, 8-CH_2), 2.38 - 2.48 (m, 6 H, 6,7-CH_2), 2.33 (t, *J*=7.3 Hz, 2 H, 4-CH_2), 1.84 (quin, *J*=7.3 Hz, 2 H, 5-CH_2), 1.44 (s, 9 H, 11-CH_3), 1.24 (t, *J*=7.2 Hz, 3

H, 1-CH_3). ^{13}C NMR (101 MHz, $CDCl_3$) δ_C ppm 173.3 (C3), 154.6 (C9), 79.7 (C10), 60.3 (C2), 57.5 (C6), 52.7 (C7), 43.2 (C8), 32.0 (C4), 28.3 (C11), 21.6 (C5), 14.2 (C1). HRMS (ESI) m/z: $[M+H]^+$ calculated for $C_{15}H_{29}N_2O_4$: 301.2127, found 301.2123.

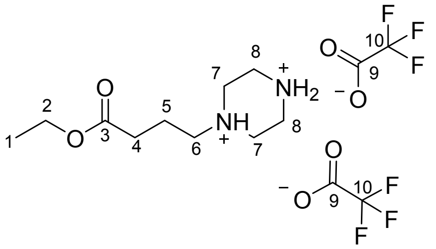

1-(4-ethoxy-4-oxobutyl)piperazine-1,4-diium 2,2,2-trifluoroacetate (49): TFA (3 mL) was added to a stirring solution of **48** (1.53 g, 5.10 mmol) in DCM (3 mL) and the resulting reaction mixture stirred at room temperature overnight. The reaction mixture was concentrated *in vacuo*, then the residue suspended in Et_2O (30 mL), filtered, then the solid washed with additional Et_2O and dried to afford **49** (1.43 g, 3.35 mmol, 66% yield) as an off-white solid. ^{1}H NMR (400 MHz, CD_3OD) δ_H ppm 4.15 (q, J=7.1 Hz, 2 H, 2-CH_2), 3.49 – 3.56 (m, 4 H, 7-CH_2), 3.38 – 3.46 (m, 4 H, 8-CH_2), 3.09 – 3.15 (m, 2 H, 6-CH_2), 2.47 (t, J=7.0 Hz, 2 H, 4-CH_2), 1.97 – 2.06 (m, 2 H, 5-CH_2), 1.26 (t, J=7.1 Hz, 3 H, 1-CH_3). ^{13}C NMR (101 MHz, CD_3OD) δ_C ppm 174.3 (C3), 162.6 (q, J_{CF}=35.9 Hz, C9) 117.9 (q, J_{CF}=291.8 Hz, C10), 62.0 (C2), 57.8 (C6), 50.1 (C7), 42.8 (C8), 31.8 (C4), 20.9 (C5), 14.6 (C1). ^{19}F NMR (376 MHz, CD_3OD) δ_F ppm -77.2. HRMS (ESI) m/z: $[M+H]^+$ calculated for $C_{10}H_{21}N_2O_2$: 201.1603, found 201.1602.

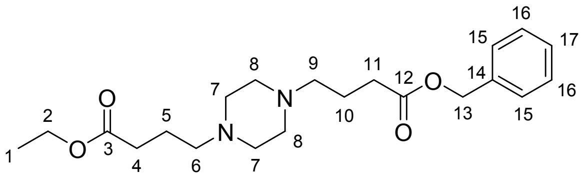

Benzyl 4-(4-(4-ethoxy-4-oxobutyl)piperazin-1-yl)butanoate (50): To a solution of **49** (500.0 mg, 1.170 mmol) in MeCN (10 mL), benzyl 4-bromobutanoate (361.0 mg, 1.404 mmol) and Na_2CO_3 (372.1 mg, 3.51 mmol) were added and the resultant suspension heated at 60 ^{0}C overnight. The reaction mixture was filtered and the filtrate concentrated *in vacuo* to afford a cloudy yellow oil (0.816 g). The crude product was purified by column chromatography (dry load, 1-8% MeOH in DCM) to afford **50** (363.7 mg, 0.966 mmol, 83% yield) as a clear yellow oil. ^{1}H NMR (400 MHz, $CDCl_3$) δ_H ppm 7.30 - 7.38 (m, 5 H, (15-17)-CH), 5.11 (s, 2 H, 13-CH_2), 4.12 (q, J=7.2 Hz, 2 H, 2-CH_2), 2.42 - 2.60 (m, 8 H, 7-CH_2,8-CH_2), 2.35 - 2.41 (m, 6 H, (6,9,11)-CH_2), 2.32 (t, J=7.4 Hz, 2 H, 4-CH_2), 1.78 - 1.88 (m, 4 H, 5-CH_2,10-CH_2), 1.25 (t, J=7.2 Hz, 3 H, 1-CH_3). ^{13}C NMR (101 MHz, $CDCl_3$) δ_C ppm 173.4 (C3), 173.2 (C12), 136.0 (C14), 128.5 (C16), 128.2 (C17), 128.1 (C15), 66.1 (C13), 60.2 (C2), 57.5 (C6/9), 57.4 (C6/9), 52.9 (C7/8), 52.8 (C7/8), 32.2 (C4/11), 32.2 (C4/11), 22.0 (C5,C10), 14.2 (C1). HRMS (ESI) m/z: $[M+H]^+$ calculated for $C_{21}H_{33}N_2O_4$: 377.2440, found 377.2440.

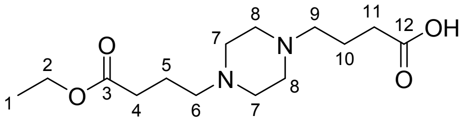

4-(4-(4-ethoxy-4-oxobutyl)piperazin-1-yl)butanoic acid (51): To a solution of **50** (341.5, 0.907 mmol) in EtOH (8 mL), 10% Pd/C (35.0 mg) was added. The reaction flask was filled with nitrogen and evacuated 3

times using a Shlenk line, before a balloon of hydrogen was added and the resultant mixture stirred vigorously overnight. The balloon of hydrogen was removed and the flask was flushed with nitrogen. The reaction mixture was filtered through a glass microfiber filter paper, and the filtrate concentrated *in vacuo* to afford **51** (250.0 mg, 0.873 mmol, 96% yield) as an off-white solid. ^{1}H NMR (400 MHz, DMSO-d_6) δ_H ppm 4.04 (q, *J*=7.2 Hz, 2 H, 2-CH_2), 2.29 - 2.47 (m, 10 H, (7,8,6)-CH_2), 2.19 - 2.29 (m, 6 H, (4,11,9)-CH_2), 1.60 - 1.70 (m, 4 H, 4,11-CH_2), 1.17 (t, *J*=7.2 Hz, 3 H, 1-CH_3). CO_2H not observed. ^{13}C NMR (101 MHz, DMSO-d_6) δ_C ppm 174.4 (C3), 172.8 (C1), 59.7 (C2), 57.0 (C6), 56.8 (C9), 52.5 (C7/8), 52.3 (C7/8), 32.2 (C4), 31.5 (C11), 21.6 (C5/10), 21.4 (C5/10), 14.1 (C1). HRMS (ESI) m/z: $[M+H]^+$ calculated for $C_{14}H_{27}N_2O_4$: 287.1971, found 287.1970.

5.3. Preparation of Compounds **1-20**

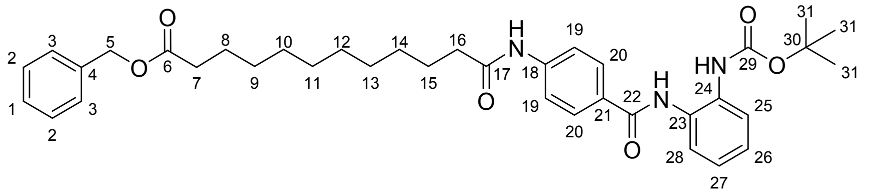

Benzyl 12-((4-((2-((tert-butoxycarbonyl)amino)phenyl)carbamoyl)phenyl)amino)-12-oxododeca-noate (52a): Following general method E, **52a** was obtained from **37d** (257 mg, 0.802 mmol) and **35a** (200 mg, 0.611 mmol). The crude product was purified by column chromatography (50% EtOAc in hexane) to give **52a** (274 mg, 0.430 mmol, 70% yield) as a pale yellow solid. ^{1}H NMR (400 MHz, $CDCl_3$) δ_H ppm 9.20 (br s, 1 H, 23-NH), 7.89 (d, *J*=8.7 Hz, 2 H, 20-CH), 7.78 (br s, 1 H, 18-NH), 7.73 (dd, *J*=7.7, 1.6 Hz, 1 H, 28-CH), 7.60 (d, *J*=8.7 Hz, 2 H, 19-CH), 7.31 - 7.40 (m, 5 H, 1,2,3-CH), 7.28 (dd, *J*=7.7, 1.6 Hz, 1 H, 25-CH), 7.11 - 7.22 (m, 2 H, 26-CH,27-CH), 7.01 (s, 1 H, 24-NH), 5.12 (s, 2 H, 5-CH_2), 2.30 - 2.40 (m, 4 H, 7-CH_2,16-CH_2), 1.68 - 1.74 (m, 2 H, 15-CH_2), 1.60 - 1.68 (m, 2 H, 8-CH_2), 1.51 (s, 9 H, 31-CH_3), 1.24 - 1.37 (m, 12 H, (9-14)-CH_2). ^{13}C NMR (101 MHz, $CDCl_3$) δ_C ppm 173.8 (C6), 171.9 (C17), 165.2 (C22), 154.6 (C29), 141.5 (C18), 136.0 (C4), 130.8 (C23), 130.1 (C24), 129.2 (C21), 128.5 (C2), 128.4 (C20), 128.2 (C1), 128.1 (C3), 125.9 (C27), 125.8 (C26), 125.7 (C28), 124.5 (C25), 119.1 (C19), 81.3 (C30), 66.1 (C5), 37.7 (C16), 34.3 (C7), 29.3 (2x alkyl CH_2), 29.25 (alkyl CH_2), 29.2 (alkyl CH_2), 29.1 (alkyl CH_2), 29.0 (alkyl CH_2), 28.3 (C31), 25.4 (C15), 24.9 (C8). HRMS (ESI) m/z: $[M+Na]^+$ calculated for $C_{37}H_{47}N_3O_6Na$: 652.3363, found 652.3364.

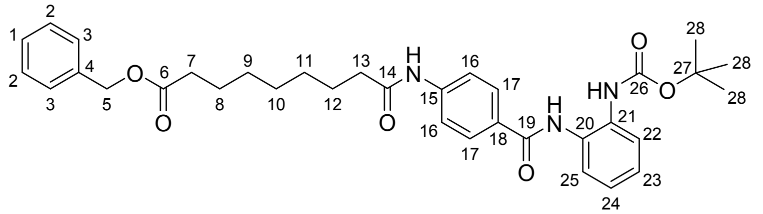

Benzyl 9-((4-((2-((tert-butoxycarbonyl)amino)phenyl)carbamoyl)phenyl)amino)-9-oxononanoate (52b): Following general method E, **52b** was obtained from **37a** (0.475 g, 1.71 mmol) and **35a** (0.430 g, 1.31 mmol). The crude product was purified by column chromatography (0-50% EtOAc in hexane) to give **52b** (0.540 g, 0.94 mmol, 53% yield) as a yellow tar. ^{1}H NMR (400 MHz, $CDCl_3$) δ_H ppm 9.21 (br s, 1 H, 20-NH), 7.89 (d,

J=8.7 Hz, 2 H, 17-CH), 7.84 (s, 1 H, 15-NH), 7.73 (dd, *J*=7.7, 1.6 Hz, 1 H, 25-CH), 7.61 (d, *J*=8.7 Hz, 2 H, 19-CH), 7.30 - 7.40 (m, 5 H, 1,2,3-CH), 7.27 (s, 1 H, 22-CH), 7.12 - 7.21 (m, 2 H, 23,24-CH), 7.01 (s, 1 H, 21-NH), 5.12 (s, 2 H, 5-CH_2), 2.28 - 2.42 (m, 4 H, 7,13-CH), 1.67 - 1.73 (m, 2 H, 12-CH_2), 1.61 - 1.67 (m, 2 H, 8-CH_2), 1.51 (s, 9 H, 28-CH_3), 1.29 - 1.37 (m, 6 H, (9-11)-CH_2). ^{13}C NMR (101 MHz, $CDCl_3$) δ_C ppm 173.8 (C6), 171.9 (C14), 165.2 (C19), 154.6 (C26), 141.5 (C15), 136.0 (C4), 130.8 (C20), 130.1 (C21), 129.2 (C18), 128.5 (C2), 128.4 (C17), 128.2 (C1), 128.1 (C3), 125.9 (C24), 125.8 (C25), 125.7 (C23), 124.4 (C22), 119.1 (C16), 81.3 (C27), 66.1 (C5), 37.6 (C13), 34.2 (C7), 28.9 (CH_2), 28.85 (CH_2), 28.8 (CH_2), 28.3 (C28), 25.2 (C12), 24.8 (C8). HRMS (ESI) m/z: $[M+H]^+$ calculated for $C_{34}H_{42}N_3O_6$: 588.3074, found 588.3067.

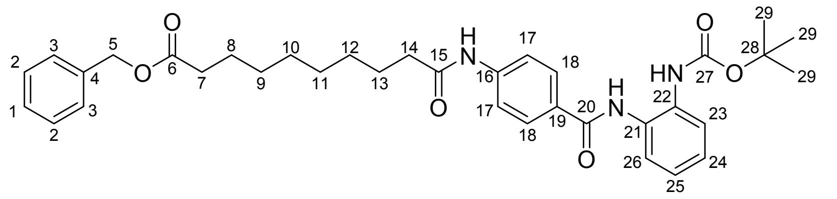

Benzyl 10-((4-((2-((tert-butoxycarbonyl)amino)phenyl)carbamoyl)phenyl)amino)-10-oxodecanoate (52c): Following general method E, **52c** was obtained from **37b** (0.348 g, 1.190 mmol) and **35a** (0.300 g, 0.916 mmol). The crude product was purified by column chromatography (10-100% EtOAc in hexane) to give **52c** (0.359 g, 0.597 mmol, 65% yield) as a pale yellow solid. ^{1}H NMR (400 MHz, $CDCl_3$) δ_H ppm 9.27 (s, 1 H, 21-NH), 8.06 (s, 1 H16-NH), 7.87 (d, *J*=8.7 Hz, 2 H, 18-CH), 7.69 (dd, *J*=7.7, 1.8 Hz, 1 H, 26-CH), 7.59 (d, *J*=8.7 Hz, 2 H, 17-CH), 7.31 - 7.40 (m, 5 H, (1-3)-CH), 7.29 (dd, *J*=7.7, 1.8 Hz, 1 H, 23-CH), 7.09 - 7.18 (m, 3 H, 22-NH,24-CH,25-CH), 5.12 (s, 2 H, 5-CH_2), 2.29 - 2.39 (m, 4 H, 7-CH_2,14-CH_2), 1.60 - 1.70 (m, 4 H, 8-CH_2,13-CH_2), 1.50 (s, 9 H, 29-CH_3), 1.26 - 1.34 (m, 8 H, (9-12)-CH_2). ^{13}C NMR (101 MHz, $CDCl_3$) δ_C ppm 173.8 (C6), 172.0 (C15), 165.3 (C20), 154.6 (C27), 141.6 (C16), 136.0 (C4), 130.6 (C21), 130.3 (C22), 129.0 (C19), 128.5 (C2), 128.4 (C18), 128.2 (C1), 128.1 (C3), 126.0 (C25), 125.8 (C24), 125.6 (C26), 124.4 (C23), 119.1 (C17), 81.2 (C28), 66.1 (C5), 37.5 (C14), 34.2 (C7), 29.1 (alkyl CH_2), 29.0 (alkyl CH_2), 29.0 (alkyl CH_2), 28.9 (alkyl CH_2), 28.2 (C29), 25.3 (C13), 24.8 (C8). HRMS (ESI) m/z: $[M+H]^+$ calculated for $C_{35}H_{44}N_3O_4$: 602.3230, found 602.3231.

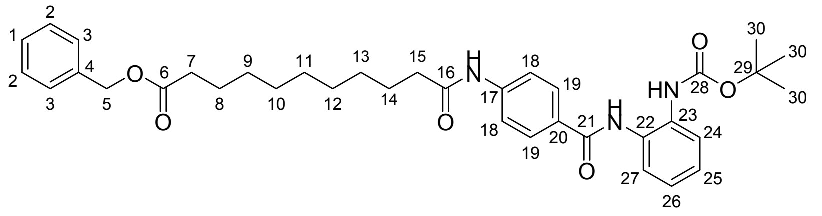

Benzyl 11-((4-((2-((tert-butoxycarbonyl)amino)phenyl)carbamoyl)phenyl)amino)-11-oxoundecanoate (52d): Following general method E, **52d** was obtained from **37c** (0.365 g, 1.191 mmol) and **35a** (0.300 g, 0.916 mmol). The crude product was purified by column chromatography (10-100% EtOAc in hexane) to give **52d** (0.402 g, 0.653 mmol, 71% yield) as a pale yellow solid. ^{1}H NMR (400 MHz, $CDCl_3$) δ_H ppm 9.10 (br s, 1 H, 22-NH), 7.94 (d, *J*=8.7 Hz, 2 H, 19-CH), 7.82 (dd, *J*=7.7, 1.8 Hz, 1 H, 27-CH), 7.65 (d, *J*=8.7 Hz, 2 H, 18-CH), 7.34 - 7.40 (m, 5 H, (1-3)-CH), 7.32 (dd, *J*=7.7, 1.8 Hz, 1 H, 24-CH), 7.25 (s, 1 H, 17-NH), 7.15 - 7.25 (m, 2 H, 25-CH,26-CH), 6.73 (s, 1 H, 23-NH), 5.13 (s, 2 H, 5-CH_2), 2.33 - 2.42 (m, 4 H, 7-CH_2,15-CH_2), 1.70 - 1.79 (m, 2 H, 14-CH_2), 1.61 - 1.69 (m, 2 H, 8-CH_2), 1.53 (s, 9 H, 30-CH_3), 1.29 - 1.39 (m, 10 H, (9-13)-

CH_2). ^{13}C NMR (101 MHz, $CDCl_3$) δ ppm 173.7 (C6), 171.8 (C16), 165.2 (C21), 154.6 (C28), 141.2 (C17), 136.1 (C4), 130.7 (C22), 130.1 (C23), 129.2 (C20), 128.5 (C2), 128.4 (C19), 128.2 (C1), 128.1 (C3), 126.0 (C26), 125.9 (C25), 125.7 (C27), 124.5 (C24), 119.0 (C18), 81.5 (C29), 66.1 (C5), 37.8 (C15), 34.3 (C7), 29.15 (2 x alkyl CH_2), 29.1 (alkyl CH_2), 29.05 (alkyl CH_2), 29.0 (alkyl CH_2), 28.3 (C30), 25.3 (C14), 24.9 (C8). HRMS (ESI) m/z: $[M+H]^+$ calculated for $C_{36}H_{46}N_3O_4$: 616.3387, found 616.3391.

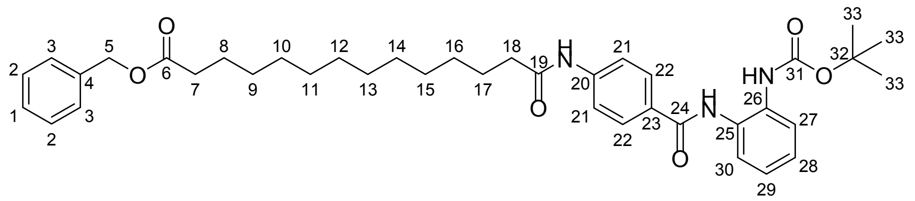

Benzyl 14-((4-((2-((tert-butoxycarbonyl)amino)phenyl)carbamoyl)phenyl)amino)-14-oxotetradecanoate (52e): Following general method E, **52e** was obtained from **37e** (421.4 mg, 1.21 mmol) and **35a** (300.0 mg, 0.92 mmol). The crude product was purified by column chromatography (0-50% EtOAc in hexane) to give **52e** (372.4 mg, 0.56 mmol, 61% yield) as a yellow solid. ^{1}H NMR (400 MHz, $CDCl_3$) δ_H ppm 9.20 (br s, 1 H, 25-NH), 7.89 (d, *J*=8.6 Hz, 2 H, 22-CH), 7.68 - 7.77 (m, 2 H, 20-NH,30-CH), 7.60 (d, *J*=8.6 Hz, 2 H, 21-CH), 7.30 - 7.40 (m, 5 H, (1-3)-CH), 7.27 - 7.30 (m, 1 H, 27-CH), 7.11 - 7.22 (m, 2 H, 28,29-CH), 6.99 (s, 1 H, 26-NH), 5.12 (s, 2 H, 5-CH_2), 2.32 - 2.40 (m, 4 H, 7,18-CH_2), 1.68 - 1.75 (m, 2 H, 17-CH_2), 1.61 - 1.68 (m, 2 H, 8-CH_2), 1.51 (s, 9 H, 33-CH_3), 1.25 - 1.38 (m, 16 H, (9-16)-CH_2). ^{13}C NMR (101 MHz, $CDCl_3$) δ_C ppm 173.8 (C6), 171.8 (C19), 165.1 (C24), 154.6 (C31), 141.4 (C20), 136.1 (C4), 130.8 (C25), 130.1 (C26), 129.3 (C23), 128.5 (C2), 128.4 (C22), 128.15 (C1), 128.1 (C3), 125.9 (C29), 125.8 (C28), 125.75 (C30), 124.5 (C27), 119.1 (C21), 81.3 (C32), 66.1 (C5), 37.7 (C18), 34.3 (C7), 29.5 (alkyl CH_2), 29.45 (alkyl CH_2), 29.4 (alkyl CH_2), 29.35 (alkyl CH_2), 29.3 (alkyl CH_2), 29.2 (alkyl CH_2), 29.15 (alkyl CH_2), 29.1 (alkyl CH_2), 28.3 (C33), 25.4 (C17), 24.9 (C8). HRMS (ESI) m/z: $[M+H]^+$ calculated for $C_{39}H_{52}N_3O_6$: 658.3856, found 658.3880.

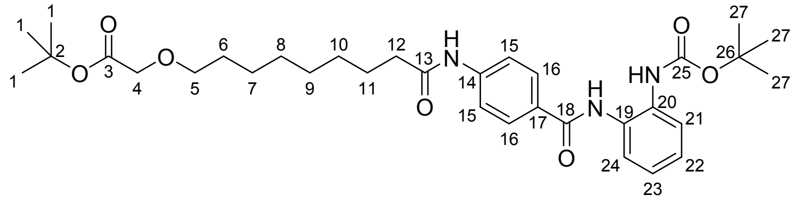

Tert-butyl 2-((9-((4-((2-((tert-butoxycarbonyl)amino)phenyl)carbamoyl)phenyl)amino)-9-oxononyl)oxy)acetate (52f): Following general method E, **52f** was obtained from **45** (85.0 mg, 0.295 mmol) and **35a** (88.4 mg, 0.270 mmol). The crude product was purified by column chromatography (0-100% EtOAc in hexane) to afford **52f** (66.3 mg, 0.110 mmol, 41% yield) as a yellow tar. ^{1}H NMR (400 MHz, $CDCl_3$) δ_H ppm 9.24 (br s, 1 H, 19-NH), 8.14 (s, 1 H, 14-NH), 7.88 (d, *J*=8.7 Hz, 2 H, 16-CH), 7.70 (dd, *J*=7.5, 1.7 Hz, 1 H, 24-CH), 7.62 (d, *J*=8.7 Hz, 2 H, 15-CH), 7.30 (dd, *J*=7.5, 1.7 Hz, 1 H, 21-CH), 7.12 - 7.19 (m, 2 H, 22,23-CH), 7.11 (s, 1 H, 20-NH), 3.95 (s, 2 H, 4-CH_2), 3.49 (t, *J*=6.6 Hz, 2 H, 5-CH_2), 2.33 (t, *J*=7.6 Hz, 2 H, 12-CH_2), 1.65 - 1.71 (m, 2 H, 11-CH_2), 1.55 - 1.62 (m, 2 H, 6-CH_2), 1.50 (s, 9 H, 27-CH_3), 1.48 (s, 9 H, 1-CH_3), 1.26 - 1.35 (m, 8 H, (7-10)-CH_2). ^{13}C NMR (101 MHz, $CDCl_3$) δ_C ppm 172.1 (C13), 170.0 (C3), 165.3 (C18), 154.6 (C25), 141.7 (C14), 130.7 (C19), 130.3 (C20), 129.0 (C17), 128.4 (C16), 125.9 (C22/23), 125.8 (C24),

125.7 (C22/23), 124.4 (C21), 119.1 (C15), 81.6 (C1), 81.2 (C27), 71.7 (C5), 68.7 (C4), 37.5 (C12), 29.5 (C6), 29.2 (alkyl CH_2), 29.1 (alkyl CH_2), 29.1 (alkyl CH_2), 28.3 (C27), 28.1 (C1), 25.9 (C7), 25.3 (C11). HRMS (ESI) m/z: $[M+H]^+$ calculated for $C_{33}H_{48}N_3O_7$: 598.3492, found 598.3484.

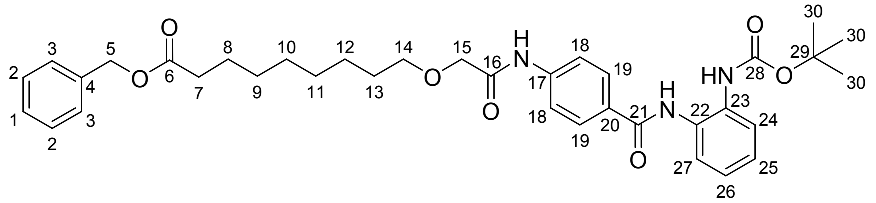

Benzyl 9-(2-((4-((2-((tert-butoxycarbonyl)amino)phenyl)carbamoyl)phenyl)amino)-2-oxoethoxy)nonanoate (52g): Following general method E, **52g** was obtained from **47** (130.0 mg, 0.403 mmol) and **35a** (110.0 mg, 0.336 mmol). The crude product was purified by column chromatography (0-100% EtOAc in hexane) to afford **52g** (157.4 mg, 0.247 mmol, 73% yield) as a colourless tar. ^{1}H NMR (400 MHz, $CDCl_3$) δ_H ppm 9.17 (br s, 1 H, 22-NH), 8.46 (br s, 1 H, 17-NH), 7.97 (d, *J*=8.7 Hz, 2 H, 19-CH), 7.78 (dd, *J*=7.8, 1.7 Hz, 1 H, 27-CH), 7.69 (d, *J*=8.7 Hz, 2 H, 18-CH), 7.30 - 7.40 (m, 5 H, (1-3)-CH), 7.27 (dd, *J*=7.8, 1.7 Hz, 1 H, 24-CH), 7.23 (app. td, *J*=7.8, 1.7 Hz, 1 H, 26-CH), 7.16 (app. td, *J*=7.8, 1.7 Hz, 1 H, 25-CH), 6.86 (s, 1 H, 23-NH), 5.12 (s, 2 H, 5-CH_2), 4.07 (s, 2 H, 15-CH_2), 3.60 (t, *J*=6.6 Hz, 2 H, 14-CH_2), 2.37 (t, *J*=7.5 Hz, 2 H, 7-CH_2), 1.63 - 1.72 (m, 4 H, 8,13-CH_2), 1.52 (s, 9 H, 30-CH_3), 1.32 - 1.43 (m, 8 H, (9-12)-CH_2). ^{13}C NMR (101 MHz, $CDCl_3$) δ_C ppm 173.6 (C6), 168.2 (C16), 164.9 (C21), 154.6 (C28), 140.3 (C17), 136.1 (C4), 130.9 (C22), 129.9 (C20,C23), 128.6 (C19), 128.5 (C2), 128.2 (C1), 128.1 (C3), 125.9 (C26), 125.9 (C25), 125.8 (C27), 124.4 (C24), 119.1 (C18), 81.4 (C29), 72.1 (C14), 70.2 (C15), 66.1 (C5), 34.3 (C7), 29.4 (C13), 29.1 (2x alkyl CH_2), 29.0 (alkyl CH_2), 28.3 (C30), 26.0 (C12), 24.9 (C8). HRMS (ESI) m/z: $[M+H]^+$ calculated for $C_{36}H_{46}N_3O_7$: 632.3336, found 632.3335.

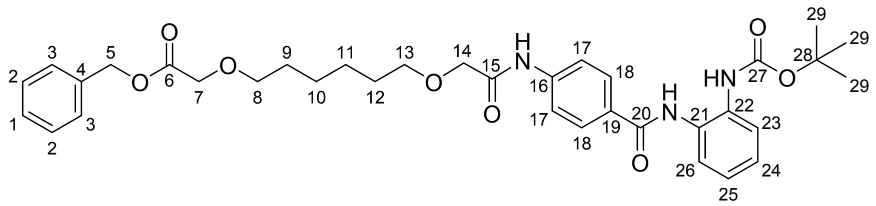

Benzyl 2-((6-(2-((4-((2-((tert-butoxycarbonyl)amino)phenyl)carbamoyl)phenyl)amino)-2-oxoethoxy)hexyl)oxy)acetate (52h): Following general method E, **52h** was obtained from **44a** (78.2 mg, 0.241 mmol) and **35a** (71.8 mg, 0.219 mmol). The crude product was purified by column chromatography (0-100% EtOAc in hexane) to afford **52h** (76.5 mg, 0.120 mmol, 55% yield) as a pale yellow oil. ^{1}H NMR (400 MHz, $CDCl_3$) δ_H ppm 9.27 (br s, 1 H, 21-NH), 8.48 (br s, 1 H, 16-NH), 7.95 (d, *J*=8.7 Hz, 2 H, 18-CH), 7.70 (dd, *J*=7.9, 1.7 Hz, 1 H, 26-CH), 7.67 (d, *J*=8.7 Hz, 2 H, 17-CH), 7.31 - 7.39 (m, 5 H, (1-3)-CH), 7.29 (dd, *J*=7.7, 1.7 Hz, 1 H, 23-CH), 7.11 - 7.20 (m, 2 H, 24,25-CH_2), 7.10 (br s, 1 H, 22-NH), 5.18 (s, 2 H, 5-CH_2), 4.12 (s, 2 H, 7-CH_2), 4.05 (s, 2 H, 14-CH_2), 3.59 (t, *J*=6.6 Hz, 2 H, 13-CH_2), 3.55 (t, *J*=6.5 Hz, 2 H, 8-CH_2), 1.62 - 1.71 (m, 4 H, 9,12-CH_2), 1.51 (s, 9 H, 29-CH_3), 1.41 - 1.48 (m, 4 H, 10,11-CH_2). ^{13}C NMR (101 MHz, $CDCl_3$) δ_C ppm 170.4 (C6), 168.2 (C15), 165.0 (C20), 154.5 (C27), 140.3 (C16), 135.4 (C4), 130.6 (C21), 130.2 (C22), 129.8 (C19), 128.6 (C2), 128.5 (C18), 128.4 (C1), 128.3 (C3), 125.9 (C24), 125.7 (C26), 125.6

(C25), 124.4 (C23), 119.1 (C17), 81.1 (C28), 71.9 (C13), 71.7 (C8), 70.1 (C14), 68.2 (C7), 66.4 (C5), 29.4 (C9), 29.3 (C12), 28.2 (C29), 25.8 (C11), 25.8 (C10). HRMS (ESI) m/z: $[M+H]^+$ calculated for $C_{35}H_{44}N_3O_8$: 634.3128, found 634.3125.

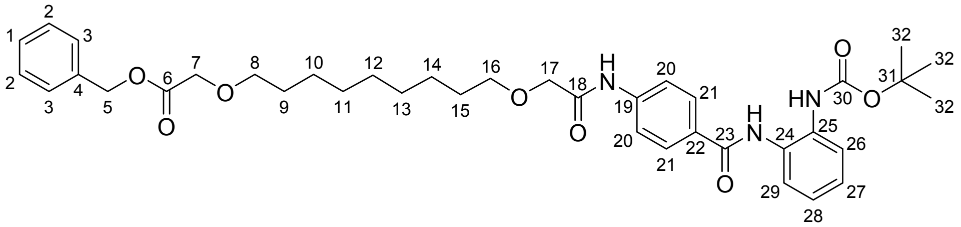

Benzyl 2-((9-(2-((4-((2-((tert-butoxycarbonyl)amino)phenyl)carbamoyl)phenyl)amino)-2-oxoethoxy)nonyl)oxy)acetate (52i): Following general method E, **52i** was obtained from **44b** (145.0 mg, 0.396 mmol) and **35a** (99.6 mg, 0.304 mmol). The crude product was purified by column chromatography (0-70% EtOAc in hexane) to afford **52i** (157.8 mg, 0.229 mmol, 75% yield) as a colourless tar. ^{1}H NMR (400 MHz, $CDCl_3$) δ_H ppm 9.17 (br s, 1 H, 24-NH), 8.47 (br s, 1 H, 19-NH), 7.97 (d, *J*=8.6 Hz, 2 H, 21-CH), 7.77 (dd, *J*=7.8, 1.6 Hz, 1 H, 29-CH), 7.69 (d, *J*=8.7 Hz, 2 H, 20-CH), 7.32 - 7.39 (m, 5 H, (1-3)-CH), 7.29 (dd, *J*=7.8, 1.6 Hz, 1 H, 26-CH), 7.22 (td, *J*=7.8, 1.6 Hz, 1 H, 28-CH), 7.16 (td, *J*=7.8, 1.6 Hz, 1 H, 27-CH), 6.88 (s, 1 H, 25-NH), 5.19 (s, 2 H, 5-CH_2), 4.11 (s, 2 H, 7-CH_2), 4.07 (s, 2 H, 17-CH_2), 3.61 (t, *J*=6.6 Hz, 2 H, 16-CH_2), 3.53 (t, *J*=6.6 Hz, 2 H, 8-CH_2), 1.65 - 1.73 (m, 2 H, 15-CH_2), 1.58 - 1.64 (m, 2 H, 9-CH_2), 1.52 (s, 9 H, 32-CH_3), 1.30 - 1.43 (m, 10 H, (10-14)-CH_2). ^{13}C NMR (101 MHz, $CDCl_3$) δ_C ppm 170.5 (C6), 168.2 (C18), 164.9 (C23), 154.6 (C30), 140.3 (C19), 135.4 (C4), 130.9 (C24), 130.0 (C25), 129.9 (C22), 128.6 (C2,C21), 128.4 (C1), 128.3 (C3), 125.9 (C27,C28), 125.8 (C29), 124.4 (C26), 119.1 (C20), 81.4 (C31), 72.1 (C16), 72.0 (C8), 70.2 (C17), 68.3 (C7), 66.5 (C5), 29.5 (alkyl CH_2), 29.4 (alkyl CH_2), 29.4 (alkyl CH_2), 29.3 (alkyl CH_2), 29.3 (alkyl CH_2), 28.3 (C32), 26.0 (C10/14), 25.9 (C10/14). HRMS (ESI) m/z: $[M+H]^+$ calculated for $C_{38}H_{50}N_3O_8$: 676.3598, found 676.3605.

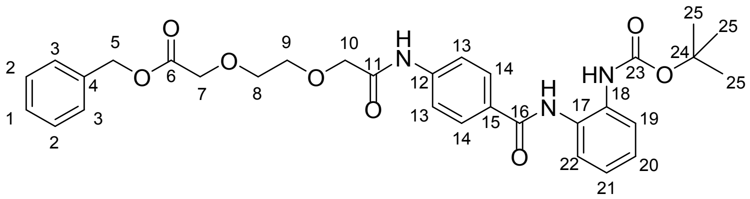

Benzyl 2-(2-(2-((4-((2-((tert-butoxycarbonyl)amino)phenyl)carbamoyl)phenyl)amino)-2-oxoethoxy)ethoxy)acetate (52j): Following general method E, **52j** was obtained from **39a** (234.4 mg, 0.87 mmol) and **35a** (220.0 mg, 0.67 mmol). The crude product was purified by column chromatography (0-100% EtOAc in hexane) to afford **52j** (295.2 mg, 0.51 mmol, 58% yield) as a white solid. ^{1}H NMR (400 MHz, $CDCl_3$) δ_H ppm 9.14 (br s, 1 H, 17-NH) 8.99 (s, 1 H, 12-NH) 7.93 (d, *J*=8.8 Hz, 2 H, 14-CH) 7.69 - 7.78 (m, 3 H, 13-CH,19-CH) 7.36 (s, 5 H, (1-3)-CH) 7.30 (dd, *J*=7.7, 1.6 Hz, 1 H, 23-CH) 7.21 (app. td, *J*=7.7, 1.6 Hz, 1 H, 21-CH) 7.15 (app. td, *J*=7.7, 1.6 Hz, 1 H, 20-CH) 6.93 (s, 1 H, 18-NH) 5.22 (s, 2 H, 5-CH_2) 4.24 (s, 2 H, 7-CH_2) 4.12 (s, 2 H, 10-CH_2) 3.77 - 3.84 (m, 4 H, 8-CH_2,9-CH_2) 1.51 (s, 9 H, 25-CH_3). ^{13}C NMR (101 MHz, $CDCl_3$) δ_C ppm 170.1 (C6) 168.4 (C11) 165.1 (C16) 154.5 (C23) 140.7 (C12) 135.1 (C4) 130.8 (C18) 130.1 (C17) 129.7 (C15) 128.7 (C2) 128.6 (C1) 128.45 (C3) 128.4 (C14) 125.9 (C20) 125.85 (C21) 125.8 (C22) 124.4 (C19)

119.3 (C13) 81.3 (C24) 71.0 (C8) 70.8 (C9) 70.5 (C10) 68.5 (C7) 66.9 (C5) 28.3 (C25). HRMS (ESI) m/z: $[M+H]^+$ calculated for $C_{31}H_{36}N_3O_8$: 578.2502, found 578.2499.

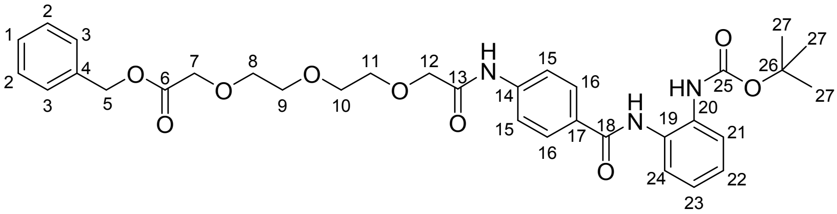

Benzyl 2-(2-(2-(2-((4-((2-((tert-butoxycarbonyl)amino)phenyl)carbamoyl)phenyl)amino)-2-oxoethoxy)ethoxy)ethoxy)acetate (52k): Following general method E, **52k** was obtained from **39b** (204.1 mg, 0.653 mmol) and **35a** (178.3 mg, 0.545 mmol). The crude product was purified by column chromatography (0-100% EtOAc in hexane, product eluted at 90% EtOAc) to afford **52k** (225.5 mg, 0.345 mmol, 63% yield) as a clear pale yellow oil. ^{1}H NMR (400 MHz, $CDCl_3$) δ_H ppm 9.18 (br s, 1 H, 19-NH), 8.85 (s, 1 H, 14-NH), 7.89 (d, *J*=8.7 Hz, 2 H, 16-CH), 7.64 - 7.71 (m, 3 H, 15-CH, 21-CH), 7.29 - 7.35 (m, 6 H, (1-3)-CH, 24-CH), 7.08 - 7.16 (m, 3 H, 22-CH,23-CH,20-NH), 5.16 (s, 2 H, 5-CH_2), 4.15 (s, 2 H, 7-CH_2), 4.10 (s, 2 H, 12-CH_2), 3.67 - 3.76 (m, 8 H, (8-11)-CH_2), 1.48 (s, 9 H, 27-CH_3). ^{13}C NMR (101 MHz, $CDCl_3$) δ_C ppm 170.6 (C6), 168.9 (C13), 165.3 (C18), 154.5 (C25), 140.6 (C14), 135.0 (C4), 130.5 (C20), 130.4 (C19), 129.6 (C17), 128.6 (C2), 128.5 (C1), 128.45 (C16), 128.4 (C3), 126.0 (C22), 125.7 (C23), 125.6 (C24), 124.4 (C21), 119.5 (C15), 81.2 (C26), 70.7 (C8), 70.4 (C9), 70.1 (C12), 70.0 (C10), 69.7 (C11), 68.2 (C7), 66.9 (C5), 28.2 (C27). HRMS (ESI) m/z: $[M+H]^+$ calculated for $C_{33}H_{40}N_3O_9$: 622.2765, found 622.2764.

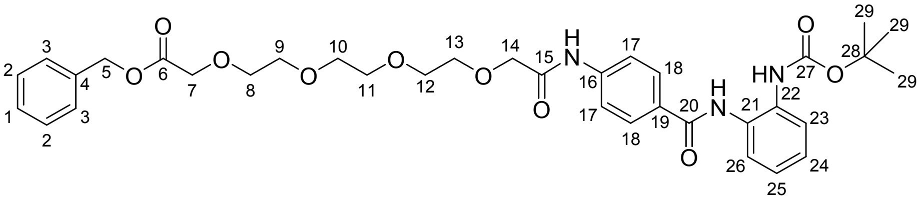

Benzyl 14-((4-((2-((tert-butoxycarbonyl)amino)phenyl)carbamoyl)phenyl)amino)-14-oxo-3,6,9,12-tetraoxatetradecanoate (52l): Following general method E, **52l** was obtained from **39c** (241.2 mg, 0.68 mmol mmol) and **35a** (222.0 mg, 0.68 mmol). The crude product was purified by column chromatography (0-100% EtOAc in hexane, product eluted at 90% EtOAc) to afford **52l** (294.2 mg, 0.44 mmol, 65 % yield) as a clear pale yellow oil. ^{1}H NMR (400 MHz, $CDCl_3$) δ_H ppm 9.14 (br s, 1 H, 21-NH), 9.03 (s, 1 H, 16-NH), 7.97 (d, *J*=8.7 Hz, 2 H, 18-CH), 7.75 (d, *J*=8.7 Hz, 2 H, 17-CH), 7.71 (dd, *J*=7.5, 1.9 Hz, 1 H, 23-CH), 7.30 - 7.35 (m, 6 H, (1-3)-CH,26-CH), 7.14 - 7.22 (m, 2 H, 24-CH,25-CH), 7.00 (s, 1 H, 22-NH), 5.19 (s, 2 H, 5-CH_2), 4.15 (s, 2 H, 7-CH_2), 4.12 (s, 2 H, 14-CH_2), 3.67 - 3.76 (m, 12 H, (8-13)-CH_2), 1.51 (s, 9 H , 29-CH_3). ^{13}C NMR (101 MHz, $CDCl_3$) δ_C ppm 170.4 (C6), 168.7 (15C), 165.1 (C20), 154.4 (C27), 140.7 (C16), 135.3 (C4), 130.6 (C22), 130.4 (C21), 129.7 (C19), 128.6 (C2), 128.5 (C1), 128.45 (C18), 128.4 (C3), 125.9 (C24), 125.7 (C25), 125.6 (C26), 124.3 (C23), 119.4 (C17), 81.2 (C28), 70.8 (alkoxy CH_2), 70.7 (alkoxy CH_2), 70.6 (alkoxy CH_2), 70.5 (alkoxy CH_2), 70.45 (alkoxy CH_2), 70.4 (alkoxy CH_2), 70.0 (alkoxy CH_2), 68.5 (C7), 66.5 (C5), 28.3 (C29). HRMS (ESI) m/z: $[M+H]^+$ calculated for $C_{35}H_{44}O_{10}N_3$ $[M+H]^+$: 666.3027, found 666.3027.

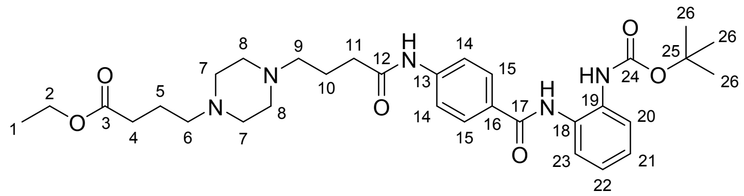

Ethyl 4-(4-(4-((4-((2-((tert-butoxycarbonyl)amino)phenyl)carbamoyl)phenyl)amino)-4-oxobutyl)piperazin-1-yl)butanoate (52m): Following general method E, **52m** was obtained from **51** (160.0 mg, 0.559 mmol) and 35a (201.2 mg, 0.615 mmol). The crude product was purified by column chromatography (1-10% MeOH in DCM) to give **52m** (121.1 mg, 0.203 mmol, 36% yield) as a pale yellow/off-white solid. ^{1}H NMR (400 MHz, CD_3OD) δ_H ppm 7.94 (d, *J*=8.7 Hz, 2 H, 15-CH), 7.75 (d, *J*=8.7 Hz, 2 H, 14-CH), 7.57 - 7.65 (m, 1 H, 23-CH), 7.41 - 7.45 (m, 1 H, 20-CH), 7.19 - 7.26 (m, 2 H, 21-CH,22-CH), 4.11 (q, *J*=7.2 Hz, 2 H, 2-CH_2), 2.80 - 3.04 (m, 8 H, 7-CH_2,8-CH_2), 2.78 (br t, *J*=7.2 Hz, 2 H, 9-CH_2), 2.68 (br t, *J*=7.4 Hz, 2 H, 6-CH_2), 2.52 (t, *J*=6.9 Hz, 2 H, 11-CH_2), 2.38 (t, *J*=7.1 Hz, 2 H, 4-CH_2), 1.92 - 2.03 (m, 2 H, 10-CH_2), 1.80 - 1.88 (m, 2 H, 5-CH_2), 1.50 (s, 9 H, 26-CH_3), 1.23 (t, *J*=7.2 Hz, 3 H, 1-CH_3). ^{13}C NMR (101 MHz, CD_3OD) δ_C ppm 174.8 (C3), 174.2 (C12), 167.8 (C17), 156.4 (C24), 143.8 (C13), 133.1 (C19), 131.8 (C18), 130.4 (C16), 129.8 (C15), 127.5 (C21/22), 127.3 (C23), 126.4 (C21/22), 125.7 (C20), 120.4 (C14), 81.9 (C25), 61.8 (C2), 58.0 (C9), 57.7 (C6), 52.7 (C7/8), 52.6 (C7/8), 35.4 (C11), 32.3 (C4), 28.8 (C26), 22.4 (C10), 21.8 (C5), 14.6 (C1). HRMS (ESI) m/z: $[M+H]^+$ calculated for $C_{32}H_{46}N_5O_6$: 596.3448, found 596.3447.

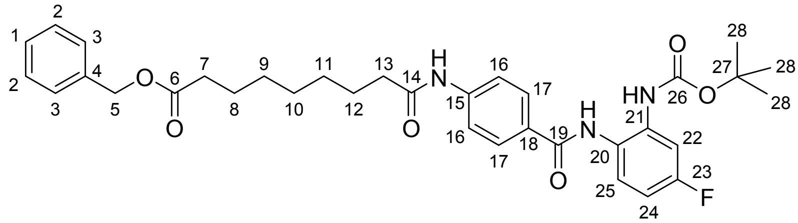

Benzyl 9-((4-((2-((tert-butoxycarbonyl)amino)-4-fluorophenyl)carbamoyl)phenyl)amino)-9-oxononanoate (52n): Following general method E, **52n** was obtained from **37a** (125.7 mg, 0.452 mmol) in and **35b** (120.0 mg, 0.347 mmol). The crude product was purified by column chromatography (10-80% EtOAc in hexane) to give **52n** (132.7 mg, 0.217 mmol, 63% yield) as a pale orange tar. ^{1}H NMR (400 MHz, $CDCl_3$) δ_H ppm 9.12 (br s, 1 H, 20-NH), 8.12 (s, 1 H, 15-NH), 7.85 (d, *J*=8.6 Hz, 2 H, 17-CH), 7.57 (d, *J*=8.6 Hz, 2 H, 16-NH), 7.43 (dd, J_{HH}=8.8, J_{HF}=5.9 Hz, 1 H, 25-CH), 7.41 (s, 1 H, 21-NH), 7.30 - 7.38 (m, 5 H, (1-3)-CH), 7.25 (dd, J_{HF}=10.0, J_{HH}=2.8 Hz, 1 H, 22-CH), 6.77 (ddd, J_{HH}=8.8, 2.8 J_{HF}=7.7 Hz, 1 H, 24-CH), 5.12 (s, 2 H, 5-CH_2), 2.30 - 2.39 (m, 4 H, 7,13-CH_2), 1.59 - 1.71 (m, 4 H, 8,12-CH_2), 1.48 (s, 9 H, 28-CH_3), 1.28 - 1.34 (m, 6 H, (9-11)-CH_2). ^{13}C NMR (101 MHz, $CDCl_3$) δ_C ppm 173.8 (C6), 172.1 (C14), 165.8 (C19), 160.5 (d, J_{CF}=245.1 Hz, C23), 153.9 (C26), 141.6 (C15), 135.9 (C4), 133.1 (d, J_{CF}=10.9 Hz, C21), 128.6 (C18), 128.5 (C2), 128.5 (C17), 128.2 (C1), 128.1 (C3), 127.4 (d, J_{CF}=9.2 Hz, C25), 125.2 (d, J_{CF}=3.2 Hz, C20), 119.2 (C16), 111.6 (d, J_{CF}=22.5 Hz, C24), 110.5 (d, J_{CF}=25.9 Hz, C22), 81.5 (C27), 66.1 (C5), 37.5 (C13), 34.2 (C7), 28.9 (alkyl CH_2), 28.8 (alkyl CH_2), 28.8 (alkyl CH_2), 28.2 (C28), 25.3 (C12), 24.7 (C8). ^{19}F NMR (376 MHz, $CDCl_3$) δ_F ppm -114.5. HRMS (ESI) m/z: $[M+H]^+$ calculated for $C_{34}H_{41}FN_3O_6$: 606.2979, found 606.2986.

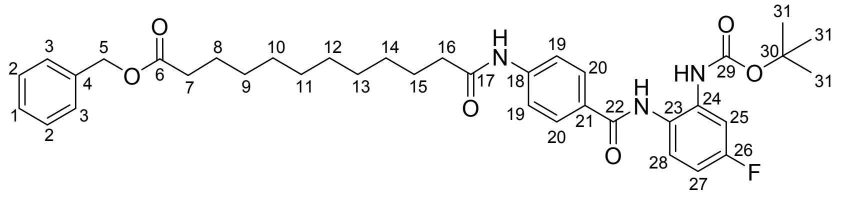

Benzyl 12-((4-((2-((tert-butoxycarbonyl)amino)-4-fluorophenyl)carbamoyl)phenyl)amino)-12-oxododecanoate (52o): Following general method E, **52o** was obtained from **37d** (246.4 mg, 0.767 mmol) in and **35b** (201.8 mg, 0.584 mmol). The crude product was purified by column chromatography (10-80% EtOAc in hexane) to give **52o** (332.4 mg, 0.508 mmol, 87% yield) as a dark orange tar. ^{1}H NMR (400 MHz, $CDCl_3$) δ_H ppm 9.07 (br s, 1 H, 23-NH), 7.94 (br s, 1 H, 18-NH), 7.86 (d, J=8.6 Hz, 2 H, 20-CH), 7.57 (d, J=8.6 Hz, 2 H, 19-CH), 7.44 (dd, J_{HH}=8.8, J_{HF}=5.9 Hz, 1 H, 28-CH), 7.30 - 7.40 (m, 6 H, (1-3)-CH, 24-NH), 7.24 (dd, J_{HF}=9.9, J_{HH}=2.8 Hz, 1 H, 25-CH), (ddd, J_{HF} =9.6, J_{HH}=8.8, 2.8 Hz, 1 H, 27-CH), 5.12 (s, 2 H, 5-CH_2), 2.32 - 2.38 (m, 4 H, 7,16-CH_2), 1.70 (quin, J=6.9 Hz, 2 H, 15-CH_2), 1.63 (quin, J=7.2 Hz, 2 H, 8-CH_2), 1.49 (s, 9 H, 31-CH_3), 1.24 - 1.35 (m, 12 H, (9-14)-CH_2). ^{13}C NMR (101 MHz, $CDCl_3$) δ_C ppm 173.8 (C6), 172.1 (C17), 160.4 (d, J_{CF}=245.7 Hz, C26), 153.9 (C29), 141.5 (C18), 136.0 (C4), 133.0 (d, J_{CF}=10.7 Hz, C24), 128.7 (C21), 128.6 (C2), 128.5 (C20), 128.2 (C1), 128.1 (C3), 127.4 (d, J_{CF}=9.0 Hz, C28), 125.3 (d, J_{CF}=3.1 Hz, C23), 119.2 (C19), 111.7 (d, J_{CF}=22.3 Hz, C27), 110.5 (d, J_{CF}=25.9 Hz, C25), 81.5 (C30), 66.1 (C5), 37.6 (C16), 34.3 (C7), 29.3 (alkyl CH_2), 29.2 (alkyl CH_2), 29.15 (alkyl CH_2), 29.1 (alkyl CH_2), 29.05 (alkyl CH_2), 29.0 (alkyl CH_2), 28.2 (C31), 25.4 (C15), 24.9 (C8). ^{19}F NMR (376 MHz, $CDCl_3$) δ_F ppm -114.5. HRMS (ESI) m/z: $[M+H]^+$ calculated for $C_{37}H_{47}FN_3O_6$: 648.3449, found 648.3452.

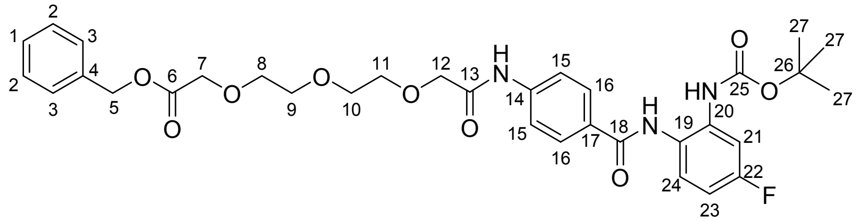

Benzyl 2-(2-(2-(2-((4-((2-((tert-butoxycarbonyl)amino)-4-fluorophenyl)carbamoyl)phenyl)amino)-2-oxoethoxy)ethoxy)ethoxy)acetate (52p): Following general method E, **52p** was obtained from **39b** (141.1 mg, 0.452 mmol) in and **35b** (120.0 mg, 0.347 mmol). The crude product was purified by column chromatography (0-100% EtOAc in hexane) to give **52p** (84.8 mg, 0.131 mmol, 38% yield) as a pale brown/purple tar. ^{1}H NMR (400 MHz, $CDCl_3$) δ_H ppm 8.91 (br s, 1 H, 19-NH), 8.88 (s, 1 H, 14-NH), 7.86 (d, J=8.6 Hz, 2 H, 16-CH), 7.66 (d, J=8.6 Hz, 2 H, 15-CH), 7.42 (dd, J_{HH}=8.7, J_{HF}=5.9 Hz, 1 H, 24-CH), 7.28 - 7.36 (m, 5 H, (1-3)-CH), 7.27 (s, 1 H, 20-NH), 7.23 - 7.27 (m, 1 H, 21-CH), 6.78 (ddd, J=8.7, 2.8, J_{HF}= 7.7 Hz, 1 H, 23-CH), 5.15 (s, 2 H, 5-CH_2), 4.13 (s, 2 H, 7-CH_2), 4.09 (s, 2 H, 12-CH_2), 3.66 - 3.76 (m, 8 H, (8-11)-CH_2), 1.46 (s, 9 H, 27-CH_3). ^{13}C NMR (101 MHz, $CDCl_3$) δ_C ppm 170.5 (C6), 168.9 (C13), 165.7 (C18), 160.5 (d, J_{CF}=244.9 Hz, C22), 153.9 (C25), 140.7 (C14), 135.1 (C4), 133.1 (d, J_{CF}=11.1 Hz, C20), 129.2 (C17), 128.6 (C2), 128.5 (C16), 128.5 (C1), 128.4 (C3), 127.4 (d, J_{CF}=9.9 Hz, C24), 125.2 (d, J_{CF}=3.1 Hz, C19), 119.5 (C15), 111.6 (d, J_{CF}=22.3 Hz, C23), 110.5 (d, J_{CF}=25.6 Hz, 21C), 81.4 (C26), 70.8 (C8), 70.5

(C9), 70.2 (C12), 70.1 (C10), 69.8 (C11), 68.3 (C7), 66.8 (C5), 28.2 (C27). ^{19}F NMR (376 MHz, $CDCl_3$) δ_F ppm -114.6.HRMS (ESI) m/z: $[M+H]^+$ calculated for $C_{33}H_{39}FN_3O_9$: 640.2670, found 640.2668.

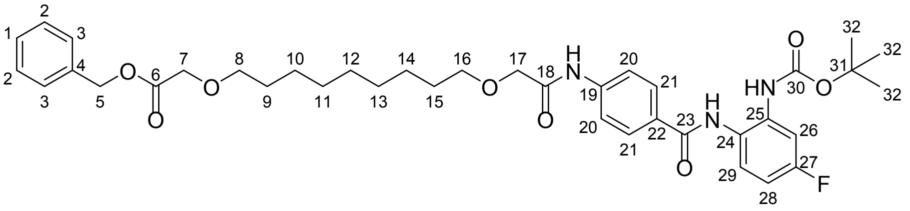

Benzyl 2-((9-(2-((4-((2-((tert-butoxycarbonyl)amino)-4-fluorophenyl)carbamoyl)phenyl)amino)-2-oxoethoxy)nonyl)oxy)acetate (52q): Following general method E, **52q** was obtained from **44b** (167.7 mg, 0.458 mmol) and **35b** (120.0 mg, 0.347 mmol). The crude product was purified by column chromatography (25-30% EtOAc in hexane) to afford **52q** (114.8 mg, 0.164 mmol, 42% yield) as an orange tar. ^{1}H NMR (400 MHz, $CDCl_3$) δ_H ppm 8.91 (br s, 1 H, 24-NH), 8.49 (s, 1 H, 19-NH), 7.96 (d, J=8.7 Hz, 2 H, 21-CH), 7.69 (d, J=8.7 Hz, 2 H, 20-CH), 7.51 (dd, J_{HH}=8.8, J_{HF}=5.8 Hz, 1 H, 29-CH), 7.30 - 7.41 (m, 5 H, (1-3)-CH), 7.24 (dd, J_{HF}=9.9, J_{HH}=2.8 Hz, 1 H, 26-CH), 7.12 (s, 1 H, 25-NH), 6.85 (ddd, J_{HH}=8.8, 2.8, J_{HF}=7.7 Hz, 1 H, 28-CH), 5.19 (s, 2 H, 5-CH_2), 4.11 (s, 2 H, 7-CH_2), 4.06 (s, 2 H, 17-CH_2), 3.61 (t, J=6.6 Hz, 2 H, 16-CH_2), 3.53 (t, J=6.6 Hz, 2 H, 8-CH_2), 1.66 - 1.73 (m, 2 H, 15-CH_2), 1.59 - 1.64 (m, 2 H, 9-CH_2), 1.51 (s, 9 H, 32-CH_3), 1.30 - 1.43 (m, 10 H, (10-14)-CH_2). ^{13}C NMR (101 MHz, $CDCl_3$) δ_C ppm 170.5 (C6), 168.3 (C18), 165.4 (C23), 160.5 (d, J_{CF}=244.9 Hz, C27), 153.9 (C30), 140.5 (C19), 135.4 (C4), 132.8 (d, J_{CF}=10.9 Hz, C25), 129.4 (C22), 128.7 (C21), 128.6 (C2), 128.4 (C1), 128.4 (C2), 127.4 (d, J_{CF}=9.3 Hz, C29), 125.5 (d, J_{CF}=2.9 Hz, C24), 119.2 (C20), 111.9 (d, J_{CF}=22.5 Hz, C28), 110.6 (d, J_{CF}=25.9 Hz, C26), 81.5 (C31), 72.1 (C16), 72.0 (C8), 70.2 (C17), 68.3 (C7), 66.5 (C5), 29.5 (alkyl CH_2), 29.4 (alkyl CH_2), 29.4 (alkyl CH_2), 29.3 (alkyl CH_2), 29.3 (alkyl CH_2), 28.2 (32), 26.0 (C10/14), 25.9 (C10/14). ^{19}F NMR (376 MHz, $CDCl_3$) δ_F ppm -114.7. HRMS (ESI) m/z: $[M+H]^+$ calculated for $C_{38}H_{49}FN_3O_8$: 694.3504, found 694.3517.

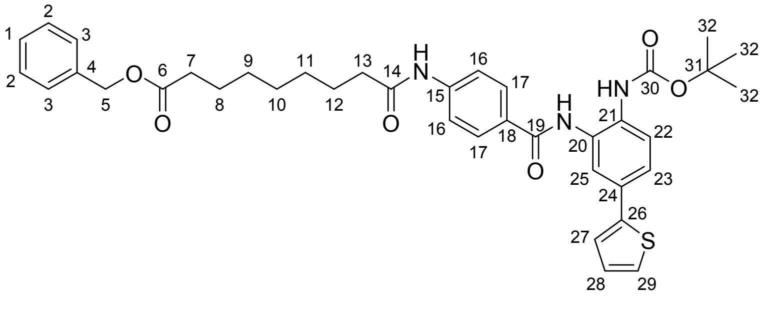

Benzyl 9-((4-((2-((tert-butoxycarbonyl)amino)-5-(thiophen-2-yl)phenyl)carbamoyl)phenyl)amino)-9-oxononanoate (52r): Following general method E, **52r** was obtained from **37a** (141.1 mg, 0.452 mmol) in and **35c** (120.0 mg, 0.347 mmol). The crude product was purified by column chromatography (0-100% EtOAc in hexane) to give **52r** (84.8 mg, 0.131 mmol, 38% yield) as a pale brown/purple tar. ^{1}H NMR (400 MHz, $CDCl_3$) δ_H ppm 9.36 (br s, 1 H, 20-NH), 8.12 (s, 1 H, 18-NH), 7.89 (s, 1 H, 25-CH), 7.87 (d, J=8.7 Hz, 2 H, 17-CH), 7.56 (d, J=8.7 Hz, 2 H, 16-CH), 7.29 - 7.40 (m, 8 H, (1,2,3,22,23)-CH, 21-NH), 7.17 - 7.20 (m, 2 H, 27,29-CH), 6.96 - 7.01 (m, 1 H, 28-CH), 5.12 (s, 2 H, 5-CH_2), 2.29 - 2.39 (m, 4 H, 7,13-CH_2), 1.57 - 1.71 (m, 4 H, 8,12-CH_2), 1.48 (s, 9 H, 32-CH_3), 1.26 - 1.32 (m, 6 H, (9-11)-CH_2). ^{13}C NMR (101 MHz, $CDCl_3$) δ_C ppm

173.8 (C6), 172.0 (C14), 165.6 (C19), 154.5 (C30), 143.1 (C26), 141.7 (C15), 135.9 (C4), 131.8 (C24), 130.6 (C20), 129.8 (C21), 128.7 (C18), 128.5 (C2), 128.4 (C17), 128.2 (C1), 128.1 (C3), 127.9 (C28), 124.8 (C22/23), 124.7 (C27/29), 123.4 (C22/23), 123.2 (C27/29), 122.9 (C25), 119.1 (C16), 81.3 (C31), 66.1 (C5), 37.5 (C13), 34.2 (C7), 28.9 (alkyl CH_2), 28.85 (alkyl CH_2), 28.8 (alkyl CH_2), 28.2 (C32), 25.3 (C12), 24.8 (C8). HRMS (ESI) m/z: $[M+H]^+$ calculated for $C_{38}H_{44}N_3O_6S$: 670.2951, found 670.2950.

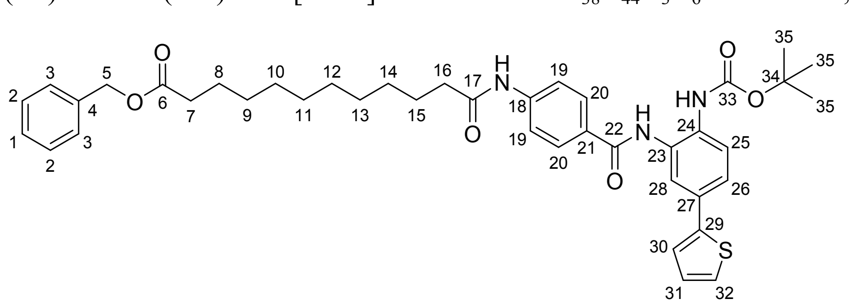

Benzyl 12-((4-((2-((tert-butoxycarbonyl)amino)-5-(thiophen-2-yl)phenyl)carbamoyl)phenyl)amino)-12-oxododecanoate (52s): Following general method E, **52s** was obtained from **37d** (59.8 mg, 0.187 mmol) in and **35c** (84.1 mg, 0.205 mmol). The crude product was purified by column chromatography (0-100% EtOAc in hexane) to give **52s** (78.1 mg, 0.109 mmol, 58% yield) as a yellow tar. ^{1}H NMR (400 MHz, $CDCl_3$) δ_H ppm 9.26 (br s, 1 H, 23-NH), 7.95 (d, *J*=1.7 Hz, 1 H, 28-CH), 7.90 (d, *J*=8.7 Hz, 2 H, 20-CH), 7.75 (br s, 1 H, 18-NH), 7.58 (d, *J*=8.7 Hz, 2 H, 19-CH), 7.32 - 7.38 (m, 6 H, 1,2,3,26-CH), 7.30 (d, *J*=8.4 Hz, 1 H, 25-CH), 7.19 - 7.25 (m, 2 H, 30,32-CH), 7.14 (s, 1 H, 24-NH), 7.02 (dd, *J*=5.0, 3.7 Hz, 1 H, 31-CH), 5.12 (s, 2 H, 5-CH_2), 2.32 - 2.38 (m, 4 H, 7,16-CH_2), 1.71 (quin, *J*=7.5 Hz, 2 H, 15-CH_2), 1.64 (quin, *J*=7.2 Hz, 2 H, 8-CH_2), 1.50 (s, 9 H, 35-CH_3), 1.27 - 1.35 (m, 12 H, (9-14)-CH_2). ^{13}C NMR (101 MHz, $CDCl_3$) δ_C ppm 173.8 (C6), 171.9 (C17), 165.4 (C22), 154.5 (C33), 143.2 (C29), 141.5 (C18), 136.0 (C4), 132.0 (C27), 130.8 (C23), 129.5 (C24), 129.0 (C21), 128.55 (C2), 128.5 (C20), 128.2 (C1), 128.1 (C3), 128.0 (C31), 124.8 (C25), 124.75 (C30), 123.4 (C26), 123.3 (C32), 122.0 (C28), 119.1 (C19), 81.4 (C34), 66.1 (C5), 37.7 (C16), 34.3 (C7), 29.35 (alkyl CH_2), 29.3 (alkyl CH_2), 29.25 (alkyl CH_2), 29.2 (alkyl CH_2), 29.1 (alkyl CH_2), 29.0 (alkyl CH_2), 28.3 (C35), 25.4 (C15), 24.9 (C8). HRMS (ESI) m/z: $[M+H]^+$ calculated for $C_{41}H_{50}N_3O_6S$: 712.3420, found 712.3419.

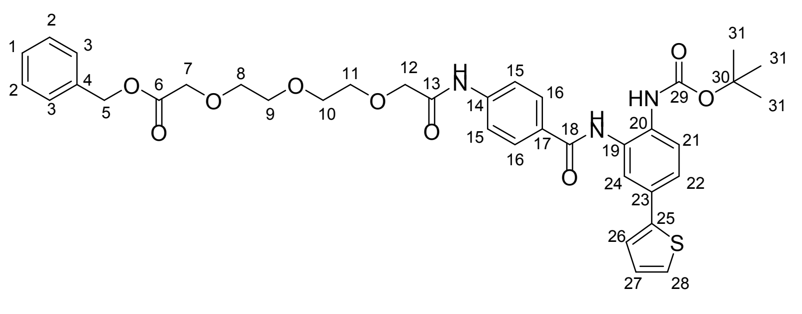

Benzyl 2-(2-(2-(2-((4-((2-((tert-butoxycarbonyl)amino)-5-(thiophen-2-yl)phenyl)carbamoyl)phenyl) amino)-2-oxoethoxy)ethoxy)ethoxy)acetate (52t): Following general method E, **52t** was obtained from **39b** (114.0 mg, 0.362 mmol) and **35c** (114.0 mg, 0.278 mmol). The crude product was purified by column chromatography (0-100% EtOAc in hexane, product eluted at 80-90% EtOAc) to afford **52t** (117.5 mg, 0.162 mmol, 58% yield) as a dark yellow tar. ^{1}H NMR (400 MHz, $CDCl_3$) δ_H ppm 9.17 (br s, 1 H, 19-NH), 8.90 (s, 1 H, 14-NH), 7.98 (d, *J*=1.8 Hz, 1 H, 24-CH), 7.94 (d, *J*=8.7 Hz, 2 H, 16-CH), 7.72 (d, *J*=8.7 Hz, 2 H, 15-CH), 7.38 (td, *J*=8.3, 2.0 Hz, 1 H, 22-CH), 7.29 - 7.35 (m, 6 H, 1,2,3,21-CH, 7.23 - 7.27 (m, 2 H, 26-CH, 28-

CH), 7.00 - 7.08 (m, 2 H, 27-CH, 20-NH), 5.18 (s, 2 H, 5-CH_2), 4.16 (s, 2 H, 7-CH_2), 4.12 (s, 2 H, 12-CH_2), 3.69 - 3.79 (m, 8 H, (8-11)-CH_2), 1.50 (s, 9 H, 31-CH_3). ^{13}C NMR (101 MHz, $CDCl_3$) δ_C ppm 170.3 (C6), 168.7 (C13), 165.3 (C18), 154.4 (C29), 143.3 (C25), 140.7 (C14), 135.2 (C4), 132.0 (C23), 130.9 (C19), 129.6 (C17),129.5 (C20), 128.6 (C2), 128.5 (C1/16), 128.45 (C1/16), 128.4 (C3), 128.0 (C27), 124.8 (C28), 124.7 (C21), 123.4 (C22), 123.3 (C26), 122.9 (C24), 119.5 (C15), 81.4 (C30), 71.1 (C11), 70.7 (C8), 70.4 (C9/10), 70.3 (C12), 70.0 (C9/10), 68.4 (C7), 66.7 (C5), 28.2 (C31). HRMS (ESI) m/z: $[M+H]^+$ calculated for $C_{37}H_{42}N_3O_9S$: 704.2642, found 704.2637.

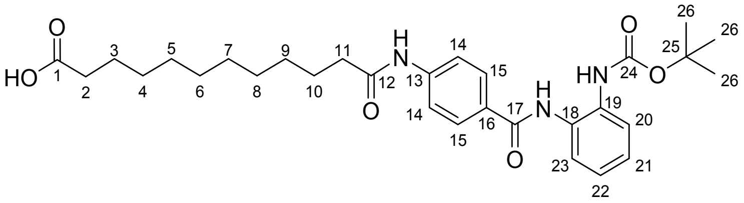

12-((4-((2-((Tert-butoxycarbonyl)amino)phenyl)carbamoyl)phenyl)amino)-12-oxododecanoic acid (53a) : Following general method F, benzyl ester hydrogenolysis of **52a** (171 mg, 0.271 mmol) was performed to afford **53a** (146 mg, 0.266 mmol, 98% yield) as an off-white solid. ^{1}H NMR (400 MHz, DMSO-d_6) δ_H ppm 11.96 (br s, 1 H, CO_2H), 10.17 (s, 1 H, 13-NH), 9.74 (s, 1 H, 18-NH), 8.68 (br s, 1 H, 19-NH), 7.91 (d, J=8.5 Hz, 2 H, 15-CH), 7.74 (d, J=8.5 Hz, 2 H, 14-CH), 7.48 - 7.59 (m, 2 H, 20-CH,23-CH), 7.09 - 7.24 (m, 2 H, 21-CH,22-CH), 2.34 (t, J=7.3 Hz, 2 H, 11-CH_2), 2.18 (t, J=7.3 Hz, 2 H, 2-CH_2), 1.55 - 1.65 (m, 2 H, 10-CH_2), 1.46 - 1.51 (m, 2 H, 3-CH_2), 1.45 (s, 9 H, 26-CH_3), 1.23 - 1.30 (m, 12 H, (4-9)-CH_2). ^{13}C NMR (101 MHz, DMSO-d_6) δ_C ppm 174.5 (C1), 171.8 (C12), 164.7 (C17), 153.5 (C24), 142.6 (C13), 131.6 (C19), 130.0 (C18), 128.5 (C15), 128.2 (C16), 126.0 (C23), 125.4 (C22), 124.1 (C21), 123.9 (C20), 118.2 (C14), 79.7 (C25), 36.5 (C11), 33.7 (C2), 28.9 (2x alkyl CH_2), 28.8 (alkyl CH_2), 28.7 (alkyl CH_2), 28.6 (alkyl CH_2), 28.5 (alkyl CH_2), 28.0 (C26), 25.0 (C10), 24.5 (C3). HRMS (ESI) m/z: $[M+Na]^+$ calculated for $C_{30}H_{41}N_3O_6Na$: 562.2893, found 562.2886.

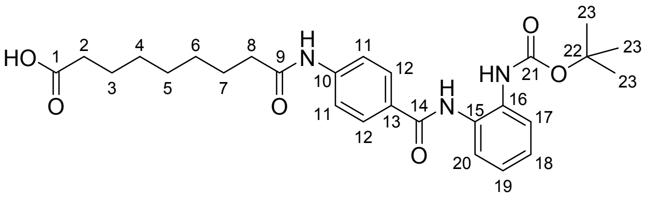

9-((4-((2-((tert-butoxycarbonyl)amino)phenyl)carbamoyl)phenyl)amino)-9-oxononanoic acid (53b): Following general method F, benzyl ester hydrogenolysis of **52b** (370.8 mg, 0.63 mmol) was performed to afford **53b** (295.1 mg, 0.59 mmol, 93% yield) as a white crystalline solid. ^{1}H NMR (400 MHz, DMSO-d_6) δ_H ppm 11.87 (br s, 1 H, CO_2H), 10.17 (s, 1 H, 10-NH), 9.74 (s, 1 H, 15-NH), 8.67 (br s, 1 H, 16-NH), 7.91 (d, J=8.8 Hz, 2 H, 12-CH), 7.74 (d, J=8.8 Hz, 2 H, 11-CH), 7.48 - 7.57 (m, 2 H, 17,20-CH), 7.10 - 7.22 (m, 2 H, 18,19-CH), 2.35 (t, J=7.4 Hz, 2 H, 8-CH_2), 2.20 (t, J=7.4 Hz, 2 H, 2-CH_2), 1.55 - 1.65 (m, 2 H, 7-CH_2), 1.47 - 1.54 (m, 2 H, 3-CH_2), 1.45 (s, 9 H, 23-CH_3), 1.30 (br s, 6 H, (4-6)-CH_2). ^{13}C NMR (101 MHz, DMSO-d_6) δ_C ppm 174.5 (C1), 171.8 (C9), 164.7 (C14), 153.5 (C21), 142.5 (C10), 131.6 (C16), 130.0 (C15), 128.5 (C12), 128.2 (C16), 126.0 (C20), 125.4 (C18), 124.1 (C19), 123.9 (C17), 118.2 (C11), 79.7 (C22), 36.4 (C8), 33.6

(C2), 28.55 (CH_2), 28.5 (CH_2), 28.4 (CH_2), 28.0 (C23), 24.9 (C7), 24.5 (C3). HRMS (ESI) m/z: $[M+H]^+$ calculated for $C_{27}H_{36}N_3O_6$: 498.2604, found 498.2597.

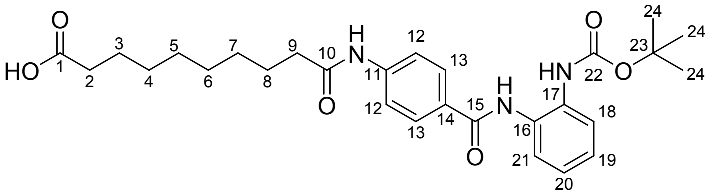

10-((4-((2-((tert-butoxycarbonyl)amino)phenyl)carbamoyl)phenyl)amino)-10-oxodecanoic acid (53c): Following general method F, benzyl ester hydrogenolysis of **52c** (322 mg, 0.535 mmol) was performed to afford **53c** (263 mg, 0.514 mmol, 96% yield) as a white crystalline solid. ^{1}H NMR (400 MHz, DMSO-d_6) δ_H ppm 11.95 (br s, 1 H, 1-CO_2H), 10.17 (s, 1 H, 11-NH), 9.74 (s, 1 H, 16-NH), 8.68 (br s, 1 H, 17-NH), 7.90 (d, *J*=8.7 Hz, 2 H, 13-CH), 7.73 (d, *J*=8.7 Hz, 2 H, 12-CH), 7.48 - 7.56 (m, 2 H, 18-CH,21-CH), 7.12 - 7.22 (m, 2 H, 19-CH,20-CH), 2.34 (t, *J*=7.4 Hz, 2 H, 9-CH_2), 2.18 (t, *J*=7.3 Hz, 2 H, 2-CH_2), 1.56 - 1.64 (m, 2 H, 8-CH_2), 1.46 - 1.52 (m, 2 H, 3-CH_2), 1.45 (s, 9 H, 24-CH_3), 1.23 - 1.32 (m, 8 H, (4-7)-CH_2). ^{13}C NMR (101 MHz, DMSO-d_6) δ_C ppm 174.5 (C1), 171.8 (10), 164.7 (C15), 153.5 (C22), 142.6 (C11), 131.6 (C17), 130.0 (C16), 128.5 (C13), 128.2 (C17), 126.0 (C21), 125.4 (C29), 124.1 (C20), 123.9 (C18), 118.2 (C12), 79.7 (C23), 36.5 (C9), 33.6 (C2), 28.8 (alkyl CH_2), 28.7 (alkyl CH_2), 28.6 (alkyl CH_2), 28.5 (alkyl CH_2), 28.0 (C24), 24.9 (C8), 24.5 (C3). HRMS (ESI) m/z: $[M+H]^+$ calculated for $C_{28}H_{38}N_3O_6$: 512.2761, found 512.2742.

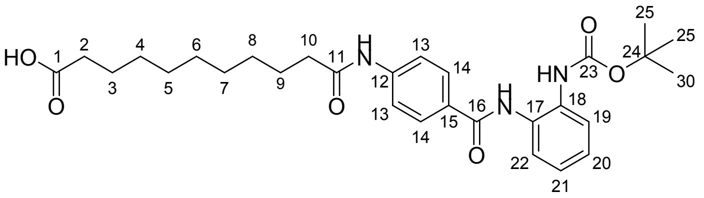

11-((4-((2-((tert-butoxycarbonyl)amino)phenyl)carbamoyl)phenyl)amino)-11-oxoundecanoic acid (53d): Following general method F, benzyl ester hydrogenolysis of **52d** (282 mg, 0.458 mmol) was performed to afford **53d** (240 mg, 0.456 mmol, 100% yield) as a white crystalline solid. ^{1}H NMR (400 MHz, CD_3CN) δ ppm 9.48 (br s, 1 H, 1-CO_2H), 8.98 (br s, 1 H, 17-NH), 8.54 (br s, 1 H, 12-NH), 7.89 (d, *J*=8.8 Hz, 2 H, 14-CH), 7.71 (d, *J*=8.8 Hz, 2 H, 13-CH), 7.56 (dd, *J*=7.7, 1.6 Hz, 1 H, 22-CH), 7.49 - 7.54 (m, 2 H, 19-CH,18-NH), 7.15 - 7.26 (m, 2 H, 20-CH,21-CH), 2.34 (t, *J*=7.4 Hz, 2 H, 10-CH_2), 2.25 (t, *J*=7.4 Hz, 2 H, 2-CH_2), 1.62 - 1.69 (m, 2 H, 9-CH_2), 1.51 - 1.59 (m, 2H, 3-CH_2), 1.47 (s, 9 H, 25-CH_3), 1.27 - 1.36 (m, 10 H, (4-8)-CH_2). ^{13}C NMR (101 MHz, CD_3CN) δ ppm 175.7 (C1), 173.5 (C11), 166.7 (C16), 155.4 (C23), 144.0 (C12), 133.2 (C18), 131.7 (C17), 130.0 (C15), 129.8 (C14), 127.4 (C20), 127.1 (C22), 126.2 (C21), 125.5 (C19), 120.0 (C13), 81.6 (C24), 38.2 (C10), 34.7 (C2), 30.4 (alkyl CH_2), 30.3 (alkyl CH_2), 30.25 (alkyl CH_2), 30.2 (alkyl CH_2), 30.1 (alkyl CH_2), 28.9 (25-CH_3), 26.5 (C9), 26.0 (C3). HRMS (ESI) m/z: $[M+H]^+$ calculated for $C_{29}H_{40}N_3O_6$: 526.2917, found 526.2909.

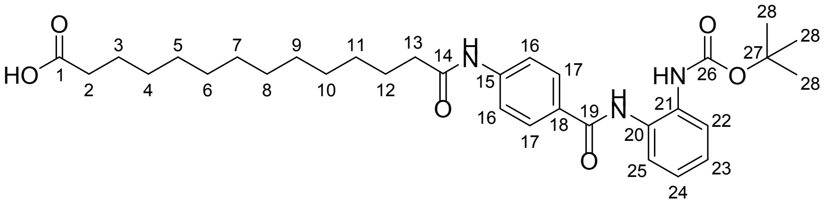

14-((4-((2-((tert-butoxycarbonyl)amino)phenyl)carbamoyl)phenyl)amino)-14-oxotetradecanoic acid (53e): Following general method F, benzyl ester hydrogenolysis of **52e** (348.6 mg, 0.530 mmol) was performed to afford **53e** (302.0 mg, 0.527 mmol, 99% yield) as a white solid. ^{1}H NMR (400 MHz, DMSO-d_6) δ_H ppm 11.95 (br s, 1 H, 1-CO_2H), 10.16 (s, 1 H, 15-NH), 9.74 (s, 1 H, 20-NH), 8.68 (br s, 1 H, 21-NH), 7.91 (d, *J*=8.8 Hz, 2 H, 17-CH), 7.74 (d, *J*=8.8 Hz, 2 H, 16-CH), 7.49 - 7.57 (m, 2 H, 22,25-CH), 7.10 - 7.23 (m, 2 H, 23,24-CH), 2.34 (t, *J*=7.4 Hz, 2 H, 13-CH_2), 2.18 (t, *J*=7.4 Hz, 2 H, 2-CH_2), 1.55 - 1.65 (m, 2 H, 12-CH_2), 1.42 - 1.53 (m, 11 H, 3-CH_2,28-CH_3), 1.22 - 1.32 (m, 16 H, (4-11)-CH_2). ^{13}C NMR (101 MHz, DMSO-d_6) δ_C ppm 174.5 (C1), 171.8 (C14), 164.7 (C19), 153.5 (C26), 142.6 (C15), 131.6 (C21), 130.0 (C20), 128.5 (C17), 128.2 (C18), 125.9 (C25), 125.4 (C24), 124.1 (C23), 123.9 (C22), 118.2 (C16), 79.6 (C27), 36.5 (C13), 33.6 (C2), 29.0 (alkyl CH_2), 29.95 (alkyl CH_2), 28.9 (alkyl CH_2), 28.85 (alkyl CH_2), 28.8 (alkyl CH_2), 28.75 (alkyl CH_2), 28.7 (alkyl CH_2), 28.5 (alkyl CH_2), 28.0 (C28), 25.0 (C12), 24.5 (C3). HRMS (ESI) m/z: $[M+H]^+$ calculated for $C_{32}H_{46}N_3O_6$: 568.3387, found 568.3391.

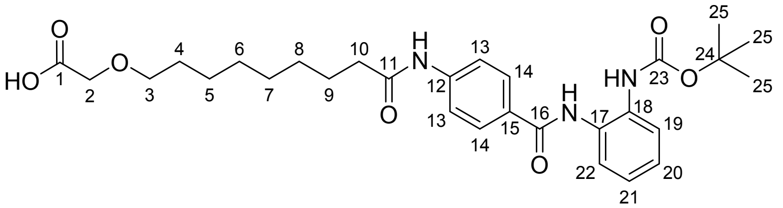

2-((9-((4-((2-((tert-butoxycarbonyl)amino)phenyl)carbamoyl)phenyl)amino)-9-oxononyl)oxy)acetic acid (53f): To a solution of **52f** (62.7 mg, 0.111 mmol) in DCM (2.7 mL) a solution of NaOH in MeOH (4M, 0.3 mL) was added. The reaction was stirred at room temperature for 16 h. The reaction mixture was concentrated *in vacuo*, then redissolved in water (10 mL) and acified with HCl (3M, ca. 1 mL) to pH 2. The product was then extracted in EtOAc (2 x 20 mL), dried over Na_2SO_4, filtered and concentrated *in vacuo* to afford **53f** as a yellow solid (55.4 mg, 0.101 mmol, 91% yield). ^{1}H NMR (400 MHz, CD_3CN) δ_H ppm 9.03 (br s, 1 H, 17-NH), 8.61 (br s, 1 H, 12-NH), 7.90 (d, *J*=8.7 Hz, 2 H, 14-CH), 7.70 (d, *J*=8.7 Hz, 2 H, 13-CH), 7.53 - 7.60 (m, 2 H, 22-CH,18-NH), 7.51 (dd, *J*=7.5, 1.5 Hz, 1 H, 19-CH), 7.22 (app. td, *J*=7.5, 1.5 Hz, 1 H, 20-CH), 7.18 (app. td, *J*=7.5, 1.5 Hz, 1 H, 21-CH), 4.00 (s, 2 H, 2-CH_2), 3.47 (t, *J*=6.6 Hz, 2 H, 3-CH_2), 2.34 (t, *J*=7.5 Hz, 2 H, 10-CH_2), 1.61 - 1.68 (m, 2 H, 9-CH_2), 1.51 - 1.57 (m, 2 H, 4-CH_2), 1.46 (s, 9 H, 25-CH_3), 1.28 - 1.35 (m, 8 H, (5-8)-CH_2). ^{13}C NMR (101 MHz, CD_3CN) δ_C ppm 173.7 (C11), 172.6 (C1), 166.8 (C16), 155.5 (C23), 143.9 (C12), 133.1 (C18), 131.6 (C17), 130.0 (C15), 129.8 (C14), 127.4 (C20), 127.1 (C22), 126.2 (C21), 125.5 (C19), 120.0 (C13), 81.6 (C24), 72.6 (C3), 68.7 (C2), 38.2 (C10), 30.6 (C4), 30.3 (alkyl CH_2), 30.3 (alkyl CH_2), 30.2 (alkyl CH_2), 28.9 (C25), 27.0 (C5), 26.5 (C9). HRMS (ESI) m/z: $[M+H]^+$ calculated for $C_{29}H_{40}N_3O_7$: 542.2866, found 542.2861.

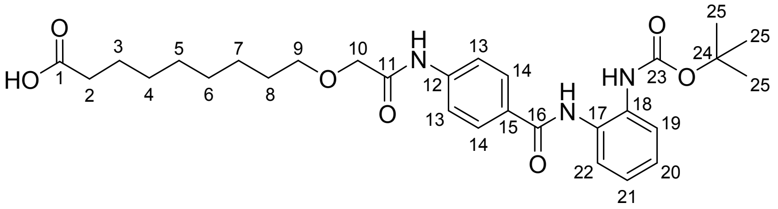

9-(2-((4-((2-((tert-butoxycarbonyl)amino)phenyl)carbamoyl)phenyl)amino)-2-oxoethoxy)nonanoic acid (53g): Following general method F, benzyl ester hydrogenolysis of **52g** (120.2 mg, 0.190 mmol) was performed to afford **53g** (105.6 mg, 0.189 mmol, 99% yield) as a colourless tar. ^{1}H NMR (400 MHz, CD_3CN) δ_H ppm 9.01 (br s, 1 H, 17-NH), 8.75 (br s, 1 H, 12-NH), 7.92 (d, *J*=8.7 Hz, 2 H, 14-CH), 7.77 (d, *J*=8.7 Hz, 2 H, 13-CH), 7.57 (dd, *J*=7.4, 1.7 Hz, 1 H, 22-CH), 7.49 - 7.55 (m, 2 H, 19-CH,18-NH), 7.24 (td, *J*=7.4, 1.7 Hz, 1 H, 20-CH), 7.19 (td, *J*=7.4, 1.7 Hz, 1 H, 21-CH), 4.02 (s, 2 H, 10-CH_2), 3.57 (t, *J*=6.6 Hz, 2 H, 9-CH_2), 2.26 (t, *J*=7.4 Hz, 2 H, 2-CH_2), 1.61 - 1.70 (m, 2 H, 8-CH_2), 1.51 - 1.60 (m, 2 H, 3-CH_2), 1.47 (s, 9 H, 25-CH_3), 1.39 - 1.43 (m, 2 H, 7-CH_2), 1.28 - 1.36 (m, 6 H, (4-6)-CH_2). ^{13}C NMR (101 MHz, CD_3CN) δ_C ppm 175.7 (C1), 170.3 (C11), 166.7 (C16), 155.5 (C23), 142.7 (C12), 133.2 (C18), 131.6 (C17), 130.8 (C15), 129.8 (C14), 127.4 (C20), 127.1 (C22), 126.2 (C21), 125.5 (C19), 120.7 (C13), 81.6 (C24), 73.1 (C9), 71.5 (C10), 34.6 (C2), 30.5 (C8), 30.3 (2x alkyl CH_2), 30.1 (alkyl CH_2), 28.9 (C25), 27.1 (C7), 26.0 (C3). HRMS (ESI) m/z: $[M+H]^+$ calculated for $C_{29}H_{40}N_3O_7$: 542.2866, found 542.2863.

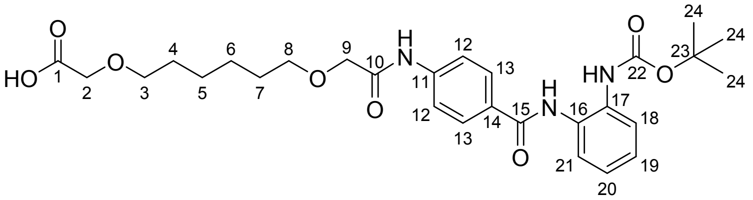

2-((6-(2-((4-((2-((tert-butoxycarbonyl)amino)phenyl)carbamoyl)phenyl)amino)-2-oxoethoxy)hex yl)oxy)acetic acid (53h): Following general method F, benzyl ester hydrogenolysis of **52h** (76.5 mg, 0.121 mmol) was performed to afford **53h** (65.8 mg, 0.120 mmol, 99% yield) as a colourless oil. ^{1}H NMR (400 MHz, CD_3CN) δ_H ppm 9.05 (br s, 1 H, 16-NH), 8.77 (br s, 1 H, 11-NH), 7.92 (d, *J*=8.7 Hz, 2 H, 13-CH), 7.76 (d, *J*=8.7 Hz, 2 H, 12-CH), 7.54 - 7.60 (m, 2 H, 21-CH, 17-NH), 7.51 (dd, *J*=7.5, 1.7 Hz, 1 H, 18-CH), 7.23 (app. td, *J*=7.5, 1.7 Hz, 1 H, 19-CH), 7.18 (app. td, *J*=7.5, 1.7 Hz, 1 H, 20-CH), 4.02 (s, 2 H, 9-CH_2), 4.01 (s, 2 H, 2-CH_2), 3.57 (t, *J*=6.6 Hz, 2 H, 8-CH_2), 3.49 (t, *J*=6.6 Hz, 2 H, 3-CH_2), 1.63 - 1.70 (m, 2 H, 7-CH_2), 1.55 - 1.63 (m, 2 H, 4-CH_2), 1.47 (s, 9 H, 24-CH_3), 1.39 - 1.43 (m, 4 H, 5,6-CH_2). CO_2H not visible. ^{13}C NMR (101 MHz, CD_3CN) δ_C ppm 172.5 (C1), 170.3 (C10), 166.7 (C15), 155.5 (C22), 142.7 (C11), 133.1 (C17), 131.6 (C16), 130.7 (C14), 129.8 (C13), 127.4 (C19), 127.1 (C21), 126.2 (C20), 125.5 (C18), 120.7 (C12), 81.6 (C23), 73.0 (C8), 72.5 (C3), 71.5 (C9), 68.7 (C2), 30.6 (C4), 30.5 (C7), 28.9 (C24), 26.95 (C5/6), 26.9 (C5/6). HRMS (ESI) m/z: $[M+H]^+$ calculated for $C_{28}H_{38}N_3O_8$: 544.2659, found 544.2645.

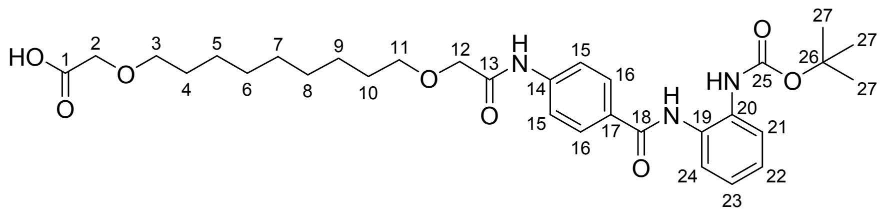

2-((9-(2-((4-((2-((tert-butoxycarbonyl)amino)phenyl)carbamoyl)phenyl)amino)-2-oxoethoxy)non yl)oxy)acetic acid (53i): Following general method F, benzyl ester hydrogenolysis of **52i** (134.8 mg, 0.199 mmol) was performed to afford **53i** (118.7 mg, 0.199 mmol, 100% yield) as a colourless tar. ^{1}H NMR (400 MHz, CD_3CN) δ_H ppm 9.03 (br s, 1 H, 19-NH), 8.75 (br s, 1 H, 14-NH), 7.92 (d, *J*=8.7 Hz, 2 H, 16-CH), 7.77 (d, *J*=8.7 Hz, 2 H, 15-CH), 7.57 (dd, *J*=7.6, 1.7 Hz, 1 H, 24-CH), 7.54 (br s, 1 H, 20-NH), 7.51 (dd, *J*=7.6, 1.7 Hz, 1 H, 21-CH), 7.23 (app. td, *J*=7.6, 1.7 Hz, 1 H, 22-CH), 7.18 (app. td, *J*=7.6, 1.7 Hz, 1 H, 23-CH), 4.02 (s, 2 H, 12-CH_2), 4.00 (s, 2 H, 2-CH_2), 3.57 (t, *J*=6.6 Hz, 2 H, 11-CH_2), 3.47 (t, *J*=6.6 Hz, 2 H, 3-CH_2), 1.65 (quin, *J*=6.7 Hz, 2 H, 10-CH_2), 1.55 (quin, *J*=6.8 Hz, 2 H, 4-CH_2), 1.47 (s, 9 H, 27-CH_3), 1.37 - 1.42 (m, 2 H, 9-CH_2), 1.29 - 1.37 (m, 8 H, (5-8)-CH_2). ^{13}C NMR (101 MHz, CD_3CN) δ_C ppm 172.5 (C1), 170.3 (C13), 166.7 (C18), 155.5 (C25), 142.7 (C14), 133.1 (C20), 131.6 (C19), 130.7 (C17), 129.8 (C16), 127.4 (C22), 127.1 (C24), 126.2 (C23), 125.5 (C21), 120.7 (C15), 81.6 (C26), 73.1 (C11), 72.6 (C3), 71.5 (C12), 68.7 (C2), 30.6 (C4), 30.6 (C10), 30.5 (C6/7/8), 30.45 (C6/7/8), 30.4 (C6/7/8), 28.9 (C27), 27.2 (C9), 27.1 (C5). HRMS (ESI) m/z: $[M+H]^+$ calculated for $C_{31}H_{44}N_3O_8$: 586.3128, found 586.3128.

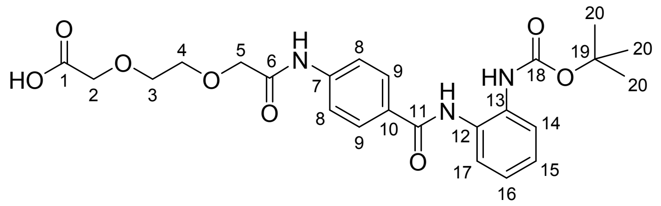

2-(2-(2-((4-((2-((tert-butoxycarbonyl)amino)phenyl)carbamoyl)phenyl)amino)-2-oxoethoxy)ethoxy)ac etic acid (53j): Following general method F, benzyl ester hydrogenolysis of **52j** (145.7 mg, 0.252 mmol) was performed to afford **53j** (122.8 mg, 0.251 mmol, 99% yield) as a pale yellow fluffy solid. ^{1}H NMR (400 MHz, DMSO-*d*$_6$) δ_H ppm 12.63 (br s, 1 H, 1-CO_2H), 9.93 (s, 1 H, 7-NH), 9.76 (s, 1 H, 12-NH), 8.67 (br s, 1 H, 13-NH), 7.93 (d, *J*=8.8 Hz, 2 H, 9-CH), 7.81 (d, *J*=8.8 Hz, 2 H, 8-CH), 7.48 - 7.58 (m, 2 H, 14-CH,17-CH), 7.11 - 7.23 (m, 2 H, 15-CH,16-CH), 4.14 (s, 2 H, 5-CH_2), 4.09 (s, 2 H, 2-CH_2), 3.65 - 3.74 (m, 4 H, 3-CH_2,4-CH_2), 1.45 (s, 9 H, 20-CH_3). ^{13}C NMR (101 MHz, DMSO-*d*$_6$) δ_C ppm 171.7 (C1), 168.8 (C6), 164.7 (C11), 153.5 (C18), 141.5 (C7), 131.7 (C12), 129.9 (C13), 128.9 (C10), 128.5 (C9), 126.0 (C17), 125.5 (C15), 124.1 (C16), 123.9 (C14), 118.8 (C8), 79.7 (C19), 70.3 (C3), 70.1 (C5), 69.8 (C4), 67.6 (C2), 28.0 (C20). HRMS (ESI) m/z: $[M+H]^+$ calculated for $C_{24}H_{30}N_3O_8$: 488.2033, found 488.2025.

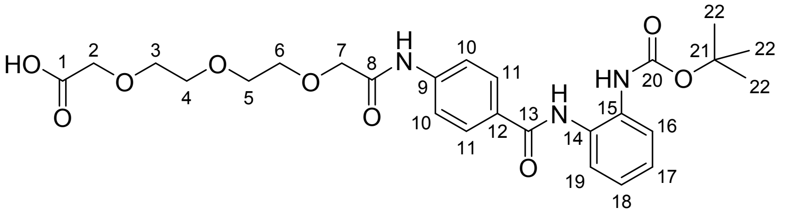

2-(2-(2-(2-((4-((2-((tert-butoxycarbonyl)amino)phenyl)carbamoyl)phenyl)amino)-2-oxoethoxy)ethoxy)ethoxy)acetic acid (53k): Following general method F, benzyl ester hydrogenolysis of **52k (**174.6 mg, 0.281 mmol) was performed to afford **53k** (139.5 mg, 0.260 mmol, 97% yield) as a pale yellow fluffy solid. ^{1}H NMR (400 MHz, CD_3OD) δ_H ppm 7.96 (d, *J*=8.8 Hz, 2 H, 11-CH), 7.82 (d, *J*=8.8 Hz, 2 H, 10-CH), 7.56 - 7.63 (m, 1 H, 19-CH), 7.40 - 7.47 (m, 1 H, 16-CH), 7.18 - 7.27 (m, 2 H, 17-CH,18-CH), 4.18 (s, 2 H, 7-CH_2), 4.12 (s, 2 H, 2-CH_2), 3.72 - 3.81 (m, 8 H, (3-6)-CH_2), 1.50 (s, 9 H, 22-CH_3). ^{13}C NMR (101 MHz, CD_3OD) δ_C ppm 174.2 (C1), 171.5 (C8), 167.9 (C13), 156.4 (C20), 142.8 (C9), 133.2 (C15), 131.8 (C14), 131.0 (C12), 129.7 (C11), 127.5 (C17), 127.3 (C18), 126.4 (C19), 125.7 (C16), 121.1 (C10), 81.9 (C21), 72.2 (C3), 71.8 (C4), 71.7 (C5), 71.6 (C7), 71.4 (C6), 69.2 (C2), 28.8 (C22). HRMS (ESI) m/z: $[M+H]^+$ calculated for $C_{26}H_{34}N_3O_9$: 532.2295, found 532.2294.

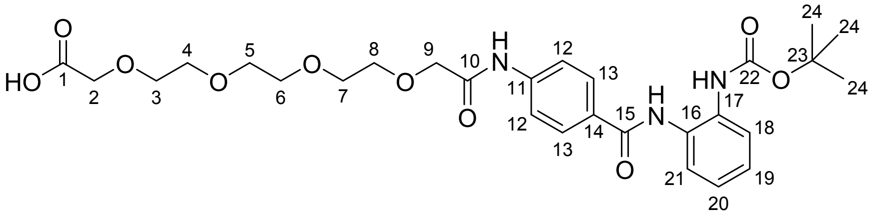

14-((4-((2-((tert-butoxycarbonyl)amino)phenyl)carbamoyl)phenyl)amino)-14-oxo-3,6,9,12-tetraoxatetradecanoic acid (53l): Following general method F, benzyl ester hydrogenolysis of **52l (**294 mg, 0.44 mmol) was performed to afford a crude yellow oil (273.1 mg). The crude product was purified by column chromatography (100% EtOAc followed by 1-10% MeOH in DCM) to afford **53l** (194 mg, 0.33 mmol, 75 %) as a pale yellow oil. ^{1}H NMR (400 MHz, $CDCl_3$) δ ppm 9.69 (br s, 1 , 11-NH), 9.38 (br s, 1 H, 16-NH), 7.82 (d, *J*=8.8 Hz, 2 H, 13-CH), 7.51 - 7.69 (m, 4 H, 12-CH,19-CH,17-NH), 7.46 (dd, *J*=7.8, 1.3 Hz, 1 H, 18-CH), 7.04 - 7.18 (m, 2 H, 19-CH,20-CH), 4.09 (s, 2 H, 9-CH_2), 3.90 (s, 2 H, 2-CH_2), 3.51 - 3.65 (m, 12 H, (3-8)-CH_2), 1.47 (s, 9 H, 24-CH_3). ^{13}C NMR (101 MHz, $CDCl_3$) δ ppm 175.4 (C1), 169.3 (C10), 166.1 (C15), 154.4 (C22), 141.1 (C11), 131.1 (C16), 130.1 (C17), 129.3 (C14), 128.5 (C13), 126.0 (C19/20), 125.6 (C21), 125.0 (C19/20), 124.4 (C18), 119.5 (C12), 80.9 (C23), 70.2 (alkoxy CH_2), 70.0 (alkoxy CH_2), 69.6 (alkoxy CH_2), 69.5 (alkoxy CH_2), 69.45 (alkoxy CH_2), 69.4 (alkoxy CH_2), 69.35 (alkoxy CH_2), 69.3 (alkoxy CH_2), 28.3 (C24). HRMS (ESI) m/z: [M+H]+ calculated for $C_{28}H_{38}O_{10}N_3$: 576.2557, found 576.2552.

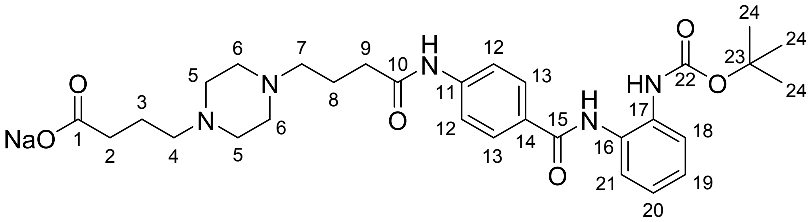

Sodium 4-(4-(4-((4-((2-((tert-butoxycarbonyl)amino)phenyl)carbamoyl)phenyl)amino)-4-oxobutyl)piperazin-1-yl)butanoate (53m): To a solution of **52m** (121.1 mg, 0.203 mmol) in THF/Water (1:2,

12 mL) was added NaOH (8.9 mg, 0.224 mmol) and the resultant solution stirred at room temperature overnight. The reaction mixture was concentrated *in vacuo* to afford **53m** (119.2 mg, 0.198 mmol, 97% yield) as a pale yellow solid. ^{1}H NMR (400 MHz, DMSO-d_6) δ_H ppm 7.93 (d, J=8.7 Hz, 2 H, 13-CH), 7.68 - 7.73 (m, 1 H, 21-CH), 7.56 - 7.65 (m, 3 H, 12-CH, 18-CH), 6.81 - 6.97 (m, 2 H, 19-CH,20-CH), 2.23 - 2.43 (m, 12 H, (4,5,7,9)-CH_2), 2.18 (t, J=7.2 Hz, 2 H, 4-CH_2), 1.85 (t, J=7.3 Hz, 2 H, 2-CH_2), 1.69 - 1.78 (m, 2 H, 8-CH_2), 1.52 - 1.60 (m, 2 H, 3-CH_2), 1.44 (s, 9 H, 24-CH_3). ^{13}C NMR (101 MHz, DMSO-d_6) δ_C ppm ^{13}C NMR (101 MHz, DMSO-d_6) δ ppm 177.3 (C1), 171.4 (C10), 165.0 (C15), 153.2 (C22), 141.0 (C11), 131.9 (C16/17), 130.4 (C16/17), 128.4 (C13), 128.3 (C14), 124.4 (C21), 122.7 (C19/20), 122.6 (C19/20), 118.0 (C12), 117.9 (C18), 78.7 (C23), 58.4 (C4), 57.4 (C7), 52.9 (C5/6), 52.8 (C5/6), 36.2 (C2), 34.6 (C9), 28.1 (C24), 23.7 (C3), 22.2 (C8). HRMS (ESI) m/z: $[M+H]^+$ calculated for $C_{30}H_{42}N_5O_6$: 568.3135, found 568.3129.

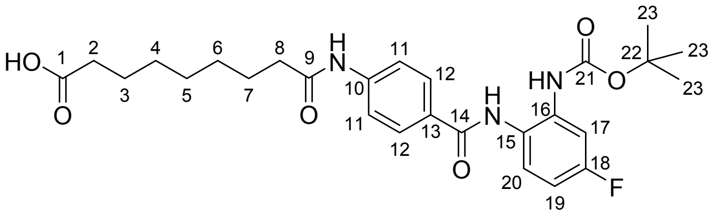

9-((4-((2-((tert-butoxycarbonyl)amino)-4-fluorophenyl)carbamoyl)phenyl)amino)-9-oxononanoic acid (53n): Following general method F, benzyl ester hydrogenolysis of **52n (**82.5 mg, 0.136 mmol) was performed to afford **53n** (70.5 mg, 0.135 mmol, 99% yield) as a pale purple/brown solid. ^{1}H NMR (400 MHz, CD_3CN) δ_H ppm 8.84 (br s, 1 H, 15-NH), 8.63 (br s, 1 H, 10-NH), 7.89 (d, J=8.6 Hz, 2 H, 12-CH), 7.69 (d, J=8.6 Hz, 2 H, 11-CH), 7.64 (br s, 1 H, 16-NH), 7.50 (dd, J_{HF}=10.9, J_{HH}=2.9 Hz, 1 H, 17-CH), 7.40 (dd, J_{HH}=8.9, J_{HF}=6.1 Hz, 1 H, 20-CH), 6.87 (ddd, J_{HH}=8.9,2.9 J_{HF}=8.1 Hz, 1 H, 19-CH), 2.33 (t, J=7.5 Hz, 2 H, 8-CH_2), 2.26 (t, J=7.4 Hz, 2 H, 2-CH_2), 1.63 (quin, J=7.0 Hz, 2 H, 7-CH_2), 1.55 (quin, J=7.2 Hz, 2 H, 3-CH_2), 1.46 (s, 9 H, 23-CH_3), 1.32 (m, J=3.6 Hz, 6 H, (4-6)-CH_2). CO_2H not visible. ^{13}C NMR (101 MHz, CD_3CN) δ_C ppm 175.4 (C1), 173.2 (C9), 166.9 (C14), 161.6 (d, J_{CF}=241.7 Hz, C18), 154.4 (C21), 143.7 (C10), 135.7 (d, J_{CF}=11.4 Hz, C16), 129.6 (C12), 129.4 (C13), 128.9 (d, J_{CF}=9.5 Hz, C20), 126.0 (d, J_{CF}=2.5 Hz, C15), 119.7 (C11), 111.5 (d, J_{CF}=22.9 Hz, C19), 110.4 (d, J_{CF}=27.1 Hz, C17), 81.6 (C22), 37.9 (C8), 34.3 (C2), 29.8 (alkyl CH_2), 29.7 (alkyl CH_2), 29.6 (alkyl CH_2), 28.5 (C23), 26.1 (C7), 25.6 (C3). ^{19}F NMR (376 MHz, CD_3CN) δ_F ppm -116.5. HRMS (ESI) m/z: $[M+H]^+$ calculated for $C_{27}H_{35}FN_3O_6$: 516.2510, found 516.2505.

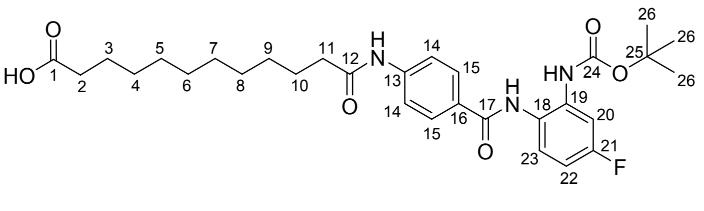

12-((4-((2-((tert-butoxycarbonyl)amino)-4-fluorophenyl)carbamoyl)phenyl)amino)-12-oxododecanoic acid (53o): Following general method F, benzyl ester hydrogenolysis of **52o** (219.6 mg, 0.339 mmol) was performed to afford **53o** (192.9 mg, 0.266 mmol, 98% yield) as a pale purple/brown solid. ^{1}H NMR (400 MHz, DMSO-d_6) δ_H ppm 11.94 (br s, 1 H, CO_2H), 10.16 (s, 1 H, 13-NH), 9.70 (s, 1 H, 18-NH), 8.75 (s, 1 H, 19-NH), 7.91 (d, J=8.7 Hz, 2 H, 15-CH), 7.73 (d, J=8.7 Hz, 2 H, 14-CH), 7.50 (dd, J_{HF}=11.1, J_{HH}=2.9 Hz, 1 H, 20-CH), 7.45 (dd, J_{HH}=8.9, J_{HF}=6.2 Hz, 1 H, 23-CH), 6.96 (ddd, J_{HF}=9.0, J_{HH}=8.9, 2.9 Hz, 1 H, 22-CH),

2.34 (t, J=7.4 Hz, 2 H, 11-CH_2), 2.18 (t, J=7.3 Hz, 2 H, 2-CH_2), 1.55 - 1.65 (m, 2 H, 10-CH_2), 1.46 - 1.52 (m, 2 H, 3-CH_2), 1.45 (s, 9 H, 26-CH_3), 1.22 - 1.31 (m, 12 H, (4-9)-CH_2). ^{13}C NMR (101 MHz, DMSO-d_6) δ_C ppm 174.5 (C1), 171.8 (C12), 165.1 (C17), 159.4 (d, J_{CF}=240.5 Hz, C21), 153.0 (C24), 142.5 (C13), 134.1 (d, J_{CF}=11.3 Hz, C19), 128.7 (C15), 128.2 (d, J_{CF}=9.3 Hz, C23), 128.1 (C16), 125.1 (d, J_{CF}=2.9 Hz, C18), 118.1 (C14), 110.1 (d, J_{CF}=22.1 Hz, C22), 109.1 (d, J_{CF}=25.7 Hz, C20), 80.1 (C25), 36.5 (C11), 33.7 (C2), 28.9 (2 x alkyl CH_2), 28.8 (alkyl CH_2), 28.7 (alkyl CH_2), 28.7 (alkyl CH_2), 28.6 (alkyl CH_2), 28.0 (C26), 25.0 (C10), 24.5 (C3). ^{19}F NMR (376 MHz, DMSO-d_6) δ_F ppm -115.7. HRMS (ESI) m/z: $[M+H]^+$ calculated for $C_{30}H_{41}FN_3O_6$: 558.2979, found 558.2974.

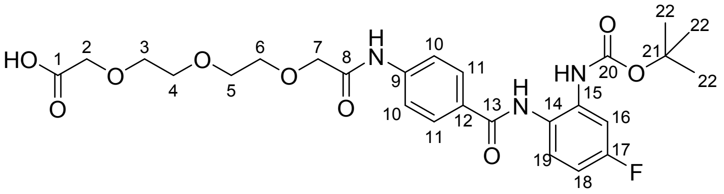

2-(2-(2-(2-((4-((2-((tert-butoxycarbonyl)amino)-4-fluorophenyl)carbamoyl)phenyl)amino)-2-oxoethoxy)ethoxy)ethoxy)acetic acid (53p): Following general method F, benzyl ester hydrogenolysis of **52p** (84.8 mg, 0.3133mmol) was performed to afford **53p** (73.9 mg, 0.132 mmol, 99% yield) as an off-white solid. ^{1}H NMR (400 MHz, CD_3CN) δ_H ppm 8.91 (br s, 1 H, 14-NH), 8.73 (br s, 1 H, 9-NH), 7.84 (d, J=8.7 Hz, 2 H, 11-CH), 7.68 (d, J=8.7 Hz, 2 H, 10-CH), 7.53 (br s, 1 H, 15-NH), 7.42 (dd, J_{HF}=10.9, J_{HH}=2.9 Hz, 1 H, 16-CH), 7.33 (dd, J_{HH}=8.9, J_{HF}=6.0 Hz, 1 H, 19-CH), 6.80 (ddd, J_{HH}=8.9, 2.9, J_{HF}=8.1Hz, 1 H, 18-CH), 4.02 (s, 2 H, 7-CH_2), 4.00 (s, 2 H, 2-CH_2), 3.58 - 3.67 (m, 8 H, (3-6)-CH_2), 1.38 (s, 9 H, 22-CH_3). CO_2H not visible. ^{13}C NMR (101 MHz, CD_3CN) δ_C ppm 172.4 (C1), 170.2 (C8), 166.9 (C13), 161.6 (d, J_{CF}=241.9 Hz, C17), 154.4 (C20), 142.4 (C9), 135.7 (d, J_{CF}=11.6 Hz, C15), 130.2 (C12), 129.7 (C11), 129.0 (d, J_{CF}=9.7 Hz, C19), 125.9 (d, J_{CF}=3.1 Hz, C14), 120.4 (C10), 111.5 (d, J_{CF}=22.9 Hz, C18), 110.4 (d, J_{CF}=27.3 Hz, C16), 81.7 (C21), 71.8 (C6), 71.4 (C3), 71.1 (C7), 70.9 (C4), 70.7 (C5), 68.7 (C2), 28.5 (C22). ^{19}F NMR (376 MHz, CD_3CN) δ_F ppm -116.3. HRMS (ESI) m/z: $[M+H]^+$ calculated for $C_{26}H_{33}FN_3O_9$: 550.2201, found 550.2205.

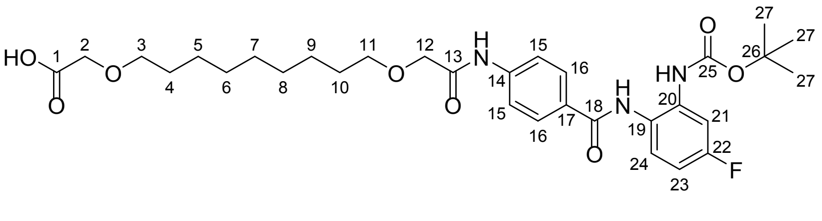

2-((9-(2-((4-((2-((tert-butoxycarbonyl)amino)-4-fluorophenyl)carbamoyl)phenyl)amino)-2-oxoethoxy)nonyl)oxy)acetic acid (53q): Following general method F, benzyl ester hydrogenolysis of **52q** (114.0 mg, 0.164 mmol) was performed to afford **53q** (99.8 mg, 0.164 mmol, 100% yield) as a yellow/brown tar. ^{1}H NMR (400 MHz, CD_3CN) δ_H ppm 8.81 (s, 1 H, 19-NH), 8.76 (s, 1 H, 14-NH), 7.93 (d, J=8.7 Hz, 2 H, 16-CH), 7.77 (d, J=8.7 Hz, 2 H, 15-CH), 7.61 (br s, 1 H, 20-NH), 7.51 (dd, J_{HF}=10.9, J_{HH}=2.9 Hz, 1 H, 21-CH), 7.43 (dd, J_{HH}=8.9, J_{HF}=6.1 Hz, 1 H, 24-CH), 6.90 (ddd, J_{HH}=8.9, 2.9, J_{HF}=7.7 Hz, 1 H, 23-CH), 4.03 (s, 2 H, 12-CH_2), 3.99 (s, 2 H, 2-CH_2), 3.58 (t, J=6.6 Hz, 2 H, 11-CH_2), 3.47 (t, J=6.6 Hz, 2 H, 3-CH_2), 1.62 - 1.70 (m, 2 H, 10-CH_2), 1.51 - 1.58 (m, 2 H, 4-CH_2), 1.47 (s, 9 H, 27-CH_3), 1.30 - 1.40 (m, 10 H, (5-9)-CH_2). ^{13}C NMR (101 MHz, CD_3CN) δ_C ppm 172.2 (C1), 170.0 (C13), 166.9 (C18), 161.6 (d, J_{CF}=241.9 Hz, C22),

154.4 (C25), 142.4 (C14), 135.7 (d, J_{CF}=11.4 Hz, C20), 130.1 (C17), 129.6 (C16), 128.9 (d, J_{CF}=9.7 Hz, C24), 125.9 (d, J_{CF}=3.1 Hz, C19), 120.4 (C15), 111.5 (d, J_{CF}=22.7 Hz, C23), 110.4 (d, J_{CF}=26.9 Hz, C21), 81.6 (C26), 72.8 (C11), 72.3 (C3), 71.2 (C12), 68.4 (C2), 30.35 (C4), 30.3 (C10), 30.2 (C6/7/8), 30.15 (C6/7/8), 30.1 (C6/7/8), 28.5 (C27), 26.8 (C9), 26.8 (C5). ^{19}F NMR (376 MHz, CD_3CN) δ_F ppm -116.3. HRMS (ESI) m/z: $[M+H]^+$ calculated for $C_{31}H_{43}FN_3O_8$: 604.3034, found 604.3046.

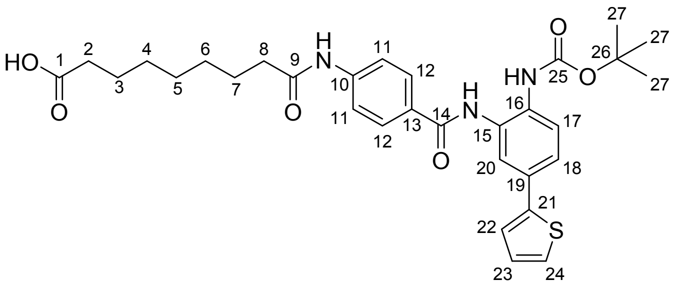

9-((4-((2-((tert-butoxycarbonyl)amino)-5-(thiophen-2-yl)phenyl)carbamoyl)phenyl)amino)-9-oxononanoic acid (53r): To a solution of **52r** (84.3 mg, 0.126 mmol) in MeOH (10 mL) was added NaOH (160 mg) until solution reached pH 10 (0.4 M NaOH in MeOH), then the resultant solution stirred at room temperature for 4 hours. The reaction mixture was concentrated *in vacuo*, then dissovled in water (10 mL) and washed with EtOAC (20 mL). The aqueous layer was acidified to pH2 with HCl (1M) and the product was extracted with EtOAc (2 x 20 mL), then the combined organic layers dried over Na_2SO_4, filtered and concentrated *in vacuo* to afford a yellow solid. The crude product was purified by column chromatography (10-100% EtOAc in hexane) to afford **53r** (40.9 mg, 0.068 mmol, 54% yield) as a pale yellow solid. ^{1}H NMR (400 MHz, DMSO-d_6) δ_H ppm 11.98 (br s, 1 H, 1-CO_2H), 10.18 (s, 1 H, 10-NH), 9.83 (s, 1 H, 15-NH), 8.73 (br s, 1 H, 16-NH), 7.94 (d, J=8.8 Hz, 2 H, 12-CH), 7.82 (d, J=2.1 Hz, 1 H, 20-CH), 7.75 (d, J=8.8 Hz, 2 H, 11-CH), 7.60 (d, J=8.5 Hz, 1 H, 17-CH), 7.49 - 7.54 (m, 2 H, 18,24-CH), 7.45 (dd, J=3.6, 1.0 Hz, 1 H, 22-CH), 7.13 (dd, J=5.0, 3.6 Hz, 1 H, 23-CH), 2.35 (t, J=7.4 Hz, 2 H, 8-CH_2), 2.20 (t, J=7.4 Hz, 2 H, 2-CH_2), 1.60 (quin, J=6.6 Hz, 2 H, 7-CH_2), 1.47 - 1.54 (m, 2 H, 3-CH_2), 1.46 (s, 9 H, 27-CH_3), 1.26 - 1.34 (m, 6 H, (4-6)-CH_2). ^{13}C NMR (101 MHz, DMSO-d_6) δ_C ppm 174.5 (C1), 171.8 (C9), 165.0 (C14), 153.3 (C25), 142.7 (C10), 142.6 (C21), 131.1 (C16), 130.1 (C15), 129.6 (C19), 128.6 (C12), 128.5 (C23), 128.1 (C13), 125.4 (C24), 124.2 (C17), 123.4 (C22), 122.8 (C20), 122.5 (C18), 118.2 (C11), 79.8 (C26), 36.4 (C8), 33.6 (C2), 28.5 (C5/6), 28.45 (C5/6), 28.4 (C4), 28.0 (C27), 24.9 (C7), 24.5 (C3). HRMS (ESI) m/z: $[M+H]^+$ calculated for $C_{31}H_{38}N_3O_6S$: 580.2481, found 580.2473.

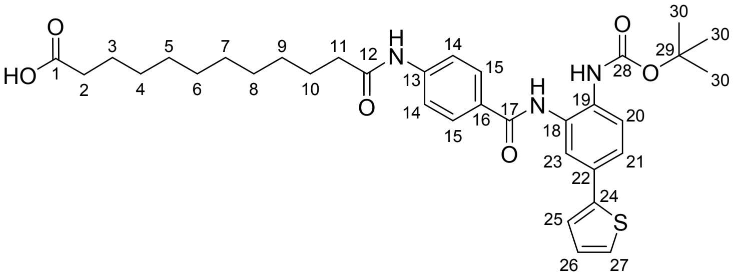

12-((4-((2-((tert-butoxycarbonyl)amino)-5-(thiophen-2-yl)phenyl)carbamoyl)phenyl)amino)-12-oxododecanoic acid (53s): To a solution of **52s** (62.7 mg, 0.088 mmol) in MeOH (10 mL) was added NaOH (160 mg) until solution reached pH 10 (0.4 M NaOH in MeOH), then the resultant solution stirred at room

temperature for 4 hours. The reaction mixture was concentrated *in vacuo*, then dissovled in water (10 mL) and washed with EtOAC (20 mL). The aqueous layer was acidified to pH2 with HCl (1M) and the product was extracted with EtOAc (2 x 20 mL), then the combined organic layers dried over Na_2SO_4, filtered and concentrated *in vacuo* to afford a yellow/brown solid (56.3 mg). The crude product was purified by column chromatography (10-100% EtOAc in hexane) to afford **53s** (23.9 mg, 0.038 mmol, 44% yield) as a pale yellow solid. ^{1}H NMR (400 MHz, DMSO-d_6) δ_H ppm 11.88 (br s, 1 H, 1-CO_2H), 10.17 (s, 1 H, 13-NH), 9.83 (s, 1 H, 18-NH), 8.73 (br s, 1 H, 19-NH), 7.94 (d, *J*=8.7 Hz, 2 H, 15-CH), 7.82 (d, *J*=2.1 Hz, 1 H, 23-CH), 7.75 (d, *J*=8.7 Hz, 2 H, 14-CH), 7.60 (d, *J*=8.5 Hz, 1 H, 20-CH), 7.48 - 7.55 (m, 2 H, 21,27-CH), 7.45 (dd, *J*=3.6, 0.8 Hz, 1 H, 25-CH), 7.13 (dd, *J*=5.0, 3.6 Hz, 1 H, 26-CH), 2.35 (t, *J*=7.4 Hz, 2 H, 11-CH_2), 2.18 (t, *J*=7.3 Hz, 2 H, 2-CH_2), 1.56 - 1.66 (m, 2 H, 10-CH_2), 1.43 - 1.50 (m, 11 H, 3-CH_2, 30-CH_3) 1.23 - 1.31 (m, 12 H, (4-9)-CH_2). ^{13}C NMR (101 MHz, DMSO-d_6) δ_C ppm 174.5 (C1), 171.8 (C12), 165.0 (C17), 153.3 (C28), 142.7 (C13), 142.6 (C24), 131.1 (C19), 130.2 (C18), 129.7 (C22), 128.6 (C15), 128.5 (C26), 128.1 (C16), 125.4 (C27), 124.2 (C20), 123.4 (C25), 122.8 (C23), 122.5 (C21), 118.2 (C14), 79.8 (C29), 36.5 (C11), 33.7 (C2), 28.9 (2x alkyl CH_2), 28.8 (alkyl CH_2), 28.7 (alkyl CH_2), 28.7 (alkyl CH_2), 28.6 (alkyl CH_2), 28.0 (C30), 25.0 (C10), 24.5 (C3). HRMS (ESI) m/z: $[M+H]^+$ calculated for $C_{34}H_{44}N_3O_6S$: 622.2951, found 622.2950.

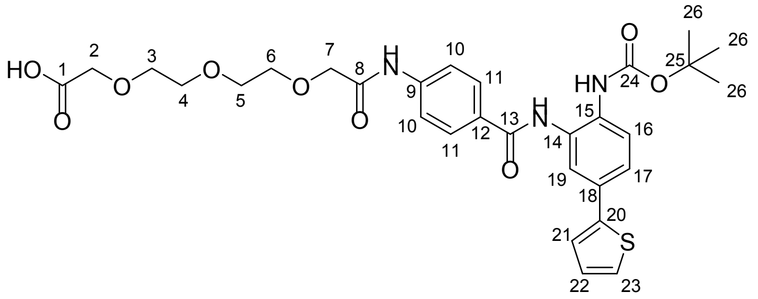

2-(2-(2-(2-((4-((2-((tert-butoxycarbonyl)amino)-5-(thiophen-2-yl)phenyl)carbamoyl)phenyl)amino)-2-oxoethoxy)ethoxy)ethoxy)acetic acid (53t): To a solution of **52t** (111.6 mg, 0.159 mmol) in MeOH (10 mL) was added NaOH (160 mg) until solution reached pH 10 (0.4 M NaOH in MeOH), then the resultant solution stirred at room temperature for 4 hours. The reaction mixture was concentrated *in vacuo*, then dissovled in water (10 mL) and washed with EtOAC (20 mL). The aqueous layer was acidified to pH2 with HCl (1M) and the product was extracted with EtOAc (2 x 20 mL), then the combined organic layers dried over Na_2SO_4, filtered and concentrated *in vacuo* to afford **53t** (96.7 mg, 0.154 mmol, 87% yield) as a white solid. ^{1}H NMR (400 MHz, CD_3CN) δ_H ppm 9.02 (br s, 2 H, 9,14-NH), 7.96 (d, J=8.8 Hz, 2 H, 11-CH), 7.88 (d, J=2.1 Hz, 1 H, 19-CH), 7.81 (d, J=8.8 Hz, 2 H, 10-CH), 7.59 (d, J=8.5 Hz, 1 H, 16-CH), 7.56 (br s, 1 H, 15-NH), 7.52 (dd, J=8.5, 2.1 Hz, 1 H, 17-CH), 7.36 - 7.40 (m, 2 H, 21,23-CH), 7.11 (dd, J=5.0, 3.7 Hz, 1 H, 22-CH), 4.10 (s, 2 H, 7-CH_2), 4.09 (s, 2 , 2-CH_2), 3.74 - 3.78 (m, 2 H, 6-CH_2), 3.69 - 3.73 (m, 6 H, (3-5)-CH_2), 1.48 (s, 9 H, 26-CH_3). CO_2H not visible. ^{13}C NMR (101 MHz, CD_3CN) δ_C ppm 172.2 (C1), 170.2 (C8), 166.6 (C13), 155.0 (C24), 144.1 (C22), 142.5 (C9), 132.2 (C15), 131.8 (C18), 131.4 (C14), 130.3 (C12), 129.6 (C11), 129.4 (C22), 126.2 (C23), 125.5 (C16), 124.5 (C21), 124.3 (C17), 123.8 (C19), 120.4 (C10), 81.5 (C25), 71.9 (C6), 71.5 (C3), 71.2 (C7), 71.0 (C4), 70.8 (C5), 68.8 (C2), 28.6 (C26). HRMS (ESI) m/z: $[M+H]^+$ calculated for $C_{30}H_{36}N_3O_9S$: 614.2172, found 614.2161.

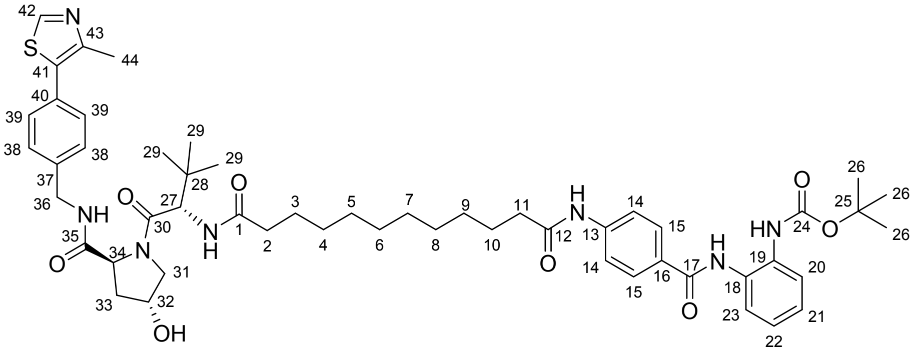

Tert-butyl(2-(4-(12-(((S)-1-((2S,4R)-4-hydroxy-2-((4-(4-methylthiazol-5-yl)benzyl)carbamoyl)pyrrolidin-1-yl)-3,3-dimethyl-1-oxobutan-2-yl)amino)-12-oxododecanamido)benzamido)phenyl)carbamate (54a): Following general method G, **54a** was obtained from **53a** (65.5 mg, 0.121 mmol) and **VH_032 amine** (50.0 mg, 0.099 mmol). The crude product was purified by column chromatography (0-5% MeOH in DCM) to afford **54a** (61.9 mg, 0.064 mmol, 64% yield) as a white solid. ^{1}H NMR (400 MHz, CD_3OD) δ_H ppm 8.86 (s, 1 H, 42-CH), 7.93 (d, *J*=8.8 Hz, 2 H, 15-CH), 7.73 (d, *J*=8.8 Hz, 2 H, 14-CH), 7.56 - 7.63 (m, 1 H, 23-CH), 7.44 - 7.47 (m, 2 H, 39-CH), 7.41 - 7.43 (m, 1 H, 20-CH), 7.38 - 7.41 (m, 2 H, 38-CH), 7.15 - 7.27 (m, 2 H, 21,22-CH), 4.61 - 4.66 (m, 1 H, 27-CH), 4.55 - 4.60 (m, 1 H, 34-CH), 4.50 - 4.55 (m, 1 H, 36-CH), 4.47 - 4.50 (m, 1 H, 32-CH), 4.30 - 4.39 (m, 1 H, 36-CH), 3.84 - 3.94 (m, 1 H, 31-CH), 3.76 - 3.82 (m, 1 H, 31-CH), 2.46 (s, 3 H, 44-CH_3), 2.39 (t, *J*=7.5 Hz, 2 H, 11-CH_2), 2.17 - 2.32 (m, 3 H, 2-CH_2 ,33-CH), 2.03 - 2.11 (m, 1 H, 33-CH), 1.65 - 1.75 (m, 2 H, 10-CH_2), 1.59 (m, 2 H, 3-CH_2), 1.49 (s, 9 H, 26-CH_3), 1.29 - 1.37 (m, 12 H, (4-9)-CH_2), 1.03 (s, 9 H, 29-CH_3). ^{13}C NMR (101 MHz, CD_3OD) δ_C ppm 176.2 (C1), 175.1 (C12), 174.6 (C35), 172.5 (C30), 167.9 (C17), 156.4 (C24), 153.0 (C42), 149.1 (C43), 143.9 (C13), 140.4 (C37), 133.6 (C41), 133.2 (C19), 131.8 (C18), 131.6 (C40), 130.5 (C38), 130.3 (C16), 129.7 (C15), 129.1 (C39), 127.5 (C21/22), 127.3 (C23), 126.4 (C21/22), 125.7 (C20), 120.4 (C14), 81.9 (C25), 71.2 (C32), 61.0 (C34), 59.1 (C27), 58.2 (C31), 43.8 (C36), 39.0 (C33), 38.2 (C11), 36.8 (C2), 36.7 (C28), 30.7 (alkyl CH_2), 30.6 (alkyl CH_2), 30.55 (alkyl CH_2), 30.5 (alkyl CH_2), 30.4 (2x alkyl CH_2), 28.8 (C26), 27.2 (C29), 27.1 (C3), 26.9 (C10), 16.0 (C44). HRMS (ESI) m/z: $[M+H]^+$ calculated for $C_{52}H_{70}N_7O_8S$: 952.5007, found 952.5009.

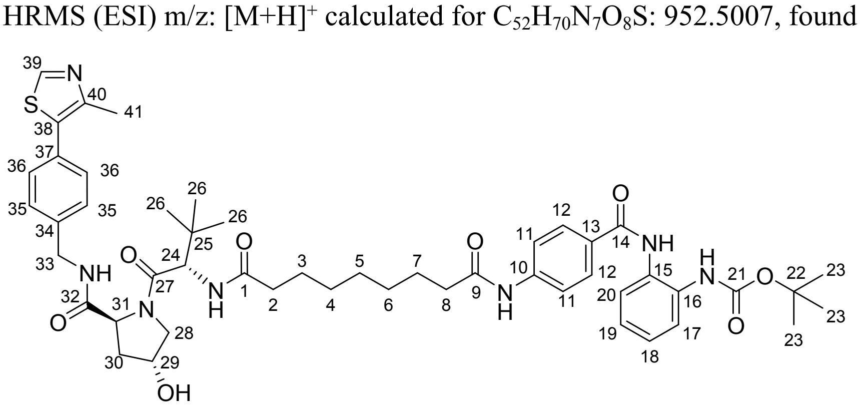

Tert-butyl (2-(4-(9-(((S)-1-((2S,4R)-4-hydroxy-2-((4-(4-methylthiazol-5-yl)benzyl)carbamoyl)pyrrolidin-1-yl)-3,3-dimethyl-1-oxobutan-2-yl)amino)-9-oxononanamido)benzamido)phenyl)carbamate (54b): Following general method G, **54b** was obtained from **43b** (59.9 mg, 0.120 mmol) and **VH_032 amine** (50.0 mg, 0.099 mmol). The crude product was purified by column chromatography (0-5% MeOH in DCM) to afford **54b** (76.1 mg, 0.083 mmol, 84% yield) as a white solid. ^{1}H NMR (400 MHz, CD_3OD) δ_H ppm 8.85 (s, 1 H,

39-CH), 7.92 (d, *J*=8.7 Hz, 2 H, 12-CH), 7.72 (d, *J*=8.7 Hz, 2 H, 11-CH), 7.54 - 7.64 (m, 1 H, 20-CH), 7.41 - 7.46 (m, 3 H, 17,36-CH), 7.39 (d, *J*=8.4 Hz, 2 H, 35-CH), 7.15 - 7.26 (m, 2 H, 18,19-CH), 4.62 - 4.66 (m, 1 H, 24-CH), 4.55 - 4.61 (m, 1 H, 31-CH), 4.49 - 4.54 (m, 1 H, 33-CH_2), 4.47 - 4.49 (m, 1 H, 29-CH), 4.30 - 4.39 (m, 1 H, 33-CH_2), 3.87 - 3.93 (m, 1 H, 28-CH), 3.76 - 3.82 (m, 1 H, 28-CH), 2.45 (s, 3 H, 41-CH_3), 2.39 (t, *J*=7.5 Hz, 2 H, 8-CH_2), 2.17 - 2.35 (m, 3 H, 2-CH_2,30-CH), 2.02 - 2.13 (m, 1 H, 30-CH_2), 1.69 (quin, *J*=7.5 Hz, 2 H, 7-CH_2), 1.62 (quin, *J*=7.0 Hz, 2 H, 3-CH_2), 1.48 (s, 9 H, 23-CH_3), 1.31 - 1.42 (m, 6 H, (4-6)-CH_2), 1.03 (s, 9 H, 26-CH_3). ^{13}C NMR (101 MHz, CD_3OD) δ_C ppm 176.1 (C1), 175.0 (C9), 174.6 (C32), 172.5 (C27), 167.8 (C14), 156.4 (C21), 152.9 (C39), 149.1 (C40), 143.9 (C10), 140.4 (C34), 133.5 (C38), 133.1 (C16), 131.8 (C15), 131.6 (C37), 130.5 (C35), 130.3 (C13), 129.7 (C12), 129.1 (C36), 127.5 (C18/19), 127.3 (C20), 126.4 (C18/19), 125.7 (C17), 120.4 (C11), 81.8 (C22), 71.2 (C29), 60.9 (C31), 59.1 (C24), 58.1 (C28), 43.8 (C33), 39.0 (C30), 38.2 (C8), 36.8 (C2), 36.7 (C25), 30.3 (CH_2), 30.25 (CH_2), 30.2 (CH_2), 28.8 (C23), 27.2 (C26), 27.1 (C3), 26.8 (C7), 16.0 (C41). HRMS (ESI) m/z: $[M+H]^+$ calculated for $C_{49}H_{64}N_7O_8S$: 910.4537, found 910.4529.

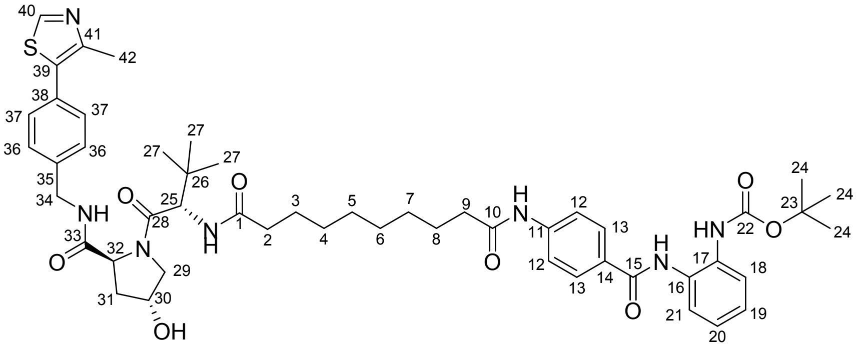

Tert-butyl (2-(4-(10-(((S)-1-((2S,4R)-4-hydroxy-2-((4-(4-methylthiazol-5-yl)benzyl)carbamoyl)pyrrolidin-1-yl)-3,3-dimethyl-1-oxobutan-2-yl)amino)-10-oxodecanamido)benzamido)phenyl)carbamate (54c): Following general method G, **54c** was obtained from **53c** (50.1 mg, 0.098 mmol) and **VH_032 amine** (40.0 mg, 0.080 mmol). The crude product was purified by column chromatography (1-10% MeOH in DCM) to afford **54c** (68.5 mg, 0.074 mmol, 93% yield) as a white solid. ^1H NMR (400 MHz, CD_3OD) δ_H ppm 8.85 (s, 1 H, 40-CH), 8.64 (t, *J*=6.0 Hz, 1 H, 33-NH), 7.93 (d, *J*=8.8 Hz, 2 H, 13-CH), 7.80 (d, *J*=9.0 Hz, 1 H, 1-NH), 7.73 (d, *J*=8.8 Hz, 2 H, 12-CH), 7.56 - 7.62 (m, 1 H, 21-CH), 7.41 - 7.47 (m, 3 H, 18-CH, 37-CH), 7.37 - 7.41 (m, 2 H, 36-CH), 7.14 - 7.27 (m, 2 H, 19-CH,20-CH), 4.64 (d, *J*=9.0 Hz, 1 H, 25-CH), 4.55 - 4.61 (m, 1 H, 32-CH), 4.46 - 4.55 (m, 2 H, 30-CH,34-CH), 4.30 - 4.39 (m, 1 H, 34-CH), 3.86 - 3.94 (m, 1 H29-CH), 3.75 - 3.83 (m, 1 H, 29-CH), 2.46 (s, 3 H, 42-CH_3), 2.39 (t, *J*=7.5 Hz, 2 H, 9-CH_2), 2.16 - 2.33 (m, 3 H, 2-CH_2,31-CH), 2.02 - 2.13 (m, 1 H, 31-CH), 1.70 (quin, *J*=7.0 Hz, 2 H, 8-CH_2), 1.55 - 1.65 (m, 2 H, 3-CH_2), 1.49 (s, 9 H, 24-CH_3), 1.30 - 1.40 (m, 8 H, (4-7)-CH_2), 1.03 (s, 9 H, 27-CH_3). ^{13}C NMR (101 MHz, CD_3OD) δ_C ppm 176.1 (C1), 175.1 (C10), 174.6 (C33), 172.5 (C28), 167.9 (C15), 156.4 (C22), 152.9 (C40), 149.1 (C41), 143.9 (C11), 140.4 (C35), 133.6 (C39), 133.2 (C17), 131.8 (C16), 131.6 (C38), 130.5 (C36), 130.3 (C14), 129.7 (C13), 129.1 (C37), 127.5 (C19/20), 127.3 (C21), 126.4 (C19/20), 125.7 (C18), 120.4 (C12), 81.8 (C23), 71.2 (C30), 61.0 (C32), 59.1 (C25), 58.1 (C29), 43.8 (C34), 39.0 (C31), 38.2 (C9), 36.8 (C2), 36.7 (C26), 30.6 (alkyl CH_2), 30.5 (alkyl CH_2), 30.45 (alkyl CH_2), 30.4 (alkyl CH_2), 28.8 (C24), 27.2 (C27), 27.1 (C3), 26.8 (C8), 16.0 (C42). HRMS (ESI) m/z: $[M+H]^+$ calculated for $C_{50}H_{66}N_7O_8S$: 924.4694, found 924.4667.

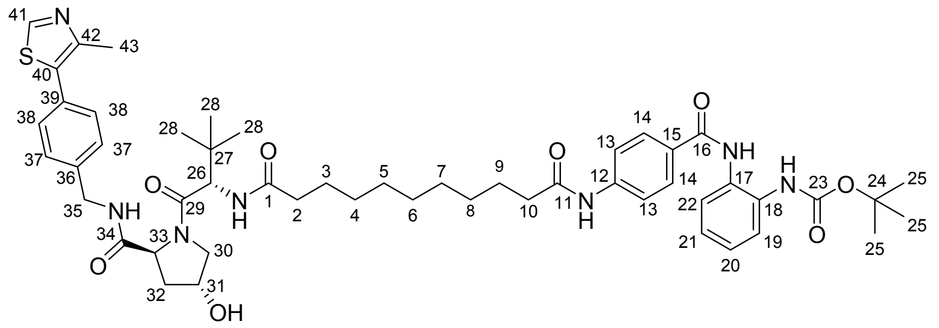

Tert-butyl (2-(4-(11-(((S)-1-((2S,4R)-4-hydroxy-2-((4-(4-methylthiazol-5- yl)benzyl)carbamoyl)pyrrolidin-1-yl)-3,3-dimethyl-1-oxobutan-2-yl)amino)-11-oxoundecanamido)benzamido)phenyl)carbamate (54d): Following general method G, **54d** was obtained from **53d** (51.4 mg, 0.098 mmol) and **VH_032 amine** (40.0 mg, 0.080 mmol). The crude product was purified by column chromatography (1-10% MeOH in DCM) to afford **54d** (73.0 mg, 0.078 mmol, 97% yield) as a white solid. ^{1}H NMR (400 MHz, CD_3OD) δ_H ppm 8.86 (s, 1 H, 41-CH), 7.93 (d, J=8.6 Hz, 2 H, 14-CH), 7.79 (d, J=8.9 Hz, 1 H, 1-NH), 7.73 (d, J=8.6 Hz, 2 H, 13-CH), 7.59 (dd, J=7.7, 1.6 Hz, 1 H, 22-CH), 7.41 - 7.47 (m, 3 H, 19-CH,38-CH), 7.36 - 7.41 (m, 2 H, 37-CH), 7.15 - 7.27 (m, 2 H, 20-CH,21-CH), 4.64 (d, J=8.9 Hz, 1 H, 26-CH), 4.55 - 4.61 (m, 1 H, 33-CH), 4.45 - 4.55 (m, 2 H, 31-CH.35-CH), 4.30 - 4.39 (m, 1 H, 35-CH), 3.86 - 3.94 (m, 1 H, 30-CH), 3.74 - 3.83 (m, 1 H, 30-CH), 2.46 (s, 3 H, 43-CH_3), 2.39 (t, J=7.5 Hz, 2 H, 10-CH_2), 2.17 - 2.32 (m, 3 H, 2-CH_2,32-CH), 2.02 - 2.12 (m, 1 H, 32-CH), 1.64 - 1.75 (m, 2 H, 9-CH_2), 1.54 - 1.64 (m, 2 H, 3-CH_2), 1.49 (s, 9 H, 25-CH_3), 1.28 - 1.40 (m, 10 H, (4-8)-CH_2), 1.03 (s, 9 H, 28-CH_3). ^{13}C NMR (101 MHz, CD_3OD) δ_C ppm 176.2 (C1), 175.1 (C11), 174.6 (C34), 172.5 (C29), 167.8 (C16), 156.4 (C23), 153.0 (C41), 149.1 (C32), 143.9 (C12), 140.4 (C36), 133.2 (C18), 131.8 (C17), 131.6 (C39), 130.5 (C37), 130.3 (C15), 129.7 (C14), 129.1 (C38), 127.5 (C20/21), 127.3 (C22), 126.4 (C20/21), 125.7 (C19), 120.4 (C13), 81.9 (C24), 71.2 (C32), 61.0 (C33), 59.1 (C26), 58.2 (C30), 43.8 (C35), 39.1 (C32), 38.2 (C10), 36.8 (C2), 36.7 (C27), 30.6 (alkyl CH_2), 30.5 (alkyl CH_2), 30.5 (alkyl CH_2), 30.45 (alkyl CH_2), 30.4 (alkyl CH_2), 28.8 (C25), 27.2 (C28), 27.1 (C3), 26.9 (C9), 16.0 (C43). HRMS (ESI) m/z: $[M+H]^+$ calculated for $C_{51}H_{68}N_7O_8S$: 938.4812.

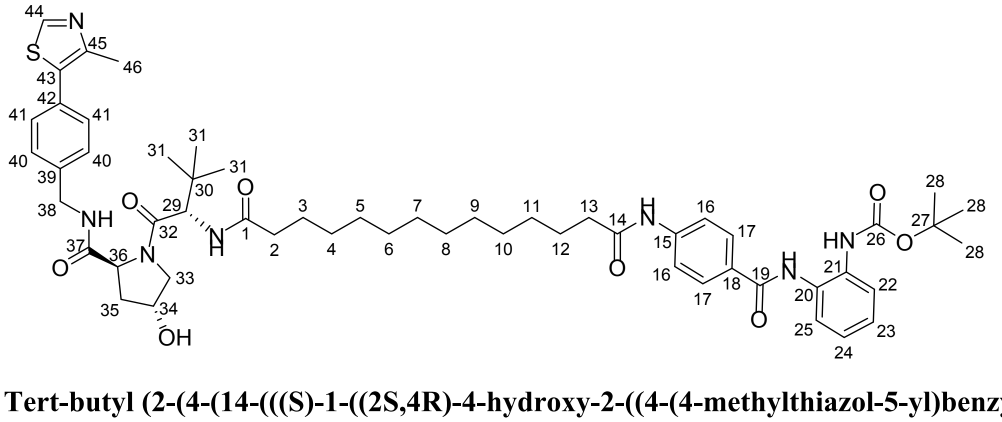

Tert-butyl (2-(4-(14-(((S)-1-((2S,4R)-4-hydroxy-2-((4-(4-methylthiazol-5-yl)benzyl)carbamoyl)pyrrolidin-1-yl)-3,3-dimethyl-1-oxobutan-2-yl)amino)-14-oxotetradecanamido)benzamido)phenyl)carbamate (54e): Following general method G, **54e** was obtained from **53e** (53.5 mg, 0.094 mmol) and **VH_032 amine** (40.0 mg, 0.080 mmol). The crude product was purified by column chromatography (0-5% MeOH in DCM) to afford **54e** (62.5 mg, 0.063 mmol, 79% yield) as a white solid. ^{1}H NMR (400 MHz, CD_3OD) δ_H ppm 8.86 (s, 1 H, 44-CH), 7.93 (d, J=8.8 Hz, 2 H, 17-CH), 7.73 (d, J=8.8 Hz, 2 H, 16-CH), 7.56 - 7.63 (m, 1 H, 25-CH),

7.42 - 7.47 (m, 3 H, 22,41-CH), 7.37 - 7.42 (m, 2 H, 40-CH), 7.16 - 7.25 (m, 2 H, 23,24-CH), 4.63 (s, 1 H, 29-CH), 4.55 - 4.60 (m, 1 H, 36-CH), 4.52 (d, *J*=15.5 Hz, 1 H, 38-CH), 4.46 - 4.50 (m, 1 H, 34-CH), 4.34 (d, *J*=15.5 Hz, 1 H, 38-CH), 3.86 - 3.93 (m, 1 H, 33-CH), 3.75 - 3.82 (m, 1 H, 33-CH), 2.39 (t, *J*=7.5 Hz, 2 H, 13-CH_2), 2.17 - 2.33 (m, 3 H, 2-CH_2,35-CH), 2.03 - 2.12 (m, 1 H, 35-CH), 1.69 (quin, *J*=7.5 Hz, 2 H, 12-CH_2), 1.54 - 1.63 (m, 2 H, 3-CH_2), 1.49 (s, 9 H, 28-CH_3), 1.27 - 1.38 (m, 16 H, (4-11)-CH_2), 1.03 (s, 9 H, 31-CH_3). ^{13}C NMR (101 MHz, CD_3OD) δ_C 176.2 (C1), 175.1 (C14), 174.6 (C37), 172.5 (C32), 167.8 (C19), 156.4 (C26), 152.9 (C44), 149.1 (C45), 143.9 (C15), 140.4 (C39), 133.5 (C43), 133.1 (C21), 131.8 (C20), 131.6 (C42), 130.5 (C40), 130.3 (C18), 129.7 (C17), 129.1 (C41), 127.5 (C23/24), 127.3 (C25), 126.4 (C23/24), 125.7 (C22), 120.4 (C16), 81.8 (C27), 71.2 (C34), 60.9 (C36), 59.1 (C29), 58.1 (C33), 43.8 (C38), 39.0 (C35), 38.2 (C13), 36.8 (C2), 36.7 (C30), 30.8 (alkyl CH_2 x 2), 30.7 (alkyl CH_2), 30.7 (alkyl CH_2), 30.6 (alkyl CH_2 x 2), 30.4 (alkyl CH_2 x 2), 28.8 (C28), 27.2 (C31), 27.1 (C3), 26.9 (C12), 16.0 (C46). HRMS (ESI) m/z: $[M+H]^+$ calculated for $C_{54}H_{74}N_7O_8S$: 980.5320, found 980.5303.

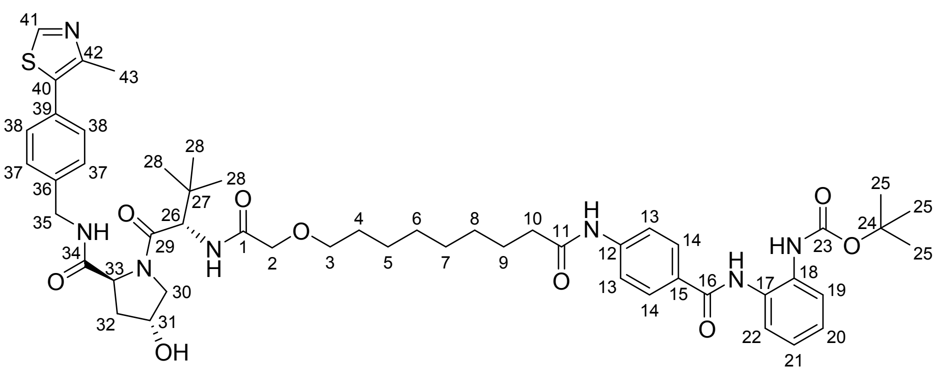

Tert-butyl (2-(4-(9-(2-(((S)-1-((2S,4R)-4-hydroxy-2-((4-(4-methylthiazol-5-yl)benzyl)carbamoyl) pyrrolidin-1-yl)-3,3-dimethyl-1-oxobutan-2-yl)amino)-2-oxoethoxy)nonanamido)benzamido)phenyl)carbamate (54f): Following general method G, **54f** was obtained from **53f** (52.3 mg, 0.099 mmol) and **VH_032 amine** (40.0 mg, 0.080 mmol). The crude product was purified by column chromatography (0-10% MeOH in DCM) to afford **54f** (68.4 mg, 0.071 mmol, 89% yield) as a white solid. ^1H NMR (400 MHz, CD_3OD) δ_H ppm 8.85 (s, 1 H, 41-CH), 7.92 (d, *J*=8.7 Hz, 2 H, 14-CH), 7.73 (d, *J*=8.7 Hz, 2 H, 13-CH), 7.59 (dd, *J*=7.0, 2.3 Hz, 1 H, 22-CH), 7.54 (d, *J*=9.5 Hz, 1 H, 1-NH), 7.42 - 7.46 (m, 3 H, 19,38-CH), 7.38 - 7.41 (d, *J*=8.3 Hz, 2 H, 37-CH), 7.16 - 7.27 (m, 2 H, 20,21-CH), 4.69 (d, *J*=9.5 Hz, 1 H, 26-CH), 4.56 - 4.63 (m, 1 H, 33-CH), 4.47 - 4.55 (m, 2 H, 31,35-CH), 4.35 (d, *J*=15.6 Hz, 1 H, 35-CH), 3.96 (d, *J*=15.4 Hz, 1 H, 2-CH), 3.94 (d, *J*=15.4 Hz, 1 H, 2-CH), 3.84 - 3.90 (m, 1 H, 30-CH), 3.76 - 3.83 (m, 1 H, 30-CH), 3.54 (t, *J*=6.4 Hz, 2 H, 3-CH_2), 2.46 (s, 3 H, 43-CH_3), 2.38 (t, *J*=7.5 Hz, 2 H, 10-CH_2), 2.19 - 2.27 (m, 1 H, 32-CH), 2.04 - 2.13 (m, 1 H, 32-CH), 1.59 - 1.71 (m, 4 H, 4,9-CH_2), 1.49 (s, 9 H, 25-CH_3), 1.35 - 1.43 (m, 8 H, (5-8)-CH_2), 1.03 (s, 9 H, 28-CH_3). ^{13}C NMR (101 MHz, CD_3OD) δ_C ppm 175.0 (C11), 174.5 (C34), 172.2 (C1), 171.8 (C29), 167.9 (C16), 156.4 (C23), 153.0 (C41), 149.2 (C42), 143.9 (C12), 140.3 (C36), 133.6 (C40), 133.2 (C18), 131.8 (C39), 131.6 (C17), 130.5 (C37), 130.3 (C15), 129.7 (C14), 129.1 (C38), 127.5 (C20/21), 127.3 (C22), 126.4 (C20/21), 125.7 (C19), 120.4 (C13), 81.9 (C24), 73.1 (C3), 71.2 (C31), 70.9 (C2), 61.0 (C33), 58.3 (C30), 58.1 (C26), 43.9 (C35), 39.1 (C32), 38.2 (C10), 37.4 (C27), 30.8 (C4), 30.6 (C6/7/8), 30.5 (C6/7/8), 30.4

(C6/7/8), 28.8 (C25), 27.3 (C5), 27.1 (C28), 26.9 (C9),16.0 (C43). HRMS (ESI) m/z: $[M+H]^+$ calculated for $C_{51}H_{68}N_7O_9S$: 954.4799, found 954.4798.

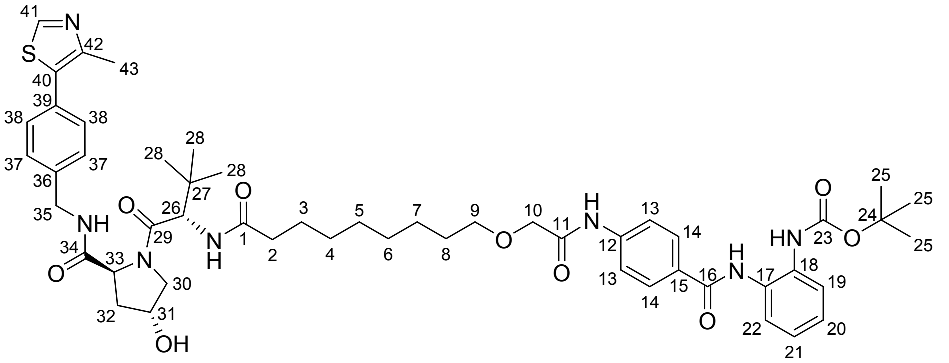

Tert-butyl (2-(4-(2-((9-(((S)-1-((2S,4R)-4-hydroxy-2-((4-(4-methylthiazol-5-yl)benzyl)carbamoyl) pyrrolidin-1-yl)-3,3-dimethyl-1-oxobutan-2-yl)amino)-9-oxononyl)oxy)acetamido)benzamido)phenyl)carbamate (54g): Following general method G, **54g** was obtained from **53g** (52.8 mg, 0.097 mmol) and **VH_032 amine** (40.0 mg, 0.080 mmol). The crude product was purified by column chromatography (0-10% MeOH in DCM) to afford **54g** (72.9 mg, 0.076 mmol, 95% yield) as a pale yellow/white solid. ^{1}H NMR (400 MHz, CD_3OD) δ_H ppm 8.86 (s, 1 H, 41-CH), 7.95 (d, *J*=8.8 Hz, 2 H, 14-CH), 7.75 - 7.82 (m, 3 H, 13-CH,1-NH), 7.58 (dd, J=7.0, 2.4 Hz, 1 H, 22-CH), 7.42 - 7.47 (m, 3 H, 19,38-CH), 7.40 (d, *J*=8.4 Hz, 2 H, 37-CH), 7.17 - 7.25 (m, 2 H, 20,21-CH), 4.61 - 4.66 (m, 1 H, 26-CH), 4.55 - 4.60 (m, 1 H, 33-CH), 4.52 (d, *J*=15.5 Hz, 1 H, 35-CH), 4.46 - 4.49 (m, 1 H, 31-CH), 4.34 (d, *J*=15.5 Hz, 1 H, 35-CH), 4.09 (s, 2 H, 10-CH_2), 3.86 - 3.93 (m, 1 H, 30-CH), 3.75 - 3.82 (m, 1 H, 30-CH), 3.58 (t, *J*=6.6 Hz, 2 H, 9-CH_2), 2.46 (s, 3 H, 43-CH_3), 2.18 - 2.33 (m, 3 H, 2-CH_2,32-CH), 2.03 - 2.12 (m, 1 H, 32-CH), 1.64 - 1.71 (m, 2 H, 8-CH_2), 1.55 - 1.63 (m, 2 H, 3-CH_2), 1.49 (s, 9 H, 25-CH_3), 1.32 - 1.41 (m, 8 H, (4-7)-CH_2), 1.03 (s, 9 H, 28-CH_3). ^{13}C NMR (101 MHz, CD_3OD) δ_C ppm 176.1 (C1), 174.6 (C34), 172.5 (C29), 171.4 (C11), 167.7 (C16), 156.4 (C23), 153.0 (C41), 149.1 (C42), 142.8 (C12), 140.4 (C36), 133.5 (C40), 133.2 (C18), 131.8 (C39), 131.6 (C17), 130.9 (C15), 130.5 (C37), 129.8 (C14), 129.1 (C38), 127.5 (C20/21), 127.3 (C22), 126.4 (C20/21), 125.7 (C19), 120.9 (C13), 81.9 (C24), 73.2 (C9), 71.5 (C10), 71.2 (C31), 60.9 (C33), 59.1 (C26), 58.2 (C30), 43.8 (C35), 39.0 (C32), 36.8 (C27), 36.7 (C2), 30.6 (C8), 30.5 (C4/5/6), 30.45 (C4/5/6), 30.4 (C4/5/6), 28.8 (C25), 27.2 (C28), 27.15 (C7), 27.1 (C3), 16.0 (C43). HRMS (ESI) m/z: $[M+H]^+$ calculated for $C_{51}H_{68}N_7O_9S$: 954.4799, found 954.4792.

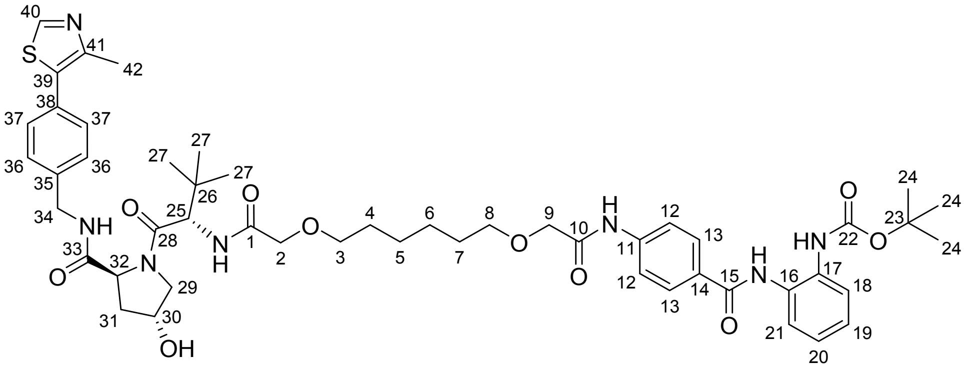

Tert-butyl (2-(4-(2-((6-(2-(((S)-1-((2S,4R)-4-hydroxy-2-((4-(4-methylthiazol-5-yl)benzyl)carbamoyl)pyrrolidin-1-yl)-3,3-dimethyl-1-oxobutan-2-yl)amino)-2-oxoethoxy)hexyl)oxy)acetamido)benz

amido)phenyl)carbamate (54h): Following general method G, **54h** was obtained from **53h** (67.2 mg, 0.124 mmol) and **VH_032 amine** (50.0 mg, 0.099 mmol). The crude product was purified by column chromatography (0-10% MeOH in DCM) to afford **54h** (92.4 mg, 0.093 mmol, 94% yield) as a white solid. ^{1}H NMR (400 MHz CD$_3$OD) δ_H ppm 8.85 (s, 1 H, 40-CH), 7.94 (d, *J*=8.7 Hz, 2 H, 13-CH), 7.77 (d, *J*=8.7 Hz, 2 H, 12-CH), 7.57 - 7.61 (m, 1 H, 21-CH), 7.42 - 7.46 (m, 3 H, 18,37-CH), 7.40 (d, *J*=8.4 Hz, 2 H, 36-CH), 7.18 - 7.26 (m, 2 H, 19,20-CH), 4.69 (s, 1 H, 25-CH), 4.55 - 4.63 (m, 1 H, 32-CH), 4.50 - 4.55 (m, 1 H, 34-CH), 4.46 - 4.50 (m, 1 H, 30-CH), 4.31 - 4.38 (m, 1 H, 34-CH), 4.07 (s, 2 H, 9-CH$_2$), 3.97 (d, *J*=15.4 Hz, 1 H, 2-CH), 3.93 (d, *J*=15.4 Hz, 1 H, 2-CH), 3.84 - 3.90 (m, 1 H, 29-CH), 3.76 - 3.83 (m, 1 H, 29-CH), 3.59 (t, *J*=6.6 Hz, 2 H, 8-CH$_2$), 3.56 (t, *J*=6.3 Hz, 2 H, 3-CH$_2$), 2.46 (s, 3 H, 42-CH$_3$), 2.19 - 2.28 (m, 1 H, 31-CH), 2.04 - 2.13 (m, 1 H, 31-CH), 1.63 - 1.74 (m, 4 H, 4,7-CH$_2$), 1.49 (s, 13 H, 5,6-CH$_2$,24-CH$_3$), 1.03 (s, 9 H, 27-CH$_3$). ^{13}C NMR (101 MHz, CD$_3$OD) δ_C ppm 174.5 (C33), 172.1 (C1), 171.8 (C28), 171.4 (C10), 167.8 (C15), 156.4 (C22), 153.0 (C40), 149.2 (C41), 142.8 (C11), 140.4 (C35), 133.5 (C39), 133.2 (C17), 131.8 (C38), 131.7 (C16), 130.9 (C14), 130.5 (C36), 129.8 (C13), 129.1 (C37), 127.5 (C19/20), 127.3 (C21), 126.4 (C19/20), 125.7 (C18), 121.0 (C12), 81.9 (C23), 73.1 (C8), 73.0 (C3), 71.5 (C9), 71.2 (C30), 70.9 (C2), 61.0 (C32), 58.3 (C29), 58.1 (C25), 43.9 (C34), 39.1 (C31), 37.4 (C26), 30.7 (C4/7), 30.5 (C4/7), 28.8 (C24), 27.25 (C5/6), 27.2 (C5/6), 27.1 (C27), 16.0 (C42). HRMS (ESI) m/z: $[M+H]^+$ calculated for $C_{50}H_{66}N_7O_{10}S$: 956.4592, found 956.4590.

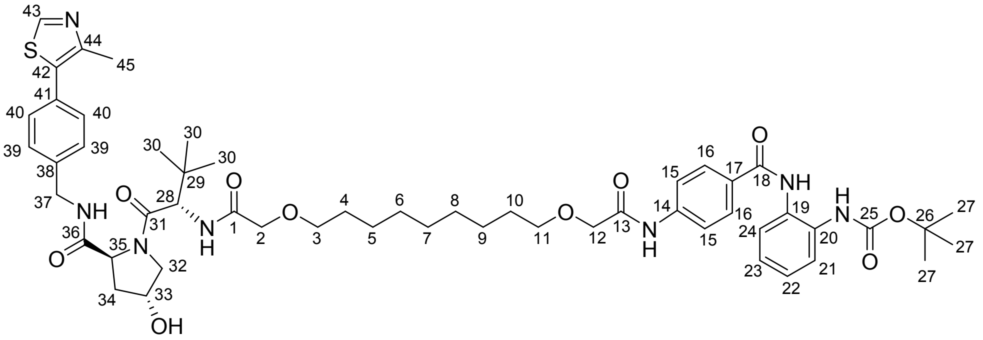

Tert-butyl (2-(4-(2-((9-(2-(((S)-1-((2S,4R)-4-hydroxy-2-((4-(4-methylthiazol-5-yl)benzyl)carbamoyl)pyrrolidin-1-yl)-3,3-dimethyl-1-oxobutan-2-yl)amino)-2-oxoethoxy)nonyl)oxy)acetamido)benzamido)phenyl)carbamate (54i): Following general method G, **54i** was obtained from **53i** (56.2 mg, 0.096 mmol) and **VH_032 amine** (40.0 mg, 0.080 mmol). The crude product was purified by column chromatography (0-8% MeOH in DCM) to afford **54i** (68.4 mg, 0.068 mmol, 85% yield) as a white solid. ^{1}H NMR (400 MHz, CD$_3$OD) δ_H ppm 8.86 (s, 1 H, 43-CH), 7.95 (d, *J*=8.7 Hz, 2 H, 16-CH), 7.77 (d, *J*=8.7 Hz, 2 H, 15-CH), 7.59 (dd, *J*=7.5, 1.8 Hz, 1 H, 24-CH), 7.53 (br d, *J*=9.6 Hz, 1 H, 1-NH), 7.42 - 7.47 (m, 3 H, 21,40-CH), 7.39 (d, *J*=8.3 Hz, 2 H, 39-CH), 7.16 - 7.26 (m, 2 H, 22,23-CH), 4.69 (d, *J*=9.6 Hz, 1 H, 28-CH), 4.56 - 4.62 (m, 1 H, 35-CH), 4.45 - 4.55 (m, 2 H, 37,33-CH), 4.34 (d, *J*=15.6 Hz, 1 H, 37-CH), 4.08 (s, 2 H, 12-CH$_2$), 3.95 (d, *J*=15.5 Hz, 1 H, 2-CH), 3.94 (d, *J*=15.5 Hz, 1 H, 2-CH), 3.84 - 3.90 (m, 1 H, 32-CH), 3.76 - 3.82 (m, 1 H, 32-CH), 3.50 - 3.60 (m, 4 H, 3,11-CH$_2$), 2.46 (s, 3 H, 45-CH$_3$), 2.17 - 2.27 (m, 1 H, 34-CH), 2.03 - 2.12 (m, 1 H, 34-CH), 1.59 - 1.69 (m, 4 H, 4,10-CH$_2$), 1.49 (s, 9 H, 27-CH$_3$), 1.31 - 1.43 (m, 10 H, (5-9)-CH$_2$), 1.03 (s, 9 H, 30-CH$_3$). ^{13}C NMR (101 MHz, CD$_3$OD) δ_C ppm 174.4 (C36), 172.2 (C1), 171.8 (C31), 171.4 (C13), 167.7 (C18), 156.4 (C25), 153.0 (C43), 149.2 (C44), 142.8 (C14), 140.4 (C38), 133.6 (C42), 133.2 (C20),

131.8 (C41), 131.6 (C19), 130.9 (C17), 130.5 (C39), 129.8 (C16), 129.1 (C40), 127.5 (C22/23), 127.3 (C24), 126.4 (C22/23), 125.7 (C21), 120.9 (C15), 81.9 (C26), 73.2 (C11), 73.1 (C3), 71.5 (C12), 71.2 (C33), 70.9 (C2), 61.0 (C35), 58.3 (C32), 58.1 (C28), 43.9 (C37), 39.1 (C34), 37.4 (C29), 30.8 (C4/10), 30.8 (C4/10), 30.7 (C6/7/8), 30.6 (C6/7/8), 30.6 (C6/7/8), 28.8 (C27), 27.4 (C5/9), 27.3 (C5/9), 27.1 (C30), 16.0 (C45). HRMS (ESI) m/z: $[M+H]^+$ calculated for $C_{53}H_{72}N_7O_{10}S$: 998.5061, found 998.5048.

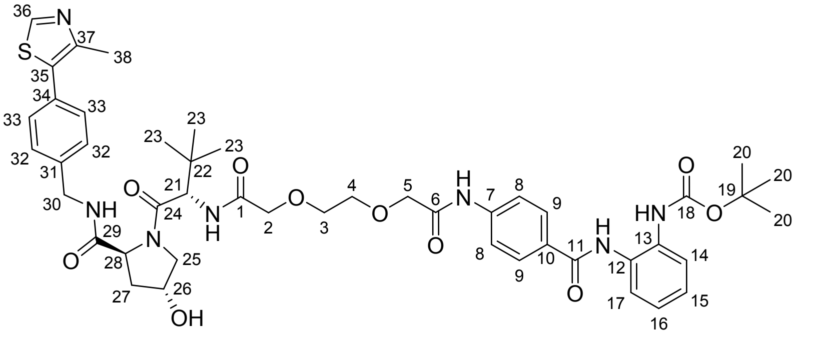

Tert-butyl (2-(4-(2-(2-(2-(((S)-1-((2S,4R)-4-hydroxy-2-((4-(4-methylthiazol-5-yl)benzyl)carbamoyl) pyrrolidin-1-yl)-3,3-dimethyl-1-oxobutan-2-yl)amino)-2-oxoethoxy)ethoxy)acetamido)benzamido)phenyl)carbamate (54j): Following general method G, **54j** was obtained from **53j** (58.1 mg, 0.121 mmol) and **VH_032 amine** (50.0 mg, 0.099 mmol). The crude product was purified by column chromatography (0-10% MeOH in DCM) to afford **54j** (85.5 mg, 0.091 mmol, 92% yield) as a white solid. ^{1}H NMR (400 MHz, CD_3OD) δ_H ppm 8.84 (s, 1 H, 36-CH), 7.93 (d, *J*=8.9 Hz, 2 H, 9-CH), 7.79 (d, *J*=8.9 Hz, 2 H, 8-CH), 7.58 (dd, *J*=7.5, 1.7 Hz, 1 H, 17-CH), 7.43 - 7.46 (m, 1 H, 14-CH), 7.40 (d, *J*=8.5 Hz, 2 H, 33-CH), 7.36 (d, *J*=8.5 Hz, 2 H, 32-CH), 7.18 - 7.26 (m, 2 H, 15,16-CH), 4.73 (s, 1 H, 21-CH), 4.56 - 4.62 (m, 1 H, 28-CH), 4.50 - 4.53 (m, 1 H, 30-CH), 4.44 - 4.49 (m, 1 H, 26-CH), 4.30 - 4.35 (m, 1 H, 30-CH), 4.22 (s, 2 H, 5-CH_2), 4.14 (d, *J*=15.7 Hz, 1 H, 2-CH), 4.09 (d, *J*=15.7 Hz, 1 H, 2-CH), 3.86 - 3.91 (m, 1 H, 25-CH), 3.77 - 3.85 (m, 5 H, 3-CH_2,4-CH_2,25-CH), 2.44 (s, 3 H, 38-CH_3), 2.19 - 2.27 (m, 1 H, 27-CH), 2.05 - 2.13 (m, 1 H, 27-CH), 1.48 (s, 9 H, 20-CH_3), 1.04 (s, 9 H, 23-CH_3). ^{13}C NMR (101 MHz, CD_3OD) δ_C ppm 174.4 (C29), 172.2 (C1), 172.0 (C24), 171.2 (C6), 167.8 (C11), 156.4 (C18), 152.9 (C36), 149.1 (C37), 142.8 (C7), 140.3 (C31), 133.5 (C35), 133.2 (C13), 131.8 (C34), 131.6 (C12), 130.9 (C10), 130.5 (C32), 129.8 (C9), 129.0 (C33), 127.5 (C15/16), 127.3 (C16), 126.3 (C15/16), 125.7 (C14), 121.0 (C8), 81.9 (C19), 72.3 (C3), 72.1 (C4), 72.0 (C5), 71.2 (C26), 71.1 (C2), 61.0 (C28), 58.4 (C21), 58.3 (C25), 43.9 (C30), 39.1 (C27), 37.3 (C22), 28.8 (C20), 27.1 (C23), 16.0 (C38). HRMS (ESI) m/z: $[M+H]^+$ calculated for $C_{46}H_{58}N_7O_{10}S$: 900.3966, found 900.3956.

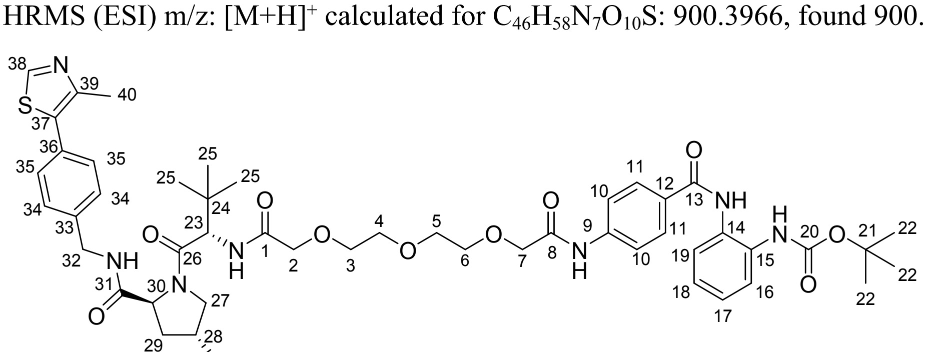

Tert-butyl (2-(4-((S)-13-((2S,4R)-4-hydroxy-2-((4-(4-methylthiazol-5-yl)benzyl)carbamoyl)pyrrolidine-1-carbonyl)-14,14-dimethyl-11-oxo-3,6,9-trioxa-12-azapentadecanamido)benzamido)phenyl)

carbamate (54k): Following general method G, **54k** was obtained from **53k** (63.4 mg, 0.119 mmol) and **VH_032 amine** (50.0 mg, 0.099 mmol). The crude product was purified by column chromatography (0-10% MeOH in DCM) to afford **54k** (72.2 mg, 0.070 mmol, 71% yield) as a white solid. ^{1}H NMR (400 MHz, CD_3OD) δ_H ppm 8.84 (s, 1 H, 38-CH), 8.60 (t, *J*=6.1 Hz, 1 H, 31-NH), 7.94 (d, *J*=8.9 Hz, 2 H, 11-CH), 7.76 (d, *J*=8.9 Hz, 2 H, 10-CH), 7.63 (d, *J*=9.6 Hz, 1 H, 1-NH), 7.56 - 7.61 (m, 1 H, 19-CH), 7.44 - 7.46 (m, 1 H, 16-CH), 7.41 - 7.43 (m, 2 H, 35-CH), 7.36 - 7.40 (m, 2 H, 34-CH), 7.16 - 7.25 (m, 2 H, 17,18-CH), 4.67 - 4.72 (m, 1 H, 23-CH), 4.56 - 4.60 (m, 1 H, 30-CH), 4.51 - 4.56 (m, 1 H, 32-CH), 4.46 - 4.50 (m, 1 H, 28-CH), 4.28 - 4.36 (m, 1 H, 32-CH), 4.13 - 4.19 (m, 1 H, 7-CH), 4.08 - 4.13 (m, 1 H, 7-CH), 4.01 - 4.07 (m, 1 H, 2-CH), 3.92 - 3.98 (m, 1 H, 2-CH), 3.82 - 3.88 (m, 1 H, 27-CH), 3.78 - 3.80 (m, 1 H, 27-CH), 3.71 - 3.78 (m, 8 H, (3-6)-CH_2), 2.45 (s, 3 H, 40-CH_3), 2.16 - 2.25 (m, 1 H, 29-CH), 2.04 - 2.13 (m, 1 H, 29-CH), 1.48 (s, 9 H, 22-CH_3), 1.02 (s, 9 H, 25-CH_3). ^{13}C NMR (101 MHz, CD_3OD) δ_C ppm 174.5 (C31), 172.1 (C1), 171.7 (C26), 171.5 (C8), 167.8 (C13), 156.4 (C20), 152.9 (C38), 149.2 (C39), 142.7 (C9), 140.3 (C33), 133.5 (C37), 133.2 (C15), 131.7 (C36), 131.6 (C14), 131.0 (C12), 130.5 (C34), 129.8 (C11), 129.1 (C35), 127.5 (C17/18), 127.3 (C19), 126.3 (C17/18), 125.6 (C16), 121.1 (C10), 81.8 (C21), 72.3 (2 x alkoxy CH_2), 71.7 (C7), 71.6 (alkoxy CH_2), 71.5 (alkoxy CH_2), 71.2 (C28), 71.1 (C2), 60.9 (C30), 58.3 (C23), 58.2 (C27), 43.9 (C32), 39.1 (C29), 37.3 (C24), 28.8 (C22), 27.1 (C25), 16.0 (C40). HRMS (ESI) m/z: $[M+H]^+$ calculated for $C_{48}H_{62}N_7O_{11}S$: 944.4228, found 944.4230.

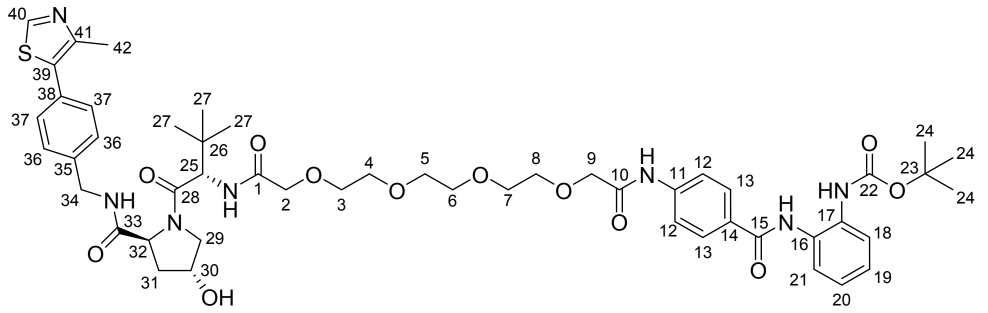

Tert-butyl (2-(4-((S)-16-((2S,4S)-4-hydroxy-2-((4-(4-methylthiazol-5-yl)benzyl)carbamoyl)pyrrolidine-1-carbonyl)-17,17-dimethyl-14-oxo-3,6,9,12-tetraoxa-15-azaoctadecanamido)benzamido)phenyl) carbamate (54l): Following general method G, **54l** was obtained from **53l** (52.1 mg, 0.09 mmol) and **VH_032 amine** (40.7 mg, 0.08 mmol). The crude product was purified by column chromatography (alumina (basic), 0-10% MeOH in DCM) to afford **54l** (15.7 mg, 0.02 mmol, 20 %) as a pale yellow tar. ^{1}H NMR (400 MHz, CD_3OD) δ_H ppm 8.86 (s, 1 H, 40-CH), 7.95 (d, *J*=8.8 Hz, 2 H, 13-CH), 7.80 (d, *J*=8.8 Hz, 2 H, 12-CH), 7.59 (dd, *J*=7.7, 1.3 Hz, 1 H, 21-CH), 7.42 - 7.46 (m, 3 H, 18-CH, 37-CH), 7.37 - 7.42 (m, 2 H, 36-CH), 7.18 - 7.26 (m, 2 H, 19-CH,20-CH), 4.68 (s, 1 H, 25-CH), 4.55 - 4.59 (m, 1 H, 32-CH), 4.47 - 4.55 (m, 2 H, 30-CH,34-CH), 4.33 (d, *J*=15.5 Hz, 1 H, 34-CH), 4.12 - 4.18 (m, 2 H, 9-CH_2), 4.01 - 4.07 (m, 1 H, 2-CH_2), 3.96 - 4.01 (m, 1 H, 2-CH_2), 3.83 - 3.88 (m, 1 H, 29-CH), 3.76 - 3.81 (m, 1 H, 29-CH), 3.65 - 3.76 (m, 12 H, (3-8)-CH_2), 2.46 (s, 3 H, 42-CH_3), 2.18 - 2.26 (m, 1 H, 31-CH), 2.03 - 2.11 (m, 1 H, 31-CH), 1.49 (s, 9 H, 24-CH_3), 1.03 (s, 9 H, 27-CH_3). ^{13}C NMR (101 MHz, CD_3OD) δ_C ppm 174.5 (C33), 172.2 (C1), 171.8 (C28), 171.5 (C10), 167.8 (C15), 156.4 (C22), 153.0 (C40), 149.2 (C41), 142.8 (C11), 140.4 (C35), 133.5 (C39), 133.3 (C17),

131.8 (C38), 131.6 (C16), 131.0 (C14), 130.5 (C36), 129.8 (C13), 129.1 (C37), 127.5 (C19/20), 127.3 (C21), 126.4 (C19/20), 125.7 (C18), 121.1 (C12), 81.9 (C23), 72.4 (alkoxy CH_2), 72.3 (alkoxy CH_2), 71.8 (alkoxy CH_2), 71.7 (alkoxy CH_2), 71.6 (2 x alkoxy CH_2), 71.5 (alkoxy CH_2), 71.3 (C30), 71.2 (C2), 61.0 (C32), 58.5 (C25), 58.2 (C29), 43.9 (C34), 39.1 (C31), 37.3 (C26), 28.8 (C24), 27.1 (C27), 16.0 (C42). HRMS (ESI) m/z: $[M+H]^+$ calculated for $C_{50}H_{66}N_7O_{12}S$: 988.4490 , found 988.4480.

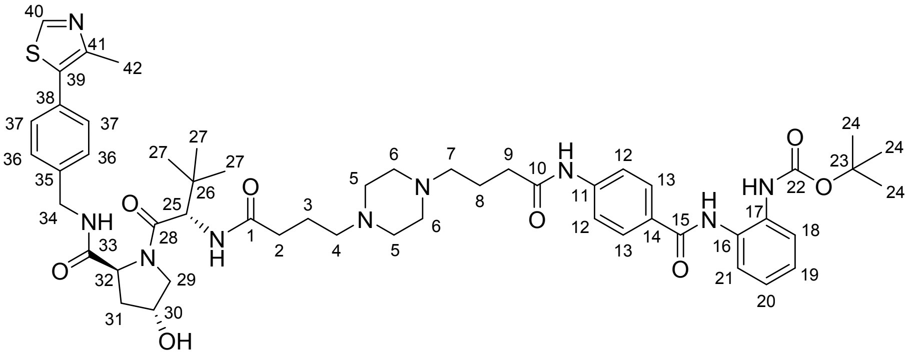

Tert-butyl (2-(4-(4-(4-(4-(((S)-1-((2S,4R)-4-hydroxy-2-((4-(4-methylthiazol-5-yl)benzyl)carbamoyl)pyrrolidin-1-yl)-3,3-dimethyl-1-oxobutan-2-yl)amino)-4-oxobutyl)piperazin-1-yl)butanamido)benzamido)phenyl)carbamate (54m): Following general method G, **54m** was obtained from **53m** (55.0 mg, 0.093 mmol) and VH_032 amine (35.0 mg, 0.075 mmol). The crude product was purified by column chromatography (alumina (basic), 0-10% MeOH in DCM) to afford **54m** (20.2 mg, 0.021 mmol, 27% yield) as a white solid. ^{1}H NMR (400 MHz, CD_3OD) δ_H ppm 8.87 (s, 1 H, 40-CH), 7.94 (d, *J*=8.8 Hz, 2 H, 13-CH), 7.73 (d, *J*=8.8 Hz, 2 H, 12-CH), 7.59 - 7.62 (m, 1 H, 21-CH), 7.44 - 7.48 (m, 2 H, 37-CH), 7.38 - 7.44 (m, 3 H, 18-CH,36-CH), 7.19 - 7.24 (m, 2 H, 19-CH,20-CH), 4.62 (s, 1 H, 25-CH), 4.51 - 4.59 (m, 2 H, 32-CH,34-CH), 4.46 - 4.51 (m, 1 H, 30-CH), 4.34 (d, *J*=15.4 Hz, 1 H, 34-CH), 3.86 - 3.93 (m, 1 H, 29-CH), 3.75 - 3.82 (m, 1 H, 29-CH), 2.39 - 2.59 (m, 15 H, (5,6,7,9)-CH_2,42-CH_3), 2.32 - 2.37 (m, 2 H, 4-CH_2), 2.26 - 2.31 (m, 2 H, 2-CH_2), 2.17 - 2.24 (m, 1 H, 31-CH), 2.04 - 2.11 (m, 1 H, 31-CH), 1.86 - 1.93 (m, 2 H, 8-CH_2), 1.74 - 1.83 (m, 2 H, 3-CH_2), 1.49 (s, 9 H, 24-CH_3), 1.03 (s, 9 H, 27-CH_3). ^{13}C NMR (101 MHz, CD_3OD) δ_C ppm 175.5 (C1), 174.6 (C10), 174.5 (C33), 172.4 (C28), 167.9 (C15), 156.4 (C22), 153.0 (C40), 149.2 (C41), 144.0 (C11), 140.4 (C35), 133.6 (C39), 133.2 (C17), 131.9 (C16), 131.6 (C38), 130.5 (C36), 130.3 (C14), 129.7 (C13), 129.1 (C37), 127.5 (C19/20), 127.3 (C21), 126.4 (C19/20), 125.7 (C18), 120.4 (C12), 81.9 (C23), 71.2 (C30), 61.0 (C32), 59.2 (C25), 59.0 (C7), 58.7 (C4), 58.2 (C29), 53.9 (C5,6), 43.8 (C34), 39.1 (C31), 36.7 (C26), 36.1 (C9), 34.5 (C2), 28.8 (C24), 27.2 (C27), 23.8 (C3), 23.6 (C8), 16.0 (C42). HRMS (ESI) m/z: $[M+H]^+$ calculated for $C_{52}H_{70}N_9O_8S$: 980.5068, found 980.5056.

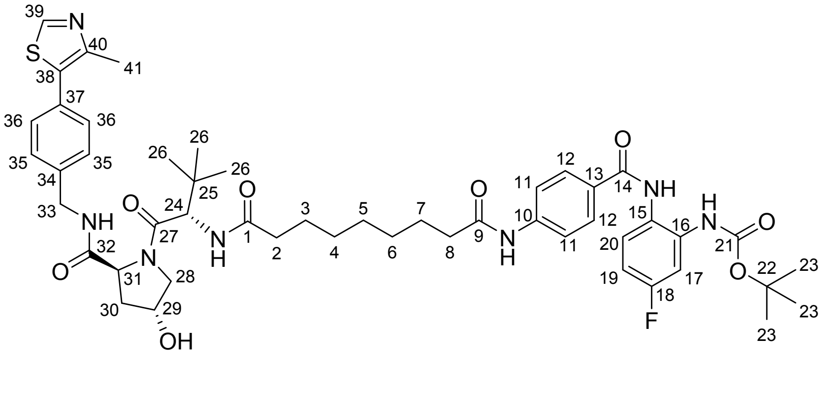

Tert-butyl (5-fluoro-2-(4-(9-(((S)-1-((2S,4R)-4-hydroxy-2-((4-(4-methylthiazol-5-yl)benzyl)carbamoyl) pyrrolidin-1-yl)-3,3-dimethyl-1-oxobutan-2-yl)amino)-9-oxononanamido)benzamido)phenyl) carbamate (54n): Following general method G, **54n** was obtained from **53n** (50.5 mg, 0.098 mmol) and **VH_032 amine** (40.0 mg, 0.080 mmol). The crude product was purified by column chromatography (0-10% MeOH in DCM) to afford **54n** (61.3 mg, 0.065 mmol, 82% yield) as a pale yellow/white solid. ^{1}H NMR (400 MHz, CD_3OD) δ_H ppm 8.85 (s, 1 H, 39-CH), 7.92 (d, *J*=8.7 Hz, 2 H, 12-CH), 7.81 (d, *J*=9.0 Hz, 1 H, 1-NH), 7.72 (d, *J*=8.7 Hz, 2 H, 11-CH), 7.43 - 7.48 (m, 3 H, 36-CH, 20-CH), 7.37 - 7.43 (m, 3 H, 35-CH, 17-CH), 6.90 (ddd, *J*=8.9, 7.8, 2.9 Hz, 1 H, 19-CH), 4.62 - 4.66 (m, 1 H, 24-CH), 4.55 - 4.61 (m, 1 H, 31-CH), 4.52 (d, *J*=15.5 Hz, 1 H, 33-CH), 4.47 - 4.50 (m, 1 H, 29-CH), 4.35 (d, *J*=15.5 Hz, 1 H, 33-CH), 3.87 - 3.94 (m, 1 H, 28-CH), 3.76 - 3.83 (m, 1 H, 28-CH), 2.46 (s, 3 H, 41-CH_3), 2.39 (t, *J*=7.5 Hz, 2 H, 8-CH_2), 2.17 - 2.33 (m, 3 H, 2-CH_2,30-CH), 2.03 - 2.12 (m, 1 H, 30-CH), 1.70 (quin, *J*=7.5 Hz, 2 H, 7-CH_2), 1.61 (quin, *J*=7.0 Hz, 2 H, 3-CH_2), 1.48 (s, 9 H, 23-CH_3), 1.34 - 1.39 (m, 6 H, (4-6)-CH_2), 1.03 (s, 9 H, 26-CH_3). ^{13}C NMR (101 MHz, CD_3OD) δ_C ppm 176.2 (C1), 175.1 (C9), 174.6 (C32), 172.5 (C27), 168.3 (C14), 162.2 (d, J_{CF}=243.0 Hz, C18), 155.6 (C21), 153.0 (C39), 149.1 (C40), 143.9 (C10), 140.4 (C34), 135.8 (d, J_{CF}=11.3 Hz, C16), 133.5 (C38), 131.6 (C37), 130.5 (C35), 130.0 (C13), 129.8 (C12), 129.3 (d, J_{CF}=9.5 Hz, C20), 129.1 (C36), 126.6 (d, J_{CF}=2.9 Hz, C15), 120.4 (C11), 112.1 (d, J_{CF}=22.9 Hz, C19), 111.0 (d, J_{CF}=25.9 Hz, C17), 82.1 (C22), 71.2 (C29), 61.0 (C31), 59.1 (C24), 58.2 (C28), 43.8 (C33), 39.1 (C30), 38.2 (C8), 36.7 (C2), 36.7 (C25), 30.3 (C4/5/6), 30.25 (C4/5/6), 30.2 (C4/5/6), 28.7 (C23), 27.2 (C26), 27.1 (C3), 26.8 (C7), 16.0 (C41). ^{19}F NMR (376 MHz, CD_3OD) δ_F ppm -116.7. HRMS (ESI) m/z: $[M+H]^+$ calculated for $C_{49}H_{63}FN_7O_8S$: 928.4443, found 928.4433.

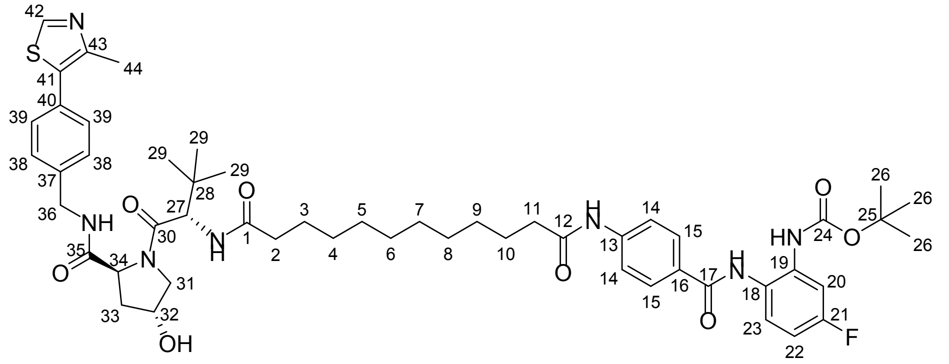

Tert-butyl (5-fluoro-2-(4-(12-(((S)-1-((2S,4R)-4-hydroxy-2-((4-(4-methylthiazol-5-yl)benzyl)carbamoyl) pyrrolidin-1-yl)-3,3-dimethyl-1-oxobutan-2-yl)amino)-12-oxododecanamido)benzamido)phenyl) carbamate (54o): Following general method G, **54o** was obtained from **53o** (53.2 mg, 0.095 mmol) and **VH_032 amine** (40.0 mg, 0.080 mmol). The crude product was purified by column chromatography (0-10% MeOH in DCM) to afford **54o** (58.7 mg, 0.060 mmol, 75% yield) as a white solid. ^{1}H NMR (400 MHz, CD_3OD) δ_H ppm 8.86 (s, 1 H, 42-CH), 8.64 (t, *J*=6.0 Hz, 1 H, 35-NH), 7.93 (d, *J*=8.7 Hz, 2 H, 15-CH), 7.79 (d, *J*=9.0 Hz, 1-NH), 7.73 (d, *J*=8.7 Hz, 2 H, 14-CH), 7.43 - 7.49 (m, 3 H, 39-CH, 23-CH), 7.37 - 7.43 (m, 3 H, 38-CH, 20-CH), 6.90 (ddd, J_{HH}=8.9, 2.9, J_{HF}=7.8 Hz, 1 H, 22-CH), 4.63 (d, *J*=9.0 Hz, 1 H, 27-CH), 4.56 - 4.61 (m, 1 H, 34-CH), 4.51 - 4.56 (m, 1 H, 36-CH), 4.46 - 4.51 (m, 1 H, 32-CH), 4.31 - 4.39 (m, 1 H, 36-CH), 3.86 - 3.93 (m, 1 H, 31-CH), 3.76 - 3.83 (m, 1 H, 31-CH), 2.46 (s, 3 H, 44-CH_3), 2.39 (t, *J*=7.5 Hz, 2 H, 11-

CH_2), 2.17 - 2.32 (m, 3 H, 2-CH_2,33-CH), 2.04 - 2.12 (m, 1 H, 33-CH), 1.69 (quin, *J*=7.5 Hz, 2 H, 10-CH_2), 1.55 - 1.64 (m, 2 H, 3-CH_2), 1.49 (s, 9 H, 26-CH_3), 1.29 - 1.38 (m, 12 H, (4-9)-CH_2), 1.03 (s, 9 H, 29-CH_3). ^{13}C NMR (101 MHz, CD_3OD) δ_C ppm 176.2 (C1), 175.1 (C12), 174.6 (C35), 172.5 (C30), 168.4 (C17), 162.2 (d, J_{CF}=243.0 Hz, C21), 155.6 (C24), 153.0 (C42), 149.1 (C43), 143.9 (C13), 140.4 (C37), 135.8 (d, J_{CF}=11.1 Hz, C19), 133.6 (C41), 131.6 (C40), 130.5 (C38), 130.1 (C16), 129.8 (C15), 129.3 (d, J_{CF}=9.5 Hz, C23), 129.1 (C39), 126.6 (d, J_{CF}=2.5 Hz, C18), 120.4 (C14), 112.1 (d, J_{CF}=22.7 Hz, C22), 111.0 (d, J_{CF}=25.4 Hz, C20), 82.1 (C25), 71.2 (C32), 60.9 (C34), 59.1 (C27), 58.1 (C31), 43.8 (C36), 39.0 (C33), 38.2 (C11), 36.8 (C2), 36.7 (C28), 30.7 (alkyl CH_2), 30.6 (alkyl CH_2), 30.55 (alkyl CH_2), 30.5 (alkyl CH_2), 30.4 (2x alkyl CH_2), 28.7 (C26), 27.2 (C29), 27.1 (C3), 26.9 (C10), 16.0 (C44). ^{19}F NMR (376 MHz, CD_3OD) δ_F ppm -116.7. HRMS (ESI) m/z: $[M+H]^+$ calculated for $C_{52}H_{69}FN_7O_8S$: 970.4912, found 970.4913.

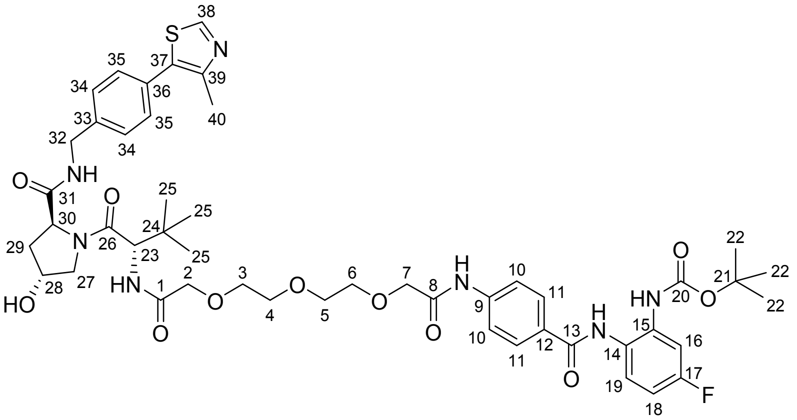

Tert-butyl (5-fluoro-2-(4-((S)-13-((2S,4R)-4-hydroxy-2-((4-(4-methylthiazol-5-yl)benzyl)carbamoyl) pyrrolidine-1-carbonyl)-14,14-dimethyl-11-oxo-3,6,9-trioxa-12-azapentadecanamido)benzamido) phenyl)carbamate (54p): Following general method G, **54p** was obtained from **53p** (52.2 mg, 0.095 mmol) and **VH_032 amine** (40.0 mg, 0.080 mmol). The crude product was purified by column chromatography (0-8% MeOH in DCM) to afford **54p** (65.8 mg, 0.068 mmol, 85% yield) as a pale yellow/white solid. ^1H NMR (400 MHz, CD_3OD) δ_H ppm 8.85 (s, 1 H, 38-CH), 7.94 (d, *J*=8.7 Hz, 2 H, 11-CH), 7.76 (d, *J*=8.7 Hz, 2 H, 10-CH), 7.41 - 7.48 (m, 4H, 16-CH,19-CH,35-CH), 7.37 - 7.41 (m, 2 H, 34-CH), 6.90 (ddd, *J*=8.9, 8.1, 2.9 Hz, 1 H, 18-CH), 4.67 - 4.71 (m, 1 H, 23-CH), 4.56 - 4.60 (m, 1 H, 30-CH), 4.53 (d, *J*=15.5 Hz, 1 H, 32-CH), 4.47 - 4.50 (m, 1 H, 28-CH), 4.34 (d, *J*=15.5 Hz, 1 H, 32-CH), 4.13 - 4.18 (m, 1 H, 7-CH), 4.08 - 4.13 (m, 1 H, 7-CH), 4.04 (d, *J*=15.6 Hz, 1 H, 2-CH), 3.94 (d, *J*=15.6 Hz, 1 H, 2-CH), 3.83 - 3.88 (m, 1 H, 27-CH), 3.70 - 3.82 (m, 9 H, 27-CH,(3-6)-CH_2), 2.46 (s, 3 H, 40-CH_3), 2.16 - 2.25 (m, 1 H, 29-CH), 2.02 - 2.13 (m, 1 H, 29-CH), 1.48 (s, 9 H, 22-CH_3), 1.02 (s, 9 H, 25-CH_3). ^{13}C NMR (101 MHz, CD_3OD) δ_C ppm 174.5 (C31), 172.0 (C1), 171.7 (C26), 171.6 (C8), 168.3 (C13), 162.3 (d, J_{CF}=242.8 Hz, C17), 155.5 (C20), 153.0 (C38), 149.2 (C39), 142.7 (C9), 140.4 (C33), 135.9 (d, J_{CF}=11.3 Hz, C15), 133.5 (C37), 131.7 (C36), 130.8 (C12), 130.5 (C34), 129.9 (C11), 129.3 (d, J_{CF}=9.5 Hz, C19), 129.1 (C35), 126.5 (d, J_{CF}=2.9 Hz, C14), 121.1 (C10), 112.0 (d, J_{CF}=22.7 Hz, C18), 111.0 (d, J_{CF}=26.9 Hz, C16), 82.1 (C21), 72.3 (2xCH_2), 71.7 (C7), 71.65 (CH_2), 71.6 (CH_2), 71.2 (C28), 71.1 (C2), 61.0 (C30), 58.3 (C23), 58.2 (C27), 43.9 (C32), 39.1 (C29), 37.3 (C24), 28.7 (C22), 27.1 (C25), 16.0 (C40). ^{19}F NMR (376 MHz, CD_3OD) δ_F ppm -116.6. HRMS (ESI) m/z: $[M+H]^+$ calculated for $C_{48}H_{61}FN_7O_{11}S$: 962.4134, found 962.4134.

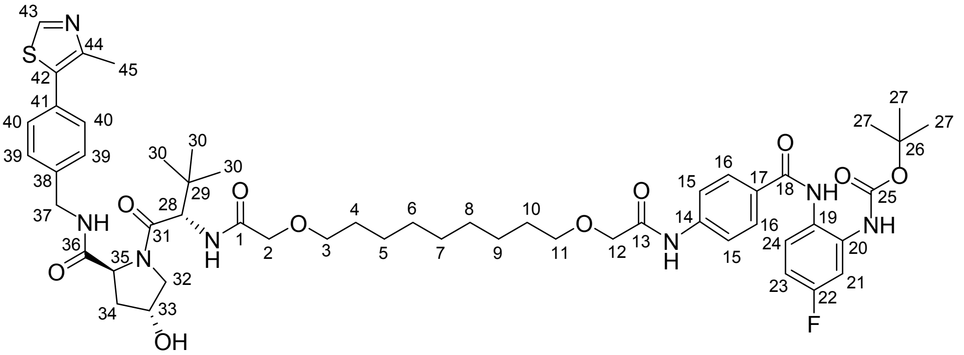

Tert-butyl (5-fluoro-2-(4-(2-((9-(2-(((S)-1-((2S,4R)-4-hydroxy-2-((4-(4-methylthiazol-5-yl)benzyl) carbamoyl)pyrrolidin-1-yl)-3,3-dimethyl-1-oxobutan-2-yl)amino)-2-oxoethoxy)nonyl)oxy)acetamido) benzamido)phenyl)carbamate (54q): Following general method G, **54q** was obtained from **53q** (57.6 mg, 0.096 mmol) and **VH_032 amine** (40.0 mg, 0.080 mmol). The crude product was purified by column chromatography (0-8% MeOH in DCM) to afford **54q** (75.7, 0.072 mmol, 90% yield) as a pale yellow/white solid. ^{1}H NMR (400 MHz, CD_3OD) δ_H ppm 8.85 (s, 1 H, 43-CH), 7.95 (d, J=8.7 Hz, 2 H, 16-CH), 7.77 (d, J=8.7 Hz, 2 H, 15-CH), 7.41 - 7.48 (m, 4 H, 21,24,40-CH), 7.37 - 7.41 (m, 2 H, 39-CH), 6.89 (ddd, J_{HH}=8.8, 2.9, J_{HF}=7.9 Hz, 1 H, 23-CH), 4.68 (s, 1 H, 28-CH), 4.59 (dd, J=9.0, 7.8 Hz, 1 H, 35-CH), 4.46 - 4.56 (m, 2 H, 33,37-CH), 4.34 (d, J=15.6 Hz, 1 H, 37-CH), 4.07 (s, 2 H, 12-CH_2), 3.94 - 3.99 (m, 1 H, 2-CH), 3.89 - 3.94 (m, 1 H, 2-CH), 3.84 - 3.89 (m, 1 H, 32-CH), 3.74 - 3.83 (m, 1 H, 32-CH), 3.49 - 3.60 (m, 4 H, 3,11-CH_2), 2.46 (s, 3 H, 45-CH_3), 2.19 - 2.27 (m, 1 H, 34-CH), 2.03 - 2.13 (m, 1 H, 34-CH), 1.58 - 1.68 (m, 4 H, 4,10-CH_2), 1.48 (s, 9 H, 27-CH_3), 1.32 - 1.42 (m, 10 H, (5-9)-CH_2), 1.03 (s, 9 H, 30-CH_3). ^{13}C NMR (101 MHz, CD_3OD) δ_C ppm 174.4 (C36), 172.1 (C1), 171.8 (C31), 171.4 (C13), 168.2 (C18), 162.2 (d, J_{CF}=243.0 Hz, C22), 155.5 (C25), 152.9 (C43), 149.1 (C44), 142.8 (C14), 140.3 (C38), 135.8 (d, J_{CF}=11.3 Hz, C20), 133.5 (C42), 131.6 (C41), 130.6 (C17), 130.5 (C39), 129.9 (C16), 129.3 (d, J_{CF}=9.5 Hz, C24), 129.0 (C40), 126.5 (d, J_{CF}=3.1 Hz, C19), 120.9 (C15), 112.0 (d, J_{CF}=22.9 Hz, C23), 111.0 (d, J_{CF}=26.7 Hz, C21), 82.1 (C26), 73.2 (C11), 73.1 (C3), 71.5 (C12), 71.2 (C33), 70.8 (C2), 60.9 (C35), 58.3 (C32), 58.1 (C28), 43.8 (C37), 39.1 (C34), 37.3 (C29), 30.8 (C4/10), 30.75 (C4/10), 30.5 (alkyl CH_2), 30.6 (2 x alkyl CH_2), 28.7 (C27), 27.4 (C5/9), 27.2 (C5/9), 27.1 (C30), 16.1 (C45). ^{19}F NMR (376 MHz, CD_3OD) δ_F ppm -116.6. HRMS (ESI) m/z: $[M+H]^+$ calculated for $C_{53}H_{71}FN_7O_{10}S$: 1016.4967, found 1016.4928.

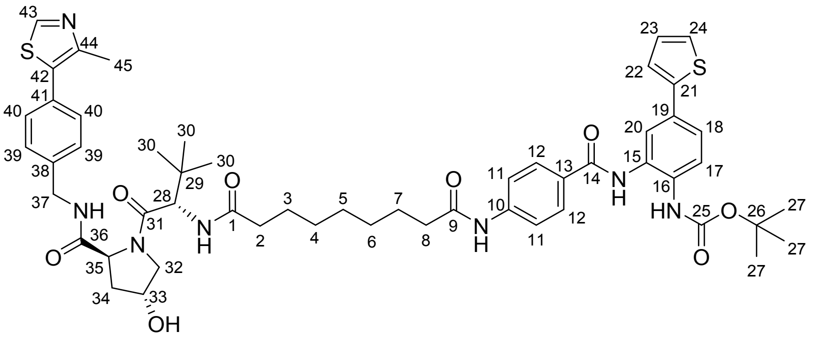

Tert-butyl (2-(4-(9-(((S)-1-((2S,4R)-4-hydroxy-2-((4-(4-methylthiazol-5-yl)benzyl)carbamoyl) pyrrolidin-1-yl)-3,3-dimethyl-1-oxobutan-2-yl)amino)-9-oxononanamido)benzamido)-4-(thiophen-2-yl)phenyl)carbamate (54r): Following general method G, **54r** was obtained from **53r** (30.7 mg, 0.053 mmol) and **VH_032 amine** (23.8 mg, 0.063 mmol). The crude product was purified by column chromatography (0-

10% MeOH in DCM) to afford **54r** (46.3 mg, 0.046 mmol, 96% yield) as a pale brown solid. ^{1}H NMR (400 MHz, CD_3OD) δ_H ppm 8.84 (s, 1 H, 43-CH), 7.94 (d, *J*=8.7 Hz, 2 H, 12-CH), 7.90 (d, *J*=1.1 Hz, 1 H, 20-CH), 7.73 (d, *J*=8.7 Hz, 2 H, 11-CH), 7.46 - 7.50 (m, 2 H, 17,18-CH), 7.41 - 7.46 (m, 2 H, 40-CH), 7.37 - 7.41 (m, 2 H, 39-CH), 7.33 - 7.37 (m, 2 H, 22,24-CH), 7.07 (dd, *J*=5.1, 3.7 Hz, 1 H, 23-CH), 4.62 - 4.66 (m, 1 H, 28-CH), 4.55 - 4.61 (m, 1 H, 35-CH), 4.51 (d, *J*=15.5 Hz, 1 H, 37-CH), 4.46 - 4.49 (m, 1 H, 33-CH), 4.34 (d, *J*=15.5 Hz, 1 H, 37-CH), 3.87 - 3.94 (m, 1 H, 32-CH), 3.75 - 3.82 (m, 1 H, 32-CH), 2.45 (s, 3 H, 45-CH_3), 2.39 (t, *J*=7.5 Hz, 2 H, 8-CH_2), 2.16 - 2.32 (m, 3 H, 2-CH_2,34-CH), 2.02 - 2.11 (m, 1 H, 34-CH), 1.65 - 1.74 (m, 2 H, 7-CH), 1.56 - 1.65 (m, 2 H, 3-CH), 1.50 (s, 9 H, 27-CH_3), 1.32 - 1.40 (m, 6 H, (4-6)-CH_2), 1.03 (s, 9 H, 30-CH_3). ^{13}C NMR (101 MHz, CD_3OD) δ_C ppm 176.2 (C1), 175.1 (C9), 174.6 (C36), 172.5 (C31), 167.9 (C14), 156.3 (C25), 152.9 (C43), 149.1 (C44), 144.6 (C21), 144.0 (C10), 140.4 (C38), 133.5 (C42), 133.0 (C19), 132.3 (C16), 132.0 (C15), 131.6 (C41), 130.5 (C39), 130.2 (C13), 129.8 (C12), 129.3 (C23), 129.1 (C40), 126.1 (C24), 126.0 (C17), 124.65 (C18), 124.6 (C22), 124.2 (C20), 120.5 (C11), 82.0 (C26), 71.2 (C33), 61.0 (C35), 59.1 (C28), 58.2 (C32), 43.8 (C37), 39.1 (C34), 38.2 (C8), 36.75 (C2), 36.7 (C29), 30.3 (C4/5/6), 30.25 (C4/5/6), 30.2 (C4/5/6), 28.8 (C27), 27.2 (C30), 27.1 (C3), 26.8 (C7), 16.0 (C45). HRMS (ESI) m/z: $[M+H]^+$ calculated for $C_{53}H_{66}N_7O_8S_2$: 992.4414, found 992.4380.

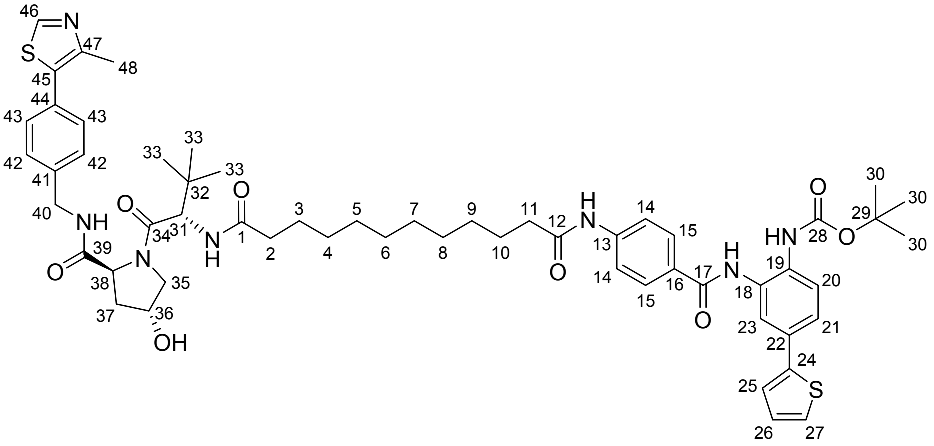

Tert-butyl (2-(4-(12-(((S)-1-((2S,4R)-4-hydroxy-2-((4-(4-methylthiazol-5-yl)benzyl)carbamoyl) pyrrolidin-1-yl)-3,3-dimethyl-1-oxobutan-2-yl)amino)-12-oxododecanamido)benzamido)-4-(thiophen-2-yl)phenyl)carbamate (54s): Following general method G, **54s** was obtained from **53s** (18.2 mg, 0.029 mmol) and **VH_032 amine** (14.7 mg, 0.029 mmol). The crude product was purified by column chromatography (5% MeOH in DCM) to afford **54s** (26.1 mg, 0.025 mmol, 86% yield) as a pale yellow solid. ^{1}H NMR (400 MHz, CD_3OD) δ_H ppm 8.86 (s, 1 H, 46-CH), 7.96 (d, *J*=8.7 Hz, 2 H, 15-CH), 7.91 (d, *J*=1.1 Hz, 1 H, 23-CH), 7.79 (d, *J*=9.1 Hz, 1 H, 31-NH), 7.74 (d, *J*=8.7 Hz, 2 H, 14-CH), 7.47 - 7.51 (m, 2 H, 20,21-CH), 7.44 (d, *J*=8.4 Hz, 2 H, 43-CH), 7.39 (d, *J*=8.4 Hz, 2 H, 42-CH), 7.34 - 7.38 (m, 2 H, 25,27-CH), 7.07 (dd, *J*=5.1, 3.6 Hz, 1 H, 26-CH), 4.61 - 4.66 (m, 1 H, 31-CH), 4.54 - 4.60 (m, 1 H, 38-CH), 4.52 (d, *J*=15.5 Hz, 1 H, 40-CH), 4.46 - 4.49 (m, 1 H, 36-CH), 4.34 (d, *J*=15.5 Hz, 1 H, 40-CH), 3.84 - 3.94 (m, 1 H, 35-CH), 3.76 - 3.82 (m, 1 H, 35-CH), 2.46 (s, 3 H, 48-CH_3), 2.40 (t, *J*=7.5 Hz, 2 H, 11-CH_2), 2.17 - 2.32 (m, 3 H, 2-CH_2,37-CH), 2.03 - 2.11 (m, 1 H, 37-CH), 1.70 (quin, *J*=7.5 Hz, 2 H, 10-CH_2), 1.55 - 1.64 (m, 2 H, 3-CH_2), 1.50 (s, 9 H, 30-CH_3), 1.29 - 1.38 (m, 12 H, (4-9)-CH_2), 1.03 (s, 9 H, 33-CH_3). ^{13}C NMR (101 MHz, CD_3OD) δ_C ppm 176.2 (C1), 175.1 (C12), 174.6 (C39), 172.5 (C34), 168.0 (C17), 156.3 (C28), 153.0 (C46), 149.2

(C47), 144.6 (C24), 144.0 (C13), 140.4 (C41), 133.6 (C45), 133.0 (C22), 132.3 (C19), 132.1 (C18), 131.6 (C44), 130.5 (C42), 130.2 (C16), 129.8 (C15), 129.3 (C26), 129.1 (C43), 126.1 (C27), 126.0 (C20), 124.65 (C21), 124.6 (C25), 124.2 (C23), 120.5 (C14), 82.0 (C29), 71.2 (C36), 61.0 (C38), 59.1 (C31), 58.2 (C35), 43.8 (C40), 39.1 (C37), 38.2 (C11), 36.8 (C2), 36.7 (C31), 30.6 (alkyl CH_2), 30.5 (alkyl CH_2), 30.5 (alkyl CH_2), 30.45 (alkyl CH_2), 30.4 (2 x alkyl CH_2), 28.8 (C30), 27.2 (C33), 27.1 (C3), 26.9 (C10), 16.0 (C48). HRMS (ESI) m/z: $[M+H]^+$ calculated for $C_{56}H_{72}N_7O_8S_2$: 1034.4884, found 1034. 4883.

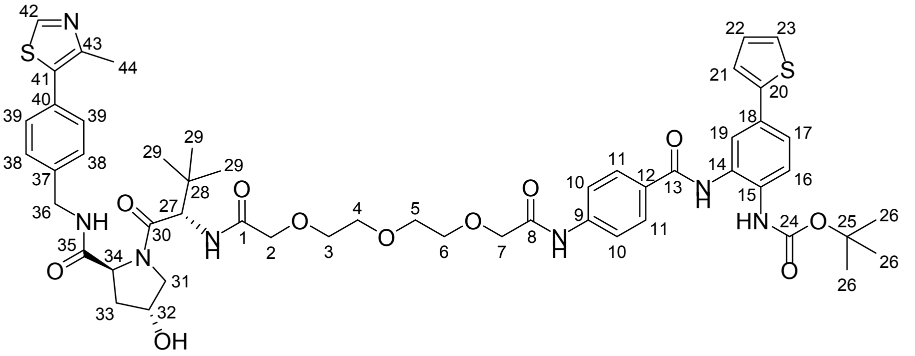

Tert-butyl (2-(4-((S)-13-((2S,4R)-4-hydroxy-2-((4-(4-methylthiazol-5-yl)benzyl)carbamoyl)pyrrolidine-1-carbonyl)-14,14-dimethyl-11-oxo-3,6,9-trioxa-12-azapentadecanamido)benzamido)-4-(thiophen-2-yl)phenyl)carbamate (54t): Following general method G, **54t** was obtained from **53t** (35.1 mg, 0.057 mmol) and **VH_032 amine** (24.0 mg, 0.048 mmol). The crude product was purified by column chromatography (0-10% MeOH in DCM) to afford **54t** (30.3 mg, 0.029 mmol, 61% yield) as a yellow tar. ^{1}H NMR (400 MHz, CD_3OD) δ_H ppm 8.83 (s, 1 H, 42-CH), 7.96 (d, *J*=8.7 Hz, 2 H, 11-CH), 7.86 - 7.93 (m, 1 H, 19-CH), 7.77 (d, *J*=8.7 Hz, 2 H, 10-CH), 7.46 - 7.51 (m, 2 H, 16,17-CH), 7.42 (d, *J*=8.4 Hz, 2 H, 39-CH), 7.33 - 7.39 (m, 4 H, 21,23,38-CH), 7.07 (dd, *J*=5.1, 3.6 Hz, 1 H, 22-CH), 4.69 (s, 1 H, 27-CH), 4.54 - 4.60 (m, 1 H, 34-CH), 4.51 (d, *J*=15.5 Hz, 1 H, 36-CH), 4.46 - 4.49 (m, 1 H, 32-CH), 4.30 (d, *J*=15.5 Hz, 1 H, 36-CH), 4.15 (d, *J*=15.7 Hz, 1 H, 7-CH), 4.10 (d, *J*=15.7 Hz, 1 H, 7-CH), 4.04 (d, *J*=15.6 Hz, 1 H, 2-CH), 3.94 (d, *J*=15.6 Hz, 1 H, 2-CH), 3.82 - 3.87 (m, 1 H, 31-CH), 3.70 - 3.81 (m, 9 H, 31-CH,(3-6)-CH_2), 2.44 (s, 3 H, 44-CH_3), 2.16 - 2.25 (m, 1 H, 33-CH), 2.03 - 2.13 (m, 1 H, 33-CH), 1.49 (s, 9 H, 26-CH_3), 1.02 (s, 9 H, 29-CH_3). ^{13}C NMR (101 MHz, CD_3OD) δ_C 174.5 (C35), 172.0 (C30), 171.7 (C1), 171.6 (C8), 167.9 (C13), 156.2 (C24), 152.9 (C42), 149.2 (C43), 144.6 (C20), 142.8 (C9), 140.3 (C37), 133.5 (C41), 132.9 (C18), 132.4 (C15), 132.0 (C14), 131.6 (C40), 130.9 (C12), 130.5 (C38), 129.9 (C11), 129.3 (C22), 129.1 (C39), 126.1 (C23), 126.0 (C16), 124.7 (C17), 124.6 (C21), 124.2 (C19), 121.2 (C10), 82.0 (C25), 72.3 (C3/4/5/6), 72.25 (C3/4/5/6), 71.7 (C7), 71.6 (C3/4/5/6), 71.55 (C3/4/5/6), 71.2 (C32), 71.1 (C2), 60.9 (C34), 58.2 (C31), 43.9 (C36), 39.1 (C33), 37.8 (C28), 37.3 (C27), 28.8 (C26), 27.1 (C29), 16.0 (C44). HRMS (ESI) m/z: $[M+H]^+$ calculated for $C_{52}H_{64}N_7O_{11}S_2$: 1026.4105, found 1026.4066.

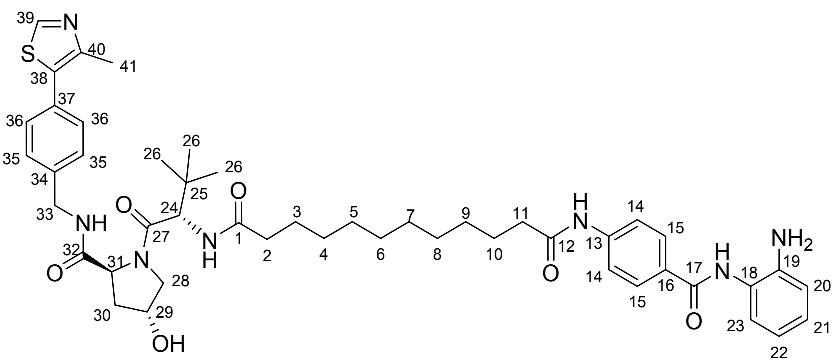

N1-(4-((2-aminophenyl)carbamoyl)phenyl)-N12-((S)-1-((2S,4R)-4-hydroxy-2-((4-(4-methylthiazol-5-yl)benzyl)carbamoyl)pyrrolidin-1-yl)-3,3-dimethyl-1-oxobutan-2-yl)dodecanediamide (1): Following general method H, Boc deprotection of **54a** (37.6 mg, 0.0395 mmol) was performed to afford **1** (25.8 mg, 0.0288 mmol, 73% yield) as a pale yellow solid. Prior to biological evaluation the PROTAC was further purified by semi-preparative HPLC (5-95% MeCN in H_2O, 260 nm, 45 min gradient). ^{1}H NMR (400 MHz, CD_3OD) δ_H ppm 8.87 (s, 1 H, 39-CH), 7.95 (d, *J*=8.8 Hz, 2 H, 15-CH), 7.72 (d, *J*=8.8 Hz, 2 H, 14-CH), 7.43 - 7.48 (m, 2 H, 36-CH), 7.38 - 7.42 (m, 2 H, 35-CH), 7.18 (dd, *J*=7.8, 1.3 Hz, 1 H, 23-CH), 7.07 (app. td, *J*=7.8, 1.3 Hz, 1 H, 21-CH), 6.90 (dd, *J*=7.8, 1.3 Hz, 1 H, 20-CH), 6.76 (app. td, *J*=7.8, 1.3 Hz, 1 H, 22-CH), 4.60 - 4.66 (m, 1 H, 24-CH), 4.55 - 4.60 (m, 1 H, 31-CH), 4.50 - 4.55 (m, 1 H, 33-CH), 4.47 - 4.50 (m, 1 H, 29-CH), 4.31 - 4.39 (m, 1 H, 33-CH), 3.86 - 3.93 (m, 1 H, 28-CH), 3.76 - 3.83 (m, 1 H, 28-CH), 2.47 (s, 3 H, 41-CH_3), 2.40 (t, *J*=7.5 Hz, 2 H, 11-CH_2), 2.18 - 2.33 (m, 3 H, 2-CH_2,30-CH), 2.03 - 2.12 (m, 1 H, 30-CH), 1.70 (quin, *J*=7.5 Hz, 2 H, 10-CH_2), 1.53 - 1.64 (m, 2 H, 3-CH_2), 1.28 - 1.41 (m, 12 H, (4-9)-CH_2), 1.03 (s, 9 H, 26-CH_3). ^{13}C NMR (101 MHz, CD_3OD) δ_C ppm 176.2 (C1), 175.1 (C12), 174.6 (C32), 172.5 (C27), 168.4 (C17), 153.0 (C39), 149.2 (C40), 144.0 (C19), 143.7 (C13), 140.4 (C34), 133.6 (C38), 131.7 (C37), 130.6 (C16), 130.5 (C35), 129.9 (C15), 129.1 (C36), 128.6 (C21), 127.8 (C23), 125.6 (C18), 120.4 (C14), 119.8 (C22), 118.9 (C20), 71.2 (C29), 61.0 (C31), 59.1 (C24), 58.2 (C28), 43.8 (C33), 39.1 (C30), 38.2 (C11), 36.8 (C25), 36.7 (C2), 30.7 (alkyl CH_2), 30.6 (alkyl CH_2), 30.55 (alkyl CH_2), 30.5 (alkyl CH_2), 30.4 (2x alkyl CH_2), 27.2 (C26), 27.1 (C3), 26.9 (C10), 16.0 (C41). HRMS (ESI) m/z: $[M+H]^+$ calculated for $C_{47}H_{62}N_7O_6S$: 852.4476, found 852.4482.[12]

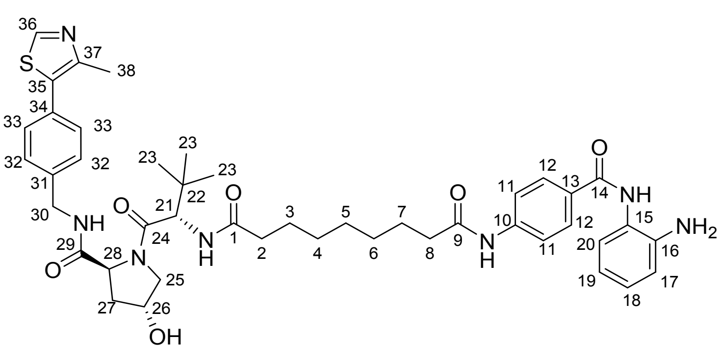

N1-(4-((2-aminophenyl)carbamoyl)phenyl)-N9-((S)-1-((2S,4R)-4-hydroxy-2-((4-(4-methylthiazol-5-yl)benzyl)carbamoyl)pyrrolidin-1-yl)-3,3-dimethyl-1-oxobutan-2-yl)nonanediamide (2): Following general method H, Boc deprotection of **54b** (37.6 mg, 0.0395 mmol) was performed to afford **2** (37.0 mg, 0.044 mmol, 99% yield) as an off-white solid. Prior to biological evaluation the PROTAC was further purified by semi-preparative HPLC (5-95% MeCN in H_2O, 260 nm, 45 min gradient). ^{1}H NMR (400 MHz, CD_3OD)

δ_H ppm 8.87 (s, 1 H, 36-CH), 7.95 (d, *J*=8.7 Hz, 2 H, 12-CH), 7.81 (d, *J*=8.9 Hz, 1 H, 1-NH), 7.72 (d, *J*=8.7 Hz, 2 H, 11-CH), 7.45 (d, *J*=8.4 Hz, 2 H, 33-CH), 7.40 (d, *J*=8.4 Hz, 2 H, 32-CH), 7.18 (app. dd, *J*=7.8, 1.3 Hz, 1 H, 20-CH), 7.07 (app. td, *J*=7.8, 1.3 Hz, 1 H, 18-CH), 6.90 (app. dd, *J*=7.8, 1.3 Hz, 1 H, 17-CH), 6.76 (app. td, *J*=7.8, 1.3 Hz, 1 H, 19-CH), 4.62 - 4.66 (m, 1 H, 21-CH), 4.55 - 4.60 (m, 1 H, 28-CH), 4.51 - 4.55 (m, 1 H, 30-CH), 4.48 - 4.50 (m, 1 H, 29-CH), 4.33 - 4.38 (m, 1 H, 30-CH), 3.87 - 3.94 (m, 1 H, 25-CH), 3.77 - 3.83 (m, 1 H, 25-CH), 2.47 (s, 3 H, 38-CH_3), 2.39 (t, *J*=7.5 Hz, 2 H, 8-CH_3), 2.18 - 2.32 (m, 3 H, 2-CH_2,27-CH), 2.03 - 2.11 (m, 1 H, 27-CH), 1.71 (quin, *J*=7.5 Hz, 2 H, 7-CH_2), 1.62 (quin, *J*=6.9 Hz, 2 H, 3-CH_2), 1.33 - 1.42 (m, 6 H, (4-6)-CH_2), 1.03 (s, 9 H, 23-CH_3). ^{13}C NMR (101 MHz, CD_3OD) δ_C ppm 176.2 (C1), 175.1 (C9), 174.6 (C29), 172.5 (C24), 168.4 (C17), 153.0 (C36), 149.2 (C37), 143.9 (C16), 143.6 (C10), 140.4 (C31), 133.6 (C35), 131.6 (C34), 130.6 (C13), 130.5 (C32), 129.9 (C12), 129.1 (C33), 128.6 (C18), 127.8 (C20), 125.5 (C15), 120.4 (C11), 119.8 (C19), 118.9 (C17), 71.2 (C26), 61.0 (C28), 59.1 (C21), 58.2 (C25), 43.8 (C30), 39.1 (C27), 38.2 (C8), 36.75 (C22), 36.7 (C2), 30.3 (C4/5/6), 30.25 (C4/5/6), 30.2 (C4/5/6), 27.2 (C23), 27.1 (C3), 26.8 (C7), 16.0 (C38). HRMS (ESI) m/z: $[M+H]^+$ calculated for $C_{44}H_{56}N_7O_6S$: 810.4013, found 810.4005.

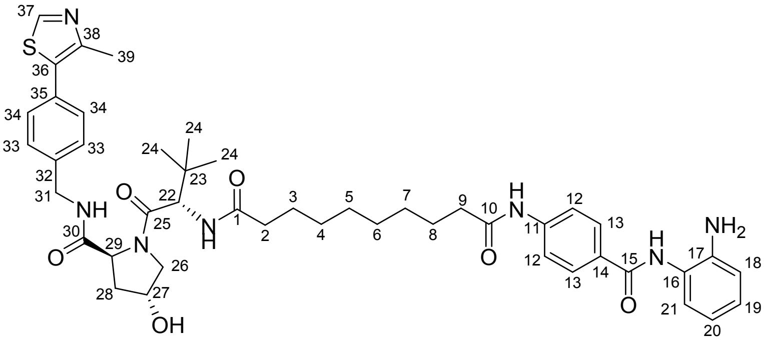

N1-(4-((2-aminophenyl)carbamoyl)phenyl)-N10-((S)-1-((2S,4R)-4-hydroxy-2-((4-(4-methylthiazol5-yl)benzyl)carbamoyl)pyrrolidin-1-yl)-3,3-dimethyl-1-oxobutan-2-yl)decanediamide (3): Following general method H, Boc deprotection of **54c** (68.5 mg, 0.074 mmol) was performed to afford **3** (49.3 mg, 0.060 mmol, 81% yield) as a white solid. Prior to biological evaluation the PROTAC was further purified by semi-preparative HPLC (5-95% MeCN in H_2O, 260 nm, 45 min gradient). ^{1}H NMR (400 MHz, CD_3OD) δ_H ppm 8.87 (s, 1 H, 37-CH), 7.95 (d, *J*=8.7 Hz, 2 H, 13-CH), 7.72 (d, *J*=8.7 Hz, 2 H, 12-CH), 7.44 - 7.48 (m, 2 H, 34-CH), 7.39 - 7.43 (m, 2 H, 33-CH), 7.18 (dd, *J*=7.7, 1.4 Hz, 1 H, 21-CH), 7.08 (app. td, *J*=7.7, 1.4 Hz, 1 H, 19-CH), 6.90 (dd, *J*=7.7, 1.4 Hz, 1 H, 18-CH), 6.77 (app. td, *J*=7.7, 1.4 Hz, 1 H, 20-CH), 4.64 (s, 1 H, 22-CH), 4.56 - 4.60 (m, 1 H, 29-CH), 4.53 (d, *J*=15.1 Hz, 1 H, 31-CH), 4.47 - 4.50 (m, 1 H, 27-CH), 4.35 (d, *J*=15.5 Hz, 1 H, 31-CH), 3.86 - 3.94 (m, 1 H, 26-CH), 3.76 - 3.84 (m, 1 H, 26-CH), 2.47 (s, 3 H, 39-CH_3), 2.40 (t, *J*=7.4 Hz, 2 H, 9-CH_2), 2.19 - 2.32 (m, 3 H, 2-CH_2,28-CH), 2.04 - 2.12 (m, 1 H, 28-CH), 1.67 - 1.76 (m, 2 H, 8-CH_2), 1.55 - 1.65 (m, 2 H, 3-CH_2), 1.33 - 1.39 (m, 8 H, (4-7)-CH_2), 1.03 (s, 9 H, 24-CH_3). ^{13}C NMR (101 MHz, CD_3OD) δ_C ppm 176.2 (C1), 175.1 (C10), 174.6 (C30), 172.5 (C25), 168.3 (C15), 153.0 (C37), 149.1 (38C), 143.8 (C17), 143.6 (C11), 140.4 (C32), 133.7 (C36), 131.6 (C35), 130.6 (C14), 130.5 (C33), 129.9 (C13), 129.1 (C34), 128.6 (C19), 127.8 (C21), 125.6 (C16), 120.4 (C12), 119.9 (C20), 118.9 (C18), 71.2 (C27), 61.0 (C29), 59.1 (C22), 58.2 (C26), 43.8 (C31), 39.1 (C28), 38.2 (C9), 36.8 (C23), 36.7 (C2), 30.4 (alkyl CH_2),

30.35 (alkyl CH_2), 30.3 (alkyl CH_2), 30.25 (alkyl CH_2), 27.2 (C24), 27.1 (C3), 26.9 (C8), 16.0 (C39). HRMS (ESI) m/z: $[M+H]^+$ calculated for $C_{45}H_{58}N_7O_6S$: 824.4169, found 824.4160.

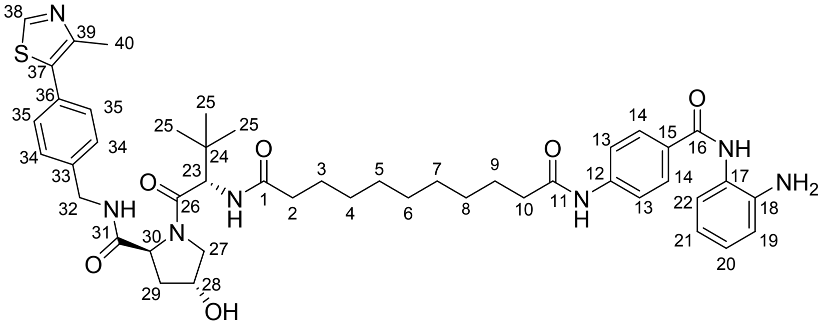

N1-(4-((2-aminophenyl)carbamoyl)phenyl)-N11-((S)-1-((2S,4R)-4-hydroxy-2-((4-(4-methylthiazol5-yl)benzyl)carbamoyl)pyrrolidin-1-yl)-3,3-dimethyl-1-oxobutan-2-yl)undecanediamide (4): Following general method H, Boc deprotection of **54d** (73.0 mg, 0.077 mmol) was performed to afford **4** (51.1 mg, 0.061 mmol, 79% yield) as a white solid. Prior to biological evaluation the PROTAC was further purified by semi-preparative HPLC (5-95% MeCN in H_2O, 260 nm, 45 min gradient). ^{1}H NMR (400 MHz, CD_3OD) δ_H ppm 8.86 (s, 1 H, 38-CH), 7.95 (d, *J*=8.7 Hz, 2 H, 14-CH), 7.80 (d, *J*=9.0 Hz, 1 H, 1-NH), 7.72 (d, *J*=8.7 Hz, 2 H, 13-CH), 7.43 - 7.47 (m, 2 H, 35-CH), 7.38 - 7.42 (m, 2 H, 34-CH), 7.18 (dd, *J*=7.8, 1.3 Hz, 1 H, 22-CH), 7.07 (app. td, *J*=7.8, 1.3 Hz, 1 H, 20-CH), 6.90 (dd, *J*=7.8, 1.3 Hz, 1 H, 19-CH), 6.77 (app. td, *J*=7.8, 1.3 Hz, 1 H, 21-CH), 4.64 (d, *J*=9.0 Hz, 1 H, 23-CH), 4.55 - 4.60 (m, 1 H, 30-CH), 4.52 (d, *J*=15.5 Hz, 1 H, 32-CH), 4.46 - 4.50 (m, 1 H, 28-CH), 4.35 (d, *J*=15.5 Hz, 1 H, 32-CH), 3.86 - 3.93 (m, 1 H, 27-CH), 3.75 - 3.82 (m, 1 H, 27-CH), 2.46 (s, 3 H, 40-CH_3), 2.39 (t, *J*=7.5 Hz, 2 H, 10-CH_2), 2.17 - 2.31 (m, 3 H, 2-CH_2,29-CH), 2.03 - 2.11 (m, 1 H, 29-CH), 1.70 (quin, *J*=7.5 Hz, 2 H, 9-CH_2), 1.55 - 1.64 (m, 2 H, 3-CH_2), 1.30 - 1.39 (m, 10 H, (4-8)-CH_2), 1.03 (s, 9 H, 25-CH_3). ^{13}C NMR (101 MHz, CD_3OD) δ_C ppm 176.2 (C1), 175.1 (C11), 174.6 (C31), 172.5 (C26), 168.4 (C16), 153.0 (C38), 149.2 (C39), 143.9 (C18), 143.6 (C12), 140.4 (C33), 133.6 (C37), 131.6 (C36), 130.6 (C15), 130.5 (C34), 129.9 (C14), 129.1 (C35), 128.6 (C20), 127.8 (C22), 125.6 (C17), 120.4 (C13), 119.8 (C21), 118.9 (C19), 71.2 (C28), 61.0 (C30), 59.1 (C23), 58.2 (C27), 43.8 (C32), 39.1 (C29), 38.2 (C10), 36.8 (C24), 36.7 (C2), 30.5 (alkyl CH_2), 30.45 (2 x alkyl CH_2), 30.4 (2 x alkyl CH_2), 27.2 (C25), 27.1 (C3), 26.9 (C9), 16.0 (C40). HRMS (ESI) m/z: $[M+H]^+$ calculated for $C_{46}H_{60}N_7O_6S$: 838.4326, found 838.4329.

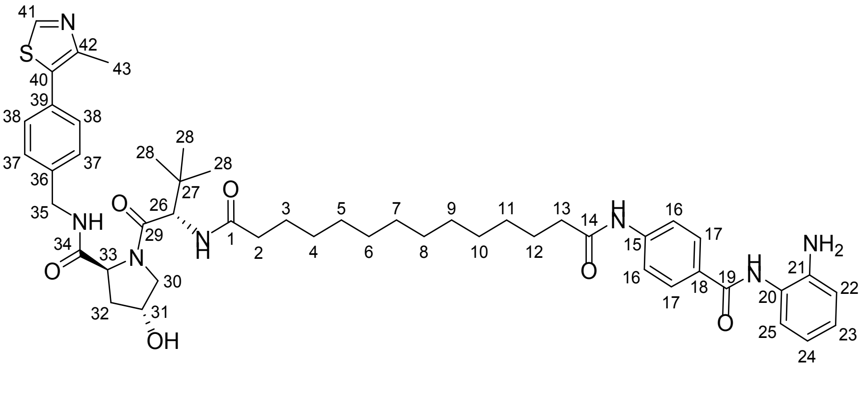

N1-(4-((2-aminophenyl)carbamoyl)phenyl)-N14-((S)-1-((2S,4R)-4-hydroxy-2-((4-(4-methylthiazol-5-yl)benzyl)carbamoyl)pyrrolidin-1-yl)-3,3-dimethyl-1-oxobutan-2-yl)tetradecanediamide (5): Following

general method H, Boc deprotection of **54e** (62.5 mg, 0.064 mmol) was performed to afford **5** (51.6 mg, 0.058 mmol, 91% yield) as a white solid. ^{1}H NMR (400 MHz, CD_3OD) δ_H ppm 8.86 (s, 1 H, 41-CH), 7.95 (d, *J*=8.7 Hz, 2 H, 17-CH), 7.72 (d, *J*=8.7 Hz, 2 H, 16-CH), 7.43 - 7.47 (m, 2 H, 38-CH), 7.38 - 7.43 (m, 2 H, 37-CH), 7.18 (dd, *J*=7.8, 1.3 Hz, 1 H, 25-CH), 7.06 (app. td, *J*=7.8, 1.3 Hz, 1 H, 23-CH), 6.90 (dd, *J*=7.8, 1.3 Hz, 1 H, 22-CH), 6.76 (app. td, *J*=7.8, 1.3 Hz, 1 H, 24-CH), 4.63 (s, 1 H, 26-CH), 4.55 - 4.60 (m, 1 H, 33-CH), 4.52 (d, *J*=15.5 Hz, 1 H, 35-CH), 4.46 - 4.50 (m, 1 H, 31-CH), 4.34 (d, *J*=15.5 Hz, 1 H, 35-CH), 3.86 - 3.93 (m, 1 H, 30-CH), 3.75 - 3.82 (m, 1 H, 30-CH), 2.46 (s, 3 H, 43-CH_3), 2.39 (t, J=7.4 Hz, 2 H, 13-CH_2), 2.17 - 2.32 (m, 3 H, 2-CH_2,32-CH), 2.03 - 2.11 (m, 1 H, 32-CH), 1.70 (quin, *J*=7.4 Hz, 2 H, 12-CH_2), 1.52 - 1.65 (m, 2 H, 3-CH_2), 1.27 - 1.39 (m, 16 H, (4-11)-CH_2), 1.03 (s, 9 H, 28-CH_3). ^{13}C NMR (101 MHz, CD_3OD) δ_C ppm 176.2 (C1), 175.1 (C14), 174.6 (C34), 172.5 (C29), 168.4 (C19), 153.0 (C41), 149.2 (C42), 143.9 (C21), 143.6 (C15), 140.4 (C36), 133.6 (C40), 131.6 (C39), 130.6 (C18), 130.5 (C37), 129.9 (C17), 129.1 (C38), 128.6 (C23), 127.8 (C25), 125.6 (C20), 120.4 (C16), 119.8 (C24), 118.9 (C22), 71.2 (C31), 61.0 (C33), 59.1 (C26), 58.2 (C30), 43.8 (C35), 39.0 (C32), 38.2 (C13), 36.8 (C27), 36.7 (C2), 30.8 (alkyl CH_2 x 2), 30.75 (alkyl CH_2), 30.7 (alkyl CH_2), 30.6 (alkyl CH_2 x 2), 30.5 (alkyl CH_2), 30.4 (alkyl CH_2), 27.2 (C28), 27.15 (C3), 26.9 (C12), 16.0 (C43). HRMS (ESI) m/z: $[M+H]^+$ calculated for $C_{49}H_{66}N_7O_6S$: 880.4795, found 880.4762.

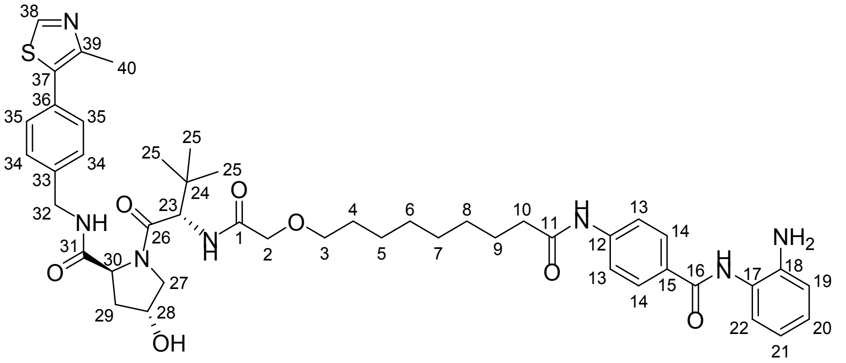

(2S,4R)-1-((S)-2-(2-((9-((4-((2-aminophenyl)carbamoyl)phenyl)amino)-9-oxononyl)oxy)acetamid o)-3,3-dimethylbutanoyl)-4-hydroxy-N-(4-(4-methylthiazol-5-yl)benzyl)pyrrolidine-2-carboxamide (6): Following general method H, Boc deprotection of **54f** (66.9 mg, 0.070 mmol) was performed to afford **6** (52.5 mg, 0.060 mmol, 86% yield) as a pale yellow solid. Prior to biological evaluation the PROTAC was further purified by semi-preparative HPLC (5-95% MeCN in H_2O, 260 nm, 45 min gradient). ^{1}H NMR (400 MHz, CD_3OD) δ_H ppm 8.86 (s, 1 H, 38-CH), 7.95 (d, *J*=8.7 Hz, 2 H, 14-CH), 7.72 (d, *J*=8.7 Hz, 2 H, 13-CH), 7.44 - 7.47 (m, 2 H, 35-CH), 7.39 - 7.43 (m, 2 H, 34-CH), 7.18 (dd, *J*=7.7, 1.1 Hz, 1 H, 22-CH), 7.07 (app. td, *J*=7.7, 1.1 Hz, 1 H, 20-CH), 6.90 (dd, *J*=7.7, 1.1 Hz, 1 H, 19-CH), 6.77 (app. td, *J*=7.7, 1.1 Hz, 1 H, 21-CH), 4.69 (s, 1 H, 23-CH), 4.56 - 4.63 (m, 1 H, 30-CH), 4.47 - 4.55 (m, 2 H, 28,32-CH), 4.36 (d, *J*=15.5 Hz, 1 H, 32-CH), 3.96 – 4.00 (m, 1 H, 2-CH), 3.91 - 3.95 (m, 1 H, 2-CH), 3.85 - 3.90 (m, 1 H, 27-CH), 3.76 - 3.82 (m, 1 H, 27-CH), 3.55 (t, *J*=6.3 Hz, 2 H, 3-CH_2), 2.47 (s, 3 H, 40-CH_3), 2.38 (t, *J*=7.5 Hz, 2 H, 10-CH_2), 2.20 - 2.26 (m, 1 H, 29-CH), 2.04 - 2.12 (m, 1 H, 29-CH), 1.62 - 1.72 (m, 4 H, 4-CH_2,9-CH_2), 1.36 - 1.46 (m, 8 H, (5-8)-CH_2), 1.03 (s, 9 H, 25-CH_3). ^{13}C NMR (101 MHz, CD_3OD) δ_C ppm 175.0 (C11), 174.4 (C31), 172.1 (C1), 171.8 (C26), 168.3 (C16), 153.0 (C38), 149.1 (C39), 143.9 (C18), 143.6 (C12), 140.3 (C33), 133.6 (C37), 131.6 (C36), 130.6 (C15), 130.5 (C34), 129.9 (C14), 129.1 (C35), 128.6 (C20), 127.8 (C22), 125.5 (C17),

120.4 (C13), 119.8 (C21), 118.8 (C19), 73.1 (C3), 71.2 (C28), 70.8 (C2), 61.0 (C30), 58.3 (C27), 58.1 (C23), 43.9 (C32), 39.1 (C29), 38.2 (C10), 37.4 (C24), 30.7 (C4), 30.5 (2 x alkyl CH_2), 30.3 (alkyl CH_2), 27.3 (C5), 27.1 (C24), 26.9 (C9), 16.0 (C40). HRMS (ESI) m/z: $[M+H]^+$ calculated for $C_{46}H_{60}N_7O_7S$: 854.4275, found 854.4277.

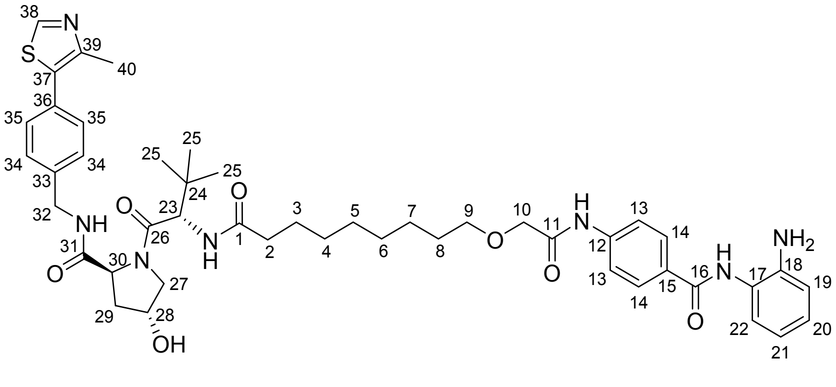

(2S,4R)-1-((S)-2-(9-(2-((4-((2-aminophenyl)carbamoyl)phenyl)amino)-2-oxoethoxy)nonanamido) -3,3-dimethylbutanoyl)-4-hydroxy-N-(4-(4-methylthiazol-5-yl)benzyl)pyrrolidine-2-carboxamide (7): Following general method H, Boc deprotection of **54g** (60.2 mg, 0.063 mmol) was performed to afford **7** (50.6 mg, 0.059 mmol, 93% yield) as a pale yellow solid. Prior to biological evaluation the PROTAC was further purified by semi-preparative HPLC (5-95% MeCN in H_2O, 260 nm, 45 min gradient). ^{1}H NMR (400 MHz, CD_3OD) δ_H ppm 8.87 (s, 1 H, 38-CH), 8.64 (t, *J*=5.9 Hz, 1 H, 31-NH),7.98 (d, *J*=8.7 Hz, 2 H, 14-CH), 7.75 - 7.83 (m, 3H, 13-CH, 1-NH), 7.46 (d, *J*=8.3 Hz, 2 H, 35-CH), 7.41 (d, *J*=8.3 Hz, 2 H, 34-CH), 7.18 (dd, *J*=7.7, 1.3 Hz, 1 H, 22-CH), 7.08 (app. td, *J*=7.7, 1.3 Hz, 1 H, 20-CH), 6.91 (dd, *J*=7.7, 1.3 Hz, 1 H, 19-CH), 6.77 (app. td, *J*=7.7, 1.3 Hz, 1 H, 21-CH), 4.62 - 4.65 (m, 1 H, 23-CH), 4.55 - 4.60 (m, 1 H, 30-CH), 4.47 - 4.54 (m, 2 H, 28-CH, 32-CH), 4.32 - 4.38 (m, 1 H, 32-CH), 4.10 (s, 2 H, 10-CH_2), 3.87 - 3.92 (m, 1 H, 27-CH), 3.77 - 3.82 (m, 1 H, 27-CH), 3.60 (t, *J*=6.6 Hz, 2 H, 9-CH_2), 2.47 (s, 3 H, 40-CH_3), 2.19 - 2.32 (m, 3 H, 2-CH_2,29-CH), 2.04 - 2.11 (m, 1 H, 29-CH), 1.66 - 1.72 (m, 2 H, 8-CH_2), 1.58 - 1.65 (m, 2 H, 3-CH_2), 1.33 - 1.44 (m, 8 H, (4-7)-CH_2), 1.03 (s, 9 H, 25-CH_3). ^{13}C NMR (101 MHz, CD_3OD) δ_C ppm δ ppm 176.1 (C1), 174.6 (C31), 172.5 (C25), 171.4 (C11), 168.3 (C16), 153.0 (C38), 149.2 (C39), 143.9 (C18), 142.5 (C12), 140.4 (C33), 133.5 (C37), 131.6 (C36), 131.1 (C15), 130.5 (C34), 130.0 (C14), 129.1 (C35), 128.6 (C20), 127.8 (C22), 125.5 (C17), 120.9 (C13), 119.8 (C21), 118.9 (C19), 73.1 (C9), 71.5 (C10), 71.2 (C28), 61.0 (C30), 59.1 (C23), 58.2 (C27), 43.8 (C32), 39.1 (C29), 36.8 (C24), 36.7 (C2), 30.6 (C8), 30.5 (alkyl CH_2), 30.45 (alkyl CH_2), 30.4 (alkyl CH_2), 27.2 (C25,C7), 27.1 (C3), 16.0 (C40). HRMS (ESI) m/z: $[M+H]^+$ calculated for $C_{46}H_{60}N_7O_7S$: 854.4275, found 854.4268.

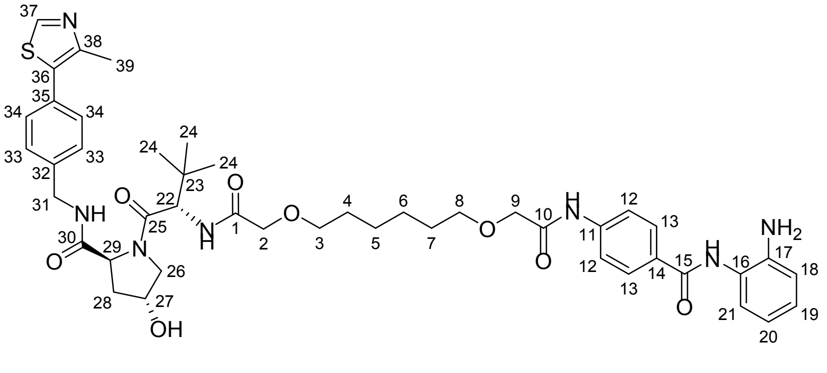

(2S,4R)-1-((S)-2-(2-((6-(2-((4-((2-aminophenyl)carbamoyl)phenyl)amino)-2-oxoethoxy)hexyl)oxy)acetamido)-3,3-dimethylbutanoyl)-4-hydroxy-N-(4-(4-methylthiazol-5-yl)benzyl)pyrrolidine -2-carboxamide (8): Following general method H, Boc deprotection of **54h** (86.3 mg, 0.090 mmol) was performed to afford **8** (71.7 mg, 0.083 mmol, 90% yield) as a pale yellow solid. Prior to biological evaluation the PROTAC was further purified by semi-preparative HPLC (5-95% MeCN in H_2O, 260 nm, 45 min gradient). ^{1}H NMR (400 MHz, CD_3OD) δ_H ppm 8.86 (s, 1 H, 37-CH), 7.97 (d, *J*=8.6 Hz, 2 H, 13-CH), 7.76 (d, *J*=8.6 Hz, 2 H, 12-CH), 7.43 - 7.47 (m, 2 H, 34-CH), 7.38 - 7.43 (m, 2 H, 33-CH), 7.18 (dd, *J*=7.7, 1.1 Hz, 1 H, 21-CH), 7.07 (td, *J*=7.7, 1.1 Hz, 1 H, 19-CH), 6.90 (dd, *J*=7.7, 1.1 Hz, 1 H, 18-CH), 6.77 (td, *J*=7.7, 1.1 Hz, 1 H, 20-CH), 4.69 (s, 1 H, 22-CH), 4.56 - 4.61 (m, 1 H, 29-CH), 4.53 (d, *J*=15.5 Hz, 1 H, 31-CH), 4.48 - 4.51 (m, 1 H, 27-CH), 4.35 (d, *J*=15.5 Hz, 1 H, 31-CH), 4.06 - 4.12 (m, 2 H, 9-CH_2), 3.99 - 4.01 (m, 1 H, 2-CH), 3.90 - 3.96 (m, 1 H, 2-CH), 3.84 - 3.90 (m, 1 H, 26-CH), 3.77 - 3.83 (m, 1 H, 26-CH), 3.55 - 3.62 (m, 4 H, 3-CH_2,8-CH_2), 2.47 (s, 3 H, 39-CH_3), 2.19 - 2.27 (m, 1 H, 28-CH), 2.05 - 2.12 (m, 1 H, 28-CH), 1.65 - 1.75 (m, 4 H, 4-CH_2,7-CH_2), 1.45 - 1.54 (m, 4 H, 5-CH_2,6-CH_2), 1.03 (s, 9 H, 24-CH_3). ^{13}C NMR (101 MHz, CD_3OD) δ_C ppm 174.5 (C30), 172.1 (C1), 171.8 (C25), 171.4 (C10), 168.2 (C15), 153.0 (C37), 149.2 (C38), 144.0 (C17), 142.5 (C11), 140.3 (C32), 133.5 (C36), 131.6 (C35), 131.1 (C14), 130.5 (C33), 130.0 (C13), 129.1 (C34), 128.6 (C19), 127.8 (C21), 125.5 (C16), 120.9 (C12), 119.8 (C20), 118.8 (C18), 73.05 (C3/8), 73.0 (C3/8), 71.5 (C9), 71.2 (C27), 70.9 (C2), 61.0 (C29), 58.3 (C26), 58.1 (C22), 43.9 (C31), 39.1 (C28), 37.4 (C23), 30.7 (C4/7), 30.5 (C4/7), 27.2 (C5/6), 27.1 (C24,C5/6), 16.0 (C39). HRMS (ESI) m/z: $[M+H]^+$ calculated for $C_{45}H_{58}N_7O_8S$: 856.4068, found 856.4064.

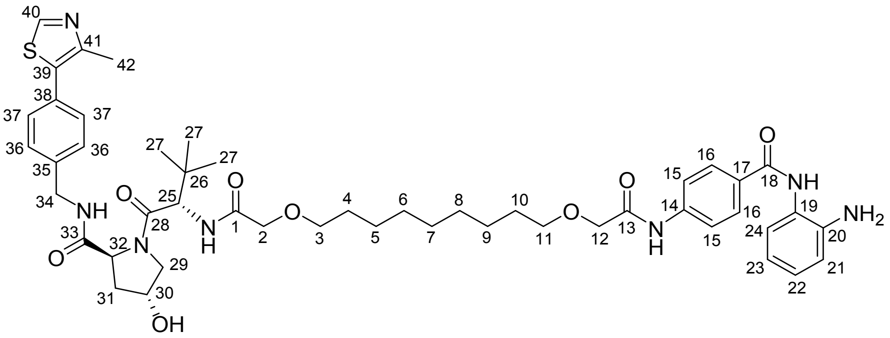

(2S,4R)-1-((S)-2-(2-((9-(2-((4-((2-aminophenyl)carbamoyl)phenyl)amino)-2-oxoethoxy)nonyl)oxy)acetamido)-3,3-dimethylbutanoyl)-4-hydroxy-N-(4-(4-methylthiazol-5-yl)benzyl)pyrrolidine-2-carboxamide (9): Following general method H, Boc deprotection of **54i** (66.7 mg, 0.067 mmol) was performed to afford **9** (58.2 mg, 0.064 mmol, 96% yield) as a pale yellow solid. Prior to biological evaluation the PROTAC was further purified by semi-preparative HPLC (5-95% MeCN in H_2O, 260 nm, 45 min gradient). ^{1}H NMR (400 MHz, CD_3OD) δ_H ppm 8.87 (s, 1 H, 40-CH), 7.98 (d, *J*=8.6 Hz, 2 H, 16-CH), 7.77 (d, *J*=8.6 Hz, 2 H, 15-CH), 7.46 (d, *J*=8.3 Hz, 2 H, 37-CH), 7.41 (d, *J*=8.3 Hz, 2 H, 36-CH), 7.18 (dd, *J*=7.7, 1.1 Hz, 1 H, 24-CH), 7.07 (app. td, *J*=7.7, 1.1 Hz, 1 H, 22-CH), 6.90 (dd, *J*=7.7, 1.1 Hz, 1 H, 21-CH), 6.77 (app. td, *J*=7.7, 1.1 Hz, 1 H, 23-CH), 4.69 (s, 1 H, 25-CH), 4.56 - 4.61 (m, 1 H, 32-CH), 4.54 (d, *J*=15.5 Hz, 1 H, 34-CH), 4.48 - 4.51 (m, 1 H, 30-CH), 4.35 (d, *J*=15.5 Hz, 1 H, 34-CH), 4.09 (s, 2 H, 12-CH_2), 3.96 - 4.01 (m, 1 H, 2-CH), 3.91 - 3.96 (m, 1 H, 2-CH), 3.84 - 3.90 (m, 1 H, 29-CH), 3.76 - 3.82 (m, 1 H, 29-CH), 3.52 - 3.60 (m, 4 H, 3-CH_2,11-CH_2), 2.47 (s, 3 H, 42-CH_3), 2.19 - 2.26 (m, 1 H, 31-CH), 2.04 - 2.11 (m, 1 H, 31-

CH), 1.61 - 1.70 (m, 4 H, 4-CH_2,10-CH_2), 1.33 - 1.44 (m, 10 H, (5-9)-CH_2), 1.03 (s, 9 H, 27-CH_3). ^{13}C NMR (101 MHz, CD_3OD) δ_C ppm 174.4 (C33), 172.2 (C1), 171.8 (C28), 171.4 (C13), 168.2 (C18), 153.0 (C40), 149.2 (C41), 144.0 (C20), 142.5 (C14), 140.4 (C35), 133.6 (C39), 131.6 (C38), 131.1 (C17), 130.5 (C36), 130.0 (C16), 129.1 (C37), 128.7 (C22), 127.8 (C24), 125.5 (C19), 120.9 (C15), 119.8 (C23), 118.9 (C21), 73.2 (C11), 73.1 (C3), 71.5 (C12), 71.2 (C30), 70.9 (C2), 61.0 (C32), 58.3 (C29), 58.1 (C25), 43.9 (C34), 39.1 (C31), 37.4 (C26), 30.8 (alkyl CH_2), 30.75 (alkyl CH_2), 30.65 (alkyl CH_2), 30.6 (alkyl CH_2), 30.55 (alkyl CH_2), 27.4 (C5/9), 27.3 (C5/9), 27.1 (C27), 16.0 (C42). HRMS (ESI) m/z: $[M+H]^+$ calculated for $C_{48}H_{64}N_7O_8S$: 898.4537, found 898.4531.

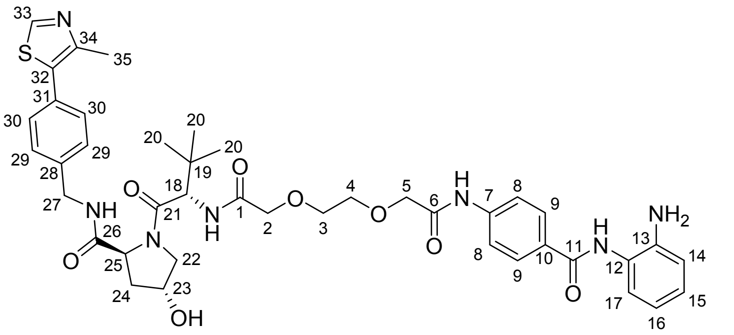

(2S,4R)-1-((S)-2-(2-(2-(2-((4-((2-aminophenyl)carbamoyl)phenyl)amino)-2-oxoethoxy)ethoxy) acetamido)-3,3-dimethylbutanoyl)-4-hydroxy-N-(4-(4-methylthiazol-5-yl)benzyl)pyrrolidine-2-carboxamide (10): Following general method H, Boc deprotection of **54j** (74.6 mg, 0.083 mmol) was performed to afford **10** (60.8, 0.074 mmol, 90% yield) as a white solid. Prior to biological evaluation the PROTAC was further purified by semi-preparative HPLC (5-95% MeCN in H_2O, 260 nm, 45 min gradient). ^1H NMR (400 MHz, CD_3OD) δ_H ppm 8.85 (s, 1 H, 33-CH), 8.58 (t, *J*=6.0 Hz, 1 H, 26-NH), 7.95 (d, *J*=8.7 Hz, 2H, 9-CH), 7.75 - 7.85 (m, 3 H, 8-CH,1-NH), 7.40 - 7.45 (m, 2 H, 30-CH), 7.36 - 7.40 (m, 2 H, 29-CH), 7.18 (dd, *J*=7.7, 1.3 Hz, 1 H, 17-CH), 7.08 (app. td, *J*=7.7, 1.3 Hz, 1 H, 15-CH), 6.91 (dd, *J*=7.7, 1.3 Hz, 1 H, 14-CH), 6.77 (app. td, *J*=7.7, 1.3 Hz, 1 H, 15-CH), 4.74 (d, *J*=9.4 Hz, 1 H, 18-CH), 4.55 - 4.61 (m, 1 H, 25-CH), 4.49 - 4.52 (m, 1 H, 23-CH), 4.43 - 4.49 (m, 1 H, 27-CH), 4.30 - 4.36 (m, 1 H, 27-CH), 4.22 (s, 2 H, 5-CH_2), 4.14 - 4.19 (m, 1 H, 2-CH), 4.05 - 4.11 (m, 1 H, 2-CH), 3.86 - 3.91 (m, 1 H, 22-CH), 3.2 - 3.86 (m, 4 H, 3-CH_2,4-CH_2), 3.77 - 3.81 (m, 1 H, 22-CH), 2.45 (s, 3 H, 35-CH_3), 2.19 - 2.26 (m, 1 H, 24-CH), 2.05 - 2.12 (m, 1 H, 24-CH), 1.04 (s, 9 H, 20-CH_3). ^{13}C NMR (101 MHz, CD_3OD) δ_C ppm 174.4 (C26), 172.1 (C1), 171.9 (C21), 171.2 (C6), 168.2 (C11), 152.9 (C33), 149.1 (C34), 144.0 (C13), 142.5 (C7), 140.3 (C28), 133.5 (C32), 131.6 (C31), 131.1 (C10), 130.5 (C29), 129.9 (C9), 129.0 (C32), 128.7 (C15), 127.8 (C17), 125.5 (C12), 121.0 (C8), 119.8 (C16), 118.8 (C14), 72.3 (C3/4), 72.1 (C3/4), 71.9 (C5), 71.2 (C23), 71.2 (C2), 61.0 (C25), 58.4 (C18), 58.3 (C22), 43.8 (C27), 39.1 (C24), 37.2 (C19), 27.1 (C20), 16.0 (C35). HRMS (ESI) m/z: $[M+H]^+$ calculated for $C_{41}H_{50}N_7O_8S$: 800.3442, found 800.3444.

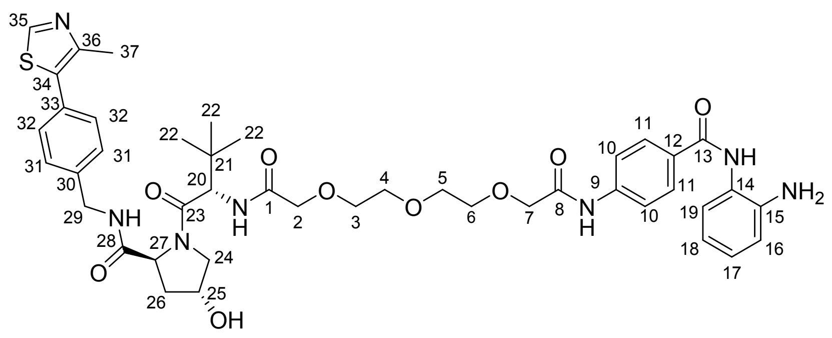

(2S,4R)-1-((S)-14-((4-((2-aminophenyl)carbamoyl)phenyl)amino)-2-(tert-butyl)-4,14-dioxo-6,9,12-trioxa-3-azatetradecanoyl)-4-hydroxy-N-(4-(4-methylthiazol-5-yl)benzyl)pyrrolidine-2-carboxamide (11): Following general method H, Boc deprotection of **54k** (45.3 mg, 0.048 mmol) was performed to afford **11** (37.1 mg, 0.044 mmol, 91% yield) as a white solid. Prior to biological evaluation the PROTAC was further purified by semi-preparative HPLC (5-95% MeCN in H_2O, 260 nm, 45 min gradient). ^1H NMR (400 MHz, CD_3OD) δ_H ppm 8.86 (s, 1 H, 35-CH), 8.62 (t, *J*=6.1 Hz, 1 H, 28-NH), 7.97 (d, *J*=8.7 Hz, 2 H, 11-CH), 7.75 (d, *J*=8.7 Hz, 2 H, 10-CH), 7.64 (d, *J*=9.4 Hz, 1 H, 1-NH), 7.43 - 7.46 (m, 2 H, 32-CH), 7.38 - 7.42 (m, 2 H, 31-CH), 7.19 (dd, *J*=7.8, 1.3 Hz, 1 H, 19-CH), 7.09 (app. td, *J*=7.8, 1.3 Hz, 1 H, 17-CH), 6.92 (dd, *J*=7.8, 1.3 Hz, 1 H, 16-CH), 6.79 (app. td, *J*=7.8, 1.3 Hz, 1 H, 18-CH), 4.69 (d, *J*=9.6 Hz, 1 H, 20-CH), 4.55 - 4.59 (m, 1 H, 27-CH), 4.51 - 4.55 (m, 1 H, 29-CH), 4.47 - 4.51 (m, 1 H, 25-CH), 4.29 - 4.36 (m, 1 H, 29-CH), 4.14 - 4.18 (m, 1 H, 7-CH), 4.08 - 4.14 (m, 1 H, 7-CH), 4.01 - 4.05 (m, 1 H, 2-CH), 3.89 - 3.95 (m, 1 H, 2-CH), 3.84 - 3.87 (m, 1 H, 24-CH), 3.72 - 3.83 (m, 9 H, (3-6)-CH_2,24-CH), 2.47 (s, 3 H, 37-CH_3), 2.16 - 2.25 (m, 1 H, 26-CH), 2.04 - 2.12 (m, 1 H, 26-CH), 1.02 (s, 9 H, 22-CH_3). ^{13}C NMR (101 MHz, CD_3OD) δ_C ppm 174.5 (C28), 172.0 (C1), 171.7 (C23), 171.6 (C8), 168.3 (C13), 153.0 (C35), 149.2 (C36), 144.0 (C15), 142.4 (C9), 140.4 (C30), 133.5 (C34), 131.7 (C33), 131.3 (C12), 130.5 (C31), 130.0 (C11), 129.2 (C32), 128.7 (C17), 127.9 (C19), 125.5 (C14), 121.2 (C10), 119.8 (C18), 118.8 (C16), 72.3 (alkoxy CH_2), 72.2 (alkoxy CH_2), 71.7 (C7), 71.6 (alkoxy CH_2), 71.5 (alkoxy CH_2), 71.2 (C25), 71.1 (C2), 61.0 (C27), 58.3 (C20), 58.2 (C24), 43.9 (C29), 39.1 (C26), 37.4 (C21), 27.1 (C22), 16.0 (C37). HRMS (ESI) m/z: $[M+H]^+$ calculated for $C_{43}H_{54}N_7O_9S$: 844.3704, found 844.3702.

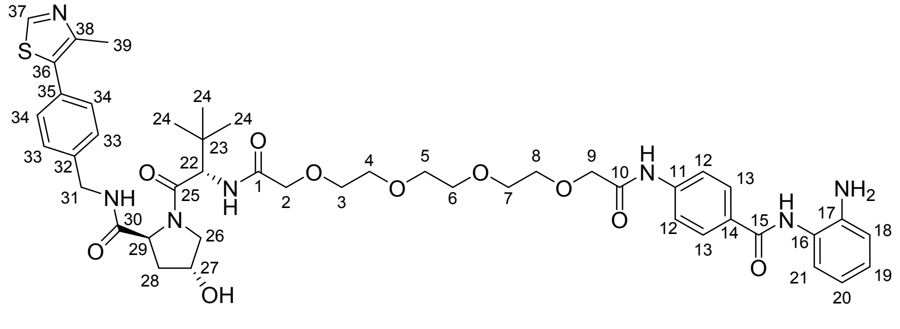

N1-(4-((2-aminophenyl)carbamoyl)phenyl)-N14-((S)-1-((2S,4S)-4-hydroxy-2-((4-(4-methylthiazol-5-yl)benzyl)carbamoyl)pyrrolidin-1-yl)-3,3-dimethyl-1-oxobutan-2-yl)-3,6,9,12-tetraoxatetradecanediamide (12): Following general method H, Boc deprotection of **54l** (15.7 mg, 0.02 mmol) was performed to afford **12** (14.1 mg, 0.02 mmol, 100 %) as a white solid. Prior to biological evaluation the PROTAC was further purified by semi-preparative HPLC (5-95% MeCN in H_2O, 260 nm, 45 min gradient). ^1H NMR (400 MHz, CD_3OD) δ_H ppm 8.86 (s, 1 H, 37-CH), 7.98 (d, *J*=8.7 Hz, 2 H, 13-CH), 7.79

(d, *J*=8.7 Hz, 2 H, 12-CH), 7.43 - 7.46 (m, 2 H, 34-CH), 7.39 - 7.42 (m, 2 H, 33-CH), 7.18 (dd, *J*=7.7, 1.4 Hz, 1 H, 21-CH), 7.08 (app. td, *J*=7.7, 1.4 Hz, 1 H, 19-CH), 6.90 (dd, *J*=7.7, 1.4 Hz, 1 H, 18-CH), 6.77 (app. td, *J*=7.7, 1.4 Hz, 1 H, 20-CH), 4.68 (s, 1 H, 22-CH), 4.56 - 4.58 (m, 1 H, 29-CH), 4.50 - 4.54 (m, 1 H, 31-CH), 4.47 - 4.50 (m, 1 H, 27-CH), 4.33 (d, *J*=15.7 Hz, 1 H, 31-CH), 4.14 (s, 2 H, 9-CH_2), 4.01 - 4.05 (m, 1 H, 2-CH), 3.94 - 3.99 (m, 1 H, 2-CH), 3.84 - 3.88 (m, 1 H, 26-CH), 3.76 - 3.80 (m, 1 H, 26-CH), 3.66 - 3.75 (m, 12 H, (3-8)-CH_2), 2.47 (s, 3 H, 39-CH_3), 2.18 - 2.23 (m, 1 H, 28-CH), 2.04 - 2.10 (m, 1 H, 28-CH), 1.03 (s, 9 H, 24-CH_3). ^{13}C NMR (101 MHz, CD_3OD) δ_C ppm 174.5 (C30), 172.1 (C1), 171.8 (C25), 171.5 (C10), 168.3 (C15), 153.0 (C37), 149.2 (C38), 144.0 (C17), 142.5 (C11), 140.4 (C32), 133.5 (C36), 131.6 (C35), 131.2 (C14), 130.5 (C33), 130.0 (C13), 129.1 (C34), 128.7 (C19), 127.9 (C21), 125.5 (C16), 121.0 (C12), 119.8 (C20), 118.8 (C18), 72.4 (alkoxy CH_2), 72.3 (alkoxy CH_2), 71.8 (alkoxy CH_2), 71.7 (alkoxy CH_2), 71.65 (alkoxy CH_2), 71.6 (alkoxy CH_2), 71.4 (alkoxy CH_2), 71.2 (C2/27), 71.2 (C2/27), 60.9 (C29), 58.25 (C22/26), 58.2 (C22/26), 43.8 (C31), 39.1 (C28), 37.3 (C23), 27.1 (C24), 16.0 (C39). HRMS (ESI) m/z: $[M+H]^+$ calculated for $C_{45}H_{58}N_7O_{10}S$: 888.3966, found 888.3962.

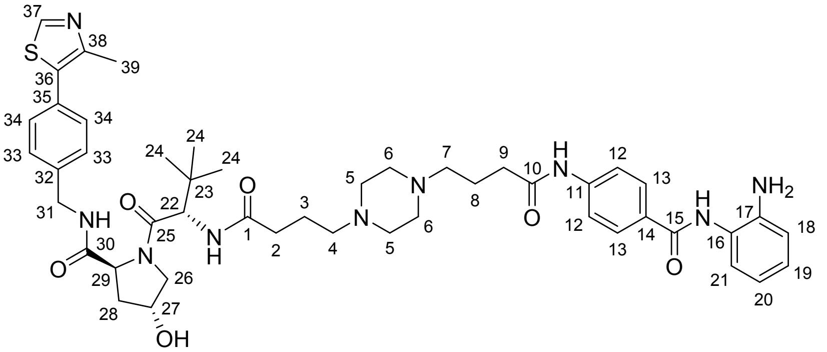

(2S,4R)-1-((S)-2-(4-(4-(4-((4-((2-aminophenyl)carbamoyl)phenyl)amino)-4-oxobutyl)piperazin-1-yl)butanamido)-3,3-dimethylbutanoyl)-4-hydroxy-N-(4-(4-methylthiazol-5-yl)benzyl)pyrrolidine-2-carboxamide (13): Following general method H, Boc deprotection of **54m** (20.2 mg, 0.021 mmol) was performed to afford **13** (17.6 mg, 0.020 mmol, 96% yield) as a pale yellow solid. Prior to biological evaluation the PROTAC was further purified by column chromatography (alumina (basic), 1-10% MeOH in DCM). ^{1}H NMR (400 MHz, CD_3OD) δ_H 8.87 (s, 1 H, 37-CH), 7.96 (d, *J*=8.7 Hz, 2 H, 13-CH), 7.73 (d, *J*=8.7 Hz, 2 H, 12-CH), 7.45 - 7.50 (m, 2 H, 34-CH), 7.38 - 7.44 (m, 2 H, 33-CH), 7.18 (dd, *J*=7.8, 1.3 Hz, 1 H, 21-CH), 7.07 (app. td, *J*=7.8, 1.3 Hz, 1 H, 19-CH), 6.91 (dd, *J*=7.8, 1.3 Hz, 1 H, 18-CH), 6.77 (app. td, *J*=7.8, 1.3 Hz, 1 H, 20-CH), 4.62 (s, 1 H, 22-CH), 4.52 - 4.58 (m, 2 H, 29-CH,31-CH), 4.46 - 4.51 (m, 1 H, 27-CH), 4.35 (d, *J*=15.6 Hz, 1 H, 31-CH), 3.86 - 3.92 (m, 1 H, 26-CH), 3.76 - 3.83 (m, 1 H, 26-CH), 2.39 - 2.63 (m, 15 H, (5,6,7,9)-CH_2), 2.32 - 2.37 (m, 2 H, 4-CH_2), 2.29 (t, *J*=7.5 Hz, 2 H, 2-CH_2), 2.18 - 2.24 (m, 1 H, 28-CH), 2.04 - 2.11 (m, 1 H, 28-CH), 1.87 - 1.94 (m, 2 H, 8-CH_2), 1.74 - 1.83 (m, 2 H, 3-CH_2), 1.03 (s, 9 H, 24-CH_3). ^{13}C NMR (101 MHz, CD_3OD) δ_C ppm 175.6 (C1), 174.6 (C10), 174.5 (C30), 172.4 (C25), 168.4 (C15), 153.0 (C37), 149.2 (C38), 143.9 (C17), 143.7 (C11), 140.4 (C32), 133.6 (C36), 131.6 (C35), 130.6 (C14), 130.5 (C35), 129.9 (C13), 129.1 (C34), 128.6 (C19), 127.8 (C21), 125.6 (C16), 120.4 (C12), 119.8 (C20), 118.9 (C18), 71.2 (C27), 61.0 (C29), 59.2 (C22), 59.0 (C7), 58.7 (C4), 58.2 (C26), 53.9 (C5/6), 53.85 (C5/6), 43.8 (C31), 39.1 (C28), 36.7 (C23), 36.2 (C9), 34.5 (C2), 27.2 (C24), 23.8 (C3), 23.6 (C8), 16.0 (C39). HRMS (ESI) m/z: $[M+H]^+$ calculated for $C_{47}H_{62}N_9O_6S$: 880.4544, found 880.4548.

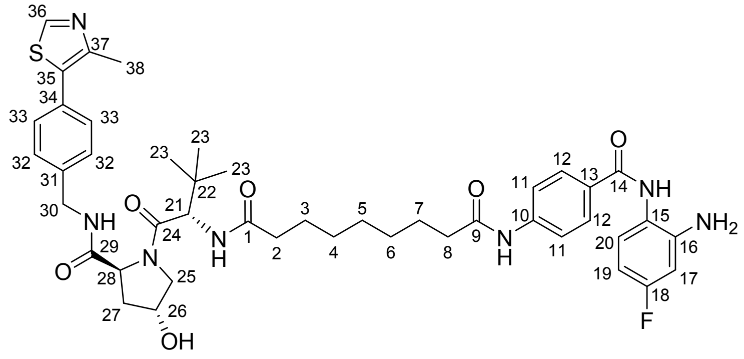

N1-(4-((2-amino-4-fluorophenyl)carbamoyl)phenyl)-N9-((S)-1-((2S,4R)-4-hydroxy-2-((4-(4-methylthiazol-5-yl)benzyl)carbamoyl)pyrrolidin-1-yl)-3,3-dimethyl-1-oxobutan-2-yl)nonanediamide (14): Following general method H, Boc deprotection of **54n** (61.3 mg, 0.066 mmol) was performed to afford **14** (56.1 mg, 0.066 mmol, 100% yield) as a pale yellow solid. ^{1}H NMR (400 MHz, CD_3OD) δ_H ppm δ ppm 8.86 (s, 1 H, 36-CH), 7.94 (d, J=8.7 Hz, 2 H, 12-CH), 7.71 (d, J=8.7 Hz, 2 H, 11-CH), 7.42 - 7.47 (m, 2 H, 33-CH), 7.37 - 7.42 (m, 2 H, 32-CH), 7.11 (dd, J_{HH}=8.6, J_{HF}=6.0 Hz, 1 H, 20-CH), 6.58 (dd, J_{HF}=10.7, J_{HH}=2.8 Hz, 1 H, 17-CH), 6.41 (app. td, J_{HF}=8.6, J_{HH}=8.6, 2.8 Hz, 1 H, 19-CH), 4.63 (s, 1 H, 21-CH), 4.55 - 4.60 (m, 1 H, 28-CH), 4.52 (d, J=15.5 Hz, 1 H, 30-CH), 4.46 - 4.50 (m, 1 H, 26-CH), 4.35 (d, J=15.5 Hz, 1 H, 30-CH), 3.87 - 3.94 (m, 1 H, 25-CH), 3.75 - 3.82 (m, 1 H, 25-CH), 2.46 (s, 3 H, 38-CH_3), 2.39 (t, J=7.5 Hz, 2 H, 8-CH_2), 2.18 - 2.32 (m, 3 H, 2-CH_2,27-CH), 2.03 - 2.12 (m, 1 H, 27-CH), 1.70 (quin, J=7.5 Hz, 2 H, 7-CH_2), 1.61 (quin, J=7.2 Hz, 2 H, 3-CH_2), 1.32 - 1.40 (m, 6 H, (4-6)-CH_2), 1.03 (s, 9 H, 23-CH_3). ^{13}C NMR (101 MHz, CD_3OD) δ_C ppm 176.2 (C1), 175.1 (C9), 174.6 (C29), 172.5 (C24), 168.7 (C14), 163.8 (d, J_{CF}=241.3 Hz, C18), 153.0 (C36), 149.1 (C37), 146.6 (d, J_{CF}=11.6 Hz, C16), 143.6 (C10), 140.4 (C31), 133.6 (C35), 131.6 (C34), 130.5 (C32), 130.4 (C13), 129.9 (C12), 129.7 (d, J_{CF}=10.5 Hz, C20), 129.1 (C33), 120.8 (d, J_{CF}=1.7 Hz, C15), 120.4 (C11), 105.2 (d, J_{CF}=23.1 Hz, C19), 104.1 (d, J_{CF}=25.7 Hz, C17), 71.2 (C28), 61.0 (C28), 59.1 (C21), 58.2 (C25), 43.8 (C30), 39.1 (C27), 38.2 (C8), 36.8 (C2), 36.7 (C25), 30.3 (C4/5/6), 30.2 (C4/5/6), 30.15 (C4/5/6), 27.2 (C23), 27.1 (C3), 26.8 (C7), 16.0 (C38). ^{19}F NMR (376 MHz, CD_3OD) δ_F ppm -117.5.HRMS (ESI) m/z: $[M+H]^+$ calculated for $C_{44}H_{55}FN_7O_6S$: 828.3919, found 828.3927.

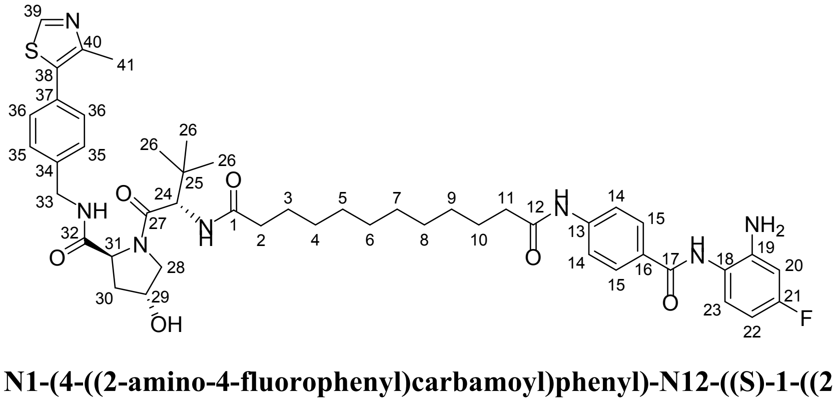

N1-(4-((2-amino-4-fluorophenyl)carbamoyl)phenyl)-N12-((S)-1-((2S,4R)-4-hydroxy-2-((4-(4-methylthiazol-5-yl)benzyl)carbamoyl)pyrrolidin-1-yl)-3,3-dimethyl-1-oxobutan-2-yl)dodecanediamide (15): Following general method H, Boc deprotection of **54o** (58.7 mg, 0.061 mmol) was performed to afford **15** (49.2 mg, 0.056 mmol, 92% yield) as a pale yellow solid. ^{1}H NMR (400 MHz, CD_3OD) δ_H ppm 8.86 (s, 1 H, 39-CH), 7.94 (d, J=8.7 Hz, 2 H, 15-CH), 7.72 (d, J=8.7 Hz, 2 H, 14-CH), 7.43 - 7.47 (m, 2 H, 36-CH), 7.37

- 7.42 (m, 2 H, 35-CH), 7.11 (dd, J_{HH}=8.6, J_{HF}=6.0 Hz, 1 H, 23-CH), 6.58 (dd, J_{HF}=10.7, J_{HH}=2.8 Hz, 1 H, 20-CH), 6.41 (app. td, J_{HF}=8.6, J_{HH} =8.6, 2.8 Hz, 1 H, 22-CH), 4.63 (s, 1 H, 24-CH), 4.55 - 4.60 (m, 1 H, 31-CH), 4.52 (d, J=15.5 Hz, 1 H, 33-CH), 4.47 - 4.50 (m, 1 H, 29-CH), 4.35 (d, J=15.5 Hz, 1 H, 33-CH), 3.86 - 3.93 (m, 1 H, 28-CH), 3.76 - 3.82 (m, 1 H, 28-CH), 2.46 (s, 3 H, 41-CH_3), 2.39 (t, J=7.5 Hz, 2 H, 11-CH_2), 2.17 - 2.31 (m, 3 H, 2-CH_2,30-CH), 2.03 - 2.12 (m, 1 H, 30-CH), 1.69 (quin, J=7.5 Hz, 2 H, 10-CH_2), 1.53 - 1.63 (m, 2 H, 3-CH_2), 1.29 - 1.37 (m, 12 H, (4-9)-CH_2), 1.03 (s, 9 H, 26-CH_3). ^{13}C NMR (101 MHz, CD_3OD) δ_C ppm 176.2 (C1), 175.1 (C12), 174.6 (C32), 172.5 (C27), 168.7 (C17), 163.8 (d, J_{CF}=241.3 Hz, C21), 153.0 (C39), 149.1 (C40), 146.6 (d, J_{CF}=11.4 Hz, C19), 143.7 (C13), 140.4 (C34), 133.5 (C38), 131.6 (C37), 130.5 (C35), 130.4 (C16), 129.9 (C15), 129.7 (d, J_{CF}=10.3 Hz, C23), 129.1 (C36), 120.8 (d, J_{CF}=2.3 Hz, C18), 120.4 (C14), 105.2 (d, J_{CF}=23.1 Hz, C22), 104.1 (d, J_{CF}=25.6 Hz, C20), 71.2 (C29), 61.0 (C31), 59.1 (C24), 58.2 (C28), 43.8 (C33), 39.1 (C30), 38.2 (C11), 36.8 (C2), 36.7 (C25), 30.65 (alkyl CH_2), 30.6 (alkyl CH_2), 30.5 (alkyl CH_2), 30.45 (alkyl CH_2), 30.4 (2x alkyl CH_2), 27.2 (C26), 27.1 (C3), 26.9 (C10), 16.0 (C41). ^{19}F NMR (376 MHz, CD_3OD) δ_F ppm -117.5. HRMS (ESI) m/z: $[M+H]^+$ calculated for $C_{47}H_{61}FN_7O_6S$: 870.4388, found 870.4376.

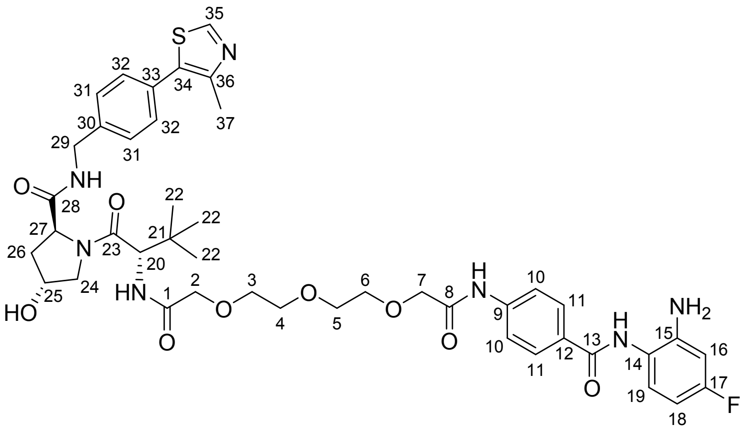

(2S,4R)-1-((S)-14-((4-((2-amino-4-fluorophenyl)carbamoyl)phenyl)amino)-2-(tert-butyl)-4,14-dioxo-6,9,12-trioxa-3-azatetradecanoyl)-4-hydroxy-N-(4-(4-methylthiazol-5-yl)benzyl)pyrrolidine-2-carboxamide (16): Following general method H, Boc deprotection of **54p** (65.8 mg, 0.068 mmol) was performed to afford **16** (58.9 mg, 0.068 mmol, 99% yield) as a pale yellow solid. ^{1}H NMR (400 MHz, CD_3OD) δ_H ppm 8.85 (s, 1 H, 35-CH), 7.95 (d, J=8.7 Hz, 2 H, 11-CH), 7.73 (d, J=8.7 Hz, 2 H, 10-CH), 7.41 - 7.45 (m, 2 H, 32-CH), 7.37 - 7.41 (m, 2 H, 31-CH), 7.11 (dd, J_{HH}=8.6, J_{HF}=6.1 Hz, 1 H, 19-CH), 6.58 (dd, J_{HF}=10.7, J_{HH}=2.8 Hz, 1 H, 16-CH), 6.41 (app. td, J_{HF}=8.6, J_{HH}=8.6, 2.8 Hz, 1 H, 18-CH), 4.68 (s, 1 H, 20-CH), 4.56 - 4.60 (m, 1 H, 27-CH), 4.53 (d, J=15.5 Hz, 1 H, 29-CH), 4.45 - 4.50 (m, 1 H, 25-CH), 4.32 (d, J=15.5 Hz, 1 H, 29-CH), 4.12 - 4.19 (m, 1 H, 7-CH), 4.05 - 4.12 (m, 1 H, 7-CH), 4.02 (d, J=15.7 Hz, 1 H, 2-CH), 3.91 (d, J=15.6 Hz, 1 H, 2-CH), 3.82 - 3.87 (m, 1 H, 24-CH), 3.70 - 3.81 (m, 9 H, 24-CH,(3-6)-CH_2), 2.46 (s, 3 H, 37-CH_3), 2.16 - 2.25 (m, 1 H, 26-CH), 2.03 - 2.12 (m, 1 H, 26-CH), 1.02 (s, 9 H, 22-CH_3). ^{13}C NMR (101 MHz, CD_3OD) δ_C ppm 174.5 (C28), 172.0 (C1), 171.7 (C23), 171.6 (C8), 168.6 (C13), 163.8 (d, J_{CF}=241.3 Hz, C17), 153.0 (C35), 149.2 (C36), 146.7 (d, J_{CF}=11.6 Hz, C15), 142.4 (C9), 140.4 (C30), 133.5 (C34), 131.7 (C33), 131.1 (C12), 130.5 (C31), 129.9 (C11), 129.8 (d, J_{CF}=10.5 Hz, C19), 129.1 (C32), 121.2 (C10), 120.7 (d, J_{CF}=2.1 Hz, C14), 105.1 (d, J_{CF}=23.1 Hz, C18), 104.0 (d, J_{CF}=25.6 Hz, C16), 72.3 (CH_2), 72.25 (CH_2), 71.7

(C7), 71.6 (CH_2), 71.55 (CH_2), 71.2 (C25), 71.1 (C2), 60.9 (C27), 58.3 (C20), 58.2 (C24), 43.9 (C29), 39.1 (C26), 37.3 (C21), 27.1 (C22), 16.0 (C37). ^{19}F NMR (376 MHz, CD_3OD) δ ppm -117.4. HRMS (ESI) m/z: $[M+H]^+$ calculated for $C_{43}H_{53}FN_7O_9S$: 862.3610, found 862.3609.

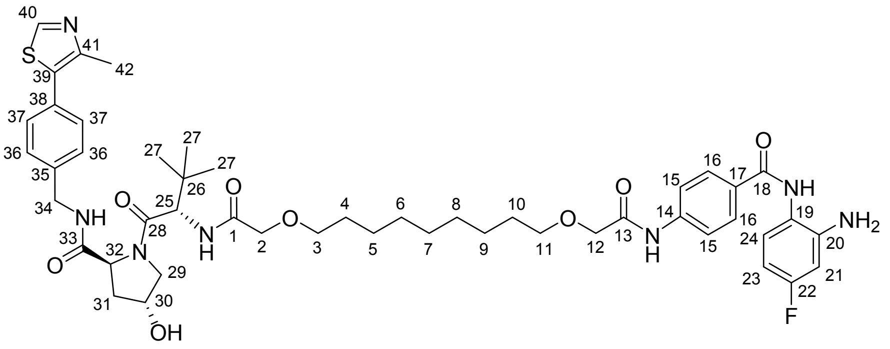

(2S,4R)-1-((S)-2-(2-((9-(2-((4-((2-amino-4-fluorophenyl)carbamoyl)phenyl)amino)-2-oxoethoxy)nonyl) oxy)acetamido)-3,3-dimethylbutanoyl)-4-hydroxy-N-(4-(4-methylthiazol-5-yl)benzyl)pyrrolidine-2-carboxamide (17): Following general method H, Boc deprotection of **54q** (70.8 mg, 0.070 mmol) was performed to afford **17** (63.3 mg, 0.068, 98% yield) as a pale brown solid. ^{1}H NMR (400 MHz, CD_3OD) δ_H ppm 8.86 (s, 1 H, 40-CH), 7.96 (d, J=8.7 Hz, 2 H, 16-CH), 7.75 (d, J=8.7 Hz, 2 H, 15-CH), 7.42 - 7.47 (m, 2 H, 37-CH), 7.37 - 7.42 (m, 2 H, 36-CH), 7.11 (dd, J_{HH}=8.6, J_{HF}=6.1 Hz, 1 H, 24-CH), 6.58 (dd, J_{HF}=10.7, J_{HH}=2.8 Hz, 1 H, 21-CH), 6.41 (td, J_{HF}=8.6, J_{HH}=8.6, 2.8 Hz, 1 H, 23-CH), 4.68 (s, 1 H, 25-CH), 4.59 (dd, J=9.0, 7.8 Hz, 1 H, 32-CH), 4.52 (d, J=15.6 Hz, 1 H, 34-CH), 4.47 - 4.50 (m, 1 H, 30-CH), 4.34 (d, J=15.6 Hz, 1 H, 34-CH), 4.07 (s, 2 H, 12-CH_2), 3.95 (d, J=15.4 Hz, 1 H, 2-CH_2), 3.94 (d, J=15.4 Hz, 1 H, 2-CH_2), 3.84 - 3.89 (m, 1 H, 29-CH), 3.74 - 3.82 (m, 1 H, 29-CH), 3.50 - 3.59 (m, 4 H, (3,11)-CH_2), 2.46 (s, 3 H, 42-CH_3), 2.18 - 2.27 (m, 1 H, 31-CH), 2.02 - 2.12 (m, 1 H, 31-CH), 1.58 - 1.68 (m, 4 H, (4,10)-CH_2), 1.32 - 1.43 (m, 10 H, (5-9)-CH_2), 1.03 (s, 9 H, 27-CH_3). ^{13}C NMR (101 MHz, CD_3OD) δ_C ppm 174.4 (C33), 172.1 (C1), 171.8 (C28), 171.3 (C13), 168.6 (C18), 163.8 (d, J_{CF}=241.7 Hz, C22), 153.0 (C40), 149.1 (C41), 146.6 (d, J_{CF}=11.6 Hz, C20), 142.6 (C14), 140.3 (C35), 133.6 (C39), 131.6 (C38), 131.0 (C17), 130.5 (C36), 129.9 (C16), 129.7 (d, J_{CF}=10.3 Hz, C24), 129.1 (C37), 120.9 (C15), 120.8 (d, J_{CF}=2.3 Hz, C19), 105.1 (d, J_{CF}=23.1 Hz, C23), 104.0 (d, J_{CF}=25.7 Hz, C21), 73.2 (C11), 73.1 (C3), 71.5 (C12), 71.2 (C30), 70.8 (C2), 61.0 (C32), 58.3 (C29), 58.1 (C25), 43.9 (C34), 39.1 (C31), 37.3 (C26), 30.8 (alkyl CH_2), 30.75 (alkyl CH_2), 30.65 (alkyl CH_2), 30.6 (alkyl CH_2), 30.55 (alkyl CH_2), 27.4 (5/9), 27.2 (5/9), 27.1 (C27), 16.0 (C42). ^{19}F NMR (376 MHz, CD_3OD) δ_F ppm -117.4. HRMS (ESI) m/z: $[M+H]^+$ calculated for $C_{48}H_{63}FN_7O_8S$: 916.4443, found 916.4426.

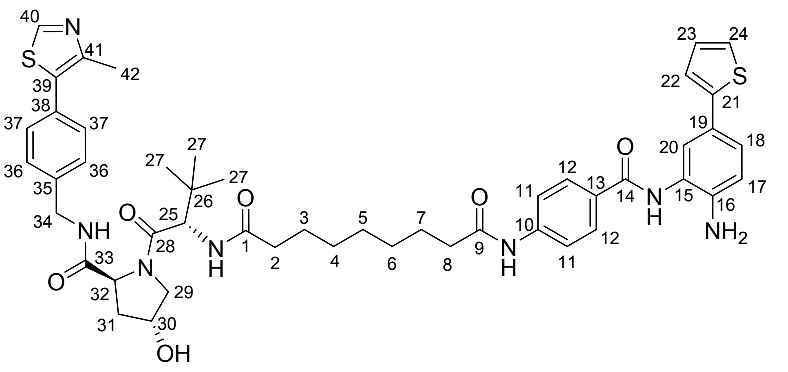

N1-(4-((2-amino-5-(thiophen-2-yl)phenyl)carbamoyl)phenyl)-N9-((S)-1-((2S,4R)-4-hydroxy-2-((4-(4-methylthiazol-5-yl)benzyl)carbamoyl)82yrrolidine-1-yl)-3,3-dimethyl-1-oxobutan-2-yl)nonanediamide (18): Following general method H, Boc deprotection of **54r** (46.3 mg, 0.047 mmol) was performed to afford **18** (36.0 mg, 0.040 mmol, 86% yield) as a pale yellow solid. ^{1}H NMR (400 MHz, CD_3OD) δ_H ppm 8.85 (s, 1 H, 40-CH), 7.97 (d, *J*=8.7 Hz, 2 H, 12-CH), 7.72 (d, *J*=8.7 Hz, 2 H, 11-CH), 7.49 (d, *J*=2.2 Hz, 1 H, 20-CH), 7.41 – 7.46 (m, 2 H, 37-CH), 7.37 – 7.41 (m, 2 H, 39-CH), 7.34 (dd, *J*=8.3, 2.2 Hz, 1 H, 18-CH), 7.22 (dd, *J*=5.1, 1.0 Hz, 1 H, 24-CH), 7.20 (dd, *J*=3.7, 1.0 Hz, 1 H, 22-CH), 7.01 (dd, *J*=5.1, 3.7 Hz, 1 H, 23-CH), 6.90 (d, *J*=8.3 Hz, 1 H, 17-CH), 4.63 (s, 1 H, 25-CH), 4.55 – 4.61 (m, 1 H, 32-CH), 4.51 (d, *J*=15.4 Hz, 1 H, 34-CH), 4.46 – 4.49 (m, 1 H, 30-CH), 4.34 (d, *J*=15.4 Hz, 1 H, 34-CH), 3.86 – 3.93 (m, 1 H, 29-CH), 3.74 – 3.82 (m, 1 H, 29-CH), 2.45 (s, 3 H, 42-CH_3), 2.39 (t, *J*=7.5 Hz, 2 H, 8-CH_2), 2.16 – 2.31 (m, 3 H, 2-CH_2,31-CH), 2.02 – 2.11 (m, 1 H, 31-CH), 1.70 (quin, *J*=7.5 Hz, 2 H, 7-CH_2), 1.60 (quin, *J*=7.0 Hz, 2 H, 3-CH_2), 1.31 – 1.40 (m, 6 H, (4-6)-CH_2), 1.03 (s, 9 H, 27-CH_3). ^{13}C NMR (101 MHz, CD_3OD) δ_C ppm 176.2 (C1), 175.1 (C9), 174.6 (C33), 172.5 (C28), 168.4 (C14), 153.0 (C40), 149.1 (C41), 145.8 (C21), 143.75 (C16), 143.7 (C10), 140.4 (C35), 133.6 (C39), 131.6 (C38), 130.5 (C36), 130.4 (C13), 130.0 (C12), 129.1 (C37), 129.0 (C23), 126.6 (C15), 126.2 (C18), 125.5 (C19), 125.3 (C20), 124.3 (C24), 122.7 (C22), 120.5 (C11), 118.9 (C17), 71.2 (C30), 61.0 (C32), 59.1 (C25), 58.2 (C29), 43.8 (C34), 39.1 (C31), 38.2 (C8), 36.75 (C2), 36.7 (C26), 30.3 (C4/5/6), 30.25 (C4/5/6), 30.2 (C4/5/6), 27.2 (C27), 27.1 (C3), 26.8 (C7), 16.0 (C42). HRMS (ESI) m/z: $[M+H]^+$ calculated for $C_{48}H_{58}N_7O_6S_2$: 892.3890, found 892.3889.

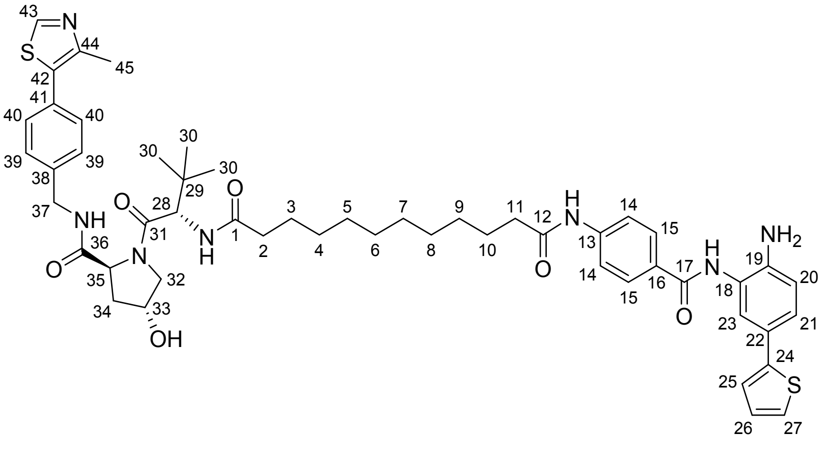

N1-(4-((2-amino-5-(thiophen-2-yl)phenyl)carbamoyl)phenyl)-N12-((S)-1-((2S,4R)-4-hydroxy-2-((4-(4-methylthiazol-5-yl)benzyl)carbamoyl)82yrrolidine-1-yl)-3,3-dimethyl-1-oxobutan-2-yl)dodecanediamide (19): Following general method H, Boc deprotection of **54s** (26.1 mg, 0.025 mmol) was performed to afford **19** (19.4 mg, 0.021 mmol, 82% yield) as a beige solid. ^{1}H NMR (400 MHz, CD_3OD) δ_H ppm 8.86 (s, 1 H, 43-CH), 7.98 (d, *J*=8.7 Hz, 2 H, 15-CH), 7.73 (d, *J*=8.7 Hz, 2 H, 14-CH), 7.49 (d, *J*=2.1 Hz, 1 H, 23-CH), 7.43 – 7.47 (m, 2 H, 40-CH), 7.38 – 7.42 (m, 2 H, 39-CH), 7.35 (dd, *J*=8.3, 2.1 Hz, 1 H, 21-CH), 7.16 – 7.26 (m, 2 H, 25-CH,27-CH), 7.01 (dd, *J*=5.1, 3.6 Hz, 1 H, 26-CH), 6.90 (d, *J*=8.3 Hz, 1 H, 20-CH), 4.63 (s, 1 H, 28-CH), 4.55 – 4.60 (m, 1 H, 35-CH), 4.52 (d, *J*=15.5 Hz, 1 H, 37-CH), 4.46 – 4.50 (m, 1 H, 33-CH), 4.34 (d, *J*=15.5 Hz, 1 H, 37-CH), 3.86 – 3.92 (m, 1 H, 32-CH), 3.75 – 3.81 (m, 1 H, 32-CH), 2.46 (s, 3 H, 45-CH_3), 2.40 (t, *J*=7.4 Hz, 2 H, 11-CH_2), 2.18 – 2.30 (m, 3 H, 2-CH_2,34-CH), 2.03 – 2.12 (m, 1 H, 34-CH), 1.70 (quin, *J*=7.4 Hz, 2 H, 10-CH_2), 1.54 – 1.63 (m, 2 H, 3-CH_2), 1.30 – 1.38 (m, 12 H, (4-9)-CH_2), 1.03

(s, 9 H, 30-CH_3). ^{13}C NMR (101 MHz, CD_3OD) δ_C ppm 176.2 (C1), 175.1 (C12), 174.6 (C36), 172.5 (C31), 168.5 (C17), 153.0 (C43), 149.2 (C44), 145.8 (C24), 143.8 (C19), 143.7 (C13), 140.4 (C38), 133.6 (C42), 131.6 (C41), 130.5 (C39), 130.4 (C16), 130.0 (C15), 129.1 (C40), 129.0 (C26), 126.6 (C18), 126.2 (C21), 125.5 (C22), 125.3 (C23), 124.3 (C27), 122.7 (C25), 120.5 (C14), 118.9 (C20), 71.2 (C33), 61.0 (C35), 59.1 (C28), 58.2 (C32), 43.8 (C37), 39.1 (C34), 38.2 (C11), 36.8 (C2), 36.7 (C29), 30.6 (2 x alkyl CH_2), 30.5 (2 x alkyl CH_2), 30.45 (alkyl CH_2), 30.4 (alkyl CH_2), 27.2 (C30), 27.1 (C3), 26.9 (C10), 16.0 (C45). HRMS (ESI) m/z: $[M+H]^+$ calculated for $C_{51}H_{64}N_7O_6S_2$: 934.4359, found 934.4355.

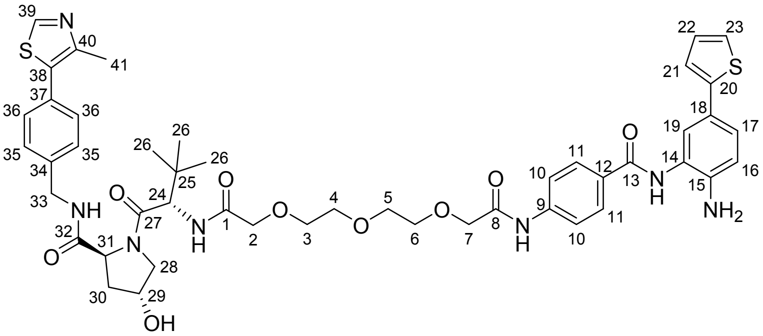

(2S,4R)-1-((S)-14-((4-((2-amino-5-(thiophen-2-yl)phenyl)carbamoyl)phenyl)amino)-2-(tert-butyl)-4,14-dioxo-6,9,12-trioxa-3-azatetradecanoyl)-4-hydroxy-N-(4-(4-methylthiazol-5-yl)benzyl)pyrrolidine-2-carboxamide (20): Following general method H, Boc deprotection of **54t** (30.3 mg, 0.029 mmol) was performed to afford **20** (26.8 mg, 0.029 mmol, 99% yield) as a pale yellow solid. ^1H NMR (400 MHz, CD_3OD) δ_H ppm 8.84 (s, 1 H, 39-CH), 7.98 (d, J=8.7 Hz, 2 H, 11-CH), 7.75 (d, J=8.7 Hz, 2 H, 10-CH), 7.49 (d, J=2.1 Hz, 1 H, 19-CH), 7.40 - 7.44 (m, 2 H, 36-CH), 7.36 - 7.39 (m, 2 H, 35-CH), 7.34 (dd, J=8.3, 2.1 Hz, 1 H, 17-CH), 7.22 (d, J=5.0 Hz, 1 H, 23-CH), 7.19 (d, J=3.7 Hz, 1 H, 21-CH), 7.01 (dd, J=5.0, 3.7 Hz, 1 H, 22-CH), 6.89 (d, J=8.3 Hz, 1 H, 16-CH), 4.68 (s, 1 H, 24-CH), 4.54 - 4.60 (m, 1 H, 31-CH), 4.51 (d, J=15.5 Hz, 1 H, 33-CH), 4.46 - 4.49 (m, 1 H, 29-CH), 4.31 (d, J=15.5 Hz, 1 H, 33-CH), 4.15 (d, J=15.8 Hz, 1 H, 7-CH), 4.09 (d, J=15.8 Hz, 1 H, 7-CH), 4.02 (d, J=15.6 Hz, 1 H, 2-CH), 3.91 (d, J=15.6 Hz, 1 H, 2-CH), 3.82 - 3.87 (m, 1 H, 28-CH), 3.69 - 3.81 (m, 9 H, 28-CH,(3-6)-CH_2), 2.45 (s, 3 H, 41-CH_3), 2.16 - 2.24 (m, 1 H, 30-CH), 2.03 - 2.11 (m, 1 H, 30-CH), 1.02 (s, 9 H, 26-CH_3). ^{13}C NMR (101 MHz, CD_3OD) δ_C ppm 174.5 (C32), 172.0 (C27), 171.7 (C1), 171.6 (C8), 168.4 (C13), 153.0 (C39), 149.2 (C40), 145.8 (C20), 143.8 (C15), 142.5 (C9), 140.4 (C34), 133.5 (C38), 131.7 (C37), 131.2 (C12), 130.5 (C35), 130.0 (C11), 129.1 (C36), 129.0 (C22), 126.5 (C14), 126.2 (C17), 125.4 (C18), 125.3 (C19), 124.3 (C23), 122.7 (C21), 121.2 (C10), 118.9 (C16), 72.3 (C3/4/5/6), 72.25 (C3/4/5/6), 71.7 (C7), 71.6 (C3/4/5/6), 71.55 (C3/4/5/6), 71.2 (C29), 71.1 (C2), 61.0 (C31), 58.2 (C28), 43.9 (C33), 39.1 (C30), 37.8 (C25), 37.4 (C24), 27.1 (C26), 16.0 (C41). HRMS (ESI) m/z: $[M+H]^+$ calculated for $C_{47}H_{56}N_7O_9S_2$: 926.3581, found 926.3556.

5.4. Preparation of Compounds **21-24**

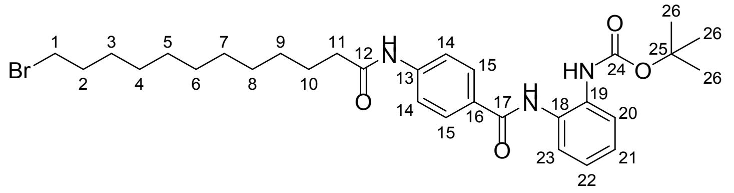

Tert-butyl (2-(4-(12-bromododecanamido)benzamido)phenyl)carbamate (56): Following general method E, **56** was obtained from **55** (332.6 mg, 1.19 mmol) and **35a** (300.0 mg, 0.92 mmol). The crude product was purified by column chromatography (0-50% EtOAc in hexane) to give **56** (207.0 mg, 0.35 mmol, 38% yield) as a white solid. ^{1}H NMR (400 MHz, $CDCl_3$) δ_H ppm 9.25 (br s, 1 H, 18-NH), 7.92 (br s, 1 H, 13-NH), 7.86 (d, *J*=8.7 Hz, 2 H, 15-CH), 7.68 (dd, *J*=7.6, 1.8 Hz, 1 H, 23-CH), 7.58 (d, *J*=8.7 Hz, 2 H, 14-CH), 7.29 (d, *J*=7.6, 1.8 Hz, 1 H, 20-CH), 7.09 - 7.18 (m, 3 H, 21,22-CH,19-NH), 3.40 (t, *J*=6.9 Hz, 2 H, 1-CH_2), 2.34 (t, *J*=7.6 Hz, 2 H, 11-CH_2), 1.85 (quin, *J*=7.1 Hz, 2 H, 2-CH_2), 1.69 (quin, *J*=7.4 Hz, 2 H, 10-CH_2), 1.50 (s, 9 H, 26-CH_3), 1.38 - 1.46 (m, 2 H, 3-CH_2), 1.26 - 1.35 (m, 12 H, (4-9)-CH_2). ^{13}C NMR (101 MHz, $CDCl_3$) δ_C ppm 172.0 (C12), 165.3 (C17), 154.6 (C24), 141.5 (C13), 130.6 (C18), 130.2 (C19), 129.1 (C16), 128.4 (C15), 126.0 (C22), 125.75 (C21), 125.7 (C23), 124.4 (C20), 119.1 (C14), 81.3 (C25), 37.6 (C11), 34.1 (C2), 32.8 (C3), 29.45 (alkyl CH_2), 29.4 (alkyl CH_2), 29.35 (alkyl CH_2), 29.3 (alkyl CH_2), 29.2 (alkyl CH_2), 28.7 (alkyl CH_2), 28.3 (C26), 28.1 (C3), 25.4 (C10). HRMS (ESI) m/z: $[M+H]^+$ calculated for $C_{30}H_{43}{}^{79}BrN_3O_4$: 588.2437, found 588.2427. HRMS (ESI) m/z: $[M+H]^+$ calculated for $C_{30}H_{43}{}^{81}BrN_3O_4$: 590.2416, found 590.2411.

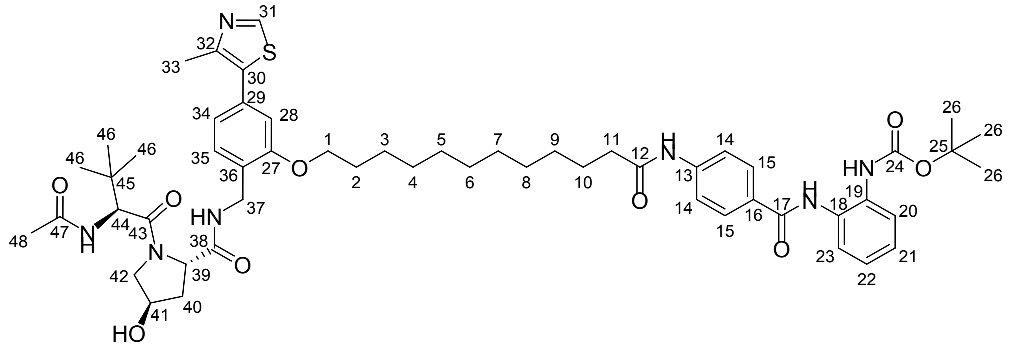

Tert-butyl (2-(4-(12-(2-(((2S,4R)-1-((S)-2-acetamido-3,3-dimethylbutanoyl)-4-hydroxypyrrolidine-2-carboxamido)methyl)-5-(4-methylthiazol-5-yl)phenoxy)dodecanamido)benzamido)phenyl)carbamate (57a): Following general method I, **57a** was obtained from (2S,4R)-1-((S)-2-acetamido-3,3-dimethylbutanoyl)-4-hydroxy-N-(2-hydroxy-4-(4-methylthiazol-5-yl)benzyl)pyrrolidine-2-carboxamide (**VH_032 phenol**, 17.2 mg, 0.034 mmol) and **56** (20.0 mg, 0.034 mmol). The crude product was purified by column chromatography (0-10% MeOH in DCM) to afford **57a** (17.8 mg, 0.018 mmol, 52% yield) as a white solid. ^{1}H NMR (400 MHz, CD_3OD) δ_H ppm 8.85 (s, 1 H, 31-CH), 7.93 (d, *J*=8.8 Hz, 2 H, 15-CH), 7.73 (d, *J*=8.8 Hz, 2 H, 14-CH), 7.55 - 7.62 (m, 1 H, 23-CH), 7.47 (d, *J*=8.3 Hz, 1 H, 35-CH), 7.40 - 7.45 (m, 1 H, 20-CH), 7.16 - 7.26 (m, 2 H, 21-CH,22-CH), 6.94 - 6.99 (m, 2 H, 28-CH,34-CH), 4.57 - 4.64 (m, 2 H, 39-CH,44-CH), 4.48 - 4.52 (m, 1 H, 41-CH), 4.46 (d, *J*=16.1 Hz, 1 H, 37-CH), 4.39 (d, *J*=16.1 Hz, 1 H, 37-CH), 4.04 (t, *J*=6.3 Hz, 2 H, 1-CH_2), 3.86 - 3.92 (m, 1 H, 42-CH), 3.75 - 3.82 (m, 1 H, 42-CH), 2.48 (s, 3 H, 33-CH_3), 2.40 (t, *J*=7.5 Hz, 2 H, 11-CH_2), 2.17 - 2.25 (m, 1 H, 40-CH), 2.07 - 2.15 (m, 1 H, 40-CH), 1.99 (s, 3 H, 48-CH_3), 1.78 - 1.86 (m, 2 H, 2-CH_2), 1.66 - 1.75 (m, 2 H, 10-CH_2), 1.47 - 1.54 (m, 11 H, 3-CH_2,26-CH_3), 1.32 - 1.42 (m, 12 H, (4-9)-CH_2), 1.02 (s, 9H, 46-CH_3). ^{13}C NMR (101 MHz, CD_3OD) δ_C 175.1 (C12), 174.6 (C38), 173.2

(C47), 172.5 (C43), 167.9 (C17), 158.1 (C27), 156.4 (C24), 152.9 (C31), 149.1 (C32), 143.9 (C13), 133.8 (C30), 133.2 (C19), 132.8 (C29), 131.8 (C18), 130.3 (C16), 129.7 (C15,C35), 128.1 (C36), 127.5 (C22), 127.3 (C23), 126.4 (C21), 125.7 (C20), 122.5 (C34), 120.4 (C14), 113.1 (C28), 81.9 (C25), 71.2 (C41), 69.5 (C1), 60.9 (C39), 59.3 (C44), 58.1 (C42), 39.4 (C37), 39.0 (C40), 38.2 (C11), 36.6 (C45), 30.8 (alkyl CH_2), 30.75 (alkyl CH_2), 30.7 (alkyl CH_2), 30.6 (alkyl CH_2), 30.55 (alkyl CH_2), 30.5 (C2), 30.4 (alkyl CH_2), 28.8 (C26), 27.4 (C3), 27.1 (C46), 26.9 (C10), 22.5 (C48), 16.1 (C33). HRMS (ESI) m/z: $[M+H]^+$ calculated for $C_{54}H_{74}N_7O_9S$: 996.5269 , found 996.5239.

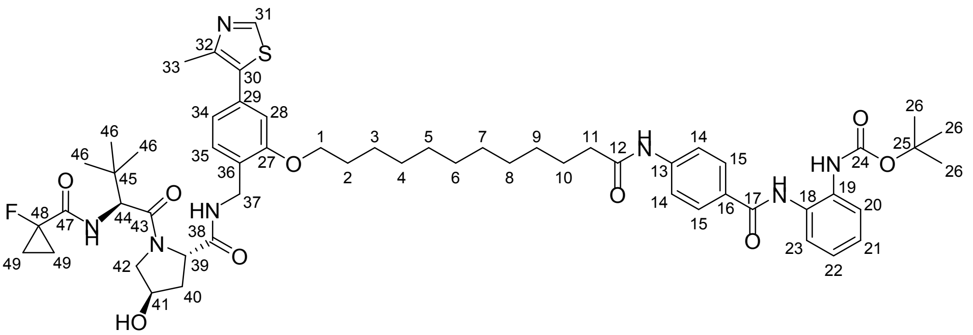

Tert-butyl (2-(4-(12-(2-(((2S,4R)-1-((S)-2-(1-fluorocyclopropane-1-carboxamido)-3,3-dimethylbutanoyl)-4-hydroxypyrrolidine-2-carboxamido)methyl)-5-(4-methylthiazol-5-yl)phenoxy)dodecanamido)benzamido)phenyl)carbamate (57b): Following general method I, **57b** was obtained from (2S,4R)-1-((S)-2-(1-fluorocyclopropane-1-carboxamido)-3,3-dimethylbutanoyl)-4-hydroxy-N-(2-hydroxy-4-(4-methylthiazol-5-yl)benzyl)pyrrolidine-2-carboxamide (**VH_101 phenol**, 18.6 mg, 0.034 mmol) and **56** (20.0 mg, 0.034 mmol). The crude product was purified by column chromatography (1-10% MeOH in DCM) to afford **57b** (24.2 mg, 0.022 mmol, 64% yield) as a white solid. ^{1}H NMR (400 MHz, CD_3OD) δ_H ppm 8.85 (s, 1 H, 31-CH), 7.93 (d, J=8.8 Hz, 2 H, 15-CH), 7.73 (d, J=8.8 Hz, 2 H, 14-CH), 7.56 - 7.62 (m, 1 H, 23-CH), 7.47 (d, J=7.7 Hz, 1 H, 35-CH), 7.40 - 7.44 (m, 1 H, 20-CH), 7.17 - 7.25 (m, 2 H, 21-CH,22-CH), 6.99 (dd, J=7.7, 1.5 Hz, 1 H, 34-CH), 6.97 (d, J=1.5 Hz, 1 H, 28-CH), 4.74 (d, J_{HF}=0.8 Hz, 1 H, 44-CH), 4.61 - 4.68 (m, 1 H, 39-CH), 4.49 - 4.53 (m, 1 H, 41-CH), 4.47 (d, J=16.1 Hz, 1 H, 37-CH), 4.39 (d, J=16.1 Hz, 1 H, 37-CH), 4.05 (t, J=6.3 Hz, 2 H, 1-CH_2), 3.82 - 3.88 (m, 1 H, 42-CH), 3.75 - 3.82 (m, 1 H, 42-CH), 2.48 (s, 3 H, 33-CH_3), 2.40 (t, J=7.5 Hz, 2 H, 11-CH_2), 2.20 - 2.28 (m, 1 H, 40-CH), 2.09 - 2.17 (m, 1 H, 40-CH), 1.78 - 1.87 (m, 2 H, 2-CH_2), 1.66 - 1.75 (m, 2 H, 10-CH_2), 1.46 - 1.54 (m, 11 H, 3-CH_2,26-CH_3), 1.30 - 1.40 (m, 16 H, (4-9)-CH_2,49-CH_2), 1.03 (s, 9 H, 46-CH_3). ^{13}C NMR (101 MHz, CD_3OD) δ_C 175.1 (C12), 174.4 (C38), 171.9 (C43), 171.6 (d, J_{CF}=20.4 Hz, C47), 167.9 (C17), 158.2 (C27), 156.4 (C24), 152.9 (C31), 149.2 (C32), 143.9 (C13), 133.8 (C30), 133.2 (C19), 132.9 (C29), 131.8 (C18), 130.3 (C16), 129.75 (C15), 129.7 (C35), 128.1 (C36), 127.5 (C22), 127.3 (C23), 126.4 (C21), 125.7 (C20), 122.5 (C34), 120.4 (C14), 113.2 (C28), 81.9 (C25), 79.3 (d, J_{CF}=231.6 Hz, C48), 71.2 (C41), 69.5 (C1), 60.9 (C39), 58.8 (C44), 58.3 (C42), 39.4 (C37), 39.0 (C40), 38.2 (C11), 37.5 (C45), 30.8 (alkyl CH_2), 30.75 (alkyl CH_2), 30.7 (alkyl CH_2), 30.6 (alkyl CH_2), 30.55 (alkyl CH_2), 30.5 (alkyl CH_2), 30.4 (alkyl CH_2), 28.8 (C26), 27.4 (C3), 27.0 (C46), 26.9 (C10), 16.1 (C33), 14.1 (app. t, J_{CF}=11.1 Hz, C49). ^{19}F NMR (376 MHz, CD_3OD) δ ppm -199.4. HRMS (ESI) m/z: $[M+H]^+$ calculated for $C_{56}H_{75}FN_7O_9S$: 1040.5331, found 1040.5304.

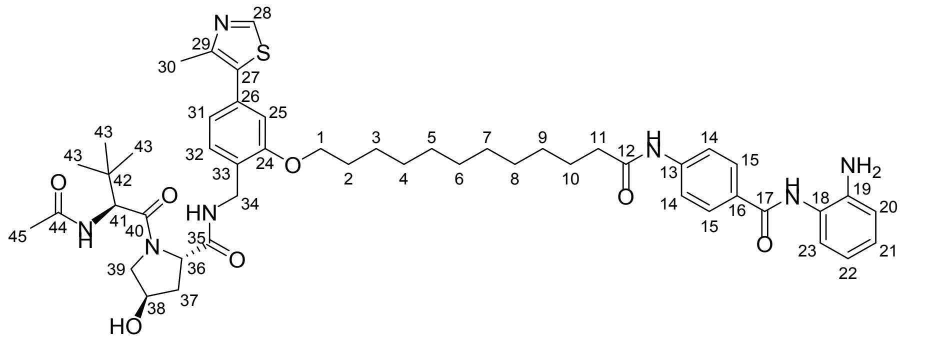

(2S,4R)-1-((S)-2-acetamido-3,3-dimethylbutanoyl)-N-(2-((12-((4-((2-aminophenyl)carbamoyl) phenyl)amino)-12-oxododecyl)oxy)-4-(4-methylthiazol-5-yl)benzyl)-4-hydroxypyrrolidine-2-carboxamide (21): Following general method H, Boc deprotection of **57a** (17.8 mg, 0.018 mmol) was performed to afford **21** (16.1 mg, 0.018 mmol, 99% yield) as a white solid. ^{1}H NMR (400 MHz, CD_3OD) δ_H ppm 8.86 (s, 1 H, 28-CH), 7.95 (d, *J*=8.7 Hz, 2 H, 15-CH), 7.72 (d, *J*=8.7 Hz, 2 H, 14-CH), 7.47 (d, *J*=8.2 Hz, 1 H, 32-CH), 7.17 (dd, *J*=7.7, 1.3 Hz, 1 H, 23-CH), 7.07 (app. td, *J*=7.7, 1.3 Hz, 1 H, 21-CH), 6.94 - 7.00 (m, 2 H, 25-CH,31-CH), 6.90 (dd, *J*=7.7, 1.3 Hz, 1 H, 20-CH), 6.76 (app. td, *J*=7.7, 1.3 Hz, 1 H, 22-CH), 4.57 - 4.65 (m, 2 H, 36-CH,41-CH), 4.48 - 4.52 (m, 1 H, 38-CH), 4.46 (d, *J*=16.1 Hz, 1 H, 34-CH), 4.39 (d, *J*=16.1 Hz, 1 H, 34-CH), 4.05 (t, *J*=6.3 Hz, 2 H, 1-CH_2), 3.86 - 3.92 (m, 1 H, 39-CH_2), 3.75 - 3.81 (m, 1 H, 39-CH_2), 2.48 (s, 3 H, 30-CH_3), 2.40 (t, J=7.5 Hz, 2 H, 11-CH_2), 2.17 - 2.25 (m, 1 H, 37-CH), 2.07 - 2.15 (m, 1 H, 37-CH), 1.99 (s, 3 H, 45-CH_3), 1.78 - 1.87 (m, 2 H, 2-CH_2), 1.71 (quin, *J*=7.3 Hz, 2 H, 10-CH_2), 1.46 - 1.56 (m, 2 H, 3-CH_2), 1.32 - 1.42 (m, 12 H, (4-9)-CH_2), 1.02 (s, 9 H, 43-CH_3). ^{13}C NMR (101 MHz, CD_3OD) δ_C ppm 175.1 (C12), 174.6 (C35), 173.3 (C44), 172.5 (C40), 168.4 (C17), 158.1 (C24), 152.9 (C28), 149.2 (C29), 143.9 (C19), 143.7 (C13), 133.8 (C27), 132.8 (C26), 130.5 (C16), 129.9 (C15), 129.7 (C35), 128.6 (C21), 128.1 (C33), 127.8 (C23), 125.6 (C18), 122.5 (C31), 120.4 (C14), 119.8 (C22), 118.9 (C20), 113.1 (C25), 71.2 (C38), 69.5 (C1), 60.9 (C36), 59.3 (C41), 58.1 (C39), 39.4 (C34), 39.0 (C37), 38.2 (C11), 36.6 (C42), 30.8 (alkyl CH_2), 30.75 (alkyl CH_2), 30.7 (alkyl CH_2), 30.6 (alkyl CH_2), 30.55 (alkyl CH_2), 30.5 (alkyl CH_2), 30.4 (alkyl CH_2), 27.4 (C3), 27.1 (C45), 26.9 (C10), 22.5 (C45), 16.1 (C30). HRMS (ESI) m/z: $[M+H]^+$ calculated for $C_{49}H_{66}N_7O_7S$: 896.4744, found 896.4744.

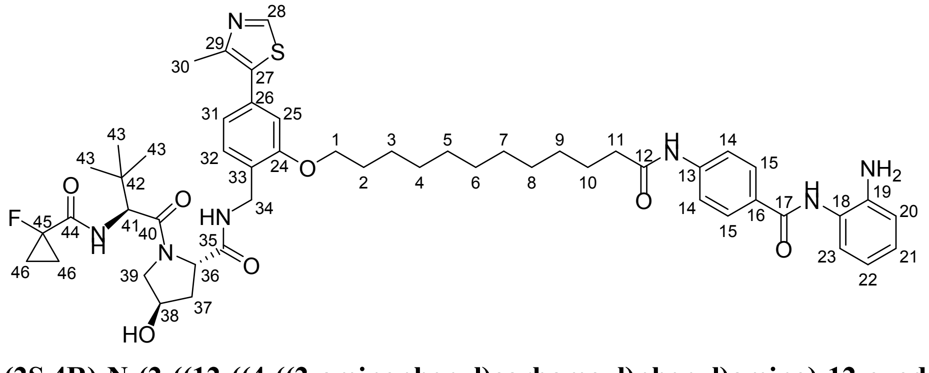

(2S,4R)-N-(2-((12-((4-((2-aminophenyl)carbamoyl)phenyl)amino)-12-oxododecyl)oxy)-4-(4-methylthiazol-5-yl)benzyl)-1-((S)-2-(1-fluorocyclopropane-1-carboxamido)-3,3-dimethylbutanoyl)-4-hydroxypyrrolidine-2-carboxamide (22): Following general method H, Boc deprotection of **57b** (24.2 mg, 0.022 mmol) was performed followed by purification by column chromatography (2-5% MeOH in DCM) to afford **22** (11.7 mg, 0.012 mmol, 57% yield) as a white solid. ^{1}H NMR (400 MHz, CD_3OD) δ_H ppm 8.86 (s, 1

H, 28-CH), 7.95 (d, *J*=8.7 Hz, 2 H, 15-CH), 7.72 (d, *J*=8.7 Hz, 2 H, 14-CH), 7.47 (d, *J*=7.7 Hz, 1 H, 32-CH), 7.17 (dd, *J*=7.8, 1.3 Hz, 1 H, 23-CH), 7.06 (app. td, *J*=7.8, 1.3 Hz, 1 H, 21-CH), 6.96 - 7.02 (m, 2 H, 25-CH,31-CH), 6.90 (dd, *J*=7.8, 1.3 Hz, 1 H, 20-CH), 6.76 (app. td, *J*=7.8, 1.3 Hz, 1 H, 22-CH), 4.74 (d, J_{HF}=0.8 Hz, 1 H, 41-CH), 4.60 - 4.67 (m, 1 H, 36-CH), 4.49 - 4.53 (m, 1 H, 38-CH), 4.47 (d, *J*=16.0 Hz, 1 H, 34-CH), 4.39 (d, *J*=16.0 Hz, 1 H, 34-CH), 4.06 (t, *J*=6.3 Hz, 2 H, 1-CH_2), 3.82 - 3.88 (m, 1 H, 39-CH), 3.76 - 3.81 (m, 1 H, 39-CH), 2.48 (s, 3 H, 30-CH_3), 2.40 (t, *J*=7.5 Hz, 2 H, 11-CH_2), 2.19 - 2.27 (m, 1 H, 37-CH), 2.09 - 2.16 (m, 1 H, 37-CH), 1.78 - 1.89 (m, 2 H, 2-CH_2), 1.64 - 1.76 (m, 2 H, 10-CH_2), 1.48 - 1.57 (m, 2 H, 3-CH_2), 1.28 - 1.40 (m, 16 H, (4-9)-CH_2,46-CH_2), 1.04 (s, 9 H, 43-CH_3). ^{13}C NMR (101 MHz, CD_3OD) δ_C ppm 175.1 (C12), 174.4 (C38), 171.9 (C40), 171.6 (d, J_{CF}=20.4 Hz, C44), 168.4 (C17), 158.2 (C24), 152.9 (C28), 149.2 (C29), 143.9 (C19), 143.7 (C13), 133.8 (C27), 132.9 (C26), 130.5 (C16), 129.9 (C15), 129.7 (C32), 128.6 (C21), 128.1 (C33), 127.8 (C23), 125.6 (C18), 122.5 (C31), 120.4 (C14), 119.8 (C22), 118.9 (C20), 113.2 (C25), 79.3 (d, J_{CF}=231.6 Hz, C45), 71.2 (C38), 69.5 (C1), 60.9 (C36), 58.8 (C41), 58.3 (C39), 39.4 (C34), 39.0 (C37), 38.2 (C11), 37.5 (C42), 30.8 (alkyl CH_2), 30.75 (alkyl CH_2), 30.7 (alkyl CH_2), 30.6 (alkyl CH_2), 30.55 (alkyl CH_2), 30.5 (alkyl CH_2), 30.4 (alkyl CH_2), 27.4 (C3), 27.0 (C43), 26.9 (C10), 16.1 (C30), 14.1 (app. t, J_{CF}=11.2 Hz, C46). ^{19}F NMR (376 MHz, CD_3OD) δ ppm -199.4. HRMS (ESI) m/z: $[M+H]^+$ calculated for $C_{51}H_{67}FN_7O_7S$: 940.4807, found 940.4781.

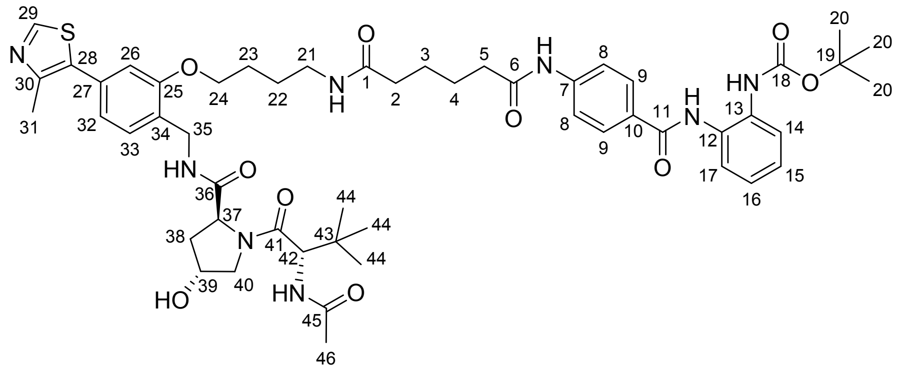

Tert-butyl (2-(4-(6-((4-(2-(((2S,4R)-1-((S)-2-acetamido-3,3-dimethylbutanoyl)-4-hydroxypyrrolidine-2-carboxamido)methyl)-5-(4-methylthiazol-5-yl)phenoxy)butyl)amino)-6-oxohexanamido)benzamido) phenyl)carbamate (59a): Following general method J, **59a** was obtained from **58** (19.7 mg, 0.043 mmol) and (2*S*,4*R*)-1-((*S*)-2-Acetamido-3,3-dimethylbutanoyl)-*N*-(2-(4-aminobutoxy)-4-(4-methylthiazol-5-yl)benzyl)-4-hydroxypyrrolidine-2-carboxamide (**VH032 phenol-alkylC4-amine**, 24.0 mg, 0.034 mmol). The crude product was purified by column chromatography (2-10% MeOH in DCM) to afford **59a** (28.5 mg, 0.028 mmol, 83% yield) as a white solid. ^{1}H NMR (400 MHz, CD_3OD) δ_H ppm 8.84 (s, 1 H, 29-CH), 7.90 (d, *J*=8.8 Hz, 2 H, 9-CH), 7.70 (d, *J*=8.8 Hz, 2 H, 8-CH), 7.54 - 7.62 (m, 1 H, 17-CH), 7.47 (d, *J*=7.7 Hz, 1 H, 33-CH), 7.41 - 7.45 (m, 1 H, 14-CH), 7.18 - 7.26 (m, 2 H, 15-CH,16-CH), 6.94 - 7.00 (m, 2 H, 26-CH,32-CH), 4.57 - 4.65 (m, 2 H, 37-CH,42-CH), 4.48 - 4.52 (m, 1 H, 39-CH), 4.45 (d, *J*=16.0 Hz, 1 H, 35-CH), 4.39 (d, *J*=16.0 Hz, 1 H, 35-CH), 4.06 (t, *J*=6.1 Hz, 2 H, 24-CH_2), 3.86 - 3.93 (m, 1 H, 40-CH), 3.74 - 3.82 (m, 1 H, 40-CH), 3.27 (t, *J*=6.8 Hz, 2 H, 21-CH_2), 2.47 (s, 3 H, 31-CH_3), 2.42 (t, *J*=6.9 Hz, 2 H, 5-CH_2), 2.23 (t, *J*=6.9 Hz, 2 H, 2-CH_2), 2.16 - 2.21 (m, 1 H, 38-CH), 2.06 - 2.15 (m, 1 H, 38-CH), 1.99 (s, 3 H, 46-CH_3), 1.82 - 1.91 (m, 2 H, 23-CH_2), 1.65 - 1.78 (m, 6 H, 3-CH_2,4-CH_2,22-CH_2), 1.49 (s, 9 H, 20-CH_3), 1.01 (s, 9 H, 44-CH_3). ^{13}C NMR

(101 MHz, CD_3OD) δ_C ppm 176.0 (C1), 174.6 (C6), 174.55 (C36), 173.2 (C45), 172.5 (C41), 167.9 (C11), 158.0 (C25), 156.4 (C18), 152.9 (C29), 149.2 (C30), 143.9 (C7), 133.8 (C28), 133.2 (C13), 132.9 (C34), 131.8 (C17), 130.3 (C10), 129.8 (C33), 129.7 (C9), 128.1 (C27), 127.5 (C15), 127.3 (C17), 126.4 (C16), 125.7 (C14), 122.6 (C32), 120.4 (C8), 113.1 (C26), 81.9 (C19), 71.2 (C39), 69.0 (C24), 60.9 (C37), 59.3 (C42), 58.1 (C40), 40.2 (C21), 39.4 (C35), 39.0 (C38), 37.9 (C5), 37.0 (C2), 36.6 (C43), 28.8 (C20), 27.9 (C23), 27.3 (C22), 27.1 (C44), 26.8 (C4), 26.5 (C3), 22.5 (C46), 16.1 (C31). HRMS (ESI) m/z: $[M+Na]^+$ calculated for $C_{52}H_{69}N_8O_{10}S$: 997.4857, found 997.4851.

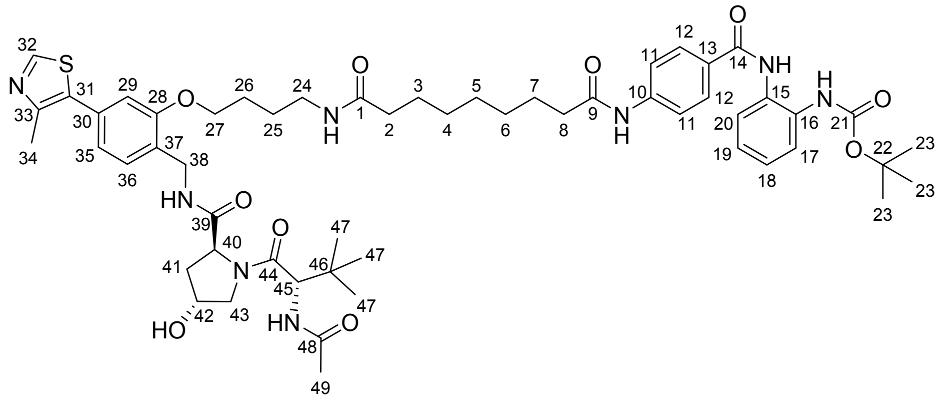

Tert-butyl (2-(4-(9-((4-(2-(((2S,4R)-1-((S)-2-acetamido-3,3-dimethylbutanoyl)-4-hydroxypyrrolidine-2-carboxamido)methyl)-5-(4-methylthiazol-5-yl)phenoxy)butyl)amino)-9-oxononanamido)benzamido) phenyl)carbamate (59b): Following general method J, **59b** was obtained from **53b** (21.7 mg, 0.044 mmol) and **VH_032 phenol-alkylC4-amine** (24.0 mg, 0.035 mmol). The crude product was purified by column chromatography (0-10% MeOH in DCM) to afford **59b** (33.1 mg, 0.032 mmol, 90% yield) as a pale yellow solid. ^1H NMR (400 MHz, CD_3OD) δ_H ppm 8.85 (s, 1 H, 32-CH), 7.91 (d, *J*=8.8 Hz, 2 H, 12-CH), 7.72 (d, *J*=8.8 Hz, 2 H, 11-CH), 7.55 - 7.62 (m, 1 H, 20-CH), 7.47 (d, *J*=8.3 Hz, 1 H, 36-CH), 7.41 - 7.45 (m, 1 H, 17-CH), 7.17 - 7.25 (m, 2 H, 18-CH,19-CH), 6.94 - 7.00 (m, 2 H, 29-CH,35-CH), 4.57 - 4.64 (m, 2 H, 40-CH,45-CH), 4.48 - 4.52 (m, 1 H, 42-CH), 4.45 (d, *J*=16.0 Hz, 1 H, 38-CH), 4.39 (d, *J*=16.0 Hz, 1 H, 38-CH), 4.07 (t, *J*=6.1 Hz, 2 H, 27-CH_2), 3.86 - 3.93 (m, 1 H, 43-CH), 3.74 - 3.82 (m, 1 H, 43-CH), 3.26 (t, *J*=6.8 Hz, 2 H, 24-CH_2), 2.47 (s, 3 H, 34-CH_3), 2.39 (t, *J*=7.4 Hz, 2 H, 8-CH_2), 2.15 - 2.24 (m, 3 H, 2-CH_2,41-CH), 2.07 - 2.14 (m, 1 H, 41-CH), 1.99 (s, 3 H, 49-CH_3), 1.81 - 1.90 (m, 2 H, 26-CH_2), 1.66 - 1.76 (m, 4 H, 7-CH_2,25-CH_2), 1.57 - 1.64 (m, 2 H, 3-CH_2), 1.49 (s, 9 H, 23-CH_3), 1.32 - 1.40 (m, 6 H, (4-6)-CH_2), 1.01 (s, 9 H, 47-CH_3). ^{13}C NMR (101 MHz, CD_3OD) δ_C ppm 176.4 (C1), 175.0 (C9), 174.6 (C39), 173.2 (C48), 172.5 (C44), 167.9 (C14), 158.0 (C28), 156.4 (C21), 152.9 (C32), 149.2 (C33), 143.9 (C10), 133.8 (C31), 133.2 (C16), 132.9 (C37), 131.8 (C20), 130.3 (C13), 129.8 (C36), 129.7 (C12), 128.1 (C30), 127.5 (C18), 127.3 (C20), 126.4 (C19), 125.7 (C17), 122.6 (C35), 120.4 (C11), 113.1 (C29), 81.9 (C22), 71.2 (C42), 69.0 (C27), 60.9 (C40), 59.3 (C45), 58.1 (C43), 40.1 (C24), 39.4 (C38), 39.0 (C41), 38.2 (C8), 37.3 (C2), 36.6 (C46), 30.3 (C4/5/6), 30.25 (C4/5/6), 30.2 (C4/5/6), 28.8 (C23), 27.9 (C26), 27.3 (C25), 27.2 (C47), 27.1 (C3), 26.8 (C7), 22.5 (C49), 16.1 (C34). HRMS (ESI) m/z: $[M+H]^+$ calculated for $C_{55}H_{75}N_8O_{10}S$: 1039.5327, found 1039.5293.

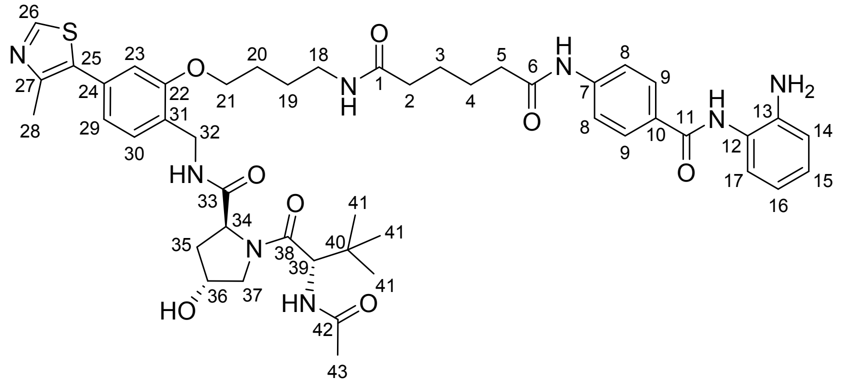

N1-(4-(2-(((2S,4R)-1-((S)-2-acetamido-3,3-dimethylbutanoyl)-4-hydroxypyrrolidine-2-carboxamido) methyl)-5-(4-methylthiazol-5-yl)phenoxy)butyl)-N6-(4-((2-aminophenyl)carbamoyl)phenyl)adipamide (23): Following general method H, Boc deprotection of **59a** (22.9 mg, 0.023 mmol) was performed to afford **23** (20.2 mg, 0.022 mmol 97% yield) as a pale yellow solid. ^{1}H NMR (400 MHz, CD_3OD) δ_H ppm 8.85 (s, 1 H, 26-CH), 7.92 (d, *J*=8.8 Hz, 2 H, 9-CH), 7.70 (d, *J*=8.8 Hz, 2 H, 8-CH), 7.47 (d, *J*=7.8 Hz, 1 H, 30-CH), 7.17 (dd, *J*=7.7, 1.3 Hz, 1 H, 17-CH), 7.07 (app. td, *J*=7.7, 1.3 Hz, 1 H, 15-CH), 6.94 - 7.00 (m, 2 H, 23-CH,29-CH), 6.90 (dd, *J*=7.7, 1.3 Hz, 1 H, 14-CH), 6.76 (app. td, *J*=7.7, 1.3 Hz, 1 H, 16-CH), 4.58 - 4.64 (m, 2 H, 34-CH,39-CH), 4.48 - 4.51 (m, 1 H, 36-CH), 4.45 (d, *J*=15.9 Hz, 1 H, 32-CH), 4.39 (d, *J*=15.9 Hz, 1 H, 32-CH), 4.07 (t, *J*=6.1 Hz, 2 H, 21-CH_2), 3.85 - 3.93 (m, 1 H, 37-CH), 3.74 - 3.81 (m, 1 H, 37-CH), 3.27 (t, *J*=7.0 Hz, 2 H, 18-CH_2), 2.47 (s, 3 H, 28-CH_3), 2.42 (t, *J*=6.9 Hz, 2 H, 5-CH_2), 2.23 (t, *J*=6.9 Hz, 2 H, 2-CH_2), 2.16 - 2.21 (m, 1 H, 35-CH), 2.06 - 2.14 (m, 1 H, 35-CH), 1.99 (s, 3 H, 43-CH_3), 1.82 - 1.91 (m, 2 H, 20-CH_2), 1.66 - 1.78 (m, 6 H, 3-CH_2,4-CH_2,19-CH_2), 1.01 (s, 9 H, 41-CH_3). ^{13}C NMR (101 MHz, CD_3OD) δ_C ppm 176.0 (C1), 174.6 (C6), 174.55 (C33), 173.3 (C42), 172.5 (C38), 168.4 (C11), 158.1 (C22), 153.0 (C26), 149.2 (C27), 143.9 (C7), 143.6 (C13), 133.8 (C25), 132.9 (C31), 130.5 (C10), 129.9 (C9), 129.8 (C30), 128.6 (C15), 128.1 (C24), 127.8 (C17), 125.6 (C12), 122.6 (C29), 120.4 (C8), 119.8 (C16), 118.9 (C14), 113.1 (C23), 71.2 (C36), 69.0 (C21), 60.9 (C34), 59.3 (C39), 58.1 (C37), 40.2 (C18), 39.4 (C32), 39.0 (C35), 37.9 (C5), 37.0 (C2), 36.6 (C40), 27.9 (C20), 27.3 (C19), 27.1 (C41), 26.8 (C4), 26.5 (C3), 22.5 (C43), 16.1 (C28). HRMS (ESI) m/z: $[M+H]^+$ calculated for $C_{47}H_{61}N_8O_8S$: 897.4333, found 897.4324.

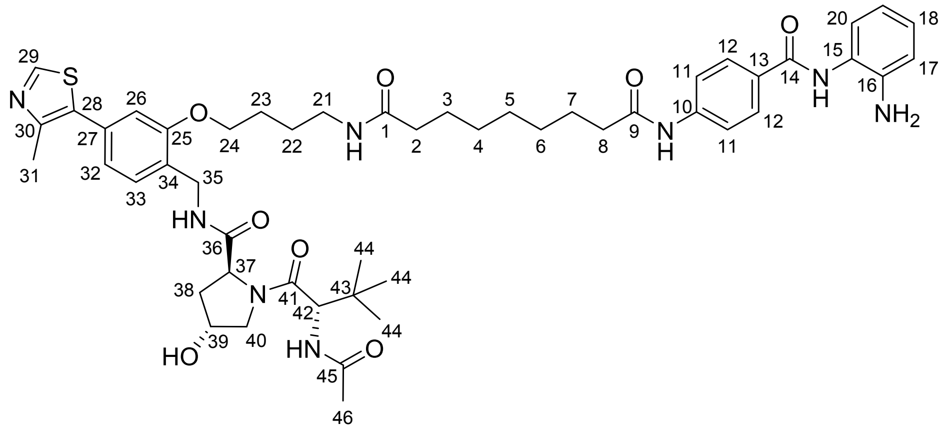

N1-(4-(2-(((2S,4R)-1-((S)-2-acetamido-3,3-dimethylbutanoyl)-4-hydroxypyrrolidine-2-carboxamido) methyl)-5-(4-methylthiazol-5-yl)phenoxy)butyl)-N9-(4-((2-aminophenyl)carbamoyl)phenyl) nonanediamide (24): Following general method H, Boc deprotection of **59b** (33.1 mg, 0.032 mmol) was

performed to afford **24** (24.1 mg, 0.025 mmol, 80% yield) as a pale yellow solid. ^{1}H NMR (400 MHz, CD_3OD) δ_H ppm 8.85 (s, 1 H, 29-CH), 7.93 (d, J=8.7 Hz, 2 H, 12-CH), 7.71 (d, J=8.7 Hz, 2 H, 11-CH), 7.47 (d, J=8.2 Hz, 1 H, 33-CH), 7.17 (dd, J=7.7, 1.3 Hz, 1 H, 20-CH), 7.07 (app. td, J=7.7, 1.3 Hz, 1 H, 18-CH), 6.95 - 6.99 (m, 2 H, 26-CH,32-CH), 6.90 (dd, J=7.7, 1.3 Hz, 1 H, 17-CH), 6.76 (app. td, J=7.7, 1.3 Hz, 1 H, 19-CH), 4.57 - 4.64 (m, 2 H, 37-CH,42-CH), 4.48 - 4.52 (m, 1 H, 39-CH), 4.45 (d, J=15.9 Hz, 1 H, 35-CH), 4.39 (d, J=15.9 Hz, 1 H, 5-CH), 4.07 (t, J=6.1 Hz, 2 H, 24-CH_2), 3.85 - 3.93 (m, 1 H, 40-CH), 3.74 - 3.81 (m, 1 H, 40-CH), 3.26 (t, J=6.8 Hz, 2 H, 21-CH_2), 2.48 (s, 3 H, 31-CH_3), 2.38 (t, J=7.4 Hz, 2 H, 8-CH_2), 2.14 - 2.22 (m, 3 H, 2-CH_2,38-CH), 2.06 - 2.14 (m, 1 H, 38-CH), 1.99 (s, 3 H, 46-CH_3), 1.81 - 1.90 (m, 2 H, 23-CH_2), 1.66 - 1.76 (m, 4 H, 7-CH_2,22-CH_2), 1.60 (quin, J=7.2 Hz, 2 H, 3-CH_2), 1.33 - 1.40 (m, 6 H, (4-6)-CH_2), 1.01 (s, 9 H, 44-CH_3). ^{13}C NMR (101 MHz, CD_3OD) δ_C ppm 176.4 (C1), 175.0 (C9), 174.6 (C36), 173.2 (C45), 172.5 (C41), 168.4 (C14), 158.0 (C25), 152.9 (C29), 149.2 (C30), 143.9 (C10), 143.6 (C16), 133.8 (C28), 132.9 (C34), 130.5 (C13), 129.9 (C12), 129.8 (C33), 128.6 (C18), 128.1 (C27), 127.8 (C20), 125.6 (C15), 122.6 (C32), 120.4 (C11), 119.8 (C19), 118.9 (C17), 113.1 (C26), 71.2 (C39), 69.0 (C24), 60.9 (C37), 59.3 (C42), 58.1 (C40), 40.1 (C21), 39.4 (C35), 39.0 (C38), 38.2 (C8), 37.3 (C2), 36.6 (C43), 30.3 (C4/5/6), 30.25 (C4/5/6), 30.2 (C4/5/6), 27.9 (C23), 27.3 (C22), 27.2 (C44), 27.1 (C3), 26.8 (C7), 22.5 (C46), 16.1 (C31). HRMS (ESI) m/z: $[M+H]^+$ calculated for $C_{50}H_{67}N_8O_8S$: 939.4803, found 939.4764.

5.5. Preparation of JPS016 Negative Control (**25**)

Scheme S3. Synthesis of 25.

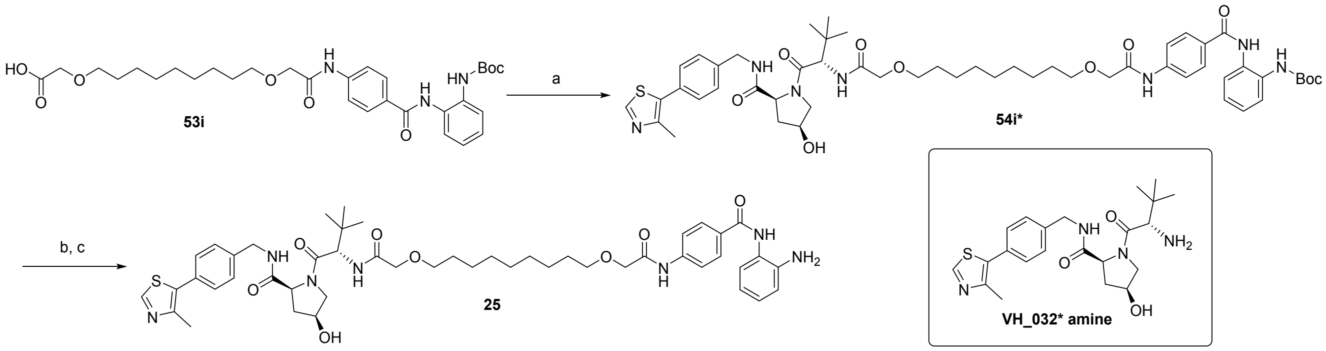

Reagents and conditions: (a) **VH_032* amine**, HATU, DIPEA, DMF, r.t., overnight; (b) TFA, DCM, r.t., 4 h; (c) MP-carbonate resin, MeOH, r.t., 2 h.

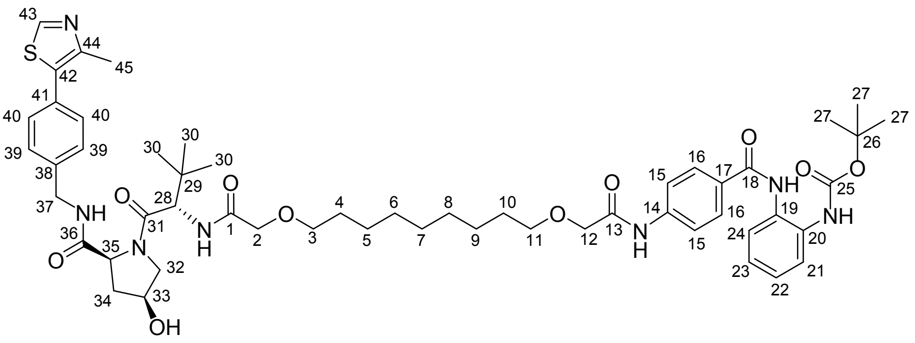

Tert-butyl (2-(4-(2-((9-(2-(((S)-1-((2S,4S)-4-hydroxy-2-((4-(4-methylthiazol-5-yl)benzyl)carbamoyl) pyrrolidin-1-yl)-3,3-dimethyl-1-oxobutan-2-yl)amino)-2-oxoethoxy)nonyl)oxy)acetamido)benzamido) phenyl)carbamate (54i*): To a solution of **53i** (27.8 mg, 0.047 mmol) in dry DMF (1 mL) at 0 °C, DIPEA (0.023 mL, 0.129 mmol) and HATU (21.3 mg, 0.056 mmol) were added. The reaction mixture was stirred for 15 minutes, after which a solution of (2S,4S)-1-[(2S)-2-Amino-3,3-dimethyl-butanoyl]-4-hydroxy-N-[[4-(4-methylthiazol-5-yl)phenyl]methyl]pyrrolidine-2-carboxamide dihydrochloride (**VH_032* amine**, 21.7 mg, 0.043 mmol) in DMF (1 mL) was added slowly and the resultant solution stirred at room temperature for 16 hours. The reaction mixture was diluted in EtOAc (10 mL), then washed with sat. $NaHCO_3$ (2 x 5 mL) and sat. NaCl (2 x 5 mL). The organic layer was dried over $MgSO_4$, filtered and concentrated *in vacuo* to afford a dark yellow tar. The crude product was purified by column chromatography (0-8% MeOH in DCM) to afford **54i*** (30.9 mg, 0.043 mmol, 72% yield) as a white solid. ^{1}H NMR (400 MHz, CD_3OD) δ_H ppm 8.86 (s, 1 H, 43-CH), 7.95 (d, *J*=8.7 Hz, 2 H, 16-CH), 7.78 (d, *J*=8.7 Hz, 2 H, 15-CH), 7.55 - 7.63 (m, 1 H, 24-CH), 7.48 (d, *J*=9.1 Hz, 1 H, 1-NH), 7.42 - 7.45 (m, 3 H, 21-CH,40-CH), 7.37 - 7.41 (m, 2 H, 39-CH), 7.18 - 7.26 (m, 2 H, 22-CH,23-CH), 4.59 - 4.64 (m, 1 H, 35-CH), 4.48 - 4.57 (m, 2 H, 28-CH,37-CH), 4.33 - 4.42 (m, 2 H, 33-CH,37-CH), 4.08 (s, 2 H, 12-CH_2), 3.90 - 4.01 (m, 3 H, 2-CH_2,32-CH), 3.68 – 3.74 (m, 1 H, 32-CH), 3.57 (t, *J*=6.6 Hz, 2 H, 11-CH_2), 3.53 (t, *J*=6.4 Hz, 2 H, 3-CH_2), 2.46 (s, 3 H, 45-CH_3), 2.38 - 2.45 (m, 1 H, 34-CH), 1.93 - 2.01 (m, 1 H, 34-CH), 1.56 - 1.70 (m, 4 H, 4-CH_2,10-CH_2), 1.49 (s, 9 H, 27-CH_3), 1.31 - 1.44 (m, 10 H, (5-9)-CH_2), 1.03 (s, 9 H, 30-CH_3). ^{13}C NMR (101 MHz, CD_3OD) δ_C ppm 174.9 (C36), 172.4 (C1), 172.1 (C31), 171.4 (C13), 167.8 (C18), 156.4 (C25), 153.0 (C43), 149.2 (C44), 142.8 (C14), 140.1 (C38), 133.5 (C42), 133.2 (C20), 131.8 (C41), 131.7 (C19), 130.9 (C17), 130.5 (C39), 129.8 (C16), 129.1 (C40), 127.5 (C22/23), 127.3 (C24), 126.4 (C22/23), 125.7 (C21), 120.9 (C15), 81.9 (C26), 73.2 (C11), 73.1 (C3), 71.6 (C12/33), 71.5 (C12/33), 70.8 (C2), 61.1 (C35), 58.1 (C28), 57.7 (C32), 44.0 (C37), 38.0 (C34), 36.8 (C29), 30.8 (alkyl CH_2), 30.8 (alkyl CH_2), 30.6 (alkyl CH_2), 30.6 (alkyl CH_2), 30.6 (alkyl CH_2), 28.8 (C27), 27.4 (C5/9), 27.3 (C5/9), 27.1 (C30), 16.0 (C45). HRMS (ESI) m/z: $[M+H]^+$ calculated for $C_{53}H_{72}N_7O_{10}S$: 998.5061, found 998.5040.

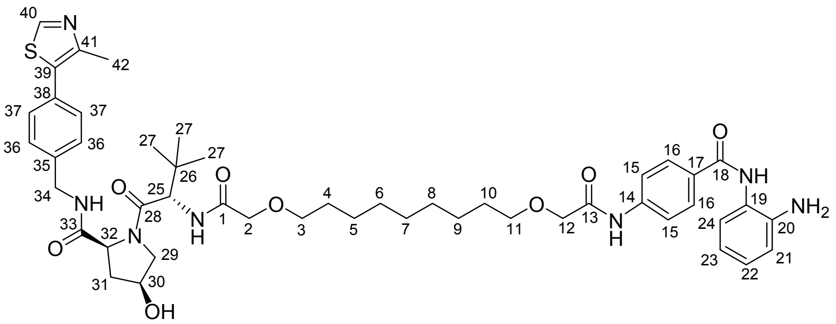

(2S,4S)-1-((S)-2-(2-((9-(2-((4-((2-aminophenyl)carbamoyl)phenyl)amino)-2-oxoethoxy)nonyl)oxy) acetamido)-3,3-dimethylbutanoyl)-4-hydroxy-N-(4-(4-methylthiazol-5-yl)benzyl)pyrrolidine-2-carboxamide (25): Following general method H, Boc deprotection of **54i*** (33.9 mg, 0.034 mmol) was performed to afford **25** (28.3 mg, 0.031 mmol, 92% yield) as a pale yellow solid. Prior to biological evaluation the product was further purified by semi-preparative HPLC (5-95% MeCN in H_2O, 260 nm, 45 min gradient). ^1H NMR (400 MHz, CD_3OD) δ_H ppm 8.86 (s, 1 H, 40-CH), 7.97 (d, *J*=8.8 Hz, 2 H, 16-CH), 7.76 (d, *J*=8.8 Hz, 2 H, 15-CH), 7.42 - 7.46 (m, 2 H, 37-CH), 7.37 - 7.41 (m, 2 H, 36-CH), 7.17 (dd, *J*=7.7, 1.4 Hz, 1 H, 24-CH), 7.07 (app. td, *J*=7.7, 1.4 Hz, 1 H, 22-CH), 6.90 (dd, *J*=7.7, 1.4 Hz, 1 H, 21-CH), 6.76 (app. td, *J*=7.7, 1.4 Hz, 1 H, 23-CH), 4.61 (s, 1 H, 25-CH), 4.54 (d, *J*=15.2 Hz, 1 H, 34-CH), 4.47 - 4.51 (m, 1 H, 32-CH), 4.33 - 4.43 (m, 2 H, 30-CH,34-CH), 4.08 (s, 2 H, 12-CH_2), 3.89 - 4.01 (m, 3 H, 2-CH_2,29-CH), 3.68 - 3.74 (m, 1 H, 29-CH), 3.57 (t, *J*=6.6 Hz, 2 H, 11-CH_2), 3.53 (t, *J*=6.4 Hz, 2 H, 3-CH_2), 2.46 (s, 3 H, 42-CH_3), 2.37 - 2.45 (m, 1 H, 31-CH), 1.93 - 2.00 (m, 1 H, 31-CH), 1.57 - 1.70 (m, 4 H, 4-CH_2,10-CH_2), 1.31 - 1.44 (m, 10 H, (5-9)-CH_2), 1.03 (s, 9 H, 27-CH_3). ^{13}C NMR (101 MHz, CD_3OD) δ_C ppm 174.9 (C33), 172.4 (C1), 172.1 (C28), 171.4 (C13), 168.2 (C18), 153.0 (C40), 149.2 (C41), 144.0 (C20), 142.6 (C14), 140.1 (C35), 133.5 (C39), 131.7 (C38), 131.1 (C17), 130.5 (C36), 130.0 (C16), 129.1 (C37), 128.7 (C22), 127.8 (C24), 125.5 (C19), 120.9 (C15), 119.8 (C23), 118.9 (C21), 73.2 (C11), 73.1 (C3), 71.6 (C12/30), 71.5 (C12/30), 70.8 (C2), 61.1 (C32), 58.1 (C25), 57.7 (C29), 44.0 (C34), 38.0 (C31), 36.8 (C26), 30.8 (alkyl CH_2), 30.75 (alkyl CH_2), 30.65 (alkyl CH_2), 30.6 (alkyl CH_2), 30.55 (alkyl CH_2), 27.4 (C5/9), 27.2 (C5/9), 27.1 (C30), 16.0 (C42). HRMS (ESI) m/z: $[M+H]^+$ calculated for $C_{48}H_{64}N_7O_8S$: 898.4537, found 898.4543.

6. UPLC Traces of Potent Degraders

The target compounds were analysed by UPLC-MS using a Xevo G2-XS QToF mass spectrometer (Waters) coupled to an Acquity LC system (Waters) with an Acquity UPLC BEH C18 column (130Å, 1.7 µm, 2.1 x 50 mm, Waters). The flow rate was 0.6 ml/min and the gradient was as follows: 95% Solvent A (0.1% formic acid in water) with 5% solvent B (0.1% formic acid in acetonitrile) was held constant for 0.5 min, followed by a linear gradient to 100% B over the next 9.5 min. After 1.6 min at 100% solvent B, the gradient was returned to 95% solvent A and 5% solvent B over 0.2 min and held for a further 1.2min to re-equilibrate the column. 3µl of sample was injected and DAD detector was set to acquire absorbance at 260 nm wavelength with 2.4nm resolution. PROTAC solutions were made up in HPLC Grade acetonitrile (MeCN) and deionised water (1:1).

Figure S11. UPLC results for potent degraders (JPS004, JPS014, JPS016, JPS035, JPS036).

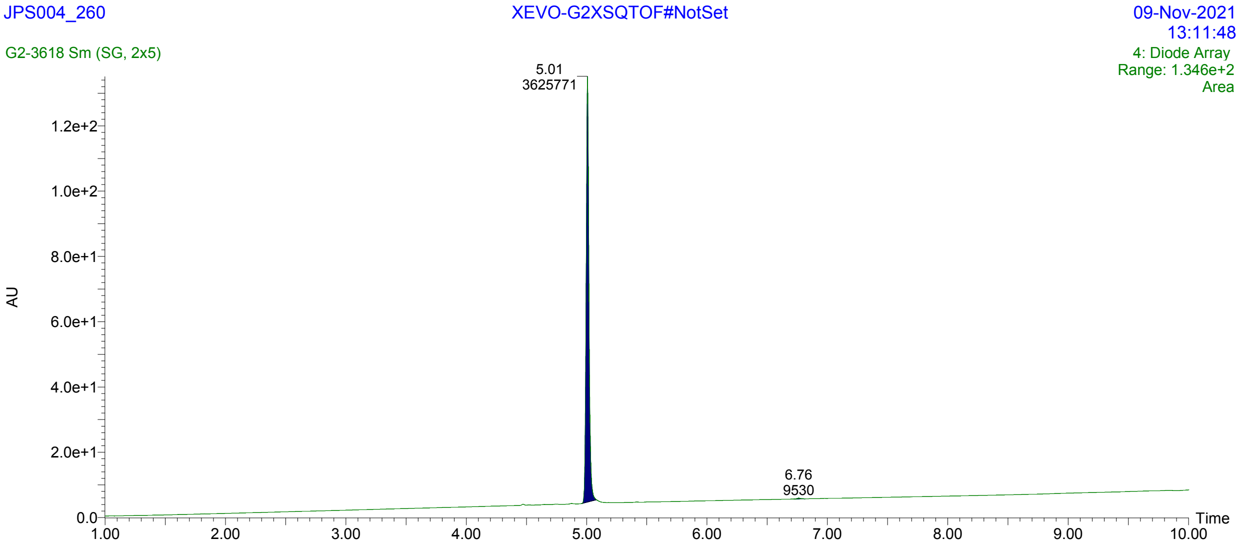

Peak #	Ret. Time (min)	Height (AU)	Area	Ret. Area %
1	5.008	130331384	3625771	99.738%
2	6.761	263639	9530	0.262%

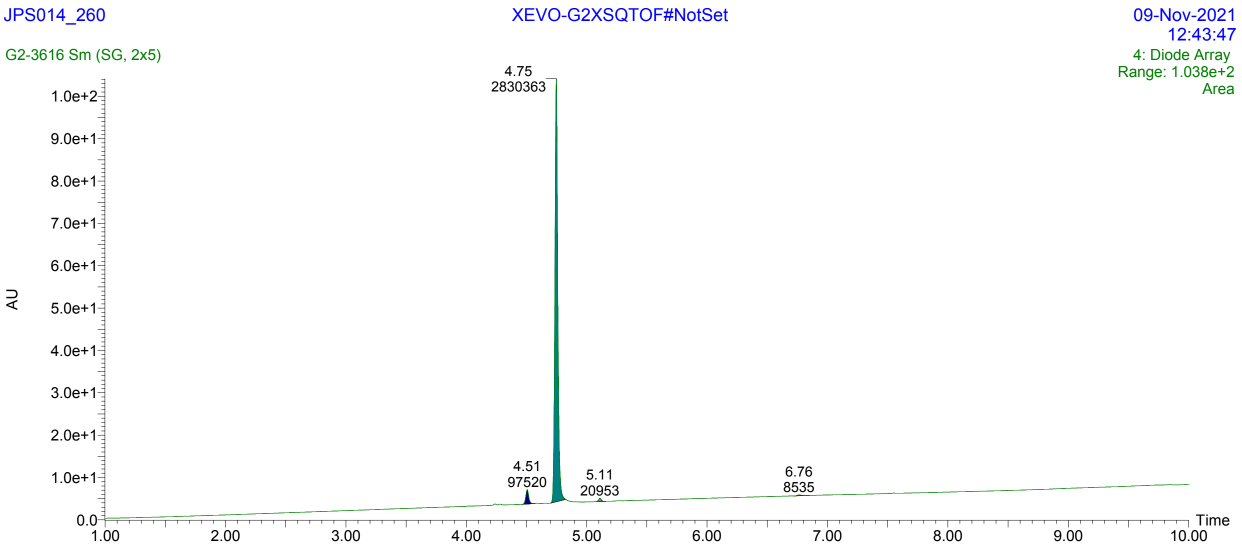

Peak #	Ret. Time (min)	Height (AU)	Area	Ret. Area %
1	4.506	3407423	97520	3.307%
2	4.748	99842536	2830363	95.982%
3	5.109	784252	20953	0.711%

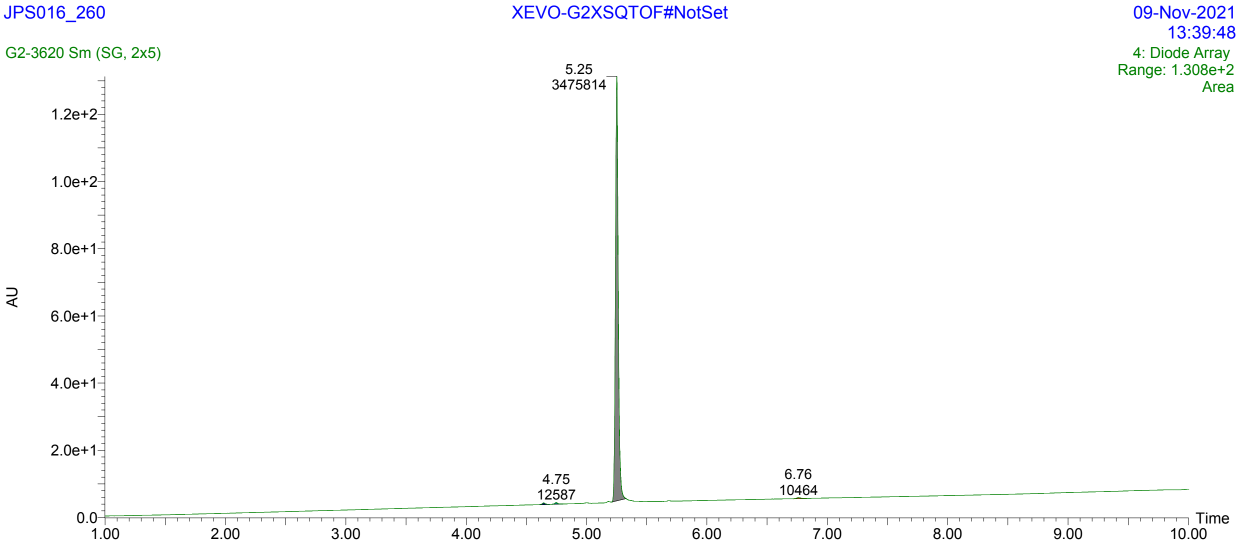

Peak #	Ret. Time (min)	Height (AU)	Area	Ret. Area %
1	4.643	454846	11948	0.340%
2	4.748	458415	12587	0.359%
3	5.251	126276664	3475814	99.003%
4	6.761	282770	10464	0.340%

JPS035_260 XEVO-G2XSQTOF#NotSet 09-Nov-2021 14:08:12

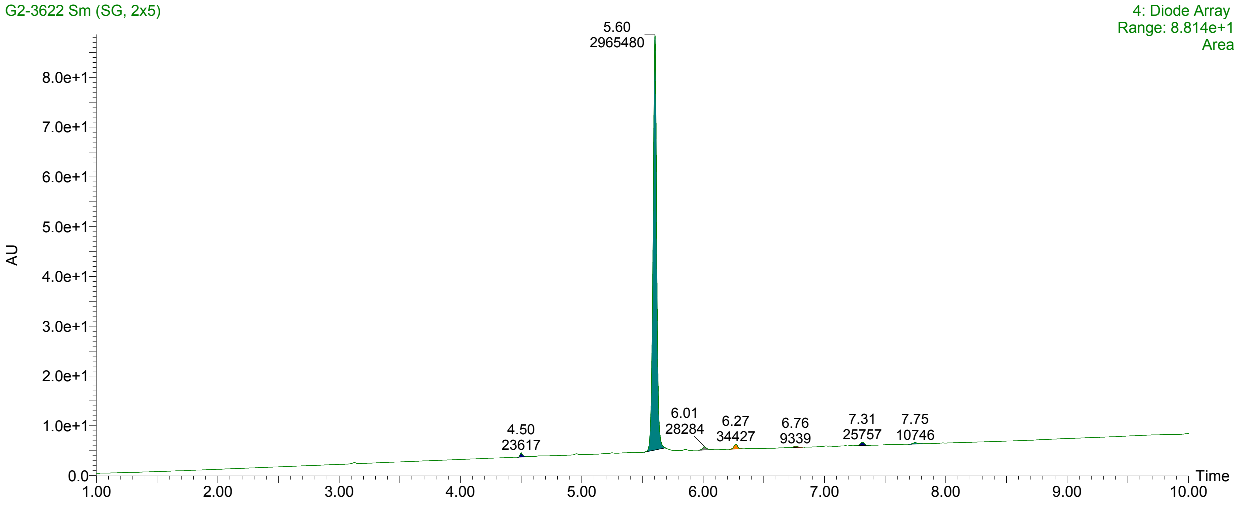

Peak #	Ret. Time (min)	Height (AU)	Area	Ret. Area %
1	4.500	857918	23617	0.762%
2	5.604	83453800	2965480	95.733%
3	6.010	676900	28284	0.913%
4	6.270	989001	34427	1.111%
5	6.761	262151	9339	0.301%
6	7.312	653188	25757	0.832%
7	7.747	290649	10746	0.347%

JPS036_260 XEVO-G2XSQTOF#NotSet 09-Nov-2021 14:36:12

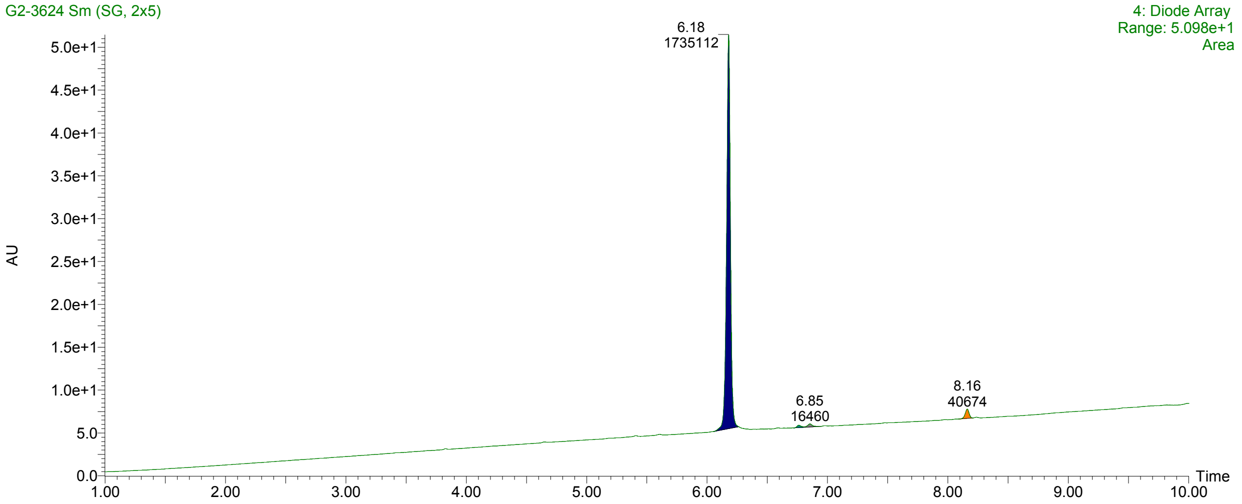

Peak #	Ret. Time (min)	Height (AU)	Area	Ret. Area %
1	6.179	45944088	1735112	96.176%
2	6.761	275868	11861	0.657%
3	6.854	361773	16460	0.912%
4	8.161	1085871	40674	2.255%

References

1. Song, Y.; Dagil, L.; Fairall, L.; Robertson, N.; Wu, M.; Ragan, T.; Savva, C.; Saleh, A.; Morone, N.; Kunze, M.; Jamieson, A.; Cole, P.; Hansen, F.; Schwabe, J.; Mechanism of Crosstalk between the LSD1 Demethylase and HDAC1 Deacetylase in the CoREST Complex. *Cell Reports* **2020**, *30* (8), 2699-2711.
2. Watson, P.; Fairall, L.; Santos, G.; Schwabe, J.; Structure of HDAC3 bound to corepressor and inositol tetraphosphate. *Nature* **2012**, *481* (7381), 335–340.
3. Kim, D.; Langmead, B; Salzberg, S. HISAT: a fast spliced aligner with low memory requirements. *Nat. Methods* **2015**, *12*, 357–360. DOI:10.1038/nmeth.3317
4. Andrews, S. FastQC: A Quality Control Tool for High Throughput Sequence Data. **2010.**
5. Ewels, P.; Magnusson, M.; Lundin, S.; Käller, M. MultiQC: summarize analysis results for multiple tools and samples in a single report. *Bioinformatics* **2016**, *32***,** 3047-3048. DOI:10.1093/bioinformatics/btw354
6. Li, H.; Handsaker, B.; Wysoker, A.; Fennell, T.; Ruan, J.; Homer, N.; Marth, G.; Abecasis, G.; Durbin, R. The Sequence Alignment/Map format and SAMtools, *Bioinformatics* **2009**, *25*, 2078–2079. DOI:10.1093/bioinformatics/btp352
7. Dyer, N.P.; Shahrezaei, V.; Hebenstreit, D. LiBiNorm: an htseq-count analogue with improved normalisation of Smart-seq2 data and library preparation diagnostics. *PeerJ.* **2019**, *7,* e6222. DOI:10.7717/peerj.6222
8. Love, M.I.; Huber, W.; Anders, S. Moderated estimation of fold change and dispersion for RNA-seq data with DESeq2. *Genome Biol.* **2014**, *15*, 550. DOI:10.1186/s13059-014-0550-8
9. Alexa, A.; Rahnenfuhrer, J. topGO: Enrichment Analysis for Gene Ontology. R package version 2.44.0. **2021**.
10. Carlson, M. org.Hs.eg.db: Genome wide annotation for Human. R package version 3.8.2. **2019**.
11. Girardini, M.; Maniaci, C.; Hughes, S.J.; Testa, A.; Ciulli, A. Cereblon versus VHL: Hijacking E3 ligases against each other using PROTACs. Bioorganic Med. Chem. Lett. **2019**, *27*, 2466-2479. DOI:10.1016/j.bmc.2019.02.048
12. Smalley, J.P.; Adams, G.E.; Millard, C.J.; Song, Y.; Norris, J.K.S.; Schwabe, J.W.R.; Cowley, S.M.; Hodgkinson, J.T. PROTAC-mediated degradation of class I histone deacetylase enzymes in corepressor complexes. *Chem. Commun.* **2020**, *56* (32), 4476-4479. DOI:10.1039/D0CC01485K

Appendix: ^{1}H NMR and ^{13}C NMR of Novel PROTACs

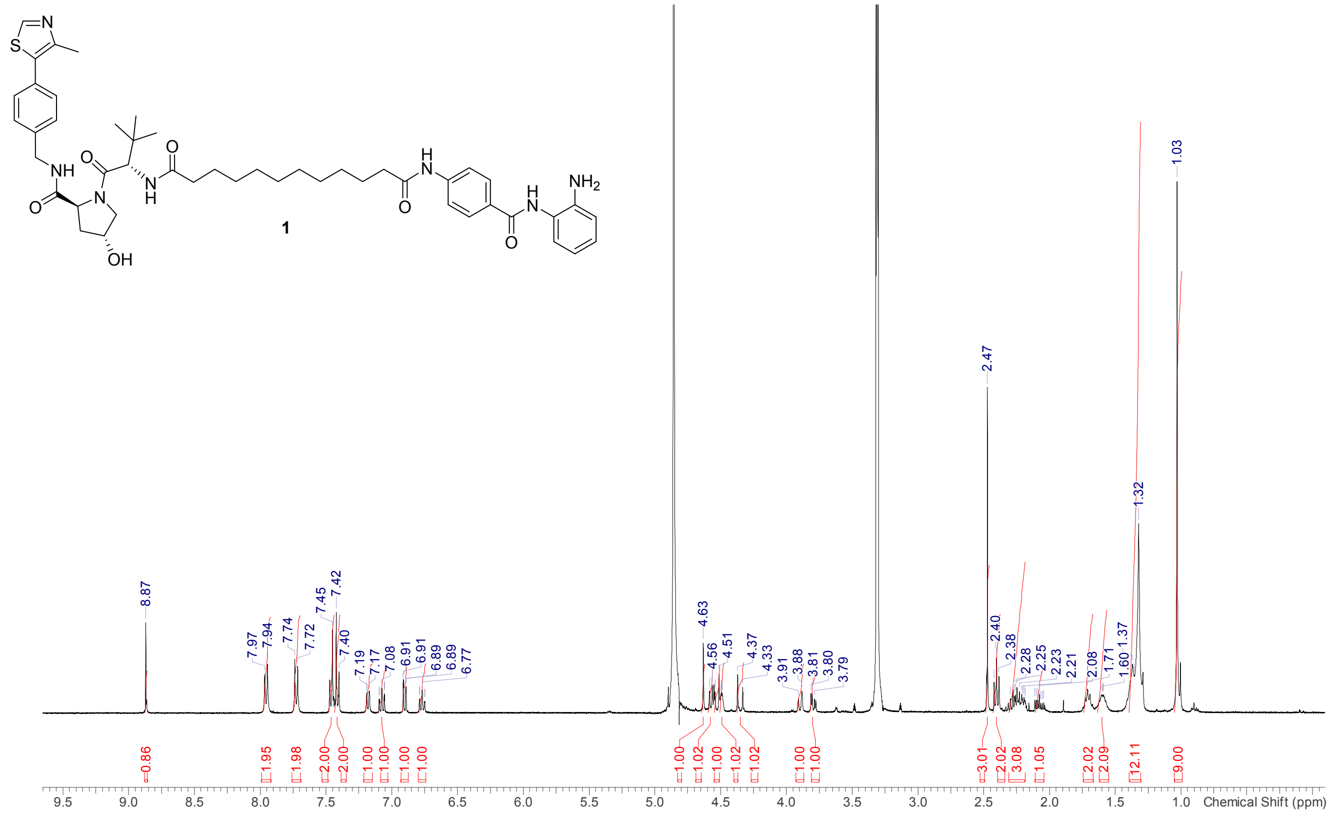

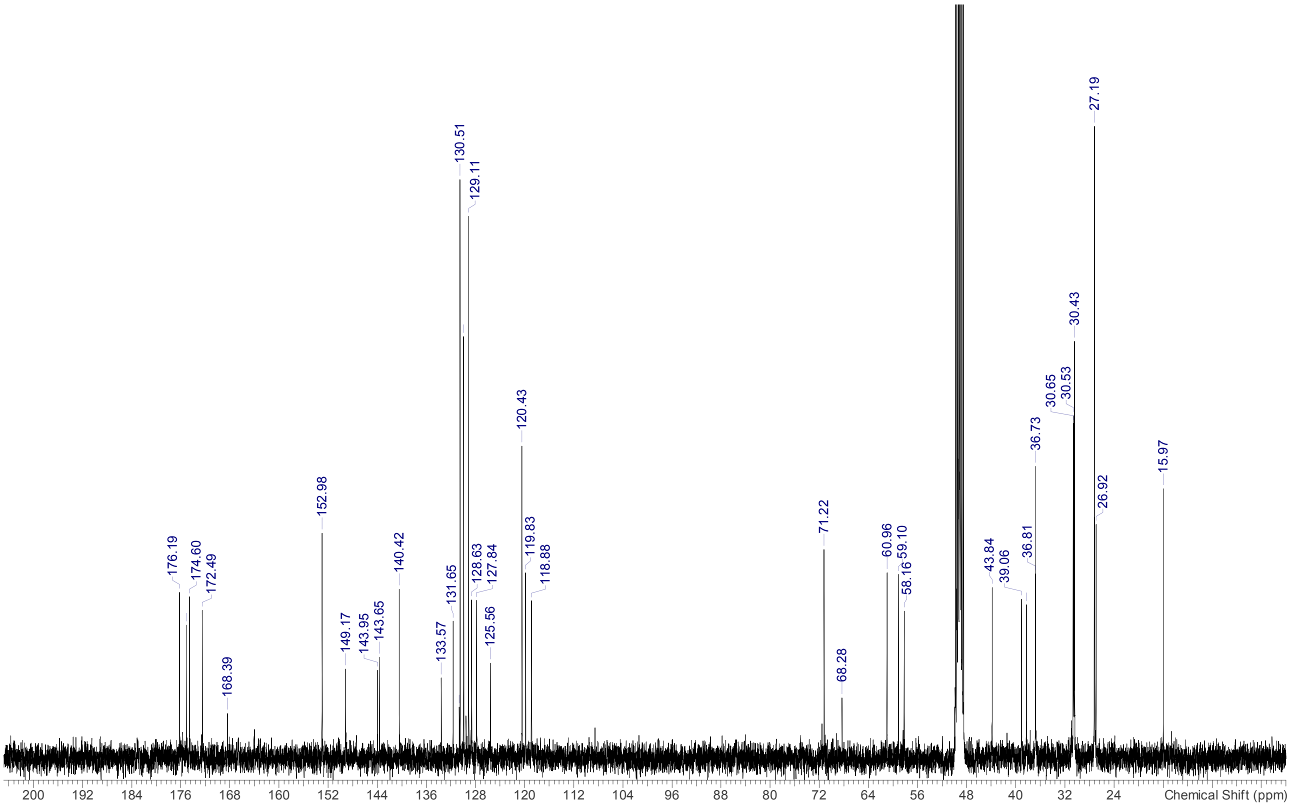

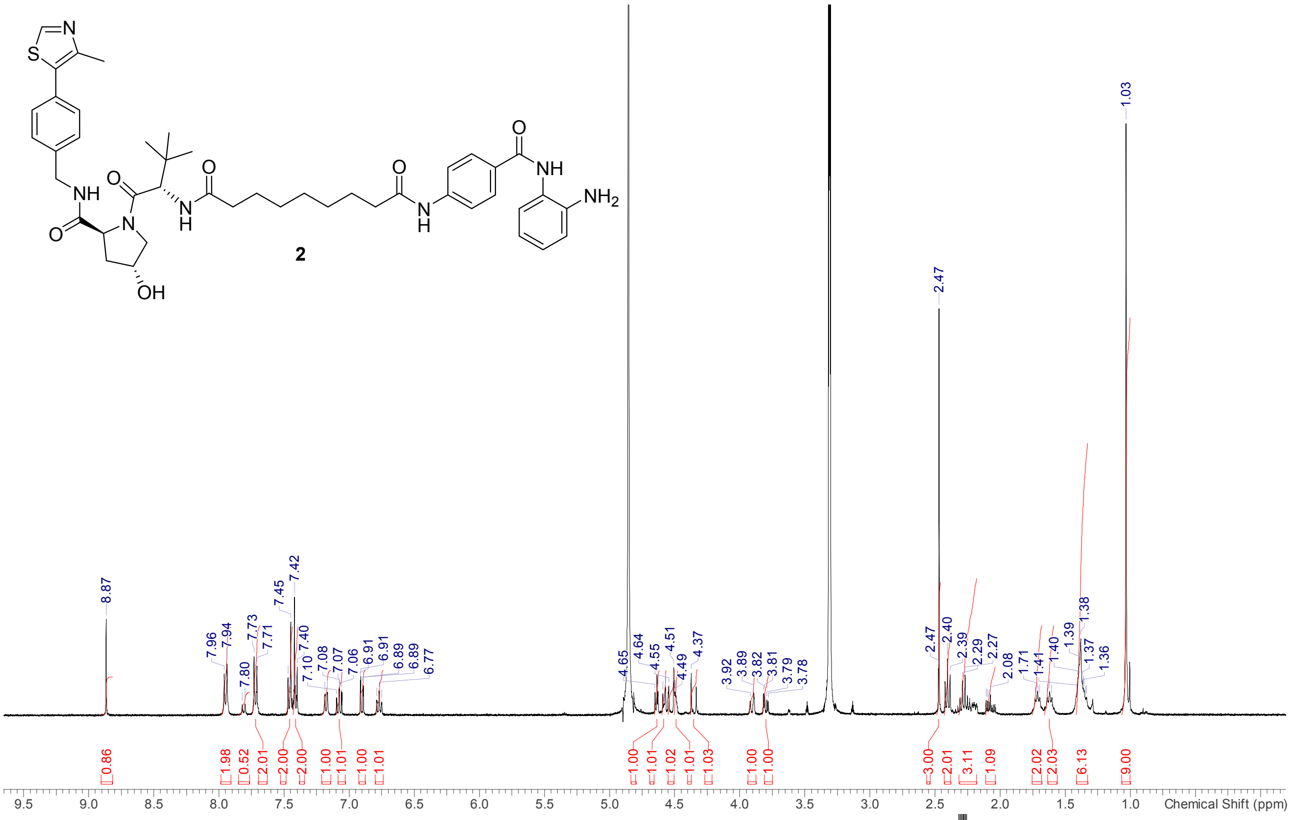
2
N
S
NH
O
O
N
H
OH
NH
NH2
8.87
7.96
7.94
7.80
7.73
7.71
7.45
7.42
7.40
7.10
7.08
7.07
7.06
6.91
6.91
6.89
6.89
6.77
4.65
4.64
4.55
4.51
4.49
4.37
3.92
3.89
3.82
3.81
3.79
3.78
2.47
2.47
2.40
2.39
2.29
2.27
2.08
1.71
1.41
1.40
1.39
1.38
1.37
1.36
1.03
0.86
1.98
0.52
2.01
2.00
2.00
1.00
1.01
1.00
1.01
1.00
1.01
1.02
1.01
1.03
1.00
1.00
3.00
2.01
3.11
1.09
2.02
2.03
6.13
9.00
Chemical Shift (ppm)

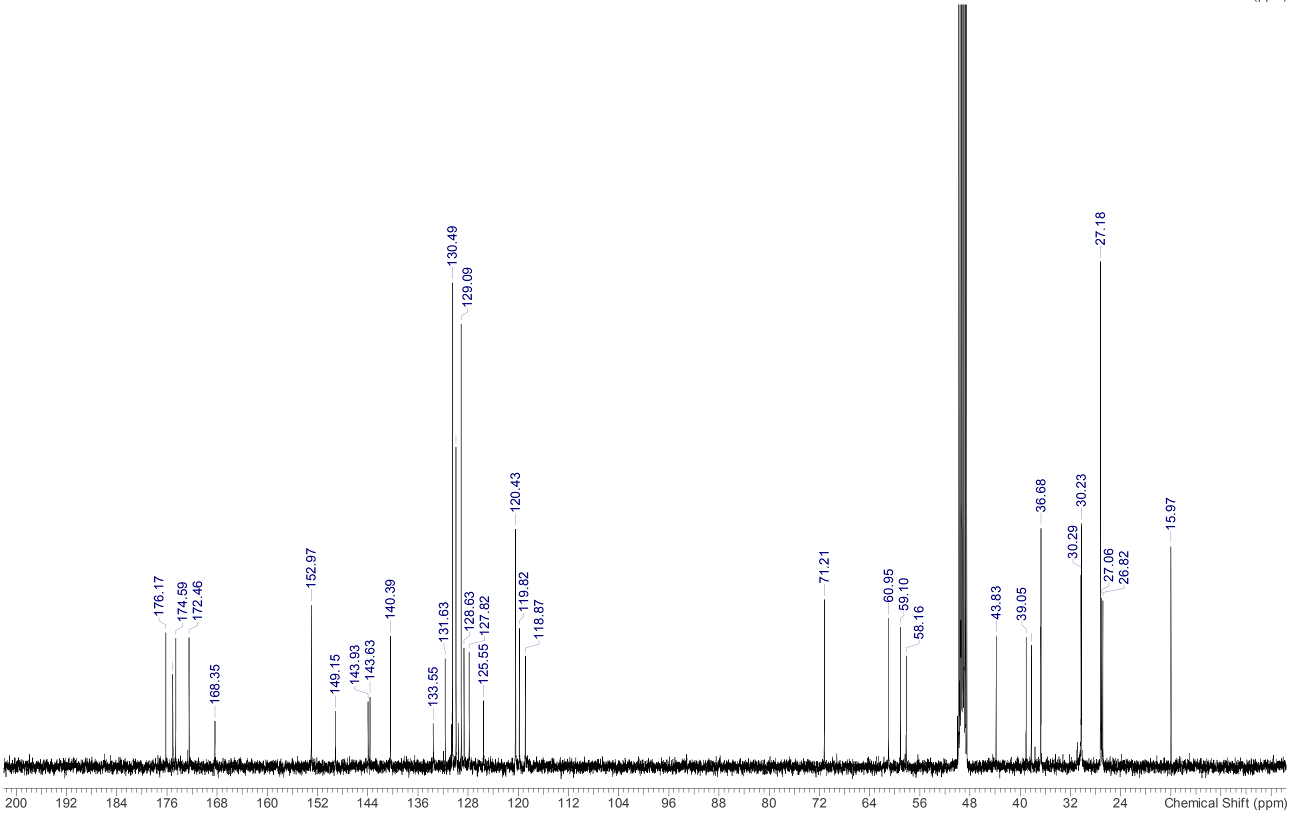
176.17
174.59
172.46
168.35
152.97
149.15
143.93
143.63
140.39
133.55
131.63
130.49
129.09
128.63
127.82
125.55
120.43
119.82
118.87
71.21
60.95
59.10
58.16
43.83
39.05
36.68
30.29
30.23
27.18
27.06
26.82
15.97
Chemical Shift (ppm)

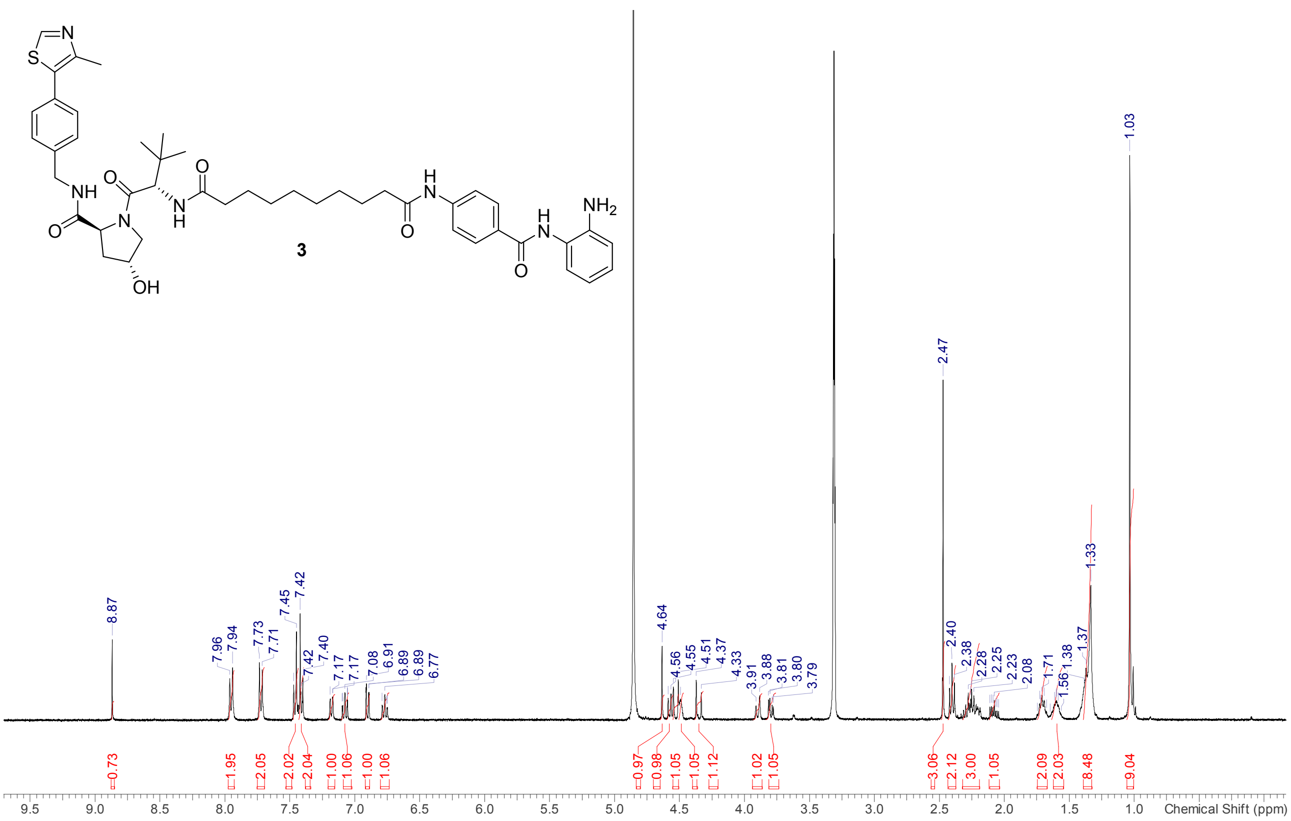

NH
O
N
H
N
O
O
H
N
O
NH$_2$
H
N
O
S
N
OH
3
8.87
7.96
7.94
7.73
7.71
7.45
7.42
7.42
7.40
7.17
7.17
7.08
6.91
6.89
6.89
6.77
4.64
4.56
4.55
4.51
4.37
4.33
3.91
3.88
3.81
3.80
3.79
2.47
2.40
2.38
2.28
2.25
2.23
2.08
1.71
1.56
1.38
1.37
1.33
1.03
0.73
1.95
2.05
2.02
2.04
1.00
1.06
1.00
1.06
0.97
0.98
1.05
1.05
1.12
1.02
1.05
3.06
2.12
3.00
1.05
2.09
2.03
8.48
9.04
9.5 9.0 8.5 8.0 7.5 7.0 6.5 6.0 5.5 5.0 4.5 4.0 3.5 3.0 2.5 2.0 1.5 1.0
Chemical Shift (ppm)

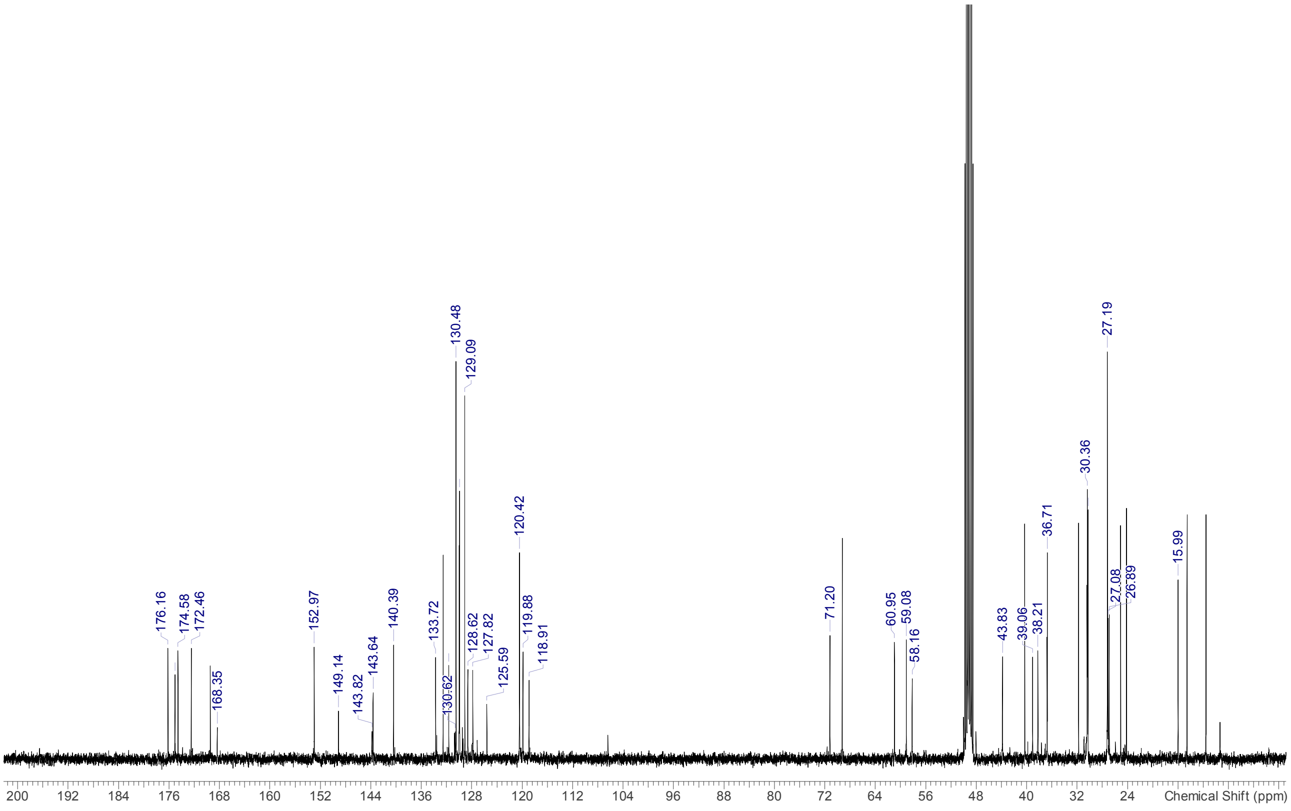

176.16
174.58
172.46
168.35
152.97
149.14
143.82
143.64
140.39
133.72
130.62
130.48
129.09
128.62
127.82
125.59
120.42
119.88
118.91
71.20
60.95
59.08
58.16
43.83
39.06
38.21
36.71
30.36
27.19
27.08
26.89
15.99
200 192 184 176 168 160 152 144 136 128 120 112 104 96 88 80 72 64 56 48 40 32 24
Chemical Shift (ppm)

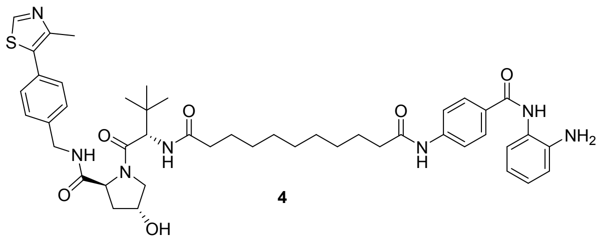
N
S
O
NH
N
H
NH2
OH

4

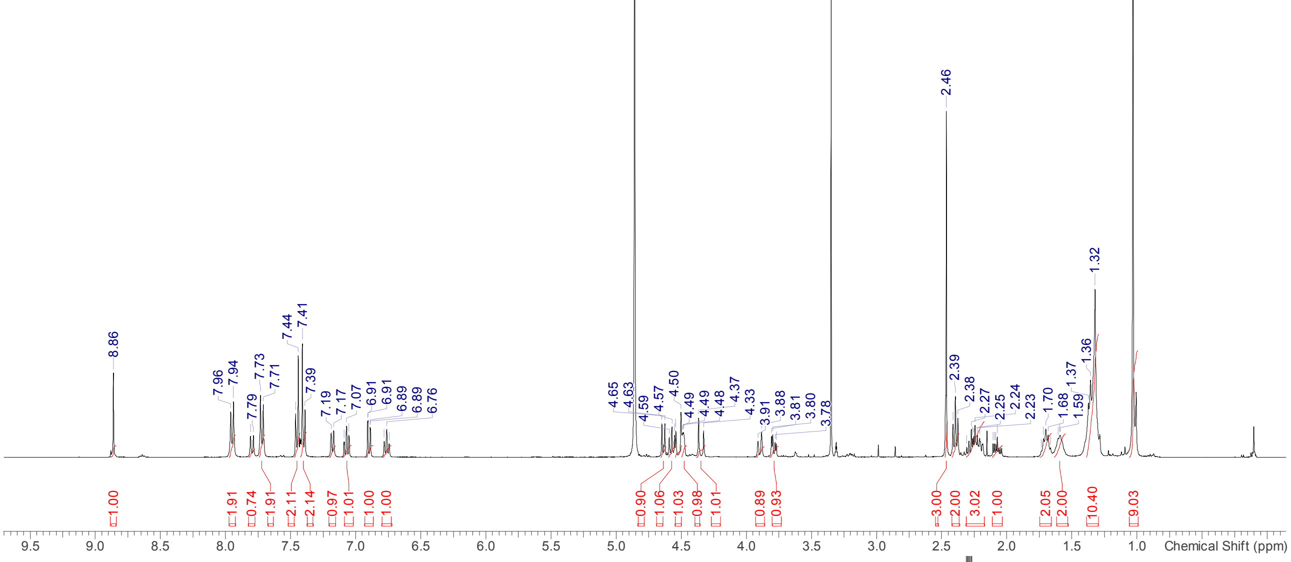
8.86
7.96
7.94
7.79
7.73
7.71
7.44
7.41
7.39
7.19
7.17
7.07
6.91
6.91
6.89
6.89
6.76
4.65
4.63
4.59
4.57
4.50
4.49
4.49
4.48
4.37
4.33
3.91
3.88
3.81
3.80
3.78
2.46
2.39
2.38
2.27
2.25
2.24
2.23
1.70
1.68
1.59
1.37
1.36
1.32
1.03
1.00
1.91
0.74
1.91
2.11
2.14
0.97
1.01
1.00
1.00
0.90
1.06
1.03
0.98
1.01
0.89
0.93
3.00
2.00
3.02
1.00
2.05
2.00
10.40
9.03
9.5
9.0
8.5
8.0
7.5
7.0
6.5
6.0
5.5
5.0
4.5
4.0
3.5
3.0
2.5
2.0
1.5
1.0
Chemical Shift (ppm)

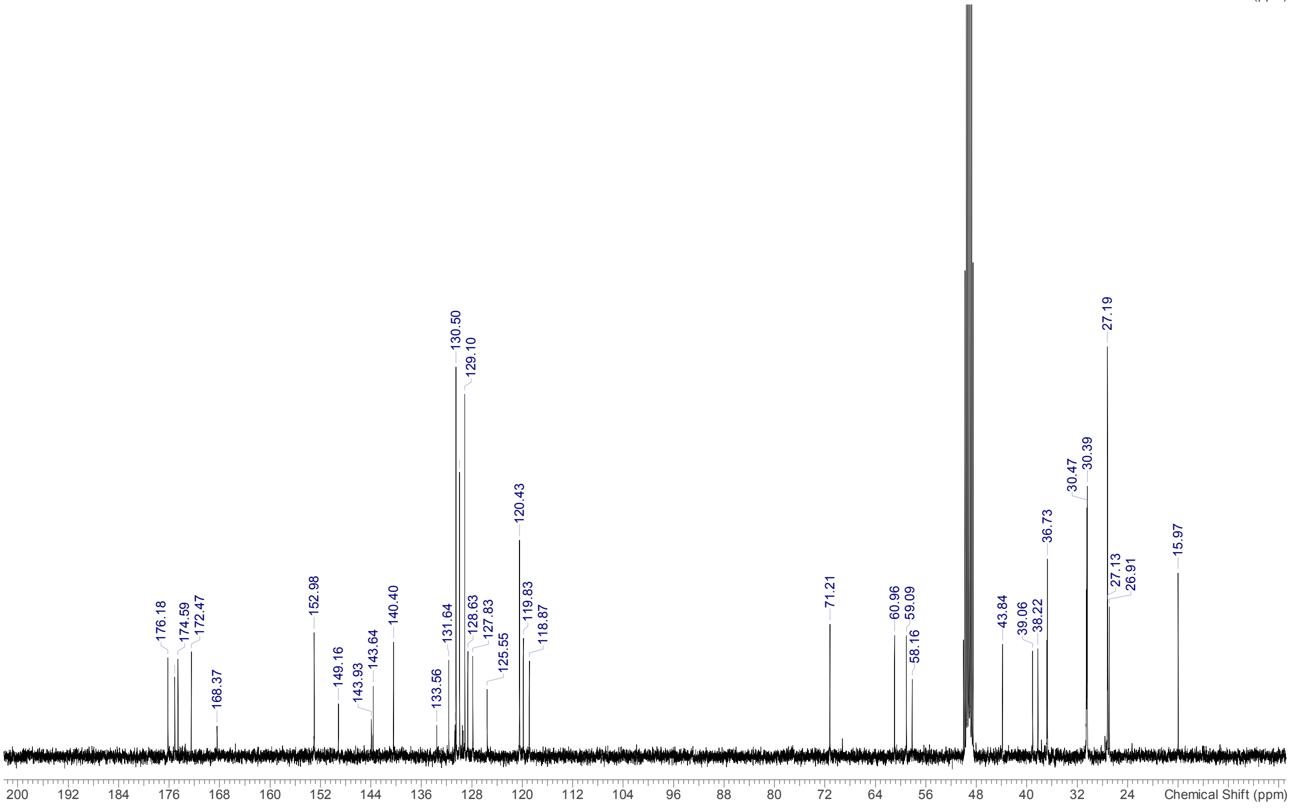
176.18
174.59
172.47
168.37
152.98
149.16
143.93
143.64
140.40
133.56
131.64
130.50
129.10
128.63
127.83
125.55
120.43
119.83
118.87
71.21
60.96
59.09
58.16
43.84
39.06
38.22
36.73
30.47
30.39
27.19
27.13
26.91
15.97
200
192
184
176
168
160
152
144
136
128
120
112
104
96
88
80
72
64
56
48
40
32
24
Chemical Shift (ppm)

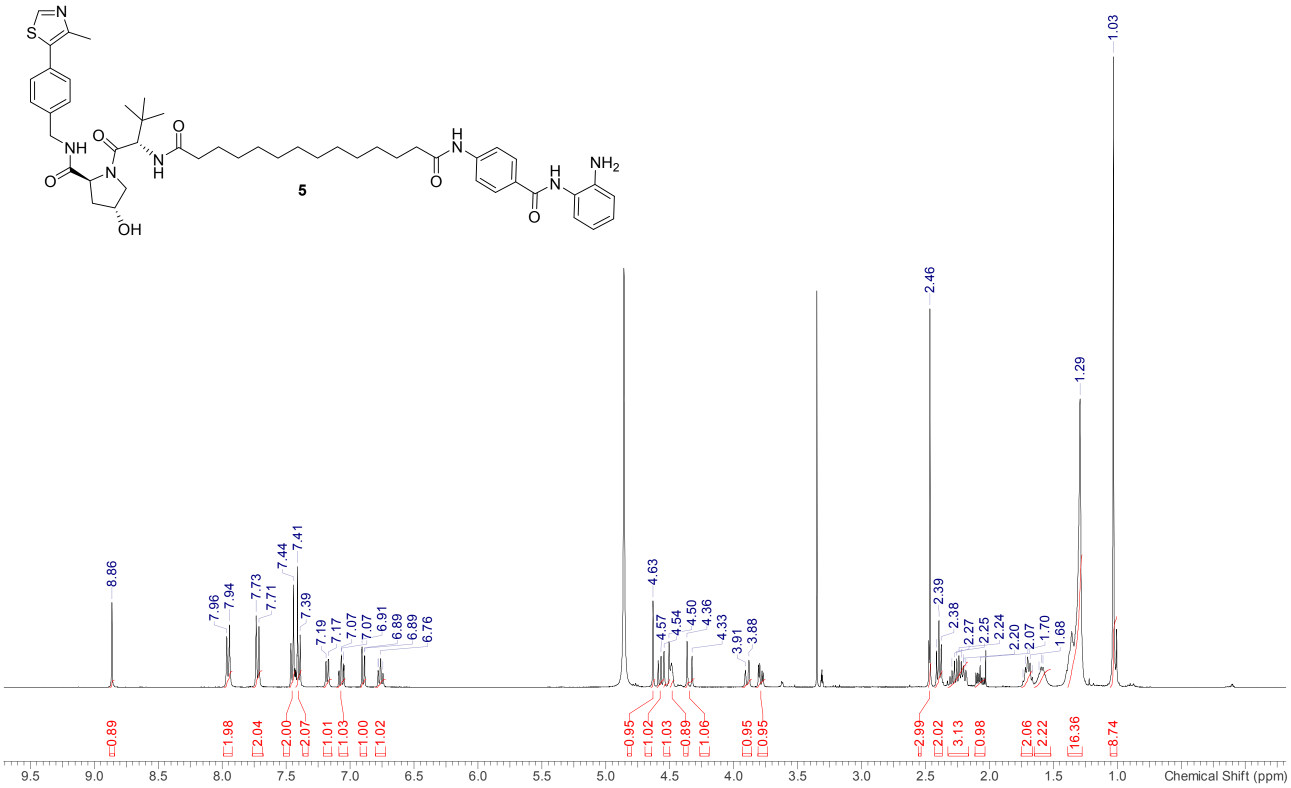

NH
O
N
O
H
N
O
OH
S
N
5
H
N
O
H
N
O
NH_2
8.86
7.96
7.94
7.73
7.71
7.44
7.41
7.39
7.19
7.17
7.07
7.07
6.91
6.89
6.89
6.76
4.63
4.57
4.54
4.50
4.36
4.33
3.91
3.88
2.46
2.39
2.38
2.27
2.25
2.24
2.20
2.07
1.70
1.68
1.29
1.03
0.89
1.98
2.04
2.00
2.07
1.01
1.03
1.00
1.02
0.95
1.02
1.03
0.89
1.06
0.95
0.95
2.99
2.02
3.13
0.98
2.06
2.22
16.36
8.74
9.5 9.0 8.5 8.0 7.5 7.0 6.5 6.0 5.5 5.0 4.5 4.0 3.5 3.0 2.5 2.0 1.5 1.0
Chemical Shift (ppm)

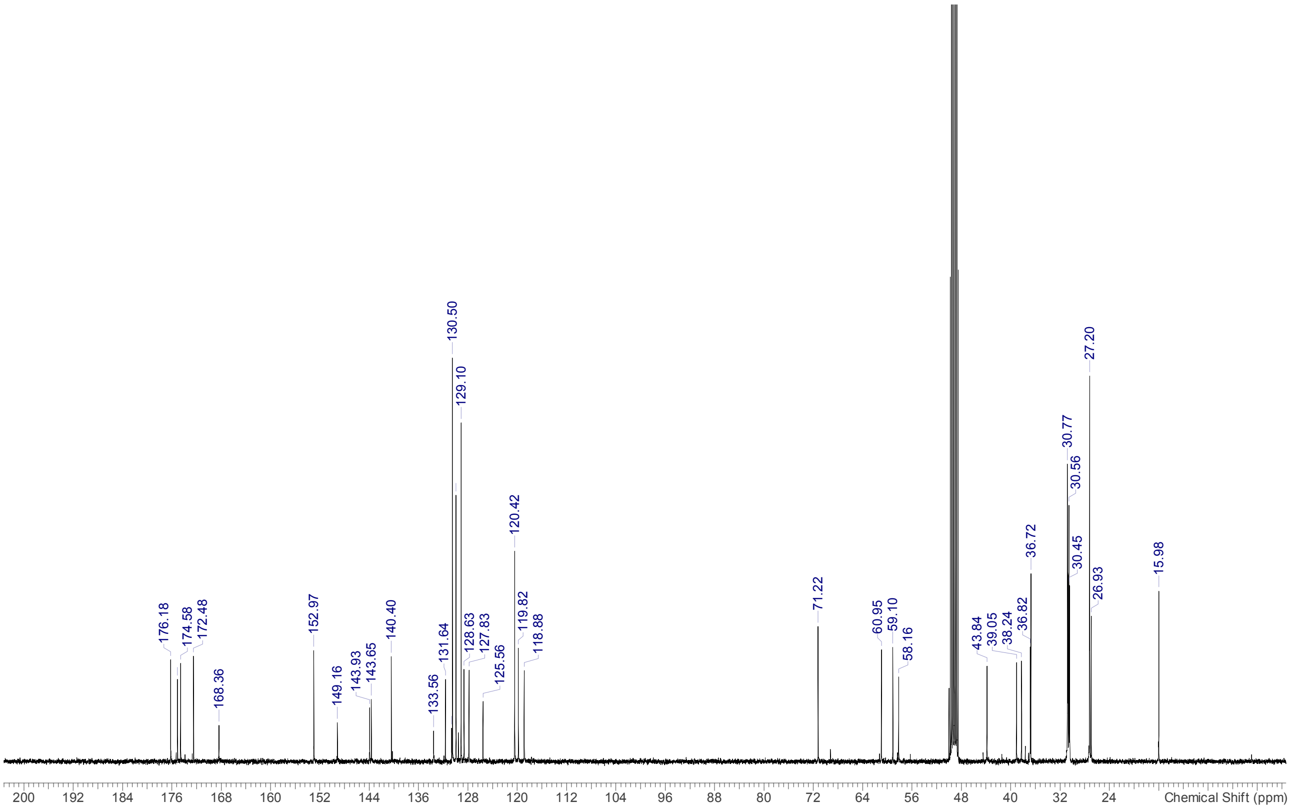

176.18
174.58
172.48
168.36
152.97
149.16
143.93
143.65
140.40
133.56
131.64
130.50
129.10
128.63
127.83
125.56
120.42
119.82
118.88
71.22
60.95
59.10
58.16
43.84
39.05
38.24
36.82
36.72
30.77
30.56
30.45
27.20
26.93
15.98
200 192 184 176 168 160 152 144 136 128 120 112 104 96 88 80 72 64 56 48 40 32 24
Chemical Shift (ppm)

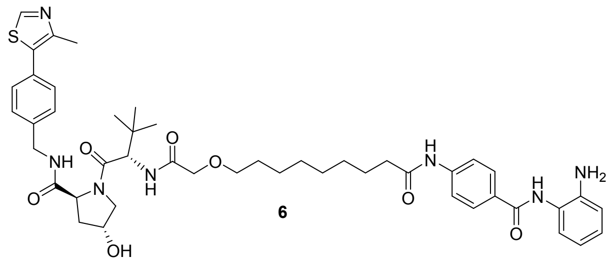

S
N
NH
O
O
N
H
N
O
O
OH
H
N
O
H
N
NH2
O

6

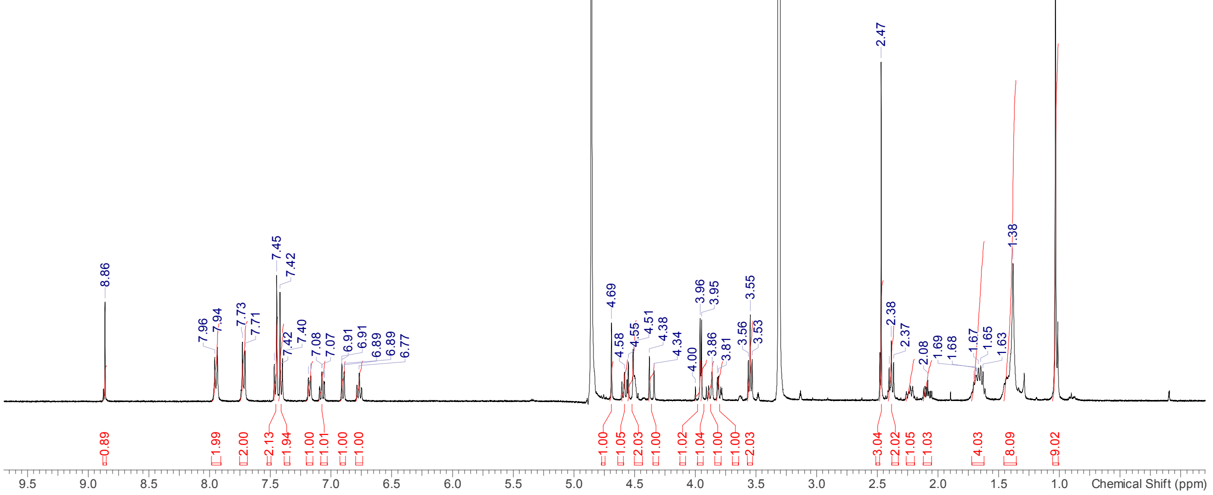

8.86
7.96
7.94
7.73
7.71
7.45
7.42
7.42
7.40
7.08
7.07
6.91
6.91
6.89
6.89
6.77
4.69
4.58
4.55
4.51
4.38
4.34
4.00
3.96
3.95
3.86
3.81
3.56
3.55
3.53
2.47
2.38
2.37
2.08
1.69
1.68
1.67
1.65
1.63
1.38
1.03
0.89
1.99
2.00
2.13
1.94
1.00
1.01
1.00
1.00
1.00
1.05
2.03
1.00
1.02
1.04
1.00
1.00
2.03
3.04
2.02
1.05
1.03
4.03
8.09
9.02
Chemical Shift (ppm)

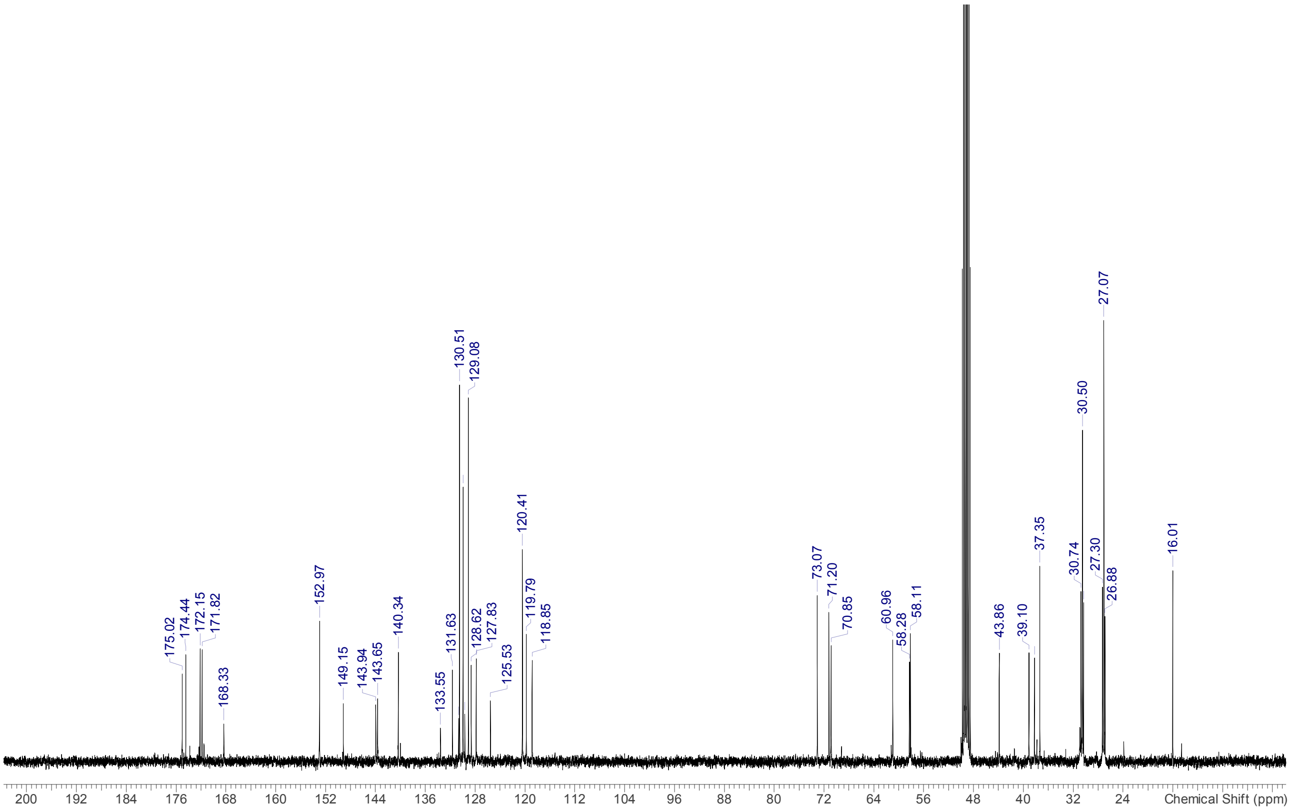

175.02
174.44
172.15
171.82
168.33
152.97
149.15
143.94
143.65
140.34
133.55
131.63
130.51
129.08
128.62
127.83
125.53
120.41
119.79
118.85
73.07
71.20
70.85
60.96
58.28
58.11
43.86
39.10
37.35
30.74
30.50
27.30
27.07
26.88
16.01
Chemical Shift (ppm)

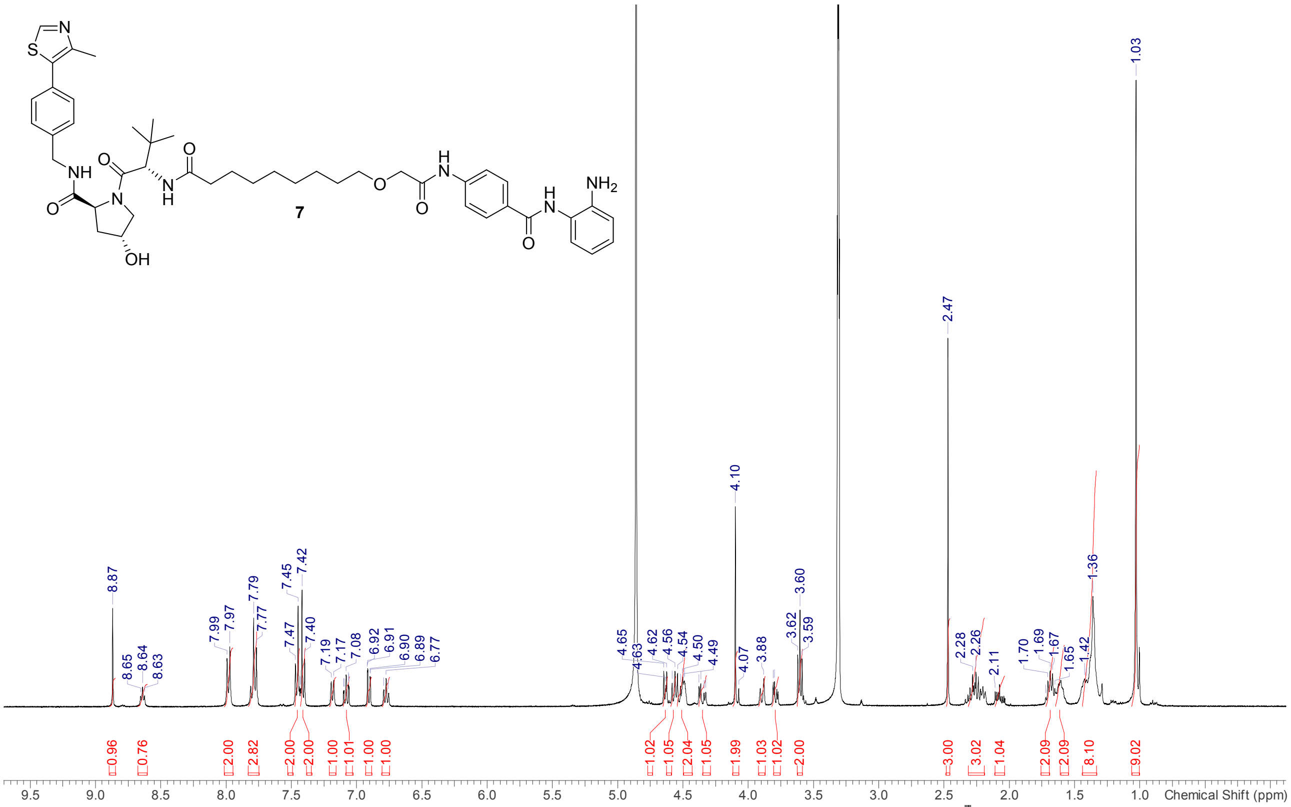

S
N
NH
O
N
OH
O
H
N
O
7
O
H
N
O
H
N
NH2
O
8.87
8.65
8.64
8.63
7.99
7.97
7.79
7.77
7.47
7.45
7.42
7.40
7.19
7.17
7.08
6.92
6.91
6.90
6.89
6.77
4.65
4.63
4.62
4.56
4.54
4.50
4.49
4.10
4.07
3.88
3.62
3.60
3.59
2.47
2.28
2.26
2.11
1.70
1.69
1.67
1.65
1.42
1.36
1.03
0.96
0.76
2.00
2.82
2.00
2.00
1.00
1.01
1.00
1.00
1.02
1.05
2.04
1.05
1.99
1.03
1.02
2.00
3.00
3.02
1.04
2.09
2.09
8.10
9.02
9.5
9.0
8.5
8.0
7.5
7.0
6.5
6.0
5.5
5.0
4.5
4.0
3.5
3.0
2.5
2.0
1.5
1.0
Chemical Shift (ppm)

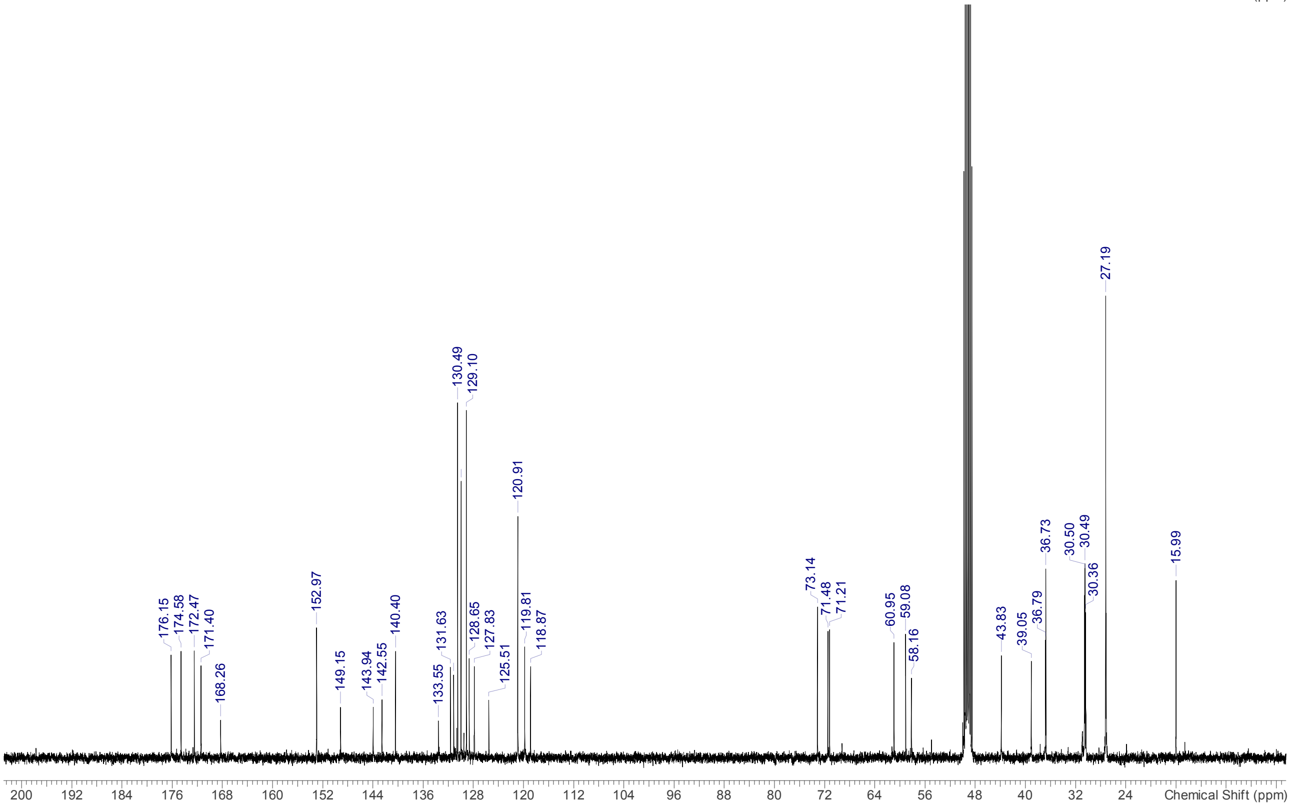

176.15
174.58
172.47
171.40
168.26
152.97
149.15
143.94
142.55
140.40
133.55
131.63
130.49
129.10
128.65
127.83
125.51
120.91
119.81
118.87
73.14
71.48
71.21
60.95
59.08
58.16
43.83
39.05
36.79
36.73
30.50
30.49
30.36
27.19
15.99
200
192
184
176
168
160
152
144
136
128
120
112
104
96
88
80
72
64
56
48
40
32
24
Chemical Shift (ppm)

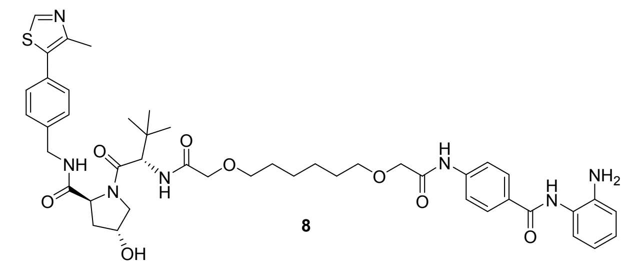
N
S
NH
O
N
OH
O
H
N
O
O
O
H
N
O
H
N
NH_2
O

8

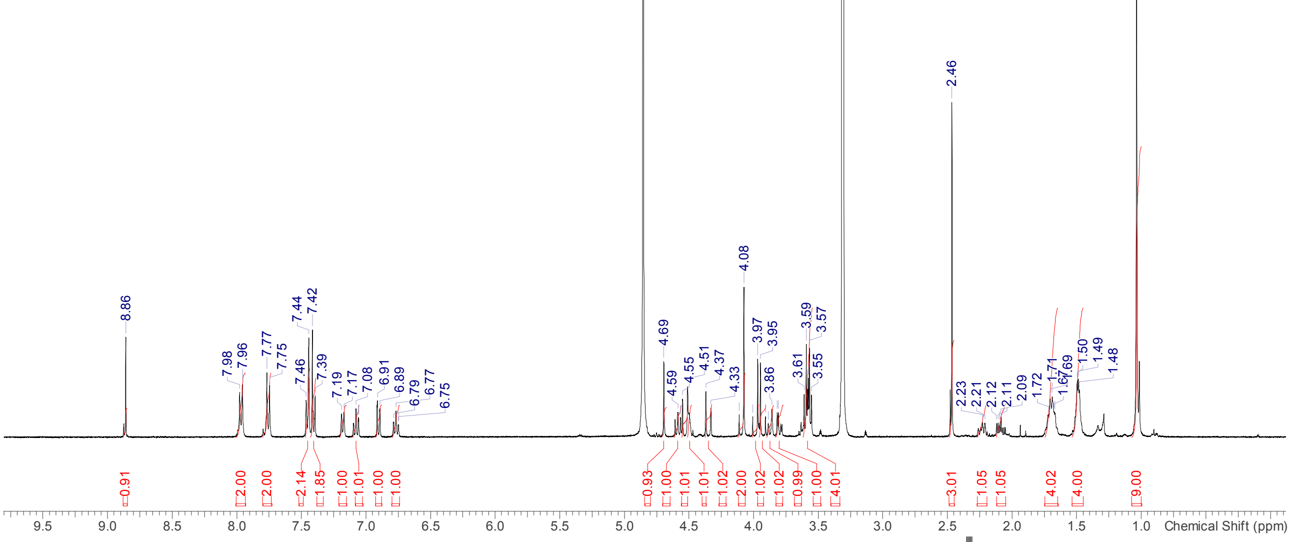
8.86
7.98
7.96
7.77
7.75
7.46
7.44
7.42
7.39
7.19
7.17
7.08
6.91
6.89
6.79
6.77
6.75
4.69
4.59
4.55
4.51
4.37
4.33
4.08
3.97
3.95
3.86
3.61
3.59
3.57
3.55
2.46
2.23
2.21
2.12
2.11
2.09
1.72
1.71
1.69
1.67
1.50
1.49
1.48
1.04
0.91
2.00
2.00
2.14
1.85
1.00
1.01
1.00
1.00
0.93
1.00
1.01
1.01
1.02
2.00
1.02
1.02
0.99
1.00
4.01
3.01
1.05
1.05
4.02
4.00
9.00
9.5
9.0
8.5
8.0
7.5
7.0
6.5
6.0
5.5
5.0
4.5
4.0
3.5
3.0
2.5
2.0
1.5
1.0
Chemical Shift (ppm)

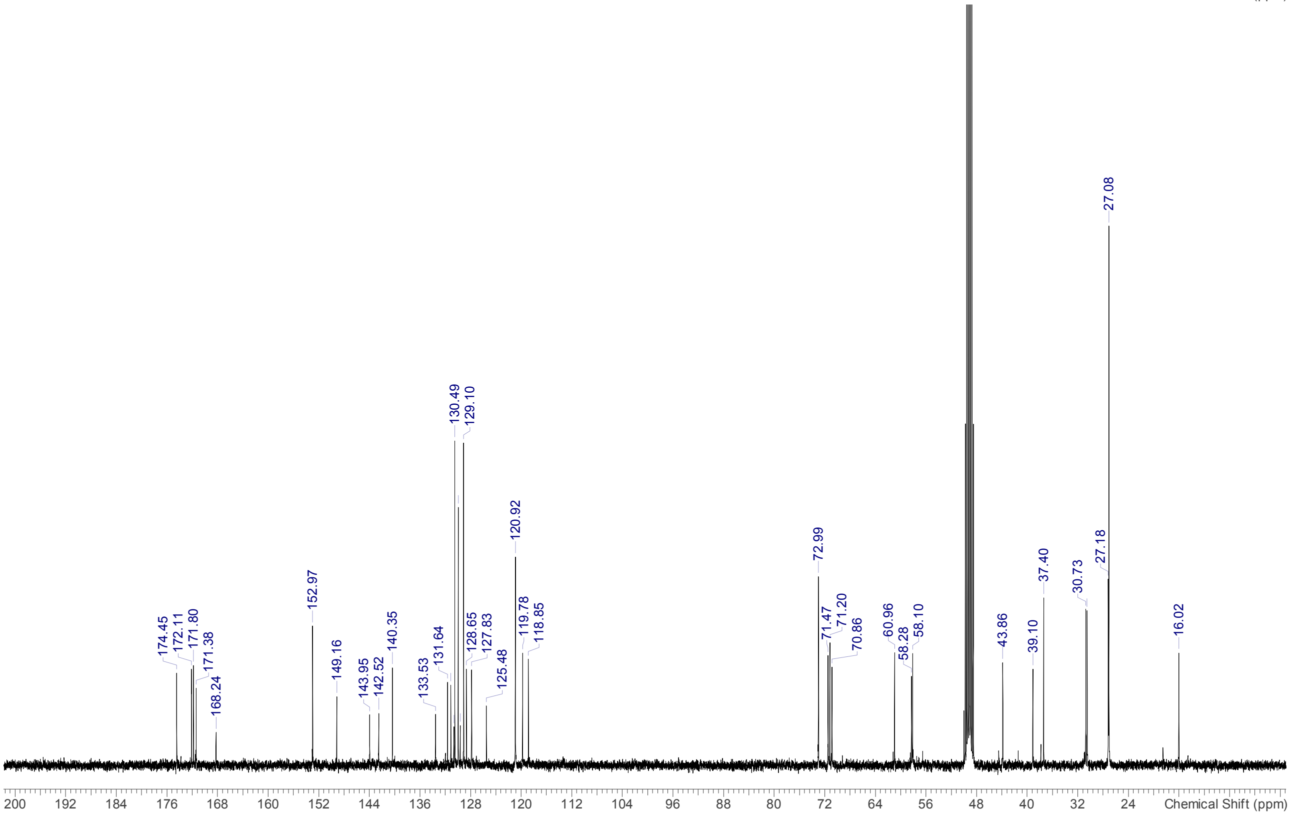
174.45
172.11
171.80
171.38
168.24
152.97
149.16
143.95
142.52
140.35
133.53
131.64
130.49
129.10
128.65
127.83
125.48
120.92
119.78
118.85
72.99
71.47
71.20
70.86
60.96
58.28
58.10
43.86
39.10
37.40
30.73
27.18
27.08
16.02
200
192
184
176
168
160
152
144
136
128
120
112
104
96
88
80
72
64
56
48
40
32
24
Chemical Shift (ppm)

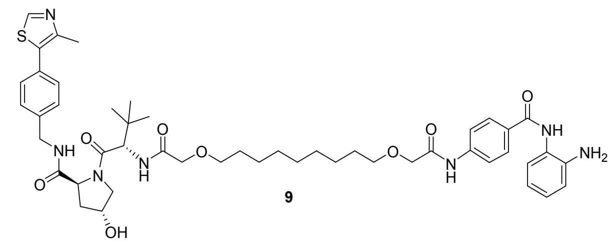
S
N
NH
O
N
O
OH
N
H
O
O
O
O
N
H
O
NH
NH2

9

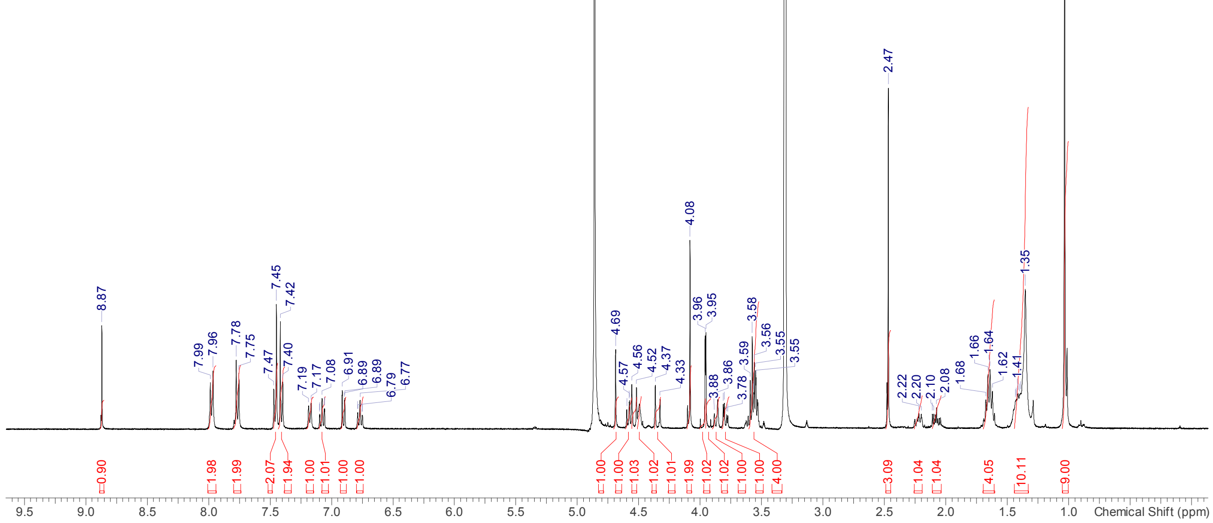
8.87
7.99
7.96
7.78
7.75
7.47
7.45
7.42
7.40
7.19
7.17
7.08
6.91
6.89
6.89
6.79
6.77
4.69
4.57
4.56
4.52
4.37
4.33
4.08
3.96
3.95
3.88
3.86
3.78
3.59
3.58
3.56
3.55
3.55
2.47
2.22
2.20
2.10
2.08
1.68
1.66
1.64
1.62
1.41
1.35
1.03
0.90
1.98
1.99
2.07
1.94
1.00
1.01
1.00
1.00
1.00
1.00
1.03
1.02
1.01
1.99
1.02
1.02
1.00
1.00
4.00
3.09
1.04
1.04
4.05
10.11
9.00
9.5 9.0 8.5 8.0 7.5 7.0 6.5 6.0 5.5 5.0 4.5 4.0 3.5 3.0 2.5 2.0 1.5 1.0
Chemical Shift (ppm)

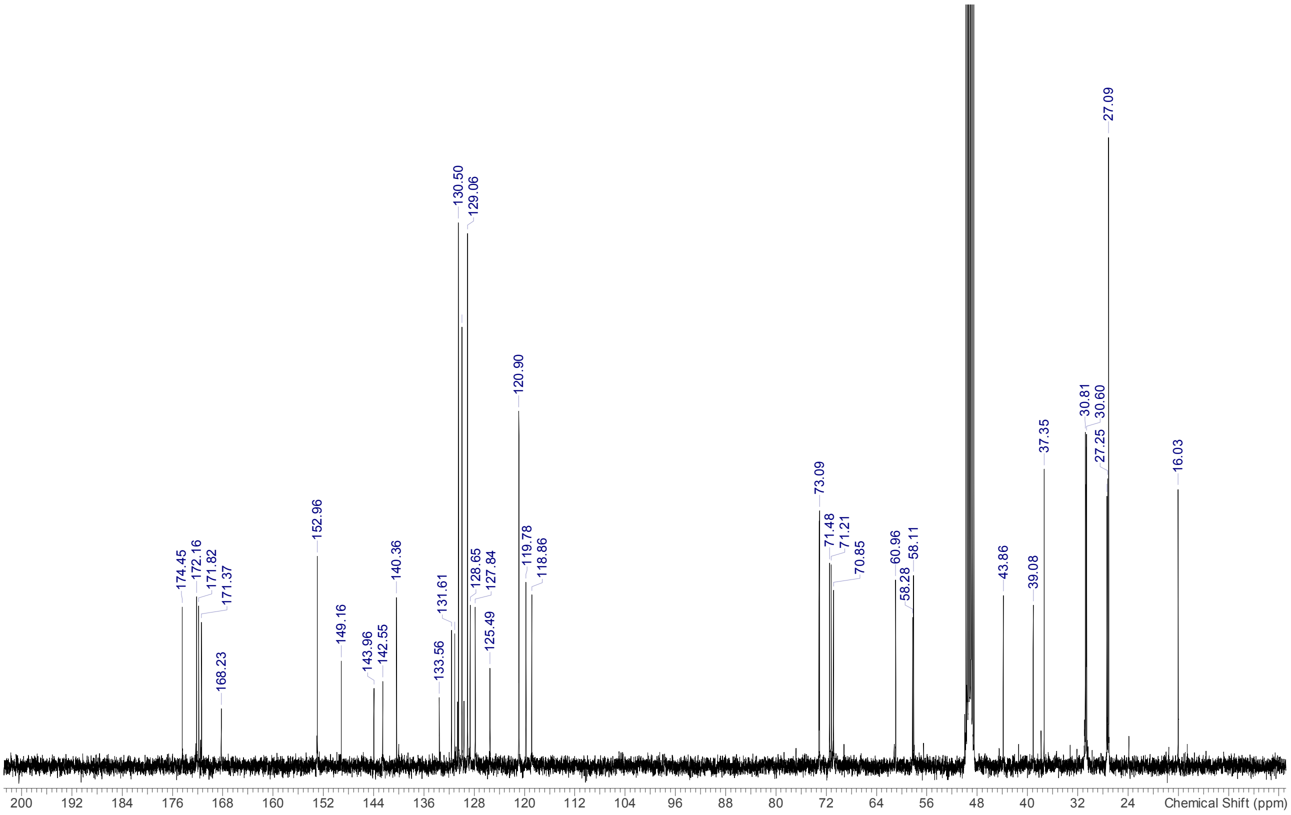
174.45
172.16
171.82
171.37
168.23
152.96
149.16
143.96
142.55
140.36
133.56
131.61
130.50
129.06
128.65
127.84
125.49
120.90
119.78
118.86
73.09
71.48
71.21
70.85
60.96
58.28
58.11
43.86
39.08
37.35
30.81
30.60
27.25
27.09
16.03
200 192 184 176 168 160 152 144 136 128 120 112 104 96 88 80 72 64 56 48 40 32 24
Chemical Shift (ppm)

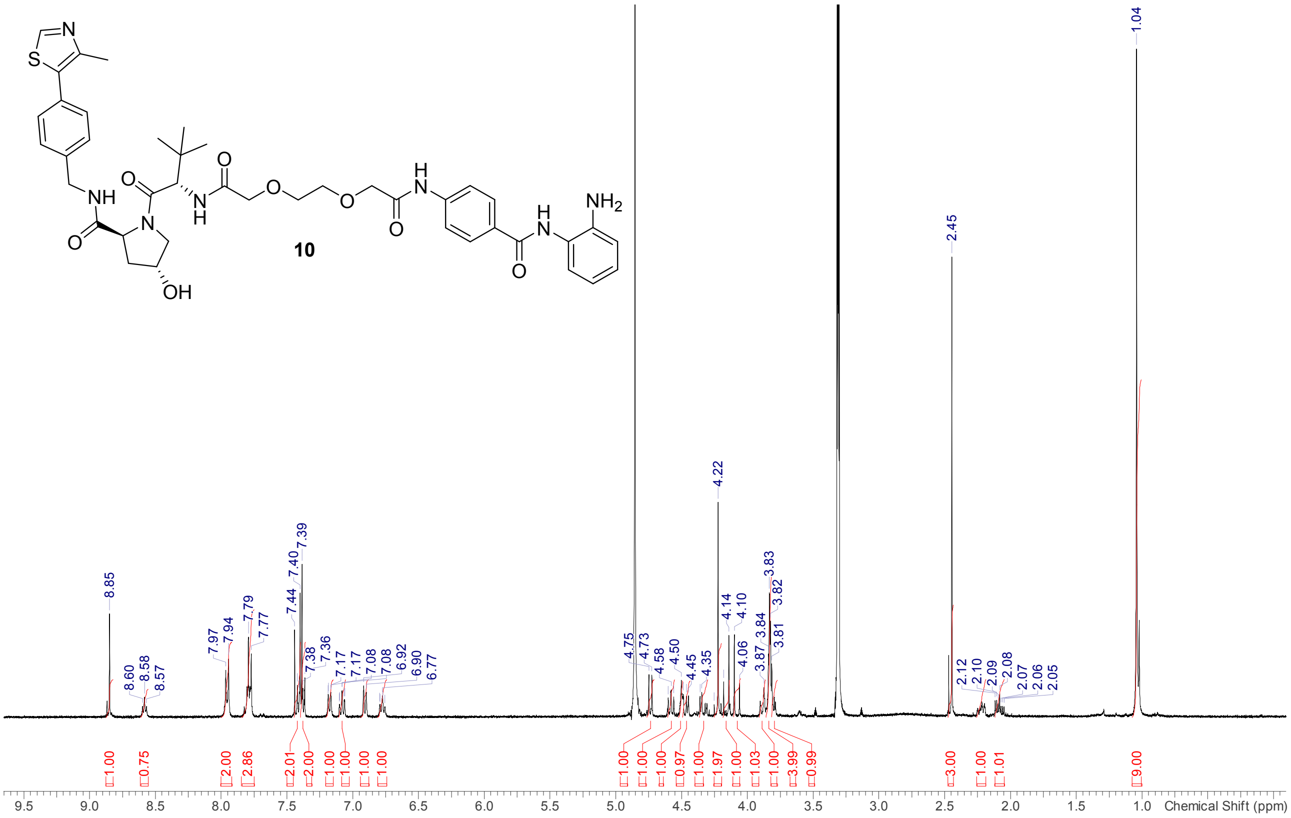
S
N
NH
O
O
N
OH
N
H
O
O
O
H
N
O
H
N
O
NH2
8.85
8.60
8.58
8.57
7.97
7.94
7.79
7.77
7.44
7.40
7.39
7.38
7.36
7.17
7.17
7.08
7.08
6.92
6.90
6.77
4.75
4.73
4.58
4.50
4.45
4.35
4.22
4.14
4.10
4.06
3.87
3.84
3.83
3.82
3.81
2.45
2.12
2.10
2.09
2.08
2.07
2.06
2.05
1.04
1.00
0.75
2.00
2.86
2.01
2.00
1.00
1.00
1.00
1.00
1.00
1.00
1.00
0.97
1.00
1.97
1.00
1.03
1.00
3.99
0.99
3.00
1.00
1.01
9.00
9.5 9.0 8.5 8.0 7.5 7.0 6.5 6.0 5.5 5.0 4.5 4.0 3.5 3.0 2.5 2.0 1.5 1.0
Chemical Shift (ppm)

10

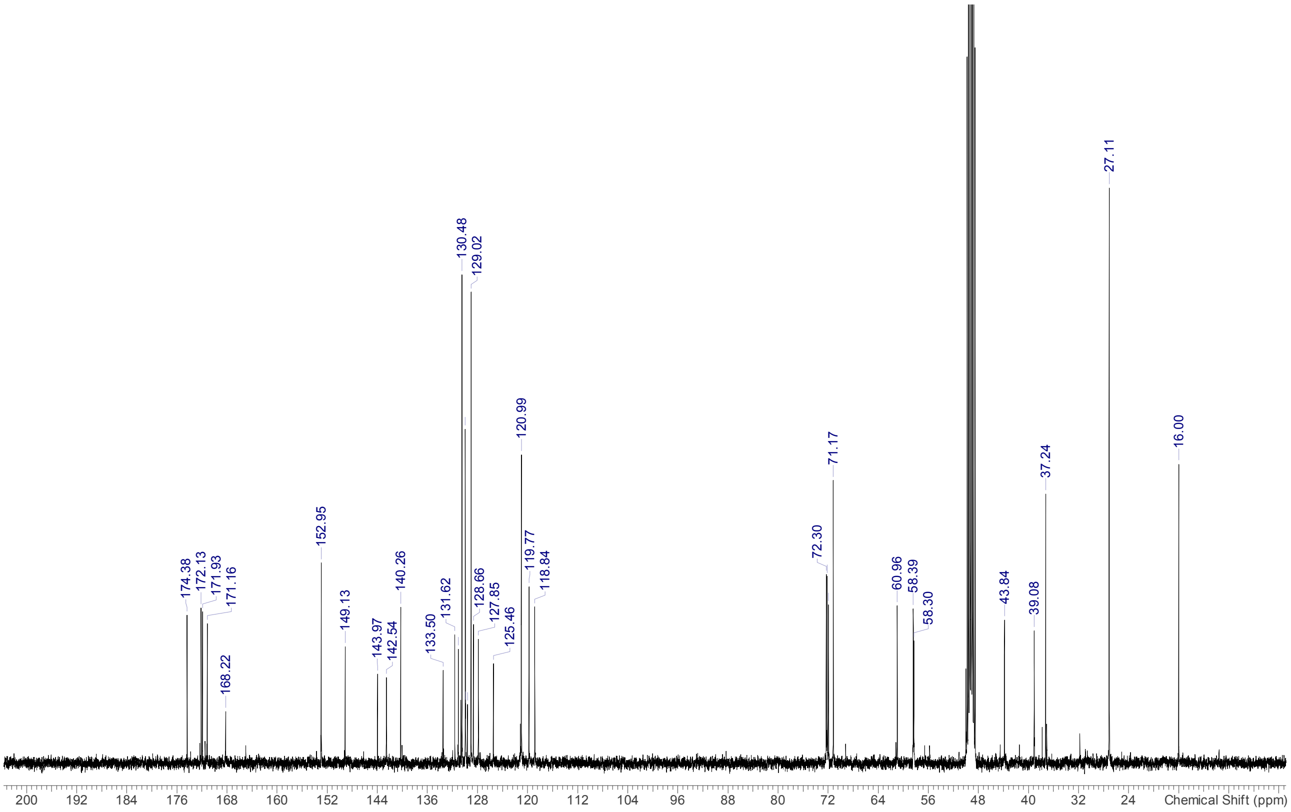
174.38
172.13
171.93
171.16
168.22
152.95
149.13
143.97
142.54
140.26
133.50
131.62
130.48
129.02
128.66
127.85
125.46
120.99
119.77
118.84
72.30
71.17
60.96
58.39
58.30
43.84
39.08
37.24
27.11
16.00
200 192 184 176 168 160 152 144 136 128 120 112 104 96 88 80 72 64 56 48 40 32 24
Chemical Shift (ppm)

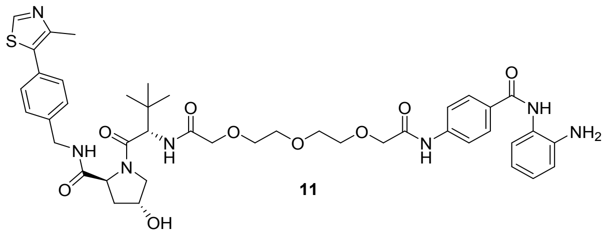
S
N
NH
O
N
OH
O
H
N
O
O
O
O
O
H
N
O
NH
NH2

11

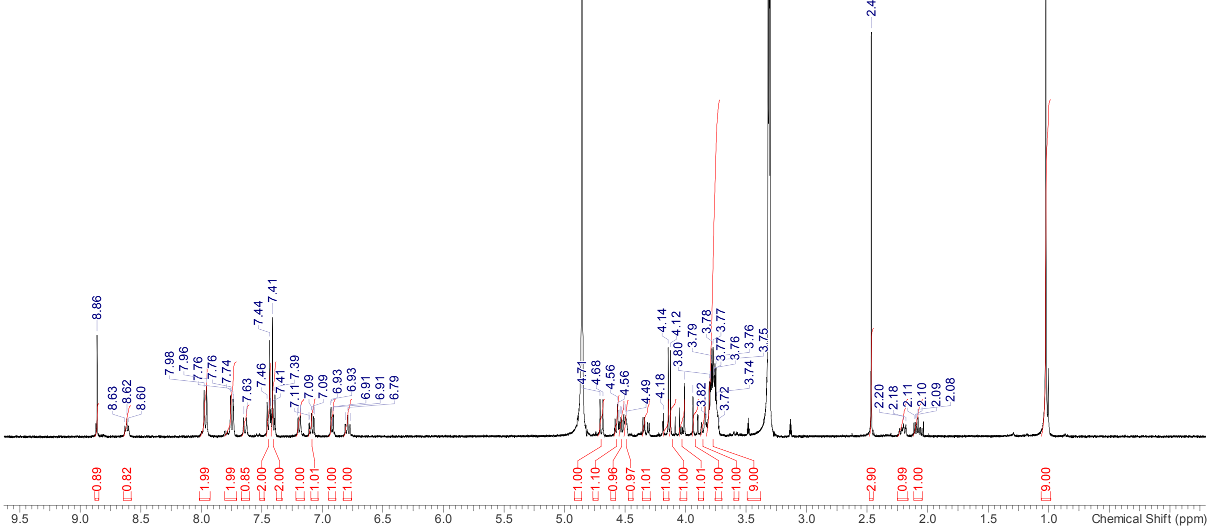
8.86
8.63
8.62
8.60
7.98
7.96
7.76
7.76
7.74
7.63
7.46
7.44
7.41
7.41
7.39
7.11
7.09
7.09
6.93
6.93
6.91
6.91
6.79
4.71
4.68
4.56
4.56
4.49
4.18
4.14
4.12
3.80
3.79
3.78
3.77
3.77
3.76
3.76
3.75
3.74
3.82
3.72
2.47
2.20
2.18
2.11
2.10
2.09
2.08
1.02
0.89
0.82
1.99
1.99
0.85
2.00
2.00
1.00
1.01
1.00
1.00
1.00
1.10
0.96
0.97
1.01
1.00
1.00
1.01
1.00
1.00
9.00
2.90
0.99
1.00
9.00
9.5
9.0
8.5
8.0
7.5
7.0
6.5
6.0
5.5
5.0
4.5
4.0
3.5
3.0
2.5
2.0
1.5
1.0
Chemical Shift (ppm)

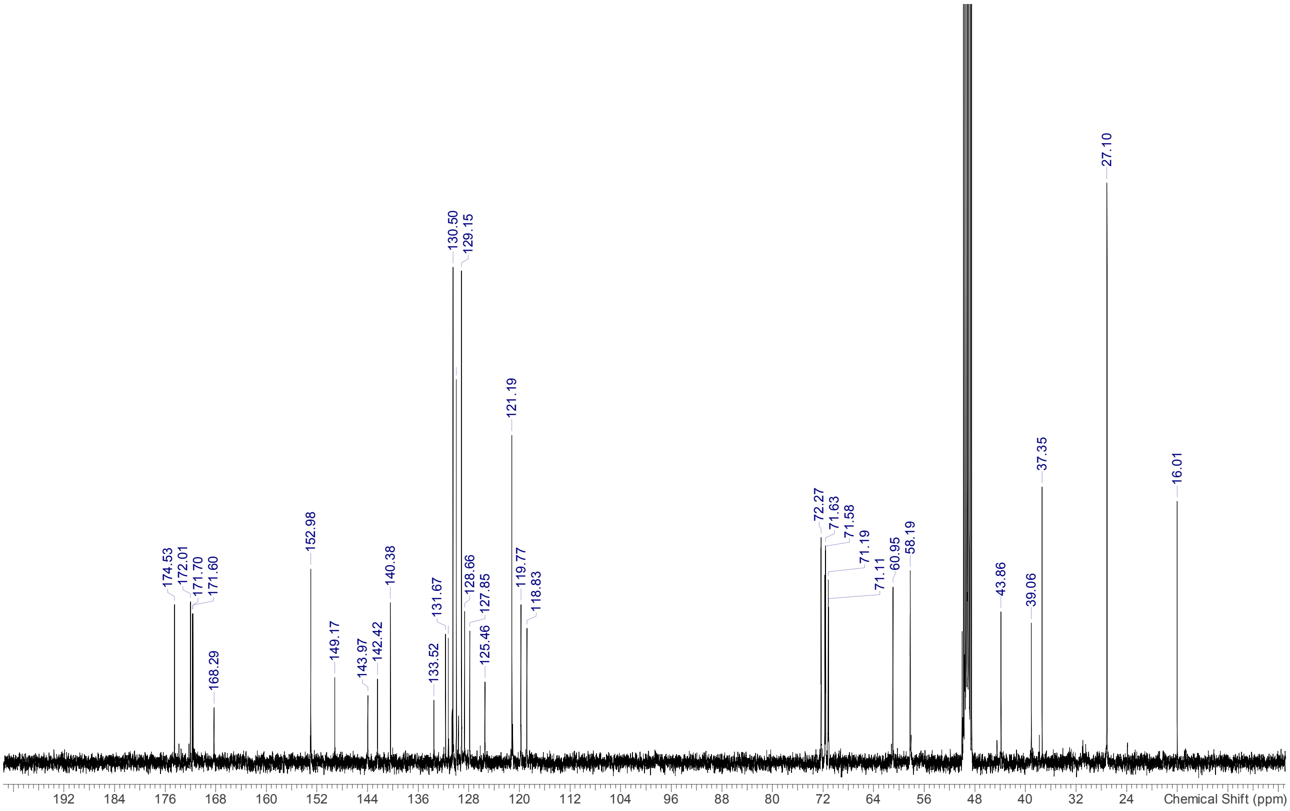
174.53
172.01
171.70
171.60
168.29
152.98
149.17
143.97
142.42
140.38
133.52
131.67
130.50
129.15
128.66
127.85
125.46
121.19
119.77
118.83
72.27
71.63
71.58
71.19
71.11
60.95
58.19
43.86
39.06
37.35
27.10
16.01
192
184
176
168
160
152
144
136
128
120
112
104
96
88
80
72
64
56
48
40
32
24
Chemical Shift (ppm)

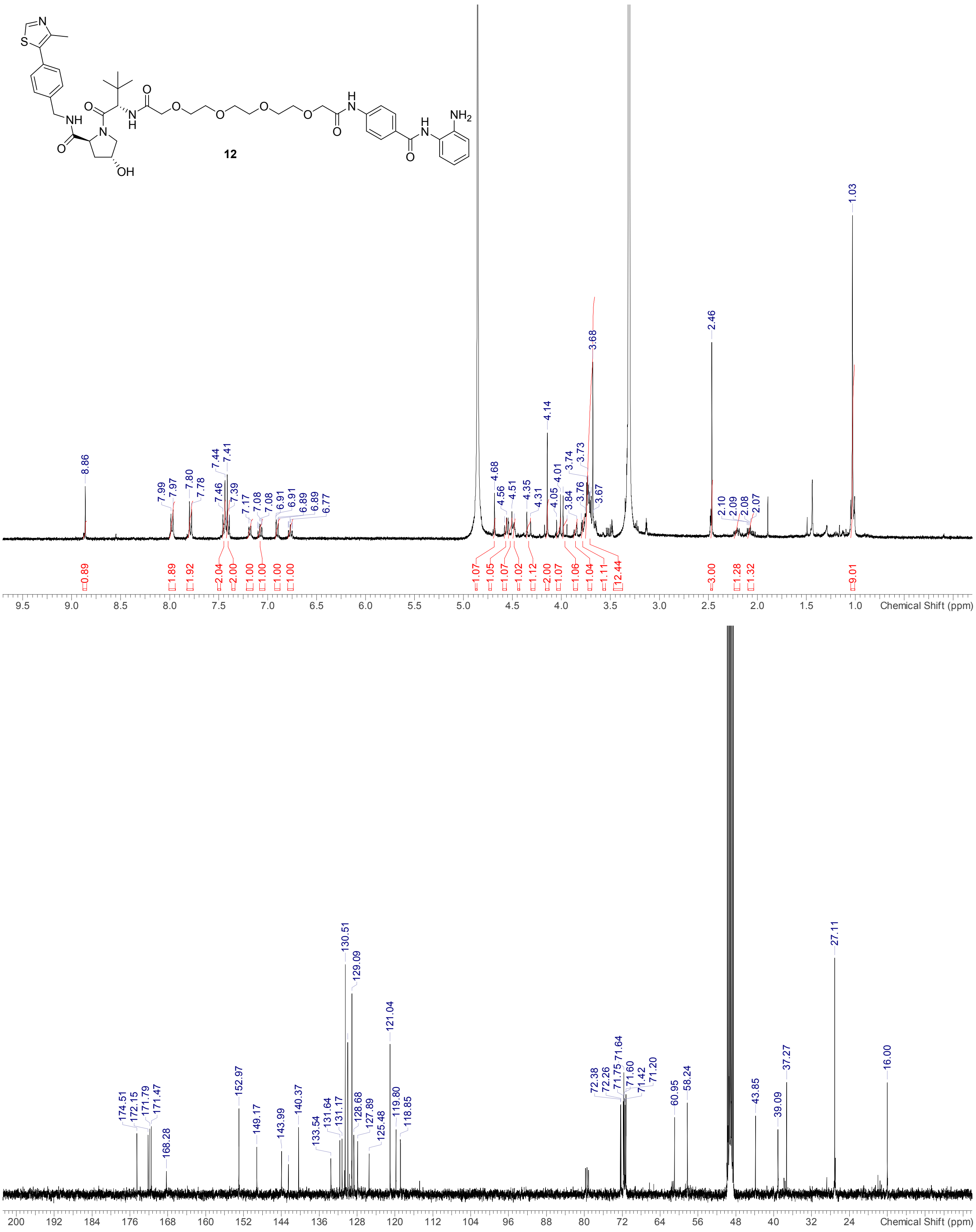

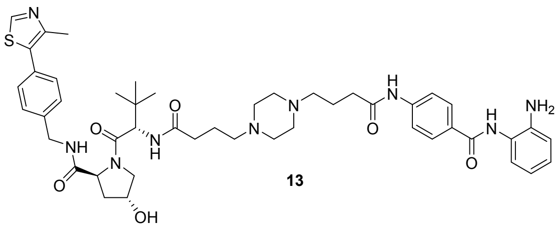
NH
O
N
OH
O
H
N
O
N
N
O
H
N
O
H
N
NH2
S
N

13

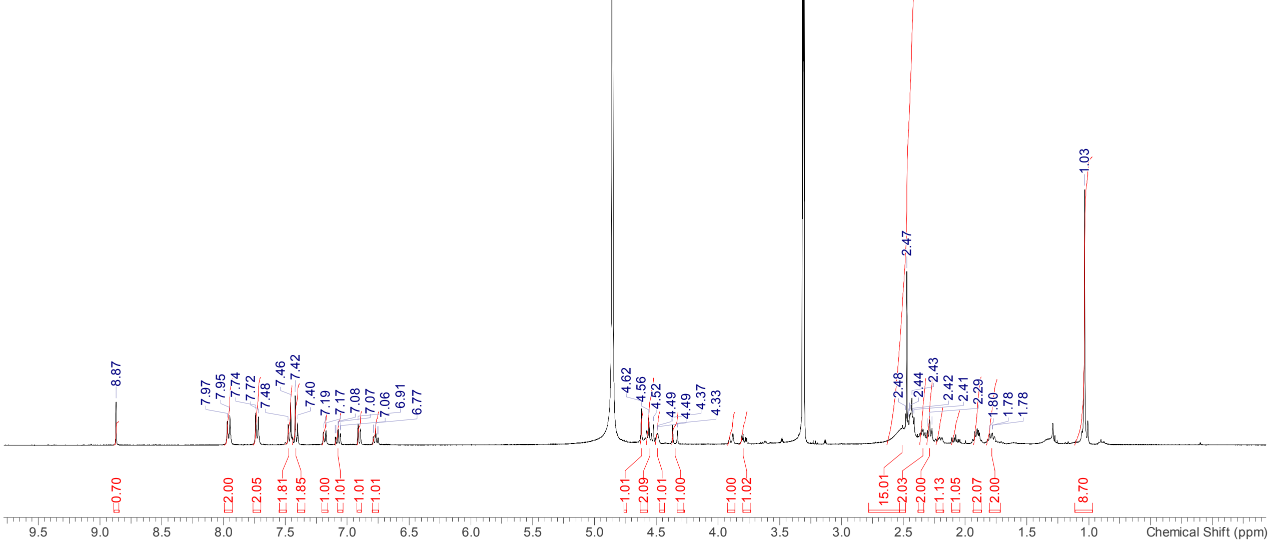
8.87
7.97
7.95
7.74
7.72
7.48
7.46
7.42
7.40
7.19
7.17
7.08
7.07
7.06
6.91
6.77
4.62
4.56
4.52
4.49
4.49
4.37
4.33
2.48
2.47
2.44
2.43
2.42
2.41
2.29
1.80
1.78
1.78
1.03
0.70
2.00
2.05
1.81
1.85
1.00
1.01
1.01
1.01
1.01
2.09
1.01
1.00
1.00
1.02
15.01
2.03
2.00
1.13
1.05
2.07
2.00
8.70
Chemical Shift (ppm)

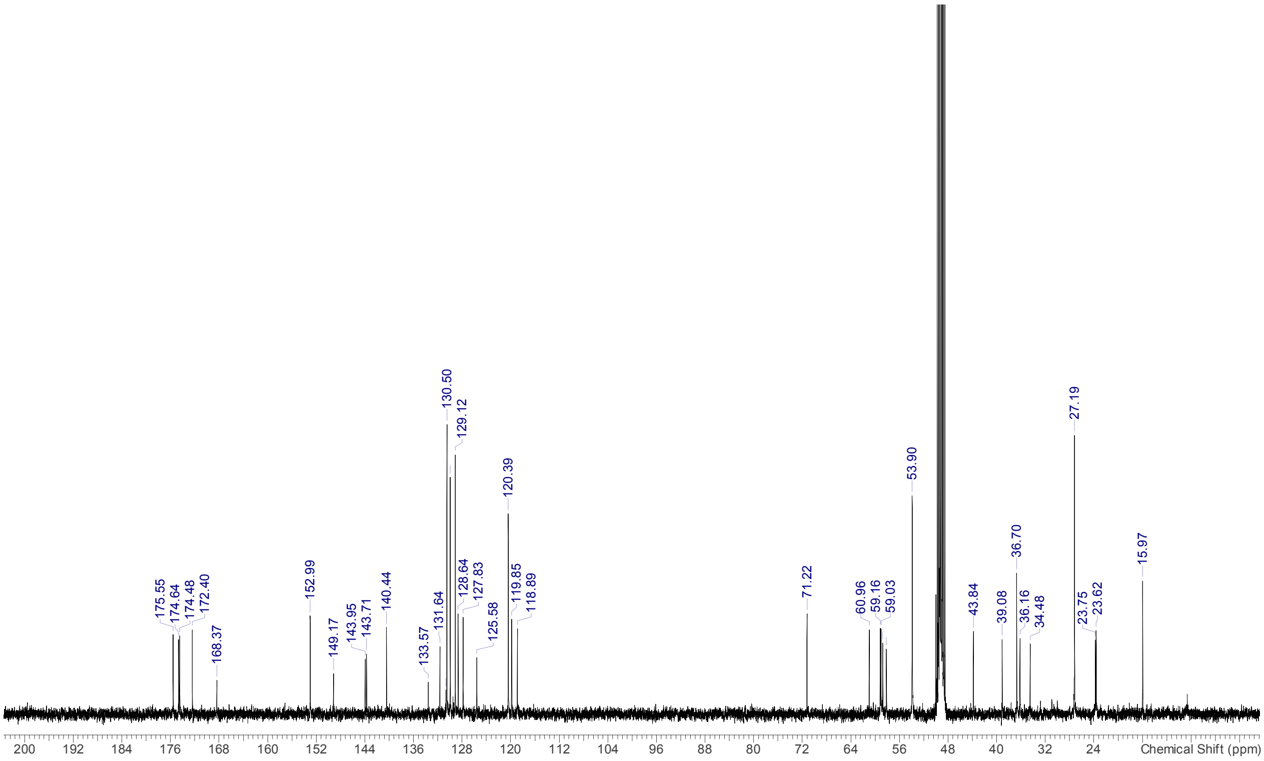
175.55
174.64
174.48
172.40
168.37
152.99
149.17
143.95
143.71
140.44
133.57
131.64
130.50
129.12
128.64
127.83
125.58
120.39
119.85
118.89
71.22
60.96
59.16
59.03
53.90
43.84
39.08
36.70
36.16
34.48
27.19
23.75
23.62
15.97
Chemical Shift (ppm)

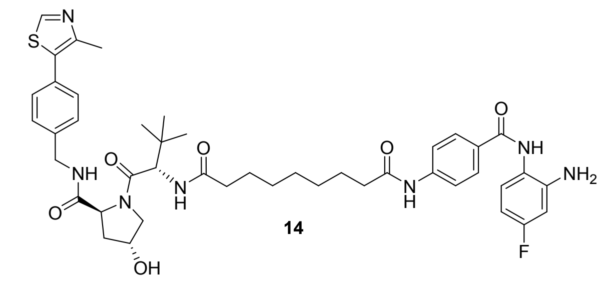
S
N
O
NH
O
N
OH
H
N
O
O
H
N
O
NH
NH2
F

14

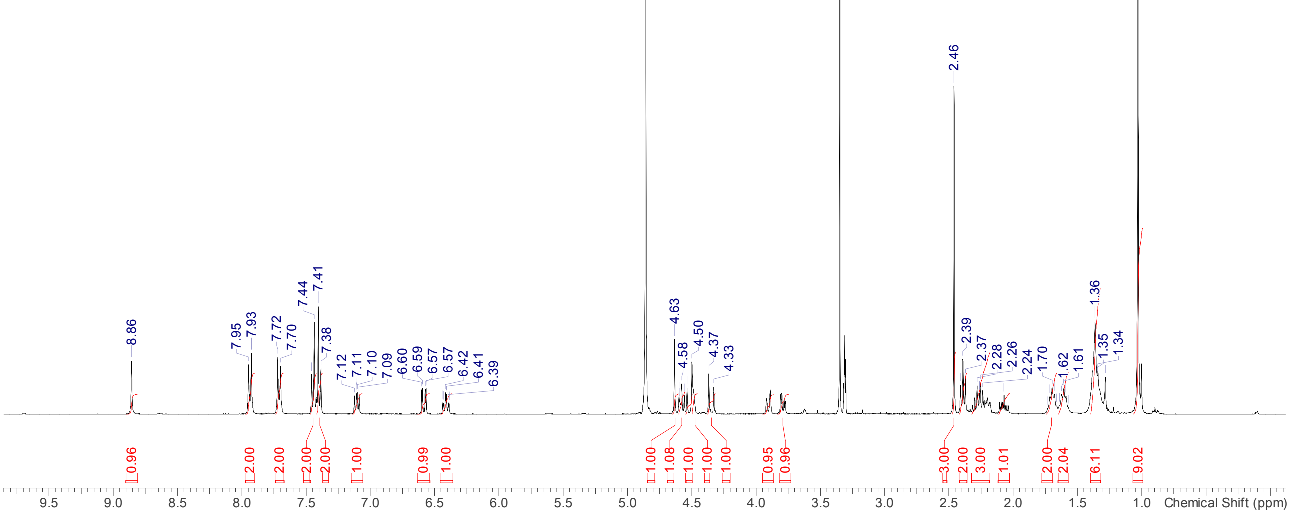
8.86
7.95
7.93
7.72
7.70
7.44
7.41
7.38
7.12
7.11
7.10
7.09
6.60
6.59
6.57
6.57
6.42
6.41
6.39
4.63
4.58
4.50
4.37
4.33
2.46
2.39
2.37
2.28
2.26
2.24
1.70
1.62
1.61
1.36
1.35
1.34
1.03
0.96
2.00
2.00
2.00
2.00
1.00
0.99
1.00
1.00
1.08
1.00
1.00
1.00
0.95
0.96
3.00
2.00
3.00
1.01
2.00
2.04
6.11
9.02
9.5
9.0
8.5
8.0
7.5
7.0
6.5
6.0
5.5
5.0
4.5
4.0
3.5
3.0
2.5
2.0
1.5
1.0
Chemical Shift (ppm)

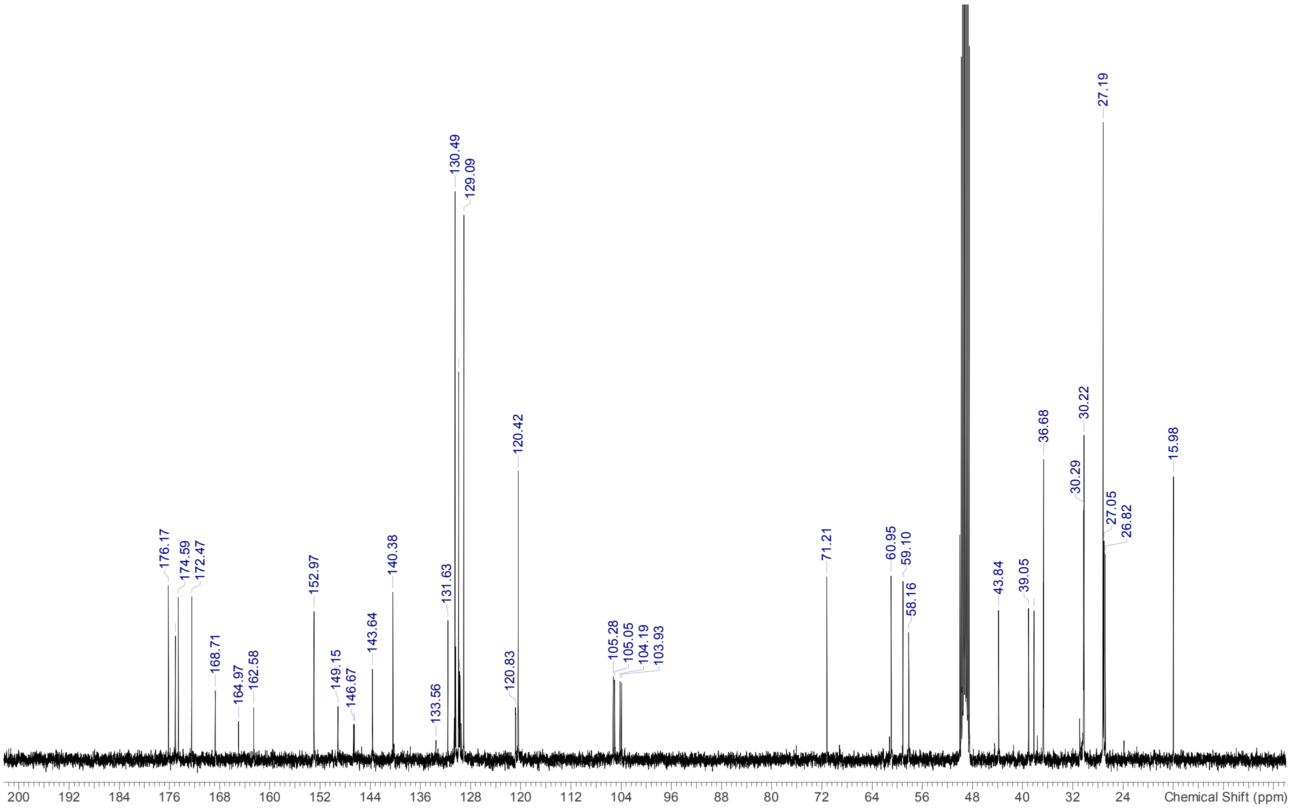
176.17
174.59
172.47
168.71
164.97
162.58
152.97
149.15
146.67
143.64
140.38
133.56
131.63
130.49
129.09
120.83
120.42
105.28
105.05
104.19
103.93
71.21
60.95
59.10
58.16
43.84
39.05
36.68
30.29
30.22
27.19
27.05
26.82
15.98
200
192
184
176
168
160
152
144
136
128
120
112
104
96
88
80
72
64
56
48
40
32
24
Chemical Shift (ppm)

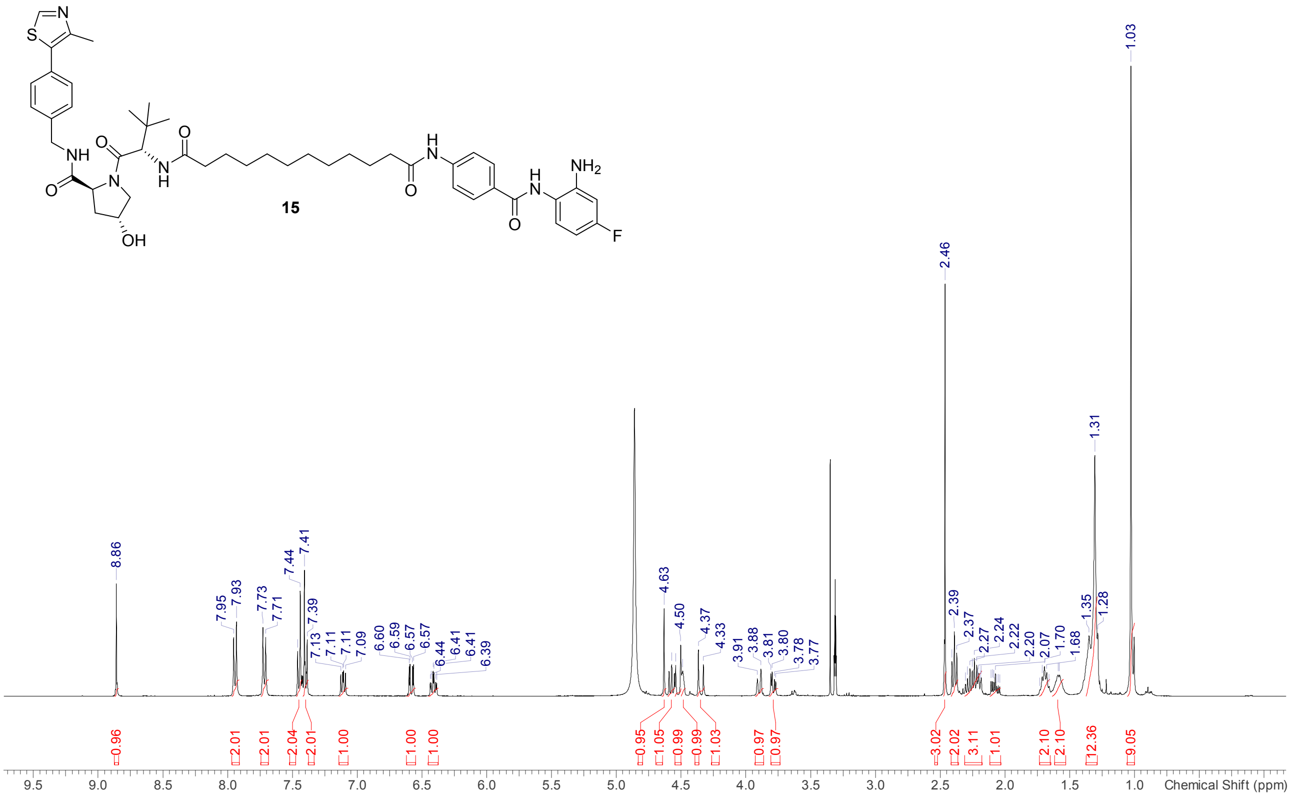
15
Chemical Shift (ppm)

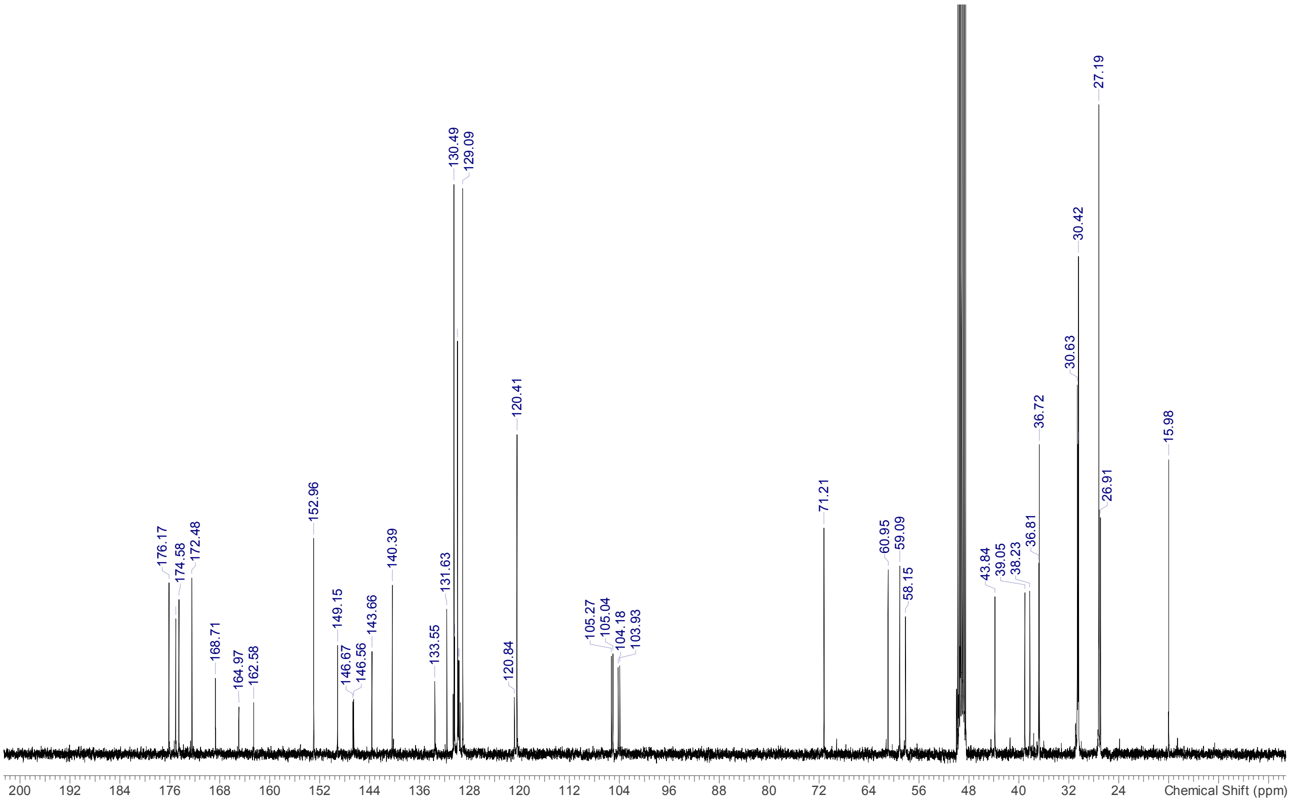
Chemical Shift (ppm)

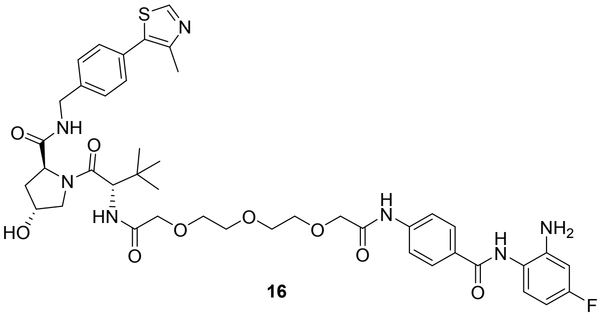
S
N
NH
O
O
N
HO
HN
O
O
O
O
H
N
O
H
N
NH_2
O
F

16

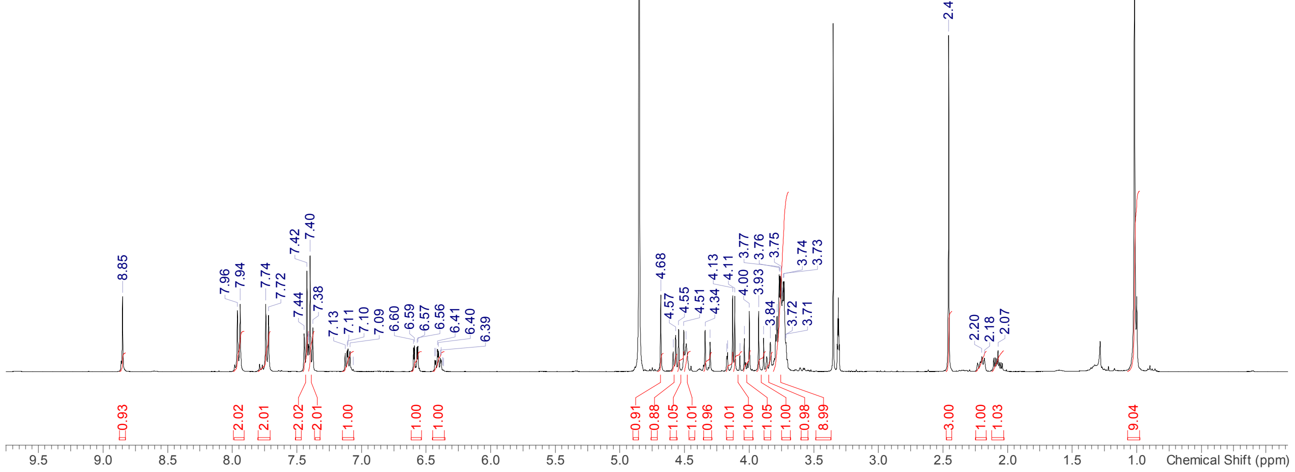
8.85
7.96
7.94
7.74
7.72
7.44
7.42
7.40
7.38
7.13
7.11
7.10
7.09
6.60
6.59
6.57
6.56
6.41
6.40
6.39
4.68
4.57
4.55
4.51
4.34
4.13
4.11
4.00
3.93
3.84
3.77
3.76
3.75
3.74
3.73
3.72
3.71
2.46
2.20
2.18
2.07
1.02
0.93
2.02
2.01
2.02
2.01
1.00
1.00
1.00
0.91
0.88
1.05
1.01
0.96
1.01
1.00
1.05
1.00
0.98
8.99
3.00
1.00
1.03
9.04
9.5 9.0 8.5 8.0 7.5 7.0 6.5 6.0 5.5 5.0 4.5 4.0 3.5 3.0 2.5 2.0 1.5 1.0
Chemical Shift (ppm)

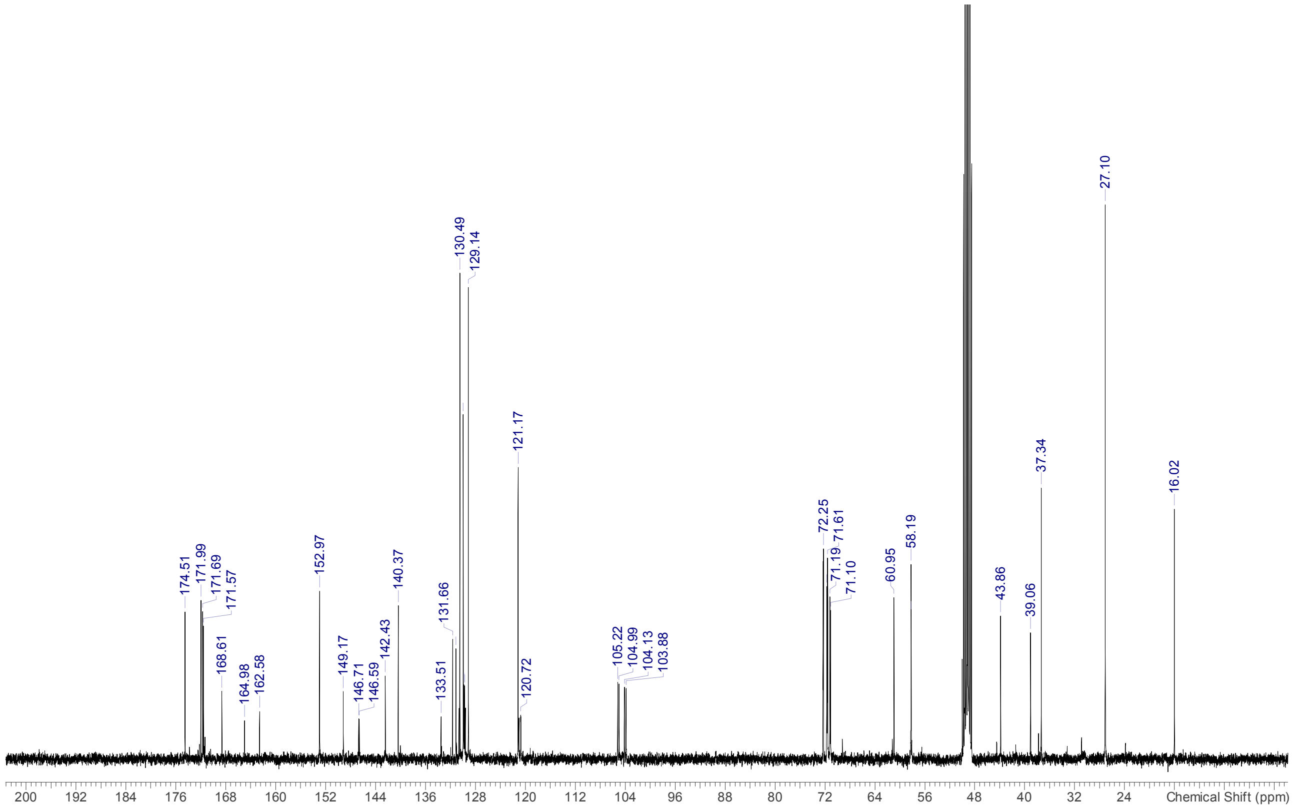
174.51
171.99
171.69
171.57
168.61
164.98
162.58
152.97
149.17
146.71
146.59
142.43
140.37
133.51
131.66
130.49
129.14
121.17
120.72
105.22
104.99
104.13
103.88
72.25
71.61
71.19
71.10
60.95
58.19
43.86
39.06
37.34
27.10
16.02
200 192 184 176 168 160 152 144 136 128 120 112 104 96 88 80 72 64 56 48 40 32 24
Chemical Shift (ppm)

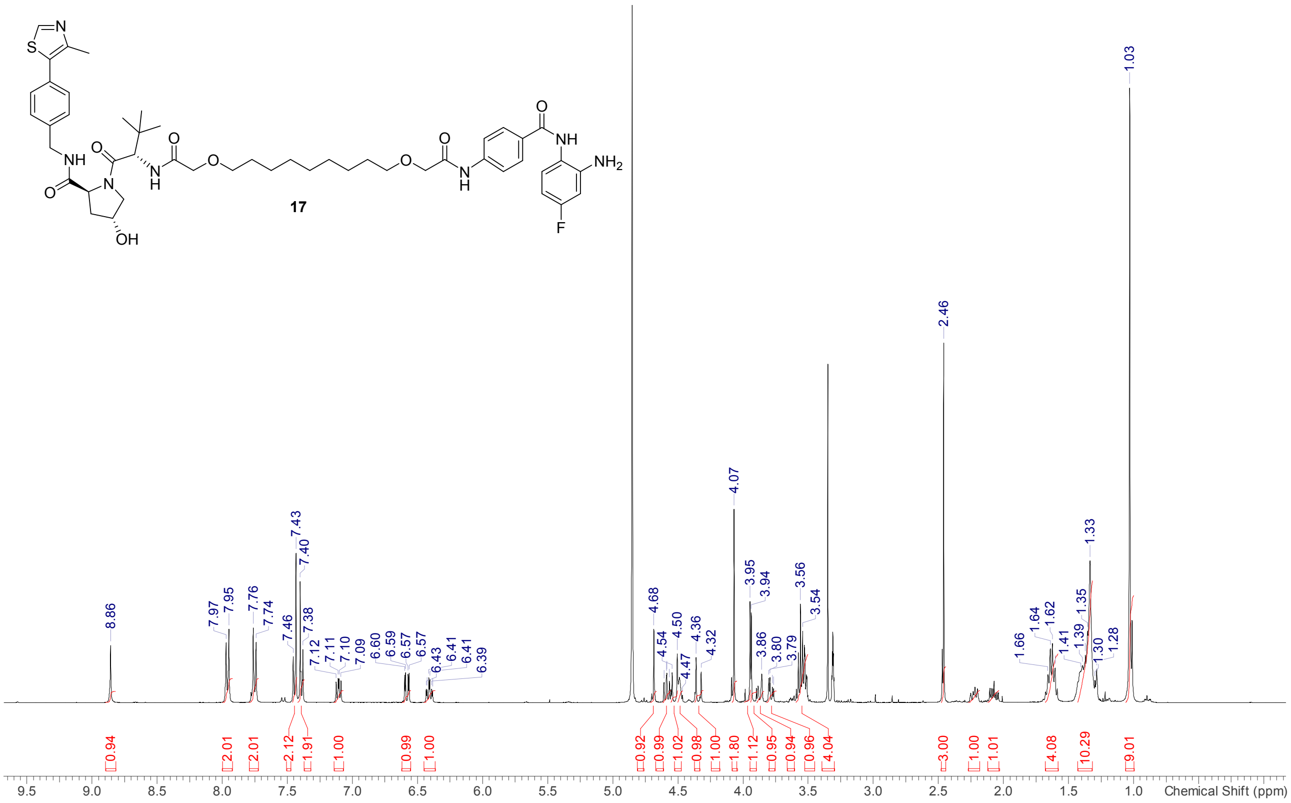

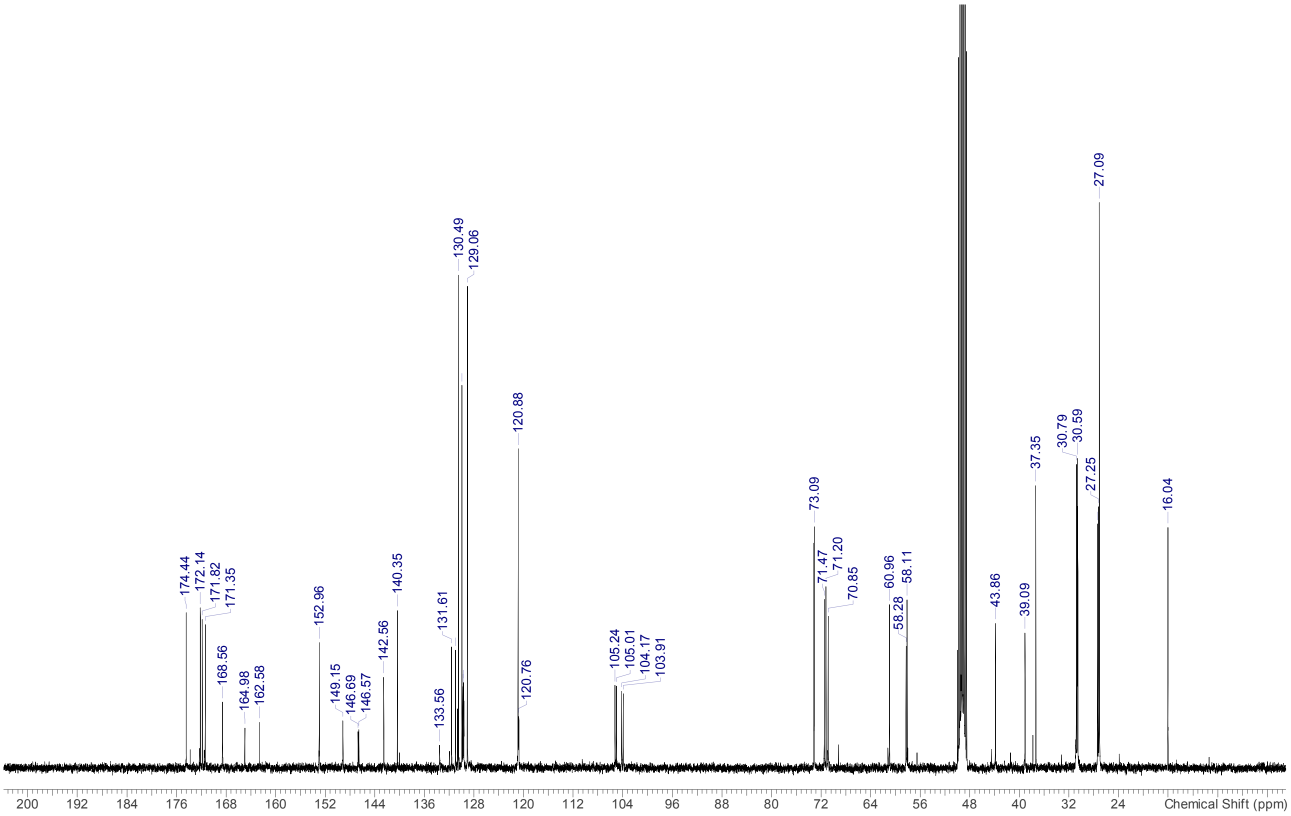

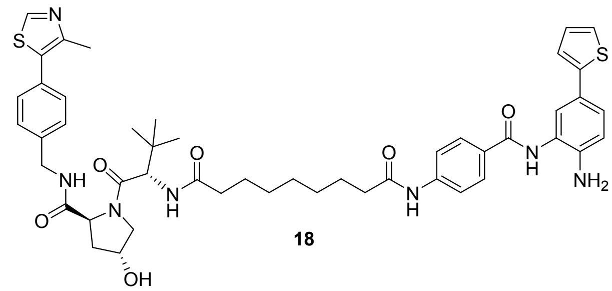

S
N
O
NH
N
OH
H
NH2

18

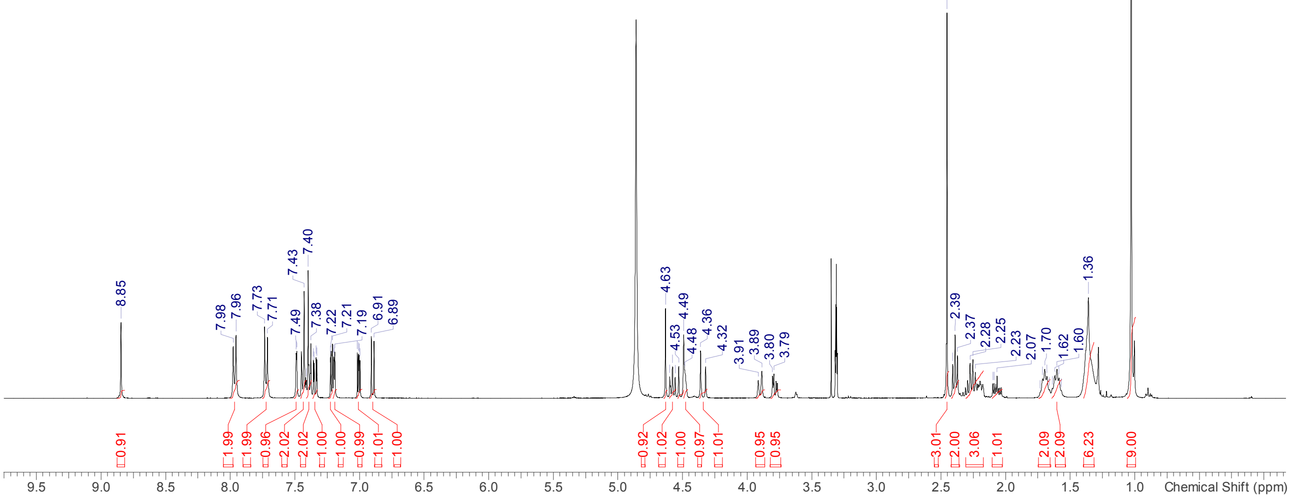

8.85
7.98
7.96
7.73
7.71
7.49
7.43
7.40
7.38
7.22
7.21
7.19
6.91
6.89
4.63
4.53
4.49
4.48
4.36
4.32
3.91
3.89
3.80
3.79
2.45
2.39
2.37
2.28
2.25
2.23
2.07
1.70
1.62
1.60
1.36
1.03
0.91
1.99
1.99
0.96
2.02
2.02
1.00
1.00
0.99
1.01
1.00
0.92
1.02
1.00
0.97
1.01
0.95
0.95
3.01
2.00
3.06
1.01
2.09
2.09
6.23
9.00
Chemical Shift (ppm)

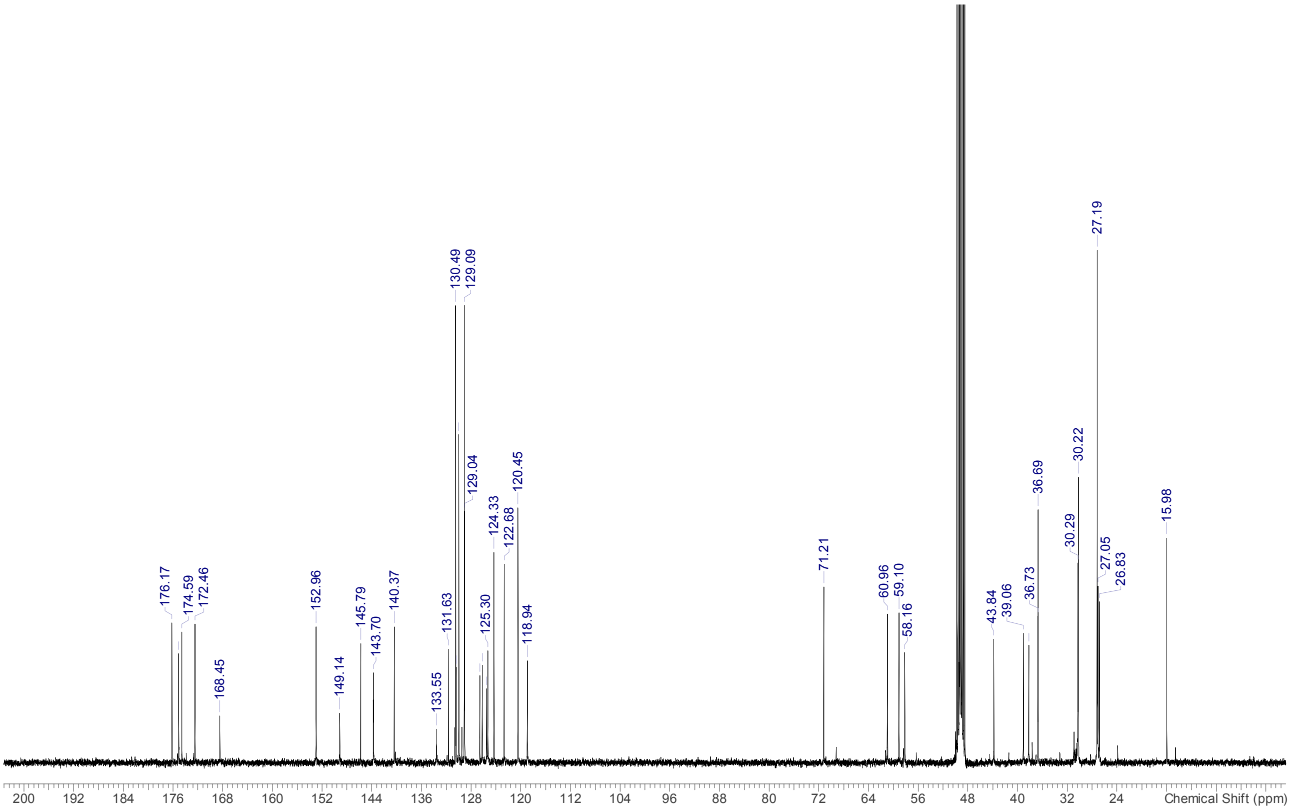

176.17
174.59
172.46
168.45
152.96
149.14
145.79
143.70
140.37
133.55
131.63
130.49
129.09
129.04
125.30
124.33
122.68
120.45
118.94
71.21
60.96
59.10
58.16
43.84
39.06
36.73
36.69
30.29
30.22
27.19
27.05
26.83
15.98
Chemical Shift (ppm)

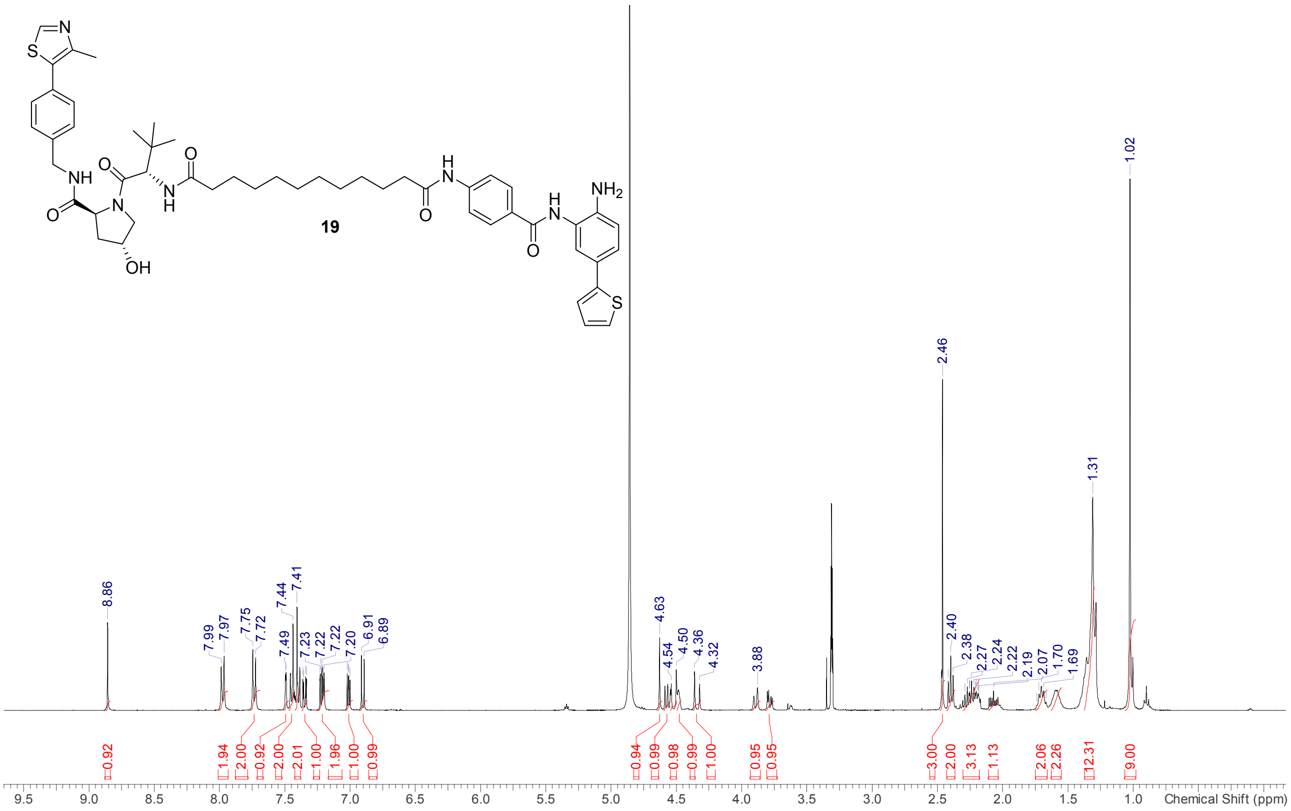
19
Chemical Shift (ppm)

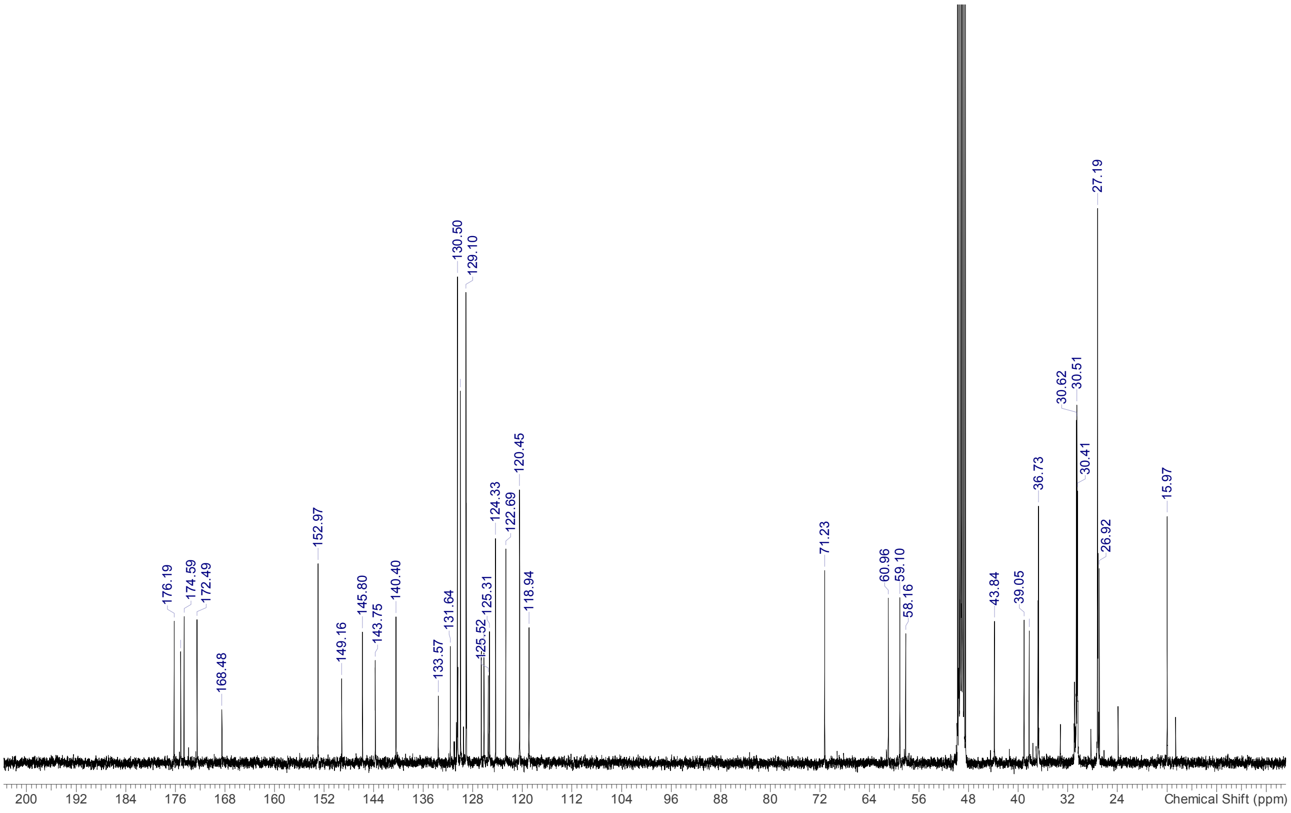
Chemical Shift (ppm)

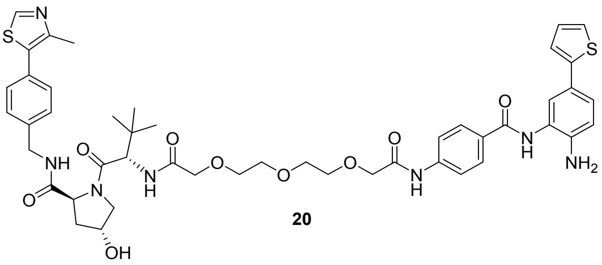
S
N
NH
O
N
H
O
OH
NH$_2$

20

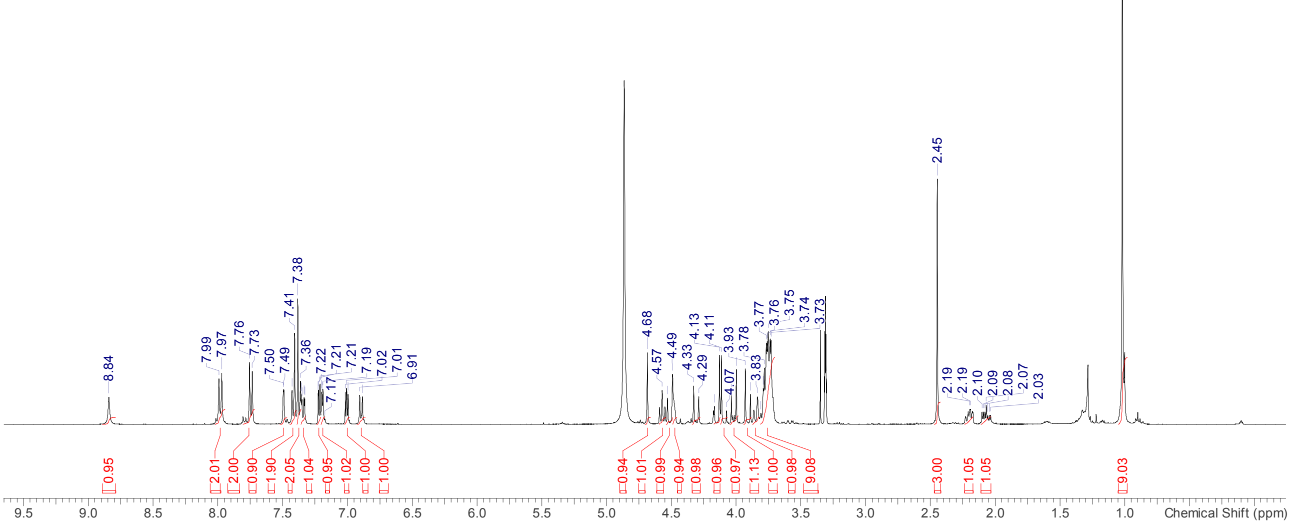
8.84
7.99
7.97
7.76
7.73
7.50
7.49
7.41
7.38
7.36
7.22
7.21
7.21
7.19
7.17
7.02
7.01
6.91
4.68
4.57
4.49
4.33
4.29
4.13
4.11
4.07
3.93
3.83
3.78
3.77
3.76
3.75
3.74
3.73
2.45
2.19
2.19
2.10
2.09
2.08
2.07
2.03
1.02
0.95
2.01
2.00
0.90
1.90
2.05
1.04
0.95
1.02
1.00
1.00
0.94
1.01
0.99
0.94
0.98
0.96
0.97
1.13
1.00
0.98
9.08
3.00
1.05
1.05
9.03
9.5 9.0 8.5 8.0 7.5 7.0 6.5 6.0 5.5 5.0 4.5 4.0 3.5 3.0 2.5 2.0 1.5 1.0
Chemical Shift (ppm)

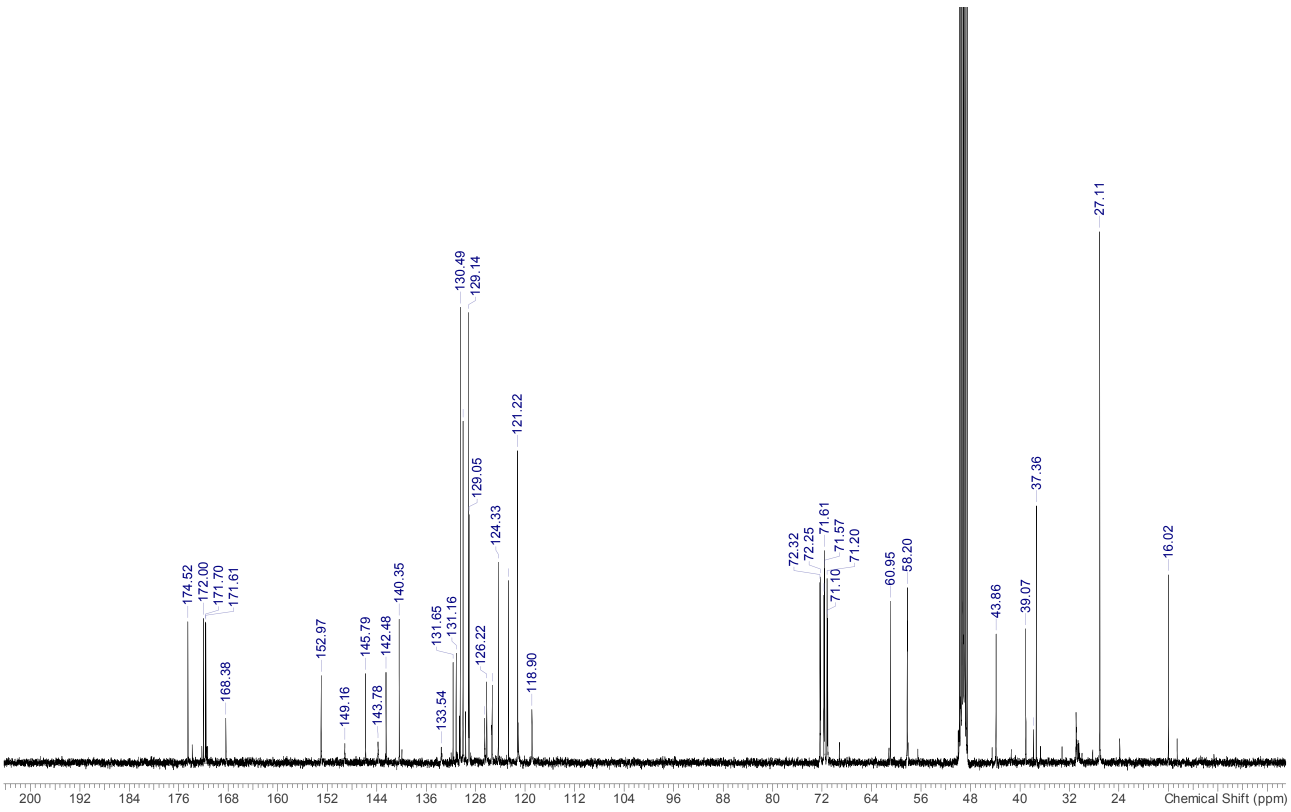
174.52
172.00
171.70
171.61
168.38
152.97
149.16
145.79
143.78
142.48
140.35
133.54
131.65
131.16
130.49
129.14
129.05
126.22
124.33
121.22
118.90
72.32
72.25
71.61
71.57
71.20
71.10
60.95
58.20
43.86
39.07
37.36
27.11
16.02
200 192 184 176 168 160 152 144 136 128 120 112 104 96 88 80 72 64 56 48 40 32 24
Chemical Shift (ppm)

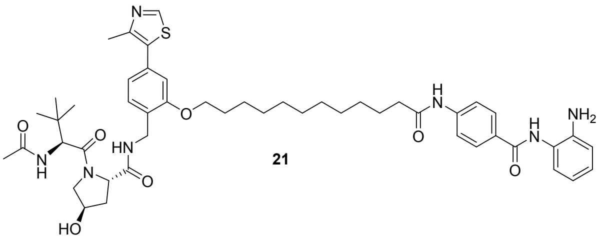

N
S
O
HN
NH2
H
N
O
O
HN
N
O
N
H
O
HO

21

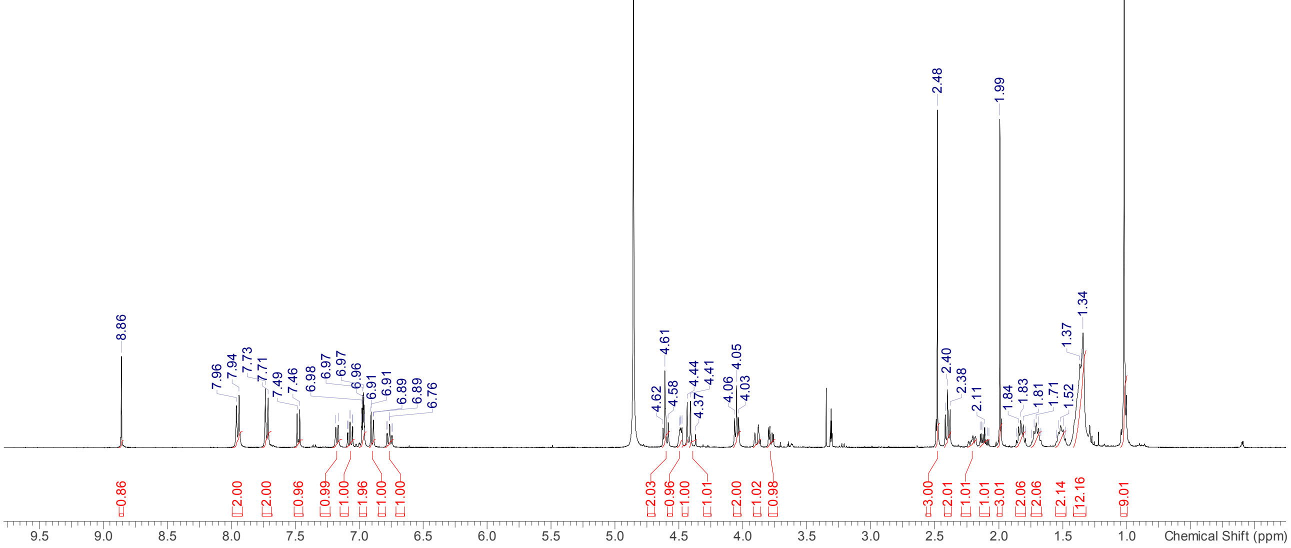

8.86
7.96
7.94
7.73
7.71
7.49
7.46
6.98
6.97
6.97
6.96
6.91
6.91
6.89
6.89
6.76
4.62
4.61
4.58
4.44
4.41
4.37
4.06
4.05
4.03
2.48
2.40
2.38
2.11
1.99
1.84
1.83
1.81
1.71
1.52
1.37
1.34
1.02
0.86
2.00
2.00
0.96
0.99
1.00
1.96
1.00
1.00
2.03
0.96
1.00
1.01
2.00
1.02
0.98
3.00
2.01
1.01
1.01
3.01
2.06
2.06
2.14
12.16
9.01
9.5 9.0 8.5 8.0 7.5 7.0 6.5 6.0 5.5 5.0 4.5 4.0 3.5 3.0 2.5 2.0 1.5 1.0
Chemical Shift (ppm)

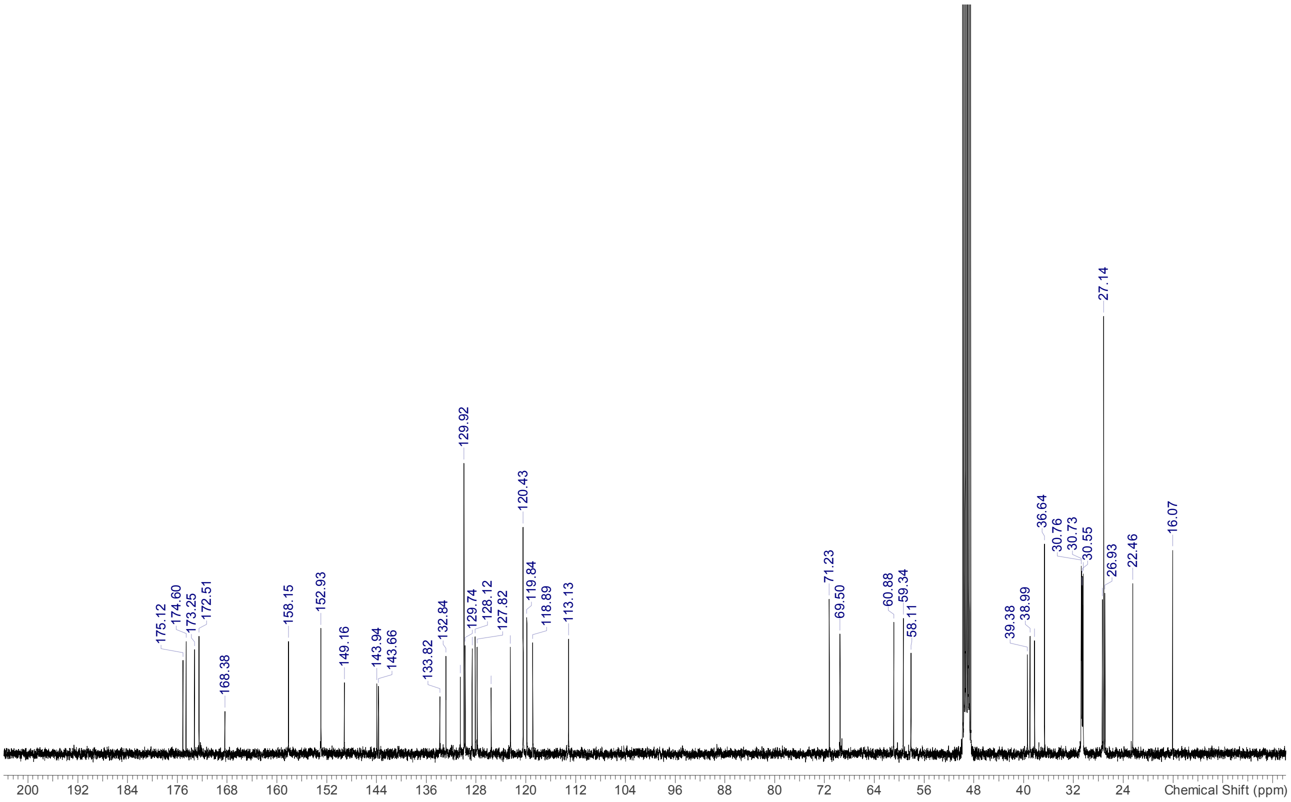

175.12
174.60
173.25
172.51
168.38
158.15
152.93
149.16
143.94
143.66
133.82
132.84
129.92
129.74
128.12
127.82
120.43
119.84
118.89
113.13
71.23
69.50
60.88
59.34
58.11
39.38
38.99
36.64
30.76
30.73
30.55
27.14
26.93
22.46
16.07
200 192 184 176 168 160 152 144 136 128 120 112 104 96 88 80 72 64 56 48 40 32 24
Chemical Shift (ppm)

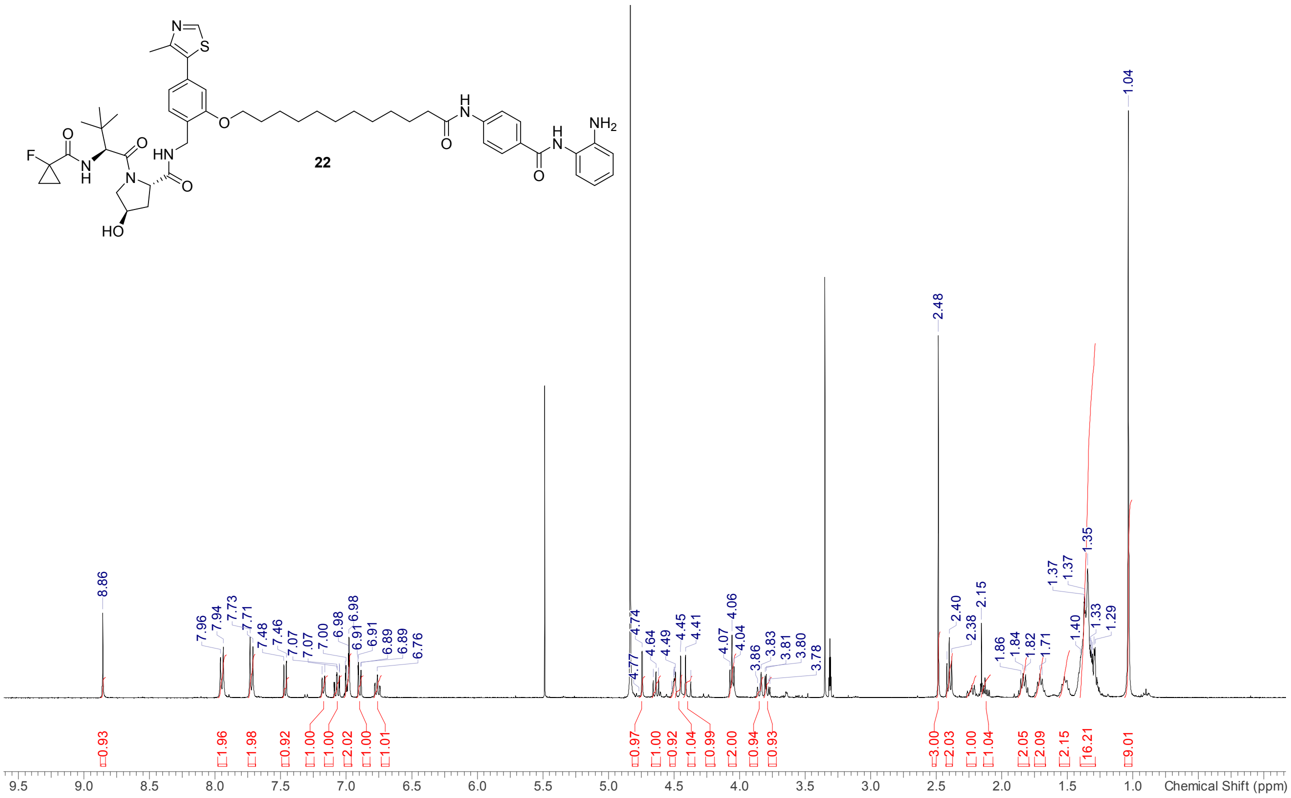
22
NH2
HO
F
8.86
7.96 7.94 7.73 7.71 7.48 7.46 7.07 7.07 7.00 6.98 6.98 6.91 6.91 6.89 6.89 6.76
4.77 4.74 4.64 4.49 4.45 4.41 4.07 4.06 4.04 3.86 3.83 3.81 3.80 3.78
2.48 2.40 2.38 2.15 1.86 1.84 1.82 1.71 1.40 1.37 1.37 1.35 1.33 1.29 1.04
0.93 1.96 1.98 0.92 1.00 1.00 2.02 1.00 1.01 0.97 1.00 0.92 1.04 0.99 2.00 0.94 0.93 3.00 2.03 1.00 1.04 2.05 2.09 2.15 16.21 9.01
Chemical Shift (ppm)

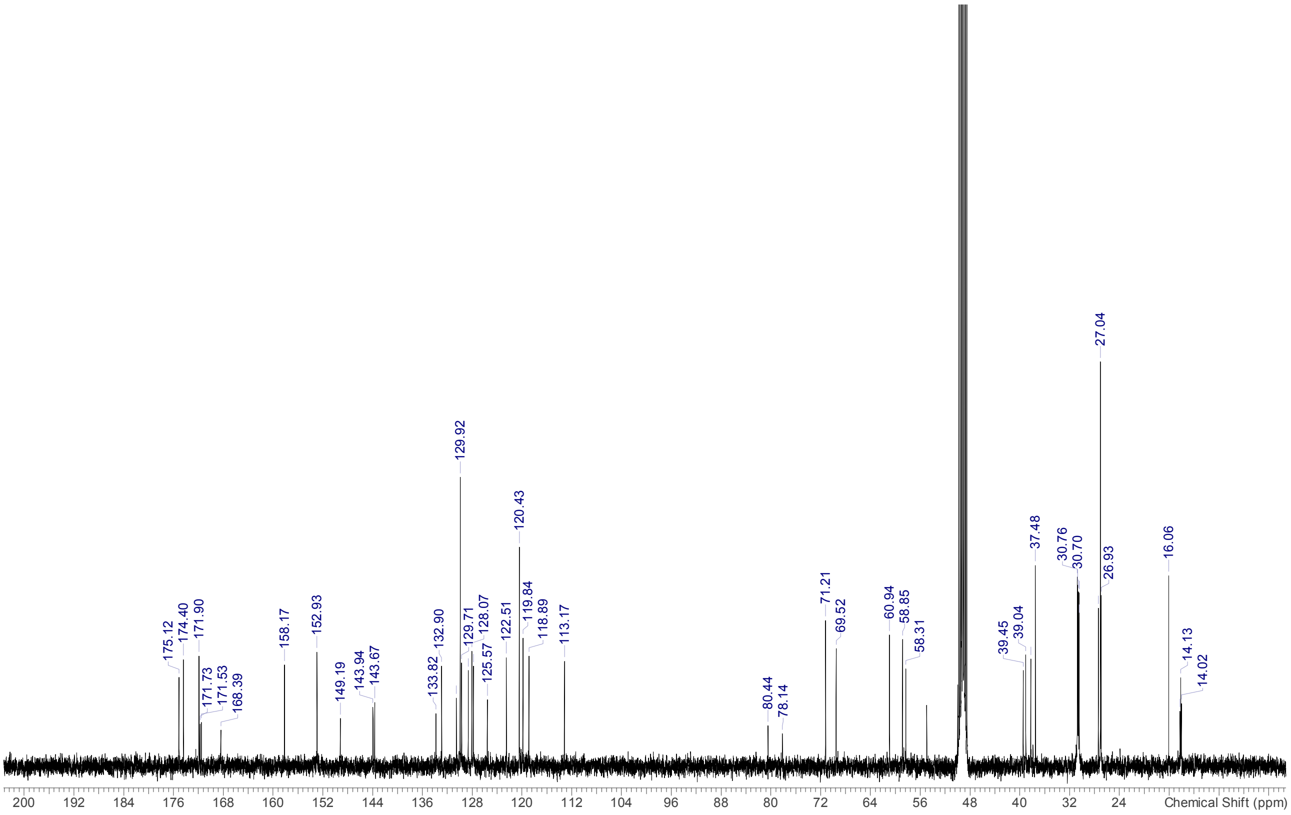
175.12 174.40 171.90 171.73 171.53 168.39 158.17 152.93 149.19 143.94 143.67
133.82 132.90 129.92 129.71 128.07 125.57 122.51 120.43 119.84 118.89 113.17
80.44 78.14 71.21 69.52 60.94 58.85 58.31
39.45 39.04 37.48 30.76 30.70 27.04 26.93 16.06 14.13 14.02
Chemical Shift (ppm)

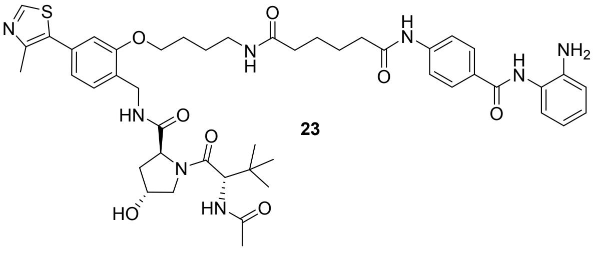
S
N
O
N
H
O
O
H
N
NH2
H
N
O
HN
O
O
N
HO
HN
O

23

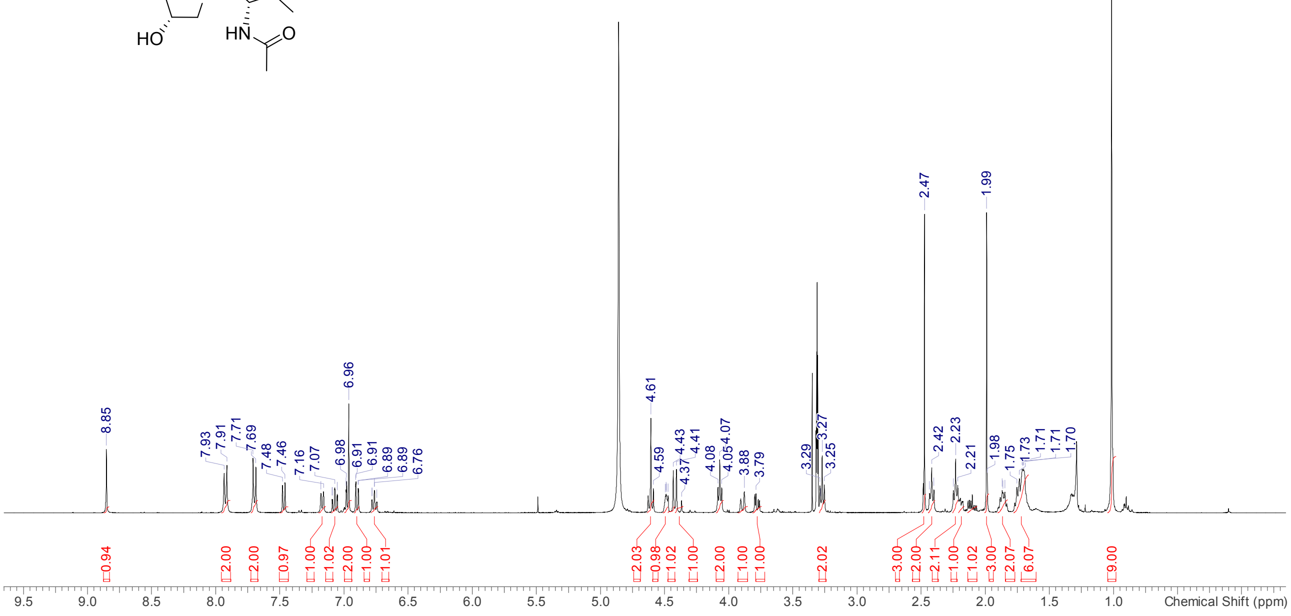
8.85
7.93
7.91
7.71
7.69
7.48
7.46
7.16
7.07
6.98
6.96
6.91
6.91
6.89
6.89
6.76
4.61
4.59
4.43
4.41
4.37
4.08
4.07
4.05
3.88
3.79
3.29
3.27
3.25
2.47
2.42
2.23
2.21
1.99
1.98
1.75
1.73
1.71
1.71
1.70
1.01
0.94
2.00
2.00
0.97
1.00
1.02
2.00
1.00
1.01
2.03
0.98
1.02
1.00
2.00
1.00
1.00
2.02
3.00
2.00
2.11
1.00
1.02
3.00
2.07
6.07
9.00
9.5
9.0
8.5
8.0
7.5
7.0
6.5
6.0
5.5
5.0
4.5
4.0
3.5
3.0
2.5
2.0
1.5
1.0
Chemical Shift (ppm)

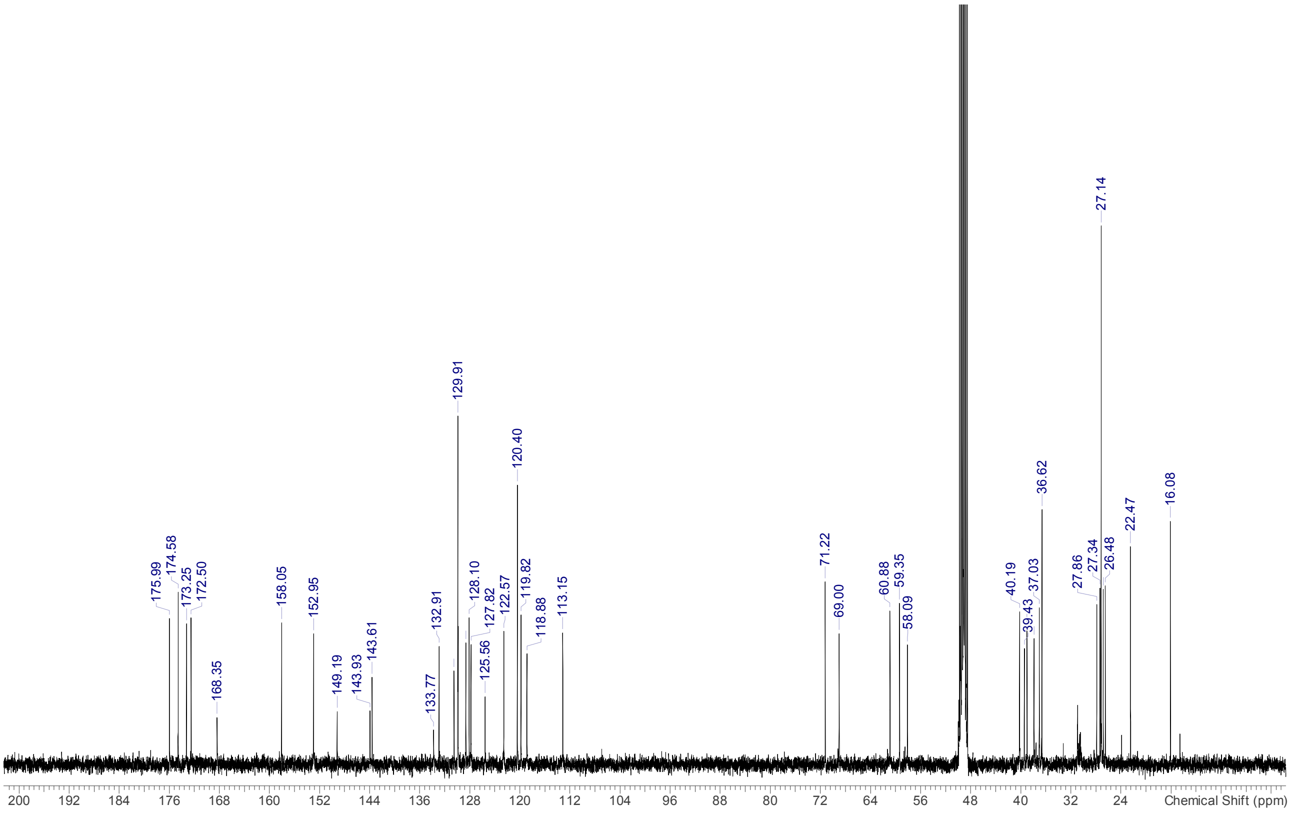
175.99
174.58
173.25
172.50
168.35
158.05
152.95
149.19
143.93
143.61
133.77
132.91
129.91
128.10
127.82
125.56
122.57
120.40
119.82
118.88
113.15
71.22
69.00
60.88
59.35
58.09
40.19
39.43
37.03
36.62
27.86
27.34
27.14
26.48
22.47
16.08
200
192
184
176
168
160
152
144
136
128
120
112
104
96
88
80
72
64
56
48
40
32
24
Chemical Shift (ppm)

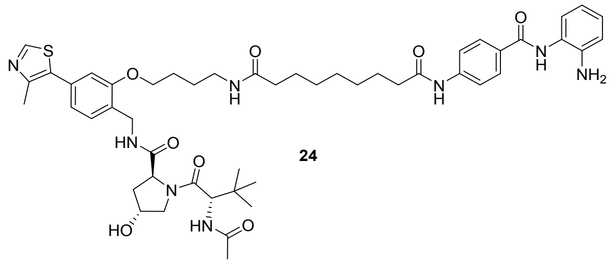

S
N
O
O
N
H
O
N
H
O
N
H
NH2
HN
O
N
O
HO
HN
O

24

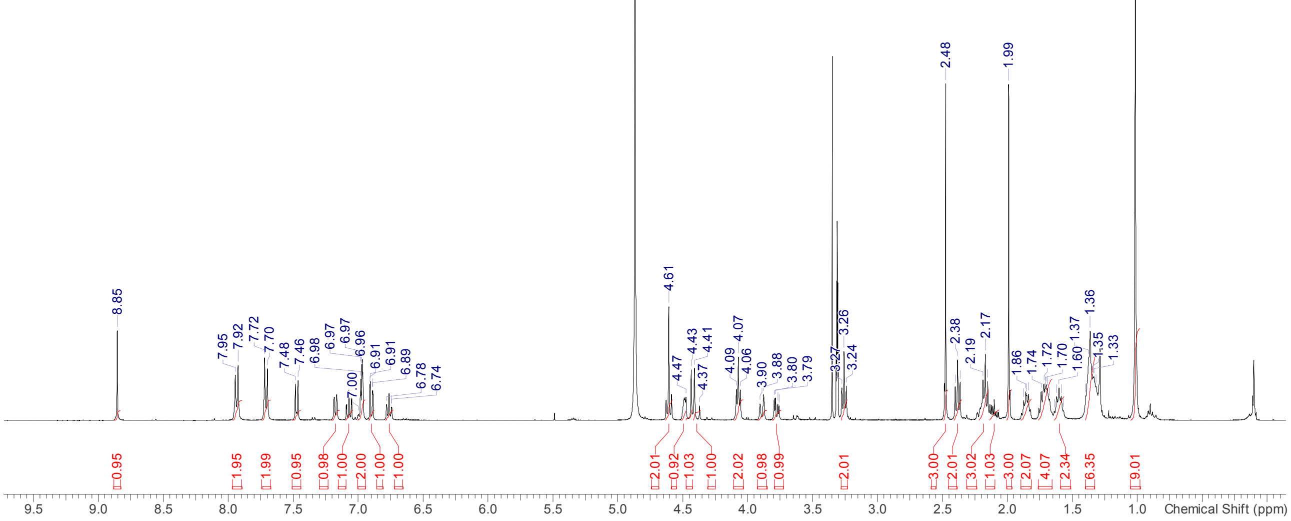

8.85
7.95
7.92
7.72
7.70
7.48
7.46
6.98
6.97
6.97
6.96
7.00
6.91
6.91
6.89
6.78
6.74
4.61
4.47
4.43
4.41
4.37
4.09
4.07
4.06
3.90
3.88
3.80
3.79
3.27
3.26
3.24
2.48
2.38
2.19
2.17
1.99
1.86
1.74
1.72
1.70
1.60
1.37
1.36
1.35
1.33
1.01
0.95
1.95
1.99
0.95
0.98
1.00
2.00
1.00
1.00
2.01
0.92
1.03
1.00
2.02
0.98
0.99
2.01
3.00
2.01
3.02
1.03
3.00
2.07
4.07
2.34
6.35
9.01
9.5
9.0
8.5
8.0
7.5
7.0
6.5
6.0
5.5
5.0
4.5
4.0
3.5
3.0
2.5
2.0
1.5
1.0
Chemical Shift (ppm)

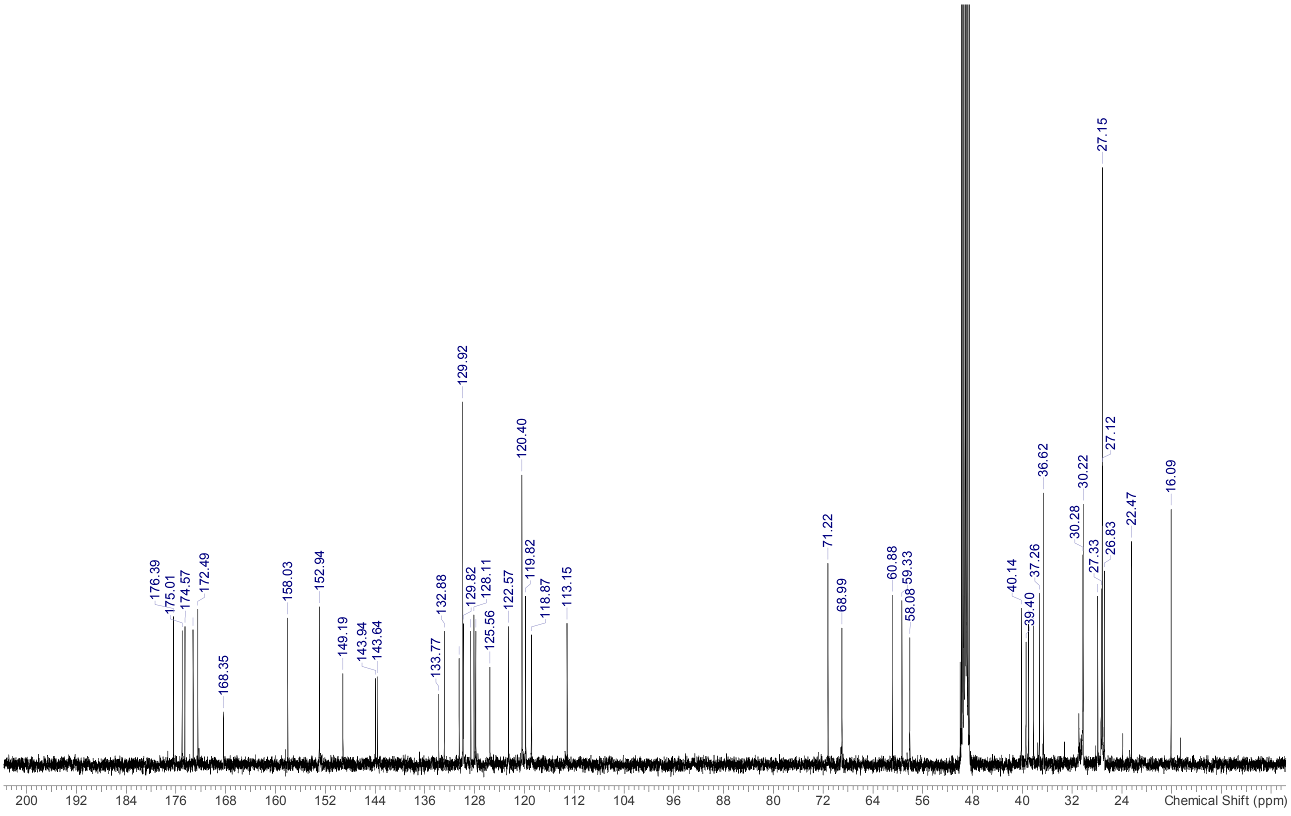

176.39
175.01
174.57
172.49
168.35
158.03
152.94
149.19
143.94
143.64
133.77
132.88
129.92
129.82
128.11
125.56
122.57
120.40
119.82
118.87
113.15
71.22
68.99
60.88
59.33
58.08
40.14
39.40
37.26
36.62
30.28
30.22
27.33
27.15
27.12
26.83
22.47
16.09
200
192
184
176
168
160
152
144
136
128
120
112
104
96
88
80
72
64
56
48
40
32
24
Chemical Shift (ppm)

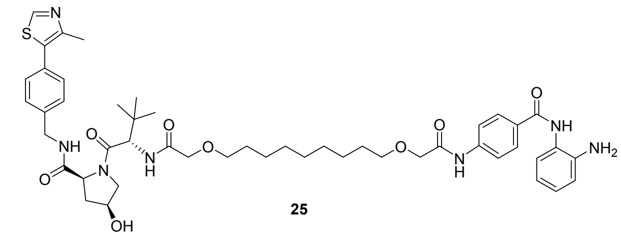

S
N
NH
O
O
N
OH
N
H
O
O
O
O
N
H
O
NH
NH2

25

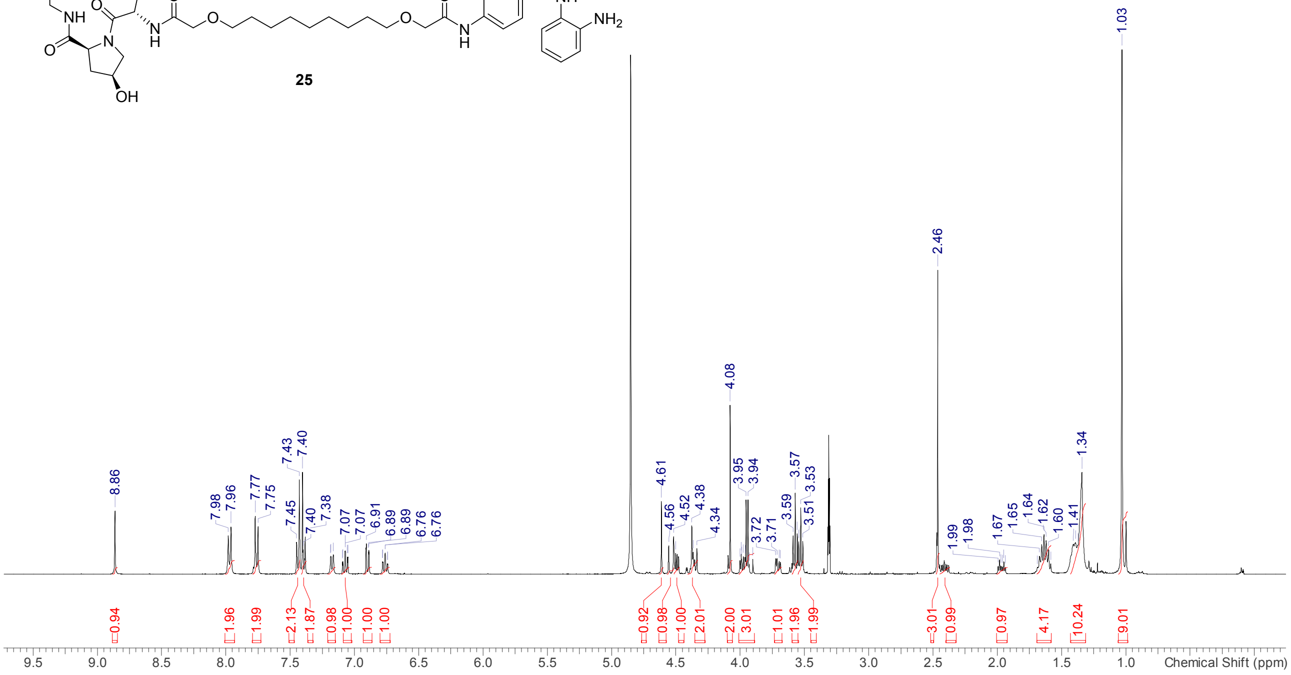

8.86
7.98 7.96
7.77 7.75
7.45 7.43 7.40 7.40 7.38
7.07 7.07
6.91 6.89 6.89
6.76 6.76
4.61
4.56 4.52
4.38 4.34
4.08
3.95 3.94
3.72 3.71
3.59 3.57 3.53 3.51
2.46
1.99 1.98
1.67 1.65 1.64 1.62 1.60
1.41 1.34
1.03
0.94 1.96 1.99 2.13 1.87 0.98 1.00 1.00 1.00
0.92 0.98 1.00 2.01 2.00 3.01 1.01 1.96 1.99
3.01 0.99 0.97 4.17 10.24 9.01
9.5 9.0 8.5 8.0 7.5 7.0 6.5 6.0 5.5 5.0 4.5 4.0 3.5 3.0 2.5 2.0 1.5 1.0
Chemical Shift (ppm)

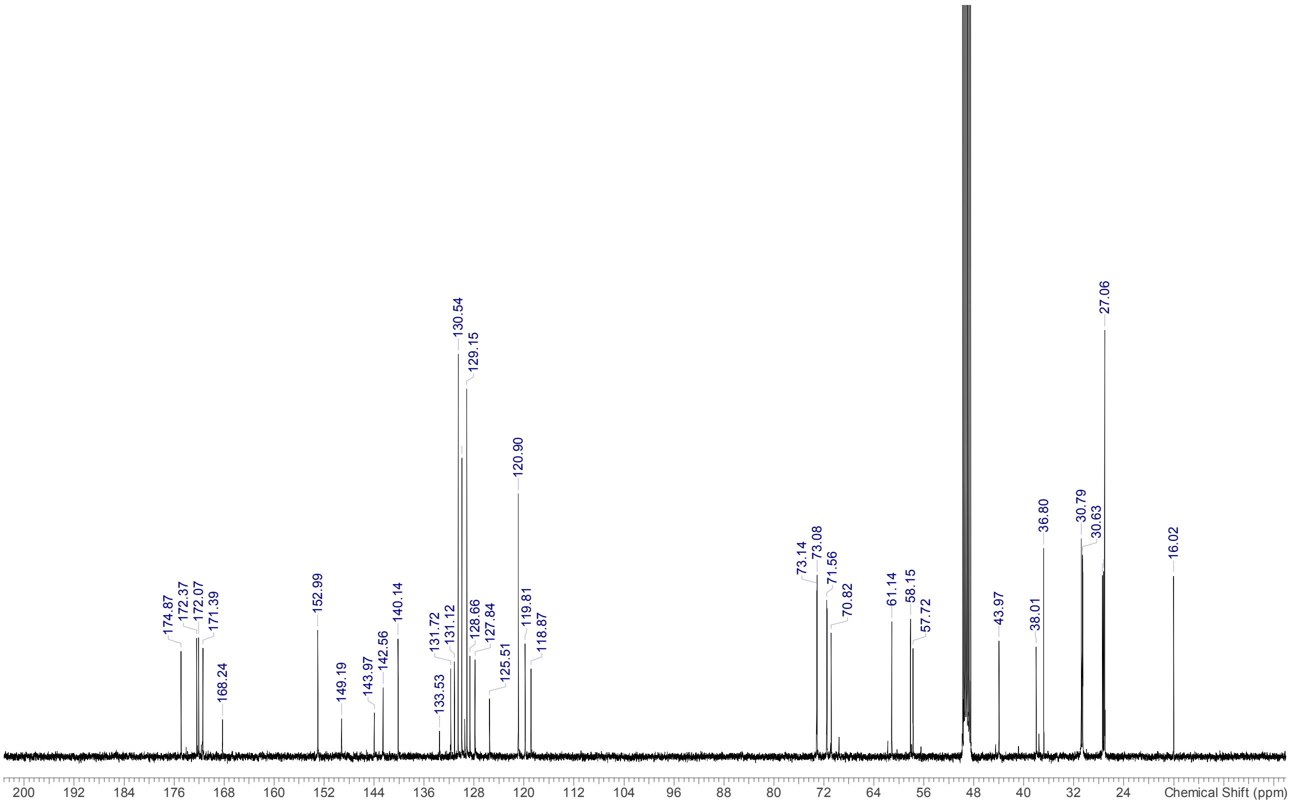

174.87 172.37 172.07 171.39
168.24
152.99
149.19
143.97 142.56
140.14
133.53
131.72 131.12
130.54 129.15
128.66 127.84
125.51
120.90
119.81 118.87
73.14 73.08
71.56
70.82
61.14
58.15 57.72
43.97
38.01
36.80
30.79 30.63
27.06
16.02
200 192 184 176 168 160 152 144 136 128 120 112 104 96 88 80 72 64 56 48 40 32 24
Chemical Shift (ppm)
